# THE CONCISE GUIDE TO PHARMACOLOGY 2019/20: G protein‐coupled receptors

**DOI:** 10.1111/bph.14748

**Published:** 2019-11-11

**Authors:** Stephen PH Alexander, Arthur Christopoulos, Anthony P Davenport, Eamonn Kelly, Alistair Mathie, John A Peters, Emma L Veale, Jane F Armstrong, Elena Faccenda, Simon D Harding, Adam J Pawson, Joanna L Sharman, Christopher Southan, Jamie A Davies, CGTP Collaborators, Maria P Abbracchio, Wayne Alexander, Khaled Al-hosaini, Magnus Bäck, Jean‐Martin Beaulieu, Kenneth E. Bernstein, Bernhard Bettler, Nigel JM Birdsall, Victoria Blaho, Corinne Bousquet, Hans Bräuner-Osborne, Geoffrey Burnstock, Girolamo Caló, Justo P Castaño, Kevin J Catt, Stefania Ceruti, Paul Chazot, Nan Chiang, Jerold Chun, Antonia Cianciulli, Lucie H Clapp, Réjean Couture, Zsolt Csaba, Gordon Dent, Khuraijam Dhanachandra Singh, Steven D Douglas, Pascal Dournaud, Satoru Eguchi, Emanuel Escher, Edward Filardo, Tung M Fong, Marta Fumagalli, Raul R Gainetdinov, Marc de Gasparo, Marvin Gershengorn, Fernand Gobeil, Theodore L Goodfriend, Cyril Goudet, Karen J Gregory, Andrew L Gundlach, Jörg Hamann, Julien Hanson, Richard L Hauger, Debbie Hay, Akos Heinemann, Morley D Hollenberg, Nicholas D Holliday, Mastgugu Horiuchi, Daniel Hoyer, László Hunyady, Ahsan Husain, Adriaan P Ijzerman, Tadashi Inagami, Kenneth A Jacobson, Robert T Jensen, Ralf Jockers, Deepa Jonnalagadda, Sadashiva Karnik, Klemens Kaupmann, Jacqueline Kemp, Charles Kennedy, Yasuyuki Kihara, Paweł Kozielewicz, Hans‐Jürgen Kreienkamp, Jyrki P Kukkonen, Tobias Langenhan, Katie Leach, Davide Lecca, John D Lee, Susan E Leeman, Jérôme Leprince, Stephen J Lolait, Amelie Lupp, Robyn Macrae, Janet Maguire, Jean Mazella, Craig A McArdle, Shlomo Melmed, Martin C Michel, Laurence Miller, Vincenzo Mitolo, Bernard Mouillac, Philip M Murphy, Jean‐Louis Nahon, Xavier Norel, Duuamene Nyimanu, Anne‐Marie O'Carroll, Stefan Offermanns, Maria A Panaro, Roger G Pertwee, Jean‐Philippe Pin, Eric Prossnitz, Rithwik Ramachandran, Rainer K Reinscheid, Philippe Rondard, G Enrico Rovati, Chiara Ruzza, Gareth Sanger, Torsten Schöneberg, Gunnar Schulte, Stefan Schulz, Deborah L Segaloff, Charles N Serhan, Leigh A Stoddart, Yukihiko Sugimoto, Roger Summers, Valerie Tan, Walter Thomas, Pieter BMWM Timmermans, Kalyan Tirupula, Giovanni Tulipano, Hamiyet Unal, Thomas Unger, Patrick Vanderheyden, David Vaudry, Hubert Vaudry, Jean‐Pierre Vilardaga, Christopher S Walker, Donald T Ward, Hans‐Jürgen Wester, Gary B Willars, Tom Lloyd Williams, Trent M Woodruff, Chengcan Yao

**Affiliations:** ^1^ School of Life Sciences University of Nottingham Medical School Nottingham NG7 2UH UK; ^2^ Monash Institute of Pharmaceutical Sciences and Department of Pharmacology Monash University Parkville Victoria 3052 Australia; ^3^ Clinical Pharmacology Unit University of Cambridge Cambridge CB2 0QQ UK; ^4^ School of Physiology, Pharmacology and Neuroscience University of Bristol Bristol BS8 1TD UK; ^5^ Medway School of Pharmacy The Universities of Greenwich and Kent at Medway Anson Building, Central Avenue, Chatham Maritime Chatham, Kent ME4 4TB UK; ^6^ Neuroscience Division, Medical Education Institute, Ninewells Hospital and Medical School University of Dundee Dundee DD1 9SY UK; ^7^ Centre for Discovery Brain Sciences University of Edinburgh Edinburgh EH8 9XD UK; ^8^ Milan Italy; ^9^ Atlanta USA; ^10^ Riyadh Saudi Arabia; ^11^ Stockholm Sweden; ^12^ Toronto Canada; ^13^ Basel Switzerland; ^14^ London UK; ^15^ La Jolla USA; ^16^ Toulouse France; ^17^ Copenhagen Denmark; ^18^ Melbourne Australia; ^19^ Ferrara Italy; ^20^ Córdoba Spain; ^21^ Bethesda USA; ^22^ Durham UK; ^23^ Boston USA; ^24^ Bari Italy; ^25^ Montréal Canada; ^26^ Paris France; ^27^ Keele UK; ^28^ Cleveland USA; ^29^ Philadelphia USA; ^30^ Sherbrooke Canada; ^31^ Iowa City USA; ^32^ Princeton USA; ^33^ Saint Petersburg Russia; ^34^ Madison USA; ^35^ Montpellier France; ^36^ Parkville Australia; ^37^ Amsterdam The Netherlands; ^38^ Liège Belgium; ^39^ Auckland New Zealand; ^40^ Graz Austria; ^41^ Calgary Canada; ^42^ Nottingham UK; ^43^ Ehime Japan; ^44^ Budapest Hungary; ^45^ Birmingham USA; ^46^ Leiden The Netherlands; ^47^ Nashville USA; ^48^ Glasgow UK; ^49^ Hamburg Germany; ^50^ Helsinki Finland; ^51^ Leipzig Germany; ^52^ Brisbane Australia; ^53^ Rouen France; ^54^ Bristol UK; ^55^ Jena Germany; ^56^ Cambridge UK; ^57^ Valbonne France; ^58^ Los Angeles USA; ^59^ Mainz Germany; ^60^ Scottsdale USA; ^61^ Bad Nauheim Germany; ^62^ Aberdeen UK; ^63^ Albuquerque USA; ^64^ London Canada; ^65^ Munster Germany; ^66^ Kumamoto Japan; ^67^ Queensland Australia; ^68^ San Francisco USA; ^69^ Brescia Italy; ^70^ Kiel Germany; ^71^ Brussels Belgium; ^72^ Pittsburgh USA; ^73^ Manchester UK; ^74^ Munich Germany; ^75^ Leicester UK; ^76^ Edinburgh UK

## Abstract

The Concise Guide to PHARMACOLOGY 2019/20 is the fourth in this series of biennial publications. The Concise Guide provides concise overviews of the key properties of nearly 1800 human drug targets with an emphasis on selective pharmacology (where available), plus links to the open access knowledgebase source of drug targets and their ligands (http://www.guidetopharmacology.org/), which provides more detailed views of target and ligand properties. Although the Concise Guide represents approximately 400 pages, the material presented is substantially reduced compared to information and links presented on the website. It provides a permanent, citable, point‐in‐time record that will survive database updates. The full contents of this section can be found at http://onlinelibrary.wiley.com/doi/10.1111/bph.14748. G protein‐coupled receptors are one of the six major pharmacological targets into which the Guide is divided, with the others being: ion channels, nuclear hormone receptors, catalytic receptors, enzymes and transporters. These are presented with nomenclature guidance and summary information on the best available pharmacological tools, alongside key references and suggestions for further reading. The landscape format of the Concise Guide is designed to facilitate comparison of related targets from material contemporary to mid‐2019, and supersedes data presented in the 2017/18, 2015/16 and 2013/14 Concise Guides and previous Guides to Receptors and Channels. It is produced in close conjunction with the International Union of Basic and Clinical Pharmacology Committee on Receptor Nomenclature and Drug Classification (NC‐IUPHAR), therefore, providing official IUPHAR classification and nomenclature for human drug targets, where appropriate.

1

### Conflict of interest

The authors state that there are no conflicts of interest to disclose.

#### Overview

G protein‐coupled receptors (GPCRs) are the largest class of membrane proteins in the human genome. The term "7TM receptor" is commonly used interchangeably with "GPCR", although there are some receptors with seven transmembrane domains that do not signal through G proteins. GPCRs share a common architecture, each consisting of a single polypeptide with an extracellular N‐terminus, an intracellular C‐terminus and seven hydrophobic transmembrane domains (TM1‐TM7) linked by three extracellular loops (ECL1‐ECL3) and three intracellular loops (ICL1‐ICL3). About 800 GPCRs have been identified in man, of which about half have sensory functions, mediating olfaction (˜400), taste (33), light perception (10) and pheromone signalling (5) [http://www.ncbi.nlm.nih.gov/pubmed/15034552?dopt=AbstractPlus]. The remaining 350 non‐sensory GPCRs mediate signalling by ligands that range in size from small molecules to peptides to large proteins; they are the targets for the majority of drugs in clinical usage [http://www.ncbi.nlm.nih.gov/pubmed/17139284?dopt=AbstractPlus, http://www.ncbi.nlm.nih.gov/pubmed/24016212?dopt=AbstractPlus], although only a minority of these receptors are exploited therapeutically. The first classification scheme to be proposed for GPCRs [http://www.ncbi.nlm.nih.gov/pubmed/8081729?dopt=AbstractPlus] divided them, on the basic of sequence homology, into six classes. These classes and their prototype members were as follows: **Class A** (rhodopsin‐like), **Class B** (secretin receptor family), **Class C** (metabotropic glutamate), **Class D** (fungal mating pheromone receptors), **Class E** (cyclic AMP receptors) and **Class F** (frizzled/smoothened). Of these, classes D and E are not found in vertebrates. An alternative classification scheme "GRAFS" [http://www.ncbi.nlm.nih.gov/pubmed/15862553?dopt=AbstractPlus] divides vertebrate GPCRs into five classes, overlapping with the A‐F nomenclature, *viz*:

**Glutamate family** (http://www.guidetopharmacology.org/GRAC/GPCRListForward?class=C), which includes metabotropic glutamate receptors, a calcium‐sensing receptor and GABA_B_ receptors, as well as three taste type 1 receptors and a family of pheromone receptors (V2 receptors) that are abundant in rodents but absent in man [http://www.ncbi.nlm.nih.gov/pubmed/15034552?dopt=AbstractPlus].

**Rhodopsin family** (http://www.guidetopharmacology.org/GRAC/GPCRListForward?class=A), which includes receptors for a wide variety of small molecules, neurotransmitters, peptides and hormones, together with olfactory receptors, visual pigments, taste type 2 receptors and five pheromone receptors (V1 receptors).

http://www.guidetopharmacology.org/GRAC/GPCRListForward?class=Adhesion GPCRs are phylogenetically related to class B receptors, from which they differ by possessing large extracellular N‐termini that are autoproteolytically cleaved from their 7TM domains at a conserved "GPCR proteolysis site" (GPS) which lies within a much larger (320 residue) "GPCR autoproteolysis‐inducing" (GAIN) domain, an evolutionary ancient mofif also found in polycystic kidney disease 1 (PKD1)‐like proteins, which has been suggested to be both required and sufficient for autoproteolysis [http://www.ncbi.nlm.nih.gov/pubmed/23850273?dopt=AbstractPlus].

http://www.guidetopharmacology.org/GRAC/GPCRListForward?class=Frizzled consists of 10 Frizzled proteins (FZD(1‐10)) and Smoothened (SMO). The FZDs are activated by secreted lipoglycoproteins of the WNT family, whereas SMO is indirectly activated by the Hedgehog (HH) family of proteins acting on the transmembrane protein Patched (PTCH).

**Secretin family,** encoded by 15 genes in humans. The ligands for receptors in this family are polypeptide hormones of 27‐141 amino acid residues; nine of the mammalian receptors respond to ligands that are structurally related to one another (glucagon, glucagon‐like peptides (GLP‐1, GLP‐2), glucose‐dependent insulinotropic polypeptide (GIP), secretin, vasoactive intestinal peptide (VIP), pituitary adenylate cyclase‐activating polypeptide (PACAP) and growth‐hormone‐releasing hormone (GHRH)) [http://www.ncbi.nlm.nih.gov/pubmed/11790261?dopt=AbstractPlus].

### GPCR families

**Table:** 

**Family**	**Class A**	**Class B (Secretin)**	**Class C (Glutamate)**	**Adhesion**	**Frizzled**
**Receptors with known ligands**	197	15	12	0	11
**Orphans**	87 (54)^a^	‐	8 (1)^a^	26 (6)^a^	0
**Sensory (olfaction)**	390^b,c^	‐	‐	‐	‐
**Sensory (vision)**	10^d^ opsins	‐	‐	‐	‐
**Sensory (taste)**	30^c^ taste 2	‐	3^c^ taste 1	‐	‐
**Sensory (pheromone)**	5^c^ vomeronasal 1	‐	‐	‐	‐
**Total**	719	15	22	33	11

^a^Numbers in brackets refer to orphan receptors for which an endogenous ligand has been proposed in at least one publication, see [http://www.ncbi.nlm.nih.gov/pubmed/23686350?dopt=AbstractPlus]; ^b^[http://www.ncbi.nlm.nih.gov/pubmed/19129093?dopt=AbstractPlus]; ^c^[http://www.ncbi.nlm.nih.gov/pubmed/15034552?dopt=AbstractPlus]; ^d^[http://www.ncbi.nlm.nih.gov/pubmed/15774036?dopt=AbstractPlus].

Much of our current understanding of the structure and function of GPCRs is the result of pioneering work on the visual pigment rhodopsin and on the β_2_ adrenoceptor, the latter culminating in the award of the 2012 Nobel Prize in chemistry to Robert Lefkowitz and Brian Kobilka [http://www.ncbi.nlm.nih.gov/pubmed/23650120?dopt=AbstractPlus, http://www.ncbi.nlm.nih.gov/pubmed/23650015?dopt=AbstractPlus].

### Pseudogenes

Below is a curated list of pseudogenes that in humans are non‐coding for receptor protein. In some cases these have a shared ancestry with genes that encode functional receptors in rats and mice.

https://www.genenames.org/data/gene‐symbol‐report/#!/hgnc_id/HGNC:19240, https://www.genenames.org/data/gene‐symbol‐report/#!/hgnc_id/HGNC:16341, https://www.genenames.org/data/gene‐symbol‐report/#!/hgnc_id/HGNC:4529, https://www.genenames.org/data/gene‐symbol‐report/#!/hgnc_id/HGNC:16291, https://www.genenames.org/data/gene‐symbol‐report/#!/hgnc_id/HGNC:7959, https://www.genenames.org/data/gene‐symbol‐report/#!/hgnc_id/HGNC:4513, https://www.genenames.org/data/gene‐symbol‐report/#!/hgnc_id/HGNC:31924, https://www.genenames.org/data/gene‐symbol‐report/#!/hgnc_id/HGNC:31925, https://www.genenames.org/data/gene‐symbol‐report/#!/hgnc_id/HGNC:19103, https://www.genenames.org/data/gene‐symbol‐report/#!/hgnc_id/HGNC:19106, https://www.genenames.org/data/gene‐symbol‐report/#!/hgnc_id/HGNC:19107, https://www.genenames.org/data/gene‐symbol‐report/#!/hgnc_id/HGNC:20615, https://www.genenames.org/data/gene‐symbol‐report/#!/hgnc_id/HGNC:20640, https://www.genenames.org/data/gene‐symbol‐report/#!/hgnc_id/HGNC:20641, https://www.genenames.org/data/gene‐symbol‐report/#!/hgnc_id/HGNC:20642, https://www.genenames.org/data/gene‐symbol‐report/#!/hgnc_id/HGNC:43898, https://www.genenames.org/data/gene‐symbol‐report/#!/hgnc_id/HGNC:43905, https://www.genenames.org/data/gene‐symbol‐report/#!/hgnc_id/HGNC:20616. A more detailed listing containg further information can be viewed http://www.guidetopharmacology.org/GRAC//DATA/GPCR_pseudogenes.xlsx.

### Olfactory receptors

Olfactory receptors are also seven‐transmembrane spanning G protein‐coupled receptors, responsible for the detection of odorants. These are not currently included as they are not yet associated with extensive pharmacological data but are curated in the following databases: The gene list of olfactory receptors at https://www.genenames.org/cgi‐bin/genefamilies/set/141, and curated by https://genome.weizmann.ac.il/horde/ and https://senselab.med.yale.edu/ordb/.

### Further reading on G protein‐coupled receptors

Kenakin T. (2018) Is the Quest for Signaling Bias Worth the Effort? *Mol. Pharmacol.* **93**: 266‐269 [https://www.ncbi.nlm.nih.gov/pubmed/29348268?dopt=AbstractPlus]

Michel MC *et al*. (2018) Biased Agonism in Drug Discovery‐Is It Too Soon to Choose a Path? *Mol. Pharmacol.* **93**: 259‐265 [https://www.ncbi.nlm.nih.gov/pubmed/29326242?dopt=AbstractPlus]

Roth BL *et al*. (2017) Discovery of new GPCR ligands to illuminate new biology. *Nat. Chem. Biol.* **13**: 1143‐1151 [https://www.ncbi.nlm.nih.gov/pubmed/29045379?dopt=AbstractPlus]

Sriram K *et al*. (2018) G Protein‐Coupled Receptors as Targets for Approved Drugs: How Many Targets and How Many Drugs? *Mol. Pharmacol.* **93**: 251‐258 [https://www.ncbi.nlm.nih.gov/pubmed/29298813?dopt=AbstractPlus]

### Family structure

S23 Orphan and other 7TM receptors

S24 Class A Orphans

– http://www.guidetopharmacology.org/GRAC/FamilyDisplayForward?familyId=706

S33 Class C Orphans

–http://www.guidetopharmacology.org/GRAC/FamilyDisplayForward?familyId=975

S33 Taste 1 receptors

S34 Taste 2 receptors

S35 Other 7TM proteins

S36 5‐Hydroxytryptamine receptors

S39 Acetylcholine receptors (muscarinic)

S41 Adenosine receptors

S42 Adhesion Class GPCRs

S45 Adrenoceptors

S48 Angiotensin receptors

S50 Apelin receptor

S51 Bile acid receptor

S51 Bombesin receptors

S53 Bradykinin receptors

S54 Calcitonin receptors

S56 Calcium‐sensing receptor

S57 Cannabinoid receptors

S58 Chemerin receptors

S59 Chemokine receptors

S63 Cholecystokinin receptors

S64 Class Frizzled GPCRs

S67 Complement peptide receptors

S68 Corticotropin‐releasing factor receptors

S69 Dopamine receptors

S71 Endothelin receptors

S72 G protein‐coupled estrogen receptor

S73 Formylpeptide receptors

S74 Free fatty acid receptors

S76 GABA_B_ receptors

S78 Galanin receptors

S79 Ghrelin receptor

S80 Glucagon receptor family

S81 Glycoprotein hormone receptors

S82 Gonadotrophin‐releasing hormone receptors

S83 GPR18, GPR55 and GPR119

S84 Histamine receptors

S86 Hydroxycarboxylic acid receptors

S87 Kisspeptin receptor

S88 Leukotriene receptors

S89 Lysophospholipid (LPA) receptors

S90 Lysophospholipid (S1P) receptors

S92 Melanin‐concentrating hormone receptors

S93 Melanocortin receptors

S94 Melatonin receptors

S95 Metabotropic glutamate receptors

S97 Motilin receptor

S98 Neuromedin U receptors

S99 Neuropeptide FF/neuropeptide AF receptors

S100 Neuropeptide S receptor

S101 Neuropeptide W/neuropeptide B receptors

S102 Neuropeptide Y receptors

S103 Neurotensin receptors

S104 Opioid receptors

S106 Orexin receptors

S107 Oxoglutarate receptor

S108 P2Y receptors

S110 Parathyroid hormone receptors

S111 Platelet‐activating factor receptor

S112 Prokineticin receptors

S113 Prolactin‐releasing peptide receptor

S114 Prostanoid receptors

S116 Proteinase‐activated receptors

S117 QRFP receptor

S118 Relaxin family peptide receptors

S120 Somatostatin receptors

S121 Succinate receptor

S122 Tachykinin receptors

S123 Thyrotropin‐releasing hormone receptors

S124 Trace amine receptor

S125 Urotensin receptor

S126 Vasopressin and oxytocin receptors

S127 VIP and PACAP receptors

### http://www.guidetopharmacology.org/GRAC/FamilyDisplayForward?familyId=115

#### Overview

This set contains ’orphan’ G protein coupled receptors where the endogenous ligand(s) is not known, and other 7TM receptors.

### http://www.guidetopharmacology.org/GRAC/FamilyDisplayForward?familyId=16

#### Overview

Table 1 lists a number of putative GPCRs identified by **NC‐IUPHAR**[http://www.ncbi.nlm.nih.gov/pubmed/15914470?dopt=AbstractPlus], for which preliminary evidence for an endogenous ligand has been published, or for which there exists a potential link to a disease, or disorder. These GPCRs have recently been reviewed in detail [http://www.ncbi.nlm.nih.gov/pubmed/23686350?dopt=AbstractPlus]. The GPCRs in Table 1 are all Class A, rhodopsin‐like GPCRs. Class A orphan GPCRs not listed in Table 1 are putative GPCRs with as‐yet unidentified endogenous ligands.

#### Table 1

Class A orphan GPCRs with putative endogenous ligands

**Table:** 

http://www.guidetopharmacology.org/GRAC/ObjectDisplayForward?objectId=83	http://www.guidetopharmacology.org/GRAC/ObjectDisplayForward?objectId=84	http://www.guidetopharmacology.org/GRAC/ObjectDisplayForward?objectId=85	http://www.guidetopharmacology.org/GRAC/ObjectDisplayForward?objectId=86	http://www.guidetopharmacology.org/GRAC/ObjectDisplayForward?objectId=87	http://www.guidetopharmacology.org/GRAC/ObjectDisplayForward?objectId=88	http://www.guidetopharmacology.org/GRAC/ObjectDisplayForward?objectId=91
http://www.guidetopharmacology.org/GRAC/ObjectDisplayForward?objectId=93	http://www.guidetopharmacology.org/GRAC/ObjectDisplayForward?objectId=96	http://www.guidetopharmacology.org/GRAC/ObjectDisplayForward?objectId=98	http://www.guidetopharmacology.org/GRAC/ObjectDisplayForward?objectId=101	http://www.guidetopharmacology.org/GRAC/ObjectDisplayForward?objectId=102	http://www.guidetopharmacology.org/GRAC/ObjectDisplayForward?objectId=103	http://www.guidetopharmacology.org/GRAC/ObjectDisplayForward?objectId=105
http://www.guidetopharmacology.org/GRAC/ObjectDisplayForward?objectId=107	http://www.guidetopharmacology.org/GRAC/ObjectDisplayForward?objectId=112	http://www.guidetopharmacology.org/GRAC/ObjectDisplayForward?objectId=113	http://www.guidetopharmacology.org/GRAC/ObjectDisplayForward?objectId=114	http://www.guidetopharmacology.org/GRAC/ObjectDisplayForward?objectId=115	http://www.guidetopharmacology.org/GRAC/ObjectDisplayForward?objectId=120	http://www.guidetopharmacology.org/GRAC/ObjectDisplayForward?objectId=122
http://www.guidetopharmacology.org/GRAC/ObjectDisplayForward?objectId=123	http://www.guidetopharmacology.org/GRAC/ObjectDisplayForward?objectId=128	http://www.guidetopharmacology.org/GRAC/ObjectDisplayForward?objectId=135	http://www.guidetopharmacology.org/GRAC/ObjectDisplayForward?objectId=141	http://www.guidetopharmacology.org/GRAC/ObjectDisplayForward?objectId=81	http://www.guidetopharmacology.org/GRAC/ObjectDisplayForward?objectId=147	http://www.guidetopharmacology.org/GRAC/ObjectDisplayForward?objectId=148
http://www.guidetopharmacology.org/GRAC/ObjectDisplayForward?objectId=149	http://www.guidetopharmacology.org/GRAC/ObjectDisplayForward?objectId=150	http://www.guidetopharmacology.org/GRAC/ObjectDisplayForward?objectId=152	http://www.guidetopharmacology.org/GRAC/ObjectDisplayForward?objectId=156	http://www.guidetopharmacology.org/GRAC/ObjectDisplayForward?objectId=157	http://www.guidetopharmacology.org/GRAC/ObjectDisplayForward?objectId=165	http://www.guidetopharmacology.org/GRAC/ObjectDisplayForward?objectId=167

In addition the orphan receptors http://www.guidetopharmacology.org/GRAC/ObjectDisplayForward?objectId=89, http://www.guidetopharmacology.org/GRAC/ObjectDisplayForward?objectId=109 and http://www.guidetopharmacology.org/GRAC/ObjectDisplayForward?objectId=126 which are reported to respond to endogenous agents analogous to the endogenous cannabinoid ligands have been grouped together (http://www.guidetopharmacology.org/GRAC/FamilyDisplayForward?familyId=114).

**Table:** 

Nomenclature	http://www.guidetopharmacology.org/GRAC/ObjectDisplayForward?objectId=83	http://www.guidetopharmacology.org/GRAC/ObjectDisplayForward?objectId=84	http://www.guidetopharmacology.org/GRAC/ObjectDisplayForward?objectId=85
HGNC, UniProt	https://www.genenames.org/data/gene‐symbol‐report/#!/hgnc_id/HGNC:4484, http://www.uniprot.org/uniprot/P46089	https://www.genenames.org/data/gene‐symbol‐report/#!/hgnc_id/HGNC:4497, http://www.uniprot.org/uniprot/P46093	https://www.genenames.org/data/gene‐symbol‐report/#!/hgnc_id/HGNC:4515, http://www.uniprot.org/uniprot/P46095
Agonists	http://www.guidetopharmacology.org/GRAC/LigandDisplayForward?ligandId=7802 [http://www.ncbi.nlm.nih.gov/pubmed/24633425?dopt=AbstractPlus]	–	–
Endogenous ligands	–	Protons	–
Comments	http://www.guidetopharmacology.org/GRAC/LigandDisplayForward?ligandId=911 was reported to be an endogenous agonist [http://www.ncbi.nlm.nih.gov/pubmed/12220620?dopt=AbstractPlus], but this finding was not replicated in subsequent studies [http://www.ncbi.nlm.nih.gov/pubmed/19286662?dopt=AbstractPlus]. Reported to activate adenylyl cyclase constitutively through G_s_ [http://www.ncbi.nlm.nih.gov/pubmed/7639700?dopt=AbstractPlus]. Gene disruption results in premature ovarian ageing [http://www.ncbi.nlm.nih.gov/pubmed/15956199?dopt=AbstractPlus], reduced β‐amyloid deposition [http://www.ncbi.nlm.nih.gov/pubmed/19213921?dopt=AbstractPlus] and hypersensitivity to thermal pain [http://www.ncbi.nlm.nih.gov/pubmed/21352831?dopt=AbstractPlus] in mice. First small molecule inverse agonist [http://www.ncbi.nlm.nih.gov/pubmed/25442311?dopt=AbstractPlus] and agonists identified [http://www.ncbi.nlm.nih.gov/pubmed/24633425?dopt=AbstractPlus]	An initial report suggesting activation by http://www.guidetopharmacology.org/GRAC/LigandDisplayForward?ligandId=2508 and http://www.guidetopharmacology.org/GRAC/LigandDisplayForward?ligandId=4032 [http://www.ncbi.nlm.nih.gov/pubmed/11535583?dopt=AbstractPlus] has been retracted [http://www.ncbi.nlm.nih.gov/pubmed/16498716?dopt=AbstractPlus]. GPR4, GPR65, GPR68 and GPR132 are now thought to function as proton‐sensing receptors detecting acidic pH [http://www.ncbi.nlm.nih.gov/pubmed/23686350?dopt=AbstractPlus, http://www.ncbi.nlm.nih.gov/pubmed/17118800?dopt=AbstractPlus]. Gene disruption is associated with increased perinatal mortality and impaired vascular proliferation [http://www.ncbi.nlm.nih.gov/pubmed/17145776?dopt=AbstractPlus]. Negative allosteric modulators of GPR4 have been reported [http://www.ncbi.nlm.nih.gov/pubmed/26070068?dopt=AbstractPlus].	An initial report that http://www.guidetopharmacology.org/GRAC/LigandDisplayForward?ligandId=911 (S1P) was a high‐affinity ligand (EC_50_ value of 39nM) [http://www.ncbi.nlm.nih.gov/pubmed/14592418?dopt=AbstractPlus, http://www.ncbi.nlm.nih.gov/pubmed/12220620?dopt=AbstractPlus] was not repeated in arrestin‐based assays [http://www.ncbi.nlm.nih.gov/pubmed/23396314?dopt=AbstractPlus, http://www.ncbi.nlm.nih.gov/pubmed/19286662?dopt=AbstractPlus]. Reported to activate adenylyl cyclase constitutively through G_s_ and to be located intracellularly [http://www.ncbi.nlm.nih.gov/pubmed/19059244?dopt=AbstractPlus]. GPR6‐deficient mice showed reduced striatal cyclic AMP production *in vitro* and selected alterations in instrumental conditioning *in vivo*. [http://www.ncbi.nlm.nih.gov/pubmed/17934457?dopt=AbstractPlus].

**Table:** 

Nomenclature	http://www.guidetopharmacology.org/GRAC/ObjectDisplayForward?objectId=86	http://www.guidetopharmacology.org/GRAC/ObjectDisplayForward?objectId=87	http://www.guidetopharmacology.org/GRAC/ObjectDisplayForward?objectId=88
HGNC, UniProt	https://www.genenames.org/data/gene‐symbol‐report/#!/hgnc_id/HGNC:4466, http://www.uniprot.org/uniprot/P47775	https://www.genenames.org/data/gene‐symbol‐report/#!/hgnc_id/HGNC:4469, http://www.uniprot.org/uniprot/P49685	https://www.genenames.org/data/gene‐symbol‐report/#!/hgnc_id/HGNC:4471, http://www.uniprot.org/uniprot/Q13304
Endogenous agonists	–	–	http://www.guidetopharmacology.org/GRAC/LigandDisplayForward?ligandId=1783 [http://www.ncbi.nlm.nih.gov/pubmed/20148890?dopt=AbstractPlus, http://www.ncbi.nlm.nih.gov/pubmed/16990797?dopt=AbstractPlus], http://www.guidetopharmacology.org/GRAC/LigandDisplayForward?ligandId=3354 [http://www.ncbi.nlm.nih.gov/pubmed/16990797?dopt=AbstractPlus], http://www.guidetopharmacology.org/GRAC/LigandDisplayForward?ligandId=1782 [http://www.ncbi.nlm.nih.gov/pubmed/20148890?dopt=AbstractPlus, http://www.ncbi.nlm.nih.gov/pubmed/16990797?dopt=AbstractPlus], http://www.guidetopharmacology.org/GRAC/LigandDisplayForward?ligandId=1749 [http://www.ncbi.nlm.nih.gov/pubmed/20148890?dopt=AbstractPlus, http://www.ncbi.nlm.nih.gov/pubmed/16990797?dopt=AbstractPlus], http://www.guidetopharmacology.org/GRAC/LigandDisplayForward?ligandId=3353[http://www.ncbi.nlm.nih.gov/pubmed/16990797?dopt=AbstractPlus]
Comments	Reports that http://www.guidetopharmacology.org/GRAC/LigandDisplayForward?ligandId=911 is a ligand of GPR12 [http://www.ncbi.nlm.nih.gov/pubmed/12574419?dopt=AbstractPlus, http://www.ncbi.nlm.nih.gov/pubmed/12220620?dopt=AbstractPlus] have not been replicated in arrestin‐based assays [http://www.ncbi.nlm.nih.gov/pubmed/23396314?dopt=AbstractPlus, http://www.ncbi.nlm.nih.gov/pubmed/19286662?dopt=AbstractPlus]. Gene disruption results in dyslipidemia and obesity [http://www.ncbi.nlm.nih.gov/pubmed/16887097?dopt=AbstractPlus].	Reported to act as a co‐receptor for HIV [http://www.ncbi.nlm.nih.gov/pubmed/9405683?dopt=AbstractPlus]. In an infection‐induced colitis model, *Gpr15* knockout mice were more prone to tissue damage and inflammatory cytokine expression [http://www.ncbi.nlm.nih.gov/pubmed/23661644?dopt=AbstractPlus].	Reported to be a dual leukotriene and http://www.guidetopharmacology.org/GRAC/LigandDisplayForward?ligandId=1749 receptor [http://www.ncbi.nlm.nih.gov/pubmed/16990797?dopt=AbstractPlus]. Another group instead proposed that GPR17 functions as a negative regulator of the CysLT_1_ receptor response to leukotriene D_4_ (http://www.guidetopharmacology.org/GRAC/LigandDisplayForward?ligandId=3353). For further discussion, see [http://www.ncbi.nlm.nih.gov/pubmed/23686350?dopt=AbstractPlus]. Reported to antagonize CysLT_1_ receptor signalling *in vivo* and *in vitro* [http://www.ncbi.nlm.nih.gov/pubmed/19561298?dopt=AbstractPlus]. See reviews [http://www.ncbi.nlm.nih.gov/pubmed/24588652?dopt=AbstractPlus] and [http://www.ncbi.nlm.nih.gov/pubmed/23686350?dopt=AbstractPlus].

**Table:** 

Nomenclature	http://www.guidetopharmacology.org/GRAC/ObjectDisplayForward?objectId=90	http://www.guidetopharmacology.org/GRAC/ObjectDisplayForward?objectId=91	http://www.guidetopharmacology.org/GRAC/ObjectDisplayForward?objectId=92	http://www.guidetopharmacology.org/GRAC/ObjectDisplayForward?objectId=93	http://www.guidetopharmacology.org/GRAC/ObjectDisplayForward?objectId=95	http://www.guidetopharmacology.org/GRAC/ObjectDisplayForward?objectId=96	http://www.guidetopharmacology.org/GRAC/ObjectDisplayForward?objectId=97
HGNC, UniProt	https://www.genenames.org/data/gene‐symbol‐report/#!/hgnc_id/HGNC:4473, http://www.uniprot.org/uniprot/Q15760	https://www.genenames.org/data/gene‐symbol‐report/#!/hgnc_id/HGNC:4475, http://www.uniprot.org/uniprot/Q99678	https://www.genenames.org/data/gene‐symbol‐report/#!/hgnc_id/HGNC:4476, http://www.uniprot.org/uniprot/Q99679	https://www.genenames.org/data/gene‐symbol‐report/#!/hgnc_id/HGNC:4477, http://www.uniprot.org/uniprot/Q99680	https://www.genenames.org/data/gene‐symbol‐report/#!/hgnc_id/HGNC:4480, http://www.uniprot.org/uniprot/O00155	https://www.genenames.org/data/gene‐symbol‐report/#!/hgnc_id/HGNC:4481, http://www.uniprot.org/uniprot/Q8NDV2	https://www.genenames.org/data/gene‐symbol‐report/#!/hgnc_id/HGNC:4482, http://www.uniprot.org/uniprot/Q9NS67
Agonists	http://www.guidetopharmacology.org/GRAC/LigandDisplayForward?ligandId=9573 (https://www.genenames.org/data/gene‐symbol‐report/#!/hgnc_id/HGNC:24838, http://www.uniprot.org/uniprot/Q6UWT2) [http://www.ncbi.nlm.nih.gov/pubmed/28476646?dopt=AbstractPlus]	–	–	–	–	–	–
Comments	–	Reported to inhibit adenylyl cyclase constitutively through G_i/o_ [http://www.ncbi.nlm.nih.gov/pubmed/18347022?dopt=AbstractPlus]. GPR20 deficient mice exhibit hyperactivity characterised by increased total distance travelled in an open field test [230].	*Gpr21* knockout mice were resistant to diet‐induced obesity, exhibiting an increase in glucose tolerance and insulin sensitivity, as well as a modest lean phenotype [http://www.ncbi.nlm.nih.gov/pubmed/22653059?dopt=AbstractPlus].	Gene disruption results in increased severity of functional decompensation following aortic banding [http://www.ncbi.nlm.nih.gov/pubmed/18539757?dopt=AbstractPlus]. Identified as a susceptibility locus for osteoarthritis [http://www.ncbi.nlm.nih.gov/pubmed/21068099?dopt=AbstractPlus, http://www.ncbi.nlm.nih.gov/pubmed/20112360?dopt=AbstractPlus, http://www.ncbi.nlm.nih.gov/pubmed/20090528?dopt=AbstractPlus].	–	Has been reported to activate adenylyl cyclase constitutively through G_s_ [http://www.ncbi.nlm.nih.gov/pubmed/17363172?dopt=AbstractPlus]. *Gpr26* knockout mice show increased levels of anxiety and depression‐like behaviours [http://www.ncbi.nlm.nih.gov/pubmed/21924326?dopt=AbstractPlus].	Knockdown of Gpr27 reduces endogenous mouse insulin promotor activity and glucose‐stimulated insulin secretion [http://www.ncbi.nlm.nih.gov/pubmed/22253604?dopt=AbstractPlus].

**Table:** 

Nomenclature	http://www.guidetopharmacology.org/GRAC/ObjectDisplayForward?objectId=98	http://www.guidetopharmacology.org/GRAC/ObjectDisplayForward?objectId=99	http://www.guidetopharmacology.org/GRAC/ObjectDisplayForward?objectId=100	http://www.guidetopharmacology.org/GRAC/ObjectDisplayForward?objectId=101
HGNC, UniProt	https://www.genenames.org/data/gene‐symbol‐report/#!/hgnc_id/HGNC:4486, http://www.uniprot.org/uniprot/O00270	https://www.genenames.org/data/gene‐symbol‐report/#!/hgnc_id/HGNC:4487, http://www.uniprot.org/uniprot/O75388	https://www.genenames.org/data/gene‐symbol‐report/#!/hgnc_id/HGNC:4489, http://www.uniprot.org/uniprot/Q49SQ1	https://www.genenames.org/data/gene‐symbol‐report/#!/hgnc_id/HGNC:4490, http://www.uniprot.org/uniprot/Q9UPC5
Potency order of endogenous ligands	–	http://www.guidetopharmacology.org/GRAC/LigandDisplayForward?ligandId=3934 > http://www.guidetopharmacology.org/GRAC/LigandDisplayForward?ligandId=1034	–	–
Endogenous agonists	http://www.guidetopharmacology.org/GRAC/LigandDisplayForward?ligandId=3404 [http://www.ncbi.nlm.nih.gov/pubmed/21712392?dopt=AbstractPlus] – Mouse	http://www.guidetopharmacology.org/GRAC/LigandDisplayForward?ligandId=3934 [http://www.ncbi.nlm.nih.gov/pubmed/20080636?dopt=AbstractPlus], http://www.guidetopharmacology.org/GRAC/LigandDisplayForward?ligandId=1034 [http://www.ncbi.nlm.nih.gov/pubmed/20080636?dopt=AbstractPlus]	–	http://www.guidetopharmacology.org/GRAC/LigandDisplayForward?ligandId=4064 [http://www.ncbi.nlm.nih.gov/pubmed/22343749?dopt=AbstractPlus, http://www.ncbi.nlm.nih.gov/pubmed/16460680?dopt=AbstractPlus]
Labelled ligands	–	[http://www.guidetopharmacology.org/GRAC/LigandDisplayForward?ligandId=4356 (Agonist) [http://www.ncbi.nlm.nih.gov/pubmed/20080636?dopt=AbstractPlus]	–	–
Comments	See [http://www.ncbi.nlm.nih.gov/pubmed/23686350?dopt=AbstractPlus] for discussion of pairing.	http://www.guidetopharmacology.org/GRAC/LigandDisplayForward?ligandId=3934 has been demonstrated to activate GPR32 in two publications [http://www.ncbi.nlm.nih.gov/pubmed/22538616?dopt=AbstractPlus, http://www.ncbi.nlm.nih.gov/pubmed/20080636?dopt=AbstractPlus]. The pairing was not replicated in a recent study based on arrestin recruitment [http://www.ncbi.nlm.nih.gov/pubmed/23396314?dopt=AbstractPlus]. *GPR32* is a pseudogene in mice and rats. See reviews [http://www.ncbi.nlm.nih.gov/pubmed/24588652?dopt=AbstractPlus] and [http://www.ncbi.nlm.nih.gov/pubmed/23686350?dopt=AbstractPlus].	*GPR33* is a pseudogene in most individuals, containing a premature stop codon within the coding sequence of the second intracellular loop [http://www.ncbi.nlm.nih.gov/pubmed/15987686?dopt=AbstractPlus].	Lysophosphatidylserine has been reported to be a ligand of GPR34 in several publications, but the pairing was not replicated in a recent study based on arrestin recruitment [http://www.ncbi.nlm.nih.gov/pubmed/23396314?dopt=AbstractPlus]. Fails to respond to a variety of lipid‐derived agents [http://www.ncbi.nlm.nih.gov/pubmed/19286662?dopt=AbstractPlus]. Gene disruption results in an enhanced immune response [http://www.ncbi.nlm.nih.gov/pubmed/21097509?dopt=AbstractPlus]. Characterization of agonists at this receptor is discussed in [http://www.ncbi.nlm.nih.gov/pubmed/25970039?dopt=AbstractPlus] and [http://www.ncbi.nlm.nih.gov/pubmed/23686350?dopt=AbstractPlus].

**Table:** 

Nomenclature	http://www.guidetopharmacology.org/GRAC/ObjectDisplayForward?objectId=102	http://www.guidetopharmacology.org/GRAC/ObjectDisplayForward?objectId=103
HGNC, UniProt	https://www.genenames.org/data/gene‐symbol‐report/#!/hgnc_id/HGNC:4492, http://www.uniprot.org/uniprot/Q9HC97	https://www.genenames.org/data/gene‐symbol‐report/#!/hgnc_id/HGNC:4494, http://www.uniprot.org/uniprot/O15354
Endogenous agonists	http://www.guidetopharmacology.org/GRAC/LigandDisplayForward?ligandId=2936 [http://www.ncbi.nlm.nih.gov/pubmed/20361937?dopt=AbstractPlus], http://www.guidetopharmacology.org/GRAC/LigandDisplayForward?ligandId=2918 [http://www.ncbi.nlm.nih.gov/pubmed/23396314?dopt=AbstractPlus, http://www.ncbi.nlm.nih.gov/pubmed/16754668?dopt=AbstractPlus]	–
Agonists	–	http://www.guidetopharmacology.org/GRAC/LigandDisplayForward?ligandId=2496 [http://www.ncbi.nlm.nih.gov/pubmed/16443751?dopt=AbstractPlus]
Comments	Several studies have shown that kynurenic acid is an agonist of GPR35 but it remains controversial whether the proposed endogenous ligand reaches sufficient tissue concentrations to activate the receptor [http://www.ncbi.nlm.nih.gov/pubmed/18235993?dopt=AbstractPlus]. http://www.guidetopharmacology.org/GRAC/LigandDisplayForward?ligandId=2936 has also been proposed as an endogenous ligand [http://www.ncbi.nlm.nih.gov/pubmed/20361937?dopt=AbstractPlus] but these results were not replicated in an arrestin assay [http://www.ncbi.nlm.nih.gov/pubmed/23396314?dopt=AbstractPlus]. The phosphodiesterase inhibitor http://www.guidetopharmacology.org/GRAC/LigandDisplayForward?ligandId=2919 [http://www.ncbi.nlm.nih.gov/pubmed/16934253?dopt=AbstractPlus] has become widely used as a surrogate agonist to investigate GPR35 pharmacology and signalling [http://www.ncbi.nlm.nih.gov/pubmed/16934253?dopt=AbstractPlus]. GPR35 is also activated by the pharmaceutical adjunct http://www.guidetopharmacology.org/GRAC/LigandDisplayForward?ligandId=2920 [http://www.ncbi.nlm.nih.gov/pubmed/20826425?dopt=AbstractPlus]. See reviews [http://www.ncbi.nlm.nih.gov/pubmed/23686350?dopt=AbstractPlus] and [http://www.ncbi.nlm.nih.gov/pubmed/25805994?dopt=AbstractPlus].	Reported to associate and regulate the dopamine transporter [http://www.ncbi.nlm.nih.gov/pubmed/17519329?dopt=AbstractPlus] and to be a substrate for parkin [http://www.ncbi.nlm.nih.gov/pubmed/19398891?dopt=AbstractPlus]. Gene disruption results in altered striatal signalling [http://www.ncbi.nlm.nih.gov/pubmed/21372109?dopt=AbstractPlus]. The peptides prosaptide and prosaposin are proposed as endogenous ligands for GPR37 and GPR37L1 [http://www.ncbi.nlm.nih.gov/pubmed/23690594?dopt=AbstractPlus].

**Table:** 

Nomenclature	http://www.guidetopharmacology.org/GRAC/ObjectDisplayForward?objectId=104	http://www.guidetopharmacology.org/GRAC/ObjectDisplayForward?objectId=105	http://www.guidetopharmacology.org/GRAC/ObjectDisplayForward?objectId=106	http://www.guidetopharmacology.org/GRAC/ObjectDisplayForward?objectId=107
HGNC, UniProt	https://www.genenames.org/data/gene‐symbol‐report/#!/hgnc_id/HGNC:14923, http://www.uniprot.org/uniprot/O60883	https://www.genenames.org/data/gene‐symbol‐report/#!/hgnc_id/HGNC:4496, http://www.uniprot.org/uniprot/O43194	https://www.genenames.org/data/gene‐symbol‐report/#!/hgnc_id/HGNC:4503, http://www.uniprot.org/uniprot/Q9Y5Y3	https://www.genenames.org/data/gene‐symbol‐report/#!/hgnc_id/HGNC:4506, http://www.uniprot.org/uniprot/Q13585
Endogenous agonists	–	http://www.guidetopharmacology.org/GRAC/LigandDisplayForward?ligandId=566 [http://www.ncbi.nlm.nih.gov/pubmed/16959833?dopt=AbstractPlus]	–	–
Comments	The peptides prosaptide and prosaposin are proposed as endogenous ligands for GPR37 and GPR37L1 [http://www.ncbi.nlm.nih.gov/pubmed/23690594?dopt=AbstractPlus].	Zn^2+^ has been reported to be a potent and efficacious agonist of human, mouse and rat GPR39 [http://www.ncbi.nlm.nih.gov/pubmed/17214962?dopt=AbstractPlus]. http://www.guidetopharmacology.org/GRAC/LigandDisplayForward?ligandId=5336 (https://www.genenames.org/data/gene‐symbol‐report/#!/hgnc_id/HGNC:18129, http://www.uniprot.org/uniprot/Q9UBU3), a fragment from the ghrelin precursor, was reported initially as an endogenous ligand, but subsequent studies failed to reproduce these findings. *GPR39* has been reported to be down‐regulated in adipose tissue in obesity‐related diabetes [http://www.ncbi.nlm.nih.gov/pubmed/17371481?dopt=AbstractPlus]. Gene disruption results in obesity and altered adipocyte metabolism [http://www.ncbi.nlm.nih.gov/pubmed/21784784?dopt=AbstractPlus]. Reviewed in [http://www.ncbi.nlm.nih.gov/pubmed/23686350?dopt=AbstractPlus].	–	GPR50 is structurally related to MT_1_ and MT_2_ melatonin receptors, with which it heterodimerises constitutively and specifically [http://www.ncbi.nlm.nih.gov/pubmed/16778767?dopt=AbstractPlus]. *Gpr50* knockout mice display abnormal thermoregulation and are much more likely than wild‐type mice to enter fasting‐induced torpor [http://www.ncbi.nlm.nih.gov/pubmed/22197240?dopt=AbstractPlus].

**Table:** 

Nomenclature	http://www.guidetopharmacology.org/GRAC/ObjectDisplayForward?objectId=108	http://www.guidetopharmacology.org/GRAC/ObjectDisplayForward?objectId=110	http://www.guidetopharmacology.org/GRAC/ObjectDisplayForward?objectId=111	http://www.guidetopharmacology.org/GRAC/ObjectDisplayForward?objectId=112
HGNC, UniProt	https://www.genenames.org/data/gene‐symbol‐report/#!/hgnc_id/HGNC:4508, http://www.uniprot.org/uniprot/Q9Y2T5	https://www.genenames.org/data/gene‐symbol‐report/#!/hgnc_id/HGNC:13300, http://www.uniprot.org/uniprot/Q9BZJ8	https://www.genenames.org/data/gene‐symbol‐report/#!/hgnc_id/HGNC:13301, http://www.uniprot.org/uniprot/Q9BZJ7	https://www.genenames.org/data/gene‐symbol‐report/#!/hgnc_id/HGNC:13302, http://www.uniprot.org/uniprot/Q9BZJ6
Comments	First small molecule agonist reported [http://www.ncbi.nlm.nih.gov/pubmed/24884590?dopt=AbstractPlus].	GPR61 deficient mice exhibit obesity associated with hyperphagia [http://www.ncbi.nlm.nih.gov/pubmed/21971119?dopt=AbstractPlus]. Although no endogenous ligands have been identified, 5‐(nonyloxy)tryptamine has been reported to be a low affinity inverse agonist [http://www.ncbi.nlm.nih.gov/pubmed/15173198?dopt=AbstractPlus].	–	http://www.guidetopharmacology.org/GRAC/LigandDisplayForward?ligandId=911 and http://www.guidetopharmacology.org/GRAC/LigandDisplayForward?ligandId=5512 have been reported to be low affinity agonists for GPR63 [http://www.ncbi.nlm.nih.gov/pubmed/12618218?dopt=AbstractPlus] but this finding was not replicated in an arrestin‐based assay [http://www.ncbi.nlm.nih.gov/pubmed/19286662?dopt=AbstractPlus].

**Table:** 

Nomenclature	http://www.guidetopharmacology.org/GRAC/ObjectDisplayForward?objectId=113	http://www.guidetopharmacology.org/GRAC/ObjectDisplayForward?objectId=114
HGNC, UniProt	https://www.genenames.org/data/gene‐symbol‐report/#!/hgnc_id/HGNC:4517, http://www.uniprot.org/uniprot/Q8IYL9	https://www.genenames.org/data/gene‐symbol‐report/#!/hgnc_id/HGNC:4519, http://www.uniprot.org/uniprot/Q15743
Endogenous ligands	Protons	Protons
Allosteric modulators	–	http://www.guidetopharmacology.org/GRAC/LigandDisplayForward?ligandId=9155 (Positive) (p*K*_B_ 5) [http://www.ncbi.nlm.nih.gov/pubmed/26550826?dopt=AbstractPlus], http://www.guidetopharmacology.org/GRAC/LigandDisplayForward?ligandId=5884 (Positive) [http://www.ncbi.nlm.nih.gov/pubmed/26550826?dopt=AbstractPlus]
Comments	GPR4, GPR65, GPR68 and GPR132 are now thought to function as proton‐sensing receptors detecting acidic pH [http://www.ncbi.nlm.nih.gov/pubmed/23686350?dopt=AbstractPlus, http://www.ncbi.nlm.nih.gov/pubmed/17118800?dopt=AbstractPlus]. Reported to activate adenylyl cyclase; gene disruption leads to reduced eosinophilia in models of allergic airway disease [http://www.ncbi.nlm.nih.gov/pubmed/19641187?dopt=AbstractPlus].	GPR68 was previously identified as a receptor for http://www.guidetopharmacology.org/GRAC/LigandDisplayForward?ligandId=4032 (SPC) [http://www.ncbi.nlm.nih.gov/pubmed/10806476?dopt=AbstractPlus], but the original publication has been retracted [http://www.ncbi.nlm.nih.gov/pubmed/16508674?dopt=AbstractPlus]. GPR4, GPR65, GPR68 and GPR132 are now thought to function as proton‐sensing receptors detecting acidic pH [http://www.ncbi.nlm.nih.gov/pubmed/23686350?dopt=AbstractPlus, http://www.ncbi.nlm.nih.gov/pubmed/17118800?dopt=AbstractPlus]. A family of 3,5‐disubstituted isoxazoles were identified as agonists of GPR68 [http://www.ncbi.nlm.nih.gov/pubmed/22462679?dopt=AbstractPlus].

**Table:** 

Nomenclature	http://www.guidetopharmacology.org/GRAC/ObjectDisplayForward?objectId=115	http://www.guidetopharmacology.org/GRAC/ObjectDisplayForward?objectId=116	http://www.guidetopharmacology.org/GRAC/ObjectDisplayForward?objectId=117	http://www.guidetopharmacology.org/GRAC/ObjectDisplayForward?objectId=118
HGNC, UniProt	https://www.genenames.org/data/gene‐symbol‐report/#!/hgnc_id/HGNC:4526, http://www.uniprot.org/uniprot/O95800	https://www.genenames.org/data/gene‐symbol‐report/#!/hgnc_id/HGNC:4528, http://www.uniprot.org/uniprot/Q96P69	https://www.genenames.org/data/gene‐symbol‐report/#!/hgnc_id/HGNC:4529, –	https://www.genenames.org/data/gene‐symbol‐report/#!/hgnc_id/HGNC:4533, http://www.uniprot.org/uniprot/Q96P67
Comments	http://www.guidetopharmacology.org/GRAC/LigandDisplayForward?ligandId=758(https://www.genenames.org/data/gene‐symbol‐report/#!/hgnc_id/HGNC:10632, http://www.uniprot.org/uniprot/P13501) was reported to be an agonist of GPR75 [http://www.ncbi.nlm.nih.gov/pubmed/17001303?dopt=AbstractPlus], but the pairing could not be repeated in an arrestin assay [http://www.ncbi.nlm.nih.gov/pubmed/23396314?dopt=AbstractPlus].	GPR78 has been reported to be constitutively active, coupled to elevated cAMP production [http://www.ncbi.nlm.nih.gov/pubmed/17363172?dopt=AbstractPlus].	–	Mice with *Gpr82* knockout have a lower body weight and body fat content associated with reduced food intake, decreased serum triglyceride levels, as well as higher insulin sensitivity and glucose tolerance [http://www.ncbi.nlm.nih.gov/pubmed/22216272?dopt=AbstractPlus].

**Table:** 

Nomenclature	http://www.guidetopharmacology.org/GRAC/ObjectDisplayForward?objectId=119	http://www.guidetopharmacology.org/GRAC/ObjectDisplayForward?objectId=120	http://www.guidetopharmacology.org/GRAC/ObjectDisplayForward?objectId=121	http://www.guidetopharmacology.org/GRAC/ObjectDisplayForward?objectId=122	http://www.guidetopharmacology.org/GRAC/ObjectDisplayForward?objectId=123	http://www.guidetopharmacology.org/GRAC/ObjectDisplayForward?objectId=125
HGNC, UniProt	https://www.genenames.org/data/gene‐symbol‐report/#!/hgnc_id/HGNC:4523, http://www.uniprot.org/uniprot/Q9NYM4	https://www.genenames.org/data/gene‐symbol‐report/#!/hgnc_id/HGNC:4535, http://www.uniprot.org/uniprot/Q9NQS5	https://www.genenames.org/data/gene‐symbol‐report/#!/hgnc_id/HGNC:4536, http://www.uniprot.org/uniprot/P60893	https://www.genenames.org/data/gene‐symbol‐report/#!/hgnc_id/HGNC:4538, http://www.uniprot.org/uniprot/Q9BY21	https://www.genenames.org/data/gene‐symbol‐report/#!/hgnc_id/HGNC:4539, http://www.uniprot.org/uniprot/Q9GZN0	https://www.genenames.org/data/gene‐symbol‐report/#!/hgnc_id/HGNC:14963, http://www.uniprot.org/uniprot/Q96P66
Endogenous agonists	–	–	–	http://www.guidetopharmacology.org/GRAC/LigandDisplayForward?ligandId=2906 [http://www.ncbi.nlm.nih.gov/pubmed/18466763?dopt=AbstractPlus, http://www.ncbi.nlm.nih.gov/pubmed/17905198?dopt=AbstractPlus]	–	–
Agonists	http://www.guidetopharmacology.org/GRAC/LigandDisplayForward?ligandId=9139 {Mouse} [http://www.ncbi.nlm.nih.gov/pubmed/27117253?dopt=AbstractPlus] – Mouse, http://www.guidetopharmacology.org/GRAC/LigandDisplayForward?ligandId=566 [http://www.ncbi.nlm.nih.gov/pubmed/23335960?dopt=AbstractPlus] – Mouse	http://www.guidetopharmacology.org/GRAC/LigandDisplayForward?ligandId=5532 [http://www.ncbi.nlm.nih.gov/pubmed/23396314?dopt=AbstractPlus, http://www.ncbi.nlm.nih.gov/pubmed/16966319?dopt=AbstractPlus], http://www.guidetopharmacology.org/GRAC/LigandDisplayForward?ligandId=5533[http://www.ncbi.nlm.nih.gov/pubmed/16966319?dopt=AbstractPlus], http://www.guidetopharmacology.org/GRAC/LigandDisplayForward?ligandId=5534[http://www.ncbi.nlm.nih.gov/pubmed/16966319?dopt=AbstractPlus]	–	–	–	–
Comments	One isoform has been implicated in the induction of CD4(+) CD25(+) regulatory T cells (Tregs) during inflammatory immune responses [http://www.ncbi.nlm.nih.gov/pubmed/20200545?dopt=AbstractPlus]. The extracellular N‐terminal domain is reported as an intramolecular inverse agonist [http://www.ncbi.nlm.nih.gov/pubmed/25516095?dopt=AbstractPlus].	Medium chain free fatty acids with carbon chain lengths of 9‐14 activate GPR84 [http://www.ncbi.nlm.nih.gov/pubmed/23449982?dopt=AbstractPlus, http://www.ncbi.nlm.nih.gov/pubmed/16966319?dopt=AbstractPlus]. A surrogate ligand for GPR84, http://www.guidetopharmacology.org/GRAC/LigandDisplayForward?ligandId=5846 has also been proposed [http://www.ncbi.nlm.nih.gov/pubmed/23449982?dopt=AbstractPlus]. See review [http://www.ncbi.nlm.nih.gov/pubmed/23686350?dopt=AbstractPlus] for discussion of classification. Mutational analysis and molecular modelling of GPR84 has been reported [http://www.ncbi.nlm.nih.gov/pubmed/25425658?dopt=AbstractPlus].	Proposed to regulate hippocampal neurogenesis in the adult, as well as neurogenesis‐dependent learning and memory [http://www.ncbi.nlm.nih.gov/pubmed/22697179?dopt=AbstractPlus].	–	Gene disruption results in altered striatal signalling [http://www.ncbi.nlm.nih.gov/pubmed/19796684?dopt=AbstractPlus]. Small molecule agonists have been reported [http://www.ncbi.nlm.nih.gov/pubmed/25754495?dopt=AbstractPlus].	Mutations in GPR101 have been linked to gigantism and acromegaly [http://www.ncbi.nlm.nih.gov/pubmed/25470569?dopt=AbstractPlus].

**Table:** 

Nomenclature	http://www.guidetopharmacology.org/GRAC/ObjectDisplayForward?objectId=128	http://www.guidetopharmacology.org/GRAC/ObjectDisplayForward?objectId=129	http://www.guidetopharmacology.org/GRAC/ObjectDisplayForward?objectId=130	http://www.guidetopharmacology.org/GRAC/ObjectDisplayForward?objectId=131	http://www.guidetopharmacology.org/GRAC/ObjectDisplayForward?objectId=132	http://www.guidetopharmacology.org/GRAC/ObjectDisplayForward?objectId=133
HGNC, UniProt	https://www.genenames.org/data/gene‐symbol‐report/#!/hgnc_id/HGNC:17482, http://www.uniprot.org/uniprot/Q9UNW8	https://www.genenames.org/data/gene‐symbol‐report/#!/hgnc_id/HGNC:19991, http://www.uniprot.org/uniprot/Q8IZ08	https://www.genenames.org/data/gene‐symbol‐report/#!/hgnc_id/HGNC:19995, http://www.uniprot.org/uniprot/Q6DWJ6	https://www.genenames.org/data/gene‐symbol‐report/#!/hgnc_id/HGNC:19997, http://www.uniprot.org/uniprot/Q7Z602	https://www.genenames.org/data/gene‐symbol‐report/#!/hgnc_id/HGNC:20088, http://www.uniprot.org/uniprot/Q7Z601	https://www.genenames.org/data/gene‐symbol‐report/#!/hgnc_id/HGNC:21718, http://www.uniprot.org/uniprot/Q96CH1
Endogenous ligands	Protons	–	–	–	–	–
Comments	GPR4, GPR65, GPR68 and GPR132 are now thought to function as proton‐sensing receptors detecting acidic pH [http://www.ncbi.nlm.nih.gov/pubmed/23686350?dopt=AbstractPlus, http://www.ncbi.nlm.nih.gov/pubmed/17118800?dopt=AbstractPlus]. Reported to respond to http://www.guidetopharmacology.org/GRAC/LigandDisplayForward?ligandId=2508 [http://www.ncbi.nlm.nih.gov/pubmed/11474113?dopt=AbstractPlus], but later retracted [http://www.ncbi.nlm.nih.gov/pubmed/15653487?dopt=AbstractPlus].	–	Peptide agonists have been reported [http://www.ncbi.nlm.nih.gov/pubmed/24826842?dopt=AbstractPlus].	–	Small molecule agonists have been reported [http://www.ncbi.nlm.nih.gov/pubmed/24900747?dopt=AbstractPlus, http://www.ncbi.nlm.nih.gov/pubmed/24900757?dopt=AbstractPlus].	Yosten *et al*. demonstrated inhibition of http://www.guidetopharmacology.org/GRAC/LigandDisplayForward?ligandId=6145 (https://www.genenames.org/data/gene‐symbol‐report/#!/hgnc_id/HGNC:6081, http://www.uniprot.org/uniprot/P01308)‐induced stimulation of cFos expression folllowing knockdown of GPR146 in KATO III cells, suggesting proinsulin C‐peptide as an endogenous ligand of the receptor [http://www.ncbi.nlm.nih.gov/pubmed/23759446?dopt=AbstractPlus].

**Table:** 

Nomenclature	http://www.guidetopharmacology.org/GRAC/ObjectDisplayForward?objectId=134	http://www.guidetopharmacology.org/GRAC/ObjectDisplayForward?objectId=135	http://www.guidetopharmacology.org/GRAC/ObjectDisplayForward?objectId=136	http://www.guidetopharmacology.org/GRAC/ObjectDisplayForward?objectId=137	http://www.guidetopharmacology.org/GRAC/ObjectDisplayForward?objectId=138	http://www.guidetopharmacology.org/GRAC/ObjectDisplayForward?objectId=139	http://www.guidetopharmacology.org/GRAC/ObjectDisplayForward?objectId=140
HGNC, UniProt	https://www.genenames.org/data/gene‐symbol‐report/#!/hgnc_id/HGNC:23623, http://www.uniprot.org/uniprot/Q8TDV2	https://www.genenames.org/data/gene‐symbol‐report/#!/hgnc_id/HGNC:23627, http://www.uniprot.org/uniprot/Q86SP6	https://www.genenames.org/data/gene‐symbol‐report/#!/hgnc_id/HGNC:23628, http://www.uniprot.org/uniprot/Q8NGU9	https://www.genenames.org/data/gene‐symbol‐report/#!/hgnc_id/HGNC:23624, http://www.uniprot.org/uniprot/Q8TDV0	https://www.genenames.org/data/gene‐symbol‐report/#!/hgnc_id/HGNC:23622, http://www.uniprot.org/uniprot/Q8TDT2	https://www.genenames.org/data/gene‐symbol‐report/#!/hgnc_id/HGNC:23618, http://www.uniprot.org/uniprot/Q6NV75	https://www.genenames.org/data/gene‐symbol‐report/#!/hgnc_id/HGNC:23693, http://www.uniprot.org/uniprot/Q9UJ42
Comments	–	*Gpr149* knockout mice displayed increased fertility and enhanced ovulation, with increased levels of FSH receptor and cyclin D2 mRNA levels [http://www.ncbi.nlm.nih.gov/pubmed/19887567?dopt=AbstractPlus].	–	GPR151 responded to galanin with an EC_50_ value of 2 *μ*M, suggesting that the endogenous ligand shares structural features with http://www.guidetopharmacology.org/GRAC/LigandDisplayForward?ligandId=3592 (https://www.genenames.org/data/gene‐symbol‐report/#!/hgnc_id/HGNC:4114, http://www.uniprot.org/uniprot/P22466) [http://www.ncbi.nlm.nih.gov/pubmed/15111018?dopt=AbstractPlus].	–	–	–

**Table:** 

Nomenclature	http://www.guidetopharmacology.org/GRAC/ObjectDisplayForward?objectId=141	http://www.guidetopharmacology.org/GRAC/ObjectDisplayForward?objectId=142	http://www.guidetopharmacology.org/GRAC/ObjectDisplayForward?objectId=143	http://www.guidetopharmacology.org/GRAC/ObjectDisplayForward?objectId=144
HGNC, UniProt	https://www.genenames.org/data/gene‐symbol‐report/#!/hgnc_id/HGNC:23694, http://www.uniprot.org/uniprot/Q8N6U8	https://www.genenames.org/data/gene‐symbol‐report/#!/hgnc_id/HGNC:16693, http://www.uniprot.org/uniprot/Q16538	https://www.genenames.org/data/gene‐symbol‐report/#!/hgnc_id/HGNC:30057, http://www.uniprot.org/uniprot/O14626	https://www.genenames.org/data/gene‐symbol‐report/#!/hgnc_id/HGNC:18186, http://www.uniprot.org/uniprot/Q9NS66
Comments	A C‐terminal truncation (deletion) mutation in Gpr161 causes congenital cataracts and neural tube defects in the vacuolated lens (vl) mouse mutant [http://www.ncbi.nlm.nih.gov/pubmed/18250320?dopt=AbstractPlus]. The mutated receptor is associated with cataract, spina bifida and white belly spot phenotypes in mice [http://www.ncbi.nlm.nih.gov/pubmed/18796533?dopt=AbstractPlus]. Gene disruption is associated with a failure of asymmetric embryonic development in zebrafish [http://www.ncbi.nlm.nih.gov/pubmed/18755178?dopt=AbstractPlus].	–	GPR171 has been shown to be activated by the endogenous peptide http://www.guidetopharmacology.org/GRAC/LigandDisplayForward?ligandId=6704 {Mouse}. This receptor‐peptide interaction is believed to be involved in regulating feeding and metabolism responses [http://www.ncbi.nlm.nih.gov/pubmed/24043826?dopt=AbstractPlus].	–

**Table:** 

Nomenclature	http://www.guidetopharmacology.org/GRAC/ObjectDisplayForward?objectId=145	http://www.guidetopharmacology.org/GRAC/ObjectDisplayForward?objectId=637	http://www.guidetopharmacology.org/GRAC/ObjectDisplayForward?objectId=146	http://www.guidetopharmacology.org/GRAC/ObjectDisplayForward?objectId=81
HGNC, UniProt	https://www.genenames.org/data/gene‐symbol‐report/#!/hgnc_id/HGNC:30245, http://www.uniprot.org/uniprot/Q9BXC1	https://www.genenames.org/data/gene‐symbol‐report/#!/hgnc_id/HGNC:32370, http://www.uniprot.org/uniprot/Q14439	https://www.genenames.org/data/gene‐symbol‐report/#!/hgnc_id/HGNC:13708, http://www.uniprot.org/uniprot/O15218	https://www.genenames.org/data/gene‐symbol‐report/#!/hgnc_id/HGNC:3128, http://www.uniprot.org/uniprot/P32249
Endogenous agonists	http://www.guidetopharmacology.org/GRAC/LigandDisplayForward?ligandId=4064 [http://www.ncbi.nlm.nih.gov/pubmed/22983457?dopt=AbstractPlus]	–	–	http://www.guidetopharmacology.org/GRAC/LigandDisplayForward?ligandId=4350 [http://www.ncbi.nlm.nih.gov/pubmed/21796212?dopt=AbstractPlus, http://www.ncbi.nlm.nih.gov/pubmed/21796211?dopt=AbstractPlus], http://www.guidetopharmacology.org/GRAC/LigandDisplayForward?ligandId=4353[http://www.ncbi.nlm.nih.gov/pubmed/21796211?dopt=AbstractPlus], http://www.guidetopharmacology.org/GRAC/LigandDisplayForward?ligandId=4355[http://www.ncbi.nlm.nih.gov/pubmed/21796211?dopt=AbstractPlus], http://www.guidetopharmacology.org/GRAC/LigandDisplayForward?ligandId=4354[http://www.ncbi.nlm.nih.gov/pubmed/21796211?dopt=AbstractPlus]
Comments	See [http://www.ncbi.nlm.nih.gov/pubmed/25970039?dopt=AbstractPlus] which discusses characterization of agonists at this receptor.	–	Rat GPR182 was first proposed as the adrenomedullin receptor [http://www.ncbi.nlm.nih.gov/pubmed/7592696?dopt=AbstractPlus]. However, it was later reported that rat and human GPR182 did not respond to adrenomedullin [http://www.ncbi.nlm.nih.gov/pubmed/9535752?dopt=AbstractPlus] and GPR182 is not currently considered to be a genuine adrenomedullin receptor [http://www.ncbi.nlm.nih.gov/pubmed/21051558?dopt=AbstractPlus].	Two independent publications have shown that http://www.guidetopharmacology.org/GRAC/LigandDisplayForward?ligandId=4350 is an agonist of GPR183 and have demonstrated by mass spectrometry that this oxysterol is present endogenously in tissues [http://www.ncbi.nlm.nih.gov/pubmed/21796212?dopt=AbstractPlus, http://www.ncbi.nlm.nih.gov/pubmed/21796211?dopt=AbstractPlus]. Gpr183‐deficient mice show a reduction in the early antibody response to a T‐dependent antigen. GPR183‐deficient B cells fail to migrate to the outer follicle and instead stay in the follicle centre [http://www.ncbi.nlm.nih.gov/pubmed/21844396?dopt=AbstractPlus, http://www.ncbi.nlm.nih.gov/pubmed/19597478?dopt=AbstractPlus].

**Table:** 

Nomenclature	http://www.guidetopharmacology.org/GRAC/ObjectDisplayForward?objectId=147	http://www.guidetopharmacology.org/GRAC/ObjectDisplayForward?objectId=148	http://www.guidetopharmacology.org/GRAC/ObjectDisplayForward?objectId=149
HGNC, UniProt	https://www.genenames.org/data/gene‐symbol‐report/#!/hgnc_id/HGNC:13299, http://www.uniprot.org/uniprot/Q9BXB1	https://www.genenames.org/data/gene‐symbol‐report/#!/hgnc_id/HGNC:4504, http://www.uniprot.org/uniprot/O75473	https://www.genenames.org/data/gene‐symbol‐report/#!/hgnc_id/HGNC:19719, http://www.uniprot.org/uniprot/Q9HBX8
Endogenous agonists	http://www.guidetopharmacology.org/GRAC/LigandDisplayForward?ligandId=3698 (https://www.genenames.org/data/gene‐symbol‐report/#!/hgnc_id/HGNC:28583, http://www.uniprot.org/uniprot/Q6UXX9) [http://www.ncbi.nlm.nih.gov/pubmed/21693646?dopt=AbstractPlus], http://www.guidetopharmacology.org/GRAC/LigandDisplayForward?ligandId=3697 (https://www.genenames.org/data/gene‐symbol‐report/#!/hgnc_id/HGNC:21679, http://www.uniprot.org/uniprot/Q2MKA7) [http://www.ncbi.nlm.nih.gov/pubmed/21693646?dopt=AbstractPlus], http://www.guidetopharmacology.org/GRAC/LigandDisplayForward?ligandId=3699 (https://www.genenames.org/data/gene‐symbol‐report/#!/hgnc_id/HGNC:20866, http://www.uniprot.org/uniprot/Q9BXY4) [http://www.ncbi.nlm.nih.gov/pubmed/21693646?dopt=AbstractPlus], http://www.guidetopharmacology.org/GRAC/LigandDisplayForward?ligandId=3700 (https://www.genenames.org/data/gene‐symbol‐report/#!/hgnc_id/HGNC:16175, http://www.uniprot.org/uniprot/Q2I0M5) [http://www.ncbi.nlm.nih.gov/pubmed/21693646?dopt=AbstractPlus]	http://www.guidetopharmacology.org/GRAC/LigandDisplayForward?ligandId=3698 (https://www.genenames.org/data/gene‐symbol‐report/#!/hgnc_id/HGNC:28583, http://www.uniprot.org/uniprot/Q6UXX9) [http://www.ncbi.nlm.nih.gov/pubmed/21693646?dopt=AbstractPlus], http://www.guidetopharmacology.org/GRAC/LigandDisplayForward?ligandId=3697 (https://www.genenames.org/data/gene‐symbol‐report/#!/hgnc_id/HGNC:21679, http://www.uniprot.org/uniprot/Q2MKA7) http://www.uniprot.org/uniprot/Q2MKA7], http://www.guidetopharmacology.org/GRAC/LigandDisplayForward?ligandId=3699 (https://www.genenames.org/data/gene‐symbol‐report/#!/hgnc_id/HGNC:20866, http://www.uniprot.org/uniprot/Q9BXY4) [http://www.ncbi.nlm.nih.gov/pubmed/21693646?dopt=AbstractPlus], http://www.guidetopharmacology.org/GRAC/LigandDisplayForward?ligandId=3700 (https://www.genenames.org/data/gene‐symbol‐report/#!/hgnc_id/HGNC:16175, http://www.uniprot.org/uniprot/Q2I0M5) [http://www.ncbi.nlm.nih.gov/pubmed/21693646?dopt=AbstractPlus]	http://www.guidetopharmacology.org/GRAC/LigandDisplayForward?ligandId=3697 (https://www.genenames.org/data/gene‐symbol‐report/#!/hgnc_id/HGNC:21679, http://www.uniprot.org/uniprot/Q2MKA7) [http://www.ncbi.nlm.nih.gov/pubmed/21693646?dopt=AbstractPlus, http://www.ncbi.nlm.nih.gov/pubmed/21727895?dopt=AbstractPlus], http://www.guidetopharmacology.org/GRAC/LigandDisplayForward?ligandId=3698 (https://www.genenames.org/data/gene‐symbol‐report/#!/hgnc_id/HGNC:28583, http://www.uniprot.org/uniprot/Q6UXX9) [http://www.ncbi.nlm.nih.gov/pubmed/21693646?dopt=AbstractPlus, http://www.ncbi.nlm.nih.gov/pubmed/21727895?dopt=AbstractPlus], http://www.guidetopharmacology.org/GRAC/LigandDisplayForward?ligandId=3699 (https://www.genenames.org/data/gene‐symbol‐report/#!/hgnc_id/HGNC:20866, http://www.uniprot.org/uniprot/Q9BXY4) [http://www.ncbi.nlm.nih.gov/pubmed/21693646?dopt=AbstractPlus, http://www.ncbi.nlm.nih.gov/pubmed/21727895?dopt=AbstractPlus], http://www.guidetopharmacology.org/GRAC/LigandDisplayForward?ligandId=3700 (https://www.genenames.org/data/gene‐symbol‐report/#!/hgnc_id/HGNC:16175, http://www.uniprot.org/uniprot/Q2I0M5) [http://www.ncbi.nlm.nih.gov/pubmed/21693646?dopt=AbstractPlus, http://www.ncbi.nlm.nih.gov/pubmed/21727895?dopt=AbstractPlus]
Comments	LGR4 does not couple to heterotrimeric G proteins or recruit arrestins when stimulated by the R‐spondins, indicating a unique mechanism of action. R‐spondins bind to LGR4, which specifically associates with Frizzled and LDL receptor‐related proteins (LRPs) that are activated by the extracellular Wnt molecules and then trigger canonical Wnt signalling to increase gene expression [http://www.ncbi.nlm.nih.gov/pubmed/21693646?dopt=AbstractPlus, http://www.ncbi.nlm.nih.gov/pubmed/21727895?dopt=AbstractPlus, http://www.ncbi.nlm.nih.gov/pubmed/22815884?dopt=AbstractPlus]. Gene disruption leads to multiple developmental disorders [http://www.ncbi.nlm.nih.gov/pubmed/18487371?dopt=AbstractPlus, http://www.ncbi.nlm.nih.gov/pubmed/19605502?dopt=AbstractPlus, http://www.ncbi.nlm.nih.gov/pubmed/18955481?dopt=AbstractPlus, http://www.ncbi.nlm.nih.gov/pubmed/18424556?dopt=AbstractPlus].	The four R‐spondins can bind to LGR4, LGR5, and LGR6, which specifically associate with Frizzled and LDL receptor‐related proteins (LRPs), proteins that are activated by extracellular Wnt molecules and which then trigger canonical Wnt signalling to increase gene expression [http://www.ncbi.nlm.nih.gov/pubmed/21693646?dopt=AbstractPlus, http://www.ncbi.nlm.nih.gov/pubmed/21727895?dopt=AbstractPlus].	–

**Table:** 

Nomenclature	http://www.guidetopharmacology.org/GRAC/ObjectDisplayForward?objectId=150	http://www.guidetopharmacology.org/GRAC/ObjectDisplayForward?objectId=151
HGNC, UniProt	https://www.genenames.org/data/gene‐symbol‐report/#!/hgnc_id/HGNC:6899, http://www.uniprot.org/uniprot/P04201	https://www.genenames.org/data/gene‐symbol‐report/#!/hgnc_id/HGNC:13961, http://www.uniprot.org/uniprot/P35410
Agonists	http://www.guidetopharmacology.org/GRAC/LigandDisplayForward?ligandId=582) (https://www.genenames.org/data/gene‐symbol‐report/#!/hgnc_id/HGNC:333, http://www.uniprot.org/uniprot/P01019) [http://www.ncbi.nlm.nih.gov/pubmed/21670420?dopt=AbstractPlus] – Mouse	–

**Table:** 

Nomenclature	http://www.guidetopharmacology.org/GRAC/ObjectDisplayForward?objectId=152	http://www.guidetopharmacology.org/GRAC/ObjectDisplayForward?objectId=153	http://www.guidetopharmacology.org/GRAC/ObjectDisplayForward?objectId=154	http://www.guidetopharmacology.org/GRAC/ObjectDisplayForward?objectId=155
HGNC, UniProt	https://www.genenames.org/data/gene‐symbol‐report/#!/hgnc_id/HGNC:29626, http://www.uniprot.org/uniprot/Q8TDS7	https://www.genenames.org/data/gene‐symbol‐report/#!/hgnc_id/HGNC:30694, http://www.uniprot.org/uniprot/Q86SM8	https://www.genenames.org/data/gene‐symbol‐report/#!/hgnc_id/HGNC:24828, http://www.uniprot.org/uniprot/Q96AM1	https://www.genenames.org/data/gene‐symbol‐report/#!/hgnc_id/HGNC:24829, http://www.uniprot.org/uniprot/Q86SM5
Endogenous agonists	http://www.guidetopharmacology.org/GRAC/LigandDisplayForward?ligandId=2365 [http://www.ncbi.nlm.nih.gov/pubmed/15037633?dopt=AbstractPlus, http://www.ncbi.nlm.nih.gov/pubmed/23396314?dopt=AbstractPlus]	–	–	–
Comments	An endogenous peptide with a high degree of sequence similarity to http://www.guidetopharmacology.org/GRAC/LigandDisplayForward?ligandId=582) (https://www.genenames.org/data/gene‐symbol‐report/#!/hgnc_id/HGNC:333, http://www.uniprot.org/uniprot/P01019), http://www.guidetopharmacology.org/GRAC/LigandDisplayForward?ligandId=6065 (https://www.genenames.org/data/gene‐symbol‐report/#!/hgnc_id/HGNC:333), was shown to promote NO release in MRGPRD‐transfected cells. The binding of alamandine to MRGPRD to was shown to be blocked by D‐Pro^7^‐angiotensin‐(1‐7), http://www.guidetopharmacology.org/GRAC/LigandDisplayForward?ligandId=2365 and http://www.guidetopharmacology.org/GRAC/LigandDisplayForward?ligandId=597 [http://www.ncbi.nlm.nih.gov/pubmed/23446738?dopt=AbstractPlus]. Genetic ablation of MRGPRD+ neurons of adult mice decreased behavioural sensitivity to mechanical stimuli but not to thermal stimuli [http://www.ncbi.nlm.nih.gov/pubmed/19451647?dopt=AbstractPlus]. See reviews [http://www.ncbi.nlm.nih.gov/pubmed/23686350?dopt=AbstractPlus] and [http://www.ncbi.nlm.nih.gov/pubmed/24867890?dopt=AbstractPlus].	See reviews [http://www.ncbi.nlm.nih.gov/pubmed/23686350?dopt=AbstractPlus] and [http://www.ncbi.nlm.nih.gov/pubmed/24867890?dopt=AbstractPlus].	MRGPRF has been reported to respond to stimulation by angiotensin metabolites [http://www.ncbi.nlm.nih.gov/pubmed/18636314?dopt=AbstractPlus]. See reviews [http://www.ncbi.nlm.nih.gov/pubmed/23686350?dopt=AbstractPlus] and [http://www.ncbi.nlm.nih.gov/pubmed/24867890?dopt=AbstractPlus].	See reviews [http://www.ncbi.nlm.nih.gov/pubmed/23686350?dopt=AbstractPlus] and [http://www.ncbi.nlm.nih.gov/pubmed/24867890?dopt=AbstractPlus].

**Table:** 

Nomenclature	http://www.guidetopharmacology.org/GRAC/ObjectDisplayForward?objectId=156	http://www.guidetopharmacology.org/GRAC/ObjectDisplayForward?objectId=157	http://www.guidetopharmacology.org/GRAC/ObjectDisplayForward?objectId=158	http://www.guidetopharmacology.org/GRAC/ObjectDisplayForward?objectId=159	http://www.guidetopharmacology.org/GRAC/ObjectDisplayForward?objectId=164	http://www.guidetopharmacology.org/GRAC/ObjectDisplayForward?objectId=165
HGNC, UniProt	https://www.genenames.org/data/gene‐symbol‐report/#!/hgnc_id/HGNC:17962, http://www.uniprot.org/uniprot/Q96LB2	https://www.genenames.org/data/gene‐symbol‐report/#!/hgnc_id/HGNC:17983, http://www.uniprot.org/uniprot/Q96LB1	https://www.genenames.org/data/gene‐symbol‐report/#!/hgnc_id/HGNC:17980, http://www.uniprot.org/uniprot/Q96LB0	https://www.genenames.org/data/gene‐symbol‐report/#!/hgnc_id/HGNC:17617, http://www.uniprot.org/uniprot/Q96LA9	https://www.genenames.org/data/gene‐symbol‐report/#!/hgnc_id/HGNC:15524, http://www.uniprot.org/uniprot/Q86VZ1	https://www.genenames.org/data/gene‐symbol‐report/#!/hgnc_id/HGNC:19906, http://www.uniprot.org/uniprot/O00398
Endogenous agonists	http://www.guidetopharmacology.org/GRAC/LigandDisplayForward?ligandId=4058 (https://www.genenames.org/data/gene‐symbol‐report/#!/hgnc_id/HGNC:8831, http://www.uniprot.org/uniprot/P01210) [http://www.ncbi.nlm.nih.gov/pubmed/15163697?dopt=AbstractPlus, http://www.ncbi.nlm.nih.gov/pubmed/11850634?dopt=AbstractPlus, http://www.ncbi.nlm.nih.gov/pubmed/23396314?dopt=AbstractPlus]	http://www.guidetopharmacology.org/GRAC/LigandDisplayForward?ligandId=4057 (https://www.genenames.org/data/gene‐symbol‐report/#!/hgnc_id/HGNC:259, http://www.uniprot.org/uniprot/P35318) [http://www.ncbi.nlm.nih.gov/pubmed/15823563?dopt=AbstractPlus]	–	–	–	http://www.guidetopharmacology.org/GRAC/LigandDisplayForward?ligandId=911 [http://www.ncbi.nlm.nih.gov/pubmed/18466763?dopt=AbstractPlus], http://www.guidetopharmacology.org/GRAC/LigandDisplayForward?ligandId=2906 [http://www.ncbi.nlm.nih.gov/pubmed/18466763?dopt=AbstractPlus]
Agonists	–	http://www.guidetopharmacology.org/GRAC/LigandDisplayForward?ligandId=2007 {Mouse, Rat} [http://www.ncbi.nlm.nih.gov/pubmed/15823563?dopt=AbstractPlus, http://www.ncbi.nlm.nih.gov/pubmed/28288109?dopt=AbstractPlus, http://www.ncbi.nlm.nih.gov/pubmed/12915402?dopt=AbstractPlus, http://www.ncbi.nlm.nih.gov/pubmed/23396314?dopt=AbstractPlus]	–	–	–	–
Selective agonists	–	http://www.guidetopharmacology.org/GRAC/LigandDisplayForward?ligandId=4056) [http://www.ncbi.nlm.nih.gov/pubmed/15823563?dopt=AbstractPlus]	–	–	–	–
Comments	Reported to mediate the sensation of itch [http://www.ncbi.nlm.nih.gov/pubmed/20004959?dopt=AbstractPlus, http://www.ncbi.nlm.nih.gov/pubmed/21593341?dopt=AbstractPlus]. Reports that http://www.guidetopharmacology.org/GRAC/LigandDisplayForward?ligandId=4058 (https://www.genenames.org/data/gene‐symbol‐report/#!/hgnc_id/HGNC:8831, http://www.uniprot.org/uniprot/P01210) was the most potent of a series of proenkephalin A‐derived peptides as an agonist of MRGPRX1 in assays of calcium mobilisation and radioligand binding [http://www.ncbi.nlm.nih.gov/pubmed/11850634?dopt=AbstractPlus] were replicated in an independent study using an arrestin recruitment assay [http://www.ncbi.nlm.nih.gov/pubmed/23396314?dopt=AbstractPlus]. See reviews [http://www.ncbi.nlm.nih.gov/pubmed/23686350?dopt=AbstractPlus] and [http://www.ncbi.nlm.nih.gov/pubmed/24867890?dopt=AbstractPlus].	A diverse range of substances has been reported to be agonists of MRGPRX2, with cortistatin 14 the highest potency agonist in assays of calcium mobilisation [http://www.ncbi.nlm.nih.gov/pubmed/12915402?dopt=AbstractPlus], also confirmed in an independent study using an arrestin recruitment assay [http://www.ncbi.nlm.nih.gov/pubmed/23396314?dopt=AbstractPlus]. See reviews [http://www.ncbi.nlm.nih.gov/pubmed/23686350?dopt=AbstractPlus] and [http://www.ncbi.nlm.nih.gov/pubmed/24867890?dopt=AbstractPlus].	–	See reviews [http://www.ncbi.nlm.nih.gov/pubmed/23686350?dopt=AbstractPlus] and [http://www.ncbi.nlm.nih.gov/pubmed/24867890?dopt=AbstractPlus].	–	–

**Table:** 

Nomenclature	http://www.guidetopharmacology.org/GRAC/ObjectDisplayForward?objectId=167	http://www.guidetopharmacology.org/GRAC/ObjectDisplayForward?objectId=168	http://www.guidetopharmacology.org/GRAC/ObjectDisplayForward?objectId=169	http://www.guidetopharmacology.org/GRAC/ObjectDisplayForward?objectId=170	http://www.guidetopharmacology.org/GRAC/ObjectDisplayForward?objectId=171	http://www.guidetopharmacology.org/GRAC/ObjectDisplayForward?objectId=172	http://www.guidetopharmacology.org/GRAC/ObjectDisplayForward?objectId=173
HGNC, UniProt	https://www.genenames.org/data/gene‐symbol‐report/#!/hgnc_id/HGNC:4514, http://www.uniprot.org/uniprot/Q9P1P5	https://www.genenames.org/data/gene‐symbol‐report/#!/hgnc_id/HGNC:4513, http://www.uniprot.org/uniprot/Q9P1P4	https://www.genenames.org/data/gene‐symbol‐report/#!/hgnc_id/HGNC:31924, –	https://www.genenames.org/data/gene‐symbol‐report/#!/hgnc_id/HGNC:30236, http://www.uniprot.org/uniprot/O14804	https://www.genenames.org/data/gene‐symbol‐report/#!/hgnc_id/HGNC:20978, http://www.uniprot.org/uniprot/Q96RI8	https://www.genenames.org/data/gene‐symbol‐report/#!/hgnc_id/HGNC:14964, http://www.uniprot.org/uniprot/Q969N4	https://www.genenames.org/data/gene‐symbol‐report/#!/hgnc_id/HGNC:20977, http://www.uniprot.org/uniprot/Q96RI9
Potency order of endogenous ligands	http://www.guidetopharmacology.org/GRAC/LigandDisplayForward?ligandId=2144 > http://www.guidetopharmacology.org/GRAC/LigandDisplayForward?ligandId=125 [http://www.ncbi.nlm.nih.gov/pubmed/11459929?dopt=AbstractPlus]	–	–	–	–	–	–
Comments	Probable pseudogene in 10–15% of Asians due to a polymorphism (rs8192646) producing a premature stop codon at amino acid 168 [http://www.ncbi.nlm.nih.gov/pubmed/23686350?dopt=AbstractPlus].	*TAAR3* is thought to be a pseudogene in man though functional in rodents [http://www.ncbi.nlm.nih.gov/pubmed/23686350?dopt=AbstractPlus].	Pseudogene in man but functional in rodents [http://www.ncbi.nlm.nih.gov/pubmed/23686350?dopt=AbstractPlus].	Trimethylamine is reported as an agonist [http://www.ncbi.nlm.nih.gov/pubmed/23393561?dopt=AbstractPlus] and 3‐iodothyronamine an inverse agonist [http://www.ncbi.nlm.nih.gov/pubmed/25706283?dopt=AbstractPlus].	–	–	*TAAR9* appears to be functional in most individuals but has a polymorphic premature stop codon at amino acid 61 (rs2842899) with an allele frequency of 10–30% in different populations [http://www.ncbi.nlm.nih.gov/pubmed/14559210?dopt=AbstractPlus].

#### Further reading on Class A Orphans

McNeil BD *et al*. (2015) Identification of a mast‐cell‐specific receptor crucial for pseudo‐allergic drug reactions. *Nature* **519**: 237–41 [https://www.ncbi.nlm.nih.gov/pubmed/25517090?dopt=AbstractPlus]

## http://www.guidetopharmacology.org/GRAC/FamilyDisplayForward?familyId=18

### Overview

This set contains class C ’orphan’ G protein coupled receptors where the endogenous ligand(s) is not known.

**Table:** 

Nomenclature http://www.guidetopharmacology.org/GRAC/ObjectDisplayForward?objectId=209	http://www.guidetopharmacology.org/GRAC/ObjectDisplayForward?objectId=210	http://www.guidetopharmacology.org/GRAC/ObjectDisplayForward?objectId=211	http://www.guidetopharmacology.org/GRAC/ObjectDisplayForward?objectId=258	http://www.guidetopharmacology.org/GRAC/ObjectDisplayForward?objectId=259	http://www.guidetopharmacology.org/GRAC/ObjectDisplayForward?objectId=260	http://www.guidetopharmacology.org/GRAC/ObjectDisplayForward?objectId=261	http://www.guidetopharmacology.org/GRAC/ObjectDisplayForward?objectId=55
HGNC, UniProt https://www.genenames.org/data/gene‐symbol‐report/#!/hgnc_id/HGNC:20844, http://www.uniprot.org/uniprot/Q8NFN8	https://www.genenames.org/data/gene‐symbol‐report/#!/hgnc_id/HGNC:23689, http://www.uniprot.org/uniprot/Q5T848	https://www.genenames.org/data/gene‐symbol‐report/#!/hgnc_id/HGNC:31371, http://www.uniprot.org/uniprot/Q6PRD1	https://www.genenames.org/data/gene‐symbol‐report/#!/hgnc_id/HGNC:9836, http://www.uniprot.org/uniprot/Q8NFJ5	https://www.genenames.org/data/gene‐symbol‐report/#!/hgnc_id/HGNC:13308, http://www.uniprot.org/uniprot/Q9NZH0	https://www.genenames.org/data/gene‐symbol‐report/#!/hgnc_id/HGNC:13309, http://www.uniprot.org/uniprot/Q9NQ84	https://www.genenames.org/data/gene‐symbol‐report/#!/hgnc_id/HGNC:13310, http://www.uniprot.org/uniprot/Q9NZD1	https://www.genenames.org/data/gene‐symbol‐report/#!/hgnc_id/HGNC:18510, http://www.uniprot.org/uniprot/Q5T6X5
Comments	–	–	–	–	–	–	GPRC6 is a G_q_‐coupled receptor which responds to basic amino acids [http://www.ncbi.nlm.nih.gov/pubmed/15576628?dopt=AbstractPlus].

### Further reading on Class C Orphans

Harpse K *et al*. (2017) Structural insight to mutation effects uncover a common allosteric site in class C GPCRs. *Bioinformatics* **33**: 1116–1120 [https://www.ncbi.nlm.nih.gov/pubmed/28011766?dopt=AbstractPlus]

## http://www.guidetopharmacology.org/GRAC/FamilyDisplayForward?familyId=116

### Overview

Whilst the taste of acid and salty foods appear to be sensed by regulation of ion channel activity, bitter, sweet and umami tastes are sensed by specialised GPCR. Two classes of taste GPCR have been identified, T1R and T2R, which are similar in sequence and structure to Class C and Class A GPCR, respectively. Activation of taste receptors appears to involve gustducin‐ (Gαt3) and Gα14‐mediated signalling, although the precise mechanisms remain obscure. Gene disruption studies suggest the involvement of PLCβ2 [http://www.ncbi.nlm.nih.gov/pubmed/12581520?dopt=AbstractPlus], TRPM5 [http://www.ncbi.nlm.nih.gov/pubmed/12581520?dopt=AbstractPlus] and IP3 [http://www.ncbi.nlm.nih.gov/pubmed/17925404?dopt=AbstractPlus] receptors in postreceptor signalling of taste receptors. Although predominantly associated with the oral cavity, taste receptors are also located elsewhere, including further down the gastrointestinal system, in the lungs and in the brain.

### Sweet/Umami

T1R3 acts as an obligate partner in T1R1/T1R3 and T1R2/T1R3 heterodimers, which sense umami or sweet, respectively. T1R1/T1R3 heterodimers respond to http://www.guidetopharmacology.org/GRAC/LigandDisplayForward?ligandId=1369 and may be positively allosterically modulated by 5’‐nucleoside monophosphates, such as http://www.guidetopharmacology.org/GRAC/LigandDisplayForward?ligandId=5123 [http://www.ncbi.nlm.nih.gov/pubmed/12013525?dopt=AbstractPlus]. T1R2/T1R3 heterodimers respond to sugars, such as http://www.guidetopharmacology.org/GRAC/LigandDisplayForward?ligandId=5411, and artificial sweeteners, such as http://www.guidetopharmacology.org/GRAC/LigandDisplayForward?ligandId=5432 [http://www.ncbi.nlm.nih.gov/pubmed/11509186?dopt=AbstractPlus].

**Table:** 

Nomenclature	http://www.guidetopharmacology.org/GRAC/ObjectDisplayForward?objectId=656	http://www.guidetopharmacology.org/GRAC/ObjectDisplayForward?objectId=657	http://www.guidetopharmacology.org/GRAC/ObjectDisplayForward?objectId=658
HGNC, UniProt	https://www.genenames.org/data/gene‐symbol‐report/#!/hgnc_id/HGNC:14448, http://www.uniprot.org/uniprot/Q7RTX1	https://www.genenames.org/data/gene‐symbol‐report/#!/hgnc_id/HGNC:14905, http://www.uniprot.org/uniprot/Q8TE23	https://www.genenames.org/data/gene‐symbol‐report/#!/hgnc_id/HGNC:15661, http://www.uniprot.org/uniprot/Q7RTX0

### Comments

Positive allosteric modulators of T1R2/T1R3 have been reported [2363]. Such compounds enhance the sweet taste of sucrose mediated by these receptors, but are tasteless on their own.

### Further reading on Taste 1 receptors

Palmer RK. (2019) A Pharmacological Perspective on the Study of Taste. *Pharmacol. Rev.* **71**: 20–48 [https://www.ncbi.nlm.nih.gov/pubmed/30559245?dopt=AbstractPlus]

## http://www.guidetopharmacology.org/GRAC/FamilyDisplayForward?familyId=117

G protein‐coupled receptors → Orphan and other 7TM receptors → Taste 2 receptors

## Overview

The composition and stoichiometry of bitter taste receptors is not yet established. Bitter receptors appear to separate into two groups, with very restricted ligand specificity or much broader responsiveness. For example, T2R5 responded to http://www.guidetopharmacology.org/GRAC/LigandDisplayForward?ligandId=5433, but not 10 other bitter compounds [http://www.ncbi.nlm.nih.gov/pubmed/10761935?dopt=AbstractPlus], while T2R14 responded to at least eight different bitter tastants, including (‐)‐http://www.guidetopharmacology.org/GRAC/LigandDisplayForward?ligandId=5344‐http://www.guidetopharmacology.org/GRAC/LigandDisplayForward?ligandId=5344 and http://www.guidetopharmacology.org/GRAC/LigandDisplayForward?ligandId=2291 [http://www.ncbi.nlm.nih.gov/pubmed/15178431?dopt=AbstractPlus].

Specialist database http://bitterdb.agri.huji.ac.il/dbbitter.php contains additional information on bitter compounds and receptors [http://www.ncbi.nlm.nih.gov/pubmed/21940398?dopt=AbstractPlus].

**Table:** 

Nomenclature	http://www.guidetopharmacology.org/GRAC/ObjectDisplayForward?objectId=659	http://www.guidetopharmacology.org/GRAC/ObjectDisplayForward?objectId=660	http://www.guidetopharmacology.org/GRAC/ObjectDisplayForward?objectId=661	http://www.guidetopharmacology.org/GRAC/ObjectDisplayForward?objectId=662	http://www.guidetopharmacology.org/GRAC/ObjectDisplayForward?objectId=663	http://www.guidetopharmacology.org/GRAC/ObjectDisplayForward?objectId=664	http://www.guidetopharmacology.org/GRAC/ObjectDisplayForward?objectId=665
HGNC, UniProt	https://www.genenames.org/data/gene‐symbol‐report/#!/hgnc_id/HGNC:14909, http://www.uniprot.org/uniprot/Q9NYW7	https://www.genenames.org/data/gene‐symbol‐report/#!/hgnc_id/HGNC:14910, http://www.uniprot.org/uniprot/Q9NYW6	https://www.genenames.org/data/gene‐symbol‐report/#!/hgnc_id/HGNC:14911, http://www.uniprot.org/uniprot/Q9NYW5	https://www.genenames.org/data/gene‐symbol‐report/#!/hgnc_id/HGNC:14912, http://www.uniprot.org/uniprot/Q9NYW4	https://www.genenames.org/data/gene‐symbol‐report/#!/hgnc_id/HGNC:14913, http://www.uniprot.org/uniprot/Q9NYW3	https://www.genenames.org/data/gene‐symbol‐report/#!/hgnc_id/HGNC:14915, http://www.uniprot.org/uniprot/Q9NYW2	https://www.genenames.org/data/gene‐symbol‐report/#!/hgnc_id/HGNC:14917, http://www.uniprot.org/uniprot/Q9NYW1

**Table:** 

Nomenclature	http://www.guidetopharmacology.org/GRAC/ObjectDisplayForward?objectId=666	http://www.guidetopharmacology.org/GRAC/ObjectDisplayForward?objectId=667	http://www.guidetopharmacology.org/GRAC/ObjectDisplayForward?objectId=668	http://www.guidetopharmacology.org/GRAC/ObjectDisplayForward?objectId=669	http://www.guidetopharmacology.org/GRAC/ObjectDisplayForward?objectId=670	http://www.guidetopharmacology.org/GRAC/ObjectDisplayForward?objectId=671	http://www.guidetopharmacology.org/GRAC/ObjectDisplayForward?objectId=673
HGNC, UniProt	https://www.genenames.org/data/gene‐symbol‐report/#!/hgnc_id/HGNC:14918, http://www.uniprot.org/uniprot/Q9NYW0	https://www.genenames.org/data/gene‐symbol‐report/#!/hgnc_id/HGNC:14919, http://www.uniprot.org/uniprot/Q9NYV9	https://www.genenames.org/data/gene‐symbol‐report/#!/hgnc_id/HGNC:14920, http://www.uniprot.org/uniprot/Q9NYV8	https://www.genenames.org/data/gene‐symbol‐report/#!/hgnc_id/HGNC:14921, http://www.uniprot.org/uniprot/Q9NYV7	https://www.genenames.org/data/gene‐symbol‐report/#!/hgnc_id/HGNC:19108, http://www.uniprot.org/uniprot/P59542	https://www.genenames.org/data/gene‐symbol‐report/#!/hgnc_id/HGNC:19109, http://www.uniprot.org/uniprot/P59543	https://www.genenames.org/data/gene‐symbol‐report/#!/hgnc_id/HGNC:19112, http://www.uniprot.org/uniprot/P59541

**Table:** 

Nomenclature	http://www.guidetopharmacology.org/GRAC/ObjectDisplayForward?objectId=674	http://www.guidetopharmacology.org/GRAC/ObjectDisplayForward?objectId=682	http://www.guidetopharmacology.org/GRAC/ObjectDisplayForward?objectId=675	http://www.guidetopharmacology.org/GRAC/ObjectDisplayForward?objectId=676
HGNC, UniProt	https://www.genenames.org/data/gene‐symbol‐report/#!/hgnc_id/HGNC:19113, http://www.uniprot.org/uniprot/P59538	https://www.genenames.org/data/gene‐symbol‐report/#!/hgnc_id/HGNC:9584, http://www.uniprot.org/uniprot/P59533	https://www.genenames.org/data/gene‐symbol‐report/#!/hgnc_id/HGNC:18886, http://www.uniprot.org/uniprot/P59534	https://www.genenames.org/data/gene‐symbol‐report/#!/hgnc_id/HGNC:18885, http://www.uniprot.org/uniprot/P59535
Antagonists	http://www.guidetopharmacology.org/GRAC/LigandDisplayForward?ligandId=9111 (pIC_50_ 5.6) [http://www.ncbi.nlm.nih.gov/pubmed/21650152?dopt=AbstractPlus], http://www.guidetopharmacology.org/GRAC/LigandDisplayForward?ligandId=9112 (pIC_50_ 5.1–5.2) [http://www.ncbi.nlm.nih.gov/pubmed/20537538?dopt=AbstractPlus]	–	–	–

**Table:** 

Nomenclature	http://www.guidetopharmacology.org/GRAC/ObjectDisplayForward?objectId=680	http://www.guidetopharmacology.org/GRAC/ObjectDisplayForward?objectId=672	http://www.guidetopharmacology.org/GRAC/ObjectDisplayForward?objectId=678	http://www.guidetopharmacology.org/GRAC/ObjectDisplayForward?objectId=2828	http://www.guidetopharmacology.org/GRAC/ObjectDisplayForward?objectId=679	http://www.guidetopharmacology.org/GRAC/ObjectDisplayForward?objectId=677	http://www.guidetopharmacology.org/GRAC/ObjectDisplayForward?objectId=681
HGNC, UniProt	https://www.genenames.org/data/gene‐symbol‐report/#!/hgnc_id/HGNC:18883, http://www.uniprot.org/uniprot/P59536	https://www.genenames.org/data/gene‐symbol‐report/#!/hgnc_id/HGNC:18888, http://www.uniprot.org/uniprot/Q7RTR8	https://www.genenames.org/data/gene‐symbol‐report/#!/hgnc_id/HGNC:18875, http://www.uniprot.org/uniprot/P59537	https://www.genenames.org/data/gene‐symbol‐report/#!/hgnc_id/HGNC:18876, http://www.uniprot.org/uniprot/P59539	https://www.genenames.org/data/gene‐symbol‐report/#!/hgnc_id/HGNC:18877, http://www.uniprot.org/uniprot/P59540	https://www.genenames.org/data/gene‐symbol‐report/#!/hgnc_id/HGNC:18882, http://www.uniprot.org/uniprot/P59544	https://www.genenames.org/data/gene‐symbol‐report/#!/hgnc_id/HGNC:20639, http://www.uniprot.org/uniprot/P59551

### Further reading on Taste 2 receptors

Palmer RK. (2019) A Pharmacological Perspective on the Study of Taste. *Pharmacol. Rev.* **71**: 20–48 [https://www.ncbi.nlm.nih.gov/pubmed/30559245?dopt=AbstractPlus]

## http://www.guidetopharmacology.org/GRAC/FamilyDisplayForward?familyId=113

**Table:** 

Nomenclature	http://www.guidetopharmacology.org/GRAC/ObjectDisplayForward?objectId=651	http://www.guidetopharmacology.org/GRAC/ObjectDisplayForward?objectId=652	http://www.guidetopharmacology.org/GRAC/ObjectDisplayForward?objectId=655	http://www.guidetopharmacology.org/GRAC/ObjectDisplayForward?objectId=203	http://www.guidetopharmacology.org/GRAC/ObjectDisplayForward?objectId=205
HGNC, UniProt	https://www.genenames.org/data/gene‐symbol‐report/#!/hgnc_id/HGNC:17830, http://www.uniprot.org/uniprot/Q5VW38	https://www.genenames.org/data/gene‐symbol‐report/#!/hgnc_id/HGNC:24300, http://www.uniprot.org/uniprot/Q96N19	https://www.genenames.org/data/gene‐symbol‐report/#!/hgnc_id/HGNC:30413, http://www.uniprot.org/uniprot/Q86W33	https://www.genenames.org/data/gene‐symbol‐report/#!/hgnc_id/HGNC:20145, http://www.uniprot.org/uniprot/P51810	https://www.genenames.org/data/gene‐symbol‐report/#!/hgnc_id/HGNC:23687, http://www.uniprot.org/uniprot/Q5UAW9
Endogenous agonists	–	–	–	http://www.guidetopharmacology.org/GRAC/LigandDisplayForward?ligandId=3639 [http://www.ncbi.nlm.nih.gov/pubmed/18828673?dopt=AbstractPlus]	–
Comments	GPR107 is a member of the LUSTR family of proteins found in both plants and animals, having similar topology to G protein‐coupled receptors [http://www.ncbi.nlm.nih.gov/pubmed/17454009?dopt=AbstractPlus]	–	TPRA1 shows no homology to known G protein‐coupled receptors.	Loss‐of‐function mutations underlie ocular albinism type 1 [http://www.ncbi.nlm.nih.gov/pubmed/7647783?dopt=AbstractPlus].	GPR157 has ambiguous sequence similarities to several different GPCR families (class A, class B and the slime mould cyclic AMP receptor). Because of its distant relationship to other GPCRs, it cannot be readily classified.

### Further reading on Orphan and other 7TM receptors

Davenport AP *et al*. (2013) International Union of Basic and Clinical Pharmacology. LXXXVIII. G protein‐coupled receptor list: recommendations for new pairings with cognate ligands. *Pharmacol. Rev.* **65**: 967–86 [https://www.ncbi.nlm.nih.gov/pubmed/23686350?dopt=AbstractPlus]

Gilissen J *et al*. (2016) Insight into SUCNR1 (GPR91) structure and function. *Pharmacol. Ther.* **159**: 56–65 [https://www.ncbi.nlm.nih.gov/pubmed/26808164?dopt=AbstractPlus]

Insel PA *et al*. (2015) G Protein‐Coupled Receptor (GPCR) Expression in Native Cells: “Novel” endoGPCRs as Physiologic Regulators and Therapeutic Targets. *Mol. Pharmacol.* **88**: 181‐7 [https://www.ncbi.nlm.nih.gov/pubmed/25737495?dopt=AbstractPlus]

Khan MZ *et al*. (2017) Neuro‐psychopharmacological perspective of Orphan receptors of Rhodopsin (class A) family of G protein‐coupled receptors. *Psychopharmacology (Berl.)* **234**: 1181‐1207 [https://www.ncbi.nlm.nih.gov/pubmed/28289782?dopt=AbstractPlus]

Mackenzie AE *et al*. (2017) The emerging pharmacology and function of GPR35 in the nervous system. *Neuropharmacology* **113**: 661–671 [https://www.ncbi.nlm.nih.gov/pubmed/26232640?dopt=AbstractPlus]

Ngo T *et al*. (2016) Identifying ligands at orphan GPCRs: current status using structure‐based approaches. *Br. J. Pharmacol.* **173**: 2934‐51 [https://www.ncbi.nlm.nih.gov/pubmed/26837045?dopt=AbstractPlus]

## http://www.guidetopharmacology.org/GRAC/FamilyDisplayForward?familyId=1

### Overview

5‐HT receptors (**nomenclature as agreed by the NC‐IUPHAR Subcommittee on 5‐HT receptors** [http://www.ncbi.nlm.nih.gov/pubmed/7938165?dopt=AbstractPlus] **and subsequently revised** [http://www.ncbi.nlm.nih.gov/pubmed/8936345?dopt=AbstractPlus]) are, with the exception of the ionotropic 5‐HT_3_ class, GPCRs where the endogenous agonist is http://www.guidetopharmacology.org/GRAC/LigandDisplayForward?ligandId=5. The diversity of metabotropic 5‐HT receptors is increased by alternative splicing that produces isoforms of the 5‐HT_2A_ (non‐functional), 5‐HT_2C_ (non‐functional), 5‐HT_4_, 5‐HT_6_ (non‐functional) and 5‐HT_7_ receptors. Unique amongst the GPCRs, RNA editing produces 5‐HT_2C_ receptor isoforms that differ in function, such as efficiency and specificity of coupling to G_q/11_ and also pharmacology [http://www.ncbi.nlm.nih.gov/pubmed/16896947?dopt=AbstractPlus, http://www.ncbi.nlm.nih.gov/pubmed/18554725?dopt=AbstractPlus]. Most 5‐HT receptors (except 5‐ht_1e_ and 5‐ht_5b_) play specific roles mediating functional responses in different tissues (reviewed by [http://www.ncbi.nlm.nih.gov/pubmed/19086344?dopt=AbstractPlus, http://www.ncbi.nlm.nih.gov/pubmed/17703282?dopt=AbstractPlus]).

**Table:** 

Nomenclature	http://www.guidetopharmacology.org/GRAC/ObjectDisplayForward?objectId=1	http://www.guidetopharmacology.org/GRAC/ObjectDisplayForward?objectId=2
HGNC, UniProt	https://www.genenames.org/data/gene‐symbol‐report/#!/hgnc_id/HGNC:5286, http://www.uniprot.org/uniprot/P08908	https://www.genenames.org/data/gene‐symbol‐report/#!/hgnc_id/HGNC:5287, http://www.uniprot.org/uniprot/P28222
Agonists	http://www.guidetopharmacology.org/GRAC/LigandDisplayForward?ligandId=30 [http://www.ncbi.nlm.nih.gov/pubmed/7965808?dopt=AbstractPlus], http://www.guidetopharmacology.org/GRAC/LigandDisplayForward?ligandId=7427 (Partial agonist) [http://www.ncbi.nlm.nih.gov/pubmed/19499624?dopt=AbstractPlus], http://www.guidetopharmacology.org/GRAC/LigandDisplayForward?ligandId=7351 (Partial agonist) [http://www.ncbi.nlm.nih.gov/pubmed/21486038?dopt=AbstractPlus]	http://www.guidetopharmacology.org/GRAC/LigandDisplayForward?ligandId=15 [http://www.ncbi.nlm.nih.gov/pubmed/10513577?dopt=AbstractPlus], http://www.guidetopharmacology.org/GRAC/LigandDisplayForward?ligandId=45 (Partial agonist) [http://www.ncbi.nlm.nih.gov/pubmed/10193663?dopt=AbstractPlus], http://www.guidetopharmacology.org/GRAC/LigandDisplayForward?ligandId=40[http://www.ncbi.nlm.nih.gov/pubmed/10193663?dopt=AbstractPlus], http://www.guidetopharmacology.org/GRAC/LigandDisplayForward?ligandId=7191 [http://www.ncbi.nlm.nih.gov/pubmed/9986723?dopt=AbstractPlus], http://www.guidetopharmacology.org/GRAC/LigandDisplayForward?ligandId=60 (Partial agonist) [http://www.ncbi.nlm.nih.gov/pubmed/10193663?dopt=AbstractPlus], http://www.guidetopharmacology.org/GRAC/LigandDisplayForward?ligandId=7351 (Partial agonist) [http://www.ncbi.nlm.nih.gov/pubmed/21486038?dopt=AbstractPlus], http://www.guidetopharmacology.org/GRAC/LigandDisplayForward?ligandId=51 (Partial agonist) [http://www.ncbi.nlm.nih.gov/pubmed/10193663?dopt=AbstractPlus]
Selective agonists	http://www.guidetopharmacology.org/GRAC/LigandDisplayForward?ligandId=7 [http://www.ncbi.nlm.nih.gov/pubmed/9495870?dopt=AbstractPlus, http://www.ncbi.nlm.nih.gov/pubmed/12527336?dopt=AbstractPlus, http://www.ncbi.nlm.nih.gov/pubmed/15628665?dopt=AbstractPlus, http://www.ncbi.nlm.nih.gov/pubmed/9067310?dopt=AbstractPlus, http://www.ncbi.nlm.nih.gov/pubmed/10611634?dopt=AbstractPlus, http://www.ncbi.nlm.nih.gov/pubmed/9760039?dopt=AbstractPlus, http://www.ncbi.nlm.nih.gov/pubmed/9550290?dopt=AbstractPlus, http://www.ncbi.nlm.nih.gov/pubmed/1386736?dopt=AbstractPlus], http://www.guidetopharmacology.org/GRAC/LigandDisplayForward?ligandId=3924 [http://www.ncbi.nlm.nih.gov/pubmed/19154445?dopt=AbstractPlus]	http://www.guidetopharmacology.org/GRAC/LigandDisplayForward?ligandId=3221 [1122]
Antagonists	(http://www.guidetopharmacology.org/GRAC/LigandDisplayForward?ligandId=61 (p*K*_i_ 7.9) [http://www.ncbi.nlm.nih.gov/pubmed/9760039?dopt=AbstractPlus]	–
Selective antagonists	http://www.guidetopharmacology.org/GRAC/LigandDisplayForward?ligandId=80 (p*K*_i_ 7.9–9.2) [http://www.ncbi.nlm.nih.gov/pubmed/9760039?dopt=AbstractPlus, http://www.ncbi.nlm.nih.gov/pubmed/9550290?dopt=AbstractPlus], http://www.guidetopharmacology.org/GRAC/LigandDisplayForward?ligandId=72 (p*K*_i_ 9.2) [http://www.ncbi.nlm.nih.gov/pubmed/9336327?dopt=AbstractPlus]	http://www.guidetopharmacology.org/GRAC/LigandDisplayForward?ligandId=130 (Inverse agonist) (p*K*_i_ 8.2–8.6) [http://www.ncbi.nlm.nih.gov/pubmed/9548813?dopt=AbstractPlus, http://www.ncbi.nlm.nih.gov/pubmed/11040052?dopt=AbstractPlus, http://www.ncbi.nlm.nih.gov/pubmed/9776361?dopt=AbstractPlus], http://www.guidetopharmacology.org/GRAC/LigandDisplayForward?ligandId=3231 (Inverse agonist) (p*K*_i_ 8.2) [http://www.ncbi.nlm.nih.gov/pubmed/10443589?dopt=AbstractPlus], http://www.guidetopharmacology.org/GRAC/LigandDisplayForward?ligandId=113 (p*K*_B_ 7.4) [http://www.ncbi.nlm.nih.gov/pubmed/11888546?dopt=AbstractPlus]
Labelled ligands	[http://www.guidetopharmacology.org/GRAC/LigandDisplayForward?ligandId=82 (Antagonist) (p*K*_d_ 9.8) [http://www.ncbi.nlm.nih.gov/pubmed/9851589?dopt=AbstractPlus], [http://www.guidetopharmacology.org/GRAC/LigandDisplayForward?ligandId=3251 (Antagonist) (p*K*_d_ 9.5) [http://www.ncbi.nlm.nih.gov/pubmed/9048968?dopt=AbstractPlus], [http://www.guidetopharmacology.org/GRAC/LigandDisplayForward?ligandId=31 (Agonist) [http://www.ncbi.nlm.nih.gov/pubmed/11101361?dopt=AbstractPlus, http://www.ncbi.nlm.nih.gov/pubmed/15628665?dopt=AbstractPlus, http://www.ncbi.nlm.nih.gov/pubmed/10431754?dopt=AbstractPlus, http://www.ncbi.nlm.nih.gov/pubmed/9550290?dopt=AbstractPlus], [http://www.guidetopharmacology.org/GRAC/LigandDisplayForward?ligandId=3925 (Agonist) [http://www.ncbi.nlm.nih.gov/pubmed/20799027?dopt=AbstractPlus], [[http://www.guidetopharmacology.org/GRAC/LigandDisplayForward?ligandId=3252 (Antagonist) [http://www.ncbi.nlm.nih.gov/pubmed/15689356?dopt=AbstractPlus], http://www.guidetopharmacology.org/GRAC/LigandDisplayForward?ligandId=3227 (Antagonist) [http://www.ncbi.nlm.nih.gov/pubmed/16330560?dopt=AbstractPlus]	[http://www.guidetopharmacology.org/GRAC/LigandDisplayForward?ligandId=3219 (Agonist, Partial agonist) [http://www.ncbi.nlm.nih.gov/pubmed/19401496?dopt=AbstractPlus], [http://www.guidetopharmacology.org/GRAC/LigandDisplayForward?ligandId=131 (Selective Antagonist) (p*K*_d_ 8.6–9.2) [http://www.ncbi.nlm.nih.gov/pubmed/10513577?dopt=AbstractPlus, http://www.ncbi.nlm.nih.gov/pubmed/10452531?dopt=AbstractPlus], [http://www.guidetopharmacology.org/GRAC/LigandDisplayForward?ligandId=117 (Agonist) [http://www.ncbi.nlm.nih.gov/pubmed/9605573?dopt=AbstractPlus], [http://www.guidetopharmacology.org/GRAC/LigandDisplayForward?ligandId=3250 (Agonist) [http://www.ncbi.nlm.nih.gov/pubmed/1738002?dopt=AbstractPlus, http://www.ncbi.nlm.nih.gov/pubmed/8361548?dopt=AbstractPlus] – Rat, [http://www.guidetopharmacology.org/GRAC/LigandDisplayForward?ligandId=118 (Agonist, Partial agonist) [http://www.ncbi.nlm.nih.gov/pubmed/10193663?dopt=AbstractPlus], [http://www.guidetopharmacology.org/GRAC/LigandDisplayForward?ligandId=119 (Agonist, Partial agonist) [http://www.ncbi.nlm.nih.gov/pubmed/10193663?dopt=AbstractPlus], [http://www.guidetopharmacology.org/GRAC/LigandDisplayForward?ligandId=3926 (Agonist, Partial agonist) [http://www.ncbi.nlm.nih.gov/pubmed/20424633?dopt=AbstractPlus]

**Table:** 

Nomenclature	http://www.guidetopharmacology.org/GRAC/ObjectDisplayForward?objectId=3	http://www.guidetopharmacology.org/GRAC/ObjectDisplayForward?objectId=4	http://www.guidetopharmacology.org/GRAC/ObjectDisplayForward?objectId=5
HGNC, UniProt	https://www.genenames.org/data/gene‐symbol‐report/#!/hgnc_id/HGNC:5289, http://www.uniprot.org/uniprot/P28221	https://www.genenames.org/data/gene‐symbol‐report/#!/hgnc_id/HGNC:5291, http://www.uniprot.org/uniprot/P28566	https://www.genenames.org/data/gene‐symbol‐report/#!/hgnc_id/HGNC:5292, http://www.uniprot.org/uniprot/P30939
Agonists	http://www.guidetopharmacology.org/GRAC/LigandDisplayForward?ligandId=121 [http://www.ncbi.nlm.nih.gov/pubmed/1652050?dopt=AbstractPlus, http://www.ncbi.nlm.nih.gov/pubmed/9605573?dopt=AbstractPlus, http://www.ncbi.nlm.nih.gov/pubmed/8967979?dopt=AbstractPlus], http://www.guidetopharmacology.org/GRAC/LigandDisplayForward?ligandId=149 [http://www.ncbi.nlm.nih.gov/pubmed/10715164?dopt=AbstractPlus], http://www.guidetopharmacology.org/GRAC/LigandDisplayForward?ligandId=15 [http://www.ncbi.nlm.nih.gov/pubmed/9855638?dopt=AbstractPlus], http://www.guidetopharmacology.org/GRAC/LigandDisplayForward?ligandId=45 [http://www.ncbi.nlm.nih.gov/pubmed/9303569?dopt=AbstractPlus, http://www.ncbi.nlm.nih.gov/pubmed/10193663?dopt=AbstractPlus, http://www.ncbi.nlm.nih.gov/pubmed/7780656?dopt=AbstractPlus], http://www.guidetopharmacology.org/GRAC/LigandDisplayForward?ligandId=60[http://www.ncbi.nlm.nih.gov/pubmed/10193663?dopt=AbstractPlus], http://www.guidetopharmacology.org/GRAC/LigandDisplayForward?ligandId=7191 [http://www.ncbi.nlm.nih.gov/pubmed/9986723?dopt=AbstractPlus], http://www.guidetopharmacology.org/GRAC/LigandDisplayForward?ligandId=51[http://www.ncbi.nlm.nih.gov/pubmed/10193663?dopt=AbstractPlus]	http://www.guidetopharmacology.org/GRAC/LigandDisplayForward?ligandId=3927 [250]	http://www.guidetopharmacology.org/GRAC/LigandDisplayForward?ligandId=3927 [250], http://www.guidetopharmacology.org/GRAC/LigandDisplayForward?ligandId=40 [http://www.ncbi.nlm.nih.gov/pubmed/10193663?dopt=AbstractPlus], http://www.guidetopharmacology.org/GRAC/LigandDisplayForward?ligandId=54 [http://www.ncbi.nlm.nih.gov/pubmed/9225282?dopt=AbstractPlus, http://www.ncbi.nlm.nih.gov/pubmed/8380639?dopt=AbstractPlus, http://www.ncbi.nlm.nih.gov/pubmed/10193663?dopt=AbstractPlus, http://www.ncbi.nlm.nih.gov/pubmed/15900510?dopt=AbstractPlus]
Selective agonists	http://www.guidetopharmacology.org/GRAC/LigandDisplayForward?ligandId=3228 [http://www.ncbi.nlm.nih.gov/pubmed/9632349?dopt=AbstractPlus] – Gorilla, http://www.guidetopharmacology.org/GRAC/LigandDisplayForward?ligandId=40 [http://www.ncbi.nlm.nih.gov/pubmed/10193663?dopt=AbstractPlus]	–	http://www.guidetopharmacology.org/GRAC/LigandDisplayForward?ligandId=3928 [http://www.ncbi.nlm.nih.gov/pubmed/20855361?dopt=AbstractPlus], http://www.guidetopharmacology.org/GRAC/LigandDisplayForward?ligandId=20 [http://www.ncbi.nlm.nih.gov/pubmed/15900510?dopt=AbstractPlus], http://www.guidetopharmacology.org/GRAC/LigandDisplayForward?ligandId=8422 [http://www.ncbi.nlm.nih.gov/pubmed/21422162?dopt=AbstractPlus], http://www.guidetopharmacology.org/GRAC/LigandDisplayForward?ligandId=21 [http://www.ncbi.nlm.nih.gov/pubmed/9395253?dopt=AbstractPlus]
Selective antagonists	http://www.guidetopharmacology.org/GRAC/LigandDisplayForward?ligandId=77 (p*K*_i_ 9.1) [http://www.ncbi.nlm.nih.gov/pubmed/15887956?dopt=AbstractPlus]	–	–
Labelled ligands	[http://www.guidetopharmacology.org/GRAC/LigandDisplayForward?ligandId=118 (Agonist) [http://www.ncbi.nlm.nih.gov/pubmed/10193663?dopt=AbstractPlus], [http://www.guidetopharmacology.org/GRAC/LigandDisplayForward?ligandId=117 (Agonist) [http://www.ncbi.nlm.nih.gov/pubmed/9605573?dopt=AbstractPlus], [http://www.guidetopharmacology.org/GRAC/LigandDisplayForward?ligandId=3250 (Selective Agonist) [http://www.ncbi.nlm.nih.gov/pubmed/1738002?dopt=AbstractPlus, http://www.ncbi.nlm.nih.gov/pubmed/8361548?dopt=AbstractPlus] – Rat, [http://www.guidetopharmacology.org/GRAC/LigandDisplayForward?ligandId=131 (Selective Antagonist) (p*K*_d_ 8.6) [http://www.ncbi.nlm.nih.gov/pubmed/10452531?dopt=AbstractPlus], [http://www.guidetopharmacology.org/GRAC/LigandDisplayForward?ligandId=119 (Agonist) [http://www.ncbi.nlm.nih.gov/pubmed/10193663?dopt=AbstractPlus]	[http://www.guidetopharmacology.org/GRAC/LigandDisplayForward?ligandId=3248 (Agonist) [http://www.ncbi.nlm.nih.gov/pubmed/1608964?dopt=AbstractPlus, http://www.ncbi.nlm.nih.gov/pubmed/8863519?dopt=AbstractPlus]	[http://www.guidetopharmacology.org/GRAC/LigandDisplayForward?ligandId=151 (Agonist) [http://www.ncbi.nlm.nih.gov/pubmed/15900510?dopt=AbstractPlus], [http://www.guidetopharmacology.org/GRAC/LigandDisplayForward?ligandId=261 (Agonist) [http://www.ncbi.nlm.nih.gov/pubmed/1328180?dopt=AbstractPlus] – Mouse

**Table:** 

Nomenclature	http://www.guidetopharmacology.org/GRAC/ObjectDisplayForward?objectId=6	http://www.guidetopharmacology.org/GRAC/ObjectDisplayForward?objectId=7	http://www.guidetopharmacology.org/GRAC/ObjectDisplayForward?objectId=8
HGNC, UniProt	https://www.genenames.org/data/gene‐symbol‐report/#!/hgnc_id/HGNC:5293, http://www.uniprot.org/uniprot/P28223	https://www.genenames.org/data/gene‐symbol‐report/#!/hgnc_id/HGNC:5294, http://www.uniprot.org/uniprot/P41595	https://www.genenames.org/data/gene‐symbol‐report/#!/hgnc_id/HGNC:5295, http://www.uniprot.org/uniprot/P28335
Agonists	http://www.guidetopharmacology.org/GRAC/LigandDisplayForward?ligandId=147 [http://www.ncbi.nlm.nih.gov/pubmed/2233697?dopt=AbstractPlus, http://www.ncbi.nlm.nih.gov/pubmed/9933142?dopt=AbstractPlus, http://www.ncbi.nlm.nih.gov/pubmed/8534270?dopt=AbstractPlus]	http://www.guidetopharmacology.org/GRAC/LigandDisplayForward?ligandId=134 (Partial agonist) [http://www.ncbi.nlm.nih.gov/pubmed/15322733?dopt=AbstractPlus, http://www.ncbi.nlm.nih.gov/pubmed/11104741?dopt=AbstractPlus, http://www.ncbi.nlm.nih.gov/pubmed/9459568?dopt=AbstractPlus], http://www.guidetopharmacology.org/GRAC/LigandDisplayForward?ligandId=147 [http://www.ncbi.nlm.nih.gov/pubmed/8078486?dopt=AbstractPlus, http://www.ncbi.nlm.nih.gov/pubmed/9933142?dopt=AbstractPlus, http://www.ncbi.nlm.nih.gov/pubmed/12970106?dopt=AbstractPlus]	http://www.guidetopharmacology.org/GRAC/LigandDisplayForward?ligandId=147 [http://www.ncbi.nlm.nih.gov/pubmed/10611640?dopt=AbstractPlus, http://www.ncbi.nlm.nih.gov/pubmed/9933142?dopt=AbstractPlus, http://www.ncbi.nlm.nih.gov/pubmed/12970106?dopt=AbstractPlus], http://www.guidetopharmacology.org/GRAC/LigandDisplayForward?ligandId=166 [http://www.ncbi.nlm.nih.gov/pubmed/14709324?dopt=AbstractPlus, http://www.ncbi.nlm.nih.gov/pubmed/15322733?dopt=AbstractPlus]
Selective agonists	–	http://www.guidetopharmacology.org/GRAC/LigandDisplayForward?ligandId=161 [http://www.ncbi.nlm.nih.gov/pubmed/15466450?dopt=AbstractPlus, http://www.ncbi.nlm.nih.gov/pubmed/15322733?dopt=AbstractPlus, http://www.ncbi.nlm.nih.gov/pubmed/12970106?dopt=AbstractPlus], http://www.guidetopharmacology.org/GRAC/LigandDisplayForward?ligandId=166[http://www.ncbi.nlm.nih.gov/pubmed/15322733?dopt=AbstractPlus]	http://www.guidetopharmacology.org/GRAC/LigandDisplayForward?ligandId=229 [http://www.ncbi.nlm.nih.gov/pubmed/15705738?dopt=AbstractPlus], http://www.guidetopharmacology.org/GRAC/LigandDisplayForward?ligandId=2941 [http://www.ncbi.nlm.nih.gov/pubmed/18252809?dopt=AbstractPlus]
Antagonists	http://www.guidetopharmacology.org/GRAC/LigandDisplayForward?ligandId=96 (Inverse agonist) (p*K*_i_ 9.3–10) [http://www.ncbi.nlm.nih.gov/pubmed/12176106?dopt=AbstractPlus, http://www.ncbi.nlm.nih.gov/pubmed/12629531?dopt=AbstractPlus, http://www.ncbi.nlm.nih.gov/pubmed/8935801?dopt=AbstractPlus], http://www.guidetopharmacology.org/GRAC/LigandDisplayForward?ligandId=135 (p*K*_i_ 7.7–9.6) [http://www.ncbi.nlm.nih.gov/pubmed/15322733?dopt=AbstractPlus, http://www.ncbi.nlm.nih.gov/pubmed/15107597?dopt=AbstractPlus, http://www.ncbi.nlm.nih.gov/pubmed/10611634?dopt=AbstractPlus], http://www.guidetopharmacology.org/GRAC/LigandDisplayForward?ligandId=59 (p*K*_i_ 8.8–9.5) [http://www.ncbi.nlm.nih.gov/pubmed/12176106?dopt=AbstractPlus, http://www.ncbi.nlm.nih.gov/pubmed/8935801?dopt=AbstractPlus, http://www.ncbi.nlm.nih.gov/pubmed/18308814?dopt=AbstractPlus], http://www.guidetopharmacology.org/GRAC/LigandDisplayForward?ligandId=185 (pIC_50_ 6.5–9.3) [http://www.ncbi.nlm.nih.gov/pubmed/15322733?dopt=AbstractPlus, http://www.ncbi.nlm.nih.gov/pubmed/11562430?dopt=AbstractPlus, http://www.ncbi.nlm.nih.gov/pubmed/10188965?dopt=AbstractPlus], http://www.guidetopharmacology.org/GRAC/LigandDisplayForward?ligandId=7670 (p*K*_i_ 9.1) [http://www.ncbi.nlm.nih.gov/pubmed/15686911?dopt=AbstractPlus], http://www.guidetopharmacology.org/GRAC/LigandDisplayForward?ligandId=38 (Inverse agonist) (p*K*_i_ 7.6–9) [http://www.ncbi.nlm.nih.gov/pubmed/15322733?dopt=AbstractPlus, http://www.ncbi.nlm.nih.gov/pubmed/15322733?dopt=AbstractPlus, http://www.ncbi.nlm.nih.gov/pubmed/9732398?dopt=AbstractPlus, http://www.ncbi.nlm.nih.gov/pubmed/8935801?dopt=AbstractPlus, http://www.ncbi.nlm.nih.gov/pubmed/15102927?dopt=AbstractPlus], http://www.guidetopharmacology.org/GRAC/LigandDisplayForward?ligandId=9880 (pIC_50_ 7.2) [http://www.ncbi.nlm.nih.gov/pubmed/29615471?dopt=AbstractPlus]	http://www.guidetopharmacology.org/GRAC/LigandDisplayForward?ligandId=135 (p*K*_i_ 7.9–8.8) [http://www.ncbi.nlm.nih.gov/pubmed/10455251?dopt=AbstractPlus, http://www.ncbi.nlm.nih.gov/pubmed/15322733?dopt=AbstractPlus, http://www.ncbi.nlm.nih.gov/pubmed/9459568?dopt=AbstractPlus]	http://www.guidetopharmacology.org/GRAC/LigandDisplayForward?ligandId=135 (Inverse agonist) (p*K*_i_ 8.3–9.2) [http://www.ncbi.nlm.nih.gov/pubmed/10217294?dopt=AbstractPlus, http://www.ncbi.nlm.nih.gov/pubmed/15322733?dopt=AbstractPlus, http://www.ncbi.nlm.nih.gov/pubmed/10611634?dopt=AbstractPlus], http://www.guidetopharmacology.org/GRAC/LigandDisplayForward?ligandId=134 (p*K*_i_ 8.6–9.1) [http://www.ncbi.nlm.nih.gov/pubmed/10611640?dopt=AbstractPlus], http://www.guidetopharmacology.org/GRAC/LigandDisplayForward?ligandId=59 (Inverse agonist) (p*K*_i_ 7.9–9) [http://www.ncbi.nlm.nih.gov/pubmed/10991983?dopt=AbstractPlus, http://www.ncbi.nlm.nih.gov/pubmed/12629531?dopt=AbstractPlus, http://www.ncbi.nlm.nih.gov/pubmed/18308814?dopt=AbstractPlus], http://www.guidetopharmacology.org/GRAC/LigandDisplayForward?ligandId=47 (Inverse agonist) (p*K*_i_ 8.1–8.4) [http://www.ncbi.nlm.nih.gov/pubmed/10991983?dopt=AbstractPlus, http://www.ncbi.nlm.nih.gov/pubmed/18308814?dopt=AbstractPlus], http://www.guidetopharmacology.org/GRAC/LigandDisplayForward?ligandId=205 (Inverse agonist) (p*K*_i_ 7.8–8) [http://www.ncbi.nlm.nih.gov/pubmed/10991983?dopt=AbstractPlus, http://www.ncbi.nlm.nih.gov/pubmed/10991983?dopt=AbstractPlus]
Selective antagonists	http://www.guidetopharmacology.org/GRAC/LigandDisplayForward?ligandId=9825 (p*K*_i_ 10.6) [http://www.ncbi.nlm.nih.gov/pubmed/28943244?dopt=AbstractPlus], http://www.guidetopharmacology.org/GRAC/LigandDisplayForward?ligandId=88 (p*K*_i_ 8.1–9.7) [http://www.ncbi.nlm.nih.gov/pubmed/8845011?dopt=AbstractPlus, http://www.ncbi.nlm.nih.gov/pubmed/15322733?dopt=AbstractPlus, http://www.ncbi.nlm.nih.gov/pubmed/12738034?dopt=AbstractPlus], http://www.guidetopharmacology.org/GRAC/LigandDisplayForward?ligandId=8423 (Inverse agonist) (p*K*_i_ 9.3) [http://www.ncbi.nlm.nih.gov/pubmed/17519387?dopt=AbstractPlus, http://www.ncbi.nlm.nih.gov/pubmed/15102927?dopt=AbstractPlus]	http://www.guidetopharmacology.org/GRAC/LigandDisplayForward?ligandId=8424 (p*K*_i_ 10.1) [http://www.ncbi.nlm.nih.gov/pubmed/25666387?dopt=AbstractPlus], http://www.guidetopharmacology.org/GRAC/LigandDisplayForward?ligandId=188 (p*K*_i_ 9–9.5) [http://www.ncbi.nlm.nih.gov/pubmed/10455251?dopt=AbstractPlus, http://www.ncbi.nlm.nih.gov/pubmed/15322733?dopt=AbstractPlus], http://www.guidetopharmacology.org/GRAC/LigandDisplayForward?ligandId=176 (p*K*_i_ 9) [http://www.ncbi.nlm.nih.gov/pubmed/15107597?dopt=AbstractPlus]	http://www.guidetopharmacology.org/GRAC/LigandDisplayForward?ligandId=3223 (p*K*_i_ 9) [http://www.ncbi.nlm.nih.gov/pubmed/17074317?dopt=AbstractPlus], http://www.guidetopharmacology.org/GRAC/LigandDisplayForward?ligandId=193 (p*K*_i_ 8.2–9) [http://www.ncbi.nlm.nih.gov/pubmed/9225286?dopt=AbstractPlus, http://www.ncbi.nlm.nih.gov/pubmed/15322733?dopt=AbstractPlus], http://www.guidetopharmacology.org/GRAC/LigandDisplayForward?ligandId=187 (p*K*_i_ 8.3–8.4) [http://www.ncbi.nlm.nih.gov/pubmed/9225287?dopt=AbstractPlus]
Labelled ligands	[http://www.guidetopharmacology.org/GRAC/LigandDisplayForward?ligandId=3229 (Antagonist) (p*K*_d_ 9.9) [http://www.ncbi.nlm.nih.gov/pubmed/8472747?dopt=AbstractPlus] – Rat, [http://www.guidetopharmacology.org/GRAC/LigandDisplayForward?ligandId=197 (Antagonist) (p*K*_d_ 8.6–9.7) [http://www.ncbi.nlm.nih.gov/pubmed/15322733?dopt=AbstractPlus, http://www.ncbi.nlm.nih.gov/pubmed/12738034?dopt=AbstractPlus], [http://www.guidetopharmacology.org/GRAC/LigandDisplayForward?ligandId=3249 (Antagonist) [http://www.ncbi.nlm.nih.gov/pubmed/11044889?dopt=AbstractPlus], [http://www.guidetopharmacology.org/GRAC/LigandDisplayForward?ligandId=3242 (Antagonist) [http://www.ncbi.nlm.nih.gov/pubmed/9027929?dopt=AbstractPlus]	[http://www.guidetopharmacology.org/GRAC/LigandDisplayForward?ligandId=219 (Agonist) [http://www.ncbi.nlm.nih.gov/pubmed/12738034?dopt=AbstractPlus], [http://www.guidetopharmacology.org/GRAC/LigandDisplayForward?ligandId=3248 (Agonist) [http://www.ncbi.nlm.nih.gov/pubmed/8450835?dopt=AbstractPlus] – Rat, [http://www.guidetopharmacology.org/GRAC/LigandDisplayForward?ligandId=232 (Antagonist, Inverse agonist) (p*K*_d_ 7.9) [http://www.ncbi.nlm.nih.gov/pubmed/15322733?dopt=AbstractPlus], [http://www.guidetopharmacology.org/GRAC/LigandDisplayForward?ligandId=169 (Agonist)	[http://www.guidetopharmacology.org/GRAC/LigandDisplayForward?ligandId=232 (Antagonist, Inverse agonist) (p*K*_d_ 8.7–9.3) [http://www.ncbi.nlm.nih.gov/pubmed/10217294?dopt=AbstractPlus, http://www.ncbi.nlm.nih.gov/pubmed/12738034?dopt=AbstractPlus], [http://www.guidetopharmacology.org/GRAC/LigandDisplayForward?ligandId=169 (Agonist) [http://www.ncbi.nlm.nih.gov/pubmed/10217294?dopt=AbstractPlus], [http://www.guidetopharmacology.org/GRAC/LigandDisplayForward?ligandId=219 (Agonist)

**Table:** 

Nomenclature	http://www.guidetopharmacology.org/GRAC/ObjectDisplayForward?objectId=9	http://www.guidetopharmacology.org/GRAC/ObjectDisplayForward?objectId=10	http://www.guidetopharmacology.org/GRAC/ObjectDisplayForward?objectId=648
HGNC, UniProt	https://www.genenames.org/data/gene‐symbol‐report/#!/hgnc_id/HGNC:5299, http://www.uniprot.org/uniprot/Q13639	https://www.genenames.org/data/gene‐symbol‐report/#!/hgnc_id/HGNC:5300, http://www.uniprot.org/uniprot/P47898	https://www.genenames.org/data/gene‐symbol‐report/#!/hgnc_id/HGNC:16291, –
Agonists	http://www.guidetopharmacology.org/GRAC/LigandDisplayForward?ligandId=240 (Partial agonist) [http://www.ncbi.nlm.nih.gov/pubmed/11218067?dopt=AbstractPlus, http://www.ncbi.nlm.nih.gov/pubmed/10646498?dopt=AbstractPlus, http://www.ncbi.nlm.nih.gov/pubmed/7796807?dopt=AbstractPlus, http://www.ncbi.nlm.nih.gov/pubmed/10683202?dopt=AbstractPlus, http://www.ncbi.nlm.nih.gov/pubmed/11030734?dopt=AbstractPlus, http://www.ncbi.nlm.nih.gov/pubmed/9349523?dopt=AbstractPlus]		
Selective agonists	http://www.guidetopharmacology.org/GRAC/LigandDisplayForward?ligandId=8426 [http://www.ncbi.nlm.nih.gov/pubmed/23756062?dopt=AbstractPlus], http://www.guidetopharmacology.org/GRAC/LigandDisplayForward?ligandId=235 (Partial agonist) [http://www.ncbi.nlm.nih.gov/pubmed/12801225?dopt=AbstractPlus, http://www.ncbi.nlm.nih.gov/pubmed/9603189?dopt=AbstractPlus, http://www.ncbi.nlm.nih.gov/pubmed/10683202?dopt=AbstractPlus, http://www.ncbi.nlm.nih.gov/pubmed/11030734?dopt=AbstractPlus, http://www.ncbi.nlm.nih.gov/pubmed/10821780?dopt=AbstractPlus], http://www.guidetopharmacology.org/GRAC/LigandDisplayForward?ligandId=3230 [http://www.ncbi.nlm.nih.gov/pubmed/8903510?dopt=AbstractPlus] – Rat, http://www.guidetopharmacology.org/GRAC/LigandDisplayForward?ligandId=8427 (Partial agonist) [http://www.ncbi.nlm.nih.gov/pubmed/25316608?dopt=AbstractPlus], http://www.guidetopharmacology.org/GRAC/LigandDisplayForward?ligandId=8425 [http://www.ncbi.nlm.nih.gov/pubmed/22959244?dopt=AbstractPlus, http://www.ncbi.nlm.nih.gov/pubmed/18415081?dopt=AbstractPlus], http://www.guidetopharmacology.org/GRAC/LigandDisplayForward?ligandId=234 [http://www.ncbi.nlm.nih.gov/pubmed/9351641?dopt=AbstractPlus]	–	–
Selective antagonists	http://www.guidetopharmacology.org/GRAC/LigandDisplayForward?ligandId=250 (p*K*_i_ 8.7–12.2) [http://www.ncbi.nlm.nih.gov/pubmed/9351641?dopt=AbstractPlus, http://www.ncbi.nlm.nih.gov/pubmed/15351779?dopt=AbstractPlus], http://www.guidetopharmacology.org/GRAC/LigandDisplayForward?ligandId=256 (p*K*_i_ 9.8–10.4) [http://www.ncbi.nlm.nih.gov/pubmed/10646498?dopt=AbstractPlus, http://www.ncbi.nlm.nih.gov/pubmed/10683202?dopt=AbstractPlus, http://www.ncbi.nlm.nih.gov/pubmed/11030734?dopt=AbstractPlus, http://www.ncbi.nlm.nih.gov/pubmed/9349523?dopt=AbstractPlus], http://www.guidetopharmacology.org/GRAC/LigandDisplayForward?ligandId=247 (p*K*i 9.3–10.3) [http://www.ncbi.nlm.nih.gov/pubmed/11218067?dopt=AbstractPlus, http://www.ncbi.nlm.nih.gov/pubmed/10646498?dopt=AbstractPlus, http://www.ncbi.nlm.nih.gov/pubmed/9603189?dopt=AbstractPlus, http://www.ncbi.nlm.nih.gov/pubmed/11030734?dopt=AbstractPlus, http://www.ncbi.nlm.nih.gov/pubmed/15351779?dopt=AbstractPlus, http://www.ncbi.nlm.nih.gov/pubmed/9349523?dopt=AbstractPlus]	http://www.guidetopharmacology.org/GRAC/LigandDisplayForward?ligandId=264 (p*K*_i_ 8.2) [http://www.ncbi.nlm.nih.gov/pubmed/16002289?dopt=AbstractPlus]	–
Labelled ligands	[http://www.guidetopharmacology.org/GRAC/LigandDisplayForward?ligandId=259 (Antagonist) (p*K*_d_ 9.7–10.3) [http://www.ncbi.nlm.nih.gov/pubmed/11218067?dopt=AbstractPlus, http://www.ncbi.nlm.nih.gov/pubmed/10646498?dopt=AbstractPlus, http://www.ncbi.nlm.nih.gov/pubmed/10821780?dopt=AbstractPlus, http://www.ncbi.nlm.nih.gov/pubmed/9349523?dopt=AbstractPlus], [http://www.guidetopharmacology.org/GRAC/LigandDisplayForward?ligandId=3243 (Antagonist) (p*K*_d_ 10.1) [251] – Pig, [http://www.guidetopharmacology.org/GRAC/LigandDisplayForward?ligandId=239 (Selective Antagonist) (p*K*_d_ 9.7) [http://www.ncbi.nlm.nih.gov/pubmed/9225293?dopt=AbstractPlus] – Guinea pig, [http://www.guidetopharmacology.org/GRAC/LigandDisplayForward?ligandId=3244 (Antagonist) (p*K*_d_ 8.6) [http://www.ncbi.nlm.nih.gov/pubmed/21831646?dopt=AbstractPlus]	[http://www.guidetopharmacology.org/GRAC/LigandDisplayForward?ligandId=261 (Agonist) [http://www.ncbi.nlm.nih.gov/pubmed/11343685?dopt=AbstractPlus], [http://www.guidetopharmacology.org/GRAC/LigandDisplayForward?ligandId=262 (Agonist) [http://www.ncbi.nlm.nih.gov/pubmed/11343685?dopt=AbstractPlus]	[http://www.guidetopharmacology.org/GRAC/LigandDisplayForward?ligandId=261 (Agonist) [http://www.ncbi.nlm.nih.gov/pubmed/8450829?dopt=AbstractPlus] – Mouse, [http://www.guidetopharmacology.org/GRAC/LigandDisplayForward?ligandId=262 (Agonist) [http://www.ncbi.nlm.nih.gov/pubmed/9928243?dopt=AbstractPlus] – Mouse

**Table:** 

Nomenclature	http://www.guidetopharmacology.org/GRAC/ObjectDisplayForward?objectId=11	http://www.guidetopharmacology.org/GRAC/ObjectDisplayForward?objectId=12
HGNC, UniProt	https://www.genenames.org/data/gene‐symbol‐report/#!/hgnc_id/HGNC:5301, http://www.uniprot.org/uniprot/P50406	https://www.genenames.org/data/gene‐symbol‐report/#!/hgnc_id/HGNC:5302, http://www.uniprot.org/uniprot/P34969
Selective agonists	http://www.guidetopharmacology.org/GRAC/LigandDisplayForward?ligandId=3240 [http://www.ncbi.nlm.nih.gov/pubmed/17625499?dopt=AbstractPlus], http://www.guidetopharmacology.org/GRAC/LigandDisplayForward?ligandId=3217 (Partial agonist) [http://www.ncbi.nlm.nih.gov/pubmed/15771424?dopt=AbstractPlus], http://www.guidetopharmacology.org/GRAC/LigandDisplayForward?ligandId=8429 [http://www.ncbi.nlm.nih.gov/pubmed/19523834?dopt=AbstractPlus], http://www.guidetopharmacology.org/GRAC/LigandDisplayForward?ligandId=8428 [http://www.ncbi.nlm.nih.gov/pubmed/16055331?dopt=AbstractPlus]	http://www.guidetopharmacology.org/GRAC/LigandDisplayForward?ligandId=8434 [http://www.ncbi.nlm.nih.gov/pubmed/17649988?dopt=AbstractPlus], http://www.guidetopharmacology.org/GRAC/LigandDisplayForward?ligandId=8435], http://www.guidetopharmacology.org/GRAC/LigandDisplayForward?ligandId=8436 [http://www.ncbi.nlm.nih.gov/pubmed/18800769?dopt=AbstractPlus] – Rat, http://www.guidetopharmacology.org/GRAC/LigandDisplayForward?ligandId=8433 [http://www.ncbi.nlm.nih.gov/pubmed/23541835?dopt=AbstractPlus], http://www.guidetopharmacology.org/GRAC/LigandDisplayForward?ligandId=3929 [http://www.ncbi.nlm.nih.gov/pubmed/19118950?dopt=AbstractPlus]
Antagonists	–	http://www.guidetopharmacology.org/GRAC/LigandDisplayForward?ligandId=7461 (p*K*_i_ 9.3) [http://www.ncbi.nlm.nih.gov/pubmed/20404009?dopt=AbstractPlus], http://www.guidetopharmacology.org/GRAC/LigandDisplayForward?ligandId=90 (p*K*_i_ 9.3) [http://www.ncbi.nlm.nih.gov/pubmed/7908055?dopt=AbstractPlus] – Rat, http://www.guidetopharmacology.org/GRAC/LigandDisplayForward?ligandId=7351 (p*K*_i_ 6.3) [http://www.ncbi.nlm.nih.gov/pubmed/21486038?dopt=AbstractPlus]
Selective antagonists	http://www.guidetopharmacology.org/GRAC/LigandDisplayForward?ligandId=3241 (p*K*_i_ 9) [http://www.ncbi.nlm.nih.gov/pubmed/17069795?dopt=AbstractPlus], http://www.guidetopharmacology.org/GRAC/LigandDisplayForward?ligandId=276 (p*K*_i_ 8.9) [http://www.ncbi.nlm.nih.gov/pubmed/9925723?dopt=AbstractPlus], http://www.guidetopharmacology.org/GRAC/LigandDisplayForward?ligandId=7356 (p*K*_i_ 8.9) [407], http://www.guidetopharmacology.org/GRAC/LigandDisplayForward?ligandId=3235 (p*K*_i_ 8.5) [http://www.ncbi.nlm.nih.gov/pubmed/11140733?dopt=AbstractPlus], http://www.guidetopharmacology.org/GRAC/LigandDisplayForward?ligandId=275 (p*K*_i_ 7.9–8.4) [http://www.ncbi.nlm.nih.gov/pubmed/9730917?dopt=AbstractPlus, http://www.ncbi.nlm.nih.gov/pubmed/9647481?dopt=AbstractPlus]	http://www.guidetopharmacology.org/GRAC/LigandDisplayForward?ligandId=3233 (p*K*_i_ 8.6–8.9) [http://www.ncbi.nlm.nih.gov/pubmed/10807680?dopt=AbstractPlus], http://www.guidetopharmacology.org/GRAC/LigandDisplayForward?ligandId=3237 (p*K*_i_ 8.7) [http://www.ncbi.nlm.nih.gov/pubmed/12392747?dopt=AbstractPlus], http://www.guidetopharmacology.org/GRAC/LigandDisplayForward?ligandId=8431 (p*K*_i_ 8.7) [http://www.ncbi.nlm.nih.gov/pubmed/12825922?dopt=AbstractPlus, http://www.ncbi.nlm.nih.gov/pubmed/10052959?dopt=AbstractPlus], http://www.guidetopharmacology.org/GRAC/LigandDisplayForward?ligandId=8432 (p*K*_i_ 8.2) [http://www.ncbi.nlm.nih.gov/pubmed/22570363?dopt=AbstractPlus], http://www.guidetopharmacology.org/GRAC/LigandDisplayForward?ligandId=281 (Inverse agonist) (p*K*_i_ 7.5) [http://www.ncbi.nlm.nih.gov/pubmed/9720804?dopt=AbstractPlus]
Labelled ligands	[http://www.guidetopharmacology.org/GRAC/LigandDisplayForward?ligandId=8430 (Antagonist) (p*K*_i_ 9.8) [http://www.ncbi.nlm.nih.gov/pubmed/22223878?dopt=AbstractPlus], [http://www.guidetopharmacology.org/GRAC/LigandDisplayForward?ligandId=3245 (Selective Antagonist) (p*K*_d_ 9) [http://www.ncbi.nlm.nih.gov/pubmed/17069795?dopt=AbstractPlus], [http://www.guidetopharmacology.org/GRAC/LigandDisplayForward?ligandId=219 (Agonist) [http://www.ncbi.nlm.nih.gov/pubmed/9284367?dopt=AbstractPlus], [http://www.guidetopharmacology.org/GRAC/LigandDisplayForward?ligandId=3246 (Antagonist) (p*K*_d_ 8.3) [http://www.ncbi.nlm.nih.gov/pubmed/9730917?dopt=AbstractPlus], [http://www.guidetopharmacology.org/GRAC/LigandDisplayForward?ligandId=262 (Agonist)	[http://www.guidetopharmacology.org/GRAC/LigandDisplayForward?ligandId=262 (Agonist) [http://www.ncbi.nlm.nih.gov/pubmed/10807680?dopt=AbstractPlus], [http://www.guidetopharmacology.org/GRAC/LigandDisplayForward?ligandId=3248 (Agonist) [http://www.ncbi.nlm.nih.gov/pubmed/8226867?dopt=AbstractPlus, http://www.ncbi.nlm.nih.gov/pubmed/9303561?dopt=AbstractPlus], [http://www.guidetopharmacology.org/GRAC/LigandDisplayForward?ligandId=3247 (Selective Antagonist) (p*K*_d_ 8.9) [http://www.ncbi.nlm.nih.gov/pubmed/10807680?dopt=AbstractPlus], [http://www.guidetopharmacology.org/GRAC/LigandDisplayForward?ligandId=219 (Agonist) [http://www.ncbi.nlm.nih.gov/pubmed/9303561?dopt=AbstractPlus]

### Comments

Tabulated *pK*_i_ and *K*_D_ values refer to binding to human 5‐HT receptors unless indicated otherwise. The nomenclature of 5‐HT_1B_/5‐HT_1D_ receptors has been revised [http://www.ncbi.nlm.nih.gov/pubmed/8936345?dopt=AbstractPlus]. Only the non‐rodent form of the receptor was previously called 5‐HT_1D_: the human 5‐HT_1B_ receptor (tabulated) displays a different pharmacology to the rodent forms of the receptor due to Thr335 of the human sequence being replaced by Asn in rodent receptors [http://www.ncbi.nlm.nih.gov/pubmed/18571247?dopt=AbstractPlus]. Wang *et al.* (2013) report X‐ray structures which reveal the binding modality of http://www.guidetopharmacology.org/GRAC/LigandDisplayForward?ligandId=149 and http://www.guidetopharmacology.org/GRAC/LigandDisplayForward?ligandId=121 (DHE) to the 5‐HT_1B_ receptor in comparison with the structure of the 5HT_2B_ receptor [http://www.ncbi.nlm.nih.gov/pubmed/23519210?dopt=AbstractPlus]; some of these drugs adopt rather different conformations depending on the target receptor [http://www.ncbi.nlm.nih.gov/pubmed/29398112?dopt=AbstractPlus]. Various 5‐HT receptors have multiple partners in addition to G proteins, which may affect function and pharmacology [http://www.ncbi.nlm.nih.gov/pubmed/21777185?dopt=AbstractPlus]. http://www.guidetopharmacology.org/GRAC/LigandDisplayForward?ligandId=3226 is a selective antagonist of the rodent 5‐HT_1B_ receptor. http://www.guidetopharmacology.org/GRAC/LigandDisplayForward?ligandId=5434 (LSD) and http://www.guidetopharmacology.org/GRAC/LigandDisplayForward?ligandId=88 bind with high affinity to dopamine D4 and histamine H_1_ receptors respectively, and http://www.guidetopharmacology.org/GRAC/LigandDisplayForward?ligandId=88 is a potent α1 adrenoceptor antagonist, in addition to blocking 5‐HT_2A_ receptors. http://www.guidetopharmacology.org/GRAC/LigandDisplayForward?ligandId=139 (LSD) and http://www.guidetopharmacology.org/GRAC/LigandDisplayForward?ligandId=149 show a strong preference for arrestin recruitment over G protein coupling at the 5‐HT_2B_ receptor, with no such preference evident at 5‐HT_1B_ receptors, and they also antagonise 5‐HT_7A_ receptors [http://www.ncbi.nlm.nih.gov/pubmed/23519215?dopt=AbstractPlus]. DHE (http://www.guidetopharmacology.org/GRAC/LigandDisplayForward?ligandId=282), http://www.guidetopharmacology.org/GRAC/LigandDisplayForward?ligandId=48 and http://www.guidetopharmacology.org/GRAC/LigandDisplayForward?ligandId=37 also show significant preference for arrestin recruitment over G protein coupling at 5‐HT_2B_ receptors [http://www.ncbi.nlm.nih.gov/pubmed/23519215?dopt=AbstractPlus]. The 5‐HT_2B_ (and other 5‐HT) receptors interact with immunocompetent cells [http://www.ncbi.nlm.nih.gov/pubmed/28265714?dopt=AbstractPlus]. The serotonin antagonist http://www.guidetopharmacology.org/GRAC/LigandDisplayForward?ligandId=206 was key to the discovery of the 5HT_2C_ receptor [http://www.ncbi.nlm.nih.gov/pubmed/6519175?dopt=AbstractPlus], initially known as 5‐HT_1C_ [http://www.ncbi.nlm.nih.gov/pubmed/28806488?dopt=AbstractPlus]. The human 5‐HT_5A_ receptor may couple to several signal transduction pathways when stably expressed in C6 glioma cells [http://www.ncbi.nlm.nih.gov/pubmed/12558985?dopt=AbstractPlus] and rodent prefrontal cortex (layer V pyramidal neurons) [http://www.ncbi.nlm.nih.gov/pubmed/22539842?dopt=AbstractPlus]. The human orthologue of the mouse 5‐ht_5b_ receptor is non‐functional (stop codons); the 5‐ht_1e_ receptor has not been cloned from mouse, or rat, impeding definition of its function [http://www.ncbi.nlm.nih.gov/pubmed/18571247?dopt=AbstractPlus]. In addition to accepted receptors, an ’orphan’ receptor, unofficially termed5‐HT_1P_, has been described [http://www.ncbi.nlm.nih.gov/pubmed/10429737?dopt=AbstractPlus].

### Further reading on 5‐Hydroxytryptamine receptors

Bockaert J *et al*. (2011) 5‐HT(4) receptors, a place in the sun: act two. *Curr Opin Pharmacol* **11**: 87‐93 [https://www.ncbi.nlm.nih.gov/pubmed/21342787?dopt=AbstractPlus]

Hayes DJ *et al*. (2011) 5‐HT receptors and reward‐related behaviour: a review. *Neurosci Biobehav Rev* **35**: 1419‐49 [https://www.ncbi.nlm.nih.gov/pubmed/21402098?dopt=AbstractPlus]

Hoyer D *et al*. (1994) International Union of Pharmacology classification of receptors for 5hydroxytryptamine (Serotonin). *Pharmacol. Rev.* **46**: 157‐203 [https://www.ncbi.nlm.nih.gov/pubmed/7938165?dopt=AbstractPlus]

Leopoldo M *et al*. (2011)Serotonin 5‐HT_7_ receptor agents: Structure‐activity relationships and potential therapeutic applications in central nervous system disorders. *Pharmacol. Ther.* **129**: 120‐48 [https://www.ncbi.nlm.nih.gov/pubmed/20923682?dopt=AbstractPlus]

Meltzer HY *et al*. (2011) The role of serotonin receptors in the action of atypical antipsychotic drugs. *Curr Opin Pharmacol* **11**: 59‐67 [https://www.ncbi.nlm.nih.gov/pubmed/21420906?dopt=AbstractPlus]

Roberts AJ *et al*. (2012) The 5‐HT(7) receptor in learning and memory. *Hippocampus* **22**: 762‐71 [https://www.ncbi.nlm.nih.gov/pubmed/21484935?dopt=AbstractPlus]

## http://www.guidetopharmacology.org/GRAC/FamilyDisplayForward?familyId=2

### Overview

Muscarinic acetylcholine receptors (**nomenclature as agreed by the NC‐IUPHAR Subcommittee on Muscarinic Acetylcholine Receptors** [http://www.ncbi.nlm.nih.gov/pubmed/9647869?dopt=AbstractPlus]) are GPCRs of the Class A, rhodopsin‐like family where the endogenous agonist is http://www.guidetopharmacology.org/GRAC/LigandDisplayForward?ligandId=294. In addition to the agents listed in the table, http://www.guidetopharmacology.org/GRAC/LigandDisplayForward?ligandId=289, its structural analogues http://www.guidetopharmacology.org/GRAC/LigandDisplayForward?ligandId=334 and http://www.guidetopharmacology.org/GRAC/LigandDisplayForward?ligandId=3271, http://www.guidetopharmacology.org/GRAC/LigandDisplayForward?ligandId=333, http://www.guidetopharmacology.org/GRAC/LigandDisplayForward?ligandId=3258 and http://www.guidetopharmacology.org/GRAC/LigandDisplayForward?ligandId=5435 have been described as functionally selective agonists of the M_1_ receptor subtype *via* binding in a mode distinct from that utilized by non‐selective agonists [http://www.ncbi.nlm.nih.gov/pubmed/20413650?dopt=AbstractPlus, http://www.ncbi.nlm.nih.gov/pubmed/18842902?dopt=AbstractPlus, http://www.ncbi.nlm.nih.gov/pubmed/18454168?dopt=AbstractPlus, http://www.ncbi.nlm.nih.gov/pubmed/16207821?dopt=AbstractPlus, http://www.ncbi.nlm.nih.gov/pubmed/17525129?dopt=AbstractPlus, http://www.ncbi.nlm.nih.gov/pubmed/20684563?dopt=AbstractPlus, http://www.ncbi.nlm.nih.gov/pubmed/16959945?dopt=AbstractPlus, http://www.ncbi.nlm.nih.gov/pubmed/12021390?dopt=AbstractPlus, http://www.ncbi.nlm.nih.gov/pubmed/14595031?dopt=AbstractPlus]. There are two pharmacologically characterised allosteric sites on muscarinic receptors, one defined by it binding http://www.guidetopharmacology.org/GRAC/LigandDisplayForward?ligandId=356, http://www.guidetopharmacology.org/GRAC/LigandDisplayForward?ligandId=347 and http://www.guidetopharmacology.org/GRAC/LigandDisplayForward?ligandId=342, and the other defined by the binding of http://www.guidetopharmacology.org/GRAC/LigandDisplayForward?ligandId=337, http://www.guidetopharmacology.org/GRAC/LigandDisplayForward?ligandId=340, http://www.guidetopharmacology.org/GRAC/LigandDisplayForward?ligandId=339 and http://www.guidetopharmacology.org/GRAC/LigandDisplayForward?ligandId=346 [http://www.ncbi.nlm.nih.gov/pubmed/10860942?dopt=AbstractPlus, http://www.ncbi.nlm.nih.gov/pubmed/12435818?dopt=AbstractPlus].

**Table:** 

Nomenclature	http://www.guidetopharmacology.org/GRAC/ObjectDisplayForward?objectId=13	http://www.guidetopharmacology.org/GRAC/ObjectDisplayForward?objectId=14
HGNC, UniProt	https://www.genenames.org/data/gene‐symbol‐report/#!/hgnc_id/HGNC:1950, http://www.uniprot.org/uniprot/P11229	https://www.genenames.org/data/gene‐symbol‐report/#!/hgnc_id/HGNC:1951, http://www.uniprot.org/uniprot/P08172
Agonists	http://www.guidetopharmacology.org/GRAC/LigandDisplayForward?ligandId=298 [http://www.ncbi.nlm.nih.gov/pubmed/9614217?dopt=AbstractPlus, http://www.ncbi.nlm.nih.gov/pubmed/9224827?dopt=AbstractPlus, http://www.ncbi.nlm.nih.gov/pubmed/10323594?dopt=AbstractPlus], http://www.guidetopharmacology.org/GRAC/LigandDisplayForward?ligandId=305 (Partial agonist) [http://www.ncbi.nlm.nih.gov/pubmed/9224827?dopt=AbstractPlus], http://www.guidetopharmacology.org/GRAC/LigandDisplayForward?ligandId=297[http://www.ncbi.nlm.nih.gov/pubmed/9224827?dopt=AbstractPlus]	http://www.guidetopharmacology.org/GRAC/LigandDisplayForward?ligandId=298 [http://www.ncbi.nlm.nih.gov/pubmed/12235229?dopt=AbstractPlus, http://www.ncbi.nlm.nih.gov/pubmed/9224827?dopt=AbstractPlus, http://www.ncbi.nlm.nih.gov/pubmed/9454790?dopt=AbstractPlus], http://www.guidetopharmacology.org/GRAC/LigandDisplayForward?ligandId=297[http://www.ncbi.nlm.nih.gov/pubmed/9224827?dopt=AbstractPlus]
Selective agonists	http://www.guidetopharmacology.org/GRAC/LigandDisplayForward?ligandId=10259 [http://www.ncbi.nlm.nih.gov/pubmed/30276808?dopt=AbstractPlus]	–
Antagonists	http://www.guidetopharmacology.org/GRAC/LigandDisplayForward?ligandId=7459 (pIC_50_ 9.9) [http://www.ncbi.nlm.nih.gov/pubmed/21036043?dopt=AbstractPlus], http://www.guidetopharmacology.org/GRAC/LigandDisplayForward?ligandId=8586 (p*K*_i_ 9.7) [http://www.ncbi.nlm.nih.gov/pubmed/20590605?dopt=AbstractPlus], http://www.guidetopharmacology.org/GRAC/LigandDisplayForward?ligandId=320 (p*K*_i_ 8.5–9.6) [http://www.ncbi.nlm.nih.gov/pubmed/9614217?dopt=AbstractPlus, http://www.ncbi.nlm.nih.gov/pubmed/16439611?dopt=AbstractPlus, http://www.ncbi.nlm.nih.gov/pubmed/11303071?dopt=AbstractPlus, http://www.ncbi.nlm.nih.gov/pubmed/12049493?dopt=AbstractPlus, http://www.ncbi.nlm.nih.gov/pubmed/3443095?dopt=AbstractPlus, http://www.ncbi.nlm.nih.gov/pubmed/9029489?dopt=AbstractPlus], http://www.guidetopharmacology.org/GRAC/LigandDisplayForward?ligandId=367 (p*K*_i_ 9.6) [http://www.ncbi.nlm.nih.gov/pubmed/8441333?dopt=AbstractPlus], http://www.guidetopharmacology.org/GRAC/LigandDisplayForward?ligandId=307 (p*K*_i_ 9.2) [http://www.ncbi.nlm.nih.gov/pubmed/1994002?dopt=AbstractPlus]	http://www.guidetopharmacology.org/GRAC/LigandDisplayForward?ligandId=367 (p*K*_i_ 9.9) [http://www.ncbi.nlm.nih.gov/pubmed/8441333?dopt=AbstractPlus], http://www.guidetopharmacology.org/GRAC/LigandDisplayForward?ligandId=7459 (pIC_50_ 9.3) [http://www.ncbi.nlm.nih.gov/pubmed/21036043?dopt=AbstractPlus], http://www.guidetopharmacology.org/GRAC/LigandDisplayForward?ligandId=320 (p*K*_i_ 7.8–9.2) [http://www.ncbi.nlm.nih.gov/pubmed/2704370?dopt=AbstractPlus, http://www.ncbi.nlm.nih.gov/pubmed/12235229?dopt=AbstractPlus, http://www.ncbi.nlm.nih.gov/pubmed/11303071?dopt=AbstractPlus, http://www.ncbi.nlm.nih.gov/pubmed/12049493?dopt=AbstractPlus, http://www.ncbi.nlm.nih.gov/pubmed/9454790?dopt=AbstractPlus, http://www.ncbi.nlm.nih.gov/pubmed/16188951?dopt=AbstractPlus, http://www.ncbi.nlm.nih.gov/pubmed/3443095?dopt=AbstractPlus], http://www.guidetopharmacology.org/GRAC/LigandDisplayForward?ligandId=8586 (p*K*_i_ 8.6) [http://www.ncbi.nlm.nih.gov/pubmed/20590605?dopt=AbstractPlus], http://www.guidetopharmacology.org/GRAC/LigandDisplayForward?ligandId=360 (Inverse agonist) (p*K*_i_ 8.4–8.6) [http://www.ncbi.nlm.nih.gov/pubmed/9671109?dopt=AbstractPlus, http://www.ncbi.nlm.nih.gov/pubmed/20590605?dopt=AbstractPlus]
Selective antagonists	http://www.guidetopharmacology.org/GRAC/LigandDisplayForward?ligandId=7128 (p*K*_d_ 9.3) [http://www.ncbi.nlm.nih.gov/pubmed/1346637?dopt=AbstractPlus], http://www.guidetopharmacology.org/GRAC/LigandDisplayForward?ligandId=3274 (p*K*_i_ 7.8) [http://www.ncbi.nlm.nih.gov/pubmed/19407080?dopt=AbstractPlus], http://www.guidetopharmacology.org/GRAC/LigandDisplayForward?ligandId=3276 (p*K*_i_ 7.3–7.6) [http://www.ncbi.nlm.nih.gov/pubmed/10323493?dopt=AbstractPlus, http://www.ncbi.nlm.nih.gov/pubmed/8813552?dopt=AbstractPlus] – Rat	http://www.guidetopharmacology.org/GRAC/LigandDisplayForward?ligandId=361 (p*K*_i_ 9.6) [http://www.ncbi.nlm.nih.gov/pubmed/7805774?dopt=AbstractPlus]
Allosteric modulators	http://www.guidetopharmacology.org/GRAC/LigandDisplayForward?ligandId=314 (Negative) (p*K*_i_ 11–11.1) [http://www.ncbi.nlm.nih.gov/pubmed/10799315?dopt=AbstractPlus], http://www.guidetopharmacology.org/GRAC/LigandDisplayForward?ligandId=8549 (Positive) (p*K*B 6.6) [http://www.ncbi.nlm.nih.gov/pubmed/25326383?dopt=AbstractPlus], http://www.guidetopharmacology.org/GRAC/LigandDisplayForward?ligandId=337 (Positive) (p*K*_d_ 6.4) [http://www.ncbi.nlm.nih.gov/pubmed/10860942?dopt=AbstractPlus], http://www.guidetopharmacology.org/GRAC/LigandDisplayForward?ligandId=342 (Positive) (p*K*_d_ 4.5–5.8) [http://www.ncbi.nlm.nih.gov/pubmed/9224827?dopt=AbstractPlus, http://www.ncbi.nlm.nih.gov/pubmed/9495826?dopt=AbstractPlus], http://www.guidetopharmacology.org/GRAC/LigandDisplayForward?ligandId=5436 (Positive) (p*K*_B_ 4–4.8) [http://www.ncbi.nlm.nih.gov/pubmed/25326383?dopt=AbstractPlus, http://www.ncbi.nlm.nih.gov/pubmed/24443568?dopt=AbstractPlus, http://www.ncbi.nlm.nih.gov/pubmed/22086918?dopt=AbstractPlus, http://www.ncbi.nlm.nih.gov/pubmed/19717450?dopt=AbstractPlus], http://www.guidetopharmacology.org/GRAC/LigandDisplayForward?ligandId=3261 (Positive) [http://www.ncbi.nlm.nih.gov/pubmed/19047481?dopt=AbstractPlus], http://www.guidetopharmacology.org/GRAC/LigandDisplayForward?ligandId=3259 (Positive) [http://www.ncbi.nlm.nih.gov/pubmed/19047481?dopt=AbstractPlus]	http://www.guidetopharmacology.org/GRAC/LigandDisplayForward?ligandId=362 (Negative) (p*K*_d_ 6–7.5) [http://www.ncbi.nlm.nih.gov/pubmed/15163212?dopt=AbstractPlus, http://www.ncbi.nlm.nih.gov/pubmed/12815174?dopt=AbstractPlus], http://www.guidetopharmacology.org/GRAC/LigandDisplayForward?ligandId=7634 (Negative) (p*K*_d_ 7.1) [http://www.ncbi.nlm.nih.gov/pubmed/9890565?dopt=AbstractPlus], http://www.guidetopharmacology.org/GRAC/LigandDisplayForward?ligandId=341 (Negative) (p*K*_d_ 6.1–6.9) [http://www.ncbi.nlm.nih.gov/pubmed/9224827?dopt=AbstractPlus], http://www.guidetopharmacology.org/GRAC/LigandDisplayForward?ligandId=356 (Negative) (p*K*_d_ 5.9–6.3) [http://www.ncbi.nlm.nih.gov/pubmed/990587?dopt=AbstractPlus, http://www.ncbi.nlm.nih.gov/pubmed/7651370?dopt=AbstractPlus], http://www.guidetopharmacology.org/GRAC/LigandDisplayForward?ligandId=6938 (Positive) (p*K*_d_ 5.7) [http://www.ncbi.nlm.nih.gov/pubmed/24807965?dopt=AbstractPlus, http://www.ncbi.nlm.nih.gov/pubmed/24256733?dopt=AbstractPlus], http://www.guidetopharmacology.org/GRAC/LigandDisplayForward?ligandId=3262 (Positive) (p*K*_d_ 4.4) [http://www.ncbi.nlm.nih.gov/pubmed/21989256?dopt=AbstractPlus]
Labelled ligands	[http://www.guidetopharmacology.org/GRAC/LigandDisplayForward?ligandId=318 (Antagonist) (p*K*_d_ 10.6–10.8) [http://www.ncbi.nlm.nih.gov/pubmed/11578621?dopt=AbstractPlus, http://www.ncbi.nlm.nih.gov/pubmed/3443095?dopt=AbstractPlus], http://www.guidetopharmacology.org/GRAC/LigandDisplayForward?ligandId=8598 (Antagonist) (p*K*_d_ 10.5) [http://www.ncbi.nlm.nih.gov/pubmed/20133736?dopt=AbstractPlus], [http://www.guidetopharmacology.org/GRAC/LigandDisplayForward?ligandId=317 (Antagonist) (p*K*_d_ 9.4–10.3) [http://www.ncbi.nlm.nih.gov/pubmed/9846649?dopt=AbstractPlus, http://www.ncbi.nlm.nih.gov/pubmed/9614217?dopt=AbstractPlus, http://www.ncbi.nlm.nih.gov/pubmed/11303071?dopt=AbstractPlus, http://www.ncbi.nlm.nih.gov/pubmed/9224827?dopt=AbstractPlus, http://www.ncbi.nlm.nih.gov/pubmed/16675658?dopt=AbstractPlus, http://www.ncbi.nlm.nih.gov/pubmed/7925952?dopt=AbstractPlus, http://www.ncbi.nlm.nih.gov/pubmed/16369696?dopt=AbstractPlus, http://www.ncbi.nlm.nih.gov/pubmed/7651370?dopt=AbstractPlus], [http://www.guidetopharmacology.org/GRAC/LigandDisplayForward?ligandId=8594 (Antagonist) (p*K*_i_ 9.4) [http://www.ncbi.nlm.nih.gov/pubmed/2704371?dopt=AbstractPlus] – Rat, http://www.guidetopharmacology.org/GRAC/LigandDisplayForward?ligandId=8600 (Antagonist) (p*K*_d_ 9.3) [http://www.ncbi.nlm.nih.gov/pubmed/20133736?dopt=AbstractPlus], [http://www.guidetopharmacology.org/GRAC/LigandDisplayForward?ligandId=3272 (Antagonist) (p*K*_d_ 7.9) [http://www.ncbi.nlm.nih.gov/pubmed/6546354?dopt=AbstractPlus]	[http://www.guidetopharmacology.org/GRAC/LigandDisplayForward?ligandId=318 (Antagonist) (p*K*_d_ 10.1–10.6) [http://www.ncbi.nlm.nih.gov/pubmed/3443095?dopt=AbstractPlus], http://www.guidetopharmacology.org/GRAC/LigandDisplayForward?ligandId=8598 (Antagonist) (p*K*_i_ 10.4) [http://www.ncbi.nlm.nih.gov/pubmed/23357106?dopt=AbstractPlus], [http://www.guidetopharmacology.org/GRAC/LigandDisplayForward?ligandId=8592 (Antagonist) (p*K*_d_ 10.3) [http://www.ncbi.nlm.nih.gov/pubmed/23435542?dopt=AbstractPlus], [http://www.guidetopharmacology.org/GRAC/LigandDisplayForward?ligandId=317 (Antagonist) (p*K*_d_ 9.3–9.9) [http://www.ncbi.nlm.nih.gov/pubmed/9846649?dopt=AbstractPlus, http://www.ncbi.nlm.nih.gov/pubmed/12235229?dopt=AbstractPlus, http://www.ncbi.nlm.nih.gov/pubmed/11303071?dopt=AbstractPlus, http://www.ncbi.nlm.nih.gov/pubmed/9224827?dopt=AbstractPlus, http://www.ncbi.nlm.nih.gov/pubmed/16675658?dopt=AbstractPlus, http://www.ncbi.nlm.nih.gov/pubmed/7925952?dopt=AbstractPlus, http://www.ncbi.nlm.nih.gov/pubmed/16369696?dopt=AbstractPlus, http://www.ncbi.nlm.nih.gov/pubmed/7651370?dopt=AbstractPlus, http://www.ncbi.nlm.nih.gov/pubmed/7687290?dopt=AbstractPlus], http://www.guidetopharmacology.org/GRAC/LigandDisplayForward?ligandId=8600 (Antagonist) (p*K*_i_ 8.8) [http://www.ncbi.nlm.nih.gov/pubmed/23357106?dopt=AbstractPlus], [http://www.guidetopharmacology.org/GRAC/LigandDisplayForward?ligandId=8593 (Agonist) [http://www.ncbi.nlm.nih.gov/pubmed/14722259?dopt=AbstractPlus], [http://www.guidetopharmacology.org/GRAC/LigandDisplayForward?ligandId=8595 (Agonist) [http://www.ncbi.nlm.nih.gov/pubmed/6478115?dopt=AbstractPlus], [http://www.guidetopharmacology.org/GRAC/LigandDisplayForward?ligandId=364 (Allosteric modulator, Positive) (p*K*_d_ 8.5) [http://www.ncbi.nlm.nih.gov/pubmed/12815174?dopt=AbstractPlus], [http://www.guidetopharmacology.org/GRAC/LigandDisplayForward?ligandId=3266 (Agonist) [http://www.ncbi.nlm.nih.gov/pubmed/12668051?dopt=AbstractPlus] – Mouse

**Table:** 

Nomenclature	http://www.guidetopharmacology.org/GRAC/ObjectDisplayForward?objectId=15	http://www.guidetopharmacology.org/GRAC/ObjectDisplayForward?objectId=16	http://www.guidetopharmacology.org/GRAC/ObjectDisplayForward?objectId=17
HGNC, UniProt	https://www.genenames.org/data/gene‐symbol‐report/#!/hgnc_id/HGNC:1952, http://www.uniprot.org/uniprot/P20309	https://www.genenames.org/data/gene‐symbol‐report/#!/hgnc_id/HGNC:1953, http://www.uniprot.org/uniprot/P08173	https://www.genenames.org/data/gene‐symbol‐report/#!/hgnc_id/HGNC:1954, http://www.uniprot.org/uniprot/P08912
Agonists	http://www.guidetopharmacology.org/GRAC/LigandDisplayForward?ligandId=305 (Partial agonist) [http://www.ncbi.nlm.nih.gov/pubmed/9224827?dopt=AbstractPlus], http://www.guidetopharmacology.org/GRAC/LigandDisplayForward?ligandId=298 [http://www.ncbi.nlm.nih.gov/pubmed/12235229?dopt=AbstractPlus, http://www.ncbi.nlm.nih.gov/pubmed/12235229?dopt=AbstractPlus, http://www.ncbi.nlm.nih.gov/pubmed/10323594?dopt=AbstractPlus], http://www.guidetopharmacology.org/GRAC/LigandDisplayForward?ligandId=297]	http://www.guidetopharmacology.org/GRAC/LigandDisplayForward?ligandId=305 (Partial agonist) [http://www.ncbi.nlm.nih.gov/pubmed/9224827?dopt=AbstractPlus], http://www.guidetopharmacology.org/GRAC/LigandDisplayForward?ligandId=298[http://www.ncbi.nlm.nih.gov/pubmed/9224827?dopt=AbstractPlus, http://www.ncbi.nlm.nih.gov/pubmed/10323594?dopt=AbstractPlus], http://www.guidetopharmacology.org/GRAC/LigandDisplayForward?ligandId=297[http://www.ncbi.nlm.nih.gov/pubmed/9224827?dopt=AbstractPlus]	http://www.guidetopharmacology.org/GRAC/LigandDisplayForward?ligandId=305 (Partial agonist) [http://www.ncbi.nlm.nih.gov/pubmed/16002459?dopt=AbstractPlus], http://www.guidetopharmacology.org/GRAC/LigandDisplayForward?ligandId=298 [http://www.ncbi.nlm.nih.gov/pubmed/10323594?dopt=AbstractPlus]
Antagonists	http://www.guidetopharmacology.org/GRAC/LigandDisplayForward?ligandId=367 (p*K*_i_ 9.5–11.1) [http://www.ncbi.nlm.nih.gov/pubmed/8441333?dopt=AbstractPlus, http://www.ncbi.nlm.nih.gov/pubmed/16847442?dopt=AbstractPlus], http://www.guidetopharmacology.org/GRAC/LigandDisplayForward?ligandId=8586 (p*K*i 9.9) [http://www.ncbi.nlm.nih.gov/pubmed/20590605?dopt=AbstractPlus], http://www.guidetopharmacology.org/GRAC/LigandDisplayForward?ligandId=320 (p*K*_i_ 8.9–9.8) [http://www.ncbi.nlm.nih.gov/pubmed/2704370?dopt=AbstractPlus, http://www.ncbi.nlm.nih.gov/pubmed/11303071?dopt=AbstractPlus, http://www.ncbi.nlm.nih.gov/pubmed/12049493?dopt=AbstractPlus, http://www.ncbi.nlm.nih.gov/pubmed/3443095?dopt=AbstractPlus, http://www.ncbi.nlm.nih.gov/pubmed/9029489?dopt=AbstractPlus], http://www.guidetopharmacology.org/GRAC/LigandDisplayForward?ligandId=325 (p*K*_i_ 9.3–9.8) [http://www.ncbi.nlm.nih.gov/pubmed/16847442?dopt=AbstractPlus], http://www.guidetopharmacology.org/GRAC/LigandDisplayForward?ligandId=7449 (pIC_50_ 9.8) [http://www.ncbi.nlm.nih.gov/pubmed/19653626?dopt=AbstractPlus]	http://www.guidetopharmacology.org/GRAC/LigandDisplayForward?ligandId=7459 (pIC_50_ 9.8) [http://www.ncbi.nlm.nih.gov/pubmed/21036043?dopt=AbstractPlus], http://www.guidetopharmacology.org/GRAC/LigandDisplayForward?ligandId=8586 (p*K*_i_ 9.5) [http://www.ncbi.nlm.nih.gov/pubmed/20590605?dopt=AbstractPlus], http://www.guidetopharmacology.org/GRAC/LigandDisplayForward?ligandId=307 (p*K*_i_ 8.9) [http://www.ncbi.nlm.nih.gov/pubmed/1994002?dopt=AbstractPlus], http://www.guidetopharmacology.org/GRAC/LigandDisplayForward?ligandId=359 (p*K*_i_ 8.7) [http://www.ncbi.nlm.nih.gov/pubmed/20590605?dopt=AbstractPlus], http://www.guidetopharmacology.org/GRAC/LigandDisplayForward?ligandId=7128 (p*K*_d_ 8.6) [http://www.ncbi.nlm.nih.gov/pubmed/1346637?dopt=AbstractPlus], http://www.guidetopharmacology.org/GRAC/LigandDisplayForward?ligandId=8583 (p*K*i 8.3–8.4) [http://www.ncbi.nlm.nih.gov/pubmed/9671109?dopt=AbstractPlus, http://www.ncbi.nlm.nih.gov/pubmed/2043926?dopt=AbstractPlus]	http://www.guidetopharmacology.org/GRAC/LigandDisplayForward?ligandId=7459 (pIC_50_ 9.7) [http://www.ncbi.nlm.nih.gov/pubmed/21036043?dopt=AbstractPlus], http://www.guidetopharmacology.org/GRAC/LigandDisplayForward?ligandId=8586 (p*K*_i_ 9.5) [http://www.ncbi.nlm.nih.gov/pubmed/20590605?dopt=AbstractPlus], http://www.guidetopharmacology.org/GRAC/LigandDisplayForward?ligandId=307 (p*K*_i_ 9) [http://www.ncbi.nlm.nih.gov/pubmed/1994002?dopt=AbstractPlus], http://www.guidetopharmacology.org/GRAC/LigandDisplayForward?ligandId=360 (p*K*_i_ 8.5–8.8) [http://www.ncbi.nlm.nih.gov/pubmed/9671109?dopt=AbstractPlus, http://www.ncbi.nlm.nih.gov/pubmed/9671109?dopt=AbstractPlus], http://www.guidetopharmacology.org/GRAC/LigandDisplayForward?ligandId=321 (p*K*_i_ 7.9–8.6) [http://www.ncbi.nlm.nih.gov/pubmed/9671109?dopt=AbstractPlus, http://www.ncbi.nlm.nih.gov/pubmed/9113359?dopt=AbstractPlus, http://www.ncbi.nlm.nih.gov/pubmed/11303071?dopt=AbstractPlus]
Selective antagonists	–	–	http://www.guidetopharmacology.org/GRAC/LigandDisplayForward?ligandId=8591 (p*K*_i_ 6.3) [http://www.ncbi.nlm.nih.gov/pubmed/24692176?dopt=AbstractPlus]
Allosteric modulators	http://www.guidetopharmacology.org/GRAC/LigandDisplayForward?ligandId=340 (Positive) (p*K*_d_ 5.1) [http://www.ncbi.nlm.nih.gov/pubmed/12435818?dopt=AbstractPlus], http://www.guidetopharmacology.org/GRAC/LigandDisplayForward?ligandId=344 (Positive) (p*K*_d_ 3.3) [http://www.ncbi.nlm.nih.gov/pubmed/9495826?dopt=AbstractPlus]	http://www.guidetopharmacology.org/GRAC/LigandDisplayForward?ligandId=313 (Negative) (p*K*_i_ 8.7) [http://www.ncbi.nlm.nih.gov/pubmed/7925952?dopt=AbstractPlus, http://www.ncbi.nlm.nih.gov/pubmed/9862767?dopt=AbstractPlus], http://www.guidetopharmacology.org/GRAC/LigandDisplayForward?ligandId=3263 (Positive) (pEC_50_ 6.4) [http://www.ncbi.nlm.nih.gov/pubmed/18772318?dopt=AbstractPlus] – Rat, http://www.guidetopharmacology.org/GRAC/LigandDisplayForward?ligandId=3256 (Positive) (pEC_50_ 6.4) [http://www.ncbi.nlm.nih.gov/pubmed/18772318?dopt=AbstractPlus] – Rat, http://www.guidetopharmacology.org/GRAC/LigandDisplayForward?ligandId=6938 (Positive) (p*K*_d_ 5.7) [http://www.ncbi.nlm.nih.gov/pubmed/24807965?dopt=AbstractPlus], http://www.guidetopharmacology.org/GRAC/LigandDisplayForward?ligandId=348 (Positive) (p*K*_d_ 4) [http://www.ncbi.nlm.nih.gov/pubmed/14722259?dopt=AbstractPlus], http://www.guidetopharmacology.org/GRAC/LigandDisplayForward?ligandId=3262 (Positive) [http://www.ncbi.nlm.nih.gov/pubmed/18678919?dopt=AbstractPlus]	http://www.guidetopharmacology.org/GRAC/LigandDisplayForward?ligandId=8687 (Positive) (pEC_50_ 6.7) [http://www.ncbi.nlm.nih.gov/pubmed/27461343?dopt=AbstractPlus, http://www.ncbi.nlm.nih.gov/pubmed/25147929?dopt=AbstractPlus]
Selective allosteric modulators	–	–	http://www.guidetopharmacology.org/GRAC/LigandDisplayForward?ligandId=7631 (Negative) (pIC_50_ 6.5) [http://www.ncbi.nlm.nih.gov/pubmed/24164599?dopt=AbstractPlus]
Labelled ligands	[http://www.guidetopharmacology.org/GRAC/LigandDisplayForward?ligandId=8592 (Antagonist) (p*K*_d_ 10.7) [http://www.ncbi.nlm.nih.gov/pubmed/23435542?dopt=AbstractPlus], [http://www.guidetopharmacology.org/GRAC/LigandDisplayForward?ligandId=318 (Antagonist) (p*K*_d_ 10.4) [http://www.ncbi.nlm.nih.gov/pubmed/3443095?dopt=AbstractPlus], [http://www.guidetopharmacology.org/GRAC/LigandDisplayForward?ligandId=317 (Antagonist) (p*K*_d_ 9.7–10.2) [http://www.ncbi.nlm.nih.gov/pubmed/9846649?dopt=AbstractPlus, http://www.ncbi.nlm.nih.gov/pubmed/12235229?dopt=AbstractPlus, http://www.ncbi.nlm.nih.gov/pubmed/11303071?dopt=AbstractPlus, http://www.ncbi.nlm.nih.gov/pubmed/12049493?dopt=AbstractPlus, http://www.ncbi.nlm.nih.gov/pubmed/9224827?dopt=AbstractPlus, http://www.ncbi.nlm.nih.gov/pubmed/7925952?dopt=AbstractPlus, http://www.ncbi.nlm.nih.gov/pubmed/16369696?dopt=AbstractPlus, http://www.ncbi.nlm.nih.gov/pubmed/7651370?dopt=AbstractPlus], [http://www.guidetopharmacology.org/GRAC/LigandDisplayForward?ligandId=319 (Antagonist) (p*K*_d_ 9.5) [http://www.ncbi.nlm.nih.gov/pubmed/9029489?dopt=AbstractPlus]	[http://www.guidetopharmacology.org/GRAC/LigandDisplayForward?ligandId=318 (Antagonist) (p*K*_d_ 9.7–10.5) [http://www.ncbi.nlm.nih.gov/pubmed/11578621?dopt=AbstractPlus, http://www.ncbi.nlm.nih.gov/pubmed/3443095?dopt=AbstractPlus], [http://www.guidetopharmacology.org/GRAC/LigandDisplayForward?ligandId=317 (Antagonist) (p*K*_d_ 9.9–10.2) [http://www.ncbi.nlm.nih.gov/pubmed/9846649?dopt=AbstractPlus, http://www.ncbi.nlm.nih.gov/pubmed/12235229?dopt=AbstractPlus, http://www.ncbi.nlm.nih.gov/pubmed/11303071?dopt=AbstractPlus, http://www.ncbi.nlm.nih.gov/pubmed/9224827?dopt=AbstractPlus, http://www.ncbi.nlm.nih.gov/pubmed/7925952?dopt=AbstractPlus, http://www.ncbi.nlm.nih.gov/pubmed/16369696?dopt=AbstractPlus, http://www.ncbi.nlm.nih.gov/pubmed/7651370?dopt=AbstractPlus, http://www.ncbi.nlm.nih.gov/pubmed/9862767?dopt=AbstractPlus, http://www.ncbi.nlm.nih.gov/pubmed/7687290?dopt=AbstractPlus], [http://www.guidetopharmacology.org/GRAC/LigandDisplayForward?ligandId=8593 (Agonist) [http://www.ncbi.nlm.nih.gov/pubmed/14722259?dopt=AbstractPlus]	[http://www.guidetopharmacology.org/GRAC/LigandDisplayForward?ligandId=318 (Antagonist) (p*K*_d_ 10.2–10.7), [http://www.guidetopharmacology.org/GRAC/LigandDisplayForward?ligandId=317 (Antagonist) (p*K*_d_ 9.3–9.7) [http://www.ncbi.nlm.nih.gov/pubmed/9846649?dopt=AbstractPlus, http://www.ncbi.nlm.nih.gov/pubmed/12235229?dopt=AbstractPlus, http://www.ncbi.nlm.nih.gov/pubmed/11303071?dopt=AbstractPlus, http://www.ncbi.nlm.nih.gov/pubmed/7925952?dopt=AbstractPlus, http://www.ncbi.nlm.nih.gov/pubmed/16369696?dopt=AbstractPlus, http://www.ncbi.nlm.nih.gov/pubmed/7687290?dopt=AbstractPlus]

### Comments

The crystal structures of the M_1_‐M4 receptor subtypes have been reported [http://www.ncbi.nlm.nih.gov/pubmed/22278061?dopt=AbstractPlus, http://www.ncbi.nlm.nih.gov/pubmed/26958838?dopt=AbstractPlus, http://www.ncbi.nlm.nih.gov/pubmed/22358838?dopt=AbstractPlus]. Direct activation *via* an allosteric site has been reported for M_1_ receptors (http://www.guidetopharmacology.org/GRAC/LigandDisplayForward?ligandId=5436, http://www.guidetopharmacology.org/GRAC/LigandDisplayForward?ligandId=9228) and M4 receptors (http://www.guidetopharmacology.org/GRAC/LigandDisplayForward?ligandId=3262) [http://www.ncbi.nlm.nih.gov/pubmed/27275946?dopt=AbstractPlus, http://www.ncbi.nlm.nih.gov/pubmed/21300722?dopt=AbstractPlus, http://www.ncbi.nlm.nih.gov/pubmed/19940843?dopt=AbstractPlus, http://www.ncbi.nlm.nih.gov/pubmed/19717450?dopt=AbstractPlus, http://www.ncbi.nlm.nih.gov/pubmed/20406819?dopt=AbstractPlus, http://www.ncbi.nlm.nih.gov/pubmed/18628403?dopt=AbstractPlus]. The allosteric site for http://www.guidetopharmacology.org/GRAC/LigandDisplayForward?ligandId=356 and http://www.guidetopharmacology.org/GRAC/LigandDisplayForward?ligandId=347 on M_2_ receptors can be labelled by [http://www.guidetopharmacology.org/GRAC/LigandDisplayForward?ligandId=364 [http://www.ncbi.nlm.nih.gov/pubmed/12815174?dopt=AbstractPlus]. http://www.guidetopharmacology.org/GRAC/LigandDisplayForward?ligandId=290 is a functionally selective partial agonist that appears to interact in a bitopic mode with both the orthosteric and an allosteric site on the M_2_ muscarinic receptor [http://www.ncbi.nlm.nih.gov/pubmed/18723515?dopt=AbstractPlus]. http://www.guidetopharmacology.org/GRAC/LigandDisplayForward?ligandId=3255, hybrid 1 and hybrid 2, are multivalent (bitopic) ligands that also achieve selectivity for M_2_ receptors by binding both to the orthosteric and a nearby allosteric site [http://www.ncbi.nlm.nih.gov/pubmed/18842964?dopt=AbstractPlus, http://www.ncbi.nlm.nih.gov/pubmed/17478612?dopt=AbstractPlus].

Although numerous ligands for muscarinic acetylcholine receptors have been described, relatively few selective antagonists have been described, so it is common to assess the rank order of affinity of a number of antagonists of limited selectivity (*e.g.* http://www.guidetopharmacology.org/GRAC/LigandDisplayForward?ligandId=307, http://www.guidetopharmacology.org/GRAC/LigandDisplayForward?ligandId=321, http://www.guidetopharmacology.org/GRAC/LigandDisplayForward?ligandId=328) in order to identify the involvement of particular subtypes. It should be noted that the measured affinities of antagonists (and agonists) in radioligand binding studies are sensitive to ionic strength and can increase over 10‐fold at low ionic strength compared to their values at physiological ionic strengths [http://www.ncbi.nlm.nih.gov/pubmed/497538?dopt=AbstractPlus].

### Further reading on Acetylcholine receptors (muscarinic)

Burger WAC *et al*. (2018) Toward an understanding of the structural basis of allostery in muscarinic acetylcholine receptors. *J Gen Physiol* **150**: 1360‐1372 [https://www.ncbi.nlm.nih.gov/pubmed/30190312?dopt=AbstractPlus]

Caulfield MP *et al*. (1998) International Union of Pharmacology. XVII. Classification of muscarinic acetylcholine receptors. *Pharmacol Rev* **50**: 279‐290 [https://www.ncbi.nlm.nih.gov/pubmed/9647869?dopt=AbstractPlus]

Eglen RM. (2012) Overview of muscarinic receptor subtypes. *Handb Exp Pharmacol* 3‐28 [https://www.ncbi.nlm.nih.gov/pubmed/22222692?dopt=AbstractPlus]

Kruse AC *et al*. (2014) Muscarinic acetylcholine receptors: novel opportunities for drug development. *Nat Rev Drug Discov* **13**: 549‐60 [https://www.ncbi.nlm.nih.gov/pubmed/24903776?dopt=AbstractPlus]

Leach K *et al*. (2012) Structure‐function studies of muscarinic acetylcholine receptors. *Handb Exp Pharmacol* 29‐48 [https://www.ncbi.nlm.nih.gov/pubmed/22222693?dopt=AbstractPlus]

Valant C *et al*. (2012) The best of both worlds? Bitopic orthosteric/allosteric ligands of g proteincoupled receptors. *Annu Rev Pharmacol Toxicol* **52**: 153‐78 [https://www.ncbi.nlm.nih.gov/pubmed/21910627?dopt=AbstractPlus]

## http://www.guidetopharmacology.org/GRAC/FamilyDisplayForward?familyId=3

## Overview

Adenosine receptors (**nomenclature as agreed by the NC‐IUPHAR Subcommittee on Adenosine Receptors** [http://www.ncbi.nlm.nih.gov/pubmed/11734617?dopt=AbstractPlus]) are activated by the endogenous ligand http://www.guidetopharmacology.org/GRAC/LigandDisplayForward?ligandId=2844 (potentially http://www.guidetopharmacology.org/GRAC/LigandDisplayForward?ligandId=4554 also at A_3_ receptors). Crystal structures for the antagonist‐bound [http://www.ncbi.nlm.nih.gov/pubmed/22220592?dopt=AbstractPlus, http://www.ncbi.nlm.nih.gov/pubmed/18832607?dopt=AbstractPlus, http://www.ncbi.nlm.nih.gov/pubmed/22798613?dopt=AbstractPlus, http://www.ncbi.nlm.nih.gov/pubmed/27312113?dopt=AbstractPlus], agonist‐bound [http://www.ncbi.nlm.nih.gov/pubmed/25762024?dopt=AbstractPlus, http://www.ncbi.nlm.nih.gov/pubmed/21593763?dopt=AbstractPlus, http://www.ncbi.nlm.nih.gov/pubmed/21393508?dopt=AbstractPlus] and G protein‐bound A_2A_ adenosine receptors [http://www.ncbi.nlm.nih.gov/pubmed/27462812?dopt=AbstractPlus] have been described. The structures of an antagonist‐bound A1 receptor [http://www.ncbi.nlm.nih.gov/pubmed/28235198?dopt=AbstractPlus] and an adenosine‐bound A1 receptor‐G_i_ complex [http://www.ncbi.nlm.nih.gov/pubmed/29925945?dopt=AbstractPlus] have been resolved by cryo‐electronmicroscopy. Another structure of an antagonist‐bound A1 receptor obtained with X‐ray crystallography has also been reported [http://www.ncbi.nlm.nih.gov/pubmed/28712806?dopt=AbstractPlus].

**Table:** 

Nomenclature	http://www.guidetopharmacology.org/GRAC/ObjectDisplayForward?objectId=18	http://www.guidetopharmacology.org/GRAC/ObjectDisplayForward?objectId=19	http://www.guidetopharmacology.org/GRAC/ObjectDisplayForward?objectId=20	http://www.guidetopharmacology.org/GRAC/ObjectDisplayForward?objectId=21
HGNC, UniProt	https://www.genenames.org/data/gene‐symbol‐report/#!/hgnc_id/HGNC:262, http://www.uniprot.org/uniprot/P30542	https://www.genenames.org/data/gene‐symbol‐report/#!/hgnc_id/HGNC:263, http://www.uniprot.org/uniprot/P29274	https://www.genenames.org/data/gene‐symbol‐report/#!/hgnc_id/HGNC:264, http://www.uniprot.org/uniprot/P29275	https://www.genenames.org/data/gene‐symbol‐report/#!/hgnc_id/HGNC:268, http://www.uniprot.org/uniprot/P0DMS8
Endogenous agonists	http://www.guidetopharmacology.org/GRAC/LigandDisplayForward?ligandId=2844 [http://www.ncbi.nlm.nih.gov/pubmed/14662005?dopt=AbstractPlus]	http://www.guidetopharmacology.org/GRAC/LigandDisplayForward?ligandId=2844 [http://www.ncbi.nlm.nih.gov/pubmed/14662005?dopt=AbstractPlus]	http://www.guidetopharmacology.org/GRAC/LigandDisplayForward?ligandId=2844 [http://www.ncbi.nlm.nih.gov/pubmed/14662005?dopt=AbstractPlus]	http://www.guidetopharmacology.org/GRAC/LigandDisplayForward?ligandId=2844 [http://www.ncbi.nlm.nih.gov/pubmed/14662005?dopt=AbstractPlus]
Agonists	http://www.guidetopharmacology.org/GRAC/LigandDisplayForward?ligandId=377 [http://www.ncbi.nlm.nih.gov/pubmed/15476669?dopt=AbstractPlus, http://www.ncbi.nlm.nih.gov/pubmed/7798201?dopt=AbstractPlus, http://www.ncbi.nlm.nih.gov/pubmed/9920910?dopt=AbstractPlus, http://www.ncbi.nlm.nih.gov/pubmed/8300561?dopt=AbstractPlus, http://www.ncbi.nlm.nih.gov/pubmed/14662005?dopt=AbstractPlus]	http://www.guidetopharmacology.org/GRAC/LigandDisplayForward?ligandId=377 [http://www.ncbi.nlm.nih.gov/pubmed/15267242?dopt=AbstractPlus, http://www.ncbi.nlm.nih.gov/pubmed/9179373?dopt=AbstractPlus, http://www.ncbi.nlm.nih.gov/pubmed/15476669?dopt=AbstractPlus, http://www.ncbi.nlm.nih.gov/pubmed/7775460?dopt=AbstractPlus, http://www.ncbi.nlm.nih.gov/pubmed/9920286?dopt=AbstractPlus, http://www.ncbi.nlm.nih.gov/pubmed/14662005?dopt=AbstractPlus]	http://www.guidetopharmacology.org/GRAC/LigandDisplayForward?ligandId=377 [http://www.ncbi.nlm.nih.gov/pubmed/11093773?dopt=AbstractPlus, http://www.ncbi.nlm.nih.gov/pubmed/15267242?dopt=AbstractPlus, http://www.ncbi.nlm.nih.gov/pubmed/11266650?dopt=AbstractPlus, http://www.ncbi.nlm.nih.gov/pubmed/10496952?dopt=AbstractPlus, http://www.ncbi.nlm.nih.gov/pubmed/15194002?dopt=AbstractPlus, http://www.ncbi.nlm.nih.gov/pubmed/16219300?dopt=AbstractPlus, http://www.ncbi.nlm.nih.gov/pubmed/14662005?dopt=AbstractPlus]	http://www.guidetopharmacology.org/GRAC/LigandDisplayForward?ligandId=377 [http://www.ncbi.nlm.nih.gov/pubmed/15267242?dopt=AbstractPlus, http://www.ncbi.nlm.nih.gov/pubmed/15476669?dopt=AbstractPlus, http://www.ncbi.nlm.nih.gov/pubmed/9364471?dopt=AbstractPlus, http://www.ncbi.nlm.nih.gov/pubmed/8234299?dopt=AbstractPlus, http://www.ncbi.nlm.nih.gov/pubmed/10779381?dopt=AbstractPlus, http://www.ncbi.nlm.nih.gov/pubmed/14662005?dopt=AbstractPlus]
Selective agonists	http://www.guidetopharmacology.org/GRAC/LigandDisplayForward?ligandId=380 [http://www.ncbi.nlm.nih.gov/pubmed/9827575?dopt=AbstractPlus, http://www.ncbi.nlm.nih.gov/pubmed/15740718?dopt=AbstractPlus, http://www.ncbi.nlm.nih.gov/pubmed/15476669?dopt=AbstractPlus, http://www.ncbi.nlm.nih.gov/pubmed/16444290?dopt=AbstractPlus, http://www.ncbi.nlm.nih.gov/pubmed/16518376?dopt=AbstractPlus, http://www.ncbi.nlm.nih.gov/pubmed/7798201?dopt=AbstractPlus, http://www.ncbi.nlm.nih.gov/pubmed/9920910?dopt=AbstractPlus], http://www.guidetopharmacology.org/GRAC/LigandDisplayForward?ligandId=6560)‐http://www.guidetopharmacology.org/GRAC/LigandDisplayForward?ligandId=6560 [http://www.ncbi.nlm.nih.gov/pubmed/19317449?dopt=AbstractPlus], http://www.guidetopharmacology.org/GRAC/LigandDisplayForward?ligandId=5590 [http://www.ncbi.nlm.nih.gov/pubmed/12672250?dopt=AbstractPlus], http://www.guidetopharmacology.org/GRAC/LigandDisplayForward?ligandId=374[http://www.ncbi.nlm.nih.gov/pubmed/16518376?dopt=AbstractPlus, http://www.ncbi.nlm.nih.gov/pubmed/16020631?dopt=AbstractPlus], http://www.guidetopharmacology.org/GRAC/LigandDisplayForward?ligandId=10235 [http://www.ncbi.nlm.nih.gov/pubmed/30605331?dopt=AbstractPlus]	http://www.guidetopharmacology.org/GRAC/LigandDisplayForward?ligandId=3290 [http://www.ncbi.nlm.nih.gov/pubmed/11406470?dopt=AbstractPlus], http://www.guidetopharmacology.org/GRAC/LigandDisplayForward?ligandId=8420 [http://www.ncbi.nlm.nih.gov/pubmed/22324512?dopt=AbstractPlus, http://www.ncbi.nlm.nih.gov/pubmed/21393508?dopt=AbstractPlus], http://www.guidetopharmacology.org/GRAC/LigandDisplayForward?ligandId=9236 [http://www.ncbi.nlm.nih.gov/pubmed/22220592?dopt=AbstractPlus], http://www.guidetopharmacology.org/GRAC/LigandDisplayForward?ligandId=375 [http://www.ncbi.nlm.nih.gov/pubmed/15267242?dopt=AbstractPlus, http://www.ncbi.nlm.nih.gov/pubmed/9179373?dopt=AbstractPlus, http://www.ncbi.nlm.nih.gov/pubmed/15476669?dopt=AbstractPlus, http://www.ncbi.nlm.nih.gov/pubmed/16518376?dopt=AbstractPlus, http://www.ncbi.nlm.nih.gov/pubmed/7775460?dopt=AbstractPlus, http://www.ncbi.nlm.nih.gov/pubmed/9459566?dopt=AbstractPlus, http://www.ncbi.nlm.nih.gov/pubmed/9920286?dopt=AbstractPlus, http://www.ncbi.nlm.nih.gov/pubmed/16020631?dopt=AbstractPlus], http://www.guidetopharmacology.org/GRAC/LigandDisplayForward?ligandId=5596[http://www.ncbi.nlm.nih.gov/pubmed/16518376?dopt=AbstractPlus]	http://www.guidetopharmacology.org/GRAC/LigandDisplayForward?ligandId=3289 [http://www.ncbi.nlm.nih.gov/pubmed/17353435?dopt=AbstractPlus]	http://www.guidetopharmacology.org/GRAC/LigandDisplayForward?ligandId=422 [http://www.ncbi.nlm.nih.gov/pubmed/11705449?dopt=AbstractPlus, http://www.ncbi.nlm.nih.gov/pubmed/8126704?dopt=AbstractPlus, http://www.ncbi.nlm.nih.gov/pubmed/9459566?dopt=AbstractPlus, http://www.ncbi.nlm.nih.gov/pubmed/10779381?dopt=AbstractPlus], http://www.guidetopharmacology.org/GRAC/LigandDisplayForward?ligandId=457 [http://www.ncbi.nlm.nih.gov/pubmed/10731034?dopt=AbstractPlus, http://www.ncbi.nlm.nih.gov/pubmed/9364471?dopt=AbstractPlus, http://www.ncbi.nlm.nih.gov/pubmed/7932588?dopt=AbstractPlus], http://www.guidetopharmacology.org/GRAC/LigandDisplayForward?ligandId=8421 [http://www.ncbi.nlm.nih.gov/pubmed/22559880?dopt=AbstractPlus]
Antagonists	http://www.guidetopharmacology.org/GRAC/LigandDisplayForward?ligandId=384 (p*K*_i_ 8.5) [http://www.ncbi.nlm.nih.gov/pubmed/9933143?dopt=AbstractPlus], http://www.guidetopharmacology.org/GRAC/LigandDisplayForward?ligandId=404 (p*K*_d_ 7.5) [http://www.ncbi.nlm.nih.gov/pubmed/19317449?dopt=AbstractPlus]	http://www.guidetopharmacology.org/GRAC/LigandDisplayForward?ligandId=384 (p*K*_i_ 7.7–9.4) [http://www.ncbi.nlm.nih.gov/pubmed/9179373?dopt=AbstractPlus, http://www.ncbi.nlm.nih.gov/pubmed/7775460?dopt=AbstractPlus, http://www.ncbi.nlm.nih.gov/pubmed/9459566?dopt=AbstractPlus, http://www.ncbi.nlm.nih.gov/pubmed/9933143?dopt=AbstractPlus], http://www.guidetopharmacology.org/GRAC/LigandDisplayForward?ligandId=404 (p*K*_i_ 8.4–9) [http://www.ncbi.nlm.nih.gov/pubmed/9179373?dopt=AbstractPlus, http://www.ncbi.nlm.nih.gov/pubmed/9179373?dopt=AbstractPlus]	http://www.guidetopharmacology.org/GRAC/LigandDisplayForward?ligandId=404 (p*K*_i_ 6.9–8.8) [http://www.ncbi.nlm.nih.gov/pubmed/11093773?dopt=AbstractPlus, http://www.ncbi.nlm.nih.gov/pubmed/11266650?dopt=AbstractPlus, http://www.ncbi.nlm.nih.gov/pubmed/10624567?dopt=AbstractPlus, http://www.ncbi.nlm.nih.gov/pubmed/9459566?dopt=AbstractPlus, http://www.ncbi.nlm.nih.gov/pubmed/10496952?dopt=AbstractPlus, http://www.ncbi.nlm.nih.gov/pubmed/15194002?dopt=AbstractPlus], http://www.guidetopharmacology.org/GRAC/LigandDisplayForward?ligandId=384 (p*K*_i_ 6–8.1) [http://www.ncbi.nlm.nih.gov/pubmed/19141710?dopt=AbstractPlus, http://www.ncbi.nlm.nih.gov/pubmed/10624567?dopt=AbstractPlus, http://www.ncbi.nlm.nih.gov/pubmed/9459566?dopt=AbstractPlus, http://www.ncbi.nlm.nih.gov/pubmed/9933143?dopt=AbstractPlus, http://www.ncbi.nlm.nih.gov/pubmed/15194002?dopt=AbstractPlus]	http://www.guidetopharmacology.org/GRAC/LigandDisplayForward?ligandId=384 (p*K*_i_ 7–7.9) [http://www.ncbi.nlm.nih.gov/pubmed/8863790?dopt=AbstractPlus, http://www.ncbi.nlm.nih.gov/pubmed/9459566?dopt=AbstractPlus, http://www.ncbi.nlm.nih.gov/pubmed/9933143?dopt=AbstractPlus, http://www.ncbi.nlm.nih.gov/pubmed/10779381?dopt=AbstractPlus], http://www.guidetopharmacology.org/GRAC/LigandDisplayForward?ligandId=404 (p*K*_i_ 7–7.4) [http://www.ncbi.nlm.nih.gov/pubmed/9459566?dopt=AbstractPlus, http://www.ncbi.nlm.nih.gov/pubmed/8234299?dopt=AbstractPlus]
Selective antagonists	http://www.guidetopharmacology.org/GRAC/LigandDisplayForward?ligandId=3285 (p*K*_i_ 9.9) [http://www.ncbi.nlm.nih.gov/pubmed/14563788?dopt=AbstractPlus] – Rat, http://www.guidetopharmacology.org/GRAC/LigandDisplayForward?ligandId=386 (p*K*i 7.4–9.2) [http://www.ncbi.nlm.nih.gov/pubmed/15740718?dopt=AbstractPlus, http://www.ncbi.nlm.nih.gov/pubmed/8032613?dopt=AbstractPlus, http://www.ncbi.nlm.nih.gov/pubmed/16020631?dopt=AbstractPlus, http://www.ncbi.nlm.nih.gov/pubmed/9920910?dopt=AbstractPlus, http://www.ncbi.nlm.nih.gov/pubmed/16902942?dopt=AbstractPlus], http://www.guidetopharmacology.org/GRAC/LigandDisplayForward?ligandId=3281 (p*K*_i_ 9) [http://www.ncbi.nlm.nih.gov/pubmed/17558436?dopt=AbstractPlus], http://www.guidetopharmacology.org/GRAC/LigandDisplayForward?ligandId=8419 (p*K*_i_ 8.8) [http://www.ncbi.nlm.nih.gov/pubmed/8632314?dopt=AbstractPlus], http://www.guidetopharmacology.org/GRAC/LigandDisplayForward?ligandId=9530 (p*K*_i_ 7.4) [http://www.ncbi.nlm.nih.gov/pubmed/28235198?dopt=AbstractPlus]	http://www.guidetopharmacology.org/GRAC/LigandDisplayForward?ligandId=3283 (p*K*_i_ 8.4–10.3) [http://www.ncbi.nlm.nih.gov/pubmed/20801028?dopt=AbstractPlus, http://www.ncbi.nlm.nih.gov/pubmed/11087559?dopt=AbstractPlus], http://www.guidetopharmacology.org/GRAC/LigandDisplayForward?ligandId=405 (p*K*_i_ 8.8–9.1) [http://www.ncbi.nlm.nih.gov/pubmed/9933143?dopt=AbstractPlus]	http://www.guidetopharmacology.org/GRAC/LigandDisplayForward?ligandId=6561 (p*K*_i_ 9.4) [http://www.ncbi.nlm.nih.gov/pubmed/19569717?dopt=AbstractPlus], http://www.guidetopharmacology.org/GRAC/LigandDisplayForward?ligandId=3284 (p*K*_i_ 9.3) [http://www.ncbi.nlm.nih.gov/pubmed/19569717?dopt=AbstractPlus], http://www.guidetopharmacology.org/GRAC/LigandDisplayForward?ligandId=449 (p*K*_i_ 8.8) [http://www.ncbi.nlm.nih.gov/pubmed/11266650?dopt=AbstractPlus, http://www.ncbi.nlm.nih.gov/pubmed/10737749?dopt=AbstractPlus], http://www.guidetopharmacology.org/GRAC/LigandDisplayForward?ligandId=3286 (p*K*_i_ 7.3) [http://www.ncbi.nlm.nih.gov/pubmed/11906291?dopt=AbstractPlus]	http://www.guidetopharmacology.org/GRAC/LigandDisplayForward?ligandId=448 (p*K*_i_ 8.2–9.2) [http://www.ncbi.nlm.nih.gov/pubmed/9364471?dopt=AbstractPlus, http://www.ncbi.nlm.nih.gov/pubmed/8863790?dopt=AbstractPlus, http://www.ncbi.nlm.nih.gov/pubmed/11226132?dopt=AbstractPlus, http://www.ncbi.nlm.nih.gov/pubmed/16553647?dopt=AbstractPlus], http://www.guidetopharmacology.org/GRAC/LigandDisplayForward?ligandId=3280 (p*K*_i_ 8.4) [http://www.ncbi.nlm.nih.gov/pubmed/10841801?dopt=AbstractPlus], http://www.guidetopharmacology.org/GRAC/LigandDisplayForward?ligandId=474 (p*K*_i_ 7.7) [http://www.ncbi.nlm.nih.gov/pubmed/9703464?dopt=AbstractPlus], http://www.guidetopharmacology.org/GRAC/LigandDisplayForward?ligandId=470 (p*K*_i_ 7.5) [http://www.ncbi.nlm.nih.gov/pubmed/9364471?dopt=AbstractPlus, http://www.ncbi.nlm.nih.gov/pubmed/8917655?dopt=AbstractPlus, 1273]
Allosteric modulators	http://www.guidetopharmacology.org/GRAC/LigandDisplayForward?ligandId=9445 (Positive) [http://www.ncbi.nlm.nih.gov/pubmed/2174510?dopt=AbstractPlus]	–	–	http://www.guidetopharmacology.org/GRAC/LigandDisplayForward?ligandId=9446 (Positive) [http://www.ncbi.nlm.nih.gov/pubmed/16722654?dopt=AbstractPlus], http://www.guidetopharmacology.org/GRAC/LigandDisplayForward?ligandId=9447 (Positive) [http://www.ncbi.nlm.nih.gov/pubmed/19161279?dopt=AbstractPlus]
Labelled ligands	[http://www.guidetopharmacology.org/GRAC/LigandDisplayForward?ligandId=379 (Agonist) [http://www.ncbi.nlm.nih.gov/pubmed/9459566?dopt=AbstractPlus, http://www.ncbi.nlm.nih.gov/pubmed/9920910?dopt=AbstractPlus], [http://www.guidetopharmacology.org/GRAC/LigandDisplayForward?ligandId=406 (Antagonist) (p*K*_d_ 8.4–9.2) [http://www.ncbi.nlm.nih.gov/pubmed/9827575?dopt=AbstractPlus, http://www.ncbi.nlm.nih.gov/pubmed/11705449?dopt=AbstractPlus, http://www.ncbi.nlm.nih.gov/pubmed/9933143?dopt=AbstractPlus, http://www.ncbi.nlm.nih.gov/pubmed/9920910?dopt=AbstractPlus, http://www.ncbi.nlm.nih.gov/pubmed/8300561?dopt=AbstractPlus]	[http://www.guidetopharmacology.org/GRAC/LigandDisplayForward?ligandId=455 (Antagonist) (p*K*_d_ 8.7–9.1) [http://www.ncbi.nlm.nih.gov/pubmed/11164377?dopt=AbstractPlus, http://www.ncbi.nlm.nih.gov/pubmed/10927024?dopt=AbstractPlus], [http://www.guidetopharmacology.org/GRAC/LigandDisplayForward?ligandId=424 (Agonist) [http://www.ncbi.nlm.nih.gov/pubmed/2600819?dopt=AbstractPlus, http://www.ncbi.nlm.nih.gov/pubmed/2213023?dopt=AbstractPlus]	[http://www.guidetopharmacology.org/GRAC/LigandDisplayForward?ligandId=453 (Antagonist) (p*K*_d_ 9.8) [http://www.ncbi.nlm.nih.gov/pubmed/11266650?dopt=AbstractPlus]	[http://www.guidetopharmacology.org/GRAC/LigandDisplayForward?ligandId=436 (Agonist) [http://www.ncbi.nlm.nih.gov/pubmed/9933143?dopt=AbstractPlus, http://www.ncbi.nlm.nih.gov/pubmed/10779381?dopt=AbstractPlus]

### Comments

Adenosine inhibits many intracellular ATP‐utilising enzymes, including adenylyl cyclase (P‐site). A pseudogene exists for the A_2B_ adenosine receptor (https://www.genenames.org/data/gene‐symbol‐report/#!/hgnc_id/HGNC:265) with 79% identity to the A_2B_ adenosine receptor cDNA coding sequence, but which is unable to encode a functional receptor [http://www.ncbi.nlm.nih.gov/pubmed/7558011?dopt=AbstractPlus]. http://www.guidetopharmacology.org/GRAC/LigandDisplayForward?ligandId=386 also exhibits antagonism at A_2B_ receptors (p*K*i ca. 7,[http://www.ncbi.nlm.nih.gov/pubmed/8937736?dopt=AbstractPlus, http://www.ncbi.nlm.nih.gov/pubmed/9459566?dopt=AbstractPlus]). Antagonists at A_3_ receptors exhibit marked species differences, such that only http://www.guidetopharmacology.org/GRAC/LigandDisplayForward?ligandId=474 and http://www.guidetopharmacology.org/GRAC/LigandDisplayForward?ligandId=470 are selective at the rat A_3_ receptor. In the absence of other adenosine receptors, [http://www.guidetopharmacology.org/GRAC/LigandDisplayForward?ligandId=406 and [http://www.guidetopharmacology.org/GRAC/LigandDisplayForward?ligandId=455 can also be used to label A_2B_ receptors (KD *ca*. 30 and 60 nM respectively). [http://www.guidetopharmacology.org/GRAC/LigandDisplayForward?ligandId=436 also binds to A_1_ receptors [http://www.ncbi.nlm.nih.gov/pubmed/9459566?dopt=AbstractPlus]. [http://www.guidetopharmacology.org/GRAC/LigandDisplayForward?ligandId=424 is relatively selective for A_2A_ receptors, but may also bind to other sites in cerebral cortex [http://www.ncbi.nlm.nih.gov/pubmed/8692280?dopt=AbstractPlus, http://www.ncbi.nlm.nih.gov/pubmed/7566470?dopt=AbstractPlus]. [http://www.guidetopharmacology.org/GRAC/LigandDisplayForward?ligandId=425 binds to other non‐receptor elements, which also recognise adenosine [http://www.ncbi.nlm.nih.gov/pubmed/8937447?dopt=AbstractPlus]. http://www.guidetopharmacology.org/GRAC/LigandDisplayForward?ligandId=3279 has been described as a fluorescent antagonist for labelling A_1_ adenosine receptors in living cells, although activity at other adenosine receptors was not examined [http://www.ncbi.nlm.nih.gov/pubmed/15070776?dopt=AbstractPlus].

### Further reading on Adenosine receptors

Borea PA *et al*. (2015) The A_3_ adenosine receptor: history and perspectives. *Pharmacol Rev* **67**: 74‐102 [https://www.ncbi.nlm.nih.gov/pubmed/25387804?dopt=AbstractPlus]

Cronstein BN *et al*. (2017) Adenosine and adenosine receptors in the pathogenesis and treatment of rheumatic diseases. *Nat Rev Rheumatol* **13**: 41‐51 [https://www.ncbi.nlm.nih.gov/pubmed/27829671?dopt=AbstractPlus]

Fredholm BB *et al*. (2011) International Union of Basic and Clinical Pharmacology. LXXXI. Nomenclature and classification of adenosine receptors–an update. *Pharmacol Rev* **63**: 1‐34 [https://www.ncbi.nlm.nih.gov/pubmed/21303899?dopt=AbstractPlus]

Guo D *et al*. (2017) Kinetic Aspects of the Interaction between Ligand and G Protein‐Coupled Receptor: The Case of the Adenosine Receptors. *Chem Rev* **117**: 38‐66 [https://www.ncbi.nlm.nih.gov/pubmed/27088232?dopt=AbstractPlus]

Göblyös A *et al*. (2011) Allosteric modulation of adenosine receptors. *Biochim Biophys Acta* **1808**: 1309‐18 [https://www.ncbi.nlm.nih.gov/pubmed/20599682?dopt=AbstractPlus]

Lasley RD. (2011) Adenosine receptors and membrane microdomains. *Biochim Biophys Acta* **1808**: 1284‐9 [https://www.ncbi.nlm.nih.gov/pubmed/20888790?dopt=AbstractPlus]

Mundell S *et al*. (2011) Adenosine receptor desensitization and trafficking. *Biochim Biophys Acta* **1808**: 1319‐28 [https://www.ncbi.nlm.nih.gov/pubmed/20550943?dopt=AbstractPlus]

Vecchio EA *et al*. (2018) New paradigms in adenosine receptor pharmacology: allostery, oligomerization and biased agonism. *Br J Pharmacol* **175**: 4036‐4046 [https://www.ncbi.nlm.nih.gov/pubmed/29679502?dopt=AbstractPlus]

Wei CJ *etal*. (2011)Normaland abnormalfunctions ofadenosine receptors inthecentral nervous system revealed by genetic knockout studies. *Biochim Biophys Acta* **1808**: 1358‐79[https://www.ncbi.nlm.nih.gov/pubmed/21185258?dopt=AbstractPlus]

## http://www.guidetopharmacology.org/GRAC/FamilyDisplayForward?familyId=17

### Overview

Adhesion GPCRs are structurally identified on the basis of a large extracellular region, similar to the Class B GPCR, but which is linked to the 7TM region by a GPCR autoproteolysisinducing (GAIN) domain [http://www.ncbi.nlm.nih.gov/pubmed/22333914?dopt=AbstractPlus] containing a GPCR proteolytic site. The N‐terminus often shares structural homology with adhesive domains (e.g. cadherins, immunolobulin, lectins) facilitating inter‐ and matricellular interactions and leading to the term adhesion GPCR [http://www.ncbi.nlm.nih.gov/pubmed/12761335?dopt=AbstractPlus, http://www.ncbi.nlm.nih.gov/pubmed/18789697?dopt=AbstractPlus]. Several receptors have been suggested to function as mechanosensors [http://www.ncbi.nlm.nih.gov/pubmed/26841242?dopt=AbstractPlus, http://www.ncbi.nlm.nih.gov/pubmed/25695270?dopt=AbstractPlus, http://www.ncbi.nlm.nih.gov/pubmed/25937282?dopt=AbstractPlus, http://www.ncbi.nlm.nih.gov/pubmed/26499266?dopt=AbstractPlus]. **The nomenclature of these receptors was revised in 2015 as recommended by** **NC‐IUPHAR** **and the** **Adhesion GPCR Consortium** [http://www.ncbi.nlm.nih.gov/pubmed/25713288?dopt=AbstractPlus].

**Table:** 

Nomenclature	http://www.guidetopharmacology.org/GRAC/ObjectDisplayForward?objectId=197	http://www.guidetopharmacology.org/GRAC/ObjectDisplayForward?objectId=198	http://www.guidetopharmacology.org/GRAC/ObjectDisplayForward?objectId=199	http://www.guidetopharmacology.org/GRAC/ObjectDisplayForward?objectId=174	http://www.guidetopharmacology.org/GRAC/ObjectDisplayForward?objectId=175	http://www.guidetopharmacology.org/GRAC/ObjectDisplayForward?objectId=176
HGNC, UniProt	https://www.genenames.org/data/gene‐symbol‐report/#!/hgnc_id/HGNC:13838, http://www.uniprot.org/uniprot/Q86SQ6	https://www.genenames.org/data/gene‐symbol‐report/#!/hgnc_id/HGNC:17849, http://www.uniprot.org/uniprot/Q96PE1	https://www.genenames.org/data/gene‐symbol‐report/#!/hgnc_id/HGNC:13839, http://www.uniprot.org/uniprot/Q8IWK6	https://www.genenames.org/data/gene‐symbol‐report/#!/hgnc_id/HGNC:943, http://www.uniprot.org/uniprot/O14514	https://www.genenames.org/data/gene‐symbol‐report/#!/hgnc_id/HGNC:944, http://www.uniprot.org/uniprot/O60241	https://www.genenames.org/data/gene‐symbol‐report/#!/hgnc_id/HGNC:945, http://www.uniprot.org/uniprot/O60242
Endogenous agonists	–	–	–	http://www.guidetopharmacology.org/GRAC/LigandDisplayForward?ligandId=3638 [http://www.ncbi.nlm.nih.gov/pubmed/17960134?dopt=AbstractPlus]	–	–
Comments	–	Required to assemble higher‐order Reck/Gpr124/Frizzled/ Lrp5/6 complexes [http://www.ncbi.nlm.nih.gov/pubmed/30026314?dopt=AbstractPlus, http://www.ncbi.nlm.nih.gov/pubmed/25558062?dopt=AbstractPlus, http://www.ncbi.nlm.nih.gov/pubmed/30304675?dopt=AbstractPlus, http://www.ncbi.nlm.nih.gov/pubmed/26051822?dopt=AbstractPlus, http://www.ncbi.nlm.nih.gov/pubmed/25373781?dopt=AbstractPlus].	–	Reported to mediate phagocytosis through binding of phosphatidylserine [http://www.ncbi.nlm.nih.gov/pubmed/17960134?dopt=AbstractPlus] and lipopolysaccharide [http://www.ncbi.nlm.nih.gov/pubmed/21245295?dopt=AbstractPlus].	–	Reported to bind C1q‐like molecules [http://www.ncbi.nlm.nih.gov/pubmed/21262840?dopt=AbstractPlus].

**Table:** 

Nomenclature	http://www.guidetopharmacology.org/GRAC/ObjectDisplayForward?objectId=178	http://www.guidetopharmacology.org/GRAC/ObjectDisplayForward?objectId=179	http://www.guidetopharmacology.org/GRAC/ObjectDisplayForward?objectId=180	http://www.guidetopharmacology.org/GRAC/ObjectDisplayForward?objectId=202	http://www.guidetopharmacology.org/GRAC/ObjectDisplayForward?objectId=204
HGNC, UniProt	https://www.genenames.org/data/gene‐symbol‐report/#!/hgnc_id/HGNC:1850, http://www.uniprot.org/uniprot/Q9NYQ6	https://www.genenames.org/data/gene‐symbol‐report/#!/hgnc_id/HGNC:3231, http://www.uniprot.org/uniprot/Q9HCU4	https://www.genenames.org/data/gene‐symbol‐report/#!/hgnc_id/HGNC:3230, http://www.uniprot.org/uniprot/Q9NYQ7	https://www.genenames.org/data/gene‐symbol‐report/#!/hgnc_id/HGNC:19893, http://www.uniprot.org/uniprot/Q6QNK2	https://www.genenames.org/data/gene‐symbol‐report/#!/hgnc_id/HGNC:18651, http://www.uniprot.org/uniprot/Q7Z7M1
Comments	–	Mutated in Joubert syndrome patients [http://www.ncbi.nlm.nih.gov/pubmed/28052552?dopt=AbstractPlus].	High‐confidence risk gene for Tourette syndrome [http://www.ncbi.nlm.nih.gov/pubmed/30257206?dopt=AbstractPlus].	Is a G_s_ protein‐coupled receptor [http://www.ncbi.nlm.nih.gov/pubmed/22025619?dopt=AbstractPlus, http://www.ncbi.nlm.nih.gov/pubmed/25533341?dopt=AbstractPlus] and highly expressed in glioblastoma [http://www.ncbi.nlm.nih.gov/pubmed/27775701?dopt=AbstractPlus].	–

**Table:** 

Nomenclature	http://www.guidetopharmacology.org/GRAC/ObjectDisplayForward?objectId=182	http://www.guidetopharmacology.org/GRAC/ObjectDisplayForward?objectId=183	http://www.guidetopharmacology.org/GRAC/ObjectDisplayForward?objectId=184	http://www.guidetopharmacology.org/GRAC/ObjectDisplayForward?objectId=185	http://www.guidetopharmacology.org/GRAC/ObjectDisplayForward?objectId=177
HGNC, UniProt	https://www.genenames.org/data/gene‐symbol‐report/#!/hgnc_id/HGNC:3336, http://www.uniprot.org/uniprot/Q14246	https://www.genenames.org/data/gene‐symbol‐report/#!/hgnc_id/HGNC:3337, http://www.uniprot.org/uniprot/Q9UHX3	https://www.genenames.org/data/gene‐symbol‐report/#!/hgnc_id/HGNC:23647, http://www.uniprot.org/uniprot/Q9BY15	https://www.genenames.org/data/gene‐symbol‐report/#!/hgnc_id/HGNC:19240, http://www.uniprot.org/uniprot/Q86SQ3	https://www.genenames.org/data/gene‐symbol‐report/#!/hgnc_id/HGNC:1711, http://www.uniprot.org/uniprot/P48960
Comments	–	A mutation destabilizing the GAIN domain sensitizes mast cells to IgE‐independent vibration‐induced degranulation [http://www.ncbi.nlm.nih.gov/pubmed/26841242?dopt=AbstractPlus]. Reported to bind chondroitin sulfate B [http://www.ncbi.nlm.nih.gov/pubmed/12829604?dopt=AbstractPlus].	–	–	Reported to bind CD55 [http://www.ncbi.nlm.nih.gov/pubmed/9064337?dopt=AbstractPlus], chondroitin sulfate B [http://www.ncbi.nlm.nih.gov/pubmed/12829604?dopt=AbstractPlus], α_5_β_1_ and αγβ_3_ integrins [http://www.ncbi.nlm.nih.gov/pubmed/15576472?dopt=AbstractPlus], and CD90 [http://www.ncbi.nlm.nih.gov/pubmed/22210915?dopt=AbstractPlus].

**Table:** 

Nomenclature	http://www.guidetopharmacology.org/GRAC/ObjectDisplayForward?objectId=190	http://www.guidetopharmacology.org/GRAC/ObjectDisplayForward?objectId=191	http://www.guidetopharmacology.org/GRAC/ObjectDisplayForward?objectId=193	http://www.guidetopharmacology.org/GRAC/ObjectDisplayForward?objectId=195	http://www.guidetopharmacology.org/GRAC/ObjectDisplayForward?objectId=196
HGNC, UniProt	https://www.genenames.org/data/gene‐symbol‐report/#!/hgnc_id/HGNC:18990, http://www.uniprot.org/uniprot/Q5T601	https://www.genenames.org/data/gene‐symbol‐report/#!/hgnc_id/HGNC:18991, http://www.uniprot.org/uniprot/Q8IZF7	https://www.genenames.org/data/gene‐symbol‐report/#!/hgnc_id/HGNC:18989, http://www.uniprot.org/uniprot/Q8IZF5	https://www.genenames.org/data/gene‐symbol‐report/#!/hgnc_id/HGNC:19011, http://www.uniprot.org/uniprot/Q8IZF3	https://www.genenames.org/data/gene‐symbol‐report/#!/hgnc_id/HGNC:19030, http://www.uniprot.org/uniprot/Q8IZF2
Comments	Synaptamide is an agonist at ADGRF1 supporting neurogenesis [http://www.ncbi.nlm.nih.gov/pubmed/27759003?dopt=AbstractPlus] and couples to Gs and Gq pathways [http://www.ncbi.nlm.nih.gov/pubmed/28154189?dopt=AbstractPlus, http://www.ncbi.nlm.nih.gov/pubmed/25918380?dopt=AbstractPlus].	ADGRF2 is highly expressed in squamous epithelia and gene deficiency did not result in detectable defects [http://www.ncbi.nlm.nih.gov/pubmed/22837050?dopt=AbstractPlus].	ADGRF3 is highly expressed in gastrointestinal neuroendocrine tumors [http://www.ncbi.nlm.nih.gov/pubmed/23158174?dopt=AbstractPlus].	ADGRF4 couples to G_q/11_ proteins [http://www.ncbi.nlm.nih.gov/pubmed/28154189?dopt=AbstractPlus], is highly expressed in squamous epithelia and gene deficiency did not result in detectable defects [http://www.ncbi.nlm.nih.gov/pubmed/22837050?dopt=AbstractPlus].	ADGRF5 controls alveolar surfactant secretion via G_q/11_ pathway [http://www.ncbi.nlm.nih.gov/pubmed/28570277?dopt=AbstractPlus].

**Table:** 

Nomenclature	http://www.guidetopharmacology.org/GRAC/ObjectDisplayForward?objectId=186	http://www.guidetopharmacology.org/GRAC/ObjectDisplayForward?objectId=187	http://www.guidetopharmacology.org/GRAC/ObjectDisplayForward?objectId=188	http://www.guidetopharmacology.org/GRAC/ObjectDisplayForward?objectId=192	http://www.guidetopharmacology.org/GRAC/ObjectDisplayForward?objectId=194	http://www.guidetopharmacology.org/GRAC/ObjectDisplayForward?objectId=200	http://www.guidetopharmacology.org/GRAC/ObjectDisplayForward?objectId=201
HGNC, UniProt	https://www.genenames.org/data/gene‐symbol‐report/#!/hgnc_id/HGNC:4512, http://www.uniprot.org/uniprot/Q9Y653	https://www.genenames.org/data/gene‐symbol‐report/#!/hgnc_id/HGNC:4516, http://www.uniprot.org/uniprot/Q8IZP9	https://www.genenames.org/data/gene‐symbol‐report/#!/hgnc_id/HGNC:13728, http://www.uniprot.org/uniprot/Q86Y34	https://www.genenames.org/data/gene‐symbol‐report/#!/hgnc_id/HGNC:18992, http://www.uniprot.org/uniprot/Q8IZF6	https://www.genenames.org/data/gene‐symbol‐report/#!/hgnc_id/HGNC:19010, http://www.uniprot.org/uniprot/Q8IZF4	https://www.genenames.org/data/gene‐symbol‐report/#!/hgnc_id/HGNC:13841, http://www.uniprot.org/uniprot/Q86SQ4	https://www.genenames.org/data/gene‐symbol‐report/#!/hgnc_id/HGNC:19241, http://www.uniprot.org/uniprot/Q96K78
Comments	Reported to bind tissue transglutaminase 2 [http://www.ncbi.nlm.nih.gov/pubmed/16757564?dopt=AbstractPlus] and collagen, which activates the G_12/13_ pathway [http://www.ncbi.nlm.nih.gov/pubmed/21768377?dopt=AbstractPlus].	ADGRG2 is coupled to G_q_ and G_s_ pathways [http://www.ncbi.nlm.nih.gov/pubmed/26188515?dopt=AbstractPlus] and gene deficiency causes congenital obstructive azoospermia [http://www.ncbi.nlm.nih.gov/pubmed/27476656?dopt=AbstractPlus].	ADGRG3 is expressed in immune cells [http://www.ncbi.nlm.nih.gov/pubmed/27089991?dopt=AbstractPlus, http://www.ncbi.nlm.nih.gov/pubmed/24113187?dopt=AbstractPlus] and couples to G_o_ proteins [http://www.ncbi.nlm.nih.gov/pubmed/22575658?dopt=AbstractPlus].	ADGRG4 is highly expressed in enterochromaffin cells and gastrointestinal neuroendocrine tumors [http://www.ncbi.nlm.nih.gov/pubmed/18953328?dopt=AbstractPlus].	ADGRG5 is a constitutively active G_s_ protein‐coupled receptor [http://www.ncbi.nlm.nih.gov/pubmed/22575658?dopt=AbstractPlus, http://www.ncbi.nlm.nih.gov/pubmed/26499266?dopt=AbstractPlus], highly expressed in eosinophils and NK cells [http://www.ncbi.nlm.nih.gov/pubmed/21724806?dopt=AbstractPlus].	ADGRG6 is a key regulator of Schwann cell‐mediated myelination [http://www.ncbi.nlm.nih.gov/pubmed/19745155?dopt=AbstractPlus], and couples to G_s_ and G_i/o_ pathways [http://www.ncbi.nlm.nih.gov/pubmed/24227709?dopt=AbstractPlus].	ADGRG7 is expressed in interstine and involved in interstine contractility regulation [http://www.ncbi.nlm.nih.gov/pubmed/24574718?dopt=AbstractPlus].

**Table:** 

Nomenclature	http://www.guidetopharmacology.org/GRAC/ObjectDisplayForward?objectId=206	http://www.guidetopharmacology.org/GRAC/ObjectDisplayForward?objectId=207	http://www.guidetopharmacology.org/GRAC/ObjectDisplayForward?objectId=208	http://www.guidetopharmacology.org/GRAC/ObjectDisplayForward?objectId=181	http://www.guidetopharmacology.org/GRAC/ObjectDisplayForward?objectId=189
HGNC, UniProt	https://www.genenames.org/data/gene‐symbol‐report/#!/hgnc_id/HGNC:20973, http://www.uniprot.org/uniprot/O94910	https://www.genenames.org/data/gene‐symbol‐report/#!/hgnc_id/HGNC:18582, http://www.uniprot.org/uniprot/O95490	https://www.genenames.org/data/gene‐symbol‐report/#!/hgnc_id/HGNC:20974, http://www.uniprot.org/uniprot/Q9HAR2	https://www.genenames.org/data/gene‐symbol‐report/#!/hgnc_id/HGNC:20822, http://www.uniprot.org/uniprot/Q9HBW9	https://www.genenames.org/data/gene‐symbol‐report/#!/hgnc_id/HGNC:17416, http://www.uniprot.org/uniprot/Q8WXG9
Comments	Couples to G_s_ and G_q_ pathways [http://www.ncbi.nlm.nih.gov/pubmed/9261169?dopt=AbstractPlus, http://www.ncbi.nlm.nih.gov/pubmed/26505631?dopt=AbstractPlus].	–	A LPHN3 gene variant in humans is associated with attention‐deficit‐hyperactivity disorder [http://www.ncbi.nlm.nih.gov/pubmed/20157310?dopt=AbstractPlus, http://www.ncbi.nlm.nih.gov/pubmed/28472652?dopt=AbstractPlus].	–	Loss‐of‐function mutations are associated with Usher syndrome, a sensory deficit disorder [http://www.ncbi.nlm.nih.gov/pubmed/18463160?dopt=AbstractPlus].

### Further reading on Adhesion Class GPCRs

Hamann J et al. (2015) International Union of Basic and Clinical Pharmacology. XCIV. Adhesion G protein‐coupled receptors. *Pharmacol Rev* **67**: 338‐67 [https://www.ncbi.nlm.nih.gov/pubmed/25713288?dopt=AbstractPlus]

Langenhan T et al. (2013) Sticky signaling–adhesion class G protein‐coupled receptors take the stage. *Sci Signal* **6**: re3 [https://www.ncbi.nlm.nih.gov/pubmed/23695165?dopt=AbstractPlus]

Liebscher I et al. (2016) Tethered Agonism: A Common Activation Mechanism of Adhesion GPCRs. *Handb Exp Pharmacol* **234**: 111‐125 [https://www.ncbi.nlm.nih.gov/pubmed/27832486?dopt=AbstractPlus]

Monk KR et al. (2015) Adhesion G Protein‐Coupled Receptors: From In Vitro Pharmacology to In Vivo Mechanisms. *Mol Pharmacol* **88**: 617‐23 [https://www.ncbi.nlm.nih.gov/pubmed/25956432?dopt=AbstractPlus]

Purcell RH et al. (2018) Adhesion G Protein‐Coupled Receptors as Drug Targets. *Annu Rev Pharmacol Toxicol* **58**: 429‐449 [https://www.ncbi.nlm.nih.gov/pubmed/28968187?dopt=AbstractPlus]

## http://www.guidetopharmacology.org/GRAC/FamilyDisplayForward?familyId=4

### Overview

**The nomenclature of the Adrenoceptors has been agreed by the** NC‐IUPHAR Subcommittee on Adrenoceptors [http://www.ncbi.nlm.nih.gov/pubmed/7938162?dopt=AbstractPlus], **see also** [http://www.ncbi.nlm.nih.gov/pubmed/7568329?dopt=AbstractPlus].

### **Adrenoceptors,** α_**1**_

α_1_‐Adrenoceptors are activated by the endogenous agonists (‐)‐http://www.guidetopharmacology.org/GRAC/LigandDisplayForward?ligandId=479 and (‐)‐http://www.guidetopharmacology.org/GRAC/LigandDisplayForward?ligandId=505. http://www.guidetopharmacology.org/GRAC/LigandDisplayForward?ligandId=485, http://www.guidetopharmacology.org/GRAC/LigandDisplayForward?ligandId=483 and http://www.guidetopharmacology.org/GRAC/LigandDisplayForward?ligandId=515 are agonists and http://www.guidetopharmacology.org/GRAC/LigandDisplayForward?ligandId=503 and http://www.guidetopharmacology.org/GRAC/LigandDisplayForward?ligandId=515 antagonists considered selective for α_1_‐ relative to α_2_‐adrenoceptors. [http://www.guidetopharmacology.org/GRAC/LigandDisplayForward?ligandId=5385 and [http://www.guidetopharmacology.org/GRAC/LigandDisplayForward?ligandId=482 (BE2254) are relatively selective radioligands. http://www.guidetopharmacology.org/GRAC/LigandDisplayForward?ligandId=487 also has high affinity for L‐type Ca^2+^ channels. Fluorescent derivatives of http://www.guidetopharmacology.org/GRAC/LigandDisplayForward?ligandId=503 (Bodipy PLprazosin‐ QAPB) are used to examine cellular localisation of α_1_‐adrenoceptors. Selective α_1_‐adrenoceptor agonists are used as nasal decongestants; antagonists to treat hypertension (http://www.guidetopharmacology.org/GRAC/LigandDisplayForward?ligandId=7170, http://www.guidetopharmacology.org/GRAC/LigandDisplayForward?ligandId=7170) and benign prostatic hyperplasia (http://www.guidetopharmacology.org/GRAC/LigandDisplayForward?ligandId=7109, http://www.guidetopharmacology.org/GRAC/LigandDisplayForward?ligandId=488). The α_1_‐ and β_2_‐adrenoceptor antagonist http://www.guidetopharmacology.org/GRAC/LigandDisplayForward?ligandId=551 is used to treat congestive heart failure, although the contribution of α_1_‐adrenoceptor blockade to the therapeutic effect is unclear. Several anti‐depressants and anti‐psychotic drugs are α_1_‐adrenoceptor antagonists contributing to side effects such as orthostatic hypotension and extrapyramidal effects.

**Table:** 

Nomenclature	http://www.guidetopharmacology.org/GRAC/ObjectDisplayForward?objectId=22	http://www.guidetopharmacology.org/GRAC/ObjectDisplayForward?objectId=23	http://www.guidetopharmacology.org/GRAC/ObjectDisplayForward?objectId=24
HGNC, UniProt	https://www.genenames.org/data/gene‐symbol‐report/#!/hgnc_id/HGNC:277, http://www.uniprot.org/uniprot/P35348	https://www.genenames.org/data/gene‐symbol‐report/#!/hgnc_id/HGNC:278, http://www.uniprot.org/uniprot/P35368	https://www.genenames.org/data/gene‐symbol‐report/#!/hgnc_id/HGNC:280, http://www.uniprot.org/uniprot/P25100
Endogenous agonists	(‐)‐http://www.guidetopharmacology.org/GRAC/LigandDisplayForward?ligandId=479 [http://www.ncbi.nlm.nih.gov/pubmed/8564227?dopt=AbstractPlus, http://www.ncbi.nlm.nih.gov/pubmed/7651358?dopt=AbstractPlus], (‐)‐http://www.guidetopharmacology.org/GRAC/LigandDisplayForward?ligandId=505, http://www.ncbi.nlm.nih.gov/pubmed/7651358?dopt=AbstractPlus, http://www.ncbi.nlm.nih.gov/pubmed/10433504?dopt=AbstractPlus]	–	(‐)‐http://www.guidetopharmacology.org/GRAC/LigandDisplayForward?ligandId=505 [http://www.ncbi.nlm.nih.gov/pubmed/8564227?dopt=AbstractPlus, http://www.ncbi.nlm.nih.gov/pubmed/7651358?dopt=AbstractPlus], (‐)‐http://www.guidetopharmacology.org/GRAC/LigandDisplayForward?ligandId=479, http://www.ncbi.nlm.nih.gov/pubmed/7651358?dopt=AbstractPlus]
Agonists	http://www.guidetopharmacology.org/GRAC/LigandDisplayForward?ligandId=124 [http://www.ncbi.nlm.nih.gov/pubmed/8564227?dopt=AbstractPlus, http://www.ncbi.nlm.nih.gov/pubmed/8719417?dopt=AbstractPlus, http://www.ncbi.nlm.nih.gov/pubmed/7651358?dopt=AbstractPlus, http://www.ncbi.nlm.nih.gov/pubmed/10433504?dopt=AbstractPlus], http://www.guidetopharmacology.org/GRAC/LigandDisplayForward?ligandId=485 [http://www.ncbi.nlm.nih.gov/pubmed/10433504?dopt=AbstractPlus], http://www.guidetopharmacology.org/GRAC/LigandDisplayForward?ligandId=483[http://www.ncbi.nlm.nih.gov/pubmed/7651358?dopt=AbstractPlus, http://www.ncbi.nlm.nih.gov/pubmed/10433504?dopt=AbstractPlus]	http://www.guidetopharmacology.org/GRAC/LigandDisplayForward?ligandId=485 [http://www.ncbi.nlm.nih.gov/pubmed/9249248?dopt=AbstractPlus, http://www.ncbi.nlm.nih.gov/pubmed/7969082?dopt=AbstractPlus]	–
Selective agonists	http://www.guidetopharmacology.org/GRAC/LigandDisplayForward?ligandId=480 [http://www.ncbi.nlm.nih.gov/pubmed/9249248?dopt=AbstractPlus, http://www.ncbi.nlm.nih.gov/pubmed/7616455?dopt=AbstractPlus], http://www.guidetopharmacology.org/GRAC/LigandDisplayForward?ligandId=3469 [http://www.ncbi.nlm.nih.gov/pubmed/14678390?dopt=AbstractPlus]	–	–
Antagonists	http://www.guidetopharmacology.org/GRAC/LigandDisplayForward?ligandId=503 (Inverse agonist) (p*K*_i_ 9–9.9) [http://www.ncbi.nlm.nih.gov/pubmed/9490024?dopt=AbstractPlus, http://www.ncbi.nlm.nih.gov/pubmed/10334511?dopt=AbstractPlus, http://www.ncbi.nlm.nih.gov/pubmed/9249248?dopt=AbstractPlus, http://www.ncbi.nlm.nih.gov/pubmed/7651358?dopt=AbstractPlus, http://www.ncbi.nlm.nih.gov/pubmed/10369480?dopt=AbstractPlus], http://www.guidetopharmacology.org/GRAC/LigandDisplayForward?ligandId=7170 (p*K*_i_ 9.3) [0795], http://www.guidetopharmacology.org/GRAC/LigandDisplayForward?ligandId=7302 (p*K*_i_ 8.7) [http://www.ncbi.nlm.nih.gov/pubmed/9379432?dopt=AbstractPlus], http://www.guidetopharmacology.org/GRAC/LigandDisplayForward?ligandId=502 (p*K*_i_ 8.6) [http://www.ncbi.nlm.nih.gov/pubmed/7651358?dopt=AbstractPlus], http://www.guidetopharmacology.org/GRAC/LigandDisplayForward?ligandId=7109 (p*K*_i_ 8.1) [http://www.ncbi.nlm.nih.gov/pubmed/10812954?dopt=AbstractPlus]	http://www.guidetopharmacology.org/GRAC/LigandDisplayForward?ligandId=503 (Inverse agonist) (p*K*_i_ 9.6–9.9) [http://www.ncbi.nlm.nih.gov/pubmed/9249248?dopt=AbstractPlus, http://www.ncbi.nlm.nih.gov/pubmed/7651358?dopt=AbstractPlus, http://www.ncbi.nlm.nih.gov/pubmed/10369480?dopt=AbstractPlus], http://www.guidetopharmacology.org/GRAC/LigandDisplayForward?ligandId=488 (Inverse agonist) (p*K*_i_ 9.5–9.7) [http://www.ncbi.nlm.nih.gov/pubmed/9249248?dopt=AbstractPlus, http://www.ncbi.nlm.nih.gov/pubmed/10369480?dopt=AbstractPlus], http://www.guidetopharmacology.org/GRAC/LigandDisplayForward?ligandId=7170 (p*K*_i_ 9.1) [0795], http://www.guidetopharmacology.org/GRAC/LigandDisplayForward?ligandId=7109 (p*K*_i_ 8.6) [http://www.ncbi.nlm.nih.gov/pubmed/7658428?dopt=AbstractPlus], http://www.guidetopharmacology.org/GRAC/LigandDisplayForward?ligandId=7302 (p*K*_i_ 8.6) [http://www.ncbi.nlm.nih.gov/pubmed/9379432?dopt=AbstractPlus], http://www.guidetopharmacology.org/GRAC/LigandDisplayForward?ligandId=502 (p*K*_i_ 7.5) [http://www.ncbi.nlm.nih.gov/pubmed/7651358?dopt=AbstractPlus]	http://www.guidetopharmacology.org/GRAC/LigandDisplayForward?ligandId=503 (Inverse agonist) (p*K*_i_ 9.5–10.2) [http://www.ncbi.nlm.nih.gov/pubmed/9249248?dopt=AbstractPlus, http://www.ncbi.nlm.nih.gov/pubmed/7651358?dopt=AbstractPlus, http://www.ncbi.nlm.nih.gov/pubmed/10369480?dopt=AbstractPlus], http://www.guidetopharmacology.org/GRAC/LigandDisplayForward?ligandId=488 (p*K*_i_ 9.8–10.2) [http://www.ncbi.nlm.nih.gov/pubmed/9249248?dopt=AbstractPlus, http://www.ncbi.nlm.nih.gov/pubmed/10369480?dopt=AbstractPlus], http://www.guidetopharmacology.org/GRAC/LigandDisplayForward?ligandId=7170 (p*K*i 9.1) [0795], http://www.guidetopharmacology.org/GRAC/LigandDisplayForward?ligandId=7302 (p*K*_i_ 9.1) [http://www.ncbi.nlm.nih.gov/pubmed/9379432?dopt=AbstractPlus], http://www.guidetopharmacology.org/GRAC/LigandDisplayForward?ligandId=7109 (p*K*_i_ 8.4) [http://www.ncbi.nlm.nih.gov/pubmed/10812954?dopt=AbstractPlus], http://www.guidetopharmacology.org/GRAC/LigandDisplayForward?ligandId=7155 (p*K*_i_ 8.4) [0079], http://www.guidetopharmacology.org/GRAC/LigandDisplayForward?ligandId=502 (Inverse agonist) (p*K*_i_ 8.2) [http://www.ncbi.nlm.nih.gov/pubmed/7651358?dopt=AbstractPlus], http://www.guidetopharmacology.org/GRAC/LigandDisplayForward?ligandId=7381 (p*K*_i_ 7.9) [http://www.ncbi.nlm.nih.gov/pubmed/10369480?dopt=AbstractPlus], http://www.guidetopharmacology.org/GRAC/LigandDisplayForward?ligandId=7207 (p*K*_i_ 6.6) [0079]
Selective antagonists	http://www.guidetopharmacology.org/GRAC/LigandDisplayForward?ligandId=488 (p*K*_i_ 10–10.7) [http://www.ncbi.nlm.nih.gov/pubmed/9490024?dopt=AbstractPlus, http://www.ncbi.nlm.nih.gov/pubmed/10334511?dopt=AbstractPlus, http://www.ncbi.nlm.nih.gov/pubmed/9249248?dopt=AbstractPlus, http://www.ncbi.nlm.nih.gov/pubmed/7651358?dopt=AbstractPlus, http://www.ncbi.nlm.nih.gov/pubmed/10369480?dopt=AbstractPlus], http://www.guidetopharmacology.org/GRAC/LigandDisplayForward?ligandId=493 (p*K*_i_ 10.4) [http://www.ncbi.nlm.nih.gov/pubmed/7651358?dopt=AbstractPlus], http://www.guidetopharmacology.org/GRAC/LigandDisplayForward?ligandId=487 (p*K*_i_ 9.1–10) [http://www.ncbi.nlm.nih.gov/pubmed/9249248?dopt=AbstractPlus, http://www.ncbi.nlm.nih.gov/pubmed/7651358?dopt=AbstractPlus], http://www.guidetopharmacology.org/GRAC/LigandDisplayForward?ligandId=7381 (p*K*_i_ 9.6) [http://www.ncbi.nlm.nih.gov/pubmed/10369480?dopt=AbstractPlus], http://www.guidetopharmacology.org/GRAC/LigandDisplayForward?ligandId=498 (p*K*_i_ 8.8–9.4) [http://www.ncbi.nlm.nih.gov/pubmed/10812954?dopt=AbstractPlus, http://www.ncbi.nlm.nih.gov/pubmed/9190863?dopt=AbstractPlus, http://www.ncbi.nlm.nih.gov/pubmed/7752182?dopt=AbstractPlus], http://www.guidetopharmacology.org/GRAC/LigandDisplayForward?ligandId=6663‐http://www.guidetopharmacology.org/GRAC/LigandDisplayForward?ligandId=6663 (p*K*_i_ 9.2–9.3) [http://www.ncbi.nlm.nih.gov/pubmed/23935897?dopt=AbstractPlus, http://www.ncbi.nlm.nih.gov/pubmed/20015090?dopt=AbstractPlus], http://www.guidetopharmacology.org/GRAC/LigandDisplayForward?ligandId=495 (p*K*_i_ 9.2–9.3) [http://www.ncbi.nlm.nih.gov/pubmed/9490024?dopt=AbstractPlus, http://www.ncbi.nlm.nih.gov/pubmed/9490024?dopt=AbstractPlus, http://www.ncbi.nlm.nih.gov/pubmed/8632751?dopt=AbstractPlus, http://www.ncbi.nlm.nih.gov/pubmed/9249248?dopt=AbstractPlus]	http://www.guidetopharmacology.org/GRAC/LigandDisplayForward?ligandId=8459 (p*K*_i_ 9.5) [http://www.ncbi.nlm.nih.gov/pubmed/9190864?dopt=AbstractPlus], http://www.guidetopharmacology.org/GRAC/LigandDisplayForward?ligandId=506 (p*K*_i_ 7.7) [http://www.ncbi.nlm.nih.gov/pubmed/9548811?dopt=AbstractPlus], http://www.guidetopharmacology.org/GRAC/LigandDisplayForward?ligandId=8461 (p*K*_i_ 7.5) [http://www.ncbi.nlm.nih.gov/pubmed/8764344?dopt=AbstractPlus]	http://www.guidetopharmacology.org/GRAC/LigandDisplayForward?ligandId=9 (p*K*_i_ 8.7–9.1) [http://www.ncbi.nlm.nih.gov/pubmed/11354357?dopt=AbstractPlus, http://www.ncbi.nlm.nih.gov/pubmed/11459121?dopt=AbstractPlus]

### Comments

The α_1C_‐adrenoceptor corresponds to the pharmacologically defined α_1A_‐adrenoceptor [http://www.ncbi.nlm.nih.gov/pubmed/7568329?dopt=AbstractPlus]. Some tissues possess α_1A_‐adrenoceptors (α_1L_‐adrenoceptors [http://www.ncbi.nlm.nih.gov/pubmed/9249248?dopt=AbstractPlus, http://www.ncbi.nlm.nih.gov/pubmed/17162094?dopt=AbstractPlus]) that display relatively low affinity in functional and binding assays for http://www.guidetopharmacology.org/GRAC/LigandDisplayForward?ligandId=503 indicative of different receptor states or locations. α1A‐adrenoceptor C‐terminal splice variants form homo‐ and heterodimers, but fail to generate a functional α_1L_‐adrenoceptor [http://www.ncbi.nlm.nih.gov/pubmed/15266013?dopt=AbstractPlus]. α_1D_‐Adrenoceptors form heterodimers with α_1B_‐ or β_2_‐adrenoceptors that show increased cell‐surface expression [http://www.ncbi.nlm.nih.gov/pubmed/15615865?dopt=AbstractPlus]. Recombinant α_1D_‐adrenoceptors have been shown in some heterologous systems to be mainly located intracellularly but cell‐surface localization is encouraged by truncation of the Nterminus, or by co‐expression of α_1B_‐ or β_2_‐adrenoceptors [http://www.ncbi.nlm.nih.gov/pubmed/14718583?dopt=AbstractPlus, http://www.ncbi.nlm.nih.gov/pubmed/14718583?dopt=AbstractPlus]. In blood vessels all three α_1_‐adrenoceptor subtypes are located on the surface and intracellularly [http://www.ncbi.nlm.nih.gov/pubmed/19572943?dopt=AbstractPlus, http://www.ncbi.nlm.nih.gov/pubmed/19888965?dopt=AbstractPlus]. Signalling is predominantly via G_q/11_ butα1‐adrenoceptors also couple toG_i/o_, G_s_ and G_12/13_. Several α_1A_‐adrenoceptor agonists display ligand directed signalling bias relative to noradrenaline [http://www.ncbi.nlm.nih.gov/pubmed/20978120?dopt=AbstractPlus]. There are also differences between subtypes in coupling efficiency to different pathways. In vascular smooth muscle, the potency of agonists is related to the predominant subtype, α_1D_‐ conveying greater agonist sensitivity than α_1A_‐adrenoceptors [http://www.ncbi.nlm.nih.gov/pubmed/23373597?dopt=AbstractPlus].

### **Adrenoceptors,** α_**2**_

α_2_‐Adrenoceptors are activated by (‐)‐http://www.guidetopharmacology.org/GRAC/LigandDisplayForward?ligandId=479 and with lower potency by (‐)‐http://www.guidetopharmacology.org/GRAC/LigandDisplayForward?ligandId=505. http://www.guidetopharmacology.org/GRAC/LigandDisplayForward?ligandId=520 and http://www.guidetopharmacology.org/GRAC/LigandDisplayForward?ligandId=5442 are agonists and http://www.guidetopharmacology.org/GRAC/LigandDisplayForward?ligandId=136 and http://www.guidetopharmacology.org/GRAC/LigandDisplayForward?ligandId=102 antagonists selective for α_2_‐ relative to α_1_‐adrenoceptors. [http://www.guidetopharmacology.org/GRAC/LigandDisplayForward?ligandId=223, [http://www.guidetopharmacology.org/GRAC/LigandDisplayForward?ligandId=5386 and [http://www.guidetopharmacology.org/GRAC/LigandDisplayForward?ligandId=528 are relatively selective radioligands. There is species variation in the pharmacology of the α_2A_‐adrenoceptor. Multiple mutations of α_2_‐adrenoceptors have been described, some associated with alterations in function. Presynaptic α_2_‐adrenoceptors regulate many functions in the nervous system. The α_2_‐adrenoceptor agonists http://www.guidetopharmacology.org/GRAC/LigandDisplayForward?ligandId=516, http://www.guidetopharmacology.org/GRAC/LigandDisplayForward?ligandId=5443 and http://www.guidetopharmacology.org/GRAC/LigandDisplayForward?ligandId=520 affect central baroreflex control (hypotension and bradycardia), induce hypnotic effects and analgesia, and modulate seizure activity and platelet aggregation. http://www.guidetopharmacology.org/GRAC/LigandDisplayForward?ligandId=516 is an anti‐hypertensive and counteracts opioid withdrawal. http://www.guidetopharmacology.org/GRAC/LigandDisplayForward?ligandId=521 (also http://www.guidetopharmacology.org/GRAC/LigandDisplayForward?ligandId=523) is used as a sedative and analgesic in human and veterinary medicine with sympatholytic and anxiolytic properties. The α_2_‐adrenoceptor antagonist http://www.guidetopharmacology.org/GRAC/LigandDisplayForward?ligandId=102 has been used to treat erectile dysfunction and http://www.guidetopharmacology.org/GRAC/LigandDisplayForward?ligandId=7241 as an anti‐depressant. The α_2B_ subtype appears to be involved in neurotransmission in the spinal cord and α_2C_ in regulating catecholamine release from adrenal chromaffin cells.

**Table:** 

Nomenclature	http://www.guidetopharmacology.org/GRAC/ObjectDisplayForward?objectId=25	http://www.guidetopharmacology.org/GRAC/ObjectDisplayForward?objectId=26	http://www.guidetopharmacology.org/GRAC/ObjectDisplayForward?objectId=27
HGNC, UniProt	https://www.genenames.org/data/gene‐symbol‐report/#!/hgnc_id/HGNC:281, http://www.uniprot.org/uniprot/P08913	https://www.genenames.org/data/gene‐symbol‐report/#!/hgnc_id/HGNC:282, http://www.uniprot.org/uniprot/P18089	https://www.genenames.org/data/gene‐symbol‐report/#!/hgnc_id/HGNC:283, http://www.uniprot.org/uniprot/P18825
Endogenous agonists	(‐)‐http://www.guidetopharmacology.org/GRAC/LigandDisplayForward?ligandId=479 [http://www.ncbi.nlm.nih.gov/pubmed/9605427?dopt=AbstractPlus, http://www.ncbi.nlm.nih.gov/pubmed/9824686?dopt=AbstractPlus], (‐)‐http://www.guidetopharmacology.org/GRAC/LigandDisplayForward?ligandId=505, http://www.ncbi.nlm.nih.gov/pubmed/9824686?dopt=AbstractPlus]	(‐)‐http://www.guidetopharmacology.org/GRAC/LigandDisplayForward?ligandId=505 (Partial agonist) [http://www.ncbi.nlm.nih.gov/pubmed/9605427?dopt=AbstractPlus, http://www.ncbi.nlm.nih.gov/pubmed/9824686?dopt=AbstractPlus], (‐)‐http://www.guidetopharmacology.org/GRAC/LigandDisplayForward?ligandId=479[http://www.ncbi.nlm.nih.gov/pubmed/9605427?dopt=AbstractPlus]	(‐)‐http://www.guidetopharmacology.org/GRAC/LigandDisplayForward?ligandId=505 [http://www.ncbi.nlm.nih.gov/pubmed/9605427?dopt=AbstractPlus, http://www.ncbi.nlm.nih.gov/pubmed/9824686?dopt=AbstractPlus], (‐)‐http://www.guidetopharmacology.org/GRAC/LigandDisplayForward?ligandId=479]
Agonists	http://www.guidetopharmacology.org/GRAC/LigandDisplayForward?ligandId=521 (Partial agonist) [http://www.ncbi.nlm.nih.gov/pubmed/9605427?dopt=AbstractPlus, http://www.ncbi.nlm.nih.gov/pubmed/9227000?dopt=AbstractPlus, http://www.ncbi.nlm.nih.gov/pubmed/9760042?dopt=AbstractPlus, http://www.ncbi.nlm.nih.gov/pubmed/9824686?dopt=AbstractPlus], http://www.guidetopharmacology.org/GRAC/LigandDisplayForward?ligandId=516 (Partial agonist) [http://www.ncbi.nlm.nih.gov/pubmed/9605427?dopt=AbstractPlus, http://www.ncbi.nlm.nih.gov/pubmed/9760042?dopt=AbstractPlus, http://www.ncbi.nlm.nih.gov/pubmed/9824686?dopt=AbstractPlus], http://www.guidetopharmacology.org/GRAC/LigandDisplayForward?ligandId=520[http://www.ncbi.nlm.nih.gov/pubmed/9605427?dopt=AbstractPlus, http://www.ncbi.nlm.nih.gov/pubmed/9227000?dopt=AbstractPlus, http://www.ncbi.nlm.nih.gov/pubmed/9760042?dopt=AbstractPlus, http://www.ncbi.nlm.nih.gov/pubmed/9824686?dopt=AbstractPlus], http://www.guidetopharmacology.org/GRAC/LigandDisplayForward?ligandId=7117 [http://www.ncbi.nlm.nih.gov/pubmed/8784451?dopt=AbstractPlus], http://www.guidetopharmacology.org/GRAC/LigandDisplayForward?ligandId=5443 [0079], http://www.guidetopharmacology.org/GRAC/LigandDisplayForward?ligandId=522 (Partial agonist) [http://www.ncbi.nlm.nih.gov/pubmed/9605427?dopt=AbstractPlus, http://www.ncbi.nlm.nih.gov/pubmed/9283709?dopt=AbstractPlus]	http://www.guidetopharmacology.org/GRAC/LigandDisplayForward?ligandId=521 [http://www.ncbi.nlm.nih.gov/pubmed/9605427?dopt=AbstractPlus, http://www.ncbi.nlm.nih.gov/pubmed/9227000?dopt=AbstractPlus, http://www.ncbi.nlm.nih.gov/pubmed/9760042?dopt=AbstractPlus, http://www.ncbi.nlm.nih.gov/pubmed/9824686?dopt=AbstractPlus], http://www.guidetopharmacology.org/GRAC/LigandDisplayForward?ligandId=516 (Partial agonist) [http://www.ncbi.nlm.nih.gov/pubmed/9605427?dopt=AbstractPlus, http://www.ncbi.nlm.nih.gov/pubmed/9760042?dopt=AbstractPlus, http://www.ncbi.nlm.nih.gov/pubmed/9824686?dopt=AbstractPlus], http://www.guidetopharmacology.org/GRAC/LigandDisplayForward?ligandId=520 (Partial agonist) [http://www.ncbi.nlm.nih.gov/pubmed/9605427?dopt=AbstractPlus, http://www.ncbi.nlm.nih.gov/pubmed/9760042?dopt=AbstractPlus, http://www.ncbi.nlm.nih.gov/pubmed/9824686?dopt=AbstractPlus], http://www.guidetopharmacology.org/GRAC/LigandDisplayForward?ligandId=5443 [0079], http://www.guidetopharmacology.org/GRAC/LigandDisplayForward?ligandId=522[http://www.ncbi.nlm.nih.gov/pubmed/9605427?dopt=AbstractPlus]	http://www.guidetopharmacology.org/GRAC/LigandDisplayForward?ligandId=521 [http://www.ncbi.nlm.nih.gov/pubmed/9605427?dopt=AbstractPlus, http://www.ncbi.nlm.nih.gov/pubmed/9760042?dopt=AbstractPlus, http://www.ncbi.nlm.nih.gov/pubmed/9824686?dopt=AbstractPlus], http://www.guidetopharmacology.org/GRAC/LigandDisplayForward?ligandId=520 (Partial agonist) [http://www.ncbi.nlm.nih.gov/pubmed/9605427?dopt=AbstractPlus, http://www.ncbi.nlm.nih.gov/pubmed/9227000?dopt=AbstractPlus, http://www.ncbi.nlm.nih.gov/pubmed/9824686?dopt=AbstractPlus], http://www.guidetopharmacology.org/GRAC/LigandDisplayForward?ligandId=7117 [http://www.ncbi.nlm.nih.gov/pubmed/8784451?dopt=AbstractPlus], http://www.guidetopharmacology.org/GRAC/LigandDisplayForward?ligandId=522 (Partial agonist) [http://www.ncbi.nlm.nih.gov/pubmed/9605427?dopt=AbstractPlus], http://www.guidetopharmacology.org/GRAC/LigandDisplayForward?ligandId=5443 [0079]
Selective agonists	http://www.guidetopharmacology.org/GRAC/LigandDisplayForward?ligandId=124 (Partial agonist) [http://www.ncbi.nlm.nih.gov/pubmed/9605427?dopt=AbstractPlus, http://www.ncbi.nlm.nih.gov/pubmed/9227000?dopt=AbstractPlus, http://www.ncbi.nlm.nih.gov/pubmed/7996470?dopt=AbstractPlus]	–	–
Antagonists	http://www.guidetopharmacology.org/GRAC/LigandDisplayForward?ligandId=102 (p*K*_i_ 8.4–9.2) [http://www.ncbi.nlm.nih.gov/pubmed/1353247?dopt=AbstractPlus, http://www.ncbi.nlm.nih.gov/pubmed/7908642?dopt=AbstractPlus, http://www.ncbi.nlm.nih.gov/pubmed/7996470?dopt=AbstractPlus]	http://www.guidetopharmacology.org/GRAC/LigandDisplayForward?ligandId=102 (p*K*_i_ 7.9–8.9) [http://www.ncbi.nlm.nih.gov/pubmed/1353247?dopt=AbstractPlus, http://www.ncbi.nlm.nih.gov/pubmed/7908642?dopt=AbstractPlus, http://www.ncbi.nlm.nih.gov/pubmed/7996470?dopt=AbstractPlus], http://www.guidetopharmacology.org/GRAC/LigandDisplayForward?ligandId=7268 (p*K*_i_ 8.5) [http://www.ncbi.nlm.nih.gov/pubmed/1834671?dopt=AbstractPlus], http://www.guidetopharmacology.org/GRAC/LigandDisplayForward?ligandId=7310 (p*K*_i_ 5.5) [http://www.ncbi.nlm.nih.gov/pubmed/9605427?dopt=AbstractPlus]	http://www.guidetopharmacology.org/GRAC/LigandDisplayForward?ligandId=102 (p*K*_i_ 8.5–9.5) [http://www.ncbi.nlm.nih.gov/pubmed/1353247?dopt=AbstractPlus, http://www.ncbi.nlm.nih.gov/pubmed/7908642?dopt=AbstractPlus, http://www.ncbi.nlm.nih.gov/pubmed/7996470?dopt=AbstractPlus], http://www.guidetopharmacology.org/GRAC/LigandDisplayForward?ligandId=499 (p*K*i 8.4–9.4) [http://www.ncbi.nlm.nih.gov/pubmed/1353247?dopt=AbstractPlus, http://www.ncbi.nlm.nih.gov/pubmed/7996470?dopt=AbstractPlus], http://www.guidetopharmacology.org/GRAC/LigandDisplayForward?ligandId=53 (p*K*_i_ 9) [http://www.ncbi.nlm.nih.gov/pubmed/7996470?dopt=AbstractPlus], http://www.guidetopharmacology.org/GRAC/LigandDisplayForward?ligandId=7241 (p*K*_i_ 7.7) [http://www.ncbi.nlm.nih.gov/pubmed/15771415?dopt=AbstractPlus], http://www.guidetopharmacology.org/GRAC/LigandDisplayForward?ligandId=7310 (p*K*_i_ 5.4) [http://www.ncbi.nlm.nih.gov/pubmed/9605427?dopt=AbstractPlus]
Selective antagonists	http://www.guidetopharmacology.org/GRAC/LigandDisplayForward?ligandId=525 (p*K*_i_ 8.2–8.8) [http://www.ncbi.nlm.nih.gov/pubmed/7996470?dopt=AbstractPlus, http://www.ncbi.nlm.nih.gov/pubmed/2573535?dopt=AbstractPlus]	http://www.guidetopharmacology.org/GRAC/LigandDisplayForward?ligandId=3470 (p*K*_i_ 7.3) [http://www.ncbi.nlm.nih.gov/pubmed/1970500?dopt=AbstractPlus] – Rat	http://www.guidetopharmacology.org/GRAC/LigandDisplayForward?ligandId=3930 (p*K*_B_ 7.8) [http://www.ncbi.nlm.nih.gov/pubmed/17220913?dopt=AbstractPlus]
Labelled ligands	–	–	[http://www.guidetopharmacology.org/GRAC/LigandDisplayForward?ligandId=527 (Antagonist) (p*K*_d_ 10.1) [http://www.ncbi.nlm.nih.gov/pubmed/7996470?dopt=AbstractPlus]

### Comments

http://www.guidetopharmacology.org/GRAC/LigandDisplayForward?ligandId=524 and http://www.guidetopharmacology.org/GRAC/LigandDisplayForward?ligandId=503 show selectivity for α_2B_‐ and α_2C_‐adrenoceptors over α_2A_‐adrenoceptors.http://www.guidetopharmacology.org/GRAC/LigandDisplayForward?ligandId=124 is a reduced efficacy imidazoline agonist but also binds to non‐GPCR binding sites for imidazolines, classified as I_1_, I_2_ and I_3_ sites [http://www.ncbi.nlm.nih.gov/pubmed/15224384?dopt=AbstractPlus]; catecholamines have a low affinity, while rilmenidine and moxonidine are selective ligands evoking hypotensive effects *in vivo*. I_1_‐imidazoline receptors cause central inhibition of sympathetic tone, I_2_‐imidazoline receptors are an allosteric binding site on monoamine oxidase B, and I_3_‐imidazoline receptors regulate insulin secretion from pancreatic β‐cells. α_2A_‐adrenoceptor stimulation reduces insulin secretion from β‐islets [http://www.ncbi.nlm.nih.gov/pubmed/22645144?dopt=AbstractPlus], with a polymorphism in the 5’‐UTR of the ADRA2A gene being associated with increased receptor expression in β‐islets and heightened susceptibility to diabetes [http://www.ncbi.nlm.nih.gov/pubmed/19965390?dopt=AbstractPlus]. α_2A_‐ and α_2C_‐adrenoceptors form homodimers [http://www.ncbi.nlm.nih.gov/pubmed/16605244?dopt=AbstractPlus]. Heterodimers between α_2A_‐ and either the α_2C_‐adrenoceptor or *μ* opioid peptide receptor exhibit altered signalling and trafficking properties compared to the individual receptors [http://www.ncbi.nlm.nih.gov/pubmed/16605244?dopt=AbstractPlus, http://www.ncbi.nlm.nih.gov/pubmed/19126537?dopt=AbstractPlus, http://www.ncbi.nlm.nih.gov/pubmed/18193048?dopt=AbstractPlus]. Signalling by α_2_‐adrenoceptors is primarily via G_i/o_, although the α_2A_‐adrenoceptor also couples to Gs [http://www.ncbi.nlm.nih.gov/pubmed/7559592?dopt=AbstractPlus]. Imidazoline compounds display bias relative to each other at the α_2A_‐adrenoceptor [http://www.ncbi.nlm.nih.gov/pubmed/12649300?dopt=AbstractPlus]. The noradrenaline reuptake inhibitor desipramine acts directly on the α_2A_‐adrenoceptor to promote internalisation *via* recruitment of arrestin [http://www.ncbi.nlm.nih.gov/pubmed/21859713?dopt=AbstractPlus].

### Adrenoceptors, β

β‐Adrenoceptors are activated by the endogenous agonists (‐)‐http://www.guidetopharmacology.org/GRAC/LigandDisplayForward?ligandId=479 and (‐)‐http://www.guidetopharmacology.org/GRAC/LigandDisplayForward?ligandId=505. Isoprenaline is selective for β‐adrenoceptors relative to α_1_‐ and α_2_‐adrenoceptors, while http://www.guidetopharmacology.org/GRAC/LigandDisplayForward?ligandId=564 (p*K*i 8.2‐9.2) and http://www.guidetopharmacology.org/GRAC/LigandDisplayForward?ligandId=132 (p*K*i 10.011.0) are relatively β_1_ and β_2_ adrenoceptor‐selective antagonists. (‐)‐http://www.guidetopharmacology.org/GRAC/LigandDisplayForward?ligandId=505, http://www.guidetopharmacology.org/GRAC/LigandDisplayForward?ligandId=538 and (‐)‐http://www.guidetopharmacology.org/GRAC/LigandDisplayForward?ligandId=5571 show selectivity for β_1_‐ relative to β_2_‐adrenoceptors. Pharmacological differences exist between human and mouse β_3_‐adrenoceptors, and the ’rodent selective’ agonists http://www.guidetopharmacology.org/GRAC/LigandDisplayForward?ligandId=567 and http://www.guidetopharmacology.org/GRAC/LigandDisplayForward?ligandId=3462 have low efficacy at the human β_3_‐adrenoceptor whereas http://www.guidetopharmacology.org/GRAC/LigandDisplayForward?ligandId=532 and http://www.guidetopharmacology.org/GRAC/LigandDisplayForward?ligandId=3931 activate human β_3_‐adrenoceptors [88]. β_3_‐Adrenoceptors are resistant to blockade by http://www.guidetopharmacology.org/GRAC/LigandDisplayForward?ligandId=564, but can be blocked by high concentrations of http://www.guidetopharmacology.org/GRAC/LigandDisplayForward?ligandId=550. http://www.guidetopharmacology.org/GRAC/LigandDisplayForward?ligandId=547 has reasonably high affinity at β_3_‐adrenoceptors, but does not discriminate well between the three β‐ subtypes whereas http://www.guidetopharmacology.org/GRAC/LigandDisplayForward?ligandId=3931 is more selective. [^125^I]‐http://www.guidetopharmacology.org/GRAC/LigandDisplayForward?ligandId=132, [^125^I]‐hydroxy benzylpindolol and [^3^H]‐http://www.guidetopharmacology.org/GRAC/LigandDisplayForward?ligandId=563 are high affinity radioligands that label β_1_‐ and β_2_‐ adrenoceptors and β_3_‐adrenoceptors can be labelled with higher concentrations (nM) of [^125^I]‐http://www.guidetopharmacology.org/GRAC/LigandDisplayForward?ligandId=132 together with β_1_‐ and β_2_‐adrenoceptor antagonists. [^3^H]‐L748337 is a β_3_‐selective radioligand [http://www.ncbi.nlm.nih.gov/pubmed/24183974?dopt=AbstractPlus]. Fluorescent ligands such as BODIPY‐TMR‐CGP12177 can be used to track βadrenoceptors at the cellular level [8]. Somewhat selective β_1_adrenoceptor agonists (http://www.guidetopharmacology.org/GRAC/LigandDisplayForward?ligandId=534, http://www.guidetopharmacology.org/GRAC/LigandDisplayForward?ligandId=535) are used short term to treat cardiogenic shock but, chronically, reduce survival. β_1_‐Adrenoceptor‐preferring antagonists areused totreathypertension (http://www.guidetopharmacology.org/GRAC/LigandDisplayForward?ligandId=548, http://www.guidetopharmacology.org/GRAC/LigandDisplayForward?ligandId=549, http://www.guidetopharmacology.org/GRAC/LigandDisplayForward?ligandId=7129, http://www.guidetopharmacology.org/GRAC/LigandDisplayForward?ligandId=553 and http://www.guidetopharmacology.org/GRAC/LigandDisplayForward?ligandId=7246), cardiac arrhythmias (http://www.guidetopharmacology.org/GRAC/LigandDisplayForward?ligandId=548, bisoprolol, http://www.guidetopharmacology.org/GRAC/LigandDisplayForward?ligandId=7178) and cardiac failure (http://www.guidetopharmacology.org/GRAC/LigandDisplayForward?ligandId=553, nebivolol). Cardiac failure is also treated with carvedilol that blocks β_1_‐ and β_2_‐adrenoceptors, as well as α_1_‐adrenoceptors. Short (http://www.guidetopharmacology.org/GRAC/LigandDisplayForward?ligandId=558, http://www.guidetopharmacology.org/GRAC/LigandDisplayForward?ligandId=560) and long (http://www.guidetopharmacology.org/GRAC/LigandDisplayForward?ligandId=3465, http://www.guidetopharmacology.org/GRAC/LigandDisplayForward?ligandId=559) acting β_2_‐adrenoceptor‐selective agonists are powerful bronchodilators used to treat respiratory disorders. Many first generation β‐adrenoceptor antagonists (http://www.guidetopharmacology.org/GRAC/LigandDisplayForward?ligandId=564) block both β_1_‐ and β_2_‐adrenoceptors and there are no β_2_adrenoceptor‐selective antagonists used therapeutically. The β_3_‐adrenoceptor agonist http://www.guidetopharmacology.org/GRAC/LigandDisplayForward?ligandId=7445 is used to control overactive bladder syndrome.

**Table:** 

Nomenclature	http://www.guidetopharmacology.org/GRAC/ObjectDisplayForward?objectId=28‐http://www.guidetopharmacology.org/GRAC/ObjectDisplayForward?objectId=28	http://www.guidetopharmacology.org/GRAC/ObjectDisplayForward?objectId=29‐http://www.guidetopharmacology.org/GRAC/ObjectDisplayForward?objectId=29	http://www.guidetopharmacology.org/GRAC/ObjectDisplayForward?objectId=30‐http://www.guidetopharmacology.org/GRAC/ObjectDisplayForward?objectId=30
HGNC, UniProt	https://www.genenames.org/data/gene‐symbol‐report/#!/hgnc_id/HGNC:285, http://www.uniprot.org/uniprot/P08588	https://www.genenames.org/data/gene‐symbol‐report/#!/hgnc_id/HGNC:286, http://www.uniprot.org/uniprot/P07550	https://www.genenames.org/data/gene‐symbol‐report/#!/hgnc_id/HGNC:288, http://www.uniprot.org/uniprot/P13945
Potency order of endogenous ligands	(‐)‐http://www.guidetopharmacology.org/GRAC/LigandDisplayForward?ligandId=505 > (‐)‐http://www.guidetopharmacology.org/GRAC/LigandDisplayForward?ligandId=479	(‐)‐http://www.guidetopharmacology.org/GRAC/LigandDisplayForward?ligandId=479 > (‐)‐http://www.guidetopharmacology.org/GRAC/LigandDisplayForward?ligandId=505	(‐)‐http://www.guidetopharmacology.org/GRAC/LigandDisplayForward?ligandId=505 = (‐)‐http://www.guidetopharmacology.org/GRAC/LigandDisplayForward?ligandId=479
Endogenous agonists	(‐)‐http://www.guidetopharmacology.org/GRAC/LigandDisplayForward?ligandId=479 [http://www.ncbi.nlm.nih.gov/pubmed/2849109?dopt=AbstractPlus, http://www.ncbi.nlm.nih.gov/pubmed/14730417?dopt=AbstractPlus], (‐)‐http://www.guidetopharmacology.org/GRAC/LigandDisplayForward?ligandId=505, http://www.ncbi.nlm.nih.gov/pubmed/14730417?dopt=AbstractPlus], http://www.guidetopharmacology.org/GRAC/LigandDisplayForward?ligandId=484]	(‐)‐http://www.guidetopharmacology.org/GRAC/LigandDisplayForward?ligandId=479 [http://www.ncbi.nlm.nih.gov/pubmed/2849109?dopt=AbstractPlus, http://www.ncbi.nlm.nih.gov/pubmed/14730417?dopt=AbstractPlus, http://www.ncbi.nlm.nih.gov/pubmed/9295336?dopt=AbstractPlus], (‐)‐http://www.guidetopharmacology.org/GRAC/LigandDisplayForward?ligandId=505, http://www.ncbi.nlm.nih.gov/pubmed/14730417?dopt=AbstractPlus]	(‐)‐http://www.guidetopharmacology.org/GRAC/LigandDisplayForward?ligandId=505 [http://www.ncbi.nlm.nih.gov/pubmed/14730417?dopt=AbstractPlus, http://www.ncbi.nlm.nih.gov/pubmed/14744619?dopt=AbstractPlus, http://www.ncbi.nlm.nih.gov/pubmed/9131260?dopt=AbstractPlus], (‐)‐http://www.guidetopharmacology.org/GRAC/LigandDisplayForward?ligandId=479]
Agonists	http://www.guidetopharmacology.org/GRAC/LigandDisplayForward?ligandId=91 (Partial agonist) [http://www.ncbi.nlm.nih.gov/pubmed/17804228?dopt=AbstractPlus], http://www.guidetopharmacology.org/GRAC/LigandDisplayForward?ligandId=536 [http://www.ncbi.nlm.nih.gov/pubmed/2849109?dopt=AbstractPlus, http://www.ncbi.nlm.nih.gov/pubmed/8982677?dopt=AbstractPlus], http://www.guidetopharmacology.org/GRAC/LigandDisplayForward?ligandId=535 (Partial agonist) [http://www.ncbi.nlm.nih.gov/pubmed/10531390?dopt=AbstractPlus]	http://www.guidetopharmacology.org/GRAC/LigandDisplayForward?ligandId=91 (Partial agonist) [http://www.ncbi.nlm.nih.gov/pubmed/17804228?dopt=AbstractPlus], http://www.guidetopharmacology.org/GRAC/LigandDisplayForward?ligandId=7479 [http://www.ncbi.nlm.nih.gov/pubmed/15324892?dopt=AbstractPlus], http://www.guidetopharmacology.org/GRAC/LigandDisplayForward?ligandId=536 [http://www.ncbi.nlm.nih.gov/pubmed/8982677?dopt=AbstractPlus], http://www.guidetopharmacology.org/GRAC/LigandDisplayForward?ligandId=556 (Partial agonist) [http://www.ncbi.nlm.nih.gov/pubmed/9295336?dopt=AbstractPlus]	http://www.guidetopharmacology.org/GRAC/LigandDisplayForward?ligandId=569 [http://www.ncbi.nlm.nih.gov/pubmed/8719421?dopt=AbstractPlus]
Selective agonists	(‐)‐http://www.guidetopharmacology.org/GRAC/LigandDisplayForward?ligandId=5571 [http://www.ncbi.nlm.nih.gov/pubmed/9117106?dopt=AbstractPlus], http://www.guidetopharmacology.org/GRAC/LigandDisplayForward?ligandId=538 (Partial agonist) [http://www.ncbi.nlm.nih.gov/pubmed/10531390?dopt=AbstractPlus], http://www.guidetopharmacology.org/GRAC/LigandDisplayForward?ligandId=534 (Partial agonist) [http://www.ncbi.nlm.nih.gov/pubmed/10531390?dopt=AbstractPlus, http://www.ncbi.nlm.nih.gov/pubmed/7902433?dopt=AbstractPlus]	http://www.guidetopharmacology.org/GRAC/LigandDisplayForward?ligandId=3465 [http://www.ncbi.nlm.nih.gov/pubmed/20590599?dopt=AbstractPlus], http://www.guidetopharmacology.org/GRAC/LigandDisplayForward?ligandId=559], http://www.guidetopharmacology.org/GRAC/LigandDisplayForward?ligandId=3466], http://www.guidetopharmacology.org/GRAC/LigandDisplayForward?ligandId=7353 [http://www.ncbi.nlm.nih.gov/pubmed/20462258?dopt=AbstractPlus], http://www.guidetopharmacology.org/GRAC/LigandDisplayForward?ligandId=3464], http://www.guidetopharmacology.org/GRAC/LigandDisplayForward?ligandId=7455 [http://www.ncbi.nlm.nih.gov/pubmed/22932315?dopt=AbstractPlus], http://www.guidetopharmacology.org/GRAC/LigandDisplayForward?ligandId=557 [http://www.ncbi.nlm.nih.gov/pubmed/24454993?dopt=AbstractPlus], http://www.guidetopharmacology.org/GRAC/LigandDisplayForward?ligandId=558 (Partial agonist) [http://www.ncbi.nlm.nih.gov/pubmed/15655528?dopt=AbstractPlus, http://www.ncbi.nlm.nih.gov/pubmed/10531390?dopt=AbstractPlus], http://www.guidetopharmacology.org/GRAC/LigandDisplayForward?ligandId=560 (Partial agonist) [http://www.ncbi.nlm.nih.gov/pubmed/15655528?dopt=AbstractPlus], http://www.guidetopharmacology.org/GRAC/LigandDisplayForward?ligandId=7250 [http://www.ncbi.nlm.nih.gov/pubmed/19168263?dopt=AbstractPlus]	http://www.guidetopharmacology.org/GRAC/LigandDisplayForward?ligandId=3931 [http://www.ncbi.nlm.nih.gov/pubmed/20590599?dopt=AbstractPlus], http://www.guidetopharmacology.org/GRAC/LigandDisplayForward?ligandId=3467 [http://www.ncbi.nlm.nih.gov/pubmed/9873496?dopt=AbstractPlus], http://www.guidetopharmacology.org/GRAC/LigandDisplayForward?ligandId=7445 [http://www.ncbi.nlm.nih.gov/pubmed/17293563?dopt=AbstractPlus], http://www.guidetopharmacology.org/GRAC/LigandDisplayForward?ligandId=532 (Partial agonist) [http://www.ncbi.nlm.nih.gov/pubmed/7903415?dopt=AbstractPlus, http://www.ncbi.nlm.nih.gov/pubmed/10079020?dopt=AbstractPlus, http://www.ncbi.nlm.nih.gov/pubmed/8719421?dopt=AbstractPlus, http://www.ncbi.nlm.nih.gov/pubmed/9117106?dopt=AbstractPlus], http://www.guidetopharmacology.org/GRAC/LigandDisplayForward?ligandId=3468 [http://www.ncbi.nlm.nih.gov/pubmed/11959793?dopt=AbstractPlus] – Mouse, http://www.guidetopharmacology.org/GRAC/LigandDisplayForward?ligandId=567[http://www.ncbi.nlm.nih.gov/pubmed/7903415?dopt=AbstractPlus, http://www.ncbi.nlm.nih.gov/pubmed/7912272?dopt=AbstractPlus, http://www.ncbi.nlm.nih.gov/pubmed/14730417?dopt=AbstractPlus, http://www.ncbi.nlm.nih.gov/pubmed/8719421?dopt=AbstractPlus], http://www.guidetopharmacology.org/GRAC/LigandDisplayForward?ligandId=3462 [http://www.ncbi.nlm.nih.gov/pubmed/11249148?dopt=AbstractPlus]
Antagonists	http://www.guidetopharmacology.org/GRAC/LigandDisplayForward?ligandId=551 (p*K*_i_ 9.5) [http://www.ncbi.nlm.nih.gov/pubmed/10411574?dopt=AbstractPlus], http://www.guidetopharmacology.org/GRAC/LigandDisplayForward?ligandId=550 (p*K*_i_ 7.3–9) [http://www.ncbi.nlm.nih.gov/pubmed/10411574?dopt=AbstractPlus, http://www.ncbi.nlm.nih.gov/pubmed/10079020?dopt=AbstractPlus], http://www.guidetopharmacology.org/GRAC/LigandDisplayForward?ligandId=547 (p*K*_i_ 8.6) [http://www.ncbi.nlm.nih.gov/pubmed/10411574?dopt=AbstractPlus], http://www.guidetopharmacology.org/GRAC/LigandDisplayForward?ligandId=570 (p*K*_i_ 8.4) [79], http://www.guidetopharmacology.org/GRAC/LigandDisplayForward?ligandId=7207 (p*K*_i_ 8.2) [79], http://www.guidetopharmacology.org/GRAC/LigandDisplayForward?ligandId=553 (p*K*_i_ 7–7.6) [http://www.ncbi.nlm.nih.gov/pubmed/15655528?dopt=AbstractPlus, http://www.ncbi.nlm.nih.gov/pubmed/15655528?dopt=AbstractPlus, http://www.ncbi.nlm.nih.gov/pubmed/14730417?dopt=AbstractPlus, http://www.ncbi.nlm.nih.gov/pubmed/10079020?dopt=AbstractPlus], http://www.guidetopharmacology.org/GRAC/LigandDisplayForward?ligandId=7178 (p*K*_i_ 6.9) [79], http://www.guidetopharmacology.org/GRAC/LigandDisplayForward?ligandId=554 (p*K*_i_ 6.9) [http://www.ncbi.nlm.nih.gov/pubmed/10411574?dopt=AbstractPlus], http://www.guidetopharmacology.org/GRAC/LigandDisplayForward?ligandId=555 (p*K*_i_ 6.1–6.8) [http://www.ncbi.nlm.nih.gov/pubmed/15655528?dopt=AbstractPlus], http://www.guidetopharmacology.org/GRAC/LigandDisplayForward?ligandId=2561 (p*K*_i_ 6.7) [79], http://www.guidetopharmacology.org/GRAC/LigandDisplayForward?ligandId=7297 (p*K*_i_ 6.1) [79]	http://www.guidetopharmacology.org/GRAC/LigandDisplayForward?ligandId=551 (p*K*_i_ 9.4–9.9) [http://www.ncbi.nlm.nih.gov/pubmed/15655528?dopt=AbstractPlus, http://www.ncbi.nlm.nih.gov/pubmed/10411574?dopt=AbstractPlus], http://www.guidetopharmacology.org/GRAC/LigandDisplayForward?ligandId=565 (p*K*_i_ 9.7) [http://www.ncbi.nlm.nih.gov/pubmed/15655528?dopt=AbstractPlus], http://www.guidetopharmacology.org/GRAC/LigandDisplayForward?ligandId=564 (p*K*_i_ 9.1–9.5) [http://www.ncbi.nlm.nih.gov/pubmed/15655528?dopt=AbstractPlus, http://www.ncbi.nlm.nih.gov/pubmed/12920204?dopt=AbstractPlus, http://www.ncbi.nlm.nih.gov/pubmed/10531390?dopt=AbstractPlus, http://www.ncbi.nlm.nih.gov/pubmed/10079020?dopt=AbstractPlus], http://www.guidetopharmacology.org/GRAC/LigandDisplayForward?ligandId=547 (p*K*_i_ 9.3) [http://www.ncbi.nlm.nih.gov/pubmed/10411574?dopt=AbstractPlus], http://www.guidetopharmacology.org/GRAC/LigandDisplayForward?ligandId=570 (p*K*_i_ 9.3) [79], http://www.guidetopharmacology.org/GRAC/LigandDisplayForward?ligandId=550 (p*K*_i_ 8.3–9.1) [http://www.ncbi.nlm.nih.gov/pubmed/10411574?dopt=AbstractPlus, http://www.ncbi.nlm.nih.gov/pubmed/10079020?dopt=AbstractPlus], http://www.guidetopharmacology.org/GRAC/LigandDisplayForward?ligandId=563 (p*K*i 9) [http://www.ncbi.nlm.nih.gov/pubmed/15655528?dopt=AbstractPlus], http://www.guidetopharmacology.org/GRAC/LigandDisplayForward?ligandId=554 (p*K*_i_ 7–8.6) [http://www.ncbi.nlm.nih.gov/pubmed/15655528?dopt=AbstractPlus], http://www.guidetopharmacology.org/GRAC/LigandDisplayForward?ligandId=7207 (p*K*_i_ 8) [79], http://www.guidetopharmacology.org/GRAC/LigandDisplayForward?ligandId=2561 (p*K*_i_ 7.4) [79], http://www.guidetopharmacology.org/GRAC/LigandDisplayForward?ligandId=7297 (p*K*_i_ 6.5) [79]	http://www.guidetopharmacology.org/GRAC/LigandDisplayForward?ligandId=547 (p*K*_i_ 6.9–8.4) [http://www.ncbi.nlm.nih.gov/pubmed/10411574?dopt=AbstractPlus, http://www.ncbi.nlm.nih.gov/pubmed/8730727?dopt=AbstractPlus, http://www.ncbi.nlm.nih.gov/pubmed/14730417?dopt=AbstractPlus], http://www.guidetopharmacology.org/GRAC/LigandDisplayForward?ligandId=550 (p*K*_i_ 6.8–7.3) [http://www.ncbi.nlm.nih.gov/pubmed/7903415?dopt=AbstractPlus, http://www.ncbi.nlm.nih.gov/pubmed/10079020?dopt=AbstractPlus, http://www.ncbi.nlm.nih.gov/pubmed/8719421?dopt=AbstractPlus], http://www.guidetopharmacology.org/GRAC/LigandDisplayForward?ligandId=564 (p*K*i 6.3–7.2) [http://www.ncbi.nlm.nih.gov/pubmed/10079020?dopt=AbstractPlus, http://www.ncbi.nlm.nih.gov/pubmed/14744619?dopt=AbstractPlus], http://www.guidetopharmacology.org/GRAC/LigandDisplayForward?ligandId=570 (p*K*_i_ 6.8) [http://www.ncbi.nlm.nih.gov/pubmed/14744619?dopt=AbstractPlus]
Selective antagonists	http://www.guidetopharmacology.org/GRAC/LigandDisplayForward?ligandId=541 (p*K*_i_ 8.5–9.2) [http://www.ncbi.nlm.nih.gov/pubmed/15655528?dopt=AbstractPlus, http://www.ncbi.nlm.nih.gov/pubmed/10411574?dopt=AbstractPlus, http://www.ncbi.nlm.nih.gov/pubmed/10079020?dopt=AbstractPlus], http://www.guidetopharmacology.org/GRAC/LigandDisplayForward?ligandId=8035 (p*K*_i_ 9.1) [http://www.ncbi.nlm.nih.gov/pubmed/11572462?dopt=AbstractPlus], http://www.guidetopharmacology.org/GRAC/LigandDisplayForward?ligandId=549 (p*K*_i_ 8.8) [http://www.ncbi.nlm.nih.gov/pubmed/10079020?dopt=AbstractPlus], http://www.guidetopharmacology.org/GRAC/LigandDisplayForward?ligandId=7246 (pIC_50_ 8.1–8.7) [http://www.ncbi.nlm.nih.gov/pubmed/2462161?dopt=AbstractPlus] – Rabbit, http://www.guidetopharmacology.org/GRAC/LigandDisplayForward?ligandId=548 (p*K*_i_ 6.7–7.6) [http://www.ncbi.nlm.nih.gov/pubmed/15655528?dopt=AbstractPlus, http://www.ncbi.nlm.nih.gov/pubmed/15060759?dopt=AbstractPlus, http://www.ncbi.nlm.nih.gov/pubmed/15060759?dopt=AbstractPlus], http://www.guidetopharmacology.org/GRAC/LigandDisplayForward?ligandId=7107 (p*K*_i_ 6.4) [79]	http://www.guidetopharmacology.org/GRAC/LigandDisplayForward?ligandId=543 (Inverse agonist) (p*K*_i_ 9.2–9.5) [http://www.ncbi.nlm.nih.gov/pubmed/15655528?dopt=AbstractPlus, http://www.ncbi.nlm.nih.gov/pubmed/12920204?dopt=AbstractPlus, http://www.ncbi.nlm.nih.gov/pubmed/10079020?dopt=AbstractPlus]	http://www.guidetopharmacology.org/GRAC/LigandDisplayForward?ligandId=3932 (p*K*_i_ 8.4) [http://www.ncbi.nlm.nih.gov/pubmed/10411574?dopt=AbstractPlus], http://www.guidetopharmacology.org/GRAC/LigandDisplayForward?ligandId=3463 (p*K*_i_ 8.4) [http://www.ncbi.nlm.nih.gov/pubmed/10411574?dopt=AbstractPlus]
Labelled ligands	[http://www.guidetopharmacology.org/GRAC/LigandDisplayForward?ligandId=562 (Antagonist) (p*K*_d_ 10.4–11.3) [http://www.ncbi.nlm.nih.gov/pubmed/10531390?dopt=AbstractPlus, http://www.ncbi.nlm.nih.gov/pubmed/10079020?dopt=AbstractPlus, http://www.ncbi.nlm.nih.gov/pubmed/8982677?dopt=AbstractPlus]	[http://www.guidetopharmacology.org/GRAC/LigandDisplayForward?ligandId=562 (Antagonist) (p*K*_d_ 11.1) [http://www.ncbi.nlm.nih.gov/pubmed/10079020?dopt=AbstractPlus, http://www.ncbi.nlm.nih.gov/pubmed/8982677?dopt=AbstractPlus]	[http://www.guidetopharmacology.org/GRAC/LigandDisplayForward?ligandId=562 (Agonist, Partial agonist) [http://www.ncbi.nlm.nih.gov/pubmed/10079020?dopt=AbstractPlus, http://www.ncbi.nlm.nih.gov/pubmed/9117106?dopt=AbstractPlus, http://www.ncbi.nlm.nih.gov/pubmed/14744619?dopt=AbstractPlus, http://www.ncbi.nlm.nih.gov/pubmed/8982677?dopt=AbstractPlus, http://www.ncbi.nlm.nih.gov/pubmed/9131260?dopt=AbstractPlus]
Comments	The agonists indicated have less than two orders of magnitude selectivity [http://www.ncbi.nlm.nih.gov/pubmed/20590599?dopt=AbstractPlus].	–	Agonist http://www.guidetopharmacology.org/GRAC/LigandDisplayForward?ligandId=3468 has a pEC50 of 6.9 for the splice variant of the mouse β_3_ receptor, β_3b_ [http://www.ncbi.nlm.nih.gov/pubmed/11959793?dopt=AbstractPlus].

### Comments

[http://www.guidetopharmacology.org/GRAC/LigandDisplayForward?ligandId=562 can be used to define β_1_‐ or β_2_adrenoceptors when conducted in the presence of a β_1_‐ or β_2_adrenoceptor‐selective antagonist. A fluorescent analogue of http://www.guidetopharmacology.org/GRAC/LigandDisplayForward?ligandId=532 can be used to study β_2_‐adrenoceptors in living cells [http://www.ncbi.nlm.nih.gov/pubmed/12770928?dopt=AbstractPlus]. [http://www.guidetopharmacology.org/GRAC/LigandDisplayForward?ligandId=562 at higher (nM) concentrations can be used to label β_3_‐adrenoceptors in systems with few if any other β‐adrenoceptor subtypes. The β_3_‐adrenoceptor has an intron in the coding region, but splice variants have only been described for the mouse [http://www.ncbi.nlm.nih.gov/pubmed/10455305?dopt=AbstractPlus], where the isoforms display different signalling characteristics [http://www.ncbi.nlm.nih.gov/pubmed/11959793?dopt=AbstractPlus]. There are 3 β‐adrenoceptors in turkey (termed the tβ, tβ3c and tβ4c) that have a pharmacology that differs from the human β‐adrenoceptors [http://www.ncbi.nlm.nih.gov/pubmed/21152092?dopt=AbstractPlus]. Numerous polymorphisms have been described for the β‐adrenoceptors; some are associated with signalling and trafficking, altered susceptibility to disease and/or altered responses to pharmacotherapy [http://www.ncbi.nlm.nih.gov/pubmed/15090197?dopt=AbstractPlus]. All β‐adrenoceptors couple to G_s_ (activating adenylyl cyclase and elevating cAMP levels), but also activate G_i_ and β‐arrestin‐mediated signalling. Many β_1_‐ and β_2_‐adrenoceptor antagonists are agonists at β_3_adrenoceptors (http://www.guidetopharmacology.org/GRAC/LigandDisplayForward?ligandId=3462 and http://www.guidetopharmacology.org/GRAC/LigandDisplayForward?ligandId=569). Many ‘antagonists’ of cAMP accumulation, for example http://www.guidetopharmacology.org/GRAC/LigandDisplayForward?ligandId=551 and bucindolol, weakly activate MAP kinase pathways [http://www.ncbi.nlm.nih.gov/pubmed/14645666?dopt=AbstractPlus, http://www.ncbi.nlm.nih.gov/pubmed/20132209?dopt=AbstractPlus, http://www.ncbi.nlm.nih.gov/pubmed/16901982?dopt=AbstractPlus, http://www.ncbi.nlm.nih.gov/pubmed/18403719?dopt=AbstractPlus, http://www.ncbi.nlm.nih.gov/pubmed/17717109?dopt=AbstractPlus, http://www.ncbi.nlm.nih.gov/pubmed/18684840?dopt=AbstractPlus] and thus display ’protean agonism’. http://www.guidetopharmacology.org/GRAC/LigandDisplayForward?ligandId=550 acts as a neutral antagonist in most systems so far examined. Agonists also display biased signalling at the β_2_‐adrenoceptor via G_s_ or arrestins [http://www.ncbi.nlm.nih.gov/pubmed/18086673?dopt=AbstractPlus]. X‐ray crystal structures have been described of the agonist bound [http://www.ncbi.nlm.nih.gov/pubmed/21228877?dopt=AbstractPlus] and antagonist bound forms of the β_1_[http://www.ncbi.nlm.nih.gov/pubmed/18594507?dopt=AbstractPlus], agonist‐bound [http://www.ncbi.nlm.nih.gov/pubmed/17962520?dopt=AbstractPlus] and antagonist‐bound forms of the β_2_‐adrenoceptor [http://www.ncbi.nlm.nih.gov/pubmed/21228869?dopt=AbstractPlus, http://www.ncbi.nlm.nih.gov/pubmed/21228876?dopt=AbstractPlus], as well as a fully active agonistbound, G_s_ protein‐coupled β2‐adrenoceptor [http://www.ncbi.nlm.nih.gov/pubmed/21772288?dopt=AbstractPlus]. http://www.guidetopharmacology.org/GRAC/LigandDisplayForward?ligandId=551 and bucindolol bind to a site on the β1‐adrenoceptor involving contacts in TM2, 3, and 7 and extracellular loop 2 that may facilitate coupling to arrestins [http://www.ncbi.nlm.nih.gov/pubmed/18594507?dopt=AbstractPlus]. Compounds displaying arrestinbiased signalling at the β2‐adrenoceptor have a greater effect on the conformation of TM7, whereas full agonists for G_s_ coupling promote movement of TM5 and TM6 [http://www.ncbi.nlm.nih.gov/pubmed/22267580?dopt=AbstractPlus]. Recent studies using NMR spectroscopy demonstrate significant conformational flexibility in the β_2_‐adrenoceptor that is stabilized by both agonist and G proteins highlighting the dynamic nature of interactions with both ligand and downstreamsignalling partners [http://www.ncbi.nlm.nih.gov/pubmed/23721409?dopt=AbstractPlus, http://www.ncbi.nlm.nih.gov/pubmed/25981665?dopt=AbstractPlus, http://www.ncbi.nlm.nih.gov/pubmed/23374348?dopt=AbstractPlus]. Such flexibility likely has consequences for our understanding of biased agonism, and for the future therapeutic exploitation of this phenomenon.

### Further reading on Adrenoceptors

Baker JG et al. (2011) Evolution of β‐blockers: from anti‐anginal drugs to ligand‐directed signalling. *Trends Pharmacol. Sci.* **32**: 227‐34 [https://www.ncbi.nlm.nih.gov/pubmed/21429598?dopt=AbstractPlus]

Bylund DB et al. (1994) International Union of Pharmacology nomenclature of adrenoceptors. *Pharmacol. Rev.* **46**: 121‐136 [https://www.ncbi.nlm.nih.gov/pubmed/7938162?dopt=AbstractPlus]

Evans BA et al. (2010) Ligand‐directed signalling at beta‐adrenoceptors. *Br. J. Pharmacol.* **159**: 1022‐38 [https://www.ncbi.nlm.nih.gov/pubmed/20132209?dopt=AbstractPlus]

Jensen BC et al. (2011) Alpha‐1‐adrenergic receptors: targets for agonist drugs to treat heart failure. *J. Mol. Cell. Cardiol.* **51**: 518‐28 [https://www.ncbi.nlm.nih.gov/pubmed/21118696?dopt=AbstractPlus]

Kobilka BK. (2011) Structural insights into adrenergic receptor function and pharmacology. *Trends Pharmacol. Sci.* **32**: 213‐8 [https://www.ncbi.nlm.nih.gov/pubmed/21414670?dopt=AbstractPlus]

Langer SZ. (2015) a2‐Adrenoceptors in the treatment of major neuropsychiatric disorders. *Trends Pharmacol. Sci.* **36**: 196‐202 [https://www.ncbi.nlm.nih.gov/pubmed/25771972?dopt=AbstractPlus]

Michel MC et al. (2015) Selectivity of pharmacological tools: implications for use in cell physiology. A review in the theme: Cell signaling: proteins, pathways and mechanisms. *Am. J. Physiol., Cell Physiol.* **308**: C505‐20 [https://www.ncbi.nlm.nih.gov/pubmed/25631871?dopt=AbstractPlus]

## http://www.guidetopharmacology.org/GRAC/FamilyDisplayForward?familyId=6

### Overview

The actions of http://www.guidetopharmacology.org/GRAC/LigandDisplayForward?ligandId=2504 (https://www.genenames.org/data/gene‐symbol‐report/#!/hgnc_id/HGNC:333, http://www.uniprot.org/uniprot/P01019) (Ang II) are mediated by AT_1_ and AT_2_ receptors (**nomenclature as agreed by the NC‐IUPHAR Subcommittee on Angiotensin receptors** [http://www.ncbi.nlm.nih.gov/pubmed/10977869?dopt=AbstractPlus, http://www.ncbi.nlm.nih.gov/pubmed/26315714?dopt=AbstractPlus]), which have around 30% sequence similarity. The decapeptide http://www.guidetopharmacology.org/GRAC/LigandDisplayForward?ligandId=583, http://www.uniprot.org/uniprot/P01019), the octapeptide http://www.guidetopharmacology.org/GRAC/LigandDisplayForward?ligandId=2504, http://www.uniprot.org/uniprot/P01019) and the heptapeptide http://www.guidetopharmacology.org/GRAC/LigandDisplayForward?ligandId=585, http://www.uniprot.org/uniprot/P01019) are endogenous ligands. http://www.guidetopharmacology.org/GRAC/LigandDisplayForward?ligandId=590, http://www.guidetopharmacology.org/GRAC/LigandDisplayForward?ligandId=587, http://www.guidetopharmacology.org/GRAC/LigandDisplayForward?ligandId=592, etc. are clinically used AT_1_ receptor blockers.

**Table:** 

Nomenclature	http://www.guidetopharmacology.org/GRAC/ObjectDisplayForward?objectId=34	http://www.guidetopharmacology.org/GRAC/ObjectDisplayForward?objectId=35
HGNC, UniProt	https://www.genenames.org/data/gene‐symbol‐report/#!/hgnc_id/HGNC:336, http://www.uniprot.org/uniprot/P30556	https://www.genenames.org/data/gene‐symbol‐report/#!/hgnc_id/HGNC:338, http://www.uniprot.org/uniprot/P50052
Endogenous agonists	http://www.guidetopharmacology.org/GRAC/LigandDisplayForward?ligandId=2504 (https://www.genenames.org/data/gene‐symbol‐report/#!/hgnc_id/HGNC:333, http://www.uniprot.org/uniprot/P01019) [466, http://www.ncbi.nlm.nih.gov/pubmed/10193788?dopt=AbstractPlus], http://www.guidetopharmacology.org/GRAC/LigandDisplayForward?ligandId=585, http://www.uniprot.org/uniprot/P01019) [466], http://www.guidetopharmacology.org/GRAC/LigandDisplayForward?ligandId=5368, http://www.uniprot.org/uniprot/P01019) (Partial agonist) [http://www.ncbi.nlm.nih.gov/pubmed/12006574?dopt=AbstractPlus]	http://www.guidetopharmacology.org/GRAC/LigandDisplayForward?ligandId=585 (https://www.genenames.org/data/gene‐symbol‐report/#!/hgnc_id/HGNC:333, http://www.uniprot.org/uniprot/P01019) [http://www.ncbi.nlm.nih.gov/pubmed/8242249?dopt=AbstractPlus, 466, http://www.ncbi.nlm.nih.gov/pubmed/2775266?dopt=AbstractPlus], http://www.guidetopharmacology.org/GRAC/LigandDisplayForward?ligandId=2504, http://www.uniprot.org/uniprot/P01019) [466, http://www.ncbi.nlm.nih.gov/pubmed/7850406?dopt=AbstractPlus, http://www.ncbi.nlm.nih.gov/pubmed/2775266?dopt=AbstractPlus], http://www.guidetopharmacology.org/GRAC/LigandDisplayForward?ligandId=582) (https://www.genenames.org/data/gene‐symbol‐report/#!/hgnc_id/HGNC:333, http://www.uniprot.org/uniprot/P01019) [http://www.ncbi.nlm.nih.gov/pubmed/21542804?dopt=AbstractPlus]
Agonists	[http://www.guidetopharmacology.org/GRAC/LigandDisplayForward?ligandId=6903,http://www.guidetopharmacology.org/GRAC/LigandDisplayForward?ligandId=6903]http://www.guidetopharmacology.org/GRAC/LigandDisplayForward?ligandId=6903 [http://www.ncbi.nlm.nih.gov/pubmed/11901215?dopt=AbstractPlus, http://www.ncbi.nlm.nih.gov/pubmed/10066768?dopt=AbstractPlus] – Rat	–
Selective agonists	http://www.guidetopharmacology.org/GRAC/LigandDisplayForward?ligandId=3936 [http://www.ncbi.nlm.nih.gov/pubmed/7829475?dopt=AbstractPlus], http://www.guidetopharmacology.org/GRAC/LigandDisplayForward?ligandId=6895 [http://www.ncbi.nlm.nih.gov/pubmed/9383393?dopt=AbstractPlus]	http://www.guidetopharmacology.org/GRAC/LigandDisplayForward?ligandId=3944 [http://www.ncbi.nlm.nih.gov/pubmed/21542804?dopt=AbstractPlus], [http://www.guidetopharmacology.org/GRAC/LigandDisplayForward?ligandId=595 [466, http://www.ncbi.nlm.nih.gov/pubmed/2194459?dopt=AbstractPlus] – Rat, http://www.guidetopharmacology.org/GRAC/LigandDisplayForward?ligandId=6918 [http://www.ncbi.nlm.nih.gov/pubmed/22802221?dopt=AbstractPlus]
Antagonists	http://www.guidetopharmacology.org/GRAC/LigandDisplayForward?ligandId=6899 (p*K*_i_ 9.1) [http://www.ncbi.nlm.nih.gov/pubmed/7853190?dopt=AbstractPlus] – Rat, http://www.guidetopharmacology.org/GRAC/LigandDisplayForward?ligandId=6913 (pIC_50_ 8.8) [http://www.ncbi.nlm.nih.gov/pubmed/22410249?dopt=AbstractPlus] – Rat, http://www.guidetopharmacology.org/GRAC/LigandDisplayForward?ligandId=6917 (pIC_50_ 8.5) [http://www.ncbi.nlm.nih.gov/pubmed/22889560?dopt=AbstractPlus], http://www.guidetopharmacology.org/GRAC/LigandDisplayForward?ligandId=6910 (pIC_50_ 8.3) [http://www.ncbi.nlm.nih.gov/pubmed/11303957?dopt=AbstractPlus], http://www.guidetopharmacology.org/GRAC/LigandDisplayForward?ligandId=6902 (p*K*_d_ 7.7) [http://www.ncbi.nlm.nih.gov/pubmed/20801892?dopt=AbstractPlus]	http://www.guidetopharmacology.org/GRAC/LigandDisplayForward?ligandId=598 (pIC_50_ 9) [http://www.ncbi.nlm.nih.gov/pubmed/2590220?dopt=AbstractPlus] – Rat
Selective antagonists	http://www.guidetopharmacology.org/GRAC/LigandDisplayForward?ligandId=587 (pIC_50_ 9.5–9.7) [http://www.ncbi.nlm.nih.gov/pubmed/10193788?dopt=AbstractPlus], http://www.guidetopharmacology.org/GRAC/LigandDisplayForward?ligandId=588 (pIC_50_ 8.4–8.8) [http://www.ncbi.nlm.nih.gov/pubmed/1309870?dopt=AbstractPlus], http://www.guidetopharmacology.org/GRAC/LigandDisplayForward?ligandId=590 (pIC_50_ 7.4–8.7) [466, http://www.ncbi.nlm.nih.gov/pubmed/8372104?dopt=AbstractPlus], http://www.guidetopharmacology.org/GRAC/LigandDisplayForward?ligandId=592 (pIC_50_ 8.4) [http://www.ncbi.nlm.nih.gov/pubmed/9878991?dopt=AbstractPlus], http://www.guidetopharmacology.org/GRAC/LigandDisplayForward?ligandId=591 (pIC_50_ 8.1) [http://www.ncbi.nlm.nih.gov/pubmed/11451212?dopt=AbstractPlus]	http://www.guidetopharmacology.org/GRAC/LigandDisplayForward?ligandId=596 (pIC_50_ 8.5–9.5) [http://www.ncbi.nlm.nih.gov/pubmed/2314387?dopt=AbstractPlus, http://www.ncbi.nlm.nih.gov/pubmed/2590220?dopt=AbstractPlus, http://www.ncbi.nlm.nih.gov/pubmed/8469774?dopt=AbstractPlus] – Rat, http://www.guidetopharmacology.org/GRAC/LigandDisplayForward?ligandId=8374 (pIC_50_ 8.5–9.3) [http://www.ncbi.nlm.nih.gov/pubmed/24507378?dopt=AbstractPlus, http://www.ncbi.nlm.nih.gov/pubmed/24507377?dopt=AbstractPlus, http://www.ncbi.nlm.nih.gov/pubmed/23489258?dopt=AbstractPlus], http://www.guidetopharmacology.org/GRAC/LigandDisplayForward?ligandId=597 (p*K*_d_ 8.7–9.2) [466, http://www.ncbi.nlm.nih.gov/pubmed/2402226?dopt=AbstractPlus, http://www.ncbi.nlm.nih.gov/pubmed/1709220?dopt=AbstractPlus]
Labelled ligands	[http://www.guidetopharmacology.org/GRAC/LigandDisplayForward?ligandId=6907 (Antagonist) (p*K*_d_ 10.3) [http://www.ncbi.nlm.nih.gov/pubmed/10079018?dopt=AbstractPlus], [http://www.guidetopharmacology.org/GRAC/LigandDisplayForward?ligandId=6897]http://www.guidetopharmacology.org/GRAC/LigandDisplayForward?ligandId=6897 (Agonist) [http://www.ncbi.nlm.nih.gov/pubmed/7759541?dopt=AbstractPlus] – Rat, [http://www.guidetopharmacology.org/GRAC/LigandDisplayForward?ligandId=6906,http://www.guidetopharmacology.org/GRAC/LigandDisplayForward?ligandId=6906]http://www.guidetopharmacology.org/GRAC/LigandDisplayForward?ligandId=6906 (Agonist, Partial agonist) [http://www.ncbi.nlm.nih.gov/pubmed/7759541?dopt=AbstractPlus] – Rat, [http://www.guidetopharmacology.org/GRAC/LigandDisplayForward?ligandId=3940 (Antagonist) (p*K*_d_ 9.1) [http://www.ncbi.nlm.nih.gov/pubmed/8463997?dopt=AbstractPlus] – Rat, [http://www.guidetopharmacology.org/GRAC/LigandDisplayForward?ligandId=3941 (Antagonist) (p*K*_d_ 8.2) [http://www.ncbi.nlm.nih.gov/pubmed/8282008?dopt=AbstractPlus] – Rat	[http://www.guidetopharmacology.org/GRAC/LigandDisplayForward?ligandId=594 (Agonist) [466, http://www.ncbi.nlm.nih.gov/pubmed/2775266?dopt=AbstractPlus, http://www.ncbi.nlm.nih.gov/pubmed/1764088?dopt=AbstractPlus], [http://www.guidetopharmacology.org/GRAC/LigandDisplayForward?ligandId=6906,http://www.guidetopharmacology.org/GRAC/LigandDisplayForward?ligandId=6906]http://www.guidetopharmacology.org/GRAC/LigandDisplayForward?ligandId=6906 (Agonist) [http://www.ncbi.nlm.nih.gov/pubmed/10024318?dopt=AbstractPlus] – Rat
Comments	http://www.guidetopharmacology.org/GRAC/LigandDisplayForward?ligandId=592 and http://www.guidetopharmacology.org/GRAC/LigandDisplayForward?ligandId=587 are also reported to be agonists of PPARγ [http://www.ncbi.nlm.nih.gov/pubmed/19021699?dopt=AbstractPlus].	–

### Comments

AT_1_ receptors are predominantly coupled to G_q/11_, however they are also linked to arrestin recruitment and stimulate G protein‐independent arrestin signalling [http://www.ncbi.nlm.nih.gov/pubmed/20427692?dopt=AbstractPlus]. Most species express a single *AGTR1* gene, but two related *agtr1a* and a*gtr1b* receptor genes are expressed in rodents. The AT_2_ receptor counteracts several of the growth responses initiated by the AT_1_ receptors. The AT_2_ receptor is much less abundant than the AT_1_ receptor in adult tissues and is upregulated in pathological conditions. AT_1_ receptor antagonists bearing substituted 4‐phenylquinoline moieties have been synthesized, which bind to AT_1_ receptors with nanomolar affinity and are slightly more potent than http://www.guidetopharmacology.org/GRAC/LigandDisplayForward?ligandId=590 in functional studies [http://www.ncbi.nlm.nih.gov/pubmed/15115399?dopt=AbstractPlus]. The antagonist activity of http://www.guidetopharmacology.org/GRAC/LigandDisplayForward?ligandId=3944 at the AT_2_ receptor has also been reported [http://www.ncbi.nlm.nih.gov/pubmed/3071214?dopt=AbstractPlus]. The AT_1_ and bradykinin B_2_ receptors have been proposed to form a heterodimeric complex [http://www.ncbi.nlm.nih.gov/pubmed/10993080?dopt=AbstractPlus]^.^ β‐Arrestin1 prevents AT_1_‐B_2_ receptor heteromerization[http://www.ncbi.nlm.nih.gov/pubmed/30503206?dopt=AbstractPlus]. There is also evidence for an AT_4_ receptor that specifically binds http://www.guidetopharmacology.org/GRAC/LigandDisplayForward?ligandId=5368 (https://www.genenames.org/data/gene‐symbol‐report/#!/hgnc_id/HGNC:333, http://www.uniprot.org/uniprot/P01019) and is located in the brain and kidney. An additional putative endogenous ligand for the AT4 receptor has been described (http://www.guidetopharmacology.org/GRAC/LigandDisplayForward?ligandId=5353 (https://www.genenames.org/data/gene‐symbol‐report/#!/hgnc_id/HGNC:4827, http://www.uniprot.org/uniprot/P68871), a globin decapeptide) [http://www.ncbi.nlm.nih.gov/pubmed/9166749?dopt=AbstractPlus].

### Further reading on Angiotensin receptors

Asada H *et al*. (2018) Crystal structure of the human angiotensin II type 2 receptor bound to an angiotensin II analog. *Nat. Struct. Mol. Biol.* **25**: 570‐576 [https://www.ncbi.nlm.nih.gov/pubmed/29967536?dopt=AbstractPlus]

Karnik SS *et al*. (2015) International Union of Basic and Clinical Pharmacology. XCIX. Angiotensin Receptors: Interpreters of Pathophysiological Angiotensinergic Stimuli [corrected]. *Pharmacol. Rev.* **67**: 754‐819 [https://www.ncbi.nlm.nih.gov/pubmed/26315714?dopt=AbstractPlus]

Singh KD *et al*. (2019) Mechanism of Hormone Peptide Activation of a GPCR: Angiotensin II Activated State of AT_1R Initiated by van der Waals Attraction. *J Chem Inf Model* **59**: 373‐385 [https://www.ncbi.nlm.nih.gov/pubmed/30608150?dopt=AbstractPlus]

Wingler LM *et al*. (2019) Angiotensin Analogs with Divergent Bias Stabilize Distinct Receptor Conformations. *Cell* **176**: 468‐478.e11 [https://www.ncbi.nlm.nih.gov/pubmed/30639099?dopt=AbstractPlus]

Wingler LM *et al*. (2019) Distinctive Activation Mechanism for Angiotensin Receptor Revealed by a Synthetic Nanobody. *Cell* **176**: 479‐490.e12 [https://www.ncbi.nlm.nih.gov/pubmed/30639100?dopt=AbstractPlus]

Zhang H *et al*. (2015) Structure of the Angiotensin receptor revealed by serial femtosecond crystallography. *Cell* **161**: 833‐44 [https://www.ncbi.nlm.nih.gov/pubmed/25913193?dopt=AbstractPlus]

## http://www.guidetopharmacology.org/GRAC/FamilyDisplayForward?familyId=7

### Overview

The apelin receptor (**nomenclature as agreed by the NC‐IUPHAR Subcommittee on the apelin receptor** [http://www.ncbi.nlm.nih.gov/pubmed/20605969?dopt=AbstractPlus]) responds to apelin, a 36 amino‐acid peptide derived initially from bovine stomach. http://www.guidetopharmacology.org/GRAC/LigandDisplayForward?ligandId=606 (https://www.genenames.org/data/gene‐symbol‐report/#!/hgnc_id/HGNC:16665, http://www.uniprot.org/uniprot/Q9ULZ1), http://www.guidetopharmacology.org/GRAC/LigandDisplayForward?ligandId=605, http://www.uniprot.org/uniprot/Q9ULZ1) and [http://www.guidetopharmacology.org/GRAC/LigandDisplayForward?ligandId=599]http://www.guidetopharmacology.org/GRAC/LigandDisplayForward?ligandId=599 (https://www.genenames.org/data/gene‐symbol‐report/#!/hgnc_id/HGNC:16665, http://www.uniprot.org/uniprot/Q9ULZ1) are the predominant endogenous ligands which are cleaved from a 77 amino‐acid precursor peptide (https://www.genenames.org/data/gene‐symbol‐report/#!/hgnc_id/HGNC:16665 http://www.uniprot.org/uniprot/Q9ULZ1) by a so far unidentified enzymatic pathway [http://www.ncbi.nlm.nih.gov/pubmed/9792798?dopt=AbstractPlus]. A second family of peptides discovered independently and named Elabela [http://www.ncbi.nlm.nih.gov/pubmed/24316148?dopt=AbstractPlus] or Toddler, that has little sequence similarity to apelin, is present, and functional at the apelin receptor in the adult cardiovascular system [http://www.ncbi.nlm.nih.gov/pubmed/24407481?dopt=AbstractPlus, http://www.ncbi.nlm.nih.gov/pubmed/28137936?dopt=AbstractPlus]. Structure‐activity relationship Elabela analogues have been described [http://www.ncbi.nlm.nih.gov/pubmed/26986036?dopt=AbstractPlus].

**Table:** 

Nomenclature	http://www.guidetopharmacology.org/GRAC/ObjectDisplayForward?objectId=36
HGNC, UniProt	https://www.genenames.org/data/gene‐symbol‐report/#!/hgnc_id/HGNC:339, http://www.uniprot.org/uniprot/P35414
Potency order of endogenous ligands	[http://www.guidetopharmacology.org/GRAC/LigandDisplayForward?ligandId=599]http://www.guidetopharmacology.org/GRAC/LigandDisplayForward?ligandId=599 (https://www.genenames.org/data/gene‐symbol‐report/#!/hgnc_id/HGNC:16665, http://www.uniprot.org/uniprot/Q9ULZ1) ≥ http://www.guidetopharmacology.org/GRAC/LigandDisplayForward?ligandId=605 (https://www.genenames.org/data/gene‐symbol‐report/#!/hgnc_id/HGNC:16665, http://www.uniprot.org/uniprot/Q9ULZ1) > http://www.guidetopharmacology.org/GRAC/LigandDisplayForward?ligandId=606 (https://www.genenames.org/data/gene‐symbol‐report/#!/hgnc_id/HGNC:16665, http://www.uniprot.org/uniprot/Q9ULZ1) [http://www.ncbi.nlm.nih.gov/pubmed/12939143?dopt=AbstractPlus, http://www.ncbi.nlm.nih.gov/pubmed/9792798?dopt=AbstractPlus]
Endogenous agonists	http://www.guidetopharmacology.org/GRAC/LigandDisplayForward?ligandId=605 (https://www.genenames.org/data/gene‐symbol‐report/#!/hgnc_id/HGNC:16665, http://www.uniprot.org/uniprot/Q9ULZ1) [http://www.ncbi.nlm.nih.gov/pubmed/12939143?dopt=AbstractPlus, http://www.ncbi.nlm.nih.gov/pubmed/10777510?dopt=AbstractPlus, http://www.ncbi.nlm.nih.gov/pubmed/12603839?dopt=AbstractPlus], http://www.guidetopharmacology.org/GRAC/LigandDisplayForward?ligandId=7930 (https://www.genenames.org/data/gene‐symbol‐report/#!/hgnc_id/HGNC:48925, http://www.uniprot.org/uniprot/P0DMC3) [http://www.ncbi.nlm.nih.gov/pubmed/25995451?dopt=AbstractPlus], http://www.guidetopharmacology.org/GRAC/LigandDisplayForward?ligandId=3556 (https://www.genenames.org/data/gene‐symbol‐report/#!/hgnc_id/HGNC:16665, http://www.uniprot.org/uniprot/Q9ULZ1) [http://www.ncbi.nlm.nih.gov/pubmed/15341513?dopt=AbstractPlus, http://www.ncbi.nlm.nih.gov/pubmed/15341513?dopt=AbstractPlus], [http://www.guidetopharmacology.org/GRAC/LigandDisplayForward?ligandId=599]http://www.guidetopharmacology.org/GRAC/LigandDisplayForward?ligandId=599 (https://www.genenames.org/data/gene‐symbol‐report/#!/hgnc_id/HGNC:16665, http://www.uniprot.org/uniprot/Q9ULZ1) [http://www.ncbi.nlm.nih.gov/pubmed/11336787?dopt=AbstractPlus, http://www.ncbi.nlm.nih.gov/pubmed/11336787?dopt=AbstractPlus], http://www.guidetopharmacology.org/GRAC/LigandDisplayForward?ligandId=8525 (https://www.genenames.org/data/gene‐symbol‐report/#!/hgnc_id/HGNC:48925, http://www.uniprot.org/uniprot/P0DMC3) [2378], http://www.guidetopharmacology.org/GRAC/LigandDisplayForward?ligandId=8524 (https://www.genenames.org/data/gene‐symbol‐report/#!/hgnc_id/HGNC:48925, http://www.uniprot.org/uniprot/P0DMC3) [2378], http://www.guidetopharmacology.org/GRAC/LigandDisplayForward?ligandId=606 (https://www.genenames.org/data/gene‐symbol‐report/#!/hgnc_id/HGNC:16665, http://www.uniprot.org/uniprot/Q9ULZ1) [http://www.ncbi.nlm.nih.gov/pubmed/12939143?dopt=AbstractPlus, http://www.ncbi.nlm.nih.gov/pubmed/12939143?dopt=AbstractPlus, http://www.ncbi.nlm.nih.gov/pubmed/11336787?dopt=AbstractPlus, http://www.ncbi.nlm.nih.gov/pubmed/12603839?dopt=AbstractPlus], http://www.guidetopharmacology.org/GRAC/LigandDisplayForward?ligandId=8526 (https://www.genenames.org/data/gene‐symbol‐report/#!/hgnc_id/HGNC:48925, http://www.uniprot.org/uniprot/Q9ULZ1) [2378]
Selective agonists	http://www.guidetopharmacology.org/GRAC/LigandDisplayForward?ligandId=9448 (Biased agonist) [http://www.ncbi.nlm.nih.gov/pubmed/27475715?dopt=AbstractPlus], http://www.guidetopharmacology.org/GRAC/LigandDisplayForward?ligandId=8523 (Biased agonist) [http://www.ncbi.nlm.nih.gov/pubmed/25712721?dopt=AbstractPlus]
Antagonists	http://www.guidetopharmacology.org/GRAC/LigandDisplayForward?ligandId=6186 (p*K*_i_ 8.2) [http://www.ncbi.nlm.nih.gov/pubmed/21560248?dopt=AbstractPlus]
Labelled ligands	[http://www.guidetopharmacology.org/GRAC/LigandDisplayForward?ligandId=603,http://www.guidetopharmacology.org/GRAC/LigandDisplayForward?ligandId=603]http://www.guidetopharmacology.org/GRAC/LigandDisplayForward?ligandId=603) (Agonist) [http://www.ncbi.nlm.nih.gov/pubmed/11336787?dopt=AbstractPlus], [http://www.guidetopharmacology.org/GRAC/LigandDisplayForward?ligandId=3761,http://www.guidetopharmacology.org/GRAC/LigandDisplayForward?ligandId=3761]http://www.guidetopharmacology.org/GRAC/LigandDisplayForward?ligandId=3761 (Agonist) [http://www.ncbi.nlm.nih.gov/pubmed/10777510?dopt=AbstractPlus], [http://www.guidetopharmacology.org/GRAC/LigandDisplayForward?ligandId=601)http://www.guidetopharmacology.org/GRAC/LigandDisplayForward?ligandId=601 (Agonist) [http://www.ncbi.nlm.nih.gov/pubmed/11250876?dopt=AbstractPlus], [http://www.guidetopharmacology.org/GRAC/LigandDisplayForward?ligandId=602 (Agonist) [http://www.ncbi.nlm.nih.gov/pubmed/12939143?dopt=AbstractPlus], [http://www.guidetopharmacology.org/GRAC/LigandDisplayForward?ligandId=604)[http://www.guidetopharmacology.org/GRAC/LigandDisplayForward?ligandId=604 (Agonist) [http://www.ncbi.nlm.nih.gov/pubmed/12603839?dopt=AbstractPlus]

### Comments

Potency order determined for heterologously expressed human apelin receptor (p*D*_2_ values range from 9.5 to 8.6). The apelin receptor may also act as a co‐receptor with CD4 for isolates of human immunodeficiency virus, with apelin blocking this function [http://www.ncbi.nlm.nih.gov/pubmed/11090199?dopt=AbstractPlus]. A modified apelin‐13 peptide, http://www.guidetopharmacology.org/GRAC/LigandDisplayForward?ligandId=5354) was reported to block the hypotensive response to apelin in rat *in vivo* [http://www.ncbi.nlm.nih.gov/pubmed/15486224?dopt=AbstractPlus], however, this peptide exhibits agonist activity in HEK293 cells stably expressing the recombinant apelin receptor [http://www.ncbi.nlm.nih.gov/pubmed/12939143?dopt=AbstractPlus]. The apelin receptor antagonist, MM54, was reported to suppress tumour growth and increase survival in an intracranial xenograft mouse model of glioblastoma [http://www.ncbi.nlm.nih.gov/pubmed/29053791?dopt=AbstractPlus].

### Further reading on Apelin receptor

Cheng B *et al*. (2012) Neuroprotection of apelin and its signaling pathway. *Peptides* **37**: 171‐3 [https://www.ncbi.nlm.nih.gov/pubmed/22820556?dopt=AbstractPlus]

Langelaan DN *et al*. (2009) Structural insight into G‐protein coupled receptor binding by apelin. *Biochemistry* **48**: 537‐48 [https://www.ncbi.nlm.nih.gov/pubmed/19123778?dopt=AbstractPlus]

Mughal A *et al*. (2018) Vascular effects of apelin: Mechanisms and therapeutic potential. *Pharmacol. Ther.* **190**: 139‐147 [https://www.ncbi.nlm.nih.gov/pubmed/29807055?dopt=AbstractPlus]

O’Carroll AM *et al*. (2013) The apelin receptor APJ: journey from an orphan to a multifaceted regulator of homeostasis. *J. Endocrinol.* **219**: R13‐35 [https://www.ncbi.nlm.nih.gov/pubmed/23943882?dopt=AbstractPlus]

Pitkin SL *et al*. (2010) International Union of Basic and Clinical Pharmacology. LXXIV. Apelin receptor nomenclature, distribution, pharmacology, and function. *Pharmacol. Rev.* **62**: 331‐42 [https://www.ncbi.nlm.nih.gov/pubmed/20605969?dopt=AbstractPlus]

Yang P *et al*. (2015) Apelin, Elabela/Toddler, and biased agonists as novel therapeutic agents in the cardiovascular system. *Trends Pharmacol. Sci.* **36**: 560‐7 [https://www.ncbi.nlm.nih.gov/pubmed/26143239?dopt=AbstractPlus]

## http://www.guidetopharmacology.org/GRAC/FamilyDisplayForward?familyId=8

### Overview

The bile acid receptor (GPBA) responds to bile acids produced during the liver metabolism of http://www.guidetopharmacology.org/GRAC/LigandDisplayForward?ligandId=2718. Selective agonists are promising drugs for the treatment of metabolic disorders, such as type II diabetes, obesity and atherosclerosis.

**Table:** 

Nomenclature	http://www.guidetopharmacology.org/GRAC/ObjectDisplayForward?objectId=37
HGNC, UniProt	https://www.genenames.org/data/gene‐symbol‐report/#!/hgnc_id/HGNC:19680, http://www.uniprot.org/uniprot/Q8TDU6
Potency order of endogenous ligands	http://www.guidetopharmacology.org/GRAC/LigandDisplayForward?ligandId=611 > http://www.guidetopharmacology.org/GRAC/LigandDisplayForward?ligandId=610 > http://www.guidetopharmacology.org/GRAC/LigandDisplayForward?ligandId=608, http://www.guidetopharmacology.org/GRAC/LigandDisplayForward?ligandId=609 [http://www.ncbi.nlm.nih.gov/pubmed/12524422?dopt=AbstractPlus, http://www.ncbi.nlm.nih.gov/pubmed/12419312?dopt=AbstractPlus]
Selective agonists	http://www.guidetopharmacology.org/GRAC/LigandDisplayForward?ligandId=7048 [http://www.ncbi.nlm.nih.gov/pubmed/20014870?dopt=AbstractPlus] – Mouse, http://www.guidetopharmacology.org/GRAC/LigandDisplayForward?ligandId=3945 [http://www.ncbi.nlm.nih.gov/pubmed/19911773?dopt=AbstractPlus], http://www.guidetopharmacology.org/GRAC/LigandDisplayForward?ligandId=3306 [http://www.ncbi.nlm.nih.gov/pubmed/17825251?dopt=AbstractPlus]

### Comments

The triterpenoid natural product http://www.guidetopharmacology.org/GRAC/LigandDisplayForward?ligandId=3945 has also been reported to inhibit inflammatory signalling through the NF*κ*B pathway [http://www.ncbi.nlm.nih.gov/pubmed/12960358?dopt=AbstractPlus]. Disruption of GPBA expression is reported to protect from cholesterol gallstone formation [http://www.ncbi.nlm.nih.gov/pubmed/16724960?dopt=AbstractPlus]. A new series of 5‐phenoxy‐1,3‐dimethyl‐1H‐pyrazole‐4‐carboxamides have been reported as highly potent agonists [http://www.ncbi.nlm.nih.gov/pubmed/23337601?dopt=AbstractPlus].

### Further reading on Bile acid receptor

Duboc H *et al*. (2014) The bile acid TGR5 membrane receptor: from basic research to clinical application. *Dig Liver Dis* **46**: 302‐12 [https://www.ncbi.nlm.nih.gov/pubmed/24411485?dopt=AbstractPlus]

Lieu T *et al*. (2014) GPBA: a GPCR for bile acids and an emerging therapeutic target for disorders of digestion and sensation. *Br. J. Pharmacol.* **171**: 1156‐66 [https://www.ncbi.nlm.nih.gov/pubmed/24111923?dopt=AbstractPlus]

Lefebvre P *et al*. (2009) Role of bile acids and bile acid receptors in metabolic regulation. *Physiol. Rev.* **89**: 147‐91 [https://www.ncbi.nlm.nih.gov/pubmed/19126757?dopt=AbstractPlus]

van Nierop FS *et al*. (2017) Clinical relevance of the bile acid receptor TGR5 in metabolism. *Lancet Diabetes Endocrinol* **5**: 224‐233 [https://www.ncbi.nlm.nih.gov/pubmed/27639537?dopt=AbstractPlus]

## http://www.guidetopharmacology.org/GRAC/FamilyDisplayForward?familyId=9

### Overview

Mammalian bombesin (Bn) receptors comprise 3 subtypes: BB_1_, BB_2_, BB_3_ (**nomenclature recommended by the NC‐IUPHAR Subcommittee on bombesin receptors**, [http://www.ncbi.nlm.nih.gov/pubmed/18055507?dopt=AbstractPlus]). BB_1_ and BB_2_ are activated by the endogenous ligands http://www.guidetopharmacology.org/GRAC/LigandDisplayForward?ligandId=612 (https://www.genenames.org/data/gene‐symbol‐report/#!/hgnc_id/HGNC:4605, http://www.uniprot.org/uniprot/P07492) (GRP), http://www.guidetopharmacology.org/GRAC/LigandDisplayForward?ligandId=613 (https://www.genenames.org/data/gene‐symbol‐report/#!/hgnc_id/HGNC:7842, http://www.uniprot.org/uniprot/P08949) (NMB) and http://www.guidetopharmacology.org/GRAC/LigandDisplayForward?ligandId=3582) (https://www.genenames.org/data/gene‐symbol‐report/#!/hgnc_id/HGNC:4605, https://www.genenames.org/data/gene‐symbol‐report/#!/hgnc_id/HGNC:4605). http://www.guidetopharmacology.org/GRAC/LigandDisplayForward?ligandId=616 is a tetradecapeptide, originally derived from amphibians. The three Bn receptor subtypes couple primarily to the G_q/11_ and G_12_/13 family of G proteins [http://www.ncbi.nlm.nih.gov/pubmed/18055507?dopt=AbstractPlus]. Each of these receptors is widely distributed in the CNS and peripheral tissues [http://www.ncbi.nlm.nih.gov/pubmed/26066663?dopt=AbstractPlus, http://www.ncbi.nlm.nih.gov/pubmed/18055507?dopt=AbstractPlus, http://www.ncbi.nlm.nih.gov/pubmed/15726424?dopt=AbstractPlus, http://www.ncbi.nlm.nih.gov/pubmed/25976083?dopt=AbstractPlus, http://www.ncbi.nlm.nih.gov/pubmed/15203211?dopt=AbstractPlus, http://www.ncbi.nlm.nih.gov/pubmed/22911445?dopt=AbstractPlus]. Activation of BB_1_ and BB_2_ receptors causes a wide range of physiological/pathophysiogical actions, including the stimulation of normal and neoplastic tissue growth, smoothmuscle contraction, feeding behavior, secretion and many central nervous system effects including regulation of circadian rhythm and mediation of pruritus [http://www.ncbi.nlm.nih.gov/pubmed/18055507?dopt=AbstractPlus, 991, 992, http://www.ncbi.nlm.nih.gov/pubmed/21042212?dopt=AbstractPlus, http://www.ncbi.nlm.nih.gov/pubmed/15134870?dopt=AbstractPlus,http://www.ncbi.nlm.nih.gov/pubmed/25976083?dopt=AbstractPlus]. A physiological role for the BB_3_ receptor has yet to be fully defined although recently studies suggest an important role in glucose and insulin regulation, metabolic homeostasis, feeding, regulation of body temperature, obesity, diabetes mellitus and growth of normal/neoplastic tissues [http://www.ncbi.nlm.nih.gov/pubmed/26066663?dopt=AbstractPlus, http://www.ncbi.nlm.nih.gov/pubmed/22157398?dopt=AbstractPlus, http://www.ncbi.nlm.nih.gov/pubmed/9367152?dopt=AbstractPlus, http://www.ncbi.nlm.nih.gov/pubmed/27055378?dopt=AbstractPlus].

**Table:** 

Nomenclature	http://www.guidetopharmacology.org/GRAC/ObjectDisplayForward?objectId=38 http://www.guidetopharmacology.org/GRAC/ObjectDisplayForward?objectId=38	http://www.guidetopharmacology.org/GRAC/ObjectDisplayForward?objectId=39 http://www.guidetopharmacology.org/GRAC/ObjectDisplayForward?objectId=39	http://www.guidetopharmacology.org/GRAC/ObjectDisplayForward?objectId=40 http://www.guidetopharmacology.org/GRAC/ObjectDisplayForward?objectId=40
HGNC, UniProt	https://www.genenames.org/data/gene‐symbol‐report/#!/hgnc_id/HGNC:7843, http://www.uniprot.org/uniprot/P28336	https://www.genenames.org/data/gene‐symbol‐report/#!/hgnc_id/HGNC:4609, http://www.uniprot.org/uniprot/P30550	https://www.genenames.org/data/gene‐symbol‐report/#!/hgnc_id/HGNC:1113, http://www.uniprot.org/uniprot/P32247
Endogenous agonists	http://www.guidetopharmacology.org/GRAC/LigandDisplayForward?ligandId=613 (https://www.genenames.org/data/gene‐symbol‐report/#!/hgnc_id/HGNC:7842, http://www.uniprot.org/uniprot/P08949) [http://www.ncbi.nlm.nih.gov/pubmed/18055507?dopt=AbstractPlus, http://www.ncbi.nlm.nih.gov/pubmed/25976083?dopt=AbstractPlus, http://www.ncbi.nlm.nih.gov/pubmed/21729729?dopt=AbstractPlus]	http://www.guidetopharmacology.org/GRAC/LigandDisplayForward?ligandId=6178 [http://www.ncbi.nlm.nih.gov/pubmed/21729729?dopt=AbstractPlus], http://www.guidetopharmacology.org/GRAC/LigandDisplayForward?ligandId=6175]	–
Selective agonists	–	–	http://www.guidetopharmacology.org/GRAC/LigandDisplayForward?ligandId=8509 [http://www.ncbi.nlm.nih.gov/pubmed/24412111?dopt=AbstractPlus], http://www.guidetopharmacology.org/GRAC/LigandDisplayForward?ligandId=8502 [http://www.ncbi.nlm.nih.gov/pubmed/24900461?dopt=AbstractPlus], http://www.guidetopharmacology.org/GRAC/LigandDisplayForward?ligandId=6170 [http://www.ncbi.nlm.nih.gov/pubmed/23892571?dopt=AbstractPlus, http://www.ncbi.nlm.nih.gov/pubmed/24900253?dopt=AbstractPlus], [http://www.guidetopharmacology.org/GRAC/LigandDisplayForward?ligandId=3946,http://www.guidetopharmacology.org/GRAC/LigandDisplayForward?ligandId=3946,http://www.guidetopharmacology.org/GRAC/LigandDisplayForward?ligandId=3946,http://www.guidetopharmacology.org/GRAC/LigandDisplayForward?ligandId=3946]http://www.guidetopharmacology.org/GRAC/LigandDisplayForward?ligandId=3946) [http://www.ncbi.nlm.nih.gov/pubmed/15102928?dopt=AbstractPlus], http://www.guidetopharmacology.org/GRAC/LigandDisplayForward?ligandId=8507 [http://www.ncbi.nlm.nih.gov/pubmed/25497965?dopt=AbstractPlus], http://www.guidetopharmacology.org/GRAC/LigandDisplayForward?ligandId=6188 [http://www.ncbi.nlm.nih.gov/pubmed/20096642?dopt=AbstractPlus], http://www.guidetopharmacology.org/GRAC/LigandDisplayForward?ligandId=8504 [http://www.ncbi.nlm.nih.gov/pubmed/20167483?dopt=AbstractPlus]
Antagonists	http://www.guidetopharmacology.org/GRAC/LigandDisplayForward?ligandId=635 (pIC_50_ 6.2–6.6) [http://www.ncbi.nlm.nih.gov/pubmed/19463875?dopt=AbstractPlus]	–	–
Selective antagonists	http://www.guidetopharmacology.org/GRAC/LigandDisplayForward?ligandId=626 (pIC_50_ 9.3–9.8) [http://www.ncbi.nlm.nih.gov/pubmed/19463875?dopt=AbstractPlus], http://www.guidetopharmacology.org/GRAC/LigandDisplayForward?ligandId=621 (pIC_50_ 9.3–9.6) [http://www.ncbi.nlm.nih.gov/pubmed/19463875?dopt=AbstractPlus], http://www.guidetopharmacology.org/GRAC/LigandDisplayForward?ligandId=3871(http://www.guidetopharmacology.org/GRAC/LigandDisplayForward?ligandId=3871	[http://www.guidetopharmacology.org/GRAC/LigandDisplayForward?ligandId=3840, http://www.guidetopharmacology.org/GRAC/LigandDisplayForward?ligandId=3840, http://www.guidetopharmacology.org/GRAC/LigandDisplayForward?ligandId=3840,http://www.guidetopharmacology.org/GRAC/LigandDisplayForward?ligandId=3840) (p*K*_i_ 9.8) [http://www.ncbi.nlm.nih.gov/pubmed/19463875?dopt=AbstractPlus], http://www.guidetopharmacology.org/GRAC/LigandDisplayForward?ligandId=3889 (pIC_50_ 9.3) [http://www.ncbi.nlm.nih.gov/pubmed/11463790?dopt=AbstractPlus] – Mouse, [(http://www.guidetopharmacology.org/GRAC/LigandDisplayForward?ligandId=627), http://www.guidetopharmacology.org/GRAC/LigandDisplayForward?ligandId=627,http://www.guidetopharmacology.org/GRAC/LigandDisplayForward?ligandId=627,http://www.guidetopharmacology.org/GRAC/LigandDisplayForward?ligandId=627,http://www.guidetopharmacology.org/GRAC/LigandDisplayForward?ligandId=627]http://www.guidetopharmacology.org/GRAC/LigandDisplayForward?ligandId=627‐( (pIC_50_ 9.2) [http://www.ncbi.nlm.nih.gov/pubmed/19463875?dopt=AbstractPlus, http://www.ncbi.nlm.nih.gov/pubmed/8446610?dopt=AbstractPlus], http://www.guidetopharmacology.org/GRAC/LigandDisplayForward?ligandId=624 (pIC_50_ 8.9) [http://www.ncbi.nlm.nih.gov/pubmed/10231715?dopt=AbstractPlus] – Mouse, [http://www.guidetopharmacology.org/GRAC/LigandDisplayForward?ligandId=6183, http://www.guidetopharmacology.org/GRAC/LigandDisplayForward?ligandId=6183 http://www.guidetopharmacology.org/GRAC/LigandDisplayForward?ligandId=6183) (pIC_50_ 8.9) [http://www.ncbi.nlm.nih.gov/pubmed/19463875?dopt=AbstractPlus]	http://www.guidetopharmacology.org/GRAC/LigandDisplayForward?ligandId=6176 (pIC_50_ 8.6–8.7) [http://www.ncbi.nlm.nih.gov/pubmed/20096642?dopt=AbstractPlus, http://www.ncbi.nlm.nih.gov/pubmed/23892571?dopt=AbstractPlus], http://www.guidetopharmacology.org/GRAC/LigandDisplayForward?ligandId=8505 (pIC_50_ 5.3) [http://www.ncbi.nlm.nih.gov/pubmed/25554218?dopt=AbstractPlus] http://www.guidetopharmacology.org/GRAC/LigandDisplayForward?ligandId=627)
Labelled ligands	[http://www.guidetopharmacology.org/GRAC/LigandDisplayForward?ligandId=3771) (Agonist), [http://www.guidetopharmacology.org/GRAC/LigandDisplayForward?ligandId=623]http://www.guidetopharmacology.org/GRAC/LigandDisplayForward?ligandId=623 (Agonist)	[http://www.guidetopharmacology.org/GRAC/LigandDisplayForward?ligandId=628]http://www.guidetopharmacology.org/GRAC/LigandDisplayForward?ligandId=628 (Selective Antagonist) (p*K*_d_ 9.3) [http://www.ncbi.nlm.nih.gov/pubmed/7684815?dopt=AbstractPlus] – Mouse, [http://www.guidetopharmacology.org/GRAC/LigandDisplayForward?ligandId=623]http://www.guidetopharmacology.org/GRAC/LigandDisplayForward?ligandId=623 (Agonist) [http://www.ncbi.nlm.nih.gov/pubmed/7838118?dopt=AbstractPlus], [http://www.guidetopharmacology.org/GRAC/LigandDisplayForward?ligandId=3788) (Agonist)	[http://www.guidetopharmacology.org/GRAC/LigandDisplayForward?ligandId=8506 (Agonist) [http://www.ncbi.nlm.nih.gov/pubmed/20096642?dopt=AbstractPlus] – Mouse, [http://www.guidetopharmacology.org/GRAC/LigandDisplayForward?ligandId=631,http://www.guidetopharmacology.org/GRAC/LigandDisplayForward?ligandId=631‐http://www.guidetopharmacology.org/GRAC/LigandDisplayForward?ligandId=631,http://www.guidetopharmacology.org/GRAC/LigandDisplayForward?ligandId=631,http://www.guidetopharmacology.org/GRAC/LigandDisplayForward?ligandId=631]http://www.guidetopharmacology.org/GRAC/LigandDisplayForward?ligandId=631) (Agonist) [http://www.ncbi.nlm.nih.gov/pubmed/9325344?dopt=AbstractPlus, http://www.ncbi.nlm.nih.gov/pubmed/23892571?dopt=AbstractPlus]

### Comments

All three human subtypes may be activated by [http://www.guidetopharmacology.org/GRAC/LigandDisplayForward?ligandId=632,http://www.guidetopharmacology.org/GRAC/LigandDisplayForward?ligandId=632‐http://www.guidetopharmacology.org/GRAC/LigandDisplayForward?ligandId=632,http://www.guidetopharmacology.org/GRAC/LigandDisplayForward?ligandId=632,http://www.guidetopharmacology.org/GRAC/LigandDisplayForward?ligandId=632]http://www.guidetopharmacology.org/GRAC/LigandDisplayForward?ligandId=632) [http://www.ncbi.nlm.nih.gov/pubmed/9325344?dopt=AbstractPlus]. [http://www.guidetopharmacology.org/GRAC/LigandDisplayForward?ligandId=3946,http://www.guidetopharmacology.org/GRAC/LigandDisplayForward?ligandId=3946,http://www.guidetopharmacology.org/GRAC/LigandDisplayForward?ligandId=3946,http://www.guidetopharmacology.org/GRAC/LigandDisplayForward?ligandId=3946]http://www.guidetopharmacology.org/GRAC/LigandDisplayForward?ligandId=3946) has more than 200‐fold selectivity for BB_3_ receptors over BB_1_ and BB_2_ [http://www.ncbi.nlm.nih.gov/pubmed/15102928?dopt=AbstractPlus, http://www.ncbi.nlm.nih.gov/pubmed/15102928?dopt=AbstractPlus, http://www.ncbi.nlm.nih.gov/pubmed/25976083?dopt=AbstractPlus, http://www.ncbi.nlm.nih.gov/pubmed/26524625?dopt=AbstractPlus].

### Further reading on Bombesin receptors

González N *et al*. (2015) Bombesin receptor subtype 3 as a potential target for obesity and diabetes. *Expert Opin. Ther. Targets* **19**: 1153‐70 [https://www.ncbi.nlm.nih.gov/pubmed/26066663?dopt=AbstractPlus]

Jensen RT *et al*. (2008) International Union of Pharmacology. LXVIII. Mammalian bombesin receptors: nomenclature, distribution, pharmacology, signaling, and functions in normal and disease states. *Pharmacol. Rev.* **60**: 1‐42 [https://www.ncbi.nlm.nih.gov/pubmed/18055507?dopt=AbstractPlus]

Maina T *et al*. (2017) Theranostic Prospects of Gastrin‐Releasing Peptide Receptor‐Radioantagonists in Oncology. *PET Clin* **12**: 297‐309 [https://www.ncbi.nlm.nih.gov/pubmed/28576168?dopt=AbstractPlus]

Moreno P *et al*. (2016) Bombesin related peptides/receptors and their promising therapeutic roles in cancer imaging, targeting and treatment. *Expert Opin. Ther. Targets* **20**: 1055‐73 [https://www.ncbi.nlm.nih.gov/pubmed/26981612?dopt=AbstractPlus]

Qu X *et al*. (2018) Recent insights into biological functions of mammalian bombesin‐like peptides and their receptors. *Curr Opin Endocrinol Diabetes Obes* **25**: 36‐41 [https://www.ncbi.nlm.nih.gov/pubmed/29120926?dopt=AbstractPlus]

Ramos‐Álvarez I *et al*. (2015) Insights into bombesin receptors and ligands: Highlighting recent advances. *Peptides* **72**: 128‐44 [https://www.ncbi.nlm.nih.gov/pubmed/25976083?dopt=AbstractPlus]

## http://www.guidetopharmacology.org/GRAC/FamilyDisplayForward?familyId=10

### Overview

Bradykinin (or kinin) receptors (**nomenclature as agreed by the NC‐IUPHAR subcommittee on Bradykinin (kinin) Receptors** [http://www.ncbi.nlm.nih.gov/pubmed/15734727?dopt=AbstractPlus]) are activated by the endogenous peptides http://www.guidetopharmacology.org/GRAC/LigandDisplayForward?ligandId=649 (https://www.genenames.org/data/gene‐symbol‐report/#!/hgnc_id/HGNC:6383, http://www.uniprot.org/uniprot/P01042) (BK), [http://www.guidetopharmacology.org/GRAC/LigandDisplayForward?ligandId=646]http://www.guidetopharmacology.org/GRAC/LigandDisplayForward?ligandId=646, https://www.genenames.org/data/gene‐symbol‐report/#!/hgnc_id/HGNC:6383), Lys‐BK (http://www.guidetopharmacology.org/GRAC/LigandDisplayForward?ligandId=650, https://www.genenames.org/data/gene‐symbol‐report/#!/hgnc_id/HGNC:6383)), [http://www.guidetopharmacology.org/GRAC/LigandDisplayForward?ligandId=644]http://www.guidetopharmacology.org/GRAC/LigandDisplayForward?ligandId=644, https://www.genenames.org/data/gene‐symbol‐report/#!/hgnc_id/HGNC:6383), [Phospho‐Ser6]‐Bradykinin, http://www.guidetopharmacology.org/GRAC/LigandDisplayForward?ligandId=639, https://www.genenames.org/data/gene‐symbol‐report/#!/hgnc_id/HGNC:6383) (Ile‐SerBK), [http://www.guidetopharmacology.org/GRAC/LigandDisplayForward?ligandId=3578]http://www.guidetopharmacology.org/GRAC/LigandDisplayForward?ligandId=3578, https://www.genenames.org/data/gene‐symbol‐report/#!/hgnc_id/HGNC:6383) and http://www.guidetopharmacology.org/GRAC/LigandDisplayForward?ligandId=3580]‐http://www.guidetopharmacology.org/GRAC/LigandDisplayForward?ligandId=3580, https://www.genenames.org/data/gene‐symbol‐report/#!/hgnc_id/HGNC:6383). Variation in pharmacology and activity of B_1_ and B_2_ receptor antagonists at species orthologs has been documented. http://www.guidetopharmacology.org/GRAC/LigandDisplayForward?ligandId=667 (Hoe140, Firazir) is approved in North America and Europe for the treatment of acute attacks of hereditary angioedema.

**Table:** 

Nomenclature	http://www.guidetopharmacology.org/GRAC/ObjectDisplayForward?objectId=41 http://www.guidetopharmacology.org/GRAC/ObjectDisplayForward?objectId=41	http://www.guidetopharmacology.org/GRAC/ObjectDisplayForward?objectId=42 http://www.guidetopharmacology.org/GRAC/ObjectDisplayForward?objectId=42
HGNC, UniProt	https://www.genenames.org/data/gene‐symbol‐report/#!/hgnc_id/HGNC:1029, http://www.uniprot.org/uniprot/P46663	https://www.genenames.org/data/gene‐symbol‐report/#!/hgnc_id/HGNC:1030, http://www.uniprot.org/uniprot/P30411
Potency order of endogenous ligands	[http://www.guidetopharmacology.org/GRAC/LigandDisplayForward?ligandId=644]http://www.guidetopharmacology.org/GRAC/LigandDisplayForward?ligandId=644 (https://www.genenames.org/data/gene‐symbol‐report/#!/hgnc_id/HGNC:6383, http://www.uniprot.org/uniprot/P01042) > [http://www.guidetopharmacology.org/GRAC/LigandDisplayForward?ligandId=646]http://www.guidetopharmacology.org/GRAC/LigandDisplayForward?ligandId=646, https://www.genenames.org/data/gene‐symbol‐report/#!/hgnc_id/HGNC:6383) = http://www.guidetopharmacology.org/GRAC/LigandDisplayForward?ligandId=650, https://www.genenames.org/data/gene‐symbol‐report/#!/hgnc_id/HGNC:6383) > http://www.guidetopharmacology.org/GRAC/LigandDisplayForward?ligandId=649, https://www.genenames.org/data/gene‐symbol‐report/#!/hgnc_id/HGNC:6383)	http://www.guidetopharmacology.org/GRAC/LigandDisplayForward?ligandId=650 (https://www.genenames.org/data/gene‐symbol‐report/#!/hgnc_id/HGNC:6383, http://www.uniprot.org/uniprot/P01042) > http://www.guidetopharmacology.org/GRAC/LigandDisplayForward?ligandId=649, https://www.genenames.org/data/gene‐symbol‐report/#!/hgnc_id/HGNC:6383) [http://www.guidetopharmacology.org/GRAC/LigandDisplayForward?ligandId=646]http://www.guidetopharmacology.org/GRAC/LigandDisplayForward?ligandId=646, https://www.genenames.org/data/gene‐symbol‐report/#!/hgnc_id/HGNC:6383), [http://www.guidetopharmacology.org/GRAC/LigandDisplayForward?ligandId=644]http://www.guidetopharmacology.org/GRAC/LigandDisplayForward?ligandId=644, https://www.genenames.org/data/gene‐symbol‐report/#!/hgnc_id/HGNC:6383)
Endogenous agonists	[http://www.guidetopharmacology.org/GRAC/LigandDisplayForward?ligandId=644]http://www.guidetopharmacology.org/GRAC/LigandDisplayForward?ligandId=644 (https://www.genenames.org/data/gene‐symbol‐report/#!/hgnc_id/HGNC:6383, http://www.uniprot.org/uniprot/P01042) [http://www.ncbi.nlm.nih.gov/pubmed/9111052?dopt=AbstractPlus, http://www.ncbi.nlm.nih.gov/pubmed/9313952?dopt=AbstractPlus, http://www.ncbi.nlm.nih.gov/pubmed/8735629?dopt=AbstractPlus, http://www.ncbi.nlm.nih.gov/pubmed/10422787?dopt=AbstractPlus]	http://www.guidetopharmacology.org/GRAC/LigandDisplayForward?ligandId=649 (https://www.genenames.org/data/gene‐symbol‐report/#!/hgnc_id/HGNC:6383, http://www.uniprot.org/uniprot/P01042)
Selective agonists	http://www.guidetopharmacology.org/GRAC/LigandDisplayForward?ligandId=10254 [http://www.ncbi.nlm.nih.gov/pubmed/26565554?dopt=AbstractPlus], [http://www.guidetopharmacology.org/GRAC/LigandDisplayForward?ligandId=643,http://www.guidetopharmacology.org/GRAC/LigandDisplayForward?ligandId=643]http://www.guidetopharmacology.org/GRAC/LigandDisplayForward?ligandId=643 [http://www.ncbi.nlm.nih.gov/pubmed/10422787?dopt=AbstractPlus]	http://www.guidetopharmacology.org/GRAC/LigandDisplayForward?ligandId=10255 [http://www.ncbi.nlm.nih.gov/pubmed/19111586?dopt=AbstractPlus, http://www.ncbi.nlm.nih.gov/pubmed/23362191?dopt=AbstractPlus], [http://www.guidetopharmacology.org/GRAC/LigandDisplayForward?ligandId=3841,http://www.guidetopharmacology.org/GRAC/LigandDisplayForward?ligandId=3841)http://www.guidetopharmacology.org/GRAC/LigandDisplayForward?ligandId=3841]http://www.guidetopharmacology.org/GRAC/LigandDisplayForward?ligandId=3841, [http://www.guidetopharmacology.org/GRAC/LigandDisplayForward?ligandId=3846,http://www.guidetopharmacology.org/GRAC/LigandDisplayForward?ligandId=3846(http://www.guidetopharmacology.org/GRAC/LigandDisplayForward?ligandId=3846‐http://www.guidetopharmacology.org/GRAC/LigandDisplayForward?ligandId=3846]http://www.guidetopharmacology.org/GRAC/LigandDisplayForward?ligandId=3846, http://www.guidetopharmacology.org/GRAC/LigandDisplayForward?ligandId=672, http://www.ncbi.nlm.nih.gov/pubmed/19111586?dopt=AbstractPlus]
Selective antagonists	http://www.guidetopharmacology.org/GRAC/LigandDisplayForward?ligandId=655 (p*K*_i_ 9.2–10.3) [http://www.ncbi.nlm.nih.gov/pubmed/16368899?dopt=AbstractPlus, http://www.ncbi.nlm.nih.gov/pubmed/9650825?dopt=AbstractPlus], [http://www.guidetopharmacology.org/GRAC/LigandDisplayForward?ligandId=642,http://www.guidetopharmacology.org/GRAC/LigandDisplayForward?ligandId=642]http://www.guidetopharmacology.org/GRAC/LigandDisplayForward?ligandId=642 (p*K*_i_ 9.1–9.3) [http://www.ncbi.nlm.nih.gov/pubmed/9111052?dopt=AbstractPlus, http://www.ncbi.nlm.nih.gov/pubmed/9313952?dopt=AbstractPlus], http://www.guidetopharmacology.org/GRAC/LigandDisplayForward?ligandId=662 (p*K*_i_ 9.1–9.2) [http://www.ncbi.nlm.nih.gov/pubmed/14747609?dopt=AbstractPlus], http://www.guidetopharmacology.org/GRAC/LigandDisplayForward?ligandId=3906 (p*A*_2_ 8.6) [http://www.ncbi.nlm.nih.gov/pubmed/24361511?dopt=AbstractPlus], http://www.guidetopharmacology.org/GRAC/LigandDisplayForward?ligandId=661 (p*A*_2_ 8.5) [http://www.ncbi.nlm.nih.gov/pubmed/8901831?dopt=AbstractPlus]	http://www.guidetopharmacology.org/GRAC/LigandDisplayForward?ligandId=667 (p*K*_i_ 10.2) [http://www.ncbi.nlm.nih.gov/pubmed/10514288?dopt=AbstractPlus], http://www.guidetopharmacology.org/GRAC/LigandDisplayForward?ligandId=674 (p*A*_2_ 8.2) [http://www.ncbi.nlm.nih.gov/pubmed/9095082?dopt=AbstractPlus], http://www.guidetopharmacology.org/GRAC/LigandDisplayForward?ligandId=679 (p*K*_i_ 8.2) [http://www.ncbi.nlm.nih.gov/pubmed/10596852?dopt=AbstractPlus]
Labelled ligands	[http://www.guidetopharmacology.org/GRAC/LigandDisplayForward?ligandId=3791 (p*K*_d_ 10), [http://www.guidetopharmacology.org/GRAC/LigandDisplayForward?ligandId=3825]http://www.guidetopharmacology.org/GRAC/LigandDisplayForward?ligandId=3825 (Agonist), [http://www.guidetopharmacology.org/GRAC/LigandDisplayForward?ligandId=3826][http://www.guidetopharmacology.org/GRAC/LigandDisplayForward?ligandId=3826]http://www.guidetopharmacology.org/GRAC/LigandDisplayForward?ligandId=3826 (Antagonist)	[http://www.guidetopharmacology.org/GRAC/LigandDisplayForward?ligandId=3812) (Agonist) [http://www.ncbi.nlm.nih.gov/pubmed/9333122?dopt=AbstractPlus] – Mouse, [http://www.guidetopharmacology.org/GRAC/LigandDisplayForward?ligandId=3831 (Antagonist) (p*K*_d_ 9.1–9.4) [http://www.ncbi.nlm.nih.gov/pubmed/9651119?dopt=AbstractPlus, http://www.ncbi.nlm.nih.gov/pubmed/11379050?dopt=AbstractPlus], [http://www.guidetopharmacology.org/GRAC/LigandDisplayForward?ligandId=3765]http://www.guidetopharmacology.org/GRAC/LigandDisplayForward?ligandId=3765 (Agonist)

### Further reading on Bradykinin receptors

Campos MM *et al*. (2006) Non‐peptide antagonists for kinin B1 receptors: new insights into their therapeutic potential for the management of inflammation and pain. *Trends Pharmacol. Sci.* **27**: 646‐51 [https://www.ncbi.nlm.nih.gov/pubmed/17056130?dopt=AbstractPlus]

Duchene J *et al*. (2009) The kinin B(1) receptor and inflammation: new therapeutic target for cardiovascular disease. *Curr Opin Pharmacol* **9**: 125‐31 [https://www.ncbi.nlm.nih.gov/pubmed/19124274?dopt=AbstractPlus]

Marceau F *et al*. (2004) Bradykinin receptor ligands: therapeutic perspectives. *Nat Rev Drug Discov* **3**: 845‐52 [https://www.ncbi.nlm.nih.gov/pubmed/15459675?dopt=AbstractPlus]

Paquet JL *et al*. (1999) Pharmacological characterization of the bradykinin B2 receptor: inter‐species variability and dissociation between binding and functional responses. *Br. J. Pharmacol.* **126**: 1083‐90 [https://www.ncbi.nlm.nih.gov/pubmed/10204994?dopt=AbstractPlus]

Thornton E *et al*. (2010) Kinin receptor antagonists as potential neuroprotective agents in central nervous system injury. *Molecules* **15**: 6598‐618 [https://www.ncbi.nlm.nih.gov/pubmed/20877247?dopt=AbstractPlus]

Whalley ET *et al*. (2012) Discovery and therapeutic potential of kinin receptor antagonists. *Expert Opin Drug Discov* **7**: 1129‐48 [https://www.ncbi.nlm.nih.gov/pubmed/23095011?dopt=AbstractPlus]

## http://www.guidetopharmacology.org/GRAC/FamilyDisplayForward?familyId=11

### Overview

This receptor family comprises a group of receptors for the calcitonin/CGRP family of peptides. The calcitonin (CT), amylin (AMY), calcitonin gene‐related peptide (CGRP) and adrenomedullin (AM) receptors (**nomenclature as agreed by the NC‐IUPHAR Subcommittee on CGRP, AM, AMY, and CT receptors** [http://www.ncbi.nlm.nih.gov/pubmed/18552275?dopt=AbstractPlus, http://www.ncbi.nlm.nih.gov/pubmed/12037140?dopt=AbstractPlus]) are generated by the genes https://www.genenames.org/data/gene‐symbol‐report/#!/hgnc_id/HGNC:1440 (which codes for the CT receptor) and https://www.genenames.org/data/gene‐symbol‐report/#!/hgnc_id/HGNC:16709 (which codes for the calcitonin receptor‐like receptor, CLR, previously known as CRLR). Their function and pharmacology are altered in the presence of RAMPs (receptor activity‐modifying proteins), which are single TM domain proteins of *ca.* 130 amino acids, identified as a family of three members; RAMP1, RAMP2 and RAMP3. There are splice variants of the CT receptor; these in turn produce variants of the AMY receptor [http://www.ncbi.nlm.nih.gov/pubmed/12037140?dopt=AbstractPlus], some of which can be potently activated by CGRP. The endogenous agonists are the peptides http://www.guidetopharmacology.org/GRAC/LigandDisplayForward?ligandId=685 (https://www.genenames.org/data/gene‐symbol‐report/#!/hgnc_id/HGNC:1437, http://www.uniprot.org/uniprot/P01258), http://www.guidetopharmacology.org/GRAC/LigandDisplayForward?ligandId=681‐http://www.guidetopharmacology.org/GRAC/LigandDisplayForward?ligandId=681, http://www.uniprot.org/uniprot/P06881) (formerly known as CGRP‐I), http://www.guidetopharmacology.org/GRAC/LigandDisplayForward?ligandId=682 (https://www.genenames.org/data/gene‐symbol‐report/#!/hgnc_id/HGNC:1438, http://www.uniprot.org/uniprot/P10092) (formerly known as CGRP‐II), http://www.guidetopharmacology.org/GRAC/LigandDisplayForward?ligandId=687 (https://www.genenames.org/data/gene‐symbol‐report/#!/hgnc_id/HGNC:5329, http://www.uniprot.org/uniprot/P10997) (occasionally called islet‐amyloid polypeptide, diabetes‐associated polypeptide), http://www.guidetopharmacology.org/GRAC/LigandDisplayForward?ligandId=683 (https://www.genenames.org/data/gene‐symbol‐report/#!/hgnc_id/HGNC:259, http://www.uniprot.org/uniprot/P35318) and http://www.guidetopharmacology.org/GRAC/LigandDisplayForward?ligandId=684 (https://www.genenames.org/data/gene‐symbol‐report/#!/hgnc_id/HGNC:28898, http://www.uniprot.org/uniprot/Q7Z4H4). There are species differences in peptide sequences, particularly for the CTs. http://www.guidetopharmacology.org/GRAC/LigandDisplayForward?ligandId=5369 {Pig} (CRSP) is another member of the family with selectivity for the CT receptor but it is not expressed in humans [http://www.ncbi.nlm.nih.gov/pubmed/12556539?dopt=AbstractPlus]. http://www.guidetopharmacology.org/GRAC/LigandDisplayForward?ligandId=702 (also known as BIBN4096BS, *p*Ki10.5) and http://www.guidetopharmacology.org/GRAC/LigandDisplayForward?ligandId=703 (also known as MK0974, *p*Ki9) are the most selective antagonists available, showing selectivity for CGRP receptors, with a particular preference for those of primate origin. CLR (calcitonin receptor‐like receptor) by itself binds no known endogenous ligand, but in the presence of RAMPs it gives receptors for CGRP, adrenomedullin and adrenomedullin 2/intermedin.

**Table:** 

Nomenclature	http://www.guidetopharmacology.org/GRAC/ObjectDisplayForward?objectId=43	http://www.guidetopharmacology.org/GRAC/ObjectDisplayForward?objectId=44	http://www.guidetopharmacology.org/GRAC/ObjectDisplayForward?objectId=45	http://www.guidetopharmacology.org/GRAC/ObjectDisplayForward?objectId=46
HGNC, UniProt	https://www.genenames.org/data/gene‐symbol‐report/#!/hgnc_id/HGNC:1440, http://www.uniprot.org/uniprot/P30988	–	–	–
Subunits	–	http://www.guidetopharmacology.org/GRAC/ObjectDisplayForward?objectId=51 (Accessory protein), http://www.guidetopharmacology.org/GRAC/ObjectDisplayForward?objectId=43	http://www.guidetopharmacology.org/GRAC/ObjectDisplayForward?objectId=43, http://www.guidetopharmacology.org/GRAC/ObjectDisplayForward?objectId=52 (Accessory protein)	http://www.guidetopharmacology.org/GRAC/ObjectDisplayForward?objectId=43, http://www.guidetopharmacology.org/GRAC/ObjectDisplayForward?objectId=53 (Accessory protein)
Potency order of endogenous ligands	http://www.guidetopharmacology.org/GRAC/LigandDisplayForward?ligandId=6973) ≥ http://www.guidetopharmacology.org/GRAC/LigandDisplayForward?ligandId=685 (https://www.genenames.org/data/gene‐symbol‐report/#!/hgnc_id/HGNC:1437, http://www.uniprot.org/uniprot/P01258) ≥ http://www.guidetopharmacology.org/GRAC/LigandDisplayForward?ligandId=687 (https://www.genenames.org/data/gene‐symbol‐report/#!/hgnc_id/HGNC:5329, http://www.uniprot.org/uniprot/P10997), http://www.guidetopharmacology.org/GRAC/LigandDisplayForward?ligandId=681‐http://www.guidetopharmacology.org/GRAC/LigandDisplayForward?ligandId=681, http://www.uniprot.org/uniprot/P06881), http://www.guidetopharmacology.org/GRAC/LigandDisplayForward?ligandId=682 (https://www.genenames.org/data/gene‐symbol‐report/#!/hgnc_id/HGNC:1438, http://www.uniprot.org/uniprot/P10092) > http://www.guidetopharmacology.org/GRAC/LigandDisplayForward?ligandId=683 (https://www.genenames.org/data/gene‐symbol‐report/#!/hgnc_id/HGNC:259, http://www.uniprot.org/uniprot/P35318), http://www.guidetopharmacology.org/GRAC/LigandDisplayForward?ligandId=684 (https://www.genenames.org/data/gene‐symbol‐report/#!/hgnc_id/HGNC:28898, http://www.uniprot.org/uniprot/Q7Z4H4)	http://www.guidetopharmacology.org/GRAC/LigandDisplayForward?ligandId=6973) ≥ http://www.guidetopharmacology.org/GRAC/LigandDisplayForward?ligandId=687 (https://www.genenames.org/data/gene‐symbol‐report/#!/hgnc_id/HGNC:5329, http://www.uniprot.org/uniprot/P10997) ≥http://www.guidetopharmacology.org/GRAC/LigandDisplayForward?ligandId=681 (https://www.genenames.org/data/gene‐symbol‐report/#!/hgnc_id/HGNC:1437, http://www.uniprot.org/uniprot/P06881), http://www.guidetopharmacology.org/GRAC/LigandDisplayForward?ligandId=682‐http://www.guidetopharmacology.org/GRAC/LigandDisplayForward?ligandId=682 (https://www.genenames.org/data/gene‐symbol‐report/#!/hgnc_id/HGNC:1438, http://www.uniprot.org/uniprot/P10092) > http://www.guidetopharmacology.org/GRAC/LigandDisplayForward?ligandId=684 (https://www.genenames.org/data/gene‐symbol‐report/#!/hgnc_id/HGNC:28898, http://www.uniprot.org/uniprot/Q7Z4H4) ≥ http://www.guidetopharmacology.org/GRAC/LigandDisplayForward?ligandId=685, http://www.uniprot.org/uniprot/P01258) > http://www.guidetopharmacology.org/GRAC/LigandDisplayForward?ligandId=683 (https://www.genenames.org/data/gene‐symbol‐report/#!/hgnc_id/HGNC:259, http://www.uniprot.org/uniprot/P35318)	Poorly defined	http://www.guidetopharmacology.org/GRAC/LigandDisplayForward?ligandId=6973) ≥ http://www.guidetopharmacology.org/GRAC/LigandDisplayForward?ligandId=687 (https://www.genenames.org/data/gene‐symbol‐report/#!/hgnc_id/HGNC:5329, http://www.uniprot.org/uniprot/P10997) > http://www.guidetopharmacology.org/GRAC/LigandDisplayForward?ligandId=681 (https://www.genenames.org/data/gene‐symbol‐report/#!/hgnc_id/HGNC:1437, http://www.uniprot.org/uniprot/P06881),http://www.guidetopharmacology.org/GRAC/LigandDisplayForward?ligandId=682‐http://www.guidetopharmacology.org/GRAC/LigandDisplayForward?ligandId=682 (https://www.genenames.org/data/gene‐symbol‐report/#!/hgnc_id/HGNC:1438, http://www.uniprot.org/uniprot/P10092) ≥ http://www.guidetopharmacology.org/GRAC/LigandDisplayForward?ligandId=684 (https://www.genenames.org/data/gene‐symbol‐report/#!/hgnc_id/HGNC:28898, http://www.uniprot.org/uniprot/Q7Z4H4) ≥ http://www.guidetopharmacology.org/GRAC/LigandDisplayForward?ligandId=685, http://www.uniprot.org/uniprot/P01258) > http://www.guidetopharmacology.org/GRAC/LigandDisplayForward?ligandId=683 (https://www.genenames.org/data/gene‐symbol‐report/#!/hgnc_id/HGNC:259, http://www.uniprot.org/uniprot/P35318)
Endogenous agonists	http://www.guidetopharmacology.org/GRAC/LigandDisplayForward?ligandId=685 (https://www.genenames.org/data/gene‐symbol‐report/#!/hgnc_id/HGNC:1437, http://www.uniprot.org/uniprot/P01258) [http://www.ncbi.nlm.nih.gov/pubmed/7588285?dopt=AbstractPlus, http://www.ncbi.nlm.nih.gov/pubmed/11033437?dopt=AbstractPlus, http://www.ncbi.nlm.nih.gov/pubmed/15692146?dopt=AbstractPlus, http://www.ncbi.nlm.nih.gov/pubmed/12565884?dopt=AbstractPlus, http://www.ncbi.nlm.nih.gov/pubmed/11023820?dopt=AbstractPlus, http://www.ncbi.nlm.nih.gov/pubmed/10342886?dopt=AbstractPlus]	http://www.guidetopharmacology.org/GRAC/LigandDisplayForward?ligandId=681 (https://www.genenames.org/data/gene‐symbol‐report/#!/hgnc_id/HGNC:1437, http://www.uniprot.org/uniprot/P06881) [http://www.ncbi.nlm.nih.gov/pubmed/15692146?dopt=AbstractPlus, http://www.ncbi.nlm.nih.gov/pubmed/14722252?dopt=AbstractPlus, http://www.ncbi.nlm.nih.gov/pubmed/12565884?dopt=AbstractPlus, http://www.ncbi.nlm.nih.gov/pubmed/11023820?dopt=AbstractPlus, http://www.ncbi.nlm.nih.gov/pubmed/26125036?dopt=AbstractPlus], http://www.guidetopharmacology.org/GRAC/LigandDisplayForward?ligandId=687 (https://www.genenames.org/data/gene‐symbol‐report/#!/hgnc_id/HGNC:5329, http://www.uniprot.org/uniprot/P10997) [http://www.ncbi.nlm.nih.gov/pubmed/24169554?dopt=AbstractPlus], http://www.guidetopharmacology.org/GRAC/LigandDisplayForward?ligandId=682 (https://www.genenames.org/data/gene‐symbol‐report/#!/hgnc_id/HGNC:1438, http://www.uniprot.org/uniprot/P10092)	http://www.guidetopharmacology.org/GRAC/LigandDisplayForward?ligandId=687 (https://www.genenames.org/data/gene‐symbol‐report/#!/hgnc_id/HGNC:5329, http://www.uniprot.org/uniprot/P10997) [http://www.ncbi.nlm.nih.gov/pubmed/24169554?dopt=AbstractPlus]	http://www.guidetopharmacology.org/GRAC/LigandDisplayForward?ligandId=687 (https://www.genenames.org/data/gene‐symbol‐report/#!/hgnc_id/HGNC:5329, http://www.uniprot.org/uniprot/P10997) [http://www.ncbi.nlm.nih.gov/pubmed/24169554?dopt=AbstractPlus]
Sub/family‐selective agonists	http://www.guidetopharmacology.org/GRAC/LigandDisplayForward?ligandId=7482 [http://www.ncbi.nlm.nih.gov/pubmed/24169554?dopt=AbstractPlus]	http://www.guidetopharmacology.org/GRAC/LigandDisplayForward?ligandId=7482 [http://www.ncbi.nlm.nih.gov/pubmed/24169554?dopt=AbstractPlus]	–	http://www.guidetopharmacology.org/GRAC/LigandDisplayForward?ligandId=7482 [http://www.ncbi.nlm.nih.gov/pubmed/24169554?dopt=AbstractPlus]
Sub/family‐selective antagonists	http://www.guidetopharmacology.org/GRAC/LigandDisplayForward?ligandId=690) (p*K*_d_ 9) [http://www.ncbi.nlm.nih.gov/pubmed/10856900?dopt=AbstractPlus], http://www.guidetopharmacology.org/GRAC/LigandDisplayForward?ligandId=689 (p*K*_i_ 7.2) [http://www.ncbi.nlm.nih.gov/pubmed/15692146?dopt=AbstractPlus]	http://www.guidetopharmacology.org/GRAC/LigandDisplayForward?ligandId=689 (p*K*_i_ 8) [http://www.ncbi.nlm.nih.gov/pubmed/15692146?dopt=AbstractPlus], http://www.guidetopharmacology.org/GRAC/LigandDisplayForward?ligandId=690) (p*K*_i_ 7.8) [http://www.ncbi.nlm.nih.gov/pubmed/15692146?dopt=AbstractPlus], http://www.guidetopharmacology.org/GRAC/LigandDisplayForward?ligandId=702 (p*K*_d_ 7.2) [http://www.ncbi.nlm.nih.gov/pubmed/26125036?dopt=AbstractPlus]	–	http://www.guidetopharmacology.org/GRAC/LigandDisplayForward?ligandId=690) (p*K*_i_ 7.9) [http://www.ncbi.nlm.nih.gov/pubmed/15692146?dopt=AbstractPlus], http://www.guidetopharmacology.org/GRAC/LigandDisplayForward?ligandId=689 (p*K*_i_ 7.7) [http://www.ncbi.nlm.nih.gov/pubmed/15692146?dopt=AbstractPlus]
Labelled ligands	[http://www.guidetopharmacology.org/GRAC/LigandDisplayForward?ligandId=3776) (Agonist), [http://www.guidetopharmacology.org/GRAC/LigandDisplayForward?ligandId=3777) (Agonist)	[http://www.guidetopharmacology.org/GRAC/LigandDisplayForward?ligandId=3766) (Agonist), [http://www.guidetopharmacology.org/GRAC/LigandDisplayForward?ligandId=3770) (Agonist)	[http://www.guidetopharmacology.org/GRAC/LigandDisplayForward?ligandId=3770) (Agonist)	[http://www.guidetopharmacology.org/GRAC/LigandDisplayForward?ligandId=3770) (Agonist)

**Table:** 

Nomenclature	http://www.guidetopharmacology.org/GRAC/ObjectDisplayForward?objectId=47	http://www.guidetopharmacology.org/GRAC/ObjectDisplayForward?objectId=48	http://www.guidetopharmacology.org/GRAC/ObjectDisplayForward?objectId=49	http://www.guidetopharmacology.org/GRAC/ObjectDisplayForward?objectId=50
HGNC, UniProt	https://www.genenames.org/data/gene‐symbol‐report/#!/hgnc_id/HGNC:16709, http://www.uniprot.org/uniprot/Q16602	–	–	–
Subunits	–	http://www.guidetopharmacology.org/GRAC/ObjectDisplayForward?objectId=47, http://www.guidetopharmacology.org/GRAC/ObjectDisplayForward?objectId=51 (Accessory protein)	http://www.guidetopharmacology.org/GRAC/ObjectDisplayForward?objectId=47, http://www.guidetopharmacology.org/GRAC/ObjectDisplayForward?objectId=52 (Accessory protein)	http://www.guidetopharmacology.org/GRAC/ObjectDisplayForward?objectId=47, http://www.guidetopharmacology.org/GRAC/ObjectDisplayForward?objectId=53 (Accessory protein)
Potency order of endogenous ligands	–	http://www.guidetopharmacology.org/GRAC/LigandDisplayForward?ligandId=681 (https://www.genenames.org/data/gene‐symbol‐report/#!/hgnc_id/HGNC:1437, http://www.uniprot.org/uniprot/P06881), http://www.guidetopharmacology.org/GRAC/LigandDisplayForward?ligandId=682 (https://www.genenames.org/data/gene‐symbol‐report/#!/hgnc_id/HGNC:1438, http://www.uniprot.org/uniprot/P10092) > http://www.guidetopharmacology.org/GRAC/LigandDisplayForward?ligandId=683 (https://www.genenames.org/data/gene‐symbol‐report/#!/hgnc_id/HGNC:259, http://www.uniprot.org/uniprot/P35318) ≥ http://www.guidetopharmacology.org/GRAC/LigandDisplayForward?ligandId=684 (https://www.genenames.org/data/gene‐symbol‐report/#!/hgnc_id/HGNC:28898, http://www.uniprot.org/uniprot/Q7Z4H4) > http://www.guidetopharmacology.org/GRAC/LigandDisplayForward?ligandId=687 (https://www.genenames.org/data/gene‐symbol‐report/#!/hgnc_id/HGNC:5329, http://www.uniprot.org/uniprot/P10997) ≥ http://www.guidetopharmacology.org/GRAC/LigandDisplayForward?ligandId=6973)	http://www.guidetopharmacology.org/GRAC/LigandDisplayForward?ligandId=683 (https://www.genenames.org/data/gene‐symbol‐report/#!/hgnc_id/HGNC:259, http://www.uniprot.org/uniprot/P35318) > http://www.guidetopharmacology.org/GRAC/LigandDisplayForward?ligandId=684 (https://www.genenames.org/data/gene‐symbol‐report/#!/hgnc_id/HGNC:28898, http://www.uniprot.org/uniprot/Q7Z4H4) >http://www.guidetopharmacology.org/GRAC/LigandDisplayForward?ligandId=681 (https://www.genenames.org/data/gene‐symbol‐report/#!/hgnc_id/HGNC:1437, http://www.uniprot.org/uniprot/P06881), http://www.guidetopharmacology.org/GRAC/LigandDisplayForward?ligandId=682 (https://www.genenames.org/data/gene‐symbol‐report/#!/hgnc_id/HGNC:1438, http://www.uniprot.org/uniprot/P10092), http://www.guidetopharmacology.org/GRAC/LigandDisplayForward?ligandId=687 (https://www.genenames.org/data/gene‐symbol‐report/#!/hgnc_id/HGNC:5329, http://www.uniprot.org/uniprot/P10997) > http://www.guidetopharmacology.org/GRAC/LigandDisplayForward?ligandId=6973)	http://www.guidetopharmacology.org/GRAC/LigandDisplayForward?ligandId=683 (https://www.genenames.org/data/gene‐symbol‐report/#!/hgnc_id/HGNC:259, http://www.uniprot.org/uniprot/P35318) ≥ http://www.guidetopharmacology.org/GRAC/LigandDisplayForward?ligandId=684 (https://www.genenames.org/data/gene‐symbol‐report/#!/hgnc_id/HGNC:28898, http://www.uniprot.org/uniprot/Q7Z4H4) ≥http://www.guidetopharmacology.org/GRAC/LigandDisplayForward?ligandId=681 (https://www.genenames.org/data/gene‐symbol‐report/#!/hgnc_id/HGNC:1437, http://www.uniprot.org/uniprot/P06881), http://www.guidetopharmacology.org/GRAC/LigandDisplayForward?ligandId=682 (https://www.genenames.org/data/gene‐symbol‐report/#!/hgnc_id/HGNC:1438, http://www.uniprot.org/uniprot/P10092) > http://www.guidetopharmacology.org/GRAC/LigandDisplayForward?ligandId=687 (https://www.genenames.org/data/gene‐symbol‐report/#!/hgnc_id/HGNC:5329, http://www.uniprot.org/uniprot/P10997) > http://www.guidetopharmacology.org/GRAC/LigandDisplayForward?ligandId=6973)
Endogenous agonists	–	http://www.guidetopharmacology.org/GRAC/LigandDisplayForward?ligandId=682 (https://www.genenames.org/data/gene‐symbol‐report/#!/hgnc_id/HGNC:1438, http://www.uniprot.org/uniprot/P10092) [http://www.ncbi.nlm.nih.gov/pubmed/11693189?dopt=AbstractPlus, http://www.ncbi.nlm.nih.gov/pubmed/9620797?dopt=AbstractPlus], http://www.guidetopharmacology.org/GRAC/LigandDisplayForward?ligandId=681‐http://www.guidetopharmacology.org/GRAC/LigandDisplayForward?ligandId=681 (https://www.genenames.org/data/gene‐symbol‐report/#!/hgnc_id/HGNC:1437, http://www.uniprot.org/uniprot/P06881) http://www.uniprot.org/uniprot/P06881, http://www.ncbi.nlm.nih.gov/pubmed/11693189?dopt=AbstractPlus]	http://www.guidetopharmacology.org/GRAC/LigandDisplayForward?ligandId=683 (https://www.genenames.org/data/gene‐symbol‐report/#!/hgnc_id/HGNC:259, http://www.uniprot.org/uniprot/P35318) [http://www.ncbi.nlm.nih.gov/pubmed/11693189?dopt=AbstractPlus, http://www.ncbi.nlm.nih.gov/pubmed/9620797?dopt=AbstractPlus]	http://www.guidetopharmacology.org/GRAC/LigandDisplayForward?ligandId=683 (https://www.genenames.org/data/gene‐symbol‐report/#!/hgnc_id/HGNC:259, http://www.uniprot.org/uniprot/P35318) [http://www.ncbi.nlm.nih.gov/pubmed/11693189?dopt=AbstractPlus, http://www.ncbi.nlm.nih.gov/pubmed/10347248?dopt=AbstractPlus]
Antagonists	–	http://www.guidetopharmacology.org/GRAC/LigandDisplayForward?ligandId=702 (p*K*_i_ 10.7–11) [http://www.ncbi.nlm.nih.gov/pubmed/10711339?dopt=AbstractPlus, http://www.ncbi.nlm.nih.gov/pubmed/16959943?dopt=AbstractPlus, http://www.ncbi.nlm.nih.gov/pubmed/12970090?dopt=AbstractPlus, http://www.ncbi.nlm.nih.gov/pubmed/24405707?dopt=AbstractPlus, http://www.ncbi.nlm.nih.gov/pubmed/11847213?dopt=AbstractPlus], http://www.guidetopharmacology.org/GRAC/LigandDisplayForward?ligandId=703 (p*K*_i_ 9.1) [http://www.ncbi.nlm.nih.gov/pubmed/18039958?dopt=AbstractPlus]	–	–
Selective antagonists	–	–	http://www.guidetopharmacology.org/GRAC/LigandDisplayForward?ligandId=706) (p*K*_i_ 7–7.8) [http://www.ncbi.nlm.nih.gov/pubmed/12970090?dopt=AbstractPlus]	http://www.guidetopharmacology.org/GRAC/LigandDisplayForward?ligandId=706)
Labelled ligands	–	[http://www.guidetopharmacology.org/GRAC/LigandDisplayForward?ligandId=3766) (Agonist), [http://www.guidetopharmacology.org/GRAC/LigandDisplayForward?ligandId=6567) (Agonist)	[http://www.guidetopharmacology.org/GRAC/LigandDisplayForward?ligandId=3768) (Agonist)	[http://www.guidetopharmacology.org/GRAC/LigandDisplayForward?ligandId=3768) (Agonist)

### Comments

It is important to note that a complication with the interpretation of pharmacological studies with AMY receptors in transfected cells is that most of this work has likely used a mixed population of receptors, encompassing RAMP‐coupled CTR as well as CTR alone. This means that although in binding assays human http://www.guidetopharmacology.org/GRAC/LigandDisplayForward?ligandId=685 (https://www.genenames.org/data/gene‐symbol‐report/#!/hgnc_id/HGNC:1437, http://www.uniprot.org/uniprot/P01258) has low affinity for 125I‐AMY binding sites, cells transfected with CTR and RAMPs can display potent CT functional responses. Transfection of human CTR with any RAMP can generate receptors with a high affinity for both salmon CT and AMY and varying affinity for different antagonists [http://www.ncbi.nlm.nih.gov/pubmed/10385705?dopt=AbstractPlus, http://www.ncbi.nlm.nih.gov/pubmed/15692146?dopt=AbstractPlus, http://www.ncbi.nlm.nih.gov/pubmed/16959943?dopt=AbstractPlus]. The major human CTR splice variant (hCT_(_a), which does not contain an insert) with RAMP1 (*i.e.* the AMY_1(_a) receptor) has a high affinity for CGRP [http://www.ncbi.nlm.nih.gov/pubmed/26125036?dopt=AbstractPlus], unlike hCT_(_a)‐RAMP3 (*i.e*. AMY_3(_a) receptor) [http://www.ncbi.nlm.nih.gov/pubmed/10385705?dopt=AbstractPlus, http://www.ncbi.nlm.nih.gov/pubmed/15692146?dopt=AbstractPlus]. However, the AMY receptor phenotype is RAMP‐type, splice variant and cell‐line‐dependent [http://www.ncbi.nlm.nih.gov/pubmed/18599553?dopt=AbstractPlus, http://www.ncbi.nlm.nih.gov/pubmed/22946511?dopt=AbstractPlus, http://www.ncbi.nlm.nih.gov/pubmed/10871296?dopt=AbstractPlus]. Emerging data suggests that AMY_1_ could be a second CGRP receptor [http://www.ncbi.nlm.nih.gov/pubmed/29797087?dopt=AbstractPlus].

The ligands described have limited selectivity. Adrenomedullin has appreciable affinity for CGRP receptors. CGRP can show significant cross‐reactivity at AMY receptors and AM_2_ receptors. Adrenomedullin 2/intermedin also has high affinity for the AM_2_ receptor [http://www.ncbi.nlm.nih.gov/pubmed/21658025?dopt=AbstractPlus]. CGRP‐(8‐37) acts as an antagonist of CGRP (*p*Ki 8) and inhibits some AM and AMY responses (*p*Ki 6‐7). It is weak at CT receptors. HumanAM‐(22‐52)has some selectivity towardsAM receptors, but with modest potency (*p*Ki 7), limiting its use [http://www.ncbi.nlm.nih.gov/pubmed/12970090?dopt=AbstractPlus]. http://www.guidetopharmacology.org/GRAC/LigandDisplayForward?ligandId=702 shows the greatest selectivity between receptors but still has significant affinity for AMY_1_ receptors [http://www.ncbi.nlm.nih.gov/pubmed/26125036?dopt=AbstractPlus].

Gs is a prominent route for effector coupling for CLR and CTR but other pathways (*e.g.* Ca^2+^, ERK, Akt), and G proteins can be activated [http://www.ncbi.nlm.nih.gov/pubmed/20633935?dopt=AbstractPlus]. There is evidence that CGRP‐RCP (a 148 amino‐acid hydrophilic protein, https://www.genenames.org/data/gene‐symbol‐report/#!/hgnc_id/HGNC:746 (http://www.uniprot.org/uniprot/P04424) is important for the coupling of CLR to adenylyl cyclase [http://www.ncbi.nlm.nih.gov/pubmed/10903324?dopt=AbstractPlus].

[125I]‐Salmon CT is the most common radioligand for CT receptors but it has high affinity for AMY receptors and is also poorly reversible.

### Further reading on Calcitonin receptors

Hay DL *et al*. (2018) Update on the pharmacology of calcitonin/CGRP family of peptides: IUPHAR Review 25. *Br. J. Pharmacol.* **175**: 3‐17 [https://www.ncbi.nlm.nih.gov/pubmed/29059473?dopt=AbstractPlus]

Russell FA *et al*. (2014) Calcitonin gene‐related peptide: physiology and pathophysiology. *Physiol. Rev.* **94**: 1099‐142 [https://www.ncbi.nlm.nih.gov/pubmed/25287861?dopt=AbstractPlus]

Hay DL *et al*. (2016) Receptor Activity‐Modifying Proteins (RAMPs): New Insights and Roles. *Annu. Rev. Pharmacol. Toxicol.* **56**: 469‐87 [https://www.ncbi.nlm.nih.gov/pubmed/26514202?dopt=AbstractPlus]

Russo AF. (2015) Calcitonin gene‐related peptide (CGRP): a new target for migraine. *Annu. Rev. Pharmacol. Toxicol.* **55**: 533‐52 [https://www.ncbi.nlm.nih.gov/pubmed/25340934?dopt=AbstractPlus]

Kato J *et al*. (2015) Bench‐to‐bedside pharmacology of adrenomedullin. *Eur. J. Pharmacol.* **764**: 140‐8 [https://www.ncbi.nlm.nih.gov/pubmed/26144371?dopt=AbstractPlus]

## http://www.guidetopharmacology.org/GRAC/FamilyDisplayForward?familyId=12

### Overview

The calcium‐sensing receptor (CaS, **provisional nomenclature as recommended by NC‐IUPHAR** [http://www.ncbi.nlm.nih.gov/pubmed/15914470?dopt=AbstractPlus]) responds to multiple endogenous ligands, including extracellular calcium and other divalent/trivalent cations, polyamines and polycationic peptides, L‐amino acids (particularly L‐Trp and L‐Phe), glutathione and various peptide analogues, ionic strength and extracellular pH (reviewed in [http://www.ncbi.nlm.nih.gov/pubmed/24111791?dopt=AbstractPlus]). While divalent/trivalent cations, polyamines and polycations are CaS receptor agonists [http://www.ncbi.nlm.nih.gov/pubmed/8255296?dopt=AbstractPlus, http://www.ncbi.nlm.nih.gov/pubmed/9357776?dopt=AbstractPlus], L‐amino acids, glutamyl peptides, ionic strength and pH are allosteric modulators of agonist function [http://www.ncbi.nlm.nih.gov/pubmed/10781086?dopt=AbstractPlus, http://www.ncbi.nlm.nih.gov/pubmed/10781086?dopt=AbstractPlus, http://www.ncbi.nlm.nih.gov/pubmed/7493018?dopt=AbstractPlus, http://www.ncbi.nlm.nih.gov/pubmed/15201280?dopt=AbstractPlus, http://www.ncbi.nlm.nih.gov/pubmed/9677383?dopt=AbstractPlus]. Indeed, L‐amino acids have been identified as "co‐agonists", with both concomitant calcium and L‐amino acid binding required for full receptor activation [http://www.ncbi.nlm.nih.gov/pubmed/27434672?dopt=AbstractPlus, http://www.ncbi.nlm.nih.gov/pubmed/27746744?dopt=AbstractPlus]. The sensitivity of the CaS receptor to primary agonists is increased by elevated extracellular pH [http://www.ncbi.nlm.nih.gov/pubmed/25556167?dopt=AbstractPlus] or decreased extracellular ionic strength [http://www.ncbi.nlm.nih.gov/pubmed/9677383?dopt=AbstractPlus]. This receptor bears no sequence or structural relation to the plant calcium receptor, also called CaS.

**Table:** 

Nomenclature	http://www.guidetopharmacology.org/GRAC/ObjectDisplayForward?objectId=54
HGNC, UniProt	https://www.genenames.org/data/gene‐symbol‐report/#!/hgnc_id/HGNC:1514, http://www.uniprot.org/uniprot/P41180
Amino‐acid rank order of potency	http://www.guidetopharmacology.org/GRAC/LigandDisplayForward?ligandId=3313, http://www.guidetopharmacology.org/GRAC/LigandDisplayForward?ligandId=717, http://www.guidetopharmacology.org/GRAC/LigandDisplayForward?ligandId=3310 > http://www.guidetopharmacology.org/GRAC/LigandDisplayForward?ligandId=720 > http://www.guidetopharmacology.org/GRAC/LigandDisplayForward?ligandId=726, http://www.guidetopharmacology.org/GRAC/LigandDisplayForward?ligandId=3314, http://www.guidetopharmacology.org/GRAC/LigandDisplayForward?ligandId=1369 > http://www.guidetopharmacology.org/GRAC/LigandDisplayForward?ligandId=3309 (not L‐lysine, L‐arginine, L‐leucine and L‐isoleucine) [http://www.ncbi.nlm.nih.gov/pubmed/10781086?dopt=AbstractPlus]
Cation rank order of potency	http://www.guidetopharmacology.org/GRAC/LigandDisplayForward?ligandId=2426 > http://www.guidetopharmacology.org/GRAC/LigandDisplayForward?ligandId=707 > http://www.guidetopharmacology.org/GRAC/LigandDisplayForward?ligandId=708 [http://www.ncbi.nlm.nih.gov/pubmed/8255296?dopt=AbstractPlus]
Glutamyl peptide rank order of potency	S‐methylglutathione ≈ γGlu‐Val‐Gly > glutathione >γGlu‐Cys [http://www.ncbi.nlm.nih.gov/pubmed/21187282?dopt=AbstractPlus, http://www.ncbi.nlm.nih.gov/pubmed/19892707?dopt=AbstractPlus, http://www.ncbi.nlm.nih.gov/pubmed/16455645?dopt=AbstractPlus]
Polyamine rank order of potency	http://www.guidetopharmacology.org/GRAC/LigandDisplayForward?ligandId=710 > http://www.guidetopharmacology.org/GRAC/LigandDisplayForward?ligandId=2390 > http://www.guidetopharmacology.org/GRAC/LigandDisplayForward?ligandId=2388 [http://www.ncbi.nlm.nih.gov/pubmed/9357776?dopt=AbstractPlus]
Allosteric modulators	http://www.guidetopharmacology.org/GRAC/LigandDisplayForward?ligandId=9475 (Negative) (pIC_50_ 8.9) [http://www.ncbi.nlm.nih.gov/pubmed/20158186?dopt=AbstractPlus], http://www.guidetopharmacology.org/GRAC/LigandDisplayForward?ligandId=9474 (Negative) (pIC_50_ 7.9) [http://www.ncbi.nlm.nih.gov/pubmed/24900301?dopt=AbstractPlus], http://www.guidetopharmacology.org/GRAC/LigandDisplayForward?ligandId=9476 (Negative) (pIC_50_ 7.1) [http://www.ncbi.nlm.nih.gov/pubmed/19786130?dopt=AbstractPlus], http://www.guidetopharmacology.org/GRAC/LigandDisplayForward?ligandId=9473 (Negative) (pIC_50_ 6.5–6.8) [http://www.ncbi.nlm.nih.gov/pubmed/19442519?dopt=AbstractPlus], http://www.guidetopharmacology.org/GRAC/LigandDisplayForward?ligandId=716 (Negative) (p*K*_B_ 6.2–6.7) [http://www.ncbi.nlm.nih.gov/pubmed/22210744?dopt=AbstractPlus, http://www.ncbi.nlm.nih.gov/pubmed/27002221?dopt=AbstractPlus, http://www.ncbi.nlm.nih.gov/pubmed/23372019?dopt=AbstractPlus], http://www.guidetopharmacology.org/GRAC/LigandDisplayForward?ligandId=3308 (Positive) (p*K*_B_ 5.9–6.6) [http://www.ncbi.nlm.nih.gov/pubmed/25220431?dopt=AbstractPlus, http://www.ncbi.nlm.nih.gov/pubmed/22210744?dopt=AbstractPlus, http://www.ncbi.nlm.nih.gov/pubmed/27002221?dopt=AbstractPlus], http://www.guidetopharmacology.org/GRAC/LigandDisplayForward?ligandId=718 (Positive) (p*K*_B_ 6.2–6.6) [http://www.ncbi.nlm.nih.gov/pubmed/25220431?dopt=AbstractPlus, http://www.ncbi.nlm.nih.gov/pubmed/25220431?dopt=AbstractPlus], http://www.guidetopharmacology.org/GRAC/LigandDisplayForward?ligandId=3947 (Positive) (p*K*_B_ 6.3–6.4) [http://www.ncbi.nlm.nih.gov/pubmed/25220431?dopt=AbstractPlus, http://www.ncbi.nlm.nih.gov/pubmed/27002221?dopt=AbstractPlus], http://www.guidetopharmacology.org/GRAC/LigandDisplayForward?ligandId=714 (Negative) (pIC_50_ 6.4) [http://www.ncbi.nlm.nih.gov/pubmed/14506236?dopt=AbstractPlus], http://www.guidetopharmacology.org/GRAC/LigandDisplayForward?ligandId=719 (Positive) (p*K*_B_ 6.3) [http://www.ncbi.nlm.nih.gov/pubmed/25220431?dopt=AbstractPlus]

### Comments

The CaS receptor has a number of physiological functions, but it is best known for its central role in parathyroid and renal regulation of extracellular calcium homeostasis [http://www.ncbi.nlm.nih.gov/pubmed/27647839?dopt=AbstractPlus]. This is seen most clearly in patients with loss‐of‐function CaS receptor mutations who develop familial hypocalciuric hypercalcaemia (heterozygous mutations) or neonatal severe hyperparathyroidism (heterozygous, compound heterozygous or homozygous mutations) [http://www.ncbi.nlm.nih.gov/pubmed/27647839?dopt=AbstractPlus] and in *Casr* null mice [http://www.ncbi.nlm.nih.gov/pubmed/18765830?dopt=AbstractPlus, http://www.ncbi.nlm.nih.gov/pubmed/7493018?dopt=AbstractPlus], which exhibit similar increases in PTH secretion and blood calcium levels. Gain‐of‐function CaS mutations are associated with autosomal dominant hypocalcaemia and Bartter syndrome type V [http://www.ncbi.nlm.nih.gov/pubmed/27647839?dopt=AbstractPlus].

The CaS receptor primarily couples to G_q/11_, G_12_/13 and G_i/o_ [http://www.ncbi.nlm.nih.gov/pubmed/22210744?dopt=AbstractPlus, http://www.ncbi.nlm.nih.gov/pubmed/16247029?dopt=AbstractPlus, http://www.ncbi.nlm.nih.gov/pubmed/12954603?dopt=AbstractPlus, http://www.ncbi.nlm.nih.gov/pubmed/22192592?dopt=AbstractPlus], but in some cell types can couple to Gs [http://www.ncbi.nlm.nih.gov/pubmed/20032198?dopt=AbstractPlus]. However, the CaS receptor can form heteromers with Class C GABAB [http://www.ncbi.nlm.nih.gov/pubmed/17591780?dopt=AbstractPlus, http://www.ncbi.nlm.nih.gov/pubmed/17615148?dopt=AbstractPlus] and mGlu1/5 receptors [http://www.ncbi.nlm.nih.gov/pubmed/11489900?dopt=AbstractPlus], which may introduce further complexity in its signalling capabilities.

Multiple other small molecule chemotypes are positive and negative allosteric modulators of the CaS receptor [http://www.ncbi.nlm.nih.gov/pubmed/21406038?dopt=AbstractPlus, http://www.ncbi.nlm.nih.gov/pubmed/24050279?dopt=AbstractPlus]. Further, http://www.guidetopharmacology.org/GRAC/LigandDisplayForward?ligandId=8375 is a novel peptide positive allosteric modulator of the receptor [http://www.ncbi.nlm.nih.gov/pubmed/23674604?dopt=AbstractPlus]. Agonists and positive allosteric modulators of the CaS receptor are termed Type I and II calcimimetics, respectively, and can suppress parathyroid hormone (http://www.guidetopharmacology.org/GRAC/LigandDisplayForward?ligandId=1785 (https://www.genenames.org/data/gene‐symbol‐report/#!/hgnc_id/HGNC:9606, http://www.uniprot.org/uniprot/P01270)) secretion [http://www.ncbi.nlm.nih.gov/pubmed/9520489?dopt=AbstractPlus]. Negative allosteric modulators are called calcilytics and can act to increase http://www.guidetopharmacology.org/GRAC/LigandDisplayForward?ligandId=1785, https://www.genenames.org/data/gene‐symbol‐report/#!/hgnc_id/HGNC:9606) secretion [http://www.ncbi.nlm.nih.gov/pubmed/11561095?dopt=AbstractPlus].

Where functional pKB values are provided for allosteric modulators, this refers to ligand affinity determined in an assay that measures a functional readout of receptor activity (*i.e.* a receptor signalling assay), as opposed to affinity determined in a radioligand binding assay. The functional pKB may differ depending on the signalling pathway studied. Consult the http://www.guidetopharmacology.org/GRAC/ObjectDisplayForward?objectId=54&familyId=12&familyType=GPCR#Allosterics for the assay description, as well as other functional readouts.

### Further reading on Calcium‐sensing receptor

Brown EM. (2013) Role of the calcium‐sensing receptor in extracellular calcium homeostasis. *Best Pract. Res. Clin. Endocrinol. Metab.* **27**: 333‐43 [https://www.ncbi.nlm.nih.gov/pubmed/23856263?dopt=AbstractPlus]

Hannan FM *et al*. (2018) The calcium‐sensing receptor in physiology and in calcitropic and noncal citropic diseases. *Nat Rev Endocrinol* **15**: 33‐51 [https://www.ncbi.nlm.nih.gov/pubmed/30443043?dopt=AbstractPlus]

Conigrave AD *et al*. (2013) Calcium‐sensing receptor (CaSR): pharmacological properties and signaling pathways. *Best Pract. Res. Clin. Endocrinol. Metab.* **27**: 315‐31 [https://www.ncbi.nlm.nih.gov/pubmed/23856262?dopt=AbstractPlus]

Nemeth EF *et al*. (2018) Discovery and Development of Calcimimetic and Calcilytic Compounds. *Prog Med Chem* **57**: 1‐86 [https://www.ncbi.nlm.nih.gov/pubmed/29680147?dopt=AbstractPlus]

## http://www.guidetopharmacology.org/GRAC/FamilyDisplayForward?familyId=13

### Overview

Cannabinoid receptors (**nomenclature as agreed by the NC‐IUPHAR Subcommittee on Cannabinoid Receptors** [http://www.ncbi.nlm.nih.gov/pubmed/21079038?dopt=AbstractPlus]) are activated by endogenous ligands that include N‐arachidonoylethanolamine (http://www.guidetopharmacology.org/GRAC/LigandDisplayForward?ligandId=2364), http://www.guidetopharmacology.org/GRAC/LigandDisplayForward?ligandId=5444‐http://www.guidetopharmacology.org/GRAC/LigandDisplayForward?ligandId=5444, http://www.guidetopharmacology.org/GRAC/LigandDisplayForward?ligandId=5445 and http://www.guidetopharmacology.org/GRAC/LigandDisplayForward?ligandId=729. Potency determinations of endogenous agonists at these receptors are complicated by the possibility of differential susceptibility of endogenous ligands to enzymatic conversion [http://www.ncbi.nlm.nih.gov/pubmed/17876303?dopt=AbstractPlus].

There are currently three licenced cannabinoid medicines each of which contains a compound that can activate CB_1_ and CB_2_ receptors [http://www.ncbi.nlm.nih.gov/pubmed/23108552?dopt=AbstractPlus]. Two of these medicines were developed to suppress nausea and vomiting produced by chemotherapy. These are http://www.guidetopharmacology.org/GRAC/LigandDisplayForward?ligandId=9071 (Cesamet®), a synthetic CB_1_/CB_2_ receptor agonist, and synthetic http://www.guidetopharmacology.org/GRAC/LigandDisplayForward?ligandId=2424 (Marinol®; dronabinol), which can also be used as an appetite stimulant. The third medicine, Sativex®, contains mainly http://www.guidetopharmacology.org/GRAC/LigandDisplayForward?ligandId=2424 and http://www.guidetopharmacology.org/GRAC/LigandDisplayForward?ligandId=4150, both extracted from cannabis, and is used to treat multiple sclerosis and cancer pain.

**Table:** 

Nomenclature	http://www.guidetopharmacology.org/GRAC/ObjectDisplayForward?objectId=56 http://www.guidetopharmacology.org/GRAC/ObjectDisplayForward?objectId=56	http://www.guidetopharmacology.org/GRAC/ObjectDisplayForward?objectId=57 http://www.guidetopharmacology.org/GRAC/ObjectDisplayForward?objectId=57
HGNC, UniProt	https://www.genenames.org/data/gene‐symbol‐report/#!/hgnc_id/HGNC:2159, http://www.uniprot.org/uniprot/P21554	https://www.genenames.org/data/gene‐symbol‐report/#!/hgnc_id/HGNC:2160, http://www.uniprot.org/uniprot/P34972
Agonists	http://www.guidetopharmacology.org/GRAC/LigandDisplayForward?ligandId=731 [http://www.ncbi.nlm.nih.gov/pubmed/7565624?dopt=AbstractPlus, http://www.ncbi.nlm.nih.gov/pubmed/8819477?dopt=AbstractPlus], http://www.guidetopharmacology.org/GRAC/LigandDisplayForward?ligandId=730, http://www.ncbi.nlm.nih.gov/pubmed/10188977?dopt=AbstractPlus, http://www.ncbi.nlm.nih.gov/pubmed/10188977?dopt=AbstractPlus], http://www.guidetopharmacology.org/GRAC/LigandDisplayForward?ligandId=733, http://www.ncbi.nlm.nih.gov/pubmed/8679694?dopt=AbstractPlus, http://www.ncbi.nlm.nih.gov/pubmed/8679694?dopt=AbstractPlus], http://www.guidetopharmacology.org/GRAC/LigandDisplayForward?ligandId=2424‐http://www.guidetopharmacology.org/GRAC/LigandDisplayForward?ligandId=2424 (Partial agonist) [http://www.ncbi.nlm.nih.gov/pubmed/7565624?dopt=AbstractPlus], http://www.guidetopharmacology.org/GRAC/LigandDisplayForward?ligandId=740 (Partial agonist) [http://www.ncbi.nlm.nih.gov/pubmed/7565624?dopt=AbstractPlus, http://www.ncbi.nlm.nih.gov/pubmed/7565624?dopt=AbstractPlus]	http://www.guidetopharmacology.org/GRAC/LigandDisplayForward?ligandId=731 [http://www.ncbi.nlm.nih.gov/pubmed/7565624?dopt=AbstractPlus, http://www.ncbi.nlm.nih.gov/pubmed/9379442?dopt=AbstractPlus, http://www.ncbi.nlm.nih.gov/pubmed/8819477?dopt=AbstractPlus], http://www.guidetopharmacology.org/GRAC/LigandDisplayForward?ligandId=733, http://www.ncbi.nlm.nih.gov/pubmed/8679694?dopt=AbstractPlus, http://www.ncbi.nlm.nih.gov/pubmed/8679694?dopt=AbstractPlus], http://www.guidetopharmacology.org/GRAC/LigandDisplayForward?ligandId=730, http://www.ncbi.nlm.nih.gov/pubmed/10188977?dopt=AbstractPlus, http://www.ncbi.nlm.nih.gov/pubmed/10188977?dopt=AbstractPlus], http://www.guidetopharmacology.org/GRAC/LigandDisplayForward?ligandId=2424‐http://www.guidetopharmacology.org/GRAC/LigandDisplayForward?ligandId=2424 (Partial agonist) [http://www.ncbi.nlm.nih.gov/pubmed/8626625?dopt=AbstractPlus, http://www.ncbi.nlm.nih.gov/pubmed/8626625?dopt=AbstractPlus, http://www.ncbi.nlm.nih.gov/pubmed/9379442?dopt=AbstractPlus, http://www.ncbi.nlm.nih.gov/pubmed/8819477?dopt=AbstractPlus]
Selective agonists	http://www.guidetopharmacology.org/GRAC/LigandDisplayForward?ligandId=738 [http://www.ncbi.nlm.nih.gov/pubmed/10336536?dopt=AbstractPlus] – Rat, http://www.guidetopharmacology.org/GRAC/LigandDisplayForward?ligandId=739] – Rat, http://www.guidetopharmacology.org/GRAC/LigandDisplayForward?ligandId=732 [http://www.ncbi.nlm.nih.gov/pubmed/11181068?dopt=AbstractPlus] – Rat, http://www.guidetopharmacology.org/GRAC/LigandDisplayForward?ligandId=2506 [http://www.ncbi.nlm.nih.gov/pubmed/8893848?dopt=AbstractPlus] – Rat	http://www.guidetopharmacology.org/GRAC/LigandDisplayForward?ligandId=747 [http://www.ncbi.nlm.nih.gov/pubmed/10658595?dopt=AbstractPlus, http://www.ncbi.nlm.nih.gov/pubmed/11060760?dopt=AbstractPlus], http://www.guidetopharmacology.org/GRAC/LigandDisplayForward?ligandId=748 [0663, http://www.ncbi.nlm.nih.gov/pubmed/10188977?dopt=AbstractPlus], http://www.guidetopharmacology.org/GRAC/LigandDisplayForward?ligandId=3316 [http://www.ncbi.nlm.nih.gov/pubmed/16894349?dopt=AbstractPlus], http://www.guidetopharmacology.org/GRAC/LigandDisplayForward?ligandId=749 [0663, http://www.ncbi.nlm.nih.gov/pubmed/10188977?dopt=AbstractPlus], http://www.guidetopharmacology.org/GRAC/LigandDisplayForward?ligandId=746 [http://www.ncbi.nlm.nih.gov/pubmed/10588688?dopt=AbstractPlus]
Selective antagonists	http://www.guidetopharmacology.org/GRAC/LigandDisplayForward?ligandId=743 (p*K*_i_ 7.9–8.7) [http://www.ncbi.nlm.nih.gov/pubmed/9435190?dopt=AbstractPlus, http://www.ncbi.nlm.nih.gov/pubmed/7565624?dopt=AbstractPlus, http://www.ncbi.nlm.nih.gov/pubmed/8070571?dopt=AbstractPlus, http://www.ncbi.nlm.nih.gov/pubmed/12663689?dopt=AbstractPlus, http://www.ncbi.nlm.nih.gov/pubmed/8819477?dopt=AbstractPlus], http://www.guidetopharmacology.org/GRAC/LigandDisplayForward?ligandId=10029 (p*K*_i_ 8.5) [http://www.ncbi.nlm.nih.gov/pubmed/25535367?dopt=AbstractPlus], http://www.guidetopharmacology.org/GRAC/LigandDisplayForward?ligandId=3317 (p*K*_i_ 8.1) [http://www.ncbi.nlm.nih.gov/pubmed/10052983?dopt=AbstractPlus] – Rat, http://www.guidetopharmacology.org/GRAC/LigandDisplayForward?ligandId=741 (p*K*_i_ 7.9) [http://www.ncbi.nlm.nih.gov/pubmed/11741201?dopt=AbstractPlus] – Rat, http://www.guidetopharmacology.org/GRAC/LigandDisplayForward?ligandId=742 (p*K*_i_ 6.9) [http://www.ncbi.nlm.nih.gov/pubmed/9435190?dopt=AbstractPlus]	http://www.guidetopharmacology.org/GRAC/LigandDisplayForward?ligandId=751 (p*K*_i_ 8.3–9.2) [http://www.ncbi.nlm.nih.gov/pubmed/9454810?dopt=AbstractPlus, http://www.ncbi.nlm.nih.gov/pubmed/10188977?dopt=AbstractPlus], http://www.guidetopharmacology.org/GRAC/LigandDisplayForward?ligandId=750 (p*K*_i_ 7.5) [http://www.ncbi.nlm.nih.gov/pubmed/10188977?dopt=AbstractPlus]
Allosteric modulators	http://www.guidetopharmacology.org/GRAC/LigandDisplayForward?ligandId=9237 (Negative) (pEC_50_ 7.7) [http://www.ncbi.nlm.nih.gov/pubmed/26529344?dopt=AbstractPlus], http://www.guidetopharmacology.org/GRAC/LigandDisplayForward?ligandId=9239 (Positive) (pEC_50_ 6.3) [http://www.ncbi.nlm.nih.gov/pubmed/26052038?dopt=AbstractPlus] – Mouse, http://www.guidetopharmacology.org/GRAC/LigandDisplayForward?ligandId=10272 (Positive) [http://www.ncbi.nlm.nih.gov/pubmed/28103441?dopt=AbstractPlus], http://www.guidetopharmacology.org/GRAC/LigandDisplayForward?ligandId=4150 (Negative) [http://www.ncbi.nlm.nih.gov/pubmed/26218440?dopt=AbstractPlus]	http://www.guidetopharmacology.org/GRAC/LigandDisplayForward?ligandId=9188 (Positive) (p*K*i ∼7.3) [http://www.ncbi.nlm.nih.gov/pubmed/28842619?dopt=AbstractPlus], http://www.guidetopharmacology.org/GRAC/LigandDisplayForward?ligandId=10273 (Positive) [http://www.ncbi.nlm.nih.gov/pubmed/29990428?dopt=AbstractPlus]
Labelled ligands	[http://www.guidetopharmacology.org/GRAC/LigandDisplayForward?ligandId=745 (Antagonist) (p*K*_d_ 8.9–10) [http://www.ncbi.nlm.nih.gov/pubmed/9316881?dopt=AbstractPlus, http://www.ncbi.nlm.nih.gov/pubmed/8981483?dopt=AbstractPlus, http://www.ncbi.nlm.nih.gov/pubmed/8978752?dopt=AbstractPlus, http://www.ncbi.nlm.nih.gov/pubmed/8733746?dopt=AbstractPlus, http://www.ncbi.nlm.nih.gov/pubmed/8614277?dopt=AbstractPlus, http://www.ncbi.nlm.nih.gov/pubmed/8987831?dopt=AbstractPlus, http://www.ncbi.nlm.nih.gov/pubmed/9536023?dopt=AbstractPlus] – Rat	–

### Comments

Both CB_1_ and CB_2_ receptors may be labelled with [http://www.guidetopharmacology.org/GRAC/LigandDisplayForward?ligandId=734 (0.5 nM;[http://www.ncbi.nlm.nih.gov/pubmed/8819477?dopt=AbstractPlus]) and http://www.guidetopharmacology.org/GRAC/LigandDisplayForward?ligandId=736 (2‐2.4 nM; [http://www.ncbi.nlm.nih.gov/pubmed/7651369?dopt=AbstractPlus, http://www.ncbi.nlm.nih.gov/pubmed/8622639?dopt=AbstractPlus]). http://www.guidetopharmacology.org/GRAC/LigandDisplayForward?ligandId=2364 is also an agonist at vanilloid receptors (http://www.guidetopharmacology.org/GRAC/FamilyDisplayForward?familyId=78%23show_object_507)and http://www.guidetopharmacology.org/GRAC/FamilyDisplayForward?familyId=86 [http://www.ncbi.nlm.nih.gov/pubmed/17704824?dopt=AbstractPlus, http://www.ncbi.nlm.nih.gov/pubmed/10440374?dopt=AbstractPlus]. There is evidence for an allosteric site on the CB_1_ receptor [http://www.ncbi.nlm.nih.gov/pubmed/16113085?dopt=AbstractPlus]. All of the compounds listed as antagonists behave as inverse agonists in some bioassay systems [http://www.ncbi.nlm.nih.gov/pubmed/21079038?dopt=AbstractPlus]. For some cannabinoid receptor ligands, additional pharmacological targets that include GPR55 and GPR119 have been identified [http://www.ncbi.nlm.nih.gov/pubmed/21079038?dopt=AbstractPlus]. Moreover, http://www.guidetopharmacology.org/GRAC/FamilyDisplayForward?familyId=114 although showing little structural similarity to CB_1_ and CB_2_ receptors, respond to endogenous agents that are structurally similar to the endogenous cannabinoid ligands [http://www.ncbi.nlm.nih.gov/pubmed/21079038?dopt=AbstractPlus].

### Further reading on Cannabinoid receptors

Howlett AC *et al*. (2002) International Union of Pharmacology. XXVII. Classification of cannabinoid receptors. *Pharmacol. Rev.* **54**: 161‐202 [https://www.ncbi.nlm.nih.gov/pubmed/12037135?dopt=AbstractPlus]

Pertwee RG *et al*. (2010) International Union of Basic and Clinical Pharmacology. LXXIX. Cannabinoid receptors and their ligands: beyond CB_1 and CB_2. *Pharmacol. Rev.* **62**: 588‐631 [https://www.ncbi.nlm.nih.gov/pubmed/21079038?dopt=AbstractPlus]

Pertwee RG. (2010) Receptors and channels targeted by synthetic cannabinoid receptor agonists and antagonists. *Curr. Med. Chem.* **17**: 1360‐81 [https://www.ncbi.nlm.nih.gov/pubmed/20166927?dopt=AbstractPlus]

## http://www.guidetopharmacology.org/GRAC/FamilyDisplayForward?familyId=338

### Overview

Nomenclature for the chemerin receptors is presented as **recommended by NC‐IUPHAR** [http://www.ncbi.nlm.nih.gov/pubmed/23686350?dopt=AbstractPlus, http://www.ncbi.nlm.nih.gov/pubmed/29279348?dopt=AbstractPlus]). The chemoattractant protein and adipokine, http://www.guidetopharmacology.org/GRAC/LigandDisplayForward?ligandId=2945 (https://www.genenames.org/data/gene‐symbol‐report/#!/hgnc_id/HGNC:9868, http://www.uniprot.org/uniprot/Q99969), has been shown to be the endogenous ligand for both chemerin family receptors. Chemerin_1_ was the founding family member, and when *GPR1* was de‐orphanised it was re‐named Chermerin_2_ [http://www.ncbi.nlm.nih.gov/pubmed/29279348?dopt=AbstractPlus]. Chemerin_1_ is also activated by the lipid‐derived, antiinflammatory ligand http://www.guidetopharmacology.org/GRAC/LigandDisplayForward?ligandId=3333 (RvE1), which is formed *via* the sequential metabolism of http://www.guidetopharmacology.org/GRAC/LigandDisplayForward?ligandId=3362 by aspirin‐modified cyclooxygenase and lipoxygenase [http://www.ncbi.nlm.nih.gov/pubmed/15753205?dopt=AbstractPlus, http://www.ncbi.nlm.nih.gov/pubmed/17339491?dopt=AbstractPlus]. In addition, two GPCRs for http://www.guidetopharmacology.org/GRAC/LigandDisplayForward?ligandId=3934 (RvD1) have been identified: FPR2/ALX, the lipoxin A4 receptor, and GPR32, an orphan receptor [http://www.ncbi.nlm.nih.gov/pubmed/20080636?dopt=AbstractPlus].

**Table:** 

Nomenclature	http://www.guidetopharmacology.org/GRAC/ObjectDisplayForward?objectId=79	http://www.guidetopharmacology.org/GRAC/ObjectDisplayForward?objectId=82
Common abbreviation	Chemerin_1_	Chemerin_2_
HGNC, UniProt	https://www.genenames.org/data/gene‐symbol‐report/#!/hgnc_id/HGNC:2121, http://www.uniprot.org/uniprot/Q99788	https://www.genenames.org/data/gene‐symbol‐report/#!/hgnc_id/HGNC:4463, http://www.uniprot.org/uniprot/P46091
Potency order of endogenous ligands	http://www.guidetopharmacology.org/GRAC/LigandDisplayForward?ligandId=3333 > http://www.guidetopharmacology.org/GRAC/LigandDisplayForward?ligandId=3422 > http://www.guidetopharmacology.org/GRAC/LigandDisplayForward?ligandId=3400 > http://www.guidetopharmacology.org/GRAC/LigandDisplayForward?ligandId=3362 [http://www.ncbi.nlm.nih.gov/pubmed/15753205?dopt=AbstractPlus]	–
Endogenous agonists	–	http://www.guidetopharmacology.org/GRAC/LigandDisplayForward?ligandId=2945 (https://www.genenames.org/data/gene‐symbol‐report/#!/hgnc_id/HGNC:9868, http://www.uniprot.org/uniprot/Q99969) [http://www.ncbi.nlm.nih.gov/pubmed/18165312?dopt=AbstractPlus]
Selective agonists	http://www.guidetopharmacology.org/GRAC/LigandDisplayForward?ligandId=3333	–
Labelled ligands	[http://www.guidetopharmacology.org/GRAC/LigandDisplayForward?ligandId=2901 (Agonist) [http://www.ncbi.nlm.nih.gov/pubmed/15753205?dopt=AbstractPlus, http://www.ncbi.nlm.nih.gov/pubmed/17339491?dopt=AbstractPlus]	–
Comments	–	Reported to act as a co‐receptor for HIV [http://www.ncbi.nlm.nih.gov/pubmed/10233994?dopt=AbstractPlus]. See review [http://www.ncbi.nlm.nih.gov/pubmed/23686350?dopt=AbstractPlus] for discussion of pairing with chemerin.

### Comments

CCX832 (structure not disclosed) is a selective antagonist, p*K*i=9.2 [http://www.ncbi.nlm.nih.gov/pubmed/27742615?dopt=AbstractPlus].

### Further reading on Chemerin receptors

Kennedy AJ *et al*. (2018) International Union of Basic and Clinical Pharmacology CIII: Chemerin Receptors CMKLR1 (Chemerin1) and GPR1 (Chemerin2) Nomenclature, Pharmacology, and Function. *Pharmacol. Rev.* **70**: 174‐196 [https://www.ncbi.nlm.nih.gov/pubmed/29279348?dopt=AbstractPlus]

Shin WJ *et al*. (2018) Mechanisms and Functions of Chemerin in Cancer: Potential Roles in Therapeutic Intervention. *Front Immunol* **9**: 2772 [https://www.ncbi.nlm.nih.gov/pubmed/30555465?dopt=AbstractPlus]

## http://www.guidetopharmacology.org/GRAC/FamilyDisplayForward?familyId=14

### Overview

Chemokine receptors (**nomenclature as agreed by the NC‐IUPHAR Subcommittee on Chemokine Receptors** [http://www.ncbi.nlm.nih.gov/pubmed/24218476?dopt=AbstractPlus, http://www.ncbi.nlm.nih.gov/pubmed/12037138?dopt=AbstractPlus, http://www.ncbi.nlm.nih.gov/pubmed/10699158?dopt=AbstractPlus]) comprise a large subfamily of 7TM proteins that bind one or more chemokines, a large family of small cytokines typically possessing chemotactic activity for leukocytes. Additional hematopoietic and non‐hematopoietic roles have been identified for many chemokines in the areas of embryonic development, immune cell proliferation, activation and death, viral infection, and as antibiotics, among others. Chemokine receptors can be divided by function into two main groups: G protein‐coupled chemokine receptors, which mediate leukocyte trafficking, and "Atypical chemokine receptors", which may signal through non‐G protein‐coupled mechanisms and act as chemokine scavengers to downregulate inflammation or shape chemokine gradients [http://www.ncbi.nlm.nih.gov/pubmed/24218476?dopt=AbstractPlus].

Chemokines in turn can be divided by structure into four subclasses by the number and arrangement of conserved cysteines. CC (also known as β‐chemokines; *n*= 28), CXC (also known as α‐chemokines; *n*= 17) and CX3C (*n*= 1) chemokines all have four conserved cysteines, with zero, one and three amino acids separating the first two cysteines respectively. C chemokines (*n*= 2) have only the second and fourth cysteines found in other chemokines. Chemokines can also be classified by function into homeostatic and inflammatory subgroups. Most chemokine receptors are able to bind multiple high‐affinity chemokine ligands, but the ligands for a given receptor are almost always restricted to the same structural subclass. Most chemokines bind to more than one receptor subtype. Receptors for inflammatory chemokines are typically highly promiscuous with regard to ligand specificity, and may lack a selective endogenous ligand. G protein‐coupled chemokine receptors are named acccording to the class of chemokines bound, whereas ACKR is the root acronym for atypical chemokine receptors [http://www.ncbi.nlm.nih.gov/pubmed/25958743?dopt=AbstractPlus]. There can be substantial cross‐species differences in the sequences of both chemokines and chemokine receptors, and in the pharmacology and biology of chemokine receptors. Endogenous and microbial non‐chemokine ligands have also been identified for chemokine receptors. Many chemokine receptors function as HIV co‐receptors, but CCR5 is the only one demonstrated to play an essential role in HIV/AIDS pathogenesis. The tables include bothstandard chemokine receptor names [http://www.ncbi.nlm.nih.gov/pubmed/10714678?dopt=AbstractPlus] and aliases.

**Table:** 

Nomenclature	http://www.guidetopharmacology.org/GRAC/ObjectDisplayForward?objectId=58	http://www.guidetopharmacology.org/GRAC/ObjectDisplayForward?objectId=59	http://www.guidetopharmacology.org/GRAC/ObjectDisplayForward?objectId=60
HGNC, UniProt	https://www.genenames.org/data/gene‐symbol‐report/#!/hgnc_id/HGNC:1602, http://www.uniprot.org/uniprot/P32246	https://www.genenames.org/data/gene‐symbol‐report/#!/hgnc_id/HGNC:1603, http://www.uniprot.org/uniprot/P41597	https://www.genenames.org/data/gene‐symbol‐report/#!/hgnc_id/HGNC:1604, http://www.uniprot.org/uniprot/P51677
Endogenous agonists	http://www.guidetopharmacology.org/GRAC/LigandDisplayForward?ligandId=756 (https://www.genenames.org/data/gene‐symbol‐report/#!/hgnc_id/HGNC:10627, http://www.uniprot.org/uniprot/P10147) [http://www.ncbi.nlm.nih.gov/pubmed/12381680?dopt=AbstractPlus, http://www.ncbi.nlm.nih.gov/pubmed/8530354?dopt=AbstractPlus, http://www.ncbi.nlm.nih.gov/pubmed/9624164?dopt=AbstractPlus, http://www.ncbi.nlm.nih.gov/pubmed/11170631?dopt=AbstractPlus], http://www.guidetopharmacology.org/GRAC/LigandDisplayForward?ligandId=755 (https://www.genenames.org/data/gene‐symbol‐report/#!/hgnc_id/HGNC:10622, http://www.uniprot.org/uniprot/P55773) http://www.uniprot.org/uniprot/P55773], http://www.guidetopharmacology.org/GRAC/LigandDisplayForward?ligandId=758 (https://www.genenames.org/data/gene‐symbol‐report/#!/hgnc_id/HGNC:10632, http://www.uniprot.org/uniprot/P13501) [http://www.ncbi.nlm.nih.gov/pubmed/8530354?dopt=AbstractPlus, http://www.ncbi.nlm.nih.gov/pubmed/8530354?dopt=AbstractPlus], http://www.guidetopharmacology.org/GRAC/LigandDisplayForward?ligandId=759 (https://www.genenames.org/data/gene‐symbol‐report/#!/hgnc_id/HGNC:10634, http://www.uniprot.org/uniprot/P80098) http://www.uniprot.org/uniprot/P80098, http://www.ncbi.nlm.nih.gov/pubmed/11994538?dopt=AbstractPlus], http://www.guidetopharmacology.org/GRAC/LigandDisplayForward?ligandId=754 (https://www.genenames.org/data/gene‐symbol‐report/#!/hgnc_id/HGNC:10613, http://www.uniprot.org/uniprot/Q16663) [http://www.ncbi.nlm.nih.gov/pubmed/9346309?dopt=AbstractPlus], http://www.guidetopharmacology.org/GRAC/LigandDisplayForward?ligandId=753 (https://www.genenames.org/data/gene‐symbol‐report/#!/hgnc_id/HGNC:10612, http://www.uniprot.org/uniprot/Q16627) http://www.uniprot.org/uniprot/Q16627], http://www.guidetopharmacology.org/GRAC/LigandDisplayForward?ligandId=770 (https://www.genenames.org/data/gene‐symbol‐report/#!/hgnc_id/HGNC:10611, http://www.uniprot.org/uniprot/Q99616), http://www.guidetopharmacology.org/GRAC/LigandDisplayForward?ligandId=772 (https://www.genenames.org/data/gene‐symbol‐report/#!/hgnc_id/HGNC:10635, http://www.uniprot.org/uniprot/P80075)	http://www.guidetopharmacology.org/GRAC/LigandDisplayForward?ligandId=771 (https://www.genenames.org/data/gene‐symbol‐report/#!/hgnc_id/HGNC:10618, http://www.uniprot.org/uniprot/P13500) [http://www.ncbi.nlm.nih.gov/pubmed/9346309?dopt=AbstractPlus, http://www.ncbi.nlm.nih.gov/pubmed/12554737?dopt=AbstractPlus, http://www.ncbi.nlm.nih.gov/pubmed/10770925?dopt=AbstractPlus, http://www.ncbi.nlm.nih.gov/pubmed/15207250?dopt=AbstractPlus, http://www.ncbi.nlm.nih.gov/pubmed/9276730?dopt=AbstractPlus], http://www.guidetopharmacology.org/GRAC/LigandDisplayForward?ligandId=770 (https://www.genenames.org/data/gene‐symbol‐report/#!/hgnc_id/HGNC:10611, http://www.uniprot.org/uniprot/Q99616) [http://www.ncbi.nlm.nih.gov/pubmed/12554737?dopt=AbstractPlus, http://www.ncbi.nlm.nih.gov/pubmed/12554737?dopt=AbstractPlus], http://www.guidetopharmacology.org/GRAC/LigandDisplayForward?ligandId=759 (https://www.genenames.org/data/gene‐symbol‐report/#!/hgnc_id/HGNC:10634, http://www.uniprot.org/uniprot/P80098) http://www.uniprot.org/uniprot/P80098, http://www.ncbi.nlm.nih.gov/pubmed/9346309?dopt=AbstractPlus, http://www.ncbi.nlm.nih.gov/pubmed/9276730?dopt=AbstractPlus], http://www.guidetopharmacology.org/GRAC/LigandDisplayForward?ligandId=769 (https://www.genenames.org/data/gene‐symbol‐report/#!/hgnc_id/HGNC:10610, http://www.uniprot.org/uniprot/P51671) (Partial agonist) [http://www.ncbi.nlm.nih.gov/pubmed/12554737?dopt=AbstractPlus, http://www.ncbi.nlm.nih.gov/pubmed/15207250?dopt=AbstractPlus], http://www.guidetopharmacology.org/GRAC/LigandDisplayForward?ligandId=1272 (https://www.genenames.org/data/gene‐symbol‐report/#!/hgnc_id/HGNC:10614, http://www.uniprot.org/uniprot/O15467)	http://www.guidetopharmacology.org/GRAC/LigandDisplayForward?ligandId=770 (https://www.genenames.org/data/gene‐symbol‐report/#!/hgnc_id/HGNC:10611, http://www.uniprot.org/uniprot/Q99616) [http://www.ncbi.nlm.nih.gov/pubmed/16339911?dopt=AbstractPlus, http://www.ncbi.nlm.nih.gov/pubmed/9276730?dopt=AbstractPlus], http://www.guidetopharmacology.org/GRAC/LigandDisplayForward?ligandId=775 (https://www.genenames.org/data/gene‐symbol‐report/#!/hgnc_id/HGNC:10623, http://www.uniprot.org/uniprot/O00175) http://www.uniprot.org/uniprot/O00175, http://www.ncbi.nlm.nih.gov/pubmed/15207250?dopt=AbstractPlus], http://www.guidetopharmacology.org/GRAC/LigandDisplayForward?ligandId=758 (https://www.genenames.org/data/gene‐symbol‐report/#!/hgnc_id/HGNC:10632, http://www.uniprot.org/uniprot/P13501) [http://www.ncbi.nlm.nih.gov/pubmed/8642344?dopt=AbstractPlus], http://www.guidetopharmacology.org/GRAC/LigandDisplayForward?ligandId=759 (https://www.genenames.org/data/gene‐symbol‐report/#!/hgnc_id/HGNC:10634, http://www.uniprot.org/uniprot/P80098) [http://www.ncbi.nlm.nih.gov/pubmed/8642344?dopt=AbstractPlus], http://www.guidetopharmacology.org/GRAC/LigandDisplayForward?ligandId=769 (https://www.genenames.org/data/gene‐symbol‐report/#!/hgnc_id/HGNC:10610, http://www.uniprot.org/uniprot/P51671) [http://www.ncbi.nlm.nih.gov/pubmed/12761559?dopt=AbstractPlus, http://www.ncbi.nlm.nih.gov/pubmed/10488147?dopt=AbstractPlus, http://www.ncbi.nlm.nih.gov/pubmed/10488147?dopt=AbstractPlus, http://www.ncbi.nlm.nih.gov/pubmed/10854442?dopt=AbstractPlus, http://www.ncbi.nlm.nih.gov/pubmed/9276730?dopt=AbstractPlus], http://www.guidetopharmacology.org/GRAC/LigandDisplayForward?ligandId=776 (https://www.genenames.org/data/gene‐symbol‐report/#!/hgnc_id/HGNC:10625, http://www.uniprot.org/uniprot/Q9Y258) [http://www.ncbi.nlm.nih.gov/pubmed/10488147?dopt=AbstractPlus, http://www.ncbi.nlm.nih.gov/pubmed/10488147?dopt=AbstractPlus, http://www.ncbi.nlm.nih.gov/pubmed/16339911?dopt=AbstractPlus], http://www.guidetopharmacology.org/GRAC/LigandDisplayForward?ligandId=754 (https://www.genenames.org/data/gene‐symbol‐report/#!/hgnc_id/HGNC:10613, http://www.uniprot.org/uniprot/Q16663) [http://www.ncbi.nlm.nih.gov/pubmed/9346309?dopt=AbstractPlus], http://www.guidetopharmacology.org/GRAC/LigandDisplayForward?ligandId=3649 (https://www.genenames.org/data/gene‐symbol‐report/#!/hgnc_id/HGNC:17700, http://www.uniprot.org/uniprot/Q9NRJ3), http://www.guidetopharmacology.org/GRAC/LigandDisplayForward?ligandId=772 (https://www.genenames.org/data/gene‐symbol‐report/#!/hgnc_id/HGNC:10635, http://www.uniprot.org/uniprot/P80075)
Agonists	–	–	http://www.guidetopharmacology.org/GRAC/LigandDisplayForward?ligandId=787 {Mouse} [http://www.ncbi.nlm.nih.gov/pubmed/8642344?dopt=AbstractPlus]
Endogenous antagonists	http://www.guidetopharmacology.org/GRAC/LigandDisplayForward?ligandId=757 (https://www.genenames.org/data/gene‐symbol‐report/#!/hgnc_id/HGNC:10630, http://www.uniprot.org/uniprot/P13236) (p*K*_i_ 7.1–7.8) [http://www.ncbi.nlm.nih.gov/pubmed/12381680?dopt=AbstractPlus, http://www.ncbi.nlm.nih.gov/pubmed/8530354?dopt=AbstractPlus]	http://www.guidetopharmacology.org/GRAC/LigandDisplayForward?ligandId=776 (https://www.genenames.org/data/gene‐symbol‐report/#!/hgnc_id/HGNC:10625, http://www.uniprot.org/uniprot/Q9Y258) (pIC_50_ 8.5) [http://www.ncbi.nlm.nih.gov/pubmed/15207250?dopt=AbstractPlus]	http://www.guidetopharmacology.org/GRAC/LigandDisplayForward?ligandId=835 (https://www.genenames.org/data/gene‐symbol‐report/#!/hgnc_id/HGNC:10637, http://www.uniprot.org/uniprot/P02778), http://www.guidetopharmacology.org/GRAC/LigandDisplayForward?ligandId=836 (https://www.genenames.org/data/gene‐symbol‐report/#!/hgnc_id/HGNC:10638, http://www.uniprot.org/uniprot/O14625), http://www.guidetopharmacology.org/GRAC/LigandDisplayForward?ligandId=837 (https://www.genenames.org/data/gene‐symbol‐report/#!/hgnc_id/HGNC:7098, http://www.uniprot.org/uniprot/Q07325)
Selective antagonists	http://www.guidetopharmacology.org/GRAC/LigandDisplayForward?ligandId=767 (p*K*_i_ 8.2–9) [http://www.ncbi.nlm.nih.gov/pubmed/10748002?dopt=AbstractPlus], http://www.guidetopharmacology.org/GRAC/LigandDisplayForward?ligandId=3696 (pIC_50_ 8.7) [http://www.ncbi.nlm.nih.gov/pubmed/12614873?dopt=AbstractPlus], http://www.guidetopharmacology.org/GRAC/LigandDisplayForward?ligandId=3536 (pIC_50_ 8) [http://www.ncbi.nlm.nih.gov/pubmed/10854442?dopt=AbstractPlus], http://www.guidetopharmacology.org/GRAC/LigandDisplayForward?ligandId=3497 (p*K*_d_ 8) [http://www.ncbi.nlm.nih.gov/pubmed/12909630?dopt=AbstractPlus]	http://www.guidetopharmacology.org/GRAC/LigandDisplayForward?ligandId=3502 (p*K*_i_ 7.6)	http://www.guidetopharmacology.org/GRAC/LigandDisplayForward?ligandId=796) (Inverse agonist) (p*K*_i_ 8.5) [http://www.ncbi.nlm.nih.gov/pubmed/12450563?dopt=AbstractPlus], http://www.guidetopharmacology.org/GRAC/LigandDisplayForward?ligandId=3530 (p*K*i 8.4), http://www.guidetopharmacology.org/GRAC/LigandDisplayForward?ligandId=3492 (p*K*_i_ 8.1) [http://www.ncbi.nlm.nih.gov/pubmed/12067561?dopt=AbstractPlus]
Labelled ligands	[http://www.guidetopharmacology.org/GRAC/LigandDisplayForward?ligandId=765) (Agonist) [http://www.ncbi.nlm.nih.gov/pubmed/7545673?dopt=AbstractPlus], [http://www.guidetopharmacology.org/GRAC/LigandDisplayForward?ligandId=763) (Agonist) [http://www.ncbi.nlm.nih.gov/pubmed/7545673?dopt=AbstractPlus, http://www.ncbi.nlm.nih.gov/pubmed/9115216?dopt=AbstractPlus, http://www.ncbi.nlm.nih.gov/pubmed/9336350?dopt=AbstractPlus], [http://www.guidetopharmacology.org/GRAC/LigandDisplayForward?ligandId=764) (Agonist) [http://www.ncbi.nlm.nih.gov/pubmed/9336350?dopt=AbstractPlus]	[http://www.guidetopharmacology.org/GRAC/LigandDisplayForward?ligandId=762) (Agonist), [http://www.guidetopharmacology.org/GRAC/LigandDisplayForward?ligandId=765) (Agonist)	[http://www.guidetopharmacology.org/GRAC/LigandDisplayForward?ligandId=794) (Antagonist) (p*K*_d_ 8.3) [http://www.ncbi.nlm.nih.gov/pubmed/12450563?dopt=AbstractPlus], [http://www.guidetopharmacology.org/GRAC/LigandDisplayForward?ligandId=764) (Agonist), [http://www.guidetopharmacology.org/GRAC/LigandDisplayForward?ligandId=765) (Agonist)

**Table:** 

Nomenclature	http://www.guidetopharmacology.org/GRAC/ObjectDisplayForward?objectId=61	http://www.guidetopharmacology.org/GRAC/ObjectDisplayForward?objectId=62	http://www.guidetopharmacology.org/GRAC/ObjectDisplayForward?objectId=63	http://www.guidetopharmacology.org/GRAC/ObjectDisplayForward?objectId=64	http://www.guidetopharmacology.org/GRAC/ObjectDisplayForward?objectId=65	http://www.guidetopharmacology.org/GRAC/ObjectDisplayForward?objectId=66	http://www.guidetopharmacology.org/GRAC/ObjectDisplayForward?objectId=67
HGNC, UniProt	https://www.genenames.org/data/gene‐symbol‐report/#!/hgnc_id/HGNC:1605, http://www.uniprot.org/uniprot/P51679	https://www.genenames.org/data/gene‐symbol‐report/#!/hgnc_id/HGNC:1606, http://www.uniprot.org/uniprot/P51681	https://www.genenames.org/data/gene‐symbol‐report/#!/hgnc_id/HGNC:1607, http://www.uniprot.org/uniprot/P51684	https://www.genenames.org/data/gene‐symbol‐report/#!/hgnc_id/HGNC:1608, http://www.uniprot.org/uniprot/P32248	https://www.genenames.org/data/gene‐symbol‐report/#!/hgnc_id/HGNC:1609, http://www.uniprot.org/uniprot/P51685	https://www.genenames.org/data/gene‐symbol‐report/#!/hgnc_id/HGNC:1610, http://www.uniprot.org/uniprot/P51686	https://www.genenames.org/data/gene‐symbol‐report/#!/hgnc_id/HGNC:4474, http://www.uniprot.org/uniprot/P46092
Endogenous agonists	http://www.guidetopharmacology.org/GRAC/LigandDisplayForward?ligandId=798 (https://www.genenames.org/data/gene‐symbol‐report/#!/hgnc_id/HGNC:10621, http://www.uniprot.org/uniprot/O00626) [http://www.ncbi.nlm.nih.gov/pubmed/9430724?dopt=AbstractPlus], http://www.guidetopharmacology.org/GRAC/LigandDisplayForward?ligandId=797 (https://www.genenames.org/data/gene‐symbol‐report/#!/hgnc_id/HGNC:10615, http://www.uniprot.org/uniprot/Q92583) http://www.uniprot.org/uniprot/Q92583]	http://www.guidetopharmacology.org/GRAC/LigandDisplayForward?ligandId=758 (https://www.genenames.org/data/gene‐symbol‐report/#!/hgnc_id/HGNC:10632, http://www.uniprot.org/uniprot/P13501) [http://www.ncbi.nlm.nih.gov/pubmed/10318947?dopt=AbstractPlus, http://www.ncbi.nlm.nih.gov/pubmed/16298345?dopt=AbstractPlus, http://www.ncbi.nlm.nih.gov/pubmed/9790730?dopt=AbstractPlus], http://www.guidetopharmacology.org/GRAC/LigandDisplayForward?ligandId=757 (https://www.genenames.org/data/gene‐symbol‐report/#!/hgnc_id/HGNC:10630, http://www.uniprot.org/uniprot/P13236 [http://www.ncbi.nlm.nih.gov/pubmed/16298345?dopt=AbstractPlus, http://www.ncbi.nlm.nih.gov/pubmed/9790730?dopt=AbstractPlus], http://www.guidetopharmacology.org/GRAC/LigandDisplayForward?ligandId=772 (https://www.genenames.org/data/gene‐symbol‐report/#!/hgnc_id/HGNC:10635, http://www.uniprot.org/uniprot/P80075) [http://www.ncbi.nlm.nih.gov/pubmed/9790730?dopt=AbstractPlus], http://www.guidetopharmacology.org/GRAC/LigandDisplayForward?ligandId=756 (https://www.genenames.org/data/gene‐symbol‐report/#!/hgnc_id/HGNC:10627, http://www.uniprot.org/uniprot/P10147)[http://www.ncbi.nlm.nih.gov/pubmed/16298345?dopt=AbstractPlus, http://www.ncbi.nlm.nih.gov/pubmed/16298345?dopt=AbstractPlus, http://www.ncbi.nlm.nih.gov/pubmed/11170631?dopt=AbstractPlus], http://www.guidetopharmacology.org/GRAC/LigandDisplayForward?ligandId=769 (https://www.genenames.org/data/gene‐symbol‐report/#!/hgnc_id/HGNC:10610, http://www.uniprot.org/uniprot/P51671) [http://www.ncbi.nlm.nih.gov/pubmed/10477718?dopt=AbstractPlus], http://www.guidetopharmacology.org/GRAC/LigandDisplayForward?ligandId=771 (https://www.genenames.org/data/gene‐symbol‐report/#!/hgnc_id/HGNC:10618, http://www.uniprot.org/uniprot/P13500 [http://www.ncbi.nlm.nih.gov/pubmed/16298345?dopt=AbstractPlus], http://www.guidetopharmacology.org/GRAC/LigandDisplayForward?ligandId=753 (https://www.genenames.org/data/gene‐symbol‐report/#!/hgnc_id/HGNC:10612, http://www.uniprot.org/uniprot/Q16627 [http://www.ncbi.nlm.nih.gov/pubmed/16298345?dopt=AbstractPlus], http://www.guidetopharmacology.org/GRAC/LigandDisplayForward?ligandId=1272 (https://www.genenames.org/data/gene‐symbol‐report/#!/hgnc_id/HGNC:10614, http://www.uniprot.org/uniprot/O15467)	http://www.guidetopharmacology.org/GRAC/LigandDisplayForward?ligandId=808 (https://www.genenames.org/data/gene‐symbol‐report/#!/hgnc_id/HGNC:10619, http://www.uniprot.org/uniprot/P78556) [http://www.ncbi.nlm.nih.gov/pubmed/12081481?dopt=AbstractPlus, http://www.ncbi.nlm.nih.gov/pubmed/9169459?dopt=AbstractPlus, http://www.ncbi.nlm.nih.gov/pubmed/9294137?dopt=AbstractPlus], http://www.guidetopharmacology.org/GRAC/LigandDisplayForward?ligandId=3648 (https://www.genenames.org/data/gene‐symbol‐report/#!/hgnc_id/HGNC:2767 https://www.genenames.org/data/gene‐symbol‐report/#!/hgnc_id/HGNC:30193, http://www.uniprot.org/uniprot/O15263) [http://www.ncbi.nlm.nih.gov/pubmed/10521347?dopt=AbstractPlus]	http://www.guidetopharmacology.org/GRAC/LigandDisplayForward?ligandId=811 (https://www.genenames.org/data/gene‐symbol‐report/#!/hgnc_id/HGNC:10620, http://www.uniprot.org/uniprot/O00585) [http://www.ncbi.nlm.nih.gov/pubmed/9507024?dopt=AbstractPlus], http://www.guidetopharmacology.org/GRAC/LigandDisplayForward?ligandId=810 (https://www.genenames.org/data/gene‐symbol‐report/#!/hgnc_id/HGNC:10617, http://www.uniprot.org/uniprot/Q99731) [http://www.ncbi.nlm.nih.gov/pubmed/16904643?dopt=AbstractPlus, http://www.ncbi.nlm.nih.gov/pubmed/9153236?dopt=AbstractPlus, http://www.ncbi.nlm.nih.gov/pubmed/9153236?dopt=AbstractPlus]	http://www.guidetopharmacology.org/GRAC/LigandDisplayForward?ligandId=812 (https://www.genenames.org/data/gene‐symbol‐report/#!/hgnc_id/HGNC:10609, http://www.uniprot.org/uniprot/P22362) [http://www.ncbi.nlm.nih.gov/pubmed/10419462?dopt=AbstractPlus, http://www.ncbi.nlm.nih.gov/pubmed/16221874?dopt=AbstractPlus, http://www.ncbi.nlm.nih.gov/pubmed/11154210?dopt=AbstractPlus], http://www.guidetopharmacology.org/GRAC/LigandDisplayForward?ligandId=4415 {Mouse}	http://www.guidetopharmacology.org/GRAC/LigandDisplayForward?ligandId=817 (https://www.genenames.org/data/gene‐symbol‐report/#!/hgnc_id/HGNC:10624, http://www.uniprot.org/uniprot/O15444)	http://www.guidetopharmacology.org/GRAC/LigandDisplayForward?ligandId=3646 (https://www.genenames.org/data/gene‐symbol‐report/#!/hgnc_id/HGNC:10626, http://www.uniprot.org/uniprot/Q9Y4X3) [http://www.ncbi.nlm.nih.gov/pubmed/10725697?dopt=AbstractPlus], http://www.guidetopharmacology.org/GRAC/LigandDisplayForward?ligandId=3649 (https://www.genenames.org/data/gene‐symbol‐report/#!/hgnc_id/HGNC:17700, http://www.uniprot.org/uniprot/Q9NRJ3)
Agonists	http://www.guidetopharmacology.org/GRAC/LigandDisplayForward?ligandId=4359	http://www.guidetopharmacology.org/GRAC/LigandDisplayForward?ligandId=3910	–	–	http://www.guidetopharmacology.org/GRAC/LigandDisplayForward?ligandId=815 [http://www.ncbi.nlm.nih.gov/pubmed/10419462?dopt=AbstractPlus, http://www.ncbi.nlm.nih.gov/pubmed/11154210?dopt=AbstractPlus]	–	–
Endogenous antagonists	–	http://www.guidetopharmacology.org/GRAC/LigandDisplayForward?ligandId=759 (https://www.genenames.org/data/gene‐symbol‐report/#!/hgnc_id/HGNC:10634, http://www.uniprot.org/uniprot/P80098) (p*K*_i_ 7.5) [http://www.ncbi.nlm.nih.gov/pubmed/16298345?dopt=AbstractPlus]	–	–	–	–	–
Antagonists	–	http://www.guidetopharmacology.org/GRAC/LigandDisplayForward?ligandId=807 (p*K*_i_ 9.1) [http://www.ncbi.nlm.nih.gov/pubmed/16304152?dopt=AbstractPlus], http://www.guidetopharmacology.org/GRAC/LigandDisplayForward?ligandId=804 (p*K*_i_ 7.8–8.7) [http://www.ncbi.nlm.nih.gov/pubmed/16476734?dopt=AbstractPlus, http://www.ncbi.nlm.nih.gov/pubmed/11585437?dopt=AbstractPlus, http://www.ncbi.nlm.nih.gov/pubmed/11585437?dopt=AbstractPlus]	–	–	–	–	–
Selective antagonists	http://www.guidetopharmacology.org/GRAC/LigandDisplayForward?ligandId=9478 (pIC_50_ 7.7) [http://www.ncbi.nlm.nih.gov/pubmed/19081254?dopt=AbstractPlus]	http://www.guidetopharmacology.org/GRAC/LigandDisplayForward?ligandId=3500 (pIC_50_ 8.7) [http://www.ncbi.nlm.nih.gov/pubmed/11454872?dopt=AbstractPlus], http://www.guidetopharmacology.org/GRAC/LigandDisplayForward?ligandId=805 (p*K*_i_ 8.5) [http://www.ncbi.nlm.nih.gov/pubmed/16476734?dopt=AbstractPlus], http://www.guidetopharmacology.org/GRAC/LigandDisplayForward?ligandId=806 (pIC_50_ 8.1) [http://www.ncbi.nlm.nih.gov/pubmed/16298345?dopt=AbstractPlus], http://www.guidetopharmacology.org/GRAC/LigandDisplayForward?ligandId=783 (p*K*_i_ 7.5) [http://www.ncbi.nlm.nih.gov/pubmed/16476734?dopt=AbstractPlus], http://www.guidetopharmacology.org/GRAC/LigandDisplayForward?ligandId=3651 [http://www.ncbi.nlm.nih.gov/pubmed/12604693?dopt=AbstractPlus] – Rat	–	–	http://www.guidetopharmacology.org/GRAC/LigandDisplayForward?ligandId=816 (pIC_50_ 9.4) [http://www.ncbi.nlm.nih.gov/pubmed/10419462?dopt=AbstractPlus]	–	–
Selective allosteric modulators	–	–	–	–	–	http://www.guidetopharmacology.org/GRAC/LigandDisplayForward?ligandId=9046 (Antagonist) (pIC_50_ 8.2) [http://www.ncbi.nlm.nih.gov/pubmed/20660125?dopt=AbstractPlus]	–
Antibodies	http://www.guidetopharmacology.org/GRAC/LigandDisplayForward?ligandId=6477 (Inhibition) [http://www.ncbi.nlm.nih.gov/pubmed/21154168?dopt=AbstractPlus, 1962]	–	–	–	–	–	–
Labelled ligands	[http://www.guidetopharmacology.org/GRAC/LigandDisplayForward?ligandId=3652) (Agonist), [http://www.guidetopharmacology.org/GRAC/LigandDisplayForward?ligandId=3653) (Agonist)	[http://www.guidetopharmacology.org/GRAC/LigandDisplayForward?ligandId=799) (Agonist) [http://www.ncbi.nlm.nih.gov/pubmed/16298345?dopt=AbstractPlus], [http://www.guidetopharmacology.org/GRAC/LigandDisplayForward?ligandId=763) (Agonist), [http://www.guidetopharmacology.org/GRAC/LigandDisplayForward?ligandId=764) (Agonist), [http://www.guidetopharmacology.org/GRAC/LigandDisplayForward?ligandId=766) (Agonist)	[http://www.guidetopharmacology.org/GRAC/LigandDisplayForward?ligandId=3654) (Agonist) [http://www.ncbi.nlm.nih.gov/pubmed/9294138?dopt=AbstractPlus]	[http://www.guidetopharmacology.org/GRAC/LigandDisplayForward?ligandId=3655) (Agonist), [http://www.guidetopharmacology.org/GRAC/LigandDisplayForward?ligandId=3656) (Agonist) [http://www.ncbi.nlm.nih.gov/pubmed/10201891?dopt=AbstractPlus]	[http://www.guidetopharmacology.org/GRAC/LigandDisplayForward?ligandId=814) (Agonist) [http://www.ncbi.nlm.nih.gov/pubmed/11154210?dopt=AbstractPlus, http://www.ncbi.nlm.nih.gov/pubmed/9211859?dopt=AbstractPlus]	[http://www.guidetopharmacology.org/GRAC/LigandDisplayForward?ligandId=3657) (Agonist)	–

**Table:** 

Nomenclature	http://www.guidetopharmacology.org/GRAC/ObjectDisplayForward?objectId=68	http://www.guidetopharmacology.org/GRAC/ObjectDisplayForward?objectId=69	http://www.guidetopharmacology.org/GRAC/ObjectDisplayForward?objectId=70	http://www.guidetopharmacology.org/GRAC/ObjectDisplayForward?objectId=71	http://www.guidetopharmacology.org/GRAC/ObjectDisplayForward?objectId=72	http://www.guidetopharmacology.org/GRAC/ObjectDisplayForward?objectId=73	http://www.guidetopharmacology.org/GRAC/ObjectDisplayForward?objectId=74
HGNC, UniProt	https://www.genenames.org/data/gene‐symbol‐report/#!/hgnc_id/HGNC:6026, http://www.uniprot.org/uniprot/P25024	https://www.genenames.org/data/gene‐symbol‐report/#!/hgnc_id/HGNC:6027, http://www.uniprot.org/uniprot/P25025	https://www.genenames.org/data/gene‐symbol‐report/#!/hgnc_id/HGNC:4540, http://www.uniprot.org/uniprot/P49682	https://www.genenames.org/data/gene‐symbol‐report/#!/hgnc_id/HGNC:2561, http://www.uniprot.org/uniprot/P61073	https://www.genenames.org/data/gene‐symbol‐report/#!/hgnc_id/HGNC:1060, http://www.uniprot.org/uniprot/P32302	https://www.genenames.org/data/gene‐symbol‐report/#!/hgnc_id/HGNC:16647, http://www.uniprot.org/uniprot/O00574	https://www.genenames.org/data/gene‐symbol‐report/#!/hgnc_id/HGNC:2558, http://www.uniprot.org/uniprot/P49238
Endogenous agonists	http://www.guidetopharmacology.org/GRAC/LigandDisplayForward?ligandId=821 (https://www.genenames.org/data/gene‐symbol‐report/#!/hgnc_id/HGNC:6025, http://www.uniprot.org/uniprot/P10145) [http://www.ncbi.nlm.nih.gov/pubmed/15282370?dopt=AbstractPlus, http://www.ncbi.nlm.nih.gov/pubmed/10188995?dopt=AbstractPlus, http://www.ncbi.nlm.nih.gov/pubmed/1379593?dopt=AbstractPlus, http://www.ncbi.nlm.nih.gov/pubmed/15946947?dopt=AbstractPlus, http://www.ncbi.nlm.nih.gov/pubmed/8940121?dopt=AbstractPlus], http://www.guidetopharmacology.org/GRAC/LigandDisplayForward?ligandId=820 (https://www.genenames.org/data/gene‐symbol‐report/#!/hgnc_id/HGNC:10643, http://www.uniprot.org/uniprot/P80162) [http://www.ncbi.nlm.nih.gov/pubmed/9692902?dopt=AbstractPlus]	http://www.guidetopharmacology.org/GRAC/LigandDisplayForward?ligandId=819 (https://www.genenames.org/data/gene‐symbol‐report/#!/hgnc_id/HGNC:4602, http://www.uniprot.org/uniprot/P09341) [http://www.ncbi.nlm.nih.gov/pubmed/10188995?dopt=AbstractPlus, http://www.ncbi.nlm.nih.gov/pubmed/1379593?dopt=AbstractPlus, http://www.ncbi.nlm.nih.gov/pubmed/8940121?dopt=AbstractPlus], http://www.guidetopharmacology.org/GRAC/LigandDisplayForward?ligandId=821 (https://www.genenames.org/data/gene‐symbol‐report/#!/hgnc_id/HGNC:6025, http://www.uniprot.org/uniprot/P10145) [http://www.ncbi.nlm.nih.gov/pubmed/15282370?dopt=AbstractPlus, http://www.ncbi.nlm.nih.gov/pubmed/15282370?dopt=AbstractPlus, http://www.ncbi.nlm.nih.gov/pubmed/1379593?dopt=AbstractPlus, http://www.ncbi.nlm.nih.gov/pubmed/15946947?dopt=AbstractPlus, http://www.ncbi.nlm.nih.gov/pubmed/8940121?dopt=AbstractPlus], http://www.guidetopharmacology.org/GRAC/LigandDisplayForward?ligandId=830 (https://www.genenames.org/data/gene‐symbol‐report/#!/hgnc_id/HGNC:9240, http://www.uniprot.org/uniprot/P02775) [http://www.ncbi.nlm.nih.gov/pubmed/8702798?dopt=AbstractPlus], http://www.guidetopharmacology.org/GRAC/LigandDisplayForward?ligandId=828 (https://www.genenames.org/data/gene‐symbol‐report/#!/hgnc_id/HGNC:4604, http://www.uniprot.org/uniprot/P19876) http://www.uniprot.org/uniprot/P19876], http://www.guidetopharmacology.org/GRAC/LigandDisplayForward?ligandId=827 (https://www.genenames.org/data/gene‐symbol‐report/#!/hgnc_id/HGNC:4603, http://www.uniprot.org/uniprot/P19875) http://www.uniprot.org/uniprot/P19875], http://www.guidetopharmacology.org/GRAC/LigandDisplayForward?ligandId=829 (https://www.genenames.org/data/gene‐symbol‐report/#!/hgnc_id/HGNC:10642, http://www.uniprot.org/uniprot/P42830) http://www.uniprot.org/uniprot/P42830], http://www.guidetopharmacology.org/GRAC/LigandDisplayForward?ligandId=820 (https://www.genenames.org/data/gene‐symbol‐report/#!/hgnc_id/HGNC:10643, http://www.uniprot.org/uniprot/P80162) [http://www.ncbi.nlm.nih.gov/pubmed/9692902?dopt=AbstractPlus]	http://www.guidetopharmacology.org/GRAC/LigandDisplayForward?ligandId=836 (https://www.genenames.org/data/gene‐symbol‐report/#!/hgnc_id/HGNC:10638, http://www.uniprot.org/uniprot/O14625) [http://www.ncbi.nlm.nih.gov/pubmed/15761110?dopt=AbstractPlus], http://www.guidetopharmacology.org/GRAC/LigandDisplayForward?ligandId=835 (https://www.genenames.org/data/gene‐symbol‐report/#!/hgnc_id/HGNC:10637, http://www.uniprot.org/uniprot/P02778) http://www.uniprot.org/uniprot/P02778, http://www.ncbi.nlm.nih.gov/pubmed/9660793?dopt=AbstractPlus], http://www.guidetopharmacology.org/GRAC/LigandDisplayForward?ligandId=837 (https://www.genenames.org/data/gene‐symbol‐report/#!/hgnc_id/HGNC:7098, http://www.uniprot.org/uniprot/Q07325) http://www.uniprot.org/uniprot/Q07325, http://www.ncbi.nlm.nih.gov/pubmed/15761110?dopt=AbstractPlus]	http://www.guidetopharmacology.org/GRAC/LigandDisplayForward?ligandId=4358 (https://www.genenames.org/data/gene‐symbol‐report/#!/hgnc_id/HGNC:10672, http://www.uniprot.org/uniprot/P48061) [http://www.ncbi.nlm.nih.gov/pubmed/9551924?dopt=AbstractPlus, http://www.ncbi.nlm.nih.gov/pubmed/9712844?dopt=AbstractPlus], http://www.guidetopharmacology.org/GRAC/LigandDisplayForward?ligandId=845 (https://www.genenames.org/data/gene‐symbol‐report/#!/hgnc_id/HGNC:10672, https://www.genenames.org/data/gene‐symbol‐report/#!/hgnc_id/HGNC:10672) [http://www.ncbi.nlm.nih.gov/pubmed/9551924?dopt=AbstractPlus]	http://www.guidetopharmacology.org/GRAC/LigandDisplayForward?ligandId=3645 (https://www.genenames.org/data/gene‐symbol‐report/#!/hgnc_id/HGNC:10639, http://www.uniprot.org/uniprot/O43927) [http://www.ncbi.nlm.nih.gov/pubmed/22913878?dopt=AbstractPlus]	http://www.guidetopharmacology.org/GRAC/LigandDisplayForward?ligandId=855 (https://www.genenames.org/data/gene‐symbol‐report/#!/hgnc_id/HGNC:16642, http://www.uniprot.org/uniprot/Q9H2A7) [http://www.ncbi.nlm.nih.gov/pubmed/11290797?dopt=AbstractPlus]	http://www.guidetopharmacology.org/GRAC/LigandDisplayForward?ligandId=856 (https://www.genenames.org/data/gene‐symbol‐report/#!/hgnc_id/HGNC:10647, http://www.uniprot.org/uniprot/P78423) [http://www.ncbi.nlm.nih.gov/pubmed/14607932?dopt=AbstractPlus]
Agonists	http://www.guidetopharmacology.org/GRAC/LigandDisplayForward?ligandId=8496 [http://www.ncbi.nlm.nih.gov/pubmed/20044480?dopt=AbstractPlus], http://www.guidetopharmacology.org/GRAC/LigandDisplayForward?ligandId=8495 [http://www.ncbi.nlm.nih.gov/pubmed/22262769?dopt=AbstractPlus]	http://www.guidetopharmacology.org/GRAC/LigandDisplayForward?ligandId=8496 [http://www.ncbi.nlm.nih.gov/pubmed/20044480?dopt=AbstractPlus], http://www.guidetopharmacology.org/GRAC/LigandDisplayForward?ligandId=8495 [http://www.ncbi.nlm.nih.gov/pubmed/22262769?dopt=AbstractPlus]	–	–	–	–	–
Selective agonists	–	–	–	http://www.guidetopharmacology.org/GRAC/LigandDisplayForward?ligandId=607 (Partial agonist) [http://www.ncbi.nlm.nih.gov/pubmed/11923301?dopt=AbstractPlus], http://www.guidetopharmacology.org/GRAC/LigandDisplayForward?ligandId=3919	–	–	–
Endogenous antagonists	–	–	http://www.guidetopharmacology.org/GRAC/LigandDisplayForward?ligandId=769 (https://www.genenames.org/data/gene‐symbol‐report/#!/hgnc_id/HGNC:10610, http://www.uniprot.org/uniprot/P51671) (p*K*_i_ 7.2) [http://www.ncbi.nlm.nih.gov/pubmed/9660793?dopt=AbstractPlus], http://www.guidetopharmacology.org/GRAC/LigandDisplayForward?ligandId=759 (https://www.genenames.org/data/gene‐symbol‐report/#!/hgnc_id/HGNC:10634, http://www.uniprot.org/uniprot/P80098) (p*K*_i_ 6.6) [http://www.ncbi.nlm.nih.gov/pubmed/9660793?dopt=AbstractPlus]	–	–	–	–
Antagonists	–	–	–	http://www.guidetopharmacology.org/GRAC/LigandDisplayForward?ligandId=844 (p*K*_i_ 7) [http://www.ncbi.nlm.nih.gov/pubmed/11923301?dopt=AbstractPlus]	–	–	–
Selective antagonists	–	http://www.guidetopharmacology.org/GRAC/LigandDisplayForward?ligandId=8497 (pIC_50_ 10.3) [http://www.ncbi.nlm.nih.gov/pubmed/24218476?dopt=AbstractPlus, http://www.ncbi.nlm.nih.gov/pubmed/17181143?dopt=AbstractPlus], http://www.guidetopharmacology.org/GRAC/LigandDisplayForward?ligandId=8500 (pIC_50_ 7.9) [http://www.ncbi.nlm.nih.gov/pubmed/26092545?dopt=AbstractPlus], http://www.guidetopharmacology.org/GRAC/LigandDisplayForward?ligandId=833 (pIC_50_ 7.7) [http://www.ncbi.nlm.nih.gov/pubmed/9553055?dopt=AbstractPlus], http://www.guidetopharmacology.org/GRAC/LigandDisplayForward?ligandId=8499 (pIC_50_ 7.7) [http://www.ncbi.nlm.nih.gov/pubmed/24218476?dopt=AbstractPlus], http://www.guidetopharmacology.org/GRAC/LigandDisplayForward?ligandId=8501 (pIC_50_ 7.2) [http://www.ncbi.nlm.nih.gov/pubmed/25254640?dopt=AbstractPlus]	–	http://www.guidetopharmacology.org/GRAC/LigandDisplayForward?ligandId=852 (pIC_50_ 8.4) [http://www.ncbi.nlm.nih.gov/pubmed/9918823?dopt=AbstractPlus], http://www.guidetopharmacology.org/GRAC/LigandDisplayForward?ligandId=8580 (pIC_50_ 7.9) [http://www.ncbi.nlm.nih.gov/pubmed/20297846?dopt=AbstractPlus], http://www.guidetopharmacology.org/GRAC/LigandDisplayForward?ligandId=773	–	–	–
Allosteric modulators	http://www.guidetopharmacology.org/GRAC/LigandDisplayForward?ligandId=8498 (Negative) (pIC_50_ 9) [http://www.ncbi.nlm.nih.gov/pubmed/15282370?dopt=AbstractPlus]	http://www.guidetopharmacology.org/GRAC/LigandDisplayForward?ligandId=8498 (Negative) (pIC_50_ 6.4) [http://www.ncbi.nlm.nih.gov/pubmed/15282370?dopt=AbstractPlus]	–	–	–	–	–
Labelled ligands	[http://www.guidetopharmacology.org/GRAC/LigandDisplayForward?ligandId=822) (Agonist) [http://www.ncbi.nlm.nih.gov/pubmed/10188995?dopt=AbstractPlus, http://www.ncbi.nlm.nih.gov/pubmed/12626541?dopt=AbstractPlus]	[http://www.guidetopharmacology.org/GRAC/LigandDisplayForward?ligandId=822) (Agonist) [http://www.ncbi.nlm.nih.gov/pubmed/10188995?dopt=AbstractPlus, http://www.ncbi.nlm.nih.gov/pubmed/12626541?dopt=AbstractPlus], [http://www.guidetopharmacology.org/GRAC/LigandDisplayForward?ligandId=3677) (Agonist), [http://www.guidetopharmacology.org/GRAC/LigandDisplayForward?ligandId=3659) (Agonist), [http://www.guidetopharmacology.org/GRAC/LigandDisplayForward?ligandId=3658) (Agonist)	[http://www.guidetopharmacology.org/GRAC/LigandDisplayForward?ligandId=3660) (Agonist), [http://www.guidetopharmacology.org/GRAC/LigandDisplayForward?ligandId=3661) (Agonist)	[http://www.guidetopharmacology.org/GRAC/LigandDisplayForward?ligandId=849 (http://www.guidetopharmacology.org/GRAC/LigandDisplayForward?ligandId=849) (Agonist) [http://www.ncbi.nlm.nih.gov/pubmed/11104827?dopt=AbstractPlus, http://www.ncbi.nlm.nih.gov/pubmed/9551924?dopt=AbstractPlus]	[http://www.guidetopharmacology.org/GRAC/LigandDisplayForward?ligandId=8631) (Agonist) [http://www.ncbi.nlm.nih.gov/pubmed/24190631?dopt=AbstractPlus] – Mouse	[http://www.guidetopharmacology.org/GRAC/LigandDisplayForward?ligandId=3662) (Agonist)	[http://www.guidetopharmacology.org/GRAC/LigandDisplayForward?ligandId=3664) (Agonist)

**Table:** 

Nomenclature	http://www.guidetopharmacology.org/GRAC/ObjectDisplayForward?objectId=75	http://www.guidetopharmacology.org/GRAC/ObjectDisplayForward?objectId=316	http://www.guidetopharmacology.org/GRAC/ObjectDisplayForward?objectId=314	http://www.guidetopharmacology.org/GRAC/ObjectDisplayForward?objectId=80	http://www.guidetopharmacology.org/GRAC/ObjectDisplayForward?objectId=315	http://www.guidetopharmacology.org/GRAC/ObjectDisplayForward?objectId=78
HGNC, UniProt	https://www.genenames.org/data/gene‐symbol‐report/#!/hgnc_id/HGNC:1625, http://www.uniprot.org/uniprot/P46094	https://www.genenames.org/data/gene‐symbol‐report/#!/hgnc_id/HGNC:4035, http://www.uniprot.org/uniprot/Q16570	https://www.genenames.org/data/gene‐symbol‐report/#!/hgnc_id/HGNC:1565, http://www.uniprot.org/uniprot/O00590	https://www.genenames.org/data/gene‐symbol‐report/#!/hgnc_id/HGNC:23692, http://www.uniprot.org/uniprot/P25106	https://www.genenames.org/data/gene‐symbol‐report/#!/hgnc_id/HGNC:1611, http://www.uniprot.org/uniprot/Q9NPB9	https://www.genenames.org/data/gene‐symbol‐report/#!/hgnc_id/HGNC:1612, http://www.uniprot.org/uniprot/O00421
Endogenous ligands	–	http://www.guidetopharmacology.org/GRAC/LigandDisplayForward?ligandId=829 (https://www.genenames.org/data/gene‐symbol‐report/#!/hgnc_id/HGNC:10642, http://www.uniprot.org/uniprot/P42830), http://www.guidetopharmacology.org/GRAC/LigandDisplayForward?ligandId=820 (https://www.genenames.org/data/gene‐symbol‐report/#!/hgnc_id/HGNC:10643, http://www.uniprot.org/uniprot/P80162), http://www.guidetopharmacology.org/GRAC/LigandDisplayForward?ligandId=821 (https://www.genenames.org/data/gene‐symbol‐report/#!/hgnc_id/HGNC:6025, http://www.uniprot.org/uniprot/P10145), http://www.guidetopharmacology.org/GRAC/LigandDisplayForward?ligandId=836 (https://www.genenames.org/data/gene‐symbol‐report/#!/hgnc_id/HGNC:10638, http://www.uniprot.org/uniprot/O14625), http://www.guidetopharmacology.org/GRAC/LigandDisplayForward?ligandId=771 (https://www.genenames.org/data/gene‐symbol‐report/#!/hgnc_id/HGNC:10618, http://www.uniprot.org/uniprot/P13500), http://www.guidetopharmacology.org/GRAC/LigandDisplayForward?ligandId=758 (https://www.genenames.org/data/gene‐symbol‐report/#!/hgnc_id/HGNC:10632, http://www.uniprot.org/uniprot/P13501), http://www.guidetopharmacology.org/GRAC/LigandDisplayForward?ligandId=759 (https://www.genenames.org/data/gene‐symbol‐report/#!/hgnc_id/HGNC:10634, http://www.uniprot.org/uniprot/P80098), http://www.guidetopharmacology.org/GRAC/LigandDisplayForward?ligandId=769 (https://www.genenames.org/data/gene‐symbol‐report/#!/hgnc_id/HGNC:10610, http://www.uniprot.org/uniprot/P51671), http://www.guidetopharmacology.org/GRAC/LigandDisplayForward?ligandId=753 (https://www.genenames.org/data/gene‐symbol‐report/#!/hgnc_id/HGNC:10612, http://www.uniprot.org/uniprot/Q16627), http://www.guidetopharmacology.org/GRAC/LigandDisplayForward?ligandId=797 (https://www.genenames.org/data/gene‐symbol‐report/#!/hgnc_id/HGNC:10615, http://www.uniprot.org/uniprot/Q92583)	–	–	–	http://www.guidetopharmacology.org/GRAC/LigandDisplayForward?ligandId=3422, http://www.guidetopharmacology.org/GRAC/LigandDisplayForward?ligandId=810 (https://www.genenames.org/data/gene‐symbol‐report/#!/hgnc_id/HGNC:10617, http://www.uniprot.org/uniprot/Q99731) [http://www.ncbi.nlm.nih.gov/pubmed/18165312?dopt=AbstractPlus]
Endogenous agonists	http://www.guidetopharmacology.org/GRAC/LigandDisplayForward?ligandId=3647 (https://www.genenames.org/data/gene‐symbol‐report/#!/hgnc_id/HGNC:10645, http://www.uniprot.org/uniprot/P47992) [http://www.ncbi.nlm.nih.gov/pubmed/25497737?dopt=AbstractPlus], http://www.guidetopharmacology.org/GRAC/LigandDisplayForward?ligandId=4370 (https://www.genenames.org/data/gene‐symbol‐report/#!/hgnc_id/HGNC:10646, http://www.uniprot.org/uniprot/Q9UBD3) http://www.uniprot.org/uniprot/Q9UBD3]	–	http://www.guidetopharmacology.org/GRAC/LigandDisplayForward?ligandId=771 (https://www.genenames.org/data/gene‐symbol‐report/#!/hgnc_id/HGNC:10618, http://www.uniprot.org/uniprot/P13500), http://www.guidetopharmacology.org/GRAC/LigandDisplayForward?ligandId=756 (https://www.genenames.org/data/gene‐symbol‐report/#!/hgnc_id/HGNC:10627, http://www.uniprot.org/uniprot/P10147), http://www.guidetopharmacology.org/GRAC/LigandDisplayForward?ligandId=757 (https://www.genenames.org/data/gene‐symbol‐report/#!/hgnc_id/HGNC:10630, http://www.uniprot.org/uniprot/P13236), http://www.guidetopharmacology.org/GRAC/LigandDisplayForward?ligandId=758 (https://www.genenames.org/data/gene‐symbol‐report/#!/hgnc_id/HGNC:10632, http://www.uniprot.org/uniprot/P13501), http://www.guidetopharmacology.org/GRAC/LigandDisplayForward?ligandId=759 (https://www.genenames.org/data/gene‐symbol‐report/#!/hgnc_id/HGNC:10634, http://www.uniprot.org/uniprot/P80098), http://www.guidetopharmacology.org/GRAC/LigandDisplayForward?ligandId=772 (https://www.genenames.org/data/gene‐symbol‐report/#!/hgnc_id/HGNC:10635, http://www.uniprot.org/uniprot/P80075), http://www.guidetopharmacology.org/GRAC/LigandDisplayForward?ligandId=769 (https://www.genenames.org/data/gene‐symbol‐report/#!/hgnc_id/HGNC:10610, http://www.uniprot.org/uniprot/P51671), http://www.guidetopharmacology.org/GRAC/LigandDisplayForward?ligandId=770 (https://www.genenames.org/data/gene‐symbol‐report/#!/hgnc_id/HGNC:10611, http://www.uniprot.org/uniprot/Q99616), http://www.guidetopharmacology.org/GRAC/LigandDisplayForward?ligandId=753 (https://www.genenames.org/data/gene‐symbol‐report/#!/hgnc_id/HGNC:10612, http://www.uniprot.org/uniprot/Q16627), http://www.guidetopharmacology.org/GRAC/LigandDisplayForward?ligandId=797 (https://www.genenames.org/data/gene‐symbol‐report/#!/hgnc_id/HGNC:10615, http://www.uniprot.org/uniprot/Q92583), http://www.guidetopharmacology.org/GRAC/LigandDisplayForward?ligandId=798 (https://www.genenames.org/data/gene‐symbol‐report/#!/hgnc_id/HGNC:10621, http://www.uniprot.org/uniprot/O00626)	http://www.guidetopharmacology.org/GRAC/LigandDisplayForward?ligandId=4358 (https://www.genenames.org/data/gene‐symbol‐report/#!/hgnc_id/HGNC:10672, http://www.uniprot.org/uniprot/P48061) [http://www.ncbi.nlm.nih.gov/pubmed/20956518?dopt=AbstractPlus, http://www.ncbi.nlm.nih.gov/pubmed/23396314?dopt=AbstractPlus], http://www.guidetopharmacology.org/GRAC/LigandDisplayForward?ligandId=836 (https://www.genenames.org/data/gene‐symbol‐report/#!/hgnc_id/HGNC:10638, http://www.uniprot.org/uniprot/O14625)	http://www.guidetopharmacology.org/GRAC/LigandDisplayForward?ligandId=810 (https://www.genenames.org/data/gene‐symbol‐report/#!/hgnc_id/HGNC:10617, http://www.uniprot.org/uniprot/Q99731) [http://www.ncbi.nlm.nih.gov/pubmed/23341447?dopt=AbstractPlus], http://www.guidetopharmacology.org/GRAC/LigandDisplayForward?ligandId=817 (https://www.genenames.org/data/gene‐symbol‐report/#!/hgnc_id/HGNC:10624, http://www.uniprot.org/uniprot/O15444) http://www.uniprot.org/uniprot/O15444], http://www.guidetopharmacology.org/GRAC/LigandDisplayForward?ligandId=811 (https://www.genenames.org/data/gene‐symbol‐report/#!/hgnc_id/HGNC:10620, http://www.uniprot.org/uniprot/O00585) http://www.uniprot.org/uniprot/O00585]	–
Comments	XCL1 cannot be iodinated, but a secreted alkaline phophatase (SEAP)‐XCL1 fusion peptide can be used as a probe at XCR1.	ACKR1 is used by *Plasmodium vivax* and *Plasmodium knowlsei* for entering erythrocytes.	–	Several lines of evidence have suggested that CGRP and adrenomedullin could be ligands for ACKR3; however, classical direct binding to the receptor has not yet been convincingly demonstrated [http://www.ncbi.nlm.nih.gov/pubmed/29530506?dopt=AbstractPlus].	–	–

### Comments

Specific chemokine receptors facilitate cell entry by microbes, such as ACKR1 for *Plasmodium vivax*, and CCR5 and CXCR4 for HIV‐1. Virally encoded chemokine receptors are known (*e.g.* US28, a homologue of CCR1 from human cytomegalovirus and ORF74, which encodes a homolog of CXCR2 in *Herpesvirus saimiri* and gamma‐Herpesvirus‐68), but their role in viral life cycles is not established. Viruses can exploit or subvert the chemokine system by producing chemokine antagonists and scavengers. Three chemokine receptor antagonists have now been approved by the FDA: 1) the CCR5 antagonist http://www.guidetopharmacology.org/GRAC/LigandDisplayForward?ligandId=806 (Pfizer) for treatment of HIV/AIDS in patients with CCR5‐using strains; and 2) the CXCR4 antagonist http://www.guidetopharmacology.org/GRAC/LigandDisplayForward?ligandId=844 (Sanofi) for hematopoietic stem cell mobilization with http://www.guidetopharmacology.org/GRAC/LigandDisplayForward?ligandId=4934 (https://www.genenames.org/data/gene‐symbol‐report/#!/hgnc_id/HGNC:2438, http://www.uniprot.org/uniprot/P09919) in patients undergoing transplantation in the context of chemotherapy for Hodgkins’ Disease and multiple myeloma; and 3) the CCR4 blocking antibody Poteligeo (mogamulizumab‐kpkc, Kyowa Kirin, Inc.) for mycosis fungoides or Sezary syndrome.

### Further reading on Chemokine receptors

Bachelerie F *et al*. (2015) An atypical addition to the chemokine receptor nomenclature: IUPHAR Review 15. *Br. J. Pharmacol.* **172**: 3945‐9 [https://www.ncbi.nlm.nih.gov/pubmed/25958743?dopt=AbstractPlus]

Murphy PM *et al*. (2000) International Union of Pharmacology. XXII. Nomenclature for chemokine receptors. *Pharmacol. Rev.* **52**: 145‐176 [https://www.ncbi.nlm.nih.gov/pubmed/10699158?dopt=AbstractPlus]

Koelink PJ *et al*. (2012) Targeting chemokine receptors in chronic inflammatory diseases: an extensive review. *Pharmacol. Ther.* **133**: 1‐18 [https://www.ncbi.nlm.nih.gov/pubmed/21839114?dopt=AbstractPlus]

Scholten DJ *et al*. (2012) Pharmacological modulation of chemokine receptor function. *Br. J. Pharmacol.* **165**: 1617‐43 [https://www.ncbi.nlm.nih.gov/pubmed/21699506?dopt=AbstractPlus]

Murphy PM. (2002) International Union of Pharmacology. XXX. Update on chemokine receptor nomenclature. *Pharmacol. Rev.* **54**: 227‐9 [https://www.ncbi.nlm.nih.gov/pubmed/12037138?dopt=AbstractPlus]

## http://www.guidetopharmacology.org/GRAC/FamilyDisplayForward?familyId=15

### Overview

Cholecystokinin receptors (**nomenclature as agreed by the NC‐IUPHAR Subcommittee on CCK receptors** [http://www.ncbi.nlm.nih.gov/pubmed/10581329?dopt=AbstractPlus) are activated by the endogenous peptides cholecystokinin‐8 (http://www.guidetopharmacology.org/GRAC/LigandDisplayForward?ligandId=864 (https://www.genenames.org/data/gene‐symbol‐report/#!/hgnc_id/HGNC:1569, http://www.uniprot.org/uniprot/P06307)), http://www.guidetopharmacology.org/GRAC/LigandDisplayForward?ligandId=860, https://www.genenames.org/data/gene‐symbol‐report/#!/hgnc_id/HGNC:1569), http://www.guidetopharmacology.org/GRAC/LigandDisplayForward?ligandId=3552, https://www.genenames.org/data/gene‐symbol‐report/#!/hgnc_id/HGNC:1569) and gastrin (http://www.guidetopharmacology.org/GRAC/LigandDisplayForward?ligandId=3559 (https://www.genenames.org/data/gene‐symbol‐report/#!/hgnc_id/HGNC:4164, http://www.uniprot.org/uniprot/P01350)). There are only two distinct subtypes of CCK receptors, CCK_1_ and CCK_2_ receptors [http://www.ncbi.nlm.nih.gov/pubmed/1373504?dopt=AbstractPlus, http://www.ncbi.nlm.nih.gov/pubmed/1313582?dopt=AbstractPlus], with some alternatively spliced forms most often identified in neoplastic cells. The CCK receptor subtypes are distinguished by their peptide selectivity, with the CCK_1_ receptor requiring the carboxyl‐terminal heptapeptide‐amide that includes a sulfated tyrosine for high affinity and potency, while the CCK_2_ receptor requires only the carboxyl‐terminal tetrapeptide shared by each CCK and gastrin peptides. These receptors have characteristic and distinct distributions, with both present in both the central nervous system and peripheral tissues.

**Table:** 

Nomenclature	http://www.guidetopharmacology.org/GRAC/ObjectDisplayForward?objectId=76	http://www.guidetopharmacology.org/GRAC/ObjectDisplayForward?objectId=77
HGNC, UniProt	https://www.genenames.org/data/gene‐symbol‐report/#!/hgnc_id/HGNC:1570, http://www.uniprot.org/uniprot/P32238	https://www.genenames.org/data/gene‐symbol‐report/#!/hgnc_id/HGNC:1571, http://www.uniprot.org/uniprot/P32239
Potency order of endogenous ligands	http://www.guidetopharmacology.org/GRAC/LigandDisplayForward?ligandId=864 (https://www.genenames.org/data/gene‐symbol‐report/#!/hgnc_id/HGNC:1569, http://www.uniprot.org/uniprot/P06307), http://www.guidetopharmacology.org/GRAC/LigandDisplayForward?ligandId=3552, https://www.genenames.org/data/gene‐symbol‐report/#!/hgnc_id/HGNC:1569), http://www.guidetopharmacology.org/GRAC/LigandDisplayForward?ligandId=10221), http://www.guidetopharmacology.org/GRAC/LigandDisplayForward?ligandId=860, https://www.genenames.org/data/gene‐symbol‐report/#!/hgnc_id/HGNC:1569) http://www.guidetopharmacology.org/GRAC/LigandDisplayForward?ligandId=3559 (https://www.genenames.org/data/gene‐symbol‐report/#!/hgnc_id/HGNC:4164, http://www.uniprot.org/uniprot/P01350), http://www.guidetopharmacology.org/GRAC/LigandDisplayForward?ligandId=3725 > http://www.guidetopharmacology.org/GRAC/LigandDisplayForward?ligandId=861, https://www.genenames.org/data/gene‐symbol‐report/#!/hgnc_id/HGNC:1569)	http://www.guidetopharmacology.org/GRAC/LigandDisplayForward?ligandId=864 (https://www.genenames.org/data/gene‐symbol‐report/#!/hgnc_id/HGNC:1569, http://www.uniprot.org/uniprot/P06307), http://www.guidetopharmacology.org/GRAC/LigandDisplayForward?ligandId=10221), http://www.guidetopharmacology.org/GRAC/LigandDisplayForward?ligandId=860, https://www.genenames.org/data/gene‐symbol‐report/#!/hgnc_id/HGNC:1569), http://www.guidetopharmacology.org/GRAC/LigandDisplayForward?ligandId=3552, https://www.genenames.org/data/gene‐symbol‐report/#!/hgnc_id/HGNC:1569) ≥ http://www.guidetopharmacology.org/GRAC/LigandDisplayForward?ligandId=3559 (https://www.genenames.org/data/gene‐symbol‐report/#!/hgnc_id/HGNC:4164, http://www.uniprot.org/uniprot/P01350), http://www.guidetopharmacology.org/GRAC/LigandDisplayForward?ligandId=3725, http://www.guidetopharmacology.org/GRAC/LigandDisplayForward?ligandId=861, https://www.genenames.org/data/gene‐symbol‐report/#!/hgnc_id/HGNC:1569)
Endogenous agonists	http://www.guidetopharmacology.org/GRAC/LigandDisplayForward?ligandId=860 (https://www.genenames.org/data/gene‐symbol‐report/#!/hgnc_id/HGNC:1569, http://www.uniprot.org/uniprot/P06307), http://www.guidetopharmacology.org/GRAC/LigandDisplayForward?ligandId=10221), http://www.guidetopharmacology.org/GRAC/LigandDisplayForward?ligandId=3552, https://www.genenames.org/data/gene‐symbol‐report/#!/hgnc_id/HGNC:1569), http://www.guidetopharmacology.org/GRAC/LigandDisplayForward?ligandId=864, https://www.genenames.org/data/gene‐symbol‐report/#!/hgnc_id/HGNC:1569)	http://www.guidetopharmacology.org/GRAC/LigandDisplayForward?ligandId=3725 [http://www.ncbi.nlm.nih.gov/pubmed/7681836?dopt=AbstractPlus], http://www.guidetopharmacology.org/GRAC/LigandDisplayForward?ligandId=3559 (https://www.genenames.org/data/gene‐symbol‐report/#!/hgnc_id/HGNC:4164, http://www.uniprot.org/uniprot/P01350) [http://www.ncbi.nlm.nih.gov/pubmed/1975695?dopt=AbstractPlus] – Mouse, http://www.guidetopharmacology.org/GRAC/LigandDisplayForward?ligandId=861 (https://www.genenames.org/data/gene‐symbol‐report/#!/hgnc_id/HGNC:1569, http://www.uniprot.org/uniprot/P06307) [http://www.ncbi.nlm.nih.gov/pubmed/8349705?dopt=AbstractPlus], http://www.guidetopharmacology.org/GRAC/LigandDisplayForward?ligandId=3558, https://www.genenames.org/data/gene‐symbol‐report/#!/hgnc_id/HGNC:4164), http://www.guidetopharmacology.org/GRAC/LigandDisplayForward?ligandId=8408, https://www.genenames.org/data/gene‐symbol‐report/#!/hgnc_id/HGNC:4164), http://www.guidetopharmacology.org/GRAC/LigandDisplayForward?ligandId=8409, https://www.genenames.org/data/gene‐symbol‐report/#!/hgnc_id/HGNC:4164), http://www.guidetopharmacology.org/GRAC/LigandDisplayForward?ligandId=8410, https://www.genenames.org/data/gene‐symbol‐report/#!/hgnc_id/HGNC:4164), http://www.guidetopharmacology.org/GRAC/LigandDisplayForward?ligandId=8411, https://www.genenames.org/data/gene‐symbol‐report/#!/hgnc_id/HGNC:4164), http://www.guidetopharmacology.org/GRAC/LigandDisplayForward?ligandId=3562, https://www.genenames.org/data/gene‐symbol‐report/#!/hgnc_id/HGNC:4164), http://www.guidetopharmacology.org/GRAC/LigandDisplayForward?ligandId=3566, https://www.genenames.org/data/gene‐symbol‐report/#!/hgnc_id/HGNC:4164)
Selective agonists	http://www.guidetopharmacology.org/GRAC/LigandDisplayForward?ligandId=858 [http://www.ncbi.nlm.nih.gov/pubmed/1636779?dopt=AbstractPlus] – Rat, http://www.guidetopharmacology.org/GRAC/LigandDisplayForward?ligandId=3888 [http://www.ncbi.nlm.nih.gov/pubmed/7654246?dopt=AbstractPlus], http://www.guidetopharmacology.org/GRAC/LigandDisplayForward?ligandId=865 [http://www.ncbi.nlm.nih.gov/pubmed/9276016?dopt=AbstractPlus]	http://www.guidetopharmacology.org/GRAC/LigandDisplayForward?ligandId=900 [http://www.ncbi.nlm.nih.gov/pubmed/14698161?dopt=AbstractPlus] – Rat, http://www.guidetopharmacology.org/GRAC/LigandDisplayForward?ligandId=3948 [http://www.ncbi.nlm.nih.gov/pubmed/8720482?dopt=AbstractPlus] – Rat
Antagonists	http://www.guidetopharmacology.org/GRAC/LigandDisplayForward?ligandId=890 (pIC_50_ 8.3) [http://www.ncbi.nlm.nih.gov/pubmed/10988332?dopt=AbstractPlus]	–
Selective antagonists	http://www.guidetopharmacology.org/GRAC/LigandDisplayForward?ligandId=878 (pIC_50_ 9.7) [http://www.ncbi.nlm.nih.gov/pubmed/1975695?dopt=AbstractPlus] – Rat, http://www.guidetopharmacology.org/GRAC/LigandDisplayForward?ligandId=884 (pIC_50_ 9.6) [http://www.ncbi.nlm.nih.gov/pubmed/8813597?dopt=AbstractPlus] – Rat, http://www.guidetopharmacology.org/GRAC/LigandDisplayForward?ligandId=904 (pIC_50_ 8.6) [http://www.ncbi.nlm.nih.gov/pubmed/8605955?dopt=AbstractPlus] – Rat, http://www.guidetopharmacology.org/GRAC/LigandDisplayForward?ligandId=891 (pIC_50_ 6.7–8.2) [http://www.ncbi.nlm.nih.gov/pubmed/1975695?dopt=AbstractPlus, http://www.ncbi.nlm.nih.gov/pubmed/2437574?dopt=AbstractPlus] – Rat	http://www.guidetopharmacology.org/GRAC/LigandDisplayForward?ligandId=887 (pIC_50_ 9.7) [http://www.ncbi.nlm.nih.gov/pubmed/22607579?dopt=AbstractPlus, http://www.ncbi.nlm.nih.gov/pubmed/9042983?dopt=AbstractPlus], http://www.guidetopharmacology.org/GRAC/LigandDisplayForward?ligandId=3503 (pIC_50_ 9.4) [http://www.ncbi.nlm.nih.gov/pubmed/11020274?dopt=AbstractPlus], http://www.guidetopharmacology.org/GRAC/LigandDisplayForward?ligandId=881 (pIC_50_ 9.2) [http://www.ncbi.nlm.nih.gov/pubmed/11738246?dopt=AbstractPlus], http://www.guidetopharmacology.org/GRAC/LigandDisplayForward?ligandId=888 (pIC_50_ 9.2) [http://www.ncbi.nlm.nih.gov/pubmed/11738246?dopt=AbstractPlus], http://www.guidetopharmacology.org/GRAC/LigandDisplayForward?ligandId=6665 (pIC_50_ 8.5) [http://www.ncbi.nlm.nih.gov/pubmed/21493750?dopt=AbstractPlus], http://www.guidetopharmacology.org/GRAC/LigandDisplayForward?ligandId=879 (pIC_50_ 8.4) [http://www.ncbi.nlm.nih.gov/pubmed/7681836?dopt=AbstractPlus], http://www.guidetopharmacology.org/GRAC/LigandDisplayForward?ligandId=3523 (pIC_50_ 8) [http://www.ncbi.nlm.nih.gov/pubmed/10385255?dopt=AbstractPlus] – Rat, http://www.guidetopharmacology.org/GRAC/LigandDisplayForward?ligandId=3509 (pIC_50_ 7.5) [http://www.ncbi.nlm.nih.gov/pubmed/21228869?dopt=AbstractPlus] – Rat
Labelled ligands	[http://www.guidetopharmacology.org/GRAC/LigandDisplayForward?ligandId=3476 (Antagonist) (p*K*_d_ 9.7) [http://www.ncbi.nlm.nih.gov/pubmed/3018478?dopt=AbstractPlus], [http://www.guidetopharmacology.org/GRAC/LigandDisplayForward?ligandId=6666 (Agonist) [http://www.ncbi.nlm.nih.gov/pubmed/3410633?dopt=AbstractPlus]	[http://www.guidetopharmacology.org/GRAC/LigandDisplayForward?ligandId=3478 (Antagonist) (p*K*_i_ 9.7–10) [http://www.ncbi.nlm.nih.gov/pubmed/8474432?dopt=AbstractPlus] – Guinea pig, [http://www.guidetopharmacology.org/GRAC/LigandDisplayForward?ligandId=3472 (Antagonist) (p*K*_d_ 9.6) [0905] – Guinea pig, [http://www.guidetopharmacology.org/GRAC/LigandDisplayForward?ligandId=6666 (Agonist) [http://www.ncbi.nlm.nih.gov/pubmed/3410633?dopt=AbstractPlus], [http://www.guidetopharmacology.org/GRAC/LigandDisplayForward?ligandId=3781 (Agonist), [http://www.guidetopharmacology.org/GRAC/LigandDisplayForward?ligandId=3821 (Agonist), [http://www.guidetopharmacology.org/GRAC/LigandDisplayForward?ligandId=3477 (Antagonist) (p*K*_d_ 8.2–8.5) [http://www.ncbi.nlm.nih.gov/pubmed/11738246?dopt=AbstractPlus], [http://www.guidetopharmacology.org/GRAC/LigandDisplayForward?ligandId=6667 (Antagonist) (p*K*_i_ 8.4) [http://www.ncbi.nlm.nih.gov/pubmed/19271701?dopt=AbstractPlus]

### Comments

While a cancer‐specific CCK receptor has been postulated to exist, which also might be responsive to incompletely processed forms of CCK (Gly‐extended forms), this has never been isolated. An alternatively spliced form of the CCK_2_ receptor in which intron 4 is retained, adding 69 amino acids to the intracellular loop 3 (ICL3) region, has been described to be present particularly in certain neoplasms where mRNA mis‐splicing has been commonly observed [http://www.ncbi.nlm.nih.gov/pubmed/12429993?dopt=AbstractPlus], but it is not clear that this receptor splice form plays a special role in carcinogenesis. Another alternative splicing event for the CCK_2_ receptor was reported [http://www.ncbi.nlm.nih.gov/pubmed/8415658?dopt=AbstractPlus], with alternative donor sites in exon 4 resulting in long (452 amino acids) and short (447 amino acids) forms of the receptor differing by five residues in ICL3, however, no clear functional differences have been observed.

### Further reading on Cholecystokinin receptors

Ballaz S. (2017) The unappreciated roles of the cholecystokinin receptor CCK(1) in brain functioning. *Rev Neurosci* **28**: 573‐585 [https://www.ncbi.nlm.nih.gov/pubmed/28343167?dopt=AbstractPlus]

Dockray GJ. (2009) Cholecystokinin and gut‐brain signalling. *Regul. Pept.* **155**: 6‐10 [https://www.ncbi.nlm.nih.gov/pubmed/19345244?dopt=AbstractPlus]

Cawston EE *et al*. (2010) Therapeutic potential for novel drugs targeting the type 1 cholecystokinin receptor. *Br. J. Pharmacol.* **159**: 1009‐21 [https://www.ncbi.nlm.nih.gov/pubmed/19922535?dopt=AbstractPlus]

Dufresne M *et al*. (2006) Cholecystokinin and gastrin receptors. *Physiol. Rev.* **86**: 805‐47 [https://www.ncbi.nlm.nih.gov/pubmed/16816139?dopt=AbstractPlus]

## http://www.guidetopharmacology.org/GRAC/FamilyDisplayForward?familyId=25

### Overview

Receptors of the Class Frizzled (FZD, **nomenclature as agreed by the NC‐IUPHAR subcommittee on the Class Frizzled GPCRs** [http://www.ncbi.nlm.nih.gov/pubmed/21079039?dopt=AbstractPlus]), are GPCRs originally identified in *Drosophila* [http://www.ncbi.nlm.nih.gov/pubmed/1334084?dopt=AbstractPlus], which are highly conserved across species. While SMO shows structural resemblance to the 10 FZDs, it is functionally separated as it mediates effects in the Hedgehog signaling pathway [http://www.ncbi.nlm.nih.gov/pubmed/21079039?dopt=AbstractPlus]. FZDs are activated by WNTs, which are cysteine‐rich lipoglycoproteins withfundamentalfunctions inontogeny and tissue homeostasis. FZD signalling was initially divided into two pathways, being either dependent on the accumulation of the transcription regulator http://www.guidetopharmacology.org/GRAC/LigandDisplayForward?ligandId=5371 (https://www.genenames.org/data/gene‐symbol‐report/#!/hgnc_id/HGNC:2514, http://www.uniprot.org/uniprot/P35222) or being β‐catenin‐independent (often referred to as canonical *vs.* non‐canonical WNT/FZD signalling, respectively). WNT stimulation of FZDs can, in cooperation with the low density lipoprotein receptors https://www.genenames.org/data/gene‐symbol‐report/#!/hgnc_id/HGNC:6697 (http://www.uniprot.org/uniprot/O75197) and https://www.genenames.org/data/gene‐symbol‐report/#!/hgnc_id/HGNC:6698 (http://www.uniprot.org/uniprot/O75581), lead to the inhibition of a constitutively active destruction complex, which results in the accumulation of β‐catenin and subsequently its translocation to the nucleus. β‐Catenin, in turn, modifies gene transcription by interacting with TCF/LEF transcription factors. βCatenin‐independent FZD signalling is far more complex with regard to the diversity of the activated pathways. WNT/FZD signalling can lead to the activation of heterotrimeric G proteins [http://www.ncbi.nlm.nih.gov/pubmed/24032637?dopt=AbstractPlus, http://www.ncbi.nlm.nih.gov/pubmed/28790300?dopt=AbstractPlus, http://www.ncbi.nlm.nih.gov/pubmed/30049420?dopt=AbstractPlus], the elevation of intracellular calcium [http://www.ncbi.nlm.nih.gov/pubmed/9389482?dopt=AbstractPlus], activation of cGMP‐specific PDE6 [http://www.ncbi.nlm.nih.gov/pubmed/12471263?dopt=AbstractPlus] and elevation of cAMP as well as RAC‐1, JNK, Rho and Rho kinase signalling [http://www.ncbi.nlm.nih.gov/pubmed/19651774?dopt=AbstractPlus]. Novel resonance energy transfer‐based tools have allowed the study of the GPCR‐like nature of FZDs in greater detail. Upon ligand stimulation, FZDs undergo conformational changes and signal *via* heterotrimeric G proteins [http://www.ncbi.nlm.nih.gov/pubmed/30514810?dopt=AbstractPlus, http://www.ncbi.nlm.nih.gov/pubmed/30737406?dopt=AbstractPlus]. Furthermore, the phosphoprotein Dishevelled constitutes a key player in WNT/FZD signalling. Importantly, FZDs exist in at least two distinct conformational states that regulate the pathway selection [http://www.ncbi.nlm.nih.gov/pubmed/30737406?dopt=AbstractPlus]. As with other GPCRs, members of the Frizzled family are functionally dependent on the arrestin scaffolding protein for internalization [http://www.ncbi.nlm.nih.gov/pubmed/12958365?dopt=AbstractPlus], as well as for β‐catenin‐dependent [http://www.ncbi.nlm.nih.gov/pubmed/17426148?dopt=AbstractPlus] and ‐independent [http://www.ncbi.nlm.nih.gov/pubmed/18953287?dopt=AbstractPlus, http://www.ncbi.nlm.nih.gov/pubmed/17476309?dopt=AbstractPlus] signalling. The pattern of cell signalling is complicated by the presence of additional ligands, which can enhance or inhibit FZD signalling (secreted Frizzled‐related proteins (sFRP), http://www.guidetopharmacology.org/GRAC/LigandDisplayForward?ligandId=5372 (https://www.genenames.org/data/gene‐symbol‐report/#!/hgnc_id/HGNC:18081, http://www.uniprot.org/uniprot/Q9Y5W5) (WIF), http://www.guidetopharmacology.org/GRAC/LigandDisplayForward?ligandId=3704 (https://www.genenames.org/data/gene‐symbol‐report/#!/hgnc_id/HGNC:13771, http://www.uniprot.org/uniprot/Q9BQB4) or Dickkopf (DKK)), as well as modulatory (co)‐receptors with http://www.guidetopharmacology.org/GRAC/FamilyDisplayForward?familyId=304#Type XV RTKs: RYK, http://www.guidetopharmacology.org/GRAC/FamilyDisplayForward?familyId=304#Type VIII RTKs: ROR1, http://www.guidetopharmacology.org/GRAC/FamilyDisplayForward?familyId=304#Type VIII RTKs: ROR2 and Kremen, which may also function as independent signalling proteins.

**Table:** 

Nomenclature	http://www.guidetopharmacology.org/GRAC/ObjectDisplayForward?objectId=229	http://www.guidetopharmacology.org/GRAC/ObjectDisplayForward?objectId=230	http://www.guidetopharmacology.org/GRAC/ObjectDisplayForward?objectId=231	http://www.guidetopharmacology.org/GRAC/ObjectDisplayForward?objectId=232	http://www.guidetopharmacology.org/GRAC/ObjectDisplayForward?objectId=233
HGNC, UniProt	https://www.genenames.org/data/gene‐symbol‐report/#!/hgnc_id/HGNC:4038, http://www.uniprot.org/uniprot/Q9UP38	https://www.genenames.org/data/gene‐symbol‐report/#!/hgnc_id/HGNC:4040, http://www.uniprot.org/uniprot/Q14332	https://www.genenames.org/data/gene‐symbol‐report/#!/hgnc_id/HGNC:4041, http://www.uniprot.org/uniprot/Q9NPG1	https://www.genenames.org/data/gene‐symbol‐report/#!/hgnc_id/HGNC:4042, http://www.uniprot.org/uniprot/Q9ULV1	https://www.genenames.org/data/gene‐symbol‐report/#!/hgnc_id/HGNC:4043, http://www.uniprot.org/uniprot/Q13467
Allosteric modulators	–	–	–	http://www.guidetopharmacology.org/GRAC/LigandDisplayForward?ligandId=10321 (Negative) (pIC_50_ 5.5–7.8) [http://www.ncbi.nlm.nih.gov/pubmed/25751279?dopt=AbstractPlus], http://www.guidetopharmacology.org/GRAC/LigandDisplayForward?ligandId=10321 (Positive) (pEC_50_ 6.4) [http://www.ncbi.nlm.nih.gov/pubmed/29293331?dopt=AbstractPlus], http://www.guidetopharmacology.org/GRAC/LigandDisplayForward?ligandId=10322 (Negative) (pIC_50_ 6.2) [http://www.ncbi.nlm.nih.gov/pubmed/25751279?dopt=AbstractPlus, http://www.ncbi.nlm.nih.gov/pubmed/25751279?dopt=AbstractPlus]	–
Antibodies	http://www.guidetopharmacology.org/GRAC/LigandDisplayForward?ligandId=10197 (Antagonist) (pIC_50_ ∼9.1) [http://www.ncbi.nlm.nih.gov/pubmed/22753465?dopt=AbstractPlus]	http://www.guidetopharmacology.org/GRAC/LigandDisplayForward?ligandId=10197 (Antagonist) (pIC_50_ ∼9) [http://www.ncbi.nlm.nih.gov/pubmed/22753465?dopt=AbstractPlus]	–	–	http://www.guidetopharmacology.org/GRAC/LigandDisplayForward?ligandId=10197 (Antagonist) (pIC_50_ ∼9) [http://www.ncbi.nlm.nih.gov/pubmed/22753465?dopt=AbstractPlus]
Comments	–	–	–	–	IgG‐2919 and IgG‐2921 are FZD5 antibodies that have exhibited antitumour activities *in vitro* and *in vivo* (inhibiting the growth of RNF43‐mutant pancreatic ductal adenocarcinoma cells/xenograft tumours), by blocking autocrine Wnt‐β‐catenin signalling in these mutant, FZD5‐dependent cells [http://www.ncbi.nlm.nih.gov/pubmed/27869803?dopt=AbstractPlus].

**Table:** 

Nomenclature	http://www.guidetopharmacology.org/GRAC/ObjectDisplayForward?objectId=234	http://www.guidetopharmacology.org/GRAC/ObjectDisplayForward?objectId=235	http://www.guidetopharmacology.org/GRAC/ObjectDisplayForward?objectId=236	http://www.guidetopharmacology.org/GRAC/ObjectDisplayForward?objectId=237	http://www.guidetopharmacology.org/GRAC/ObjectDisplayForward?objectId=238
HGNC, UniProt	https://www.genenames.org/data/gene‐symbol‐report/#!/hgnc_id/HGNC:4044, http://www.uniprot.org/uniprot/O60353	https://www.genenames.org/data/gene‐symbol‐report/#!/hgnc_id/HGNC:4045, http://www.uniprot.org/uniprot/O75084	https://www.genenames.org/data/gene‐symbol‐report/#!/hgnc_id/HGNC:4046, http://www.uniprot.org/uniprot/Q9H461	https://www.genenames.org/data/gene‐symbol‐report/#!/hgnc_id/HGNC:4047, http://www.uniprot.org/uniprot/O00144	https://www.genenames.org/data/gene‐symbol‐report/#!/hgnc_id/HGNC:4039, http://www.uniprot.org/uniprot/Q9ULW2
Selective antagonists	–	http://www.guidetopharmacology.org/GRAC/LigandDisplayForward?ligandId=10324 (pIC_50_ 7) [http://www.ncbi.nlm.nih.gov/pubmed/29632413?dopt=AbstractPlus]	–	–	–
Antibodies	–	http://www.guidetopharmacology.org/GRAC/LigandDisplayForward?ligandId=10197 (Antagonist) (pIC_50_ ∼9) [http://www.ncbi.nlm.nih.gov/pubmed/22753465?dopt=AbstractPlus]	http://www.guidetopharmacology.org/GRAC/LigandDisplayForward?ligandId=10197 (Antagonist) (pIC_50_ ∼8) [http://www.ncbi.nlm.nih.gov/pubmed/22753465?dopt=AbstractPlus]	–	–
Comments	–	–	FZD8‐Fc/OMP‐54F28 is a FZD8 antagonist [http://www.ncbi.nlm.nih.gov/pubmed/17545618?dopt=AbstractPlus].	–	Radio‐labelled murine monoclonal antibody MAb 92‐13 has been used to demonstrate the therapeutic potential of targeting FZD10‐positive tumours [http://www.ncbi.nlm.nih.gov/pubmed/18271942?dopt=AbstractPlus].

**Table:** 

Nomenclature	http://www.guidetopharmacology.org/GRAC/ObjectDisplayForward?objectId=239
HGNC, UniProt	https://www.genenames.org/data/gene‐symbol‐report/#!/hgnc_id/HGNC:11119, http://www.uniprot.org/uniprot/Q99835
Agonists	http://www.guidetopharmacology.org/GRAC/LigandDisplayForward?ligandId=10327) [http://www.ncbi.nlm.nih.gov/pubmed/12391318?dopt=AbstractPlus] – Mouse, http://www.guidetopharmacology.org/GRAC/LigandDisplayForward?ligandId=10356 [http://www.ncbi.nlm.nih.gov/pubmed/16408088?dopt=AbstractPlus]
Antagonists	http://www.guidetopharmacology.org/GRAC/LigandDisplayForward?ligandId=10332 (p*K*_d_ 9.5) [http://www.ncbi.nlm.nih.gov/pubmed/25636740?dopt=AbstractPlus], http://www.guidetopharmacology.org/GRAC/LigandDisplayForward?ligandId=10330 (p*K*_i_ 7.7) [http://www.ncbi.nlm.nih.gov/pubmed/12391318?dopt=AbstractPlus] – Mouse, http://www.guidetopharmacology.org/GRAC/LigandDisplayForward?ligandId=10329 (pIC_50_ 7.7) [http://www.ncbi.nlm.nih.gov/pubmed/10984056?dopt=AbstractPlus] – Mouse, http://www.guidetopharmacology.org/GRAC/LigandDisplayForward?ligandId=10328 (pIC_50_ ∼7) [http://www.ncbi.nlm.nih.gov/pubmed/27338657?dopt=AbstractPlus] – Mouse
Selective antagonists	http://www.guidetopharmacology.org/GRAC/LigandDisplayForward?ligandId=6975 (p*K*_i_ 7.8) [http://www.ncbi.nlm.nih.gov/pubmed/23063522?dopt=AbstractPlus]
Allosteric modulators	http://www.guidetopharmacology.org/GRAC/LigandDisplayForward?ligandId=10326 (Positive) (pEC_50_ 5.9) [http://www.ncbi.nlm.nih.gov/pubmed/23448715?dopt=AbstractPlus]
Comments	SANT‐3 and SANT‐4 are SMO antagonists [http://www.ncbi.nlm.nih.gov/pubmed/12391318?dopt=AbstractPlus].

### Comments

There is limited knowledge about WNT/FZD specificity and which molecular entities determine the signalling outcome of a specific WNT/FZD pair. Understanding of theFZD and SMO coupling to G proteins is incomplete, but progress have been made [http://www.ncbi.nlm.nih.gov/pubmed/27458145?dopt=AbstractPlus, http://www.ncbi.nlm.nih.gov/pubmed/24032637?dopt=AbstractPlus, http://www.ncbi.nlm.nih.gov/pubmed/24873871?dopt=AbstractPlus, http://www.ncbi.nlm.nih.gov/pubmed/26179037?dopt=AbstractPlus, http://www.ncbi.nlm.nih.gov/pubmed/16885213?dopt=AbstractPlus, http://www.ncbi.nlm.nih.gov/pubmed/23292797?dopt=AbstractPlus, http://www.ncbi.nlm.nih.gov/pubmed/22179044?dopt=AbstractPlus, http://www.ncbi.nlm.nih.gov/pubmed/30514810?dopt=AbstractPlus]. There is also a scarcity of information on basic pharmacological characteristics of FZDs, such as binding constants, ligand specificity or concentration‐response relationships [http://www.ncbi.nlm.nih.gov/pubmed/19208479?dopt=AbstractPlus]. Development of pharmacological tools for SMO has been faciliated by successful crystalization of several SMO structures [http://www.ncbi.nlm.nih.gov/pubmed/27437577?dopt=AbstractPlus, http://www.ncbi.nlm.nih.gov/pubmed/29804838?dopt=AbstractPlus, http://www.ncbi.nlm.nih.gov/pubmed/25008467?dopt=AbstractPlus, http://www.ncbi.nlm.nih.gov/pubmed/23636324?dopt=AbstractPlus, http://www.ncbi.nlm.nih.gov/pubmed/24525480?dopt=AbstractPlus, http://www.ncbi.nlm.nih.gov/pubmed/28513578?dopt=AbstractPlus]. The recently solved FZD4 in apo state has provided first insight into FZD transmembranous organization [http://www.ncbi.nlm.nih.gov/pubmed/30135577?dopt=AbstractPlus].

### Ligands associated with FZD signalling

***WNTs***: http://www.guidetopharmacology.org/GRAC/LigandDisplayForward?ligandId=3672 (https://www.genenames.org/data/gene‐symbol‐report/#!/hgnc_id/HGNC:12774, http://www.uniprot.org/uniprot/P04628), http://www.guidetopharmacology.org/GRAC/LigandDisplayForward?ligandId=3673 (https://www.genenames.org/data/gene‐symbol‐report/#!/hgnc_id/HGNC:12780, http://www.uniprot.org/uniprot/P09544) (also known as Int‐1‐related protein), http://www.guidetopharmacology.org/GRAC/LigandDisplayForward?ligandId=3674 (https://www.genenames.org/data/gene‐symbol‐report/#!/hgnc_id/HGNC:12781, http://www.uniprot.org/uniprot/Q93097) (also known as WNT‐13), http://www.guidetopharmacology.org/GRAC/LigandDisplayForward?ligandId=3675 (https://www.genenames.org/data/gene‐symbol‐report/#!/hgnc_id/HGNC:12782, http://www.uniprot.org/uniprot/P56703), http://www.guidetopharmacology.org/GRAC/LigandDisplayForward?ligandId=3549 (https://www.genenames.org/data/gene‐symbol‐report/#!/hgnc_id/HGNC:15983, http://www.uniprot.org/uniprot/P56704), http://www.guidetopharmacology.org/GRAC/LigandDisplayForward?ligandId=3547 (https://www.genenames.org/data/gene‐symbol‐report/#!/hgnc_id/HGNC:12783, http://www.uniprot.org/uniprot/P56705), http://www.guidetopharmacology.org/GRAC/LigandDisplayForward?ligandId=3548 (https://www.genenames.org/data/gene‐symbol‐report/#!/hgnc_id/HGNC:12784, http://www.uniprot.org/uniprot/P41221) (pEC50 7.7‐8.9 [http://www.ncbi.nlm.nih.gov/pubmed/30514810?dopt=AbstractPlus]), http://www.guidetopharmacology.org/GRAC/LigandDisplayForward?ligandId=3676 (https://www.genenames.org/data/gene‐symbol‐report/#!/hgnc_id/HGNC:16265, http://www.uniprot.org/uniprot/Q9H1J7), http://www.guidetopharmacology.org/GRAC/LigandDisplayForward?ligandId=3678 (https://www.genenames.org/data/gene‐symbol‐report/#!/hgnc_id/HGNC:12785, http://www.uniprot.org/uniprot/Q9Y6F9), http://www.guidetopharmacology.org/GRAC/LigandDisplayForward?ligandId=3679 (https://www.genenames.org/data/gene‐symbol‐report/#!/hgnc_id/HGNC:12786, http://www.uniprot.org/uniprot/O00755), http://www.guidetopharmacology.org/GRAC/LigandDisplayForward?ligandId=3681 (https://www.genenames.org/data/gene‐symbol‐report/#!/hgnc_id/HGNC:12787, http://www.uniprot.org/uniprot/P56706), http://www.guidetopharmacology.org/GRAC/LigandDisplayForward?ligandId=3682 (https://www.genenames.org/data/gene‐symbol‐report/#!/hgnc_id/HGNC:12788, http://www.uniprot.org/uniprot/Q9H1J5), http://www.guidetopharmacology.org/GRAC/LigandDisplayForward?ligandId=3683 (https://www.genenames.org/data/gene‐symbol‐report/#!/hgnc_id/HGNC:12789, http://www.uniprot.org/uniprot/Q93098), http://www.guidetopharmacology.org/GRAC/LigandDisplayForward?ligandId=3684 (https://www.genenames.org/data/gene‐symbol‐report/#!/hgnc_id/HGNC:12778, http://www.uniprot.org/uniprot/O14904) (also known as WNT‐14), http://www.guidetopharmacology.org/GRAC/LigandDisplayForward?ligandId=3686 (https://www.genenames.org/data/gene‐symbol‐report/#!/hgnc_id/HGNC:12779, http://www.uniprot.org/uniprot/O14905) (also known as WNT‐15 or WNT14b), http://www.guidetopharmacology.org/GRAC/LigandDisplayForward?ligandId=3687(https://www.genenames.org/data/gene‐symbol‐report/#!/hgnc_id/HGNC:13829, http://www.uniprot.org/uniprot/Q9GZT5), http://www.guidetopharmacology.org/GRAC/LigandDisplayForward?ligandId=3688 (https://www.genenames.org/data/gene‐symbol‐report/#!/hgnc_id/HGNC:12775, http://www.uniprot.org/uniprot/O00744) (also known as WNT‐12), http://www.guidetopharmacology.org/GRAC/LigandDisplayForward?ligandId=3689 (https://www.genenames.org/data/gene‐symbol‐report/#!/hgnc_id/HGNC:12776, http://www.uniprot.org/uniprot/O96014) and http://www.guidetopharmacology.org/GRAC/LigandDisplayForward?ligandId=3690 (https://www.genenames.org/data/gene‐symbol‐report/#!/hgnc_id/HGNC:16267, http://www.uniprot.org/uniprot/Q9UBV4).

***Extracellular proteins that interact with FZDs*:** http://www.guidetopharmacology.org/GRAC/LigandDisplayForward?ligandId=1063 (https://www.genenames.org/data/gene‐symbol‐report/#!/hgnc_id/HGNC:7678, http://www.uniprot.org/uniprot/Q00604), http://www.guidetopharmacology.org/GRAC/LigandDisplayForward?ligandId=3700 (https://www.genenames.org/data/gene‐symbol‐report/#!/hgnc_id/HGNC:16175, http://www.uniprot.org/uniprot/Q2I0M5), http://www.guidetopharmacology.org/GRAC/LigandDisplayForward?ligandId=3691 (https://www.genenames.org/data/gene‐symbol‐report/#!/hgnc_id/HGNC:10776, http://www.uniprot.org/uniprot/Q8N474), http://www.guidetopharmacology.org/GRAC/LigandDisplayForward?ligandId=3692 (https://www.genenames.org/data/gene‐symbol‐report/#!/hgnc_id/HGNC:10777, http://www.uniprot.org/uniprot/Q96HF1), http://www.guidetopharmacology.org/GRAC/LigandDisplayForward?ligandId=3693 (https://www.genenames.org/data/gene‐symbol‐report/#!/hgnc_id/HGNC:3959,http://www.uniprot.org/uniprot/Q92765), http://www.guidetopharmacology.org/GRAC/LigandDisplayForward?ligandId=3694 (https://www.genenames.org/data/gene‐symbol‐report/#!/hgnc_id/HGNC:10778, http://www.uniprot.org/uniprot/Q6FHJ7), http://www.guidetopharmacology.org/GRAC/LigandDisplayForward?ligandId=3695 (https://www.genenames.org/data/gene‐symbol‐report/#!/hgnc_id/HGNC:10779, https://www.genenames.org/data/gene‐symbol‐report/#!/hgnc_id/HGNC:10779).

***Extracellular proteins that interact with WNTs or LRPs:*** http://www.guidetopharmacology.org/GRAC/LigandDisplayForward?ligandId=3701 (https://www.genenames.org/data/gene‐symbol‐report/#!/hgnc_id/HGNC:2891, http://www.uniprot.org/uniprot/O94907), https://www.genenames.org/data/gene‐symbol‐report/#!/hgnc_id/HGNC:18081 (http://www.uniprot.org/uniprot/Q9Y5W5), http://www.guidetopharmacology.org/GRAC/LigandDisplayForward?ligandId=3704 (https://www.genenames.org/data/gene‐symbol‐report/#!/hgnc_id/HGNC:13771, http://www.uniprot.org/uniprot/Q9BQB4), http://www.guidetopharmacology.org/GRAC/LigandDisplayForward?ligandId=3702 (https://www.genenames.org/data/gene‐symbol‐report/#!/hgnc_id/HGNC:17550, http://www.uniprot.org/uniprot/Q96MU8) and http://www.guidetopharmacology.org/GRAC/LigandDisplayForward?ligandId=3703 (https://www.genenames.org/data/gene‐symbol‐report/#!/hgnc_id/HGNC:18797, http://www.uniprot.org/uniprot/Q8NCW0)

***Small exogenous ligands:*** Foxy‐5 [http://www.ncbi.nlm.nih.gov/pubmed/18927296?dopt=AbstractPlus], Box‐5 [http://www.ncbi.nlm.nih.gov/pubmed/19901340?dopt=AbstractPlus], UM206 [http://www.ncbi.nlm.nih.gov/pubmed/21931076?dopt=AbstractPlus], and XWnt8 (http://www.uniprot.org/uniprot/P28026) also known as mini‐Wnt8.

**Ligands associated with SMO signalling:** http://www.guidetopharmacology.org/GRAC/LigandDisplayForward?ligandId=2718, oxysterols [http://www.ncbi.nlm.nih.gov/pubmed/27437577?dopt=AbstractPlus, http://www.ncbi.nlm.nih.gov/pubmed/27705744?dopt=AbstractPlus, http://www.ncbi.nlm.nih.gov/pubmed/30340023?dopt=AbstractPlus].

### Further reading on Class Frizzled GPCRs

Angers S *et al*. (2009) Proximal events in Wnt signal transduction. *Nat. Rev. Mol. Cell Biol.* **10**: [https://www.ncbi.nlm.nih.gov/pubmed/22935904?dopt=AbstractPlus]

van Amerongen R. (2012) Alternative Wnt pathways and receptors. *Cold Spring Harb Perspect Biol* **4**: 468‐77 [https://www.ncbi.nlm.nih.gov/pubmed/19536106?dopt=AbstractPlus]

Schulte G.(2015)Frizzleds and WNT/β‐cateninsignaling–The black boxof ligand‐receptor selectivity, 113‐39 [https://www.ncbi.nlm.nih.gov/pubmed/26969975?dopt=AbstractPlus]

Wang Y *et al*. (2016) Frizzled Receptors in Development and Disease. *Curr. Top. Dev. Biol.* **117**: complex stoichiometry and activation kinetics. *Eur. J. Pharmacol.* **763**: 191‐5 [https://www.ncbi.nlm.nih.gov/pubmed/26003275?dopt=AbstractPlus]

Schulte G *et al*. (2018) Frizzleds as GPCRs ‐ More Conventional Than We Thought! *Trends Pharmacol. Sci.* **39**: 828‐842 [https://www.ncbi.nlm.nih.gov/pubmed/30049420?dopt=AbstractPlus]

## http://www.guidetopharmacology.org/GRAC/FamilyDisplayForward?familyId=5

### Overview

Complement peptide receptors (**nomenclature as agreed by the NC‐IUPHAR subcommittee on Complement peptide receptors** [http://www.ncbi.nlm.nih.gov/pubmed/23383423?dopt=AbstractPlus]) are activated by the endogenous 75 amino‐acid anaphylatoxin polypeptides http://www.guidetopharmacology.org/GRAC/LigandDisplayForward?ligandId=3640 (https://www.genenames.org/data/gene‐symbol‐report/#!/hgnc_id/HGNC:1318, http://www.uniprot.org/uniprot/P01024) and http://www.guidetopharmacology.org/GRAC/LigandDisplayForward?ligandId=573 (https://www.genenames.org/data/gene‐symbol‐report/#!/hgnc_id/HGNC:1331, http://www.uniprot.org/uniprot/P01031), generated upon stimulation of the complement cascade. C3a and C5a exert their functions through binding to their receptors (C3aR and C5aR), causing cell activation and triggering cellular degranulation that contributes to the local inflammation.

**Table:** 

Nomenclature	http://www.guidetopharmacology.org/GRAC/ObjectDisplayForward?objectId=31	http://www.guidetopharmacology.org/GRAC/ObjectDisplayForward?objectId=32	http://www.guidetopharmacology.org/GRAC/ObjectDisplayForward?objectId=33
HGNC, UniProt	https://www.genenames.org/data/gene‐symbol‐report/#!/hgnc_id/HGNC:1319, http://www.uniprot.org/uniprot/Q16581	https://www.genenames.org/data/gene‐symbol‐report/#!/hgnc_id/HGNC:1338, http://www.uniprot.org/uniprot/P21730	https://www.genenames.org/data/gene‐symbol‐report/#!/hgnc_id/HGNC:4527, http://www.uniprot.org/uniprot/Q9P296
Potency order of endogenous ligands	http://www.guidetopharmacology.org/GRAC/LigandDisplayForward?ligandId=3640 (https://www.genenames.org/data/gene‐symbol‐report/#!/hgnc_id/HGNC:1318, http://www.uniprot.org/uniprot/P01024) > http://www.guidetopharmacology.org/GRAC/LigandDisplayForward?ligandId=573 (https://www.genenames.org/data/gene‐symbol‐report/#!/hgnc_id/HGNC:1331, http://www.uniprot.org/uniprot/P01031) [http://www.ncbi.nlm.nih.gov/pubmed/8898085?dopt=AbstractPlus]	http://www.guidetopharmacology.org/GRAC/LigandDisplayForward?ligandId=573 (https://www.genenames.org/data/gene‐symbol‐report/#!/hgnc_id/HGNC:1331, http://www.uniprot.org/uniprot/P01031), http://www.guidetopharmacology.org/GRAC/LigandDisplayForward?ligandId=574) > http://www.guidetopharmacology.org/GRAC/LigandDisplayForward?ligandId=3640 (https://www.genenames.org/data/gene‐symbol‐report/#!/hgnc_id/HGNC:1318, http://www.uniprot.org/uniprot/P01024) [http://www.ncbi.nlm.nih.gov/pubmed/8898085?dopt=AbstractPlus]	–
Endogenous agonists	–	http://www.guidetopharmacology.org/GRAC/LigandDisplayForward?ligandId=3728 (https://www.genenames.org/data/gene‐symbol‐report/#!/hgnc_id/HGNC:10402, http://www.uniprot.org/uniprot/P39019) [http://www.ncbi.nlm.nih.gov/pubmed/11107061?dopt=AbstractPlus]	–
Agonists	http://www.guidetopharmacology.org/GRAC/LigandDisplayForward?ligandId=9449 [http://www.ncbi.nlm.nih.gov/pubmed/24257095?dopt=AbstractPlus], http://www.guidetopharmacology.org/GRAC/LigandDisplayForward?ligandId=8381 [http://www.ncbi.nlm.nih.gov/pubmed/25259874?dopt=AbstractPlus], http://www.guidetopharmacology.org/GRAC/LigandDisplayForward?ligandId=10224 [http://www.ncbi.nlm.nih.gov/pubmed/9145417?dopt=AbstractPlus, http://www.ncbi.nlm.nih.gov/pubmed/26297549?dopt=AbstractPlus], http://www.guidetopharmacology.org/GRAC/LigandDisplayForward?ligandId=10226 2086, http://www.ncbi.nlm.nih.gov/pubmed/26297549?dopt=AbstractPlus], http://www.guidetopharmacology.org/GRAC/LigandDisplayForward?ligandId=10227 [http://www.ncbi.nlm.nih.gov/pubmed/11179594?dopt=AbstractPlus, http://www.ncbi.nlm.nih.gov/pubmed/7804141?dopt=AbstractPlus]	http://www.guidetopharmacology.org/GRAC/LigandDisplayForward?ligandId=9746 (Inverse agonist) [http://www.ncbi.nlm.nih.gov/pubmed/18753409?dopt=AbstractPlus], http://www.guidetopharmacology.org/GRAC/LigandDisplayForward?ligandId=572 [http://www.ncbi.nlm.nih.gov/pubmed/1732540?dopt=AbstractPlus, http://www.ncbi.nlm.nih.gov/pubmed/7930622?dopt=AbstractPlus], http://www.guidetopharmacology.org/GRAC/LigandDisplayForward?ligandId=10228 [2276, http://www.ncbi.nlm.nih.gov/pubmed/26297549?dopt=AbstractPlus]	–
Selective agonists	–	–	http://www.guidetopharmacology.org/GRAC/LigandDisplayForward?ligandId=9385 (Biased agonist) [http://www.ncbi.nlm.nih.gov/pubmed/27108698?dopt=AbstractPlus], http://www.guidetopharmacology.org/GRAC/LigandDisplayForward?ligandId=9384 (Biased agonist) [http://www.ncbi.nlm.nih.gov/pubmed/27108698?dopt=AbstractPlus]
Antagonists	http://www.guidetopharmacology.org/GRAC/LigandDisplayForward?ligandId=3529 (pIC_50_ 7.6) [http://www.ncbi.nlm.nih.gov/pubmed/11342658?dopt=AbstractPlus], http://www.guidetopharmacology.org/GRAC/LigandDisplayForward?ligandId=8384 (pIC_50_ 5.9) [http://www.ncbi.nlm.nih.gov/pubmed/25259874?dopt=AbstractPlus]	http://www.guidetopharmacology.org/GRAC/LigandDisplayForward?ligandId=9450 (pIC_50_ 9.7) [http://www.ncbi.nlm.nih.gov/pubmed/27768695?dopt=AbstractPlus], http://www.guidetopharmacology.org/GRAC/LigandDisplayForward?ligandId=581 (p*K*_i_ 8.7) [http://www.ncbi.nlm.nih.gov/pubmed/12384495?dopt=AbstractPlus], http://www.guidetopharmacology.org/GRAC/LigandDisplayForward?ligandId=9451 (pIC_50_ 8.3) [http://www.ncbi.nlm.nih.gov/pubmed/25385614?dopt=AbstractPlus], http://www.guidetopharmacology.org/GRAC/LigandDisplayForward?ligandId=3853 (pIC_50_ 7.9) [http://www.ncbi.nlm.nih.gov/pubmed/9719594?dopt=AbstractPlus], http://www.guidetopharmacology.org/GRAC/LigandDisplayForward?ligandId=576 (pIC_50_ 7.2) [http://www.ncbi.nlm.nih.gov/pubmed/7930622?dopt=AbstractPlus]	–
Labelled ligands	[http://www.guidetopharmacology.org/GRAC/LigandDisplayForward?ligandId=3773) (Agonist) [http://www.ncbi.nlm.nih.gov/pubmed/10092660?dopt=AbstractPlus]	[http://www.guidetopharmacology.org/GRAC/LigandDisplayForward?ligandId=3774) (Agonist) [http://www.ncbi.nlm.nih.gov/pubmed/4020139?dopt=AbstractPlus]	[http://www.guidetopharmacology.org/GRAC/LigandDisplayForward?ligandId=3774) (Agonist)
Comments	C3a‐C3aR signalling plays a crucial role in inhibiting neural progenitor cell proliferation during neurodevelopment, playing a critical role in the normal development of the mammalian brain [http://www.ncbi.nlm.nih.gov/pubmed/30449309?dopt=AbstractPlus].	–	–

### Comments

http://www.guidetopharmacology.org/GRAC/LigandDisplayForward?ligandId=3529 has also been reported to have agonist properties at the C3a receptor [http://www.ncbi.nlm.nih.gov/pubmed/16154494?dopt=AbstractPlus]. The putative chemoattractant receptor termed C5a_2_ (also known as GPR77, C5L2) binds [125I]C5a with no clear signalling function, but has a putative role opposing inflammatory responses [http://www.ncbi.nlm.nih.gov/pubmed/11773063?dopt=AbstractPlus, http://www.ncbi.nlm.nih.gov/pubmed/15784721?dopt=AbstractPlus, http://www.ncbi.nlm.nih.gov/pubmed/15715664?dopt=AbstractPlus]. Binding to this site may be displaced with the rank order http://www.guidetopharmacology.org/GRAC/LigandDisplayForward?ligandId=574 (https://www.genenames.org/data/gene‐symbol‐report/#!/hgnc_id/HGNC:1331)> http://www.guidetopharmacology.org/GRAC/LigandDisplayForward?ligandId=573 (https://www.genenames.org/data/gene‐symbol‐report/#!/hgnc_id/HGNC:1331, http://www.uniprot.org/uniprot/P01031[http://www.ncbi.nlm.nih.gov/pubmed/11773063?dopt=AbstractPlus, http://www.ncbi.nlm.nih.gov/pubmed/12899627?dopt=AbstractPlus] while there is controversy over the ability of http://www.guidetopharmacology.org/GRAC/LigandDisplayForward?ligandId=3640 (https://www.genenames.org/data/gene‐symbol‐report/#!/hgnc_id/HGNC:1318, http://www.uniprot.org/uniprot/P01024) and http://www.guidetopharmacology.org/GRAC/LigandDisplayForward?ligandId=5367 (https://www.genenames.org/data/gene‐symbol‐report/#!/hgnc_id/HGNC:1318, https://www.genenames.org/data/gene‐symbol‐report/#!/hgnc_id/HGNC:1318) to compete [http://www.ncbi.nlm.nih.gov/pubmed/15990859?dopt=AbstractPlus, http://www.ncbi.nlm.nih.gov/pubmed/12540846?dopt=AbstractPlus, http://www.ncbi.nlm.nih.gov/pubmed/15833747?dopt=AbstractPlus, http://www.ncbi.nlm.nih.gov/pubmed/12899627?dopt=AbstractPlus]. C5a_2_ appears to lack G protein signalling and has been termed a decoy receptor [http://www.ncbi.nlm.nih.gov/pubmed/19100624?dopt=AbstractPlus]. However, C5a_2_ does recruit arrestin after ligand binding, which might provide a signaling pathway for this receptor [http://www.ncbi.nlm.nih.gov/pubmed/20044484?dopt=AbstractPlus, http://www.ncbi.nlm.nih.gov/pubmed/19641221?dopt=AbstractPlus], and forms heteromers with C5a_1_. C5a, but not C5a‐des Arg, induces upregulation of heteromer formation between complement C5a receptors C5a_1_ and C5a_2_ [http://www.ncbi.nlm.nih.gov/pubmed/24060963?dopt=AbstractPlus]. There are also reports of pro‐inflammatory activity of C5a_2_, mediated by HMGB1, but the signaling pathway that underlies this is currently unclear (reviewed in [http://www.ncbi.nlm.nih.gov/pubmed/23239822?dopt=AbstractPlus]). More recently, work in T cells has shown that C5a_1_ and C5a_2_ act in opposition to each other and that altering the equilibrium between the two receptors, by differential expression or production of C5a‐des Arg (which favours C5a_2_), can affect the final cellular response [http://www.ncbi.nlm.nih.gov/pubmed/27313051?dopt=AbstractPlus].

### Further reading on Complement peptide receptors

Arbore G *et al*. (2016) A novel "complement‐metabolism‐inflammasome axis" as a key regulator of immune cell effector function. *Eur. J. Immunol.* **46**: 1563‐73 [https://www.ncbi.nlm.nih.gov/pubmed/27184294?dopt=AbstractPlus]

Coulthard LG *et al*. (2018) Complement C3a receptor modulates embryonic neural progenitor cell proliferation and cognitive performance. *Mol. Immunol.* **101**: 176‐181 [https://www.ncbi.nlm.nih.gov/pubmed/30449309?dopt=AbstractPlus]

Laumonnier Y *et al*. (2017) Novel insights into the expression pattern of anaphylatoxin receptors in mice and men. *Mol. Immunol.* **89**: 44‐58 [https://www.ncbi.nlm.nih.gov/pubmed/28600003?dopt=AbstractPlus]

Li R *et al*. (2013) C5L2: a controversial receptor of complement anaphylatoxin, C5a. *FASEB J.* **27**: 855‐64 [https://www.ncbi.nlm.nih.gov/pubmed/23239822?dopt=AbstractPlus]

Monk PN *et al*. (2007) Function, structure and therapeutic potential of complement C5a receptors. *Br. J. Pharmacol.* **152**: 429‐48 [https://www.ncbi.nlm.nih.gov/pubmed/17603557?dopt=AbstractPlus]

Reichhardt MP *et al*. (2018) Intracellular complement activation‐An alarm raising mechanism? *Semin. Immunol.* **38**: 54‐62 [https://www.ncbi.nlm.nih.gov/pubmed/29631809?dopt=AbstractPlus]

## http://www.guidetopharmacology.org/GRAC/FamilyDisplayForward?familyId=19

### Overview

Corticotropin‐releasing factor (CRF, **nomenclature as agreed by the NC‐IUPHAR subcommittee on Corticotropin‐releasing Factor Receptors** [http://www.ncbi.nlm.nih.gov/pubmed/12615952?dopt=AbstractPlus]) receptors are activated by the endogenous peptides http://www.guidetopharmacology.org/GRAC/LigandDisplayForward?ligandId=912 (https://www.genenames.org/data/gene‐symbol‐report/#!/hgnc_id/HGNC:2355, http://www.uniprot.org/uniprot/P06850), a 41 aminoacid peptide, http://www.guidetopharmacology.org/GRAC/LigandDisplayForward?ligandId=919 (https://www.genenames.org/data/gene‐symbol‐report/#!/hgnc_id/HGNC:12516, http://www.uniprot.org/uniprot/P55089), 40 amino‐acids, http://www.guidetopharmacology.org/GRAC/LigandDisplayForward?ligandId=921 (https://www.genenames.org/data/gene‐symbol‐report/#!/hgnc_id/HGNC:18414, http://www.uniprot.org/uniprot/Q96RP3), 38 amino‐acids and http://www.guidetopharmacology.org/GRAC/LigandDisplayForward?ligandId=928 (https://www.genenames.org/data/gene‐symbol‐report/#!/hgnc_id/HGNC:17781, http://www.uniprot.org/uniprot/Q969E3), 38 amino‐acids. CRF_1_ and CRF_2_ receptors are activated non‐selectively by CRH and UCN. CRF_2_ receptors are selectively activated by UCN2 and UCN3. Binding to CRF receptors can be conducted using radioligands [^125^I]Tyr^0^‐CRF or [http://www.guidetopharmacology.org/GRAC/LigandDisplayForward?ligandId=5389‐http://www.guidetopharmacology.org/GRAC/LigandDisplayForward?ligandId=5389 with *K*_d_ values of 0.1‐0.4 nM. CRF_1_ and CRF_2_ receptors are non‐selectively antagonized by http://www.guidetopharmacology.org/GRAC/LigandDisplayForward?ligandId=923, http://www.guidetopharmacology.org/GRAC/LigandDisplayForward?ligandId=3865) and http://www.guidetopharmacology.org/GRAC/LigandDisplayForward?ligandId=925. CRF_1_ receptors are selectively antagonized by small molecules http://www.guidetopharmacology.org/GRAC/LigandDisplayForward?ligandId=3512, http://www.guidetopharmacology.org/GRAC/LigandDisplayForward?ligandId=3520, http://www.guidetopharmacology.org/GRAC/LigandDisplayForward?ligandId=3489, http://www.guidetopharmacology.org/GRAC/LigandDisplayForward?ligandId=3495, http://www.guidetopharmacology.org/GRAC/LigandDisplayForward?ligandId=3496. CRF_2_ receptors are selectively antagonized by http://www.guidetopharmacology.org/GRAC/LigandDisplayForward?ligandId=931 and astressin 2B.

**Table:** 

Nomenclature	http://www.guidetopharmacology.org/GRAC/ObjectDisplayForward?objectId=212	http://www.guidetopharmacology.org/GRAC/ObjectDisplayForward?objectId=213
HGNC, UniProt	https://www.genenames.org/data/gene‐symbol‐report/#!/hgnc_id/HGNC:2357, http://www.uniprot.org/uniprot/P34998	https://www.genenames.org/data/gene‐symbol‐report/#!/hgnc_id/HGNC:2358, http://www.uniprot.org/uniprot/Q13324
Endogenous agonists	http://www.guidetopharmacology.org/GRAC/LigandDisplayForward?ligandId=919 (https://www.genenames.org/data/gene‐symbol‐report/#!/hgnc_id/HGNC:12516, http://www.uniprot.org/uniprot/P55089) [http://www.ncbi.nlm.nih.gov/pubmed/15450949?dopt=AbstractPlus, http://www.ncbi.nlm.nih.gov/pubmed/11123370?dopt=AbstractPlus, http://www.ncbi.nlm.nih.gov/pubmed/8612563?dopt=AbstractPlus], http://www.guidetopharmacology.org/GRAC/LigandDisplayForward?ligandId=912 (https://www.genenames.org/data/gene‐symbol‐report/#!/hgnc_id/HGNC:2355, http://www.uniprot.org/uniprot/P06850) [http://www.ncbi.nlm.nih.gov/pubmed/7692441?dopt=AbstractPlus, http://www.ncbi.nlm.nih.gov/pubmed/9326293?dopt=AbstractPlus, http://www.ncbi.nlm.nih.gov/pubmed/11123370?dopt=AbstractPlus, http://www.ncbi.nlm.nih.gov/pubmed/9851694?dopt=AbstractPlus, http://www.ncbi.nlm.nih.gov/pubmed/7477349?dopt=AbstractPlus]	http://www.guidetopharmacology.org/GRAC/LigandDisplayForward?ligandId=921 (https://www.genenames.org/data/gene‐symbol‐report/#!/hgnc_id/HGNC:18414, http://www.uniprot.org/uniprot/Q96RP3) [http://www.ncbi.nlm.nih.gov/pubmed/15450949?dopt=AbstractPlus], http://www.guidetopharmacology.org/GRAC/LigandDisplayForward?ligandId=928 (https://www.genenames.org/data/gene‐symbol‐report/#!/hgnc_id/HGNC:17781, http://www.uniprot.org/uniprot/Q969E3)http://www.uniprot.org/uniprot/Q969E3]
Antagonists	http://www.guidetopharmacology.org/GRAC/LigandDisplayForward?ligandId=3533 (p*K*_i_ 8.7) [http://www.ncbi.nlm.nih.gov/pubmed/11907190?dopt=AbstractPlus], http://www.guidetopharmacology.org/GRAC/LigandDisplayForward?ligandId=925 (p*K*_i_ 8.7) [http://www.ncbi.nlm.nih.gov/pubmed/24269930?dopt=AbstractPlus]	http://www.guidetopharmacology.org/GRAC/LigandDisplayForward?ligandId=925 (pIC_50_ 9.2) [http://www.ncbi.nlm.nih.gov/pubmed/12361401?dopt=AbstractPlus]
Selective antagonists	http://www.guidetopharmacology.org/GRAC/LigandDisplayForward?ligandId=3495 (pIC_50_ 9.3–10.4) [http://www.ncbi.nlm.nih.gov/pubmed/8874139?dopt=AbstractPlus] – Rat, http://www.guidetopharmacology.org/GRAC/LigandDisplayForward?ligandId=3499 (p*K*_i_ 8.3–9) [http://www.ncbi.nlm.nih.gov/pubmed/10669572?dopt=AbstractPlus], http://www.guidetopharmacology.org/GRAC/LigandDisplayForward?ligandId=3512 (p*K*i 8.3–9) [http://www.ncbi.nlm.nih.gov/pubmed/8893829?dopt=AbstractPlus], http://www.guidetopharmacology.org/GRAC/LigandDisplayForward?ligandId=3520 (p*K*_i_ 8.3–9) [http://www.ncbi.nlm.nih.gov/pubmed/10867111?dopt=AbstractPlus], http://www.guidetopharmacology.org/GRAC/LigandDisplayForward?ligandId=3489 (p*K*_i_ 8.3–9) [http://www.ncbi.nlm.nih.gov/pubmed/8940412?dopt=AbstractPlus], http://www.guidetopharmacology.org/GRAC/LigandDisplayForward?ligandId=3496 (pIC_50_ 8.3) [http://www.ncbi.nlm.nih.gov/pubmed/18288792?dopt=AbstractPlus] – Rat, http://www.guidetopharmacology.org/GRAC/LigandDisplayForward?ligandId=3498 (pIC_50_ 6.4–7.1) [http://www.ncbi.nlm.nih.gov/pubmed/10357258?dopt=AbstractPlus]	http://www.guidetopharmacology.org/GRAC/LigandDisplayForward?ligandId=931 (p*K*_d_ 8.8–9.6) [http://www.ncbi.nlm.nih.gov/pubmed/11123370?dopt=AbstractPlus], http://www.guidetopharmacology.org/GRAC/LigandDisplayForward?ligandId=3891 (p*K*_i_ 9.2) [http://www.ncbi.nlm.nih.gov/pubmed/12110614?dopt=AbstractPlus], http://www.guidetopharmacology.org/GRAC/LigandDisplayForward?ligandId=10335 (pIC_50_ 8.9) [http://www.ncbi.nlm.nih.gov/pubmed/12361401?dopt=AbstractPlus], http://www.guidetopharmacology.org/GRAC/LigandDisplayForward?ligandId=3890 (p*K*_i_ 8.7–8.8) [http://www.ncbi.nlm.nih.gov/pubmed/11835994?dopt=AbstractPlus]

### Comments

A CRF binding protein has been identified (https://www.genenames.org/data/gene‐symbol‐report/#!/hgnc_id/HGNC:2356, http://www.uniprot.org/uniprot/P24387) to which both http://www.guidetopharmacology.org/GRAC/LigandDisplayForward?ligandId=912 (https://www.genenames.org/data/gene‐symbol‐report/#!/hgnc_id/HGNC:2355, http://www.uniprot.org/uniprot/P06850) and http://www.guidetopharmacology.org/GRAC/LigandDisplayForward?ligandId=919 (https://www.genenames.org/data/gene‐symbol‐report/#!/hgnc_id/HGNC:12516, http://www.uniprot.org/uniprot/P55089) bind with high affinities, which has been suggested to bind and inactivate circulating http://www.guidetopharmacology.org/GRAC/LigandDisplayForward?ligandId=912, https://www.genenames.org/data/gene‐symbol‐report/#!/hgnc_id/HGNC:2355) [http://www.ncbi.nlm.nih.gov/pubmed/7595134?dopt=AbstractPlus].

### Further reading on Corticotropin‐releasing factor receptors

Deussing JM *et al*. (2018) The Corticotropin‐Releasing Factor Family: Physiology of the Stress Response. *Physiol. Rev.* **98**: 2225‐2286 [https://www.ncbi.nlm.nih.gov/pubmed/30109816?dopt=AbstractPlus]

Hauger RL *et al*. (2003) International Union of Pharmacology. XXXVI. Current status of the nomenclature for receptors for corticotropin‐releasing factor and their ligands. *Pharmacol Rev.* **55**: 21‐26 [https://www.ncbi.nlm.nih.gov/pubmed/12615952?dopt=AbstractPlus]

Grammatopoulos DK. (2012) Insights into mechanisms of corticotropin‐releasing hormone receptor signal transduction. *Br. J. Pharmacol.* **166**: 85‐97 [https://www.ncbi.nlm.nih.gov/pubmed/21883143?dopt=AbstractPlus]

Liapakis G *et al*. (2011) Members of CRF family and their receptors: from past to future. *Curr. Med. Chem.* **18**: 2583‐600 [https://www.ncbi.nlm.nih.gov/pubmed/21568890?dopt=AbstractPlus]

Slater PG *et al*. (2016) Corticotropin‐Releasing Factor Receptors and Their Interacting Proteins: Functional Consequences. *Mol. Pharmacol.* **90**: 627‐632 [https://www.ncbi.nlm.nih.gov/pubmed/27612874?dopt=AbstractPlus]

Zelenay V *et al*. (2017) Structures of the First Extracellular Domain of CRF Receptors. *Curr Mol Pharmacol* **10**: 318‐324 [https://www.ncbi.nlm.nih.gov/pubmed/28103782?dopt=AbstractPlus]

## http://www.guidetopharmacology.org/GRAC/FamilyDisplayForward?familyId=20

### Overview

Dopamine receptors (**nomenclature as agreed by the NC‐IUPHAR Subcommittee on Dopamine Receptors** [1906]) are commonly divided into D_1_‐like (D_1_ and D5) and D_2_‐like (D_2_, D_3_ and D4) families, where the endogenous agonist is http://www.guidetopharmacology.org/GRAC/LigandDisplayForward?ligandId=940.

**Table:** 

Nomenclature	http://www.guidetopharmacology.org/GRAC/ObjectDisplayForward?objectId=214 http://www.guidetopharmacology.org/GRAC/ObjectDisplayForward?objectId=214	http://www.guidetopharmacology.org/GRAC/ObjectDisplayForward?objectId=215 http://www.guidetopharmacology.org/GRAC/ObjectDisplayForward?objectId=215
HGNC, UniProt	https://www.genenames.org/data/gene‐symbol‐report/#!/hgnc_id/HGNC:3020, http://www.uniprot.org/uniprot/P21728	https://www.genenames.org/data/gene‐symbol‐report/#!/hgnc_id/HGNC:3023, http://www.uniprot.org/uniprot/P14416
Sub/family‐selective labelled ligands	[http://www.guidetopharmacology.org/GRAC/LigandDisplayForward?ligandId=945 (Antagonist) (p*K*_d_ 9.5) [http://www.ncbi.nlm.nih.gov/pubmed/2144334?dopt=AbstractPlus], [http://www.guidetopharmacology.org/GRAC/LigandDisplayForward?ligandId=946 (Antagonist) (p*K*_d_ 9.5) [http://www.ncbi.nlm.nih.gov/pubmed/2168520?dopt=AbstractPlus]	[http://www.guidetopharmacology.org/GRAC/LigandDisplayForward?ligandId=3300 (Antagonist) (p*K*_d_ 10.2) [http://www.ncbi.nlm.nih.gov/pubmed/2974511?dopt=AbstractPlus, http://www.ncbi.nlm.nih.gov/pubmed/10882389?dopt=AbstractPlus, http://www.ncbi.nlm.nih.gov/pubmed/20122961?dopt=AbstractPlus] – Rat
Endogenous agonists	http://www.guidetopharmacology.org/GRAC/LigandDisplayForward?ligandId=940 [http://www.ncbi.nlm.nih.gov/pubmed/1826762?dopt=AbstractPlus, http://www.ncbi.nlm.nih.gov/pubmed/7525564?dopt=AbstractPlus]	http://www.guidetopharmacology.org/GRAC/LigandDisplayForward?ligandId=940 [http://www.ncbi.nlm.nih.gov/pubmed/7576010?dopt=AbstractPlus, http://www.ncbi.nlm.nih.gov/pubmed/8301582?dopt=AbstractPlus, http://www.ncbi.nlm.nih.gov/pubmed/7756621?dopt=AbstractPlus]
Agonists	http://www.guidetopharmacology.org/GRAC/LigandDisplayForward?ligandId=939 [http://www.ncbi.nlm.nih.gov/pubmed/7525564?dopt=AbstractPlus]	http://www.guidetopharmacology.org/GRAC/LigandDisplayForward?ligandId=941 [http://www.ncbi.nlm.nih.gov/pubmed/12086487?dopt=AbstractPlus], http://www.guidetopharmacology.org/GRAC/LigandDisplayForward?ligandId=37 (Partial agonist) [http://www.ncbi.nlm.nih.gov/pubmed/12388666?dopt=AbstractPlus], http://www.guidetopharmacology.org/GRAC/LigandDisplayForward?ligandId=34 (Partial agonist) [http://www.ncbi.nlm.nih.gov/pubmed/23279866?dopt=AbstractPlus], http://www.guidetopharmacology.org/GRAC/LigandDisplayForward?ligandId=35 [http://www.ncbi.nlm.nih.gov/pubmed/8301582?dopt=AbstractPlus, http://www.ncbi.nlm.nih.gov/pubmed/8301582?dopt=AbstractPlus, http://www.ncbi.nlm.nih.gov/pubmed/7756621?dopt=AbstractPlus], http://www.guidetopharmacology.org/GRAC/LigandDisplayForward?ligandId=8369 (Biased agonist) [http://www.ncbi.nlm.nih.gov/pubmed/24755247?dopt=AbstractPlus], http://www.guidetopharmacology.org/GRAC/LigandDisplayForward?ligandId=7295 [http://www.ncbi.nlm.nih.gov/pubmed/9057850?dopt=AbstractPlus], http://www.guidetopharmacology.org/GRAC/LigandDisplayForward?ligandId=33 (Partial agonist) [http://www.ncbi.nlm.nih.gov/pubmed/7576010?dopt=AbstractPlus, http://www.ncbi.nlm.nih.gov/pubmed/7576010?dopt=AbstractPlus, http://www.ncbi.nlm.nih.gov/pubmed/12388666?dopt=AbstractPlus, http://www.ncbi.nlm.nih.gov/pubmed/1354163?dopt=AbstractPlus], http://www.guidetopharmacology.org/GRAC/LigandDisplayForward?ligandId=953 [http://www.ncbi.nlm.nih.gov/pubmed/7664822?dopt=AbstractPlus, http://www.ncbi.nlm.nih.gov/pubmed/7664822?dopt=AbstractPlus], http://www.guidetopharmacology.org/GRAC/LigandDisplayForward?ligandId=7124 [http://www.ncbi.nlm.nih.gov/pubmed/22711801?dopt=AbstractPlus]
Sub/family‐selective agonists	http://www.guidetopharmacology.org/GRAC/LigandDisplayForward?ligandId=6077 [http://www.ncbi.nlm.nih.gov/pubmed/16318870?dopt=AbstractPlus], http://www.guidetopharmacology.org/GRAC/LigandDisplayForward?ligandId=935 (Partial agonist) [http://www.ncbi.nlm.nih.gov/pubmed/1826762?dopt=AbstractPlus, http://www.ncbi.nlm.nih.gov/pubmed/7525564?dopt=AbstractPlus]	http://www.guidetopharmacology.org/GRAC/LigandDisplayForward?ligandId=2 [http://www.ncbi.nlm.nih.gov/pubmed/7576010?dopt=AbstractPlus, http://www.ncbi.nlm.nih.gov/pubmed/7664822?dopt=AbstractPlus, http://www.ncbi.nlm.nih.gov/pubmed/8967990?dopt=AbstractPlus, http://www.ncbi.nlm.nih.gov/pubmed/1354163?dopt=AbstractPlus, http://www.ncbi.nlm.nih.gov/pubmed/1975644?dopt=AbstractPlus, http://www.ncbi.nlm.nih.gov/pubmed/1840645?dopt=AbstractPlus]
Selective agonists	http://www.guidetopharmacology.org/GRAC/LigandDisplayForward?ligandId=8443 (Biased agonist) [http://www.ncbi.nlm.nih.gov/pubmed/25660762?dopt=AbstractPlus], http://www.guidetopharmacology.org/GRAC/LigandDisplayForward?ligandId=9637 [http://www.ncbi.nlm.nih.gov/pubmed/17067639?dopt=AbstractPlus], http://www.guidetopharmacology.org/GRAC/LigandDisplayForward?ligandId=938 [http://www.ncbi.nlm.nih.gov/pubmed/1973652?dopt=AbstractPlus] – Rat	http://www.guidetopharmacology.org/GRAC/LigandDisplayForward?ligandId=3949 [http://www.ncbi.nlm.nih.gov/pubmed/15980060?dopt=AbstractPlus]
Antagonists	http://www.guidetopharmacology.org/GRAC/LigandDisplayForward?ligandId=948 (p*K*_i_ 7–8.4) [http://www.ncbi.nlm.nih.gov/pubmed/1826762?dopt=AbstractPlus, http://www.ncbi.nlm.nih.gov/pubmed/7525564?dopt=AbstractPlus]	http://www.guidetopharmacology.org/GRAC/LigandDisplayForward?ligandId=7670 (p*K*_i_ 9.9) [http://www.ncbi.nlm.nih.gov/pubmed/15686911?dopt=AbstractPlus], http://www.guidetopharmacology.org/GRAC/LigandDisplayForward?ligandId=7557 (p*K*_i_ 9.7) [http://www.ncbi.nlm.nih.gov/pubmed/1586393?dopt=AbstractPlus], http://www.guidetopharmacology.org/GRAC/LigandDisplayForward?ligandId=209 (p*K*i 8.9–9.6) [http://www.ncbi.nlm.nih.gov/pubmed/12629531?dopt=AbstractPlus, 1920], http://www.guidetopharmacology.org/GRAC/LigandDisplayForward?ligandId=96 (p*K*_i_ 9.4) [http://www.ncbi.nlm.nih.gov/pubmed/9430133?dopt=AbstractPlus], http://www.guidetopharmacology.org/GRAC/LigandDisplayForward?ligandId=7556 (p*K*_i_ 9.2) [http://www.ncbi.nlm.nih.gov/pubmed/1060115?dopt=AbstractPlus], http://www.guidetopharmacology.org/GRAC/LigandDisplayForward?ligandId=214 (p*K*_i_ 8.9–9) [http://www.ncbi.nlm.nih.gov/pubmed/12629531?dopt=AbstractPlus, http://www.ncbi.nlm.nih.gov/pubmed/9015795?dopt=AbstractPlus], http://www.guidetopharmacology.org/GRAC/LigandDisplayForward?ligandId=50 (p*K*_i_ 7.2) [http://www.ncbi.nlm.nih.gov/pubmed/9430133?dopt=AbstractPlus]
Sub/family‐selective antagonists	http://www.guidetopharmacology.org/GRAC/LigandDisplayForward?ligandId=943 (p*K*_i_ 7.4–9.5) [http://www.ncbi.nlm.nih.gov/pubmed/1826762?dopt=AbstractPlus, http://www.ncbi.nlm.nih.gov/pubmed/7525564?dopt=AbstractPlus], http://www.guidetopharmacology.org/GRAC/LigandDisplayForward?ligandId=944 (p*K*_i_ 9.5) [http://www.ncbi.nlm.nih.gov/pubmed/1826762?dopt=AbstractPlus], http://www.guidetopharmacology.org/GRAC/LigandDisplayForward?ligandId=3304 (p*K*i 8.3) [http://www.ncbi.nlm.nih.gov/pubmed/7862709?dopt=AbstractPlus]	http://www.guidetopharmacology.org/GRAC/LigandDisplayForward?ligandId=86 (p*K*_i_ 7.4–8.8) [http://www.ncbi.nlm.nih.gov/pubmed/8301582?dopt=AbstractPlus, http://www.ncbi.nlm.nih.gov/pubmed/7907989?dopt=AbstractPlus, http://www.ncbi.nlm.nih.gov/pubmed/7664822?dopt=AbstractPlus, http://www.ncbi.nlm.nih.gov/pubmed/1354163?dopt=AbstractPlus, http://www.ncbi.nlm.nih.gov/pubmed/7862709?dopt=AbstractPlus]
Selective antagonists	–	http://www.guidetopharmacology.org/GRAC/LigandDisplayForward?ligandId=177 (p*K*_i_ 7.9–8.5) [http://www.ncbi.nlm.nih.gov/pubmed/17095222?dopt=AbstractPlus, http://www.ncbi.nlm.nih.gov/pubmed/8642550?dopt=AbstractPlus], http://www.guidetopharmacology.org/GRAC/LigandDisplayForward?ligandId=965 (p*K*_i_ 7.9–8.4) [http://www.ncbi.nlm.nih.gov/pubmed/8301582?dopt=AbstractPlus, http://www.ncbi.nlm.nih.gov/pubmed/1354163?dopt=AbstractPlus], http://www.guidetopharmacology.org/GRAC/LigandDisplayForward?ligandId=94 (p*K*_i_ 8) [http://www.ncbi.nlm.nih.gov/pubmed/7473180?dopt=AbstractPlus], http://www.guidetopharmacology.org/GRAC/LigandDisplayForward?ligandId=8368 (p*K*_i_ 7) [http://www.ncbi.nlm.nih.gov/pubmed/24260782?dopt=AbstractPlus, http://www.ncbi.nlm.nih.gov/pubmed/24666157?dopt=AbstractPlus]
Labelled ligands	–	[http://www.guidetopharmacology.org/GRAC/LigandDisplayForward?ligandId=3299 (Antagonist) (p*K*_d_ 8.9) [http://www.ncbi.nlm.nih.gov/pubmed/4015674?dopt=AbstractPlus] – Rat

**Table:** 

Nomenclature	http://www.guidetopharmacology.org/GRAC/ObjectDisplayForward?objectId=216 http://www.guidetopharmacology.org/GRAC/ObjectDisplayForward?objectId=216	http://www.guidetopharmacology.org/GRAC/ObjectDisplayForward?objectId=217 http://www.guidetopharmacology.org/GRAC/ObjectDisplayForward?objectId=217	http://www.guidetopharmacology.org/GRAC/ObjectDisplayForward?objectId=218 http://www.guidetopharmacology.org/GRAC/ObjectDisplayForward?objectId=218
HGNC, UniProt	https://www.genenames.org/data/gene‐symbol‐report/#!/hgnc_id/HGNC:3024, http://www.uniprot.org/uniprot/P35462	https://www.genenames.org/data/gene‐symbol‐report/#!/hgnc_id/HGNC:3025, http://www.uniprot.org/uniprot/P21917	https://www.genenames.org/data/gene‐symbol‐report/#!/hgnc_id/HGNC:3026, http://www.uniprot.org/uniprot/P21918
Sub/family‐selective labelled ligands	–	[http://www.guidetopharmacology.org/GRAC/LigandDisplayForward?ligandId=3300 (Antagonist) (p*K*_d_ 9.5) [http://www.ncbi.nlm.nih.gov/pubmed/7777184?dopt=AbstractPlus, http://www.ncbi.nlm.nih.gov/pubmed/1840645?dopt=AbstractPlus]	[http://www.guidetopharmacology.org/GRAC/LigandDisplayForward?ligandId=946 (Antagonist) (p*K*_d_ 9.2) [http://www.ncbi.nlm.nih.gov/pubmed/8051291?dopt=AbstractPlus]
Endogenous agonists	http://www.guidetopharmacology.org/GRAC/LigandDisplayForward?ligandId=940 [http://www.ncbi.nlm.nih.gov/pubmed/7576010?dopt=AbstractPlus, http://www.ncbi.nlm.nih.gov/pubmed/8301582?dopt=AbstractPlus, http://www.ncbi.nlm.nih.gov/pubmed/7756621?dopt=AbstractPlus, http://www.ncbi.nlm.nih.gov/pubmed/1975644?dopt=AbstractPlus]	http://www.guidetopharmacology.org/GRAC/LigandDisplayForward?ligandId=940 [http://www.ncbi.nlm.nih.gov/pubmed/1840645?dopt=AbstractPlus]	http://www.guidetopharmacology.org/GRAC/LigandDisplayForward?ligandId=940 [http://www.ncbi.nlm.nih.gov/pubmed/1826762?dopt=AbstractPlus]
Agonists	http://www.guidetopharmacology.org/GRAC/LigandDisplayForward?ligandId=953 [http://www.ncbi.nlm.nih.gov/pubmed/7664822?dopt=AbstractPlus, http://www.ncbi.nlm.nih.gov/pubmed/7756621?dopt=AbstractPlus], http://www.guidetopharmacology.org/GRAC/LigandDisplayForward?ligandId=35 (Partial agonist) [http://www.ncbi.nlm.nih.gov/pubmed/8301582?dopt=AbstractPlus, http://www.ncbi.nlm.nih.gov/pubmed/12388666?dopt=AbstractPlus], http://www.guidetopharmacology.org/GRAC/LigandDisplayForward?ligandId=7295 [http://www.ncbi.nlm.nih.gov/pubmed/9057850?dopt=AbstractPlus], http://www.guidetopharmacology.org/GRAC/LigandDisplayForward?ligandId=33 (Partial agonist) [http://www.ncbi.nlm.nih.gov/pubmed/7576010?dopt=AbstractPlus, http://www.ncbi.nlm.nih.gov/pubmed/7576010?dopt=AbstractPlus, http://www.ncbi.nlm.nih.gov/pubmed/8301582?dopt=AbstractPlus, http://www.ncbi.nlm.nih.gov/pubmed/12388666?dopt=AbstractPlus, http://www.ncbi.nlm.nih.gov/pubmed/1354163?dopt=AbstractPlus]	http://www.guidetopharmacology.org/GRAC/LigandDisplayForward?ligandId=33 (Partial agonist) [http://www.ncbi.nlm.nih.gov/pubmed/12388666?dopt=AbstractPlus]	–
Sub/family‐selective agonists	http://www.guidetopharmacology.org/GRAC/LigandDisplayForward?ligandId=2 [http://www.ncbi.nlm.nih.gov/pubmed/7576010?dopt=AbstractPlus, http://www.ncbi.nlm.nih.gov/pubmed/7664822?dopt=AbstractPlus, http://www.ncbi.nlm.nih.gov/pubmed/7473180?dopt=AbstractPlus, http://www.ncbi.nlm.nih.gov/pubmed/8967990?dopt=AbstractPlus, http://www.ncbi.nlm.nih.gov/pubmed/7756621?dopt=AbstractPlus, http://www.ncbi.nlm.nih.gov/pubmed/1354163?dopt=AbstractPlus, http://www.ncbi.nlm.nih.gov/pubmed/1975644?dopt=AbstractPlus, http://www.ncbi.nlm.nih.gov/pubmed/1840645?dopt=AbstractPlus]	http://www.guidetopharmacology.org/GRAC/LigandDisplayForward?ligandId=2 [http://www.ncbi.nlm.nih.gov/pubmed/12388666?dopt=AbstractPlus, http://www.ncbi.nlm.nih.gov/pubmed/8967990?dopt=AbstractPlus, http://www.ncbi.nlm.nih.gov/pubmed/1840645?dopt=AbstractPlus]	http://www.guidetopharmacology.org/GRAC/LigandDisplayForward?ligandId=6077 [http://www.ncbi.nlm.nih.gov/pubmed/16318870?dopt=AbstractPlus]
Selective agonists	http://www.guidetopharmacology.org/GRAC/LigandDisplayForward?ligandId=952 [http://www.ncbi.nlm.nih.gov/pubmed/8531103?dopt=AbstractPlus, http://www.ncbi.nlm.nih.gov/pubmed/7756621?dopt=AbstractPlus]	http://www.guidetopharmacology.org/GRAC/LigandDisplayForward?ligandId=975 (Partial agonist) [http://www.ncbi.nlm.nih.gov/pubmed/15448188?dopt=AbstractPlus] – Rat, http://www.guidetopharmacology.org/GRAC/LigandDisplayForward?ligandId=3301 [http://www.ncbi.nlm.nih.gov/pubmed/16153699?dopt=AbstractPlus] – Rat, http://www.guidetopharmacology.org/GRAC/LigandDisplayForward?ligandId=3301]	–
Antagonists	http://www.guidetopharmacology.org/GRAC/LigandDisplayForward?ligandId=7556 (p*K*_i_ 9.6) [http://www.ncbi.nlm.nih.gov/pubmed/1354163?dopt=AbstractPlus], http://www.guidetopharmacology.org/GRAC/LigandDisplayForward?ligandId=98 (p*K*_i_ 8–8.8) [http://www.ncbi.nlm.nih.gov/pubmed/9430133?dopt=AbstractPlus, http://www.ncbi.nlm.nih.gov/pubmed/8935801?dopt=AbstractPlus, 1920], http://www.guidetopharmacology.org/GRAC/LigandDisplayForward?ligandId=7279 (p*K*_i_ 8.4) [79], (‐)‐http://www.guidetopharmacology.org/GRAC/LigandDisplayForward?ligandId=958 (p*K*_i_ 6.7–7.7) [http://www.ncbi.nlm.nih.gov/pubmed/8301582?dopt=AbstractPlus, http://www.ncbi.nlm.nih.gov/pubmed/1354163?dopt=AbstractPlus, http://www.ncbi.nlm.nih.gov/pubmed/8301592?dopt=AbstractPlus], http://www.guidetopharmacology.org/GRAC/LigandDisplayForward?ligandId=205 (p*K*_i_ 7.7) [1920], http://www.guidetopharmacology.org/GRAC/LigandDisplayForward?ligandId=965 (p*K*_i_ 7.1–7.6) [http://www.ncbi.nlm.nih.gov/pubmed/8301582?dopt=AbstractPlus, http://www.ncbi.nlm.nih.gov/pubmed/8301582?dopt=AbstractPlus], http://www.guidetopharmacology.org/GRAC/LigandDisplayForward?ligandId=7281 (p*K*_i_ 6.8) [http://www.ncbi.nlm.nih.gov/pubmed/16135699?dopt=AbstractPlus]	http://www.guidetopharmacology.org/GRAC/LigandDisplayForward?ligandId=7556 (p*K*_i_ 10.1) [http://www.ncbi.nlm.nih.gov/pubmed/9577836?dopt=AbstractPlus], http://www.guidetopharmacology.org/GRAC/LigandDisplayForward?ligandId=98 (p*K*_i_ 7.8–9.1) [http://www.ncbi.nlm.nih.gov/pubmed/16135699?dopt=AbstractPlus, 1920, http://www.ncbi.nlm.nih.gov/pubmed/9015795?dopt=AbstractPlus], http://www.guidetopharmacology.org/GRAC/LigandDisplayForward?ligandId=980 (p*K*_i_ 8.9) [http://www.ncbi.nlm.nih.gov/pubmed/9098699?dopt=AbstractPlus], http://www.guidetopharmacology.org/GRAC/LigandDisplayForward?ligandId=205 (p*K*_i_ 8.1) [http://www.ncbi.nlm.nih.gov/pubmed/9015795?dopt=AbstractPlus]	–
Sub/family‐selective antagonists	http://www.guidetopharmacology.org/GRAC/LigandDisplayForward?ligandId=86 (p*K*_i_ 7.5–8.6) [http://www.ncbi.nlm.nih.gov/pubmed/8301582?dopt=AbstractPlus, http://www.ncbi.nlm.nih.gov/pubmed/18308814?dopt=AbstractPlus, http://www.ncbi.nlm.nih.gov/pubmed/1354163?dopt=AbstractPlus, http://www.ncbi.nlm.nih.gov/pubmed/7862709?dopt=AbstractPlus]	http://www.guidetopharmacology.org/GRAC/LigandDisplayForward?ligandId=86 (p*K*_i_ 8.7–8.8) [http://www.ncbi.nlm.nih.gov/pubmed/8102973?dopt=AbstractPlus, http://www.ncbi.nlm.nih.gov/pubmed/18308814?dopt=AbstractPlus, http://www.ncbi.nlm.nih.gov/pubmed/7862709?dopt=AbstractPlus]	http://www.guidetopharmacology.org/GRAC/LigandDisplayForward?ligandId=943 (p*K*_i_ 7.5–9.5) [http://www.ncbi.nlm.nih.gov/pubmed/1826762?dopt=AbstractPlus], http://www.guidetopharmacology.org/GRAC/LigandDisplayForward?ligandId=944 (p*K*_i_ 9.4) [http://www.ncbi.nlm.nih.gov/pubmed/1826762?dopt=AbstractPlus], http://www.guidetopharmacology.org/GRAC/LigandDisplayForward?ligandId=3304 (p*K*_i_ 8.3) [http://www.ncbi.nlm.nih.gov/pubmed/1826762?dopt=AbstractPlus]
Selective antagonists	http://www.guidetopharmacology.org/GRAC/LigandDisplayForward?ligandId=129 (p*K*_i_ 9.6) [http://www.ncbi.nlm.nih.gov/pubmed/10869410?dopt=AbstractPlus], http://www.guidetopharmacology.org/GRAC/LigandDisplayForward?ligandId=44 (p*K*_i_ 9.5) [http://www.ncbi.nlm.nih.gov/pubmed/8531087?dopt=AbstractPlus], http://www.guidetopharmacology.org/GRAC/LigandDisplayForward?ligandId=6675 (p*K*_i_ 9.2) [http://www.ncbi.nlm.nih.gov/pubmed/17672446?dopt=AbstractPlus], http://www.guidetopharmacology.org/GRAC/LigandDisplayForward?ligandId=6674 (p*K*_i_ 8.8) [http://www.ncbi.nlm.nih.gov/pubmed/17627675?dopt=AbstractPlus], http://www.guidetopharmacology.org/GRAC/LigandDisplayForward?ligandId=143 (p*K*_i_ 8) [http://www.ncbi.nlm.nih.gov/pubmed/10945872?dopt=AbstractPlus], (http://www.guidetopharmacology.org/GRAC/LigandDisplayForward?ligandId=955 (p*K*_i_ 6.9–7.9) [http://www.ncbi.nlm.nih.gov/pubmed/7988633?dopt=AbstractPlus, http://www.ncbi.nlm.nih.gov/pubmed/7473180?dopt=AbstractPlus]	http://www.guidetopharmacology.org/GRAC/LigandDisplayForward?ligandId=3303 (p*K*_i_ 9.4) [http://www.ncbi.nlm.nih.gov/pubmed/8642550?dopt=AbstractPlus], http://www.guidetopharmacology.org/GRAC/LigandDisplayForward?ligandId=8441 (p*K*_i_ 8.8) [http://www.ncbi.nlm.nih.gov/pubmed/15992586?dopt=AbstractPlus], http://www.guidetopharmacology.org/GRAC/LigandDisplayForward?ligandId=3302 (p*K*_i_ 8.5) [http://www.ncbi.nlm.nih.gov/pubmed/8642551?dopt=AbstractPlus], http://www.guidetopharmacology.org/GRAC/LigandDisplayForward?ligandId=8440 (p*K*_i_ 7.4) [http://www.ncbi.nlm.nih.gov/pubmed/25221667?dopt=AbstractPlus]	–
Selective allosteric modulators	http://www.guidetopharmacology.org/GRAC/LigandDisplayForward?ligandId=7694 (Negative) (p*K*i ∼9) [http://www.ncbi.nlm.nih.gov/pubmed/25583363?dopt=AbstractPlus]	–	–
Labelled ligands	[http://www.guidetopharmacology.org/GRAC/LigandDisplayForward?ligandId=3300 (Antagonist) (p*K*_d_ 9.9) [http://www.ncbi.nlm.nih.gov/pubmed/10882389?dopt=AbstractPlus, http://www.ncbi.nlm.nih.gov/pubmed/20122961?dopt=AbstractPlus] – Rat, [http://www.guidetopharmacology.org/GRAC/LigandDisplayForward?ligandId=3296 (Agonist) [http://www.ncbi.nlm.nih.gov/pubmed/7759603?dopt=AbstractPlus], [http://www.guidetopharmacology.org/GRAC/LigandDisplayForward?ligandId=3298 (Agonist) [http://www.ncbi.nlm.nih.gov/pubmed/7674830?dopt=AbstractPlus]	[http://www.guidetopharmacology.org/GRAC/LigandDisplayForward?ligandId=3295 (Antagonist) (p*K*_d_ 9.8) [http://www.ncbi.nlm.nih.gov/pubmed/8967990?dopt=AbstractPlus], [http://www.guidetopharmacology.org/GRAC/LigandDisplayForward?ligandId=3297 (Antagonist) (p*K*_d_ 8.3) [http://www.ncbi.nlm.nih.gov/pubmed/9262371?dopt=AbstractPlus]	[http://www.guidetopharmacology.org/GRAC/LigandDisplayForward?ligandId=945 (Antagonist) (p*K*_d_ 9.1)

### Comments

The selectivity of many of these agents is less than two orders of magnitude. [http://www.guidetopharmacology.org/GRAC/LigandDisplayForward?ligandId=3299 exhibits similar high affinity for D_2_ and D_3_ receptors (low affinity for D4), but has been used to label D_2_ receptors in the presence of a D_3_‐selective antagonist. [http://www.guidetopharmacology.org/GRAC/LigandDisplayForward?ligandId=3296 has similar affinity for D_2_ and D_3_ receptors, but labels only D_3_ receptors in the absence of divalent cations. The pharmacological profile of the D5 receptor is similar to, yet distinct from, that of the D_1_ receptor. The splice variants of the D_2_ receptor are commonly termed D_2_S and D_2_L (short and long). The *DRD4* gene encoding the D4 receptor is highly polymorphic in humans, with allelic variations of the protein from amino acid 387 to 515.

### Further reading on Dopamine receptors

Beaulieu JM *et al*. (2015) Dopamine receptors ‐ IUPHAR Review 13. *Br. J. Pharmacol.* **172**: 1‐23 [https://www.ncbi.nlm.nih.gov/pubmed/25671228?dopt=AbstractPlus]

Beaulieu JM *et al*. (2011) The physiology, signaling, and pharmacology of dopamine receptors. *Pharmacol. Rev.* **63**: 182‐217 [https://www.ncbi.nlm.nih.gov/pubmed/21303898?dopt=AbstractPlus]

Cumming P. (2011) Absolute abundances and affinity states of dopamine receptors in mammalian brain: A review. *Synapse* **65**: 892‐909 [https://www.ncbi.nlm.nih.gov/pubmed/21308799?dopt=AbstractPlus]

Maggio R *et al*. (2010) Dopamine D2‐D3 receptor heteromers: pharmacological properties and therapeutic significance. *Curr Opin Pharmacol* **10**: 100‐7 [https://www.ncbi.nlm.nih.gov/pubmed/19896900?dopt=AbstractPlus]

Ptácek R *et al*. (2011) Dopamine D4 receptor gene DRD4 and its association with psychiatric disorders. *Med. Sci. Monit.* **17**: RA215‐20 [https://www.ncbi.nlm.nih.gov/pubmed/21873960?dopt=AbstractPlus]

Schwartz J‐C *et al*. (1998) Dopamine Receptors. *In The IUPHAR Compendium of Receptor Characterization and Classification* Edited by Girdlestone D: IUPHAR Media: 141‐151

Undieh AS. (2010) Pharmacology of signaling induced by dopamine D(1)‐like receptor activation. *Pharmacol. Ther.* **128**: 37‐60 [https://www.ncbi.nlm.nih.gov/pubmed/20547182?dopt=AbstractPlus]

## http://www.guidetopharmacology.org/GRAC/FamilyDisplayForward?familyId=21

### Overview

Endothelin receptors (**nomenclature as agreed by the NC‐IUPHAR Subcommittee on Endothelin Receptors** [http://www.ncbi.nlm.nih.gov/pubmed/12037137?dopt=AbstractPlus]) are activated by the endogenous 21 amino‐acid peptides endothelins 1‐3 (http://www.guidetopharmacology.org/GRAC/LigandDisplayForward?ligandId=989 (https://www.genenames.org/data/gene‐symbol‐report/#!/hgnc_id/HGNC:3176, http://www.uniprot.org/uniprot/P05305), http://www.guidetopharmacology.org/GRAC/LigandDisplayForward?ligandId=990 (https://www.genenames.org/data/gene‐symbol‐report/#!/hgnc_id/HGNC:3177, http://www.uniprot.org/uniprot/P20800) and http://www.guidetopharmacology.org/GRAC/LigandDisplayForward?ligandId=1004 (https://www.genenames.org/data/gene‐symbol‐report/#!/hgnc_id/HGNC:3178, http://www.uniprot.org/uniprot/P14138)).

**Table:** 

Nomenclature	http://www.guidetopharmacology.org/GRAC/ObjectDisplayForward?objectId=219	http://www.guidetopharmacology.org/GRAC/ObjectDisplayForward?objectId=220
HGNC, UniProt	https://www.genenames.org/data/gene‐symbol‐report/#!/hgnc_id/HGNC:3179, http://www.uniprot.org/uniprot/P25101	https://www.genenames.org/data/gene‐symbol‐report/#!/hgnc_id/HGNC:3180, http://www.uniprot.org/uniprot/P24530
Potency order of endogenous ligands	http://www.guidetopharmacology.org/GRAC/LigandDisplayForward?ligandId=989 (https://www.genenames.org/data/gene‐symbol‐report/#!/hgnc_id/HGNC:3176, http://www.uniprot.org/uniprot/P05305) = http://www.guidetopharmacology.org/GRAC/LigandDisplayForward?ligandId=990 (https://www.genenames.org/data/gene‐symbol‐report/#!/hgnc_id/HGNC:3177, http://www.uniprot.org/uniprot/P20800) > http://www.guidetopharmacology.org/GRAC/LigandDisplayForward?ligandId=1004 (https://www.genenames.org/data/gene‐symbol‐report/#!/hgnc_id/HGNC:3178, http://www.uniprot.org/uniprot/P14138) [http://www.ncbi.nlm.nih.gov/pubmed/7647976?dopt=AbstractPlus]	http://www.guidetopharmacology.org/GRAC/LigandDisplayForward?ligandId=989 (https://www.genenames.org/data/gene‐symbol‐report/#!/hgnc_id/HGNC:3176, http://www.uniprot.org/uniprot/P05305) = http://www.guidetopharmacology.org/GRAC/LigandDisplayForward?ligandId=990 (https://www.genenames.org/data/gene‐symbol‐report/#!/hgnc_id/HGNC:3177, http://www.uniprot.org/uniprot/P20800), http://www.guidetopharmacology.org/GRAC/LigandDisplayForward?ligandId=1004 (https://www.genenames.org/data/gene‐symbol‐report/#!/hgnc_id/HGNC:3178, http://www.uniprot.org/uniprot/P14138) [http://www.ncbi.nlm.nih.gov/pubmed/2175397?dopt=AbstractPlus]
Selective agonists	–	http://www.guidetopharmacology.org/GRAC/LigandDisplayForward?ligandId=1008 [http://www.ncbi.nlm.nih.gov/pubmed/8587419?dopt=AbstractPlus, http://www.ncbi.nlm.nih.gov/pubmed/8904635?dopt=AbstractPlus], http://www.guidetopharmacology.org/GRAC/LigandDisplayForward?ligandId=3859 [http://www.ncbi.nlm.nih.gov/pubmed/7733918?dopt=AbstractPlus], [http://www.guidetopharmacology.org/GRAC/LigandDisplayForward?ligandId=3837]http://www.guidetopharmacology.org/GRAC/LigandDisplayForward?ligandId=3837 [http://www.ncbi.nlm.nih.gov/pubmed/1472961?dopt=AbstractPlus], http://www.guidetopharmacology.org/GRAC/LigandDisplayForward?ligandId=3886 [http://www.ncbi.nlm.nih.gov/pubmed/1320877?dopt=AbstractPlus]
Antagonists	http://www.guidetopharmacology.org/GRAC/LigandDisplayForward?ligandId=3528 (p*K*_B_ 9.4) [http://www.ncbi.nlm.nih.gov/pubmed/8201588?dopt=AbstractPlus] – Rat, http://www.guidetopharmacology.org/GRAC/LigandDisplayForward?ligandId=3913 (p*A*_2_ 8.4) [http://www.ncbi.nlm.nih.gov/pubmed/7780649?dopt=AbstractPlus] – Rat, http://www.guidetopharmacology.org/GRAC/LigandDisplayForward?ligandId=3494 (p*A*_2_ 7.2) [http://www.ncbi.nlm.nih.gov/pubmed/8035319?dopt=AbstractPlus] – Rat	http://www.guidetopharmacology.org/GRAC/LigandDisplayForward?ligandId=3528 (p*K*_B_ 9.4) [http://www.ncbi.nlm.nih.gov/pubmed/8201588?dopt=AbstractPlus] – Rat, http://www.guidetopharmacology.org/GRAC/LigandDisplayForward?ligandId=3913 (p*A*_2_ 8.4) [http://www.ncbi.nlm.nih.gov/pubmed/7780649?dopt=AbstractPlus] – Rat, http://www.guidetopharmacology.org/GRAC/LigandDisplayForward?ligandId=3494 (p*K*i 7.1) [http://www.ncbi.nlm.nih.gov/pubmed/12502366?dopt=AbstractPlus]
Selective antagonists	http://www.guidetopharmacology.org/GRAC/LigandDisplayForward?ligandId=7352 (pIC_50_ 9.3) [http://www.ncbi.nlm.nih.gov/pubmed/22862294?dopt=AbstractPlus], http://www.guidetopharmacology.org/GRAC/LigandDisplayForward?ligandId=3950 (p*A*_2_ 8) [http://www.ncbi.nlm.nih.gov/pubmed/9171878?dopt=AbstractPlus], http://www.guidetopharmacology.org/GRAC/LigandDisplayForward?ligandId=998 (Inverse agonist) (pIC_50_ 7.3–7.9) [http://www.ncbi.nlm.nih.gov/pubmed/7647976?dopt=AbstractPlus], http://www.guidetopharmacology.org/GRAC/LigandDisplayForward?ligandId=997 (p*A*_2_ 6.9–7.4) [http://www.ncbi.nlm.nih.gov/pubmed/7647976?dopt=AbstractPlus], http://www.guidetopharmacology.org/GRAC/LigandDisplayForward?ligandId=3951 (p*A*_2_ 7.1) [http://www.ncbi.nlm.nih.gov/pubmed/15139756?dopt=AbstractPlus]	http://www.guidetopharmacology.org/GRAC/LigandDisplayForward?ligandId=9651 (pIC_50_ 8.2) [http://www.ncbi.nlm.nih.gov/pubmed/28805809?dopt=AbstractPlus], http://www.guidetopharmacology.org/GRAC/LigandDisplayForward?ligandId=1009 (p*K*_d_ 8.1) [http://www.ncbi.nlm.nih.gov/pubmed/10479298?dopt=AbstractPlus], http://www.guidetopharmacology.org/GRAC/LigandDisplayForward?ligandId=1010 (p*K*_d_ 7.9–8) [http://www.ncbi.nlm.nih.gov/pubmed/8904635?dopt=AbstractPlus], http://www.guidetopharmacology.org/GRAC/LigandDisplayForward?ligandId=3887 (p*K*_d_ 7.2) [http://www.ncbi.nlm.nih.gov/pubmed/8904635?dopt=AbstractPlus], http://www.guidetopharmacology.org/GRAC/LigandDisplayForward?ligandId=1011 (pIC_50_ 7.2) [http://www.ncbi.nlm.nih.gov/pubmed/8612786?dopt=AbstractPlus]
Labelled ligands	[http://www.guidetopharmacology.org/GRAC/LigandDisplayForward?ligandId=1002 (Antagonist) (p*K*_d_ 9.6–9.8) [http://www.ncbi.nlm.nih.gov/pubmed/9489609?dopt=AbstractPlus], [http://www.guidetopharmacology.org/GRAC/LigandDisplayForward?ligandId=3479 (Antagonist) (p*K*_d_ 9.2), [http://www.guidetopharmacology.org/GRAC/LigandDisplayForward?ligandId=1001 (Antagonist) (p*K*_d_ 9–9.1) [http://www.ncbi.nlm.nih.gov/pubmed/8012722?dopt=AbstractPlus], [http://www.guidetopharmacology.org/GRAC/LigandDisplayForward?ligandId=1003 (Antagonist) (p*K*_d_ 8.5) [http://www.ncbi.nlm.nih.gov/pubmed/7768260?dopt=AbstractPlus]	[http://www.guidetopharmacology.org/GRAC/LigandDisplayForward?ligandId=1007 (Agonist) [http://www.ncbi.nlm.nih.gov/pubmed/8301559?dopt=AbstractPlus], [http://www.guidetopharmacology.org/GRAC/LigandDisplayForward?ligandId=1005 (Agonist) [http://www.ncbi.nlm.nih.gov/pubmed/8587429?dopt=AbstractPlus, http://www.ncbi.nlm.nih.gov/pubmed/1472961?dopt=AbstractPlus, http://www.ncbi.nlm.nih.gov/pubmed/7881728?dopt=AbstractPlus], [http://www.guidetopharmacology.org/GRAC/LigandDisplayForward?ligandId=3760]http://www.guidetopharmacology.org/GRAC/LigandDisplayForward?ligandId=3760 (Agonist) [http://www.ncbi.nlm.nih.gov/pubmed/1472961?dopt=AbstractPlus]

### Comments

Splice variants of the ET_A_ receptor have been identified in rat pituitary cells; one of these, ET_A_R‐C13, appeared to show loss of function with comparable plasma membrane expression to wild type receptor [http://www.ncbi.nlm.nih.gov/pubmed/17312275?dopt=AbstractPlus]. Subtypes of the ET_B_ receptor have been proposed, although gene disruption studies in mice suggest that only a single gene product exists [http://www.ncbi.nlm.nih.gov/pubmed/9113361?dopt=AbstractPlus]. Crystal structures of the ETB receptor bound to the antagonist http://www.guidetopharmacology.org/GRAC/LigandDisplayForward?ligandId=3494 and ET_B_ selective analogue http://www.guidetopharmacology.org/GRAC/LigandDisplayForward?ligandId=9651 [http://www.ncbi.nlm.nih.gov/pubmed/28805809?dopt=AbstractPlus] and selective ET_B_ agonists http://www.guidetopharmacology.org/GRAC/LigandDisplayForward?ligandId=1004 (https://www.genenames.org/data/gene‐symbol‐report/#!/hgnc_id/HGNC:3178, http://www.uniprot.org/uniprot/P14138) and http://www.guidetopharmacology.org/GRAC/LigandDisplayForward?ligandId=3886 [http://www.ncbi.nlm.nih.gov/pubmed/30413709?dopt=AbstractPlus] have been reported.

### Further reading on Endothelin receptors

Clozel M *et al*. (2013) Endothelin receptor antagonists. *Handb Exp Pharmacol* **218**: 199‐227 https://www.ncbi.nlm.nih.gov/pubmed/24092342?dopt=AbstractPlus

Davenport AP. (2002) International Union of Pharmacology. XXIX. Update on endothelin receptor nomenclature. *Pharmacol. Rev.* **54**: 219‐26 https://www.ncbi.nlm.nih.gov/pubmed/12037137?dopt=AbstractPlus

Davenport AP *et al*. (2016) Endothelin. *Pharmacol. Rev.* **68**: 357‐418 https://www.ncbi.nlm.nih.gov/pubmed/26956245?dopt=AbstractPlus

Davenport AP *et al*. (2018) New drugs and emerging therapeutic targets in the endothelin signaling pathway and prospects for personalized precision medicine. *Physiol Res* **67**: S37‐S54 https://www.ncbi.nlm.nih.gov/pubmed/29947527?dopt=AbstractPlus

Maguire JJ *et al*. (2014) Endothelin@25 ‐ new agonists, antagonists, inhibitors and emerging research frontiers: IUPHAR Review 12. *Br. J. Pharmacol.* **171**: 5555‐72 https://www.ncbi.nlm.nih.gov/pubmed/25131455?dopt=AbstractPlus

## http://www.guidetopharmacology.org/GRAC/FamilyDisplayForward?familyId=22

### Overview

The G protein‐coupled estrogen receptor (GPER, **nomenclature as agreed by the NC‐IUPHAR Subcommittee on the G protein‐coupled estrogen receptor** [http://www.ncbi.nlm.nih.gov/pubmed/26023144?dopt=AbstractPlus]) was identified following observations of estrogen‐evoked http://www.guidetopharmacology.org/GRAC/LigandDisplayForward?ligandId=2352 signalling in breast cancer cells [http://www.ncbi.nlm.nih.gov/pubmed/8078914?dopt=AbstractPlus], which mirrored the differential expression of an orphan 7‐transmembrane receptor GPR30 [http://www.ncbi.nlm.nih.gov/pubmed/9367686?dopt=AbstractPlus]. There are observations of both cell‐surface and intracellular expression of the GPER receptor [http://www.ncbi.nlm.nih.gov/pubmed/15705806?dopt=AbstractPlus, http://www.ncbi.nlm.nih.gov/pubmed/15539556?dopt=AbstractPlus]. Selective agonist/ antagonists for GPER have been characterized [http://www.ncbi.nlm.nih.gov/pubmed/26023144?dopt=AbstractPlus]. Antagonists of the nuclear estrogen receptor, such as http://www.guidetopharmacology.org/GRAC/LigandDisplayForward?ligandId=1015 [http://www.ncbi.nlm.nih.gov/pubmed/11043579?dopt=AbstractPlus], http://www.guidetopharmacology.org/GRAC/LigandDisplayForward?ligandId=1016 [http://www.ncbi.nlm.nih.gov/pubmed/15705806?dopt=AbstractPlus, http://www.ncbi.nlm.nih.gov/pubmed/15539556?dopt=AbstractPlus] and http://www.guidetopharmacology.org/GRAC/LigandDisplayForward?ligandId=2820 [http://www.ncbi.nlm.nih.gov/pubmed/24379833?dopt=AbstractPlus], as well as the flavonoid ’phytoestrogens’ http://www.guidetopharmacology.org/GRAC/LigandDisplayForward?ligandId=2826 and http://www.guidetopharmacology.org/GRAC/LigandDisplayForward?ligandId=5346 [http://www.ncbi.nlm.nih.gov/pubmed/15090535?dopt=AbstractPlus], are agonists of GPER. A complete review of GPER pharmacology has been recently published [http://www.ncbi.nlm.nih.gov/pubmed/26023144?dopt=AbstractPlus]. The roles of GPER in physiological systems throughout the body (cardiovascular, metabolic, endocrine, immune, reproductive) and in cancer have also been reviewed [http://www.ncbi.nlm.nih.gov/pubmed/28595943?dopt=AbstractPlus, http://www.ncbi.nlm.nih.gov/pubmed/28249728?dopt=AbstractPlus, http://www.ncbi.nlm.nih.gov/pubmed/28343901?dopt=AbstractPlus, http://www.ncbi.nlm.nih.gov/pubmed/26023144?dopt=AbstractPlus, http://www.ncbi.nlm.nih.gov/pubmed/26189910?dopt=AbstractPlus].

**Table:** 

Nomenclature	http://www.guidetopharmacology.org/GRAC/ObjectDisplayForward?objectId=221
HGNC, UniProt	https://www.genenames.org/data/gene‐symbol‐report/#!/hgnc_id/HGNC:4485, http://www.uniprot.org/uniprot/Q99527
Agonists	http://www.guidetopharmacology.org/GRAC/LigandDisplayForward?ligandId=1015 [http://www.ncbi.nlm.nih.gov/pubmed/15539556?dopt=AbstractPlus], http://www.guidetopharmacology.org/GRAC/LigandDisplayForward?ligandId=2820 [http://www.ncbi.nlm.nih.gov/pubmed/24379833?dopt=AbstractPlus], http://www.guidetopharmacology.org/GRAC/LigandDisplayForward?ligandId=2817 [http://www.ncbi.nlm.nih.gov/pubmed/15705806?dopt=AbstractPlus]
Selective agonists	http://www.guidetopharmacology.org/GRAC/LigandDisplayForward?ligandId=1014 [http://www.ncbi.nlm.nih.gov/pubmed/16520733?dopt=AbstractPlus]
Selective antagonists	http://www.guidetopharmacology.org/GRAC/LigandDisplayForward?ligandId=6480 (pIC_50_ 6.8–6.9) [http://www.ncbi.nlm.nih.gov/pubmed/21782022?dopt=AbstractPlus], http://www.guidetopharmacology.org/GRAC/LigandDisplayForward?ligandId=3320 (pIC_50_ 6.7) [http://www.ncbi.nlm.nih.gov/pubmed/19430488?dopt=AbstractPlus]
Labelled ligands	[http://www.guidetopharmacology.org/GRAC/LigandDisplayForward?ligandId=1012 (Agonist) [http://www.ncbi.nlm.nih.gov/pubmed/15539556?dopt=AbstractPlus]

### Comments

Antagonists at the nuclear estrogen receptor, such as http://www.guidetopharmacology.org/GRAC/LigandDisplayForward?ligandId=1015, http://www.guidetopharmacology.org/GRAC/LigandDisplayForward?ligandId=1016 [http://www.ncbi.nlm.nih.gov/pubmed/11043579?dopt=AbstractPlus] and http://www.guidetopharmacology.org/GRAC/LigandDisplayForward?ligandId=2820 [http://www.ncbi.nlm.nih.gov/pubmed/24379833?dopt=AbstractPlus], as well as the flavonoid ‘phytoestrogens’ http://www.guidetopharmacology.org/GRAC/LigandDisplayForward?ligandId=2826 and http://www.guidetopharmacology.org/GRAC/LigandDisplayForward?ligandId=5346 [http://www.ncbi.nlm.nih.gov/pubmed/15090535?dopt=AbstractPlus], are agonists at GPER receptors. A complete review of GPER pharmacology has been recently published [http://www.ncbi.nlm.nih.gov/pubmed/26023144?dopt=AbstractPlus].

### Further reading on G protein‐coupled estrogen receptor

Barton M *et al*. (2018) Twenty years of the G protein‐coupled estrogen receptor GPER: Historical and personal perspectives. *J. Steroid Biochem. Mol. Biol.* **176**: 4‐15 https://www.ncbi.nlm.nih.gov/pubmed/28347854?dopt=AbstractPlus

Gaudet HM *et al*. (2015) The G‐protein coupled estrogen receptor, GPER: The inside and inside‐out story. *Mol. Cell. Endocrinol.* **418 Pt 3**: 207‐19 https://www.ncbi.nlm.nih.gov/pubmed/26190834?dopt=AbstractPlus

Prossnitz ER *et al*. (2015) International Union of Basic and Clinical Pharmacology. XCVII. G Protein‐Coupled Estrogen Receptor and Its Pharmacologic Modulators. *Pharmacol. Rev.* **67**: 505‐40 https://www.ncbi.nlm.nih.gov/pubmed/26023144?dopt=AbstractPlus

Prossnitz ER *et al*. (2015) What have we learned about GPER function in physiology and disease from knockout mice? *J. Steroid Biochem. Mol. Biol.* **153**: 114‐26 https://www.ncbi.nlm.nih.gov/pubmed/26189910?dopt=AbstractPlus

## http://www.guidetopharmacology.org/GRAC/FamilyDisplayForward?familyId=23

### Overview

The http://www.ensembl.org/Homo_sapiens/Gene/Family/Genes?family=ENSFM00510000502765 (**nomenclature agreed by the NC‐IUPHAR Subcommittee on the formylpeptide receptor family** [http://www.ncbi.nlm.nih.gov/pubmed/19498085?dopt=AbstractPlus]) respond to exogenous ligands such as the bacterial product http://www.guidetopharmacology.org/GRAC/LigandDisplayForward?ligandId=1022 (fMLP) and endogenous ligands such as http://www.guidetopharmacology.org/GRAC/LigandDisplayForward?ligandId=1031 (https://www.genenames.org/data/gene‐symbol‐report/#!/hgnc_id/HGNC:533, http://www.uniprot.org/uniprot/P04083), http://www.guidetopharmacology.org/GRAC/LigandDisplayForward?ligandId=3570 (https://www.genenames.org/data/gene‐symbol‐report/#!/hgnc_id/HGNC:2532, http://www.uniprot.org/uniprot/P08311), amyloid β42, serum amyloid A and http://www.guidetopharmacology.org/GRAC/LigandDisplayForward?ligandId=1026, derived from http://www.guidetopharmacology.org/GRAC/LigandDisplayForward?ligandId=5370 (https://www.genenames.org/data/gene‐symbol‐report/#!/hgnc_id/HGNC:4827, http://www.uniprot.org/uniprot/P68871).

**Table:** 

Nomenclature	http://www.guidetopharmacology.org/GRAC/ObjectDisplayForward?objectId=222	http://www.guidetopharmacology.org/GRAC/ObjectDisplayForward?objectId=223	http://www.guidetopharmacology.org/GRAC/ObjectDisplayForward?objectId=224
HGNC, UniProt	https://www.genenames.org/data/gene‐symbol‐report/#!/hgnc_id/HGNC:3826, http://www.uniprot.org/uniprot/P21462	https://www.genenames.org/data/gene‐symbol‐report/#!/hgnc_id/HGNC:3827, http://www.uniprot.org/uniprot/P25090	https://www.genenames.org/data/gene‐symbol‐report/#!/hgnc_id/HGNC:3828, http://www.uniprot.org/uniprot/P25089
Potency order of endogenous ligands	http://www.guidetopharmacology.org/GRAC/LigandDisplayForward?ligandId=1022 > http://www.guidetopharmacology.org/GRAC/LigandDisplayForward?ligandId=3570 (https://www.genenames.org/data/gene‐symbol‐report/#!/hgnc_id/HGNC:2532, http://www.uniprot.org/uniprot/P08311) > http://www.guidetopharmacology.org/GRAC/LigandDisplayForward?ligandId=1031 (https://www.genenames.org/data/gene‐symbol‐report/#!/hgnc_id/HGNC:533, http://www.uniprot.org/uniprot/P04083) [http://www.ncbi.nlm.nih.gov/pubmed/12401407?dopt=AbstractPlus, http://www.ncbi.nlm.nih.gov/pubmed/15210802?dopt=AbstractPlus]	http://www.guidetopharmacology.org/GRAC/LigandDisplayForward?ligandId=1034 = http://www.guidetopharmacology.org/GRAC/LigandDisplayForward?ligandId=3933 = http://www.guidetopharmacology.org/GRAC/LigandDisplayForward?ligandId=3383 = http://www.guidetopharmacology.org/GRAC/LigandDisplayForward?ligandId=3934 > http://www.guidetopharmacology.org/GRAC/LigandDisplayForward?ligandId=3354 = http://www.guidetopharmacology.org/GRAC/LigandDisplayForward?ligandId=3353 http://www.guidetopharmacology.org/GRAC/LigandDisplayForward?ligandId=3402 http://www.guidetopharmacology.org/GRAC/LigandDisplayForward?ligandId=1022 [http://www.ncbi.nlm.nih.gov/pubmed/10393980?dopt=AbstractPlus, http://www.ncbi.nlm.nih.gov/pubmed/8006586?dopt=AbstractPlus, http://www.ncbi.nlm.nih.gov/pubmed/8527441?dopt=AbstractPlus, http://www.ncbi.nlm.nih.gov/pubmed/11141472?dopt=AbstractPlus, http://www.ncbi.nlm.nih.gov/pubmed/9151906?dopt=AbstractPlus]	–
Endogenous agonists	–	http://www.guidetopharmacology.org/GRAC/LigandDisplayForward?ligandId=1034 [http://www.ncbi.nlm.nih.gov/pubmed/20080636?dopt=AbstractPlus], http://www.guidetopharmacology.org/GRAC/LigandDisplayForward?ligandId=3934 [http://www.ncbi.nlm.nih.gov/pubmed/20080636?dopt=AbstractPlus], http://www.guidetopharmacology.org/GRAC/LigandDisplayForward?ligandId=6239 [http://www.ncbi.nlm.nih.gov/pubmed/22449948?dopt=AbstractPlus], http://www.guidetopharmacology.org/GRAC/LigandDisplayForward?ligandId=3933	http://www.guidetopharmacology.org/GRAC/LigandDisplayForward?ligandId=3935 (https://www.genenames.org/data/gene‐symbol‐report/#!/hgnc_id/HGNC:17176, http://www.uniprot.org/uniprot/Q9NRV9) [http://www.ncbi.nlm.nih.gov/pubmed/15623572?dopt=AbstractPlus]
Agonists	http://www.guidetopharmacology.org/GRAC/LigandDisplayForward?ligandId=1022 [http://www.ncbi.nlm.nih.gov/pubmed/7387981?dopt=AbstractPlus, http://www.ncbi.nlm.nih.gov/pubmed/1262785?dopt=AbstractPlus]	–	–
Selective agonists	–	http://www.guidetopharmacology.org/GRAC/LigandDisplayForward?ligandId=3383 [http://www.ncbi.nlm.nih.gov/pubmed/15056011?dopt=AbstractPlus]	–
Endogenous antagonists	http://www.guidetopharmacology.org/GRAC/LigandDisplayForward?ligandId=1026 (pIC_50_ 4.3) [http://www.ncbi.nlm.nih.gov/pubmed/11714831?dopt=AbstractPlus, http://www.ncbi.nlm.nih.gov/pubmed/1373134?dopt=AbstractPlus]	–	–
Antagonists	http://www.guidetopharmacology.org/GRAC/LigandDisplayForward?ligandId=1030 (p*K*_i_ 6–6.5) [http://www.ncbi.nlm.nih.gov/pubmed/8387097?dopt=AbstractPlus]	–	–
Selective antagonists	http://www.guidetopharmacology.org/GRAC/LigandDisplayForward?ligandId=1025 (p*K*_i_ 6.1–7.1) [http://www.ncbi.nlm.nih.gov/pubmed/8387097?dopt=AbstractPlus, http://www.ncbi.nlm.nih.gov/pubmed/17082621?dopt=AbstractPlus]	http://www.guidetopharmacology.org/GRAC/LigandDisplayForward?ligandId=1040 (pIC_50_ 6.6) [http://www.ncbi.nlm.nih.gov/pubmed/15210823?dopt=AbstractPlus], http://www.guidetopharmacology.org/GRAC/LigandDisplayForward?ligandId=1030 (pIC_50_ 4.3–6) [http://www.ncbi.nlm.nih.gov/pubmed/6280748?dopt=AbstractPlus, http://www.ncbi.nlm.nih.gov/pubmed/17687636?dopt=AbstractPlus, http://www.ncbi.nlm.nih.gov/pubmed/10882119?dopt=AbstractPlus]	–
Labelled ligands	[http://www.guidetopharmacology.org/GRAC/LigandDisplayForward?ligandId=1018 (Agonist) [http://www.ncbi.nlm.nih.gov/pubmed/6285921?dopt=AbstractPlus]	[http://www.guidetopharmacology.org/GRAC/LigandDisplayForward?ligandId=3411 (Agonist) [http://www.ncbi.nlm.nih.gov/pubmed/8006586?dopt=AbstractPlus, http://www.ncbi.nlm.nih.gov/pubmed/1322894?dopt=AbstractPlus]	–
Comments	A FITC‐conjugated fMLP analogue has been used for binding to the mouse recombinant receptor [http://www.ncbi.nlm.nih.gov/pubmed/23160941?dopt=AbstractPlus].	–	–

### Comments

Note that the data for FPR2/ALX are also reproduced on the http://www.guidetopharmacology.org/GRAC/FamilyDisplayForward?familyId=35 receptor page.

### Further reading on Formylpeptide receptors

Dorward DA *et al*. (2015) The Role of Formylated Peptides and Formyl Peptide Receptor 1 in Governing Neutrophil Function during Acute Inflammation. *Am. J. Pathol.* **185**: 1172‐1184 https://www.ncbi.nlm.nih.gov/pubmed/25791526?dopt=AbstractPlus

Dufton N *et al*. (2010) Therapeutic anti‐inflammatory potential of formyl‐peptide receptor agonists. *Pharmacol. Ther.* **127**: 175‐88 https://www.ncbi.nlm.nih.gov/pubmed/20546777?dopt=AbstractPlus

Liu M *et al*. (2012) G protein‐coupled receptor FPR1 as a pharmacologic target in inflammation and human glioblastoma. *Int. Immunopharmacol.* **14**: 283‐8 https://www.ncbi.nlm.nih.gov/pubmed/22863814?dopt=AbstractPlus

Rabiet MJ *et al*. (2011) N‐formyl peptide receptor 3 (FPR3) departs from the homologous FPR2/ALX receptor with regard to the major processes governing chemoattractant receptor regulation, expression at the cell surface, and phosphorylation. *J. Biol. Chem.* **286**: 26718‐31 https://www.ncbi.nlm.nih.gov/pubmed/21543323?dopt=AbstractPlus

Yazid S *et al*. (2012) Anti‐inflammatory drugs, eicosanoids and the annexin A1/FPR2 anti‐inflammatory system. *Prostaglandins Other Lipid Mediat.* **98**: 94‐100 https://www.ncbi.nlm.nih.gov/pubmed/22123264?dopt=AbstractPlus

Ye RD *et al*. (2009) International Union of Basic and Clinical Pharmacology. LXXIII. Nomenclature for the formyl peptide receptor (FPR) family. *Pharmacol. Rev.* **61**: 119‐61 https://www.ncbi.nlm.nih.gov/pubmed/19498085?dopt=AbstractPlus

## http://www.guidetopharmacology.org/GRAC/FamilyDisplayForward?familyId=24

### Overview

Free fatty acid receptors (FFA, **nomenclature as agreed by the NC‐IUPHAR Subcommittee on free fatty acid receptors** [http://www.ncbi.nlm.nih.gov/pubmed/23686350?dopt=AbstractPlus, http://www.ncbi.nlm.nih.gov/pubmed/19047536?dopt=AbstractPlus]) are activated by free fatty acids. Long‐chain saturated and unsaturated fatty acids (including C14.0 (http://www.guidetopharmacology.org/GRAC/LigandDisplayForward?ligandId=2806), C16:0 (http://www.guidetopharmacology.org/GRAC/LigandDisplayForward?ligandId=1055), C18:1 (http://www.guidetopharmacology.org/GRAC/LigandDisplayForward?ligandId=1054), C18:2 (http://www.guidetopharmacology.org/GRAC/LigandDisplayForward?ligandId=1052), C18:3, (http://www.guidetopharmacology.org/GRAC/LigandDisplayForward?ligandId=1049), C20:4 (http://www.guidetopharmacology.org/GRAC/LigandDisplayForward?ligandId=2391), C20:5,n‐3 (http://www.guidetopharmacology.org/GRAC/LigandDisplayForward?ligandId=3362) and C22:6,n‐3 (http://www.guidetopharmacology.org/GRAC/LigandDisplayForward?ligandId=1051)) activate FFA1 [http://www.ncbi.nlm.nih.gov/pubmed/12496284?dopt=AbstractPlus, http://www.ncbi.nlm.nih.gov/pubmed/12629551?dopt=AbstractPlus, http://www.ncbi.nlm.nih.gov/pubmed/12565875?dopt=AbstractPlus] and FFA4 receptors [http://www.ncbi.nlm.nih.gov/pubmed/15619630?dopt=AbstractPlus, http://www.ncbi.nlm.nih.gov/pubmed/22343897?dopt=AbstractPlus, http://www.ncbi.nlm.nih.gov/pubmed/20813258?dopt=AbstractPlus], while short chain fatty acids (C2 (http://www.guidetopharmacology.org/GRAC/LigandDisplayForward?ligandId=1058), C3 (http://www.guidetopharmacology.org/GRAC/LigandDisplayForward?ligandId=1062), C4 (http://www.guidetopharmacology.org/GRAC/LigandDisplayForward?ligandId=1059) and C5 (http://www.guidetopharmacology.org/GRAC/LigandDisplayForward?ligandId=1061)) activate FFA2 [http://www.ncbi.nlm.nih.gov/pubmed/12496283?dopt=AbstractPlus, http://www.ncbi.nlm.nih.gov/pubmed/12711604?dopt=AbstractPlus, http://www.ncbi.nlm.nih.gov/pubmed/12684041?dopt=AbstractPlus] and FFA3 [http://www.ncbi.nlm.nih.gov/pubmed/12496283?dopt=AbstractPlus, http://www.ncbi.nlm.nih.gov/pubmed/12711604?dopt=AbstractPlus] receptors. The crystal structure for agonist bound FFA1 has been described [http://www.ncbi.nlm.nih.gov/pubmed/25043059?dopt=AbstractPlus].

**Table:** 

Nomenclature	http://www.guidetopharmacology.org/GRAC/ObjectDisplayForward?objectId=225	http://www.guidetopharmacology.org/GRAC/ObjectDisplayForward?objectId=226
HGNC, UniProt	https://www.genenames.org/data/gene‐symbol‐report/#!/hgnc_id/HGNC:4498, http://www.uniprot.org/uniprot/O14842	https://www.genenames.org/data/gene‐symbol‐report/#!/hgnc_id/HGNC:4501, http://www.uniprot.org/uniprot/O15552
Endogenous agonists	http://www.guidetopharmacology.org/GRAC/LigandDisplayForward?ligandId=1051 [http://www.ncbi.nlm.nih.gov/pubmed/12496284?dopt=AbstractPlus, http://www.ncbi.nlm.nih.gov/pubmed/12629551?dopt=AbstractPlus], http://www.guidetopharmacology.org/GRAC/LigandDisplayForward?ligandId=1049 [http://www.ncbi.nlm.nih.gov/pubmed/12496284?dopt=AbstractPlus, http://www.ncbi.nlm.nih.gov/pubmed/12629551?dopt=AbstractPlus, http://www.ncbi.nlm.nih.gov/pubmed/12565875?dopt=AbstractPlus], http://www.guidetopharmacology.org/GRAC/LigandDisplayForward?ligandId=1054 [http://www.ncbi.nlm.nih.gov/pubmed/12496284?dopt=AbstractPlus, http://www.ncbi.nlm.nih.gov/pubmed/12629551?dopt=AbstractPlus, http://www.ncbi.nlm.nih.gov/pubmed/12565875?dopt=AbstractPlus], http://www.guidetopharmacology.org/GRAC/LigandDisplayForward?ligandId=2806 [http://www.ncbi.nlm.nih.gov/pubmed/12496284?dopt=AbstractPlus, http://www.ncbi.nlm.nih.gov/pubmed/12629551?dopt=AbstractPlus, http://www.ncbi.nlm.nih.gov/pubmed/12565875?dopt=AbstractPlus]	http://www.guidetopharmacology.org/GRAC/LigandDisplayForward?ligandId=1062 [http://www.ncbi.nlm.nih.gov/pubmed/12496283?dopt=AbstractPlus, http://www.ncbi.nlm.nih.gov/pubmed/12711604?dopt=AbstractPlus, http://www.ncbi.nlm.nih.gov/pubmed/12684041?dopt=AbstractPlus, http://www.ncbi.nlm.nih.gov/pubmed/21220428?dopt=AbstractPlus], http://www.guidetopharmacology.org/GRAC/LigandDisplayForward?ligandId=1058 [http://www.ncbi.nlm.nih.gov/pubmed/12496283?dopt=AbstractPlus, http://www.ncbi.nlm.nih.gov/pubmed/12711604?dopt=AbstractPlus, http://www.ncbi.nlm.nih.gov/pubmed/12684041?dopt=AbstractPlus, http://www.ncbi.nlm.nih.gov/pubmed/21220428?dopt=AbstractPlus], http://www.guidetopharmacology.org/GRAC/LigandDisplayForward?ligandId=1059 [http://www.ncbi.nlm.nih.gov/pubmed/12496283?dopt=AbstractPlus, http://www.ncbi.nlm.nih.gov/pubmed/12711604?dopt=AbstractPlus, http://www.ncbi.nlm.nih.gov/pubmed/12684041?dopt=AbstractPlus, http://www.ncbi.nlm.nih.gov/pubmed/21220428?dopt=AbstractPlus], http://www.guidetopharmacology.org/GRAC/LigandDisplayForward?ligandId=6499 [http://www.ncbi.nlm.nih.gov/pubmed/21220428?dopt=AbstractPlus], http://www.guidetopharmacology.org/GRAC/LigandDisplayForward?ligandId=6500 [http://www.ncbi.nlm.nih.gov/pubmed/21220428?dopt=AbstractPlus]
Selective agonists	http://www.guidetopharmacology.org/GRAC/LigandDisplayForward?ligandId=6485 [http://www.ncbi.nlm.nih.gov/pubmed/22859723?dopt=AbstractPlus], http://www.guidetopharmacology.org/GRAC/LigandDisplayForward?ligandId=9148 [http://www.ncbi.nlm.nih.gov/pubmed/27074625?dopt=AbstractPlus], http://www.guidetopharmacology.org/GRAC/LigandDisplayForward?ligandId=6483 [http://www.ncbi.nlm.nih.gov/pubmed/23687558?dopt=AbstractPlus], http://www.guidetopharmacology.org/GRAC/LigandDisplayForward?ligandId=9149 [http://www.ncbi.nlm.nih.gov/pubmed/22724451?dopt=AbstractPlus], http://www.guidetopharmacology.org/GRAC/LigandDisplayForward?ligandId=1050 (Partial agonist) [http://www.ncbi.nlm.nih.gov/pubmed/16702987?dopt=AbstractPlus], http://www.guidetopharmacology.org/GRAC/LigandDisplayForward?ligandId=6484 [http://www.ncbi.nlm.nih.gov/pubmed/25787200?dopt=AbstractPlus, http://www.ncbi.nlm.nih.gov/pubmed/24900210?dopt=AbstractPlus, http://www.ncbi.nlm.nih.gov/pubmed/25043059?dopt=AbstractPlus, http://www.ncbi.nlm.nih.gov/pubmed/21752941?dopt=AbstractPlus]	http://www.guidetopharmacology.org/GRAC/LigandDisplayForward?ligandId=10107 [http://www.ncbi.nlm.nih.gov/pubmed/30247908?dopt=AbstractPlus]
Selective antagonists	http://www.guidetopharmacology.org/GRAC/LigandDisplayForward?ligandId=1057 (pIC_50_ 6) [http://www.ncbi.nlm.nih.gov/pubmed/16702987?dopt=AbstractPlus, http://www.ncbi.nlm.nih.gov/pubmed/17200419?dopt=AbstractPlus]	http://www.guidetopharmacology.org/GRAC/LigandDisplayForward?ligandId=8417 (pIC_50_ 8.1) [http://www.ncbi.nlm.nih.gov/pubmed/26852904?dopt=AbstractPlus, http://www.ncbi.nlm.nih.gov/pubmed/25380412?dopt=AbstractPlus], http://www.guidetopharmacology.org/GRAC/LigandDisplayForward?ligandId=6487 (pIC_50_ 6.5) [http://www.ncbi.nlm.nih.gov/pubmed/23066016?dopt=AbstractPlus]
Comments	A wide range of both saturated and unsaturated fatty acids containing from 6 to 22 carbons have been shown to act as agonists at FFA1 [http://www.ncbi.nlm.nih.gov/pubmed/12496284?dopt=AbstractPlus, http://www.ncbi.nlm.nih.gov/pubmed/12629551?dopt=AbstractPlus, http://www.ncbi.nlm.nih.gov/pubmed/12565875?dopt=AbstractPlus]. Antagonist GW1100 is also an oxytocin receptor antagonist [http://www.ncbi.nlm.nih.gov/pubmed/16702987?dopt=AbstractPlus]. Fasiglifam, TUG‐770 and GW9508 are approximately 100 fold selective for FFA1 over FFA4 [http://www.ncbi.nlm.nih.gov/pubmed/16702987?dopt=AbstractPlus, http://www.ncbi.nlm.nih.gov/pubmed/23687558?dopt=AbstractPlus, http://www.ncbi.nlm.nih.gov/pubmed/24900210?dopt=AbstractPlus]. AMG‐837 and the related analogue AM6331 have been suggested to have an allosteric mechanism of action at FFA1, with respect to the orthosteric fatty acid binding site [http://www.ncbi.nlm.nih.gov/pubmed/22859723?dopt=AbstractPlus, http://www.ncbi.nlm.nih.gov/pubmed/23403053?dopt=AbstractPlus].	–

**Table:** 

Nomenclature	http://www.guidetopharmacology.org/GRAC/ObjectDisplayForward?objectId=227	http://www.guidetopharmacology.org/GRAC/ObjectDisplayForward?objectId=127	http://www.guidetopharmacology.org/GRAC/ObjectDisplayForward?objectId=228
HGNC, UniProt	https://www.genenames.org/data/gene‐symbol‐report/#!/hgnc_id/HGNC:4499, http://www.uniprot.org/uniprot/O14843	https://www.genenames.org/data/gene‐symbol‐report/#!/hgnc_id/HGNC:19061, http://www.uniprot.org/uniprot/Q5NUL3	https://www.genenames.org/data/gene‐symbol‐report/#!/hgnc_id/HGNC:4500, http://www.uniprot.org/uniprot/O15529
Endogenous agonists	http://www.guidetopharmacology.org/GRAC/LigandDisplayForward?ligandId=1062 [http://www.ncbi.nlm.nih.gov/pubmed/12496283?dopt=AbstractPlus, http://www.ncbi.nlm.nih.gov/pubmed/12711604?dopt=AbstractPlus, http://www.ncbi.nlm.nih.gov/pubmed/21220428?dopt=AbstractPlus, http://www.ncbi.nlm.nih.gov/pubmed/14722361?dopt=AbstractPlus], http://www.guidetopharmacology.org/GRAC/LigandDisplayForward?ligandId=1059 [http://www.ncbi.nlm.nih.gov/pubmed/12496283?dopt=AbstractPlus, http://www.ncbi.nlm.nih.gov/pubmed/12711604?dopt=AbstractPlus, http://www.ncbi.nlm.nih.gov/pubmed/21220428?dopt=AbstractPlus, http://www.ncbi.nlm.nih.gov/pubmed/14722361?dopt=AbstractPlus], http://www.guidetopharmacology.org/GRAC/LigandDisplayForward?ligandId=6500 [http://www.ncbi.nlm.nih.gov/pubmed/21220428?dopt=AbstractPlus]	http://www.guidetopharmacology.org/GRAC/LigandDisplayForward?ligandId=1049 [http://www.ncbi.nlm.nih.gov/pubmed/22519963?dopt=AbstractPlus], http://www.guidetopharmacology.org/GRAC/LigandDisplayForward?ligandId=2806 [http://www.ncbi.nlm.nih.gov/pubmed/22282525?dopt=AbstractPlus], http://www.guidetopharmacology.org/GRAC/LigandDisplayForward?ligandId=1049 [http://www.ncbi.nlm.nih.gov/pubmed/18320172?dopt=AbstractPlus] – Rat, http://www.guidetopharmacology.org/GRAC/LigandDisplayForward?ligandId=1054 [http://www.ncbi.nlm.nih.gov/pubmed/22282525?dopt=AbstractPlus]	–
Agonists	http://www.guidetopharmacology.org/GRAC/LigandDisplayForward?ligandId=1058 [http://www.ncbi.nlm.nih.gov/pubmed/12496283?dopt=AbstractPlus, http://www.ncbi.nlm.nih.gov/pubmed/12711604?dopt=AbstractPlus, http://www.ncbi.nlm.nih.gov/pubmed/21220428?dopt=AbstractPlus, http://www.ncbi.nlm.nih.gov/pubmed/14722361?dopt=AbstractPlus]	–	–
Selective agonists	–	http://www.guidetopharmacology.org/GRAC/LigandDisplayForward?ligandId=8418 [http://www.ncbi.nlm.nih.gov/pubmed/24997608?dopt=AbstractPlus], http://www.guidetopharmacology.org/GRAC/LigandDisplayForward?ligandId=6490 [http://www.ncbi.nlm.nih.gov/pubmed/22519963?dopt=AbstractPlus], http://www.guidetopharmacology.org/GRAC/LigandDisplayForward?ligandId=5587 [http://www.ncbi.nlm.nih.gov/pubmed/19007110?dopt=AbstractPlus]	–
Comments	Beta‐hydroxybutyrate has been reported to antagonise FFA3 responses to short chain fatty acids [http://www.ncbi.nlm.nih.gov/pubmed/21518883?dopt=AbstractPlus]. A range of FFA3 selective molecules with agonist and antagonist properties, but which bind at sites distinct from the short chain fatty acid binding site (*i.e.* allosteric modulators), have been described [http://www.ncbi.nlm.nih.gov/pubmed/27385588?dopt=AbstractPlus, http://www.ncbi.nlm.nih.gov/pubmed/24870406?dopt=AbstractPlus, http://www.ncbi.nlm.nih.gov/pubmed/26808470?dopt=AbstractPlus].	A wide range of both saturated and unsaturated fatty acids containing from 6 to 22 carbons have been shown to act as agonists at FFA4 [http://www.ncbi.nlm.nih.gov/pubmed/25916176?dopt=AbstractPlus] with a small subset listed above. Compound A [PMID 24997608] exhibits more than 1000 fold selectivity [http://www.ncbi.nlm.nih.gov/pubmed/24997608?dopt=AbstractPlus], and TUG‐891 50‐1000 fold selectivity for FFA4 over FFA1 [http://www.ncbi.nlm.nih.gov/pubmed/22519963?dopt=AbstractPlus], dependent on the assay. NGC21 exhibits approximately 15 fold selectivity for FFA4 over FFA1 [http://www.ncbi.nlm.nih.gov/pubmed/20685848?dopt=AbstractPlus].	–

### Comments

Short (361 amino acids) and long (377 amino acids) splice variants of human FFA4 have been reported [http://www.ncbi.nlm.nih.gov/pubmed/19723586?dopt=AbstractPlus], which differ by a 16 amino acid insertion in intracellular loop 3, and exhibit differences in intracellular signalling properties in recombinant systems [http://www.ncbi.nlm.nih.gov/pubmed/22282525?dopt=AbstractPlus]. The long FFA4 splice variant has not been identified in other primates or rodents to date [http://www.ncbi.nlm.nih.gov/pubmed/15619630?dopt=AbstractPlus, http://www.ncbi.nlm.nih.gov/pubmed/19723586?dopt=AbstractPlus].

https://www.genenames.org/data/gene‐symbol‐report/%23!/hgnc_id/HGNC:4500 was originally described as a pseudogene within the family (http://www.ensembl.org/Homo_sapiens/Gene/Family/Genes?family=ENSFM00250000002583), but the discovery of several polymorphisms suggests that some versions of GPR42 may be functional [http://www.ncbi.nlm.nih.gov/pubmed/19630535?dopt=AbstractPlus]. https://www.genenames.org/data/gene‐symbol‐report/%23!/hgnc_id/HGNC:4535 is a structurally‐unrelated G protein‐coupled receptor which has been found to respond to medium chain fatty acids [http://www.ncbi.nlm.nih.gov/pubmed/16966319?dopt=AbstractPlus].

### Further reading on Free fatty acid receptors

Bolognini D *et al*. (2016) The Pharmacology and Function of Receptors for Short‐Chain Fatty Acids. *Mol. Pharmacol.* **89**: 388‐98 https://www.ncbi.nlm.nih.gov/pubmed/26719580?dopt=AbstractPlus

Mancini AD *et al*. (2013) The fatty acid receptor FFA1/GPR40 a decade later: how much do we know? *Trends Endocrinol. Metab.* **24**: 398‐407 https://www.ncbi.nlm.nih.gov/pubmed/23631851?dopt=AbstractPlus

Milligan G *et al*. (2017) Complex Pharmacology of Free Fatty Acid Receptors. *Chem. Rev.* **117**: 67‐110 https://www.ncbi.nlm.nih.gov/pubmed/27299848?dopt=AbstractPlus

Moniri NH. (2016) Free‐fatty acid receptor‐4 (GPR120): Cellular and molecular function and its role in metabolic disorders. *Biochem. Pharmacol.* **110‐111**: 1‐15 https://www.ncbi.nlm.nih.gov/pubmed/26827942?dopt=AbstractPlus

Stoddart LA *et al*. (2008) International Union of Pharmacology. LXXI. Free fatty acid receptors FFA1, ‐2, and ‐3: pharmacology and pathophysiological functions. *Pharmacol. Rev.* **60**: 405‐17 [https://www.ncbi.nlm.nih.gov/pubmed/19047536?dopt=AbstractPlus]

Watterson KR *et al*. (2014) Treatment of type 2 diabetes by free Fatty Acid receptor agonists. *Front Endocrinol (Lausanne)* **5**: 137 [https://www.ncbi.nlm.nih.gov/pubmed/25221541?dopt=AbstractPlus]

## http://www.guidetopharmacology.org/GRAC/FamilyDisplayForward?familyId=26

### Overview

Functional GABA_B_ receptors (**nomenclature as agreed by the NC‐IUPHAR Subcommittee on GABA_B_ receptors** [http://www.ncbi.nlm.nih.gov/pubmed/12037141?dopt=AbstractPlus, http://www.ncbi.nlm.nih.gov/pubmed/17329545?dopt=AbstractPlus]) are formed from the heterodimerization of two similar 7TM subunits termed GABA_B1_ and GABA_B2_ [http://www.ncbi.nlm.nih.gov/pubmed/12037141?dopt=AbstractPlus, http://www.ncbi.nlm.nih.gov/pubmed/17499108?dopt=AbstractPlus, http://www.ncbi.nlm.nih.gov/pubmed/15451400?dopt=AbstractPlus, http://www.ncbi.nlm.nih.gov/pubmed/17329545?dopt=AbstractPlus, http://www.ncbi.nlm.nih.gov/pubmed/17433877?dopt=AbstractPlus]. GABAB receptors are widespread in the CNS and regulate both pre‐ and postsynaptic activity. The GABA_B1_ subunit, when expressed alone, binds both antagonists and agonists, but the affinity of the latter is generally 10‐100fold less than for the native receptor. Co‐expression of GABA_B1_ and GABA_B2_ subunits allows transport of GABA_B1_ to the cell surface and generates a functional receptor that can couple to signal transduction pathways such as high‐voltage‐activated Ca^2+^ channels (Ca_v_2.1, Ca_v_2.2), or inwardly rectifying potassium channels (Kir3) [http://www.ncbi.nlm.nih.gov/pubmed/15269338?dopt=AbstractPlus, http://www.ncbi.nlm.nih.gov/pubmed/12037141?dopt=AbstractPlus, http://www.ncbi.nlm.nih.gov/pubmed/10604925?dopt=AbstractPlus]. The GABA_B1_ subunit harbours the GABA (orthosteric)‐binding site within an extracellular domain (ECD) venus flytrap module (VTM), whereas the GABA_B2_ subunit mediates G protein‐coupled signalling [http://www.ncbi.nlm.nih.gov/pubmed/12037141?dopt=AbstractPlus, http://www.ncbi.nlm.nih.gov/pubmed/24305054?dopt=AbstractPlus, http://www.ncbi.nlm.nih.gov/pubmed/22660477?dopt=AbstractPlus, http://www.ncbi.nlm.nih.gov/pubmed/15451400?dopt=AbstractPlus]. The two subunits interact by direct allosteric coupling [http://www.ncbi.nlm.nih.gov/pubmed/21063387?dopt=AbstractPlus], such that GABA_B2_ increases the affinity of GABA_B1_ for agonists and reciprocally GABA_B1_ facilitates the coupling of GABA_B2_ to G proteins [http://www.ncbi.nlm.nih.gov/pubmed/24305054?dopt=AbstractPlus, http://www.ncbi.nlm.nih.gov/pubmed/15922585?dopt=AbstractPlus, http://www.ncbi.nlm.nih.gov/pubmed/15451400?dopt=AbstractPlus]. GABA_B1_ and GABA_B2_ subunits assemble in a 1:1 stoichiometry by means of a coiled‐coil interaction between α‐helices within their carboxy‐termini that masks an endoplasmic reticulum retention motif (RXRR) within the GABA_B1_ subunit but other domains of the proteins also contribute to their heteromerization [http://www.ncbi.nlm.nih.gov/pubmed/15269338?dopt=AbstractPlus, http://www.ncbi.nlm.nih.gov/pubmed/24778228?dopt=AbstractPlus, http://www.ncbi.nlm.nih.gov/pubmed/15451400?dopt=AbstractPlus]. Recent evidence indicates that higher order assemblies of GABA_B_ receptor comprising dimers of heterodimers occur in recombinant expression systems and *in vivo* and that such complexes exhibit negative functional cooperativity between heterodimers [http://www.ncbi.nlm.nih.gov/pubmed/21552208?dopt=AbstractPlus, http://www.ncbi.nlm.nih.gov/pubmed/19723778?dopt=AbstractPlus]. Adding further complexity, KCTD (potassium channel tetramerization proteins) 8, 12, 12b and 16 associate as tetramers with the carboxy terminus of the GABA_B2_ subunit to impart altered signalling kinetics and agonist potency to the receptor complex [http://www.ncbi.nlm.nih.gov/pubmed/20406808?dopt=AbstractPlus, http://www.ncbi.nlm.nih.gov/pubmed/20400944?dopt=AbstractPlus, http://www.ncbi.nlm.nih.gov/pubmed/24836506?dopt=AbstractPlus] and are reviewed by [http://www.ncbi.nlm.nih.gov/pubmed/20655485?dopt=AbstractPlus]. The molecular complexity of GABAB receptors is further increased through association with trafficking and effector proteins [Schwenk et al., 2016, Nature Neuroscience 19(2): 233‐42] and reviewed by [http://www.ncbi.nlm.nih.gov/pubmed/27905440?dopt=AbstractPlus]. Four isoforms of the human GABA_B1_ subunit have been cloned. The predominant GABA_B1a_ and GABA_B1b_ isoforms, which are most prevalent in neonatal and adult brain tissue respectively, differ in their ECD sequences as a result of the use of alternative transcription initiation sites. GABA_B1a_‐containing heterodimers localise todistal axonsand mediate inhibition of glutamate release in the CA3‐CA1 terminals, and GABA release onto the layer 5 pyramidal neurons, whereas GABA_B1b_‐containing receptors occur within dendritic spines and mediate slow postsynaptic inhibition [http://www.ncbi.nlm.nih.gov/pubmed/16701210?dopt=AbstractPlus, http://www.ncbi.nlm.nih.gov/pubmed/16701209?dopt=AbstractPlus]. Only the 1a and 1b variants are identified as components of native receptors [http://www.ncbi.nlm.nih.gov/pubmed/12037141?dopt=AbstractPlus]. Additional GABA_B1_ subunit isoforms have been described in rodents and humans [http://www.ncbi.nlm.nih.gov/pubmed/21124972?dopt=AbstractPlus] and reviewed by [http://www.ncbi.nlm.nih.gov/pubmed/15269338?dopt=AbstractPlus].

**Table:** 

Nomenclature	http://www.guidetopharmacology.org/GRAC/ObjectDisplayForward?objectId=242
Subunits	http://www.guidetopharmacology.org/GRAC/ObjectDisplayForward?objectId=240, http://www.guidetopharmacology.org/GRAC/ObjectDisplayForward?objectId=241,http://www.guidetopharmacology.org/GRAC/ObjectDisplayForward?objectId=1919 (Accessory protein), http://www.guidetopharmacology.org/GRAC/ObjectDisplayForward?objectId=1920 (Accessory protein), http://www.guidetopharmacology.org/GRAC/ObjectDisplayForward?objectId=1918 (Accessory protein), http://www.guidetopharmacology.org/GRAC/ObjectDisplayForward?objectId=1917 (Accessory protein)
Agonists	http://www.guidetopharmacology.org/GRAC/LigandDisplayForward?ligandId=1082 [635] – Rat, (‐)‐http://www.guidetopharmacology.org/GRAC/LigandDisplayForward?ligandId=1064 [635] – Rat, http://www.guidetopharmacology.org/GRAC/LigandDisplayForward?ligandId=1081 [http://www.ncbi.nlm.nih.gov/pubmed/12663046?dopt=AbstractPlus], http://www.guidetopharmacology.org/GRAC/LigandDisplayForward?ligandId=1084 [http://www.ncbi.nlm.nih.gov/pubmed/12663046?dopt=AbstractPlus, http://www.ncbi.nlm.nih.gov/pubmed/11082110?dopt=AbstractPlus], http://www.guidetopharmacology.org/GRAC/LigandDisplayForward?ligandId=1080 [http://www.ncbi.nlm.nih.gov/pubmed/11082110?dopt=AbstractPlus]
Antagonists	http://www.guidetopharmacology.org/GRAC/LigandDisplayForward?ligandId=1072 (p*K*_i_ 8.5–8.9) [http://www.ncbi.nlm.nih.gov/pubmed/12663046?dopt=AbstractPlus, http://www.ncbi.nlm.nih.gov/pubmed/11082110?dopt=AbstractPlus], http://www.guidetopharmacology.org/GRAC/LigandDisplayForward?ligandId=1088 (p*K*_i_ 7.8) [http://www.ncbi.nlm.nih.gov/pubmed/11082110?dopt=AbstractPlus], http://www.guidetopharmacology.org/GRAC/LigandDisplayForward?ligandId=1075 (p*K*_i_ 5.5–6) [http://www.ncbi.nlm.nih.gov/pubmed/12663046?dopt=AbstractPlus, http://www.ncbi.nlm.nih.gov/pubmed/11082110?dopt=AbstractPlus], http://www.guidetopharmacology.org/GRAC/LigandDisplayForward?ligandId=1069 (p*K*_i_ 4.4) [http://www.ncbi.nlm.nih.gov/pubmed/11082110?dopt=AbstractPlus], http://www.guidetopharmacology.org/GRAC/LigandDisplayForward?ligandId=1068 (pIC_50_ 4.1) [http://www.ncbi.nlm.nih.gov/pubmed/9069281?dopt=AbstractPlus] – Rat
Allosteric modulators	http://www.guidetopharmacology.org/GRAC/LigandDisplayForward?ligandId=10267 (Positive) (pEC_50_ 6.6) [http://www.ncbi.nlm.nih.gov/pubmed/18536733?dopt=AbstractPlus], http://www.guidetopharmacology.org/GRAC/LigandDisplayForward?ligandId=5446 (Positive) (p*K*_B_ 4.7) [http://www.ncbi.nlm.nih.gov/pubmed/29514854?dopt=AbstractPlus, http://www.ncbi.nlm.nih.gov/pubmed/12954816?dopt=AbstractPlus], http://www.guidetopharmacology.org/GRAC/LigandDisplayForward?ligandId=8539 (Negative) (pIC_50_ 4.4) [http://www.ncbi.nlm.nih.gov/pubmed/25050158?dopt=AbstractPlus], http://www.guidetopharmacology.org/GRAC/LigandDisplayForward?ligandId=1079 (Positive) [http://www.ncbi.nlm.nih.gov/pubmed/11641424?dopt=AbstractPlus]
Labelled ligands	[http://www.guidetopharmacology.org/GRAC/LigandDisplayForward?ligandId=1090 (Antagonist) (p*K*_i_ 9.1) [http://www.ncbi.nlm.nih.gov/pubmed/9872315?dopt=AbstractPlus] – Rat, http://www.guidetopharmacology.org/GRAC/LigandDisplayForward?ligandId=3429 (Antagonist) (p*K*_d_ 9.1) [http://www.ncbi.nlm.nih.gov/pubmed/10521582?dopt=AbstractPlus] – Rat, http://www.guidetopharmacology.org/GRAC/LigandDisplayForward?ligandId=1076 (Antagonist) (p*K*_d_ 9) [http://www.ncbi.nlm.nih.gov/pubmed/10692480?dopt=AbstractPlus] – Rat, http://www.guidetopharmacology.org/GRAC/LigandDisplayForward?ligandId=1077 (Antagonist) (p*K*_d_ 9) [http://www.ncbi.nlm.nih.gov/pubmed/9069281?dopt=AbstractPlus] – Rat, http://www.guidetopharmacology.org/GRAC/LigandDisplayForward?ligandId=3428 (Agonist)

**Subunits**

**Table:** 

Nomenclature	http://www.guidetopharmacology.org/GRAC/ObjectDisplayForward?objectId=240	http://www.guidetopharmacology.org/GRAC/ObjectDisplayForward?objectId=241
HGNC, UniProt	https://www.genenames.org/data/gene‐symbol‐report/#!/hgnc_id/HGNC:4070, http://www.uniprot.org/uniprot/Q9UBS5	https://www.genenames.org/data/gene‐symbol‐report/#!/hgnc_id/HGNC:4507, http://www.uniprot.org/uniprot/O75899

### Comments

Potencies of agonists and antagonists listed in the table, quantified as IC_50_ values for the inhibition of http://www.guidetopharmacology.org/GRAC/LigandDisplayForward?ligandId=5381 binding to rat cerebral cortex membranes, are from [http://www.ncbi.nlm.nih.gov/pubmed/12037141?dopt=AbstractPlus, http://www.ncbi.nlm.nih.gov/pubmed/21428811?dopt=AbstractPlus, 635]. Radioligand *K*_D_ values relate to binding to rat brain membranes. http://www.guidetopharmacology.org/GRAC/LigandDisplayForward?ligandId=1074 is a photoaffinity ligand for the GABA_B1_ subunit [http://www.ncbi.nlm.nih.gov/pubmed/10658574?dopt=AbstractPlus]. CGP27492 (http://www.guidetopharmacology.org/GRAC/LigandDisplayForward?ligandId=1081), CGP35024 (http://www.guidetopharmacology.org/GRAC/LigandDisplayForward?ligandId=1080) and http://www.guidetopharmacology.org/GRAC/LigandDisplayForward?ligandId=1082 act as antagonists at human GABA_A_ *ρ*1 receptors, with potencies in the low micromolar range [http://www.ncbi.nlm.nih.gov/pubmed/21428811?dopt=AbstractPlus]. In addition to the ligands listed in the table, http://www.guidetopharmacology.org/GRAC/LigandDisplayForward?ligandId=707 binds to the VTM of the GABA_B1_ subunit to act as a positive allosteric modulator of http://www.guidetopharmacology.org/GRAC/LigandDisplayForward?ligandId=1067 [http://www.ncbi.nlm.nih.gov/pubmed/10692480?dopt=AbstractPlus]. Synthetic positive allosteric modulators with low, or no, intrinsic activity include http://www.guidetopharmacology.org/GRAC/LigandDisplayForward?ligandId=1079, http://www.guidetopharmacology.org/GRAC/LigandDisplayForward?ligandId=5446, http://www.guidetopharmacology.org/GRAC/LigandDisplayForward?ligandId=5503 [http://www.ncbi.nlm.nih.gov/pubmed/21181127?dopt=AbstractPlus] and http://www.guidetopharmacology.org/GRAC/LigandDisplayForward?ligandId=5504 [http://www.ncbi.nlm.nih.gov/pubmed/17894647?dopt=AbstractPlus, http://www.ncbi.nlm.nih.gov/pubmed/15269338?dopt=AbstractPlus, http://www.ncbi.nlm.nih.gov/pubmed/15126507?dopt=AbstractPlus, http://www.ncbi.nlm.nih.gov/pubmed/21428811?dopt=AbstractPlus]. The site of action of http://www.guidetopharmacology.org/GRAC/LigandDisplayForward?ligandId=1079 and http://www.guidetopharmacology.org/GRAC/LigandDisplayForward?ligandId=5446 appears to be on the heptahelical domain of the GABA_B2_ subunit [http://www.ncbi.nlm.nih.gov/pubmed/16966477?dopt=AbstractPlus, http://www.ncbi.nlm.nih.gov/pubmed/15451400?dopt=AbstractPlus]. In the presence of http://www.guidetopharmacology.org/GRAC/LigandDisplayForward?ligandId=1079 or http://www.guidetopharmacology.org/GRAC/LigandDisplayForward?ligandId=5446, http://www.guidetopharmacology.org/GRAC/LigandDisplayForward?ligandId=1069 and http://www.guidetopharmacology.org/GRAC/LigandDisplayForward?ligandId=1068 behave as partial agonists [http://www.ncbi.nlm.nih.gov/pubmed/21428811?dopt=AbstractPlus]. A negative allosteric modulator of GABA_B_ activity has been reported [http://www.ncbi.nlm.nih.gov/pubmed/25050158?dopt=AbstractPlus]. Knock‐out of the GABA_B1_ subunit in C57B mice causes the development of severe tonic‐clonic convulsions that prove fatal within a month of birth, whereas GABA_B1_^‐/‐^ BALB/c mice, although also displaying spontaneous epileptiform activity, are viable. The phenotype of the latter animals additionally includes hyperalgesia, hyperlocomotion (in a novel, but not familiar, environment), hyperdopaminergia, memory impairment and behaviours indicative of anxiety [http://www.ncbi.nlm.nih.gov/pubmed/15451397?dopt=AbstractPlus, http://www.ncbi.nlm.nih.gov/pubmed/16606363?dopt=AbstractPlus]. A similar phenotype has been found for GABA_B2_^‐/‐^ BALB/c mice [http://www.ncbi.nlm.nih.gov/pubmed/15240800?dopt=AbstractPlus].

### Further reading on GABA_B_ receptors

Bowery NG *et al*. (2002) International Union of Pharmacology. XXXIII. Mammalian gammaaminobutyricacid(B) receptors: structure and function. *Pharmacol Rev.* **54**: 247‐264 [https://www.ncbi.nlm.nih.gov/pubmed/12037141?dopt=AbstractPlus]

Froestl W. (2011) An historical perspective on GABAergic drugs. *Future Med Chem* **3**: 163‐75 [https://www.ncbi.nlm.nih.gov/pubmed/21428811?dopt=AbstractPlus]

Gassmann M *et al*. (2012) Regulation of neuronal GABA(B) receptor functions by subunit composition. *Nat. Rev. Neurosci.* **13**: 380‐94 [https://www.ncbi.nlm.nih.gov/pubmed/22595784?dopt=AbstractPlus]

Pin JP *et al*. (2016) Organization and functions of mGlu and GABAB receptor complexes. *Nature* **540**: 60‐68 [https://www.ncbi.nlm.nih.gov/pubmed/27905440?dopt=AbstractPlus]

## http://www.guidetopharmacology.org/GRAC/FamilyDisplayForward?familyId=27

### Overview

Galanin receptors (**provisional nomenclature as recommended by NC‐IUPHAR** [http://www.ncbi.nlm.nih.gov/pubmed/15914470?dopt=AbstractPlus]) are activated by the endogenous peptides http://www.guidetopharmacology.org/GRAC/LigandDisplayForward?ligandId=3592 (https://www.genenames.org/data/gene‐symbol‐report/#!/hgnc_id/HGNC:4114, http://www.uniprot.org/uniprot/P22466) and http://www.guidetopharmacology.org/GRAC/LigandDisplayForward?ligandId=3594 (https://www.genenames.org/data/gene‐symbol‐report/#!/hgnc_id/HGNC:24840, http://www.uniprot.org/uniprot/Q9UBC7). Human http://www.guidetopharmacology.org/GRAC/LigandDisplayForward?ligandId=3592 (https://www.genenames.org/data/gene‐symbol‐report/#!/hgnc_id/HGNC:4114, http://www.uniprot.org/uniprot/P22466) is a 30 amino‐acid non‐amidated peptide [http://www.ncbi.nlm.nih.gov/pubmed/1714839?dopt=AbstractPlus]; in other species, it is 29 amino acids long and C‐terminally amidated. Amino acids 1–14 of galanin are highly conserved in mammals, birds, reptiles, amphibia and fish. Shorter peptide species (*e.g*. human galanin‐1–19 [http://www.ncbi.nlm.nih.gov/pubmed/1710578?dopt=AbstractPlus] and porcine galanin‐5–29 [http://www.ncbi.nlm.nih.gov/pubmed/1283627?dopt=AbstractPlus]) and N‐terminally extended forms (*e.g.* N‐terminally seven and nine residue elongated forms of porcine galanin [http://www.ncbi.nlm.nih.gov/pubmed/1718731?dopt=AbstractPlus, http://www.ncbi.nlm.nih.gov/pubmed/1283627?dopt=AbstractPlus]) have been reported.

**Table:** 

Nomenclature	http://www.guidetopharmacology.org/GRAC/ObjectDisplayForward?objectId=243	http://www.guidetopharmacology.org/GRAC/ObjectDisplayForward?objectId=244	http://www.guidetopharmacology.org/GRAC/ObjectDisplayForward?objectId=245
HGNC, UniProt	https://www.genenames.org/data/gene‐symbol‐report/#!/hgnc_id/HGNC:4132, http://www.uniprot.org/uniprot/P47211	https://www.genenames.org/data/gene‐symbol‐report/#!/hgnc_id/HGNC:4133, http://www.uniprot.org/uniprot/O43603	https://www.genenames.org/data/gene‐symbol‐report/#!/hgnc_id/HGNC:4134, http://www.uniprot.org/uniprot/O60755
Potency order of endogenous ligands	http://www.guidetopharmacology.org/GRAC/LigandDisplayForward?ligandId=3592 (https://www.genenames.org/data/gene‐symbol‐report/#!/hgnc_id/HGNC:4114, http://www.uniprot.org/uniprot/P22466) > http://www.guidetopharmacology.org/GRAC/LigandDisplayForward?ligandId=3594 (https://www.genenames.org/data/gene‐symbol‐report/#!/hgnc_id/HGNC:24840, http://www.uniprot.org/uniprot/Q9UBC7) [http://www.ncbi.nlm.nih.gov/pubmed/10601261?dopt=AbstractPlus]	http://www.guidetopharmacology.org/GRAC/LigandDisplayForward?ligandId=3594 (https://www.genenames.org/data/gene‐symbol‐report/#!/hgnc_id/HGNC:24840, http://www.uniprot.org/uniprot/Q9UBC7) ≥ http://www.guidetopharmacology.org/GRAC/LigandDisplayForward?ligandId=3592 (https://www.genenames.org/data/gene‐symbol‐report/#!/hgnc_id/HGNC:4114, http://www.uniprot.org/uniprot/P22466) [http://www.ncbi.nlm.nih.gov/pubmed/10601261?dopt=AbstractPlus]	http://www.guidetopharmacology.org/GRAC/LigandDisplayForward?ligandId=3594 (https://www.genenames.org/data/gene‐symbol‐report/#!/hgnc_id/HGNC:24840, http://www.uniprot.org/uniprot/Q9UBC7) > http://www.guidetopharmacology.org/GRAC/LigandDisplayForward?ligandId=3592 (https://www.genenames.org/data/gene‐symbol‐report/#!/hgnc_id/HGNC:4114, http://www.uniprot.org/uniprot/P22466) [http://www.ncbi.nlm.nih.gov/pubmed/15944009?dopt=AbstractPlus]
Agonists	–	http://www.guidetopharmacology.org/GRAC/LigandDisplayForward?ligandId=3876) [http://www.ncbi.nlm.nih.gov/pubmed/9832122?dopt=AbstractPlus, http://www.ncbi.nlm.nih.gov/pubmed/9742938?dopt=AbstractPlus, http://www.ncbi.nlm.nih.gov/pubmed/9281594?dopt=AbstractPlus, http://www.ncbi.nlm.nih.gov/pubmed/9405385?dopt=AbstractPlus] – Rat	–
Selective agonists	–	[http://www.guidetopharmacology.org/GRAC/LigandDisplayForward?ligandId=3866) [http://www.ncbi.nlm.nih.gov/pubmed/9305929?dopt=AbstractPlus] – Rat	–
Selective antagonists	http://www.guidetopharmacology.org/GRAC/LigandDisplayForward?ligandId=3486 (pIC_50_ 5.6) [http://www.ncbi.nlm.nih.gov/pubmed/10896115?dopt=AbstractPlus]	http://www.guidetopharmacology.org/GRAC/LigandDisplayForward?ligandId=3900 (p*K*_i_ 7.9) [2010]	http://www.guidetopharmacology.org/GRAC/LigandDisplayForward?ligandId=6125 (p*K*_i_ 8.3) [http://www.ncbi.nlm.nih.gov/pubmed/16789730?dopt=AbstractPlus, http://www.ncbi.nlm.nih.gov/pubmed/16730981?dopt=AbstractPlus, http://www.ncbi.nlm.nih.gov/pubmed/16287967?dopt=AbstractPlus], http://www.guidetopharmacology.org/GRAC/LigandDisplayForward?ligandId=6126 (p*K*_i_ 7.8–7.8) [http://www.ncbi.nlm.nih.gov/pubmed/16789730?dopt=AbstractPlus, http://www.ncbi.nlm.nih.gov/pubmed/16730981?dopt=AbstractPlus, http://www.ncbi.nlm.nih.gov/pubmed/16287967?dopt=AbstractPlus]
Selective allosteric modulators	–	http://www.guidetopharmacology.org/GRAC/LigandDisplayForward?ligandId=8547 (Positive) (pEC_50_ 9.2) [http://www.ncbi.nlm.nih.gov/pubmed/20660766?dopt=AbstractPlus] – Rat	–
Labelled ligands	http://www.guidetopharmacology.org/GRAC/LigandDisplayForward?ligandId=5391 (Agonist) [http://www.ncbi.nlm.nih.gov/pubmed/9808667?dopt=AbstractPlus], http://www.guidetopharmacology.org/GRAC/LigandDisplayForward?ligandId=5391 (Agonist) [http://www.ncbi.nlm.nih.gov/pubmed/9808667?dopt=AbstractPlus]	http://www.guidetopharmacology.org/GRAC/LigandDisplayForward?ligandId=5391 (Agonist) [http://www.ncbi.nlm.nih.gov/pubmed/9281594?dopt=AbstractPlus] – Rat	http://www.guidetopharmacology.org/GRAC/LigandDisplayForward?ligandId=6117 (Agonist) [http://www.ncbi.nlm.nih.gov/pubmed/9880084?dopt=AbstractPlus, http://www.ncbi.nlm.nih.gov/pubmed/9722565?dopt=AbstractPlus]
Comments	–	The CYM2503 PAM potentiates the anticonvulsant activity of endogenous galanin in mouse seizure models [http://www.ncbi.nlm.nih.gov/pubmed/20660766?dopt=AbstractPlus].	–

### Comments

http://www.guidetopharmacology.org/GRAC/LigandDisplayForward?ligandId=5356) is a high‐affinity agonist at GAL_1_/GAL_2_ (p*K*i 9), and http://www.guidetopharmacology.org/GRAC/LigandDisplayForward?ligandId=5357) is selective for GAL_2_ and GAL_3_ compared with GAL_1_ [http://www.ncbi.nlm.nih.gov/pubmed/15944007?dopt=AbstractPlus]. [^125^I]‐[Tyr^26^]galanin binds to all three subtypes with *K*_d_ values generally reported to range from 0.05 to 1 nM, depending on the assay conditions used [http://www.ncbi.nlm.nih.gov/pubmed/9808667?dopt=AbstractPlus, http://www.ncbi.nlm.nih.gov/pubmed/2436195?dopt=AbstractPlus, http://www.ncbi.nlm.nih.gov/pubmed/9305929?dopt=AbstractPlus, http://www.ncbi.nlm.nih.gov/pubmed/9722565?dopt=AbstractPlus, http://www.ncbi.nlm.nih.gov/pubmed/9281594?dopt=AbstractPlus]. Porcine galanin‐(3‐29) does not bind to cloned GAL_1_, GAL_2_ or GAL_3_ receptors, but a receptor that is functionally activated by porcine galanin‐(3–29) has been reported in pituitary and gastric smooth muscle cells [http://www.ncbi.nlm.nih.gov/pubmed/7529309?dopt=AbstractPlus, http://www.ncbi.nlm.nih.gov/pubmed/7683428?dopt=AbstractPlus]. Additional galanin receptor subtypes are also suggested from studies with chimeric peptides (*e.g*. http://www.guidetopharmacology.org/GRAC/LigandDisplayForward?ligandId=3896, http://www.guidetopharmacology.org/GRAC/LigandDisplayForward?ligandId=3898 and http://www.guidetopharmacology.org/GRAC/LigandDisplayForward?ligandId=3899), which act as antagonists in functional assays in the cardiovascular system [http://www.ncbi.nlm.nih.gov/pubmed/7693918?dopt=AbstractPlus], spinal cord [http://www.ncbi.nlm.nih.gov/pubmed/1373497?dopt=AbstractPlus], locus coeruleus, hippocampus [http://www.ncbi.nlm.nih.gov/pubmed/1720557?dopt=AbstractPlus] and hypothalamus [http://www.ncbi.nlm.nih.gov/pubmed/7504301?dopt=AbstractPlus, http://www.ncbi.nlm.nih.gov/pubmed/1283559?dopt=AbstractPlus], but exhibit agonist activity at some peripheral sites [http://www.ncbi.nlm.nih.gov/pubmed/7504301?dopt=AbstractPlus, http://www.ncbi.nlm.nih.gov/pubmed/7529309?dopt=AbstractPlus]. The chimeric peptides http://www.guidetopharmacology.org/GRAC/LigandDisplayForward?ligandId=3896, http://www.guidetopharmacology.org/GRAC/LigandDisplayForward?ligandId=3897, http://www.guidetopharmacology.org/GRAC/LigandDisplayForward?ligandId=3898, http://www.guidetopharmacology.org/GRAC/LigandDisplayForward?ligandId=3899 and http://www.guidetopharmacology.org/GRAC/LigandDisplayForward?ligandId=3861 are agonists at GAL_1_ receptors expressed endogenously in Bowes human melanoma cells [http://www.ncbi.nlm.nih.gov/pubmed/10601261?dopt=AbstractPlus], and at heterologously expressed recombinant GAL_1_, GAL_2_ and GAL_3_ receptors [http://www.ncbi.nlm.nih.gov/pubmed/9808667?dopt=AbstractPlus, http://www.ncbi.nlm.nih.gov/pubmed/9305929?dopt=AbstractPlus, http://www.ncbi.nlm.nih.gov/pubmed/9722565?dopt=AbstractPlus]. Recent studies have described the synthesis of a series of novel, systemically‐active, galanin analogues, with modest preferential binding at the GAL_2_ receptor. Specific chemical modifications to the galanin backbone increased brain levels of these peptides after *i.v.* injection and several of these peptides exerted a potent antidepressant‐like effect in mouse models of depression [http://www.ncbi.nlm.nih.gov/pubmed/23600864?dopt=AbstractPlus].

### Further reading on Galanin receptors

Foord SM *et al*. (2005) International Union of Pharmacology. XLVI. G protein‐coupled receptor list. *Pharmacol Rev* **57**: 279‐288 [https://www.ncbi.nlm.nih.gov/pubmed/15914470?dopt=AbstractPlus]

Lang R *et al*. (2015) Physiology, signaling, and pharmacology of galanin peptides and receptors: three decades of emerging diversity. *Pharmacol. Rev.* **67**: 118‐75 [https://www.ncbi.nlm.nih.gov/pubmed/25428932?dopt=AbstractPlus]

Lang R *et al*. (2011) The galanin peptide family in inflammation. *Neuropeptides* **45**: 1‐8 [https://www.ncbi.nlm.nih.gov/pubmed/21087790?dopt=AbstractPlus]

Lawrence C *et al*. (2011) Galanin‐like peptide (GALP) is a hypothalamic regulator of energy homeostasis and reproduction. *Front Neuroendocrinol* **32**: 1‐9 [https://www.ncbi.nlm.nih.gov/pubmed/20558195?dopt=AbstractPlus]

Webling KE *et al*. (2012) Galanin receptors and ligands. *Front Endocrinol (Lausanne)* **3**: 146 [https://www.ncbi.nlm.nih.gov/pubmed/23233848?dopt=AbstractPlus]

## http://www.guidetopharmacology.org/GRAC/FamilyDisplayForward?familyId=28

### Overview

The ghrelin receptor (**nomenclature as agreed by the NC‐IUPHAR Subcommittee for the Ghrelin receptor** [http://www.ncbi.nlm.nih.gov/pubmed/16382107?dopt=AbstractPlus]) is activated by a 28 amino‐acid peptide originally isolated from rat stomach, where it is cleaved from a 117 aminoacid precursor (https://www.genenames.org/data/gene‐symbol‐report/#!/hgnc_id/HGNC:18129, http://www.uniprot.org/uniprot/Q9UBU3). The human gene encoding the precursor peptide has 83% sequence homology to rat preproghrelin, although the mature peptides from rat and human differ by only two amino acids [http://www.ncbi.nlm.nih.gov/pubmed/11549267?dopt=AbstractPlus]. Alternative splicing results in the formation of a second peptide, [http://www.guidetopharmacology.org/GRAC/LigandDisplayForward?ligandId=3600 (https://www.genenames.org/data/gene‐symbol‐report/#!/hgnc_id/HGNC:18129, http://www.uniprot.org/uniprot/Q9UBU3) with equipotent biological activity [http://www.ncbi.nlm.nih.gov/pubmed/10801861?dopt=AbstractPlus]. A unique post‐translational modification (octanoylation of Ser^3^, catalysed by ghrelin *O*‐acyltransferase (https://www.genenames.org/data/gene‐symbol‐report/#!/hgnc_id/HGNC:32311, http://www.uniprot.org/uniprot/Q96T53) [http://www.ncbi.nlm.nih.gov/pubmed/18267071?dopt=AbstractPlus] occurs in both peptides, essential for full activity in binding to ghrelin receptors in the hypothalamus and pituitary, and for the release of growth hormone from the pituitary [http://www.ncbi.nlm.nih.gov/pubmed/10604470?dopt=AbstractPlus]. Structure activity studies showed the first five N‐terminal amino acids to be the minimum required for binding [http://www.ncbi.nlm.nih.gov/pubmed/11087562?dopt=AbstractPlus], and receptor mutagenesis has indicated overlap of the ghrelin binding site with those for small molecule agonists and allosteric modulators of http://www.guidetopharmacology.org/GRAC/LigandDisplayForward?ligandId=1099 (https://www.genenames.org/data/gene‐symbol‐report/#!/hgnc_id/HGNC:18129, http://www.uniprot.org/uniprot/Q9UBU3) function [http://www.ncbi.nlm.nih.gov/pubmed/18923064?dopt=AbstractPlus]. In cell systems, the ghrelin receptor is constitutively active [http://www.ncbi.nlm.nih.gov/pubmed/15383539?dopt=AbstractPlus], but this is abolished by a naturally occurring mutation (A204E) that results in decreased cell surface receptor expression and is associated with familial short stature [http://www.ncbi.nlm.nih.gov/pubmed/16511605?dopt=AbstractPlus].

**Table:** 

Nomenclature	http://www.guidetopharmacology.org/GRAC/ObjectDisplayForward?objectId=246
HGNC, UniProt	https://www.genenames.org/data/gene‐symbol‐report/#!/hgnc_id/HGNC:4267, http://www.uniprot.org/uniprot/Q92847
Potency order of endogenous ligands	http://www.guidetopharmacology.org/GRAC/LigandDisplayForward?ligandId=1099 (https://www.genenames.org/data/gene‐symbol‐report/#!/hgnc_id/HGNC:18129, http://www.uniprot.org/uniprot/Q9UBU3) = [http://www.guidetopharmacology.org/GRAC/LigandDisplayForward?ligandId=3600 (https://www.genenames.org/data/gene‐symbol‐report/#!/hgnc_id/HGNC:18129, http://www.uniprot.org/uniprot/Q9UBU3) [http://www.ncbi.nlm.nih.gov/pubmed/12969753?dopt=AbstractPlus, http://www.ncbi.nlm.nih.gov/pubmed/11549267?dopt=AbstractPlus]
Antagonists	http://www.guidetopharmacology.org/GRAC/LigandDisplayForward?ligandId=9734 (https://www.genenames.org/data/gene‐symbol‐report/#!/hgnc_id/HGNC:29571, http://www.uniprot.org/uniprot/Q969E1) (pIC_50_ 8.2) [675]
Selective antagonists	http://www.guidetopharmacology.org/GRAC/LigandDisplayForward?ligandId=5870 (pIC_50_ 8.4) [http://www.ncbi.nlm.nih.gov/pubmed/20593439?dopt=AbstractPlus], http://www.guidetopharmacology.org/GRAC/LigandDisplayForward?ligandId=5870 (p*K*_B_ 8) [http://www.ncbi.nlm.nih.gov/pubmed/21034740?dopt=AbstractPlus] – Rat
Labelled ligands	[http://www.guidetopharmacology.org/GRAC/LigandDisplayForward?ligandId=1094) (Agonist) [http://www.ncbi.nlm.nih.gov/pubmed/11522606?dopt=AbstractPlus], [http://www.guidetopharmacology.org/GRAC/LigandDisplayForward?ligandId=1096) (Agonist) [http://www.ncbi.nlm.nih.gov/pubmed/11314756?dopt=AbstractPlus]

### Comments

[http://www.guidetopharmacology.org/GRAC/LigandDisplayForward?ligandId=1098 (https://www.genenames.org/data/gene‐symbol‐report/#!/hgnc_id/HGNC:18129, http://www.uniprot.org/uniprot/Q9UBU3) has been shown to bind (as [http://www.guidetopharmacology.org/GRAC/LigandDisplayForward?ligandId=3810) and have effects in the cardiovascular system [http://www.ncbi.nlm.nih.gov/pubmed/12969753?dopt=AbstractPlus], which raises the possible existence of different receptor subtypes in peripheral tissues and the central nervous system. A potent inverse agonist has been identified ([http://www.guidetopharmacology.org/GRAC/LigandDisplayForward?ligandId=1102, p*D*_2_ 8.3; [http://www.ncbi.nlm.nih.gov/pubmed/12907757?dopt=AbstractPlus]). http://www.guidetopharmacology.org/GRAC/LigandDisplayForward?ligandId=3535, described as a ghrelin receptor agonist (p*K*i 7.8 and p*D*_2_ 7.5 at human recombinant ghrelin receptors), has been shown to stimulate ghrelin receptor mediated food intake and gastric emptying but not elicit release of growth hormone, or modify ghrelin stimulated growth hormone release, thus pharmacologically discriminating the orexigenic and gastrointestinal actions of http://www.guidetopharmacology.org/GRAC/LigandDisplayForward?ligandId=1099 (https://www.genenames.org/data/gene‐symbol‐report/#!/hgnc_id/HGNC:18129, http://www.uniprot.org/uniprot/Q9UBU3) from the release of growth hormone [http://www.ncbi.nlm.nih.gov/pubmed/18719021?dopt=AbstractPlus]. A number of selective antagonists have been reported, including peptidomimetic [http://www.ncbi.nlm.nih.gov/pubmed/22798076?dopt=AbstractPlus] and non‐peptide small molecules including http://www.guidetopharmacology.org/GRAC/LigandDisplayForward?ligandId=5870 [http://www.ncbi.nlm.nih.gov/pubmed/21034740?dopt=AbstractPlus, http://www.ncbi.nlm.nih.gov/pubmed/20593439?dopt=AbstractPlus].

### Further reading on Ghrelin receptor

Andrews ZB. (2011) The extra‐hypothalamic actions of ghrelin on neuronal function. *Trends Neurosci.* **34**: 31‐40 [https://www.ncbi.nlm.nih.gov/pubmed/21035199?dopt=AbstractPlus]

Angelidis G *et al*. (2010) Current and potential roles of ghrelin in clinical practice. *J. Endocrinol. Invest.* **33**: 823‐38 [https://www.ncbi.nlm.nih.gov/pubmed/21293171?dopt=AbstractPlus]

Briggs DI *et al*. (2011) Metabolic status regulates ghrelin function on energy homeostasis. *Neuroendocrinology* **93**: 48‐57 [https://www.ncbi.nlm.nih.gov/pubmed/21124019?dopt=AbstractPlus]

Callaghan B *et al*. (2014) Novel and conventional receptors for ghrelin, desacyl‐ghrelin, and pharmacologically related compounds. *Pharmacol. Rev.* **66**: 984‐1001 [https://www.ncbi.nlm.nih.gov/pubmed/25107984?dopt=AbstractPlus]

Davenport AP *et al*. (2005) International Union of Pharmacology. LVI. Ghrelin receptor nomenclature, distribution, and function. *Pharmacol. Rev.* **57**: 541‐6 [https://www.ncbi.nlm.nih.gov/pubmed/16382107?dopt=AbstractPlus]

## http://www.guidetopharmacology.org/GRAC/FamilyDisplayForward?familyId=29

### Overview

The glucagon family of receptors (**nomenclature as agreed by the NC‐IUPHAR Subcommittee on the Glucagon receptor family** [http://www.ncbi.nlm.nih.gov/pubmed/12615957?dopt=AbstractPlus]) are activated by the endogenous peptide (27‐44 aa) hormones http://www.guidetopharmacology.org/GRAC/LigandDisplayForward?ligandId=1136 (https://www.genenames.org/data/gene‐symbol‐report/#!/hgnc_id/HGNC:4191, http://www.uniprot.org/uniprot/P01275), http://www.guidetopharmacology.org/GRAC/LigandDisplayForward?ligandId=5194 (https://www.genenames.org/data/gene‐symbol‐report/#!/hgnc_id/HGNC:4191, http://www.uniprot.org/uniprot/P01275), http://www.guidetopharmacology.org/GRAC/LigandDisplayForward?ligandId=1140 (https://www.genenames.org/data/gene‐symbol‐report/#!/hgnc_id/HGNC:4191, http://www.uniprot.org/uniprot/P01275), glucose‐dependent insulinotropic polypeptide (also known as http://www.guidetopharmacology.org/GRAC/LigandDisplayForward?ligandId=3542 (https://www.genenames.org/data/gene‐symbol‐report/#!/hgnc_id/HGNC:4270, http://www.uniprot.org/uniprot/P09681)), http://www.guidetopharmacology.org/GRAC/LigandDisplayForward?ligandId=2270 (https://www.genenames.org/data/gene‐symbol‐report/#!/hgnc_id/HGNC:4265, http://www.uniprot.org/uniprot/P01286) and http://www.guidetopharmacology.org/GRAC/LigandDisplayForward?ligandId=3643 (https://www.genenames.org/data/gene‐symbol‐report/#!/hgnc_id/HGNC:10607, http://www.uniprot.org/uniprot/P09683). One common precursor (https://www.genenames.org/data/gene‐symbol‐report/#!/hgnc_id/HGNC:4191) generates http://www.guidetopharmacology.org/GRAC/LigandDisplayForward?ligandId=1136 (https://www.genenames.org/data/gene‐symbol‐report/#!/hgnc_id/HGNC:4191, http://www.uniprot.org/uniprot/P01275), http://www.guidetopharmacology.org/GRAC/LigandDisplayForward?ligandId=5194 (https://www.genenames.org/data/gene‐symbol‐report/#!/hgnc_id/HGNC:4191, http://www.uniprot.org/uniprot/P01275) and http://www.guidetopharmacology.org/GRAC/LigandDisplayForward?ligandId=1140 (https://www.genenames.org/data/gene‐symbol‐report/#!/hgnc_id/HGNC:4191, http://www.uniprot.org/uniprot/P01275) peptides [http://www.ncbi.nlm.nih.gov/pubmed/11179772?dopt=AbstractPlus]. For a recent review on review the current understanding of the structures of GLP‐1 and GLP‐1R, the molecular basis of their interaction, and the signaling events associated with it, see de Graaf et al., 2016 [http://www.ncbi.nlm.nih.gov/pubmed/27630114?dopt=AbstractPlus].

**Table:** 

Nomenclature	http://www.guidetopharmacology.org/GRAC/ObjectDisplayForward?objectId=247	http://www.guidetopharmacology.org/GRAC/ObjectDisplayForward?objectId=248	http://www.guidetopharmacology.org/GRAC/ObjectDisplayForward?objectId=249
HGNC, UniProt	https://www.genenames.org/data/gene‐symbol‐report/#!/hgnc_id/HGNC:4266, http://www.uniprot.org/uniprot/Q02643	https://www.genenames.org/data/gene‐symbol‐report/#!/hgnc_id/HGNC:4271, http://www.uniprot.org/uniprot/P48546	https://www.genenames.org/data/gene‐symbol‐report/#!/hgnc_id/HGNC:4324, http://www.uniprot.org/uniprot/P43220
Endogenous agonists	http://www.guidetopharmacology.org/GRAC/LigandDisplayForward?ligandId=2270 (https://www.genenames.org/data/gene‐symbol‐report/#!/hgnc_id/HGNC:4265, http://www.uniprot.org/uniprot/P01286)	http://www.guidetopharmacology.org/GRAC/LigandDisplayForward?ligandId=3542 (https://www.genenames.org/data/gene‐symbol‐report/#!/hgnc_id/HGNC:4270, http://www.uniprot.org/uniprot/P09681) [http://www.ncbi.nlm.nih.gov/pubmed/7589426?dopt=AbstractPlus]	http://www.guidetopharmacology.org/GRAC/LigandDisplayForward?ligandId=1132 (https://www.genenames.org/data/gene‐symbol‐report/#!/hgnc_id/HGNC:4191, http://www.uniprot.org/uniprot/P01275) [http://www.ncbi.nlm.nih.gov/pubmed/15528268?dopt=AbstractPlus], http://www.guidetopharmacology.org/GRAC/LigandDisplayForward?ligandId=3544) (https://www.genenames.org/data/gene‐symbol‐report/#!/hgnc_id/HGNC:4191, https://www.genenames.org/data/gene‐symbol‐report/#!/hgnc_id/HGNC:4191) [http://www.ncbi.nlm.nih.gov/pubmed/8404634?dopt=AbstractPlus]
Agonists	http://www.guidetopharmacology.org/GRAC/LigandDisplayForward?ligandId=6523 [http://www.ncbi.nlm.nih.gov/pubmed/24373935?dopt=AbstractPlus], http://www.guidetopharmacology.org/GRAC/LigandDisplayForward?ligandId=6998	–	http://www.guidetopharmacology.org/GRAC/LigandDisplayForward?ligandId=1133 [http://www.ncbi.nlm.nih.gov/pubmed/10794683?dopt=AbstractPlus], http://www.guidetopharmacology.org/GRAC/LigandDisplayForward?ligandId=7387 [http://www.ncbi.nlm.nih.gov/pubmed/20570597?dopt=AbstractPlus], http://www.guidetopharmacology.org/GRAC/LigandDisplayForward?ligandId=8544 [http://www.ncbi.nlm.nih.gov/pubmed/25176008?dopt=AbstractPlus]
Selective agonists	http://www.guidetopharmacology.org/GRAC/LigandDisplayForward?ligandId=3857 [http://www.ncbi.nlm.nih.gov/pubmed/8993400?dopt=AbstractPlus], http://www.guidetopharmacology.org/GRAC/LigandDisplayForward?ligandId=6959	–	http://www.guidetopharmacology.org/GRAC/LigandDisplayForward?ligandId=9724 [http://www.ncbi.nlm.nih.gov/pubmed/26308095?dopt=AbstractPlus], http://www.guidetopharmacology.org/GRAC/LigandDisplayForward?ligandId=1135 [http://www.ncbi.nlm.nih.gov/pubmed/18412318?dopt=AbstractPlus], http://www.guidetopharmacology.org/GRAC/LigandDisplayForward?ligandId=1135 [http://www.ncbi.nlm.nih.gov/pubmed/15528268?dopt=AbstractPlus], http://www.guidetopharmacology.org/GRAC/LigandDisplayForward?ligandId=3730 (http://www.uniprot.org/uniprot/P20394) [http://www.ncbi.nlm.nih.gov/pubmed/1704369?dopt=AbstractPlus]
Selective antagonists	http://www.guidetopharmacology.org/GRAC/LigandDisplayForward?ligandId=1107 (p*K*_i_ 10.1–10.4) [http://www.ncbi.nlm.nih.gov/pubmed/10542394?dopt=AbstractPlus, http://www.ncbi.nlm.nih.gov/pubmed/9892695?dopt=AbstractPlus, http://www.ncbi.nlm.nih.gov/pubmed/14755056?dopt=AbstractPlus] – Rat, http://www.guidetopharmacology.org/GRAC/LigandDisplayForward?ligandId=1109 (p*K*_i_ 10.1) [http://www.ncbi.nlm.nih.gov/pubmed/10542394?dopt=AbstractPlus, http://www.ncbi.nlm.nih.gov/pubmed/9892695?dopt=AbstractPlus, http://www.ncbi.nlm.nih.gov/pubmed/14755056?dopt=AbstractPlus] – Rat	[http://www.guidetopharmacology.org/GRAC/LigandDisplayForward?ligandId=3847]http://www.guidetopharmacology.org/GRAC/LigandDisplayForward?ligandId=3847 [http://www.ncbi.nlm.nih.gov/pubmed/12627321?dopt=AbstractPlus] – Mouse	http://www.guidetopharmacology.org/GRAC/LigandDisplayForward?ligandId=1138) (p*K*_i_ 8.1) [http://www.ncbi.nlm.nih.gov/pubmed/15528268?dopt=AbstractPlus], http://www.guidetopharmacology.org/GRAC/LigandDisplayForward?ligandId=6524) (pIC_50_ 6.9) [http://www.ncbi.nlm.nih.gov/pubmed/9261127?dopt=AbstractPlus] – Rat, http://www.guidetopharmacology.org/GRAC/LigandDisplayForward?ligandId=884 (pIC_50_ 4.7) [http://www.ncbi.nlm.nih.gov/pubmed/11498540?dopt=AbstractPlus]
Labelled ligands	[http://www.guidetopharmacology.org/GRAC/LigandDisplayForward?ligandId=3783) (Agonist) [http://www.ncbi.nlm.nih.gov/pubmed/11814616?dopt=AbstractPlus] – Rat	[http://www.guidetopharmacology.org/GRAC/LigandDisplayForward?ligandId=1131) (Agonist) [http://www.ncbi.nlm.nih.gov/pubmed/8795084?dopt=AbstractPlus] – Rat	[http://www.guidetopharmacology.org/GRAC/LigandDisplayForward?ligandId=1134 (Agonist) [http://www.ncbi.nlm.nih.gov/pubmed/15528268?dopt=AbstractPlus], [http://www.guidetopharmacology.org/GRAC/LigandDisplayForward?ligandId=1137) (Antagonist) (p*K*_d_ 8.3) [http://www.ncbi.nlm.nih.gov/pubmed/15528268?dopt=AbstractPlus], [http://www.guidetopharmacology.org/GRAC/LigandDisplayForward?ligandId=3784) (Agonist)

**Table:** 

Nomenclature	http://www.guidetopharmacology.org/GRAC/ObjectDisplayForward?objectId=250	http://www.guidetopharmacology.org/GRAC/ObjectDisplayForward?objectId=251	http://www.guidetopharmacology.org/GRAC/ObjectDisplayForward?objectId=252
HGNC, UniProt	https://www.genenames.org/data/gene‐symbol‐report/#!/hgnc_id/HGNC:4325, http://www.uniprot.org/uniprot/O95838	https://www.genenames.org/data/gene‐symbol‐report/#!/hgnc_id/HGNC:4192, http://www.uniprot.org/uniprot/P47871	https://www.genenames.org/data/gene‐symbol‐report/#!/hgnc_id/HGNC:10608, http://www.uniprot.org/uniprot/P47872
Endogenous agonists	http://www.guidetopharmacology.org/GRAC/LigandDisplayForward?ligandId=1140 (https://www.genenames.org/data/gene‐symbol‐report/#!/hgnc_id/HGNC:4191, http://www.uniprot.org/uniprot/P01275) [http://www.ncbi.nlm.nih.gov/pubmed/11738243?dopt=AbstractPlus]	http://www.guidetopharmacology.org/GRAC/LigandDisplayForward?ligandId=1136 (https://www.genenames.org/data/gene‐symbol‐report/#!/hgnc_id/HGNC:4191, http://www.uniprot.org/uniprot/P01275) [http://www.ncbi.nlm.nih.gov/pubmed/4305077?dopt=AbstractPlus]	http://www.guidetopharmacology.org/GRAC/LigandDisplayForward?ligandId=3643 (https://www.genenames.org/data/gene‐symbol‐report/#!/hgnc_id/HGNC:10607, http://www.uniprot.org/uniprot/P09683) [http://www.ncbi.nlm.nih.gov/pubmed/7612008?dopt=AbstractPlus]
Agonists	http://www.guidetopharmacology.org/GRAC/LigandDisplayForward?ligandId=7049 [http://www.ncbi.nlm.nih.gov/pubmed/25859983?dopt=AbstractPlus]	http://www.guidetopharmacology.org/GRAC/LigandDisplayForward?ligandId=9760 [http://www.ncbi.nlm.nih.gov/pubmed/29300013?dopt=AbstractPlus]	–
Selective antagonists	–	http://www.guidetopharmacology.org/GRAC/LigandDisplayForward?ligandId=3505 (pIC_50_ 8.4) [http://www.ncbi.nlm.nih.gov/pubmed/10085108?dopt=AbstractPlus], http://www.guidetopharmacology.org/GRAC/LigandDisplayForward?ligandId=9479 (p*K*_i_ 8.2) [http://www.ncbi.nlm.nih.gov/pubmed/26681715?dopt=AbstractPlus, http://www.ncbi.nlm.nih.gov/pubmed/25656305?dopt=AbstractPlus], http://www.guidetopharmacology.org/GRAC/LigandDisplayForward?ligandId=1150 (p*A*_2_ 7.2) [http://www.ncbi.nlm.nih.gov/pubmed/3035568?dopt=AbstractPlus, http://www.ncbi.nlm.nih.gov/pubmed/2560175?dopt=AbstractPlus] – Rat, http://www.guidetopharmacology.org/GRAC/LigandDisplayForward?ligandId=3515 (p*K*_i_ 5) [http://www.ncbi.nlm.nih.gov/pubmed/9857085?dopt=AbstractPlus], http://www.guidetopharmacology.org/GRAC/LigandDisplayForward?ligandId=3491 [http://www.ncbi.nlm.nih.gov/pubmed/11719833?dopt=AbstractPlus]	[(http://www.guidetopharmacology.org/GRAC/LigandDisplayForward?ligandId=3755 (p*K*_i_ 5.3) [http://www.ncbi.nlm.nih.gov/pubmed/1702423?dopt=AbstractPlus]
Labelled ligands	–	[http://www.guidetopharmacology.org/GRAC/LigandDisplayForward?ligandId=3785) (Agonist)	[http://www.guidetopharmacology.org/GRAC/LigandDisplayForward?ligandId=3759) (Agonist) [http://www.ncbi.nlm.nih.gov/pubmed/9843782?dopt=AbstractPlus] – Rat

### Comments

The glucagon receptor has been reported to interact with receptor activity modifying proteins (RAMPs), specifically http://www.guidetopharmacology.org/GRAC/ObjectDisplayForward?objectId=52, in heterologous expression systems [http://www.ncbi.nlm.nih.gov/pubmed/12446722?dopt=AbstractPlus], although the physiological significance of this has yet to be established.

### Further reading on Glucagon receptor family

Ahrén B. (2019) Glucagon‐like peptide‐1 receptor agonists for type 2 diabetes: A rational drug development. *J Diabetes Investig* **10**: 196‐201 [https://www.ncbi.nlm.nih.gov/pubmed/30099845?dopt=AbstractPlus]

Andersen A *et al*. (2018) Glucagon‐like peptide 1 in health and disease. *Nat Rev Endocrinol* **14**: 390‐403 [https://www.ncbi.nlm.nih.gov/pubmed/29728598?dopt=AbstractPlus]

Gentilella R *et al*. (2019) Glucagon‐like peptide‐1 receptor agonists in type 2 diabetes treatment: are they all the same? *Diabetes Metab. Res. Rev.* **35**: e3070 [https://www.ncbi.nlm.nih.gov/pubmed/30156747?dopt=AbstractPlus]

Graaf Cd *et al*. (2016) Glucagon‐Like Peptide‐1 and Its Class B G Protein‐Coupled Receptors: A Long March to Therapeutic Successes. *Pharmacol. Rev.* **68**: 954‐1013 [https://www.ncbi.nlm.nih.gov/pubmed/27630114?dopt=AbstractPlus]

Romera I *et al*. (2019) A Review of Practical Issues on the Use of Glucagon‐Like Peptide‐1 Receptor Agonists for the Management of Type 2 Diabetes. *Diabetes Ther* **10**: 5‐19 [https://www.ncbi.nlm.nih.gov/pubmed/30506340?dopt=AbstractPlus]

Trujillo JM *et al*. (2014) GLP‐1 receptor agonists for type 2 diabetes mellitus: recent developments and emerging agents. *Pharmacotherapy* **34**: 1174‐86 [https://www.ncbi.nlm.nih.gov/pubmed/25382096?dopt=AbstractPlus]

Zhang Y *et al*. (2017) Cryo‐EM structure of the activated GLP‐1 receptor in complex with a G protein. *Nature* **546**: 248‐253 [https://www.ncbi.nlm.nih.gov/pubmed/28538729?dopt=AbstractPlus]

## http://www.guidetopharmacology.org/GRAC/FamilyDisplayForward?familyId=30

### Overview

Glycoprotein hormone receptors (**provisional nomenclature** [http://www.ncbi.nlm.nih.gov/pubmed/15914470?dopt=AbstractPlus]) are activated by a non‐covalent heterodimeric glycoprotein made up of a common α chain

(http://www.guidetopharmacology.org/GRAC/LigandDisplayForward?ligandId=3731 (https://www.genenames.org/data/gene‐symbol‐report/#!/hgnc_id/HGNC:1885, http://www.uniprot.org/uniprot/P01215) https://www.genenames.org/data/gene‐symbol‐report/#!/hgnc_id/HGNC:1885, http://www.uniprot.org/uniprot/P01215), with a unique β chain that confers the biological specificity to http://www.guidetopharmacology.org/GRAC/LigandDisplayForward?ligandId=1157 (https://www.genenames.org/data/gene‐symbol‐report/#!/hgnc_id/HGNC:1885 https://www.genenames.org/data/gene‐symbol‐report/#!/hgnc_id/HGNC:3964, http://www.uniprot.org/uniprot/P01215 http://www.uniprot.org/uniprot/P01225), http://www.guidetopharmacology.org/GRAC/LigandDisplayForward?ligandId=1159 (https://www.genenames.org/data/gene‐symbol‐report/#!/hgnc_id/HGNC:1885 https://www.genenames.org/data/gene‐symbol‐report/#!/hgnc_id/HGNC:6584, http://www.uniprot.org/uniprot/P01215 http://www.uniprot.org/uniprot/P01229), http://www.guidetopharmacology.org/GRAC/LigandDisplayForward?ligandId=1160 (https://www.genenames.org/data/gene‐symbol‐report/#!/hgnc_id/HGNC:1885 https://www.genenames.org/data/gene‐symbol‐report/#!/hgnc_id/HGNC:1886, http://www.uniprot.org/uniprot/P01215 http://www.uniprot.org/uniprot/P01233) or http://www.guidetopharmacology.org/GRAC/LigandDisplayForward?ligandId=3920 (https://www.genenames.org/data/gene‐symbol‐report/#!/hgnc_id/HGNC:1885 https://www.genenames.org/data/gene‐symbol‐report/#!/hgnc_id/HGNC:12372, http://www.uniprot.org/uniprot/P01215 http://www.uniprot.org/uniprot/P01222). There is binding cross‐reactivity across the endogenous agonists for each of the glycoprotein hormone receptors. The deglycosylated hormones appear to exhibit reduced efficacy at these receptors [http://www.ncbi.nlm.nih.gov/pubmed/2542111?dopt=AbstractPlus].

**Table:** 

Nomenclature	http://www.guidetopharmacology.org/GRAC/ObjectDisplayForward?objectId=253	http://www.guidetopharmacology.org/GRAC/ObjectDisplayForward?objectId=254	http://www.guidetopharmacology.org/GRAC/ObjectDisplayForward?objectId=255
HGNC, UniProt	https://www.genenames.org/data/gene‐symbol‐report/#!/hgnc_id/HGNC:3969, http://www.uniprot.org/uniprot/P23945	https://www.genenames.org/data/gene‐symbol‐report/#!/hgnc_id/HGNC:6585, http://www.uniprot.org/uniprot/P22888	https://www.genenames.org/data/gene‐symbol‐report/#!/hgnc_id/HGNC:12373, http://www.uniprot.org/uniprot/P16473
Potency order of endogenous ligands	http://www.guidetopharmacology.org/GRAC/LigandDisplayForward?ligandId=1157 (https://www.genenames.org/data/gene‐symbol‐report/#!/hgnc_id/HGNC:1885 https://www.genenames.org/data/gene‐symbol‐report/#!/hgnc_id/HGNC:3964, http://www.uniprot.org/uniprot/P01215 http://www.uniprot.org/uniprot/P01225)	http://www.guidetopharmacology.org/GRAC/LigandDisplayForward?ligandId=1159 (https://www.genenames.org/data/gene‐symbol‐report/#!/hgnc_id/HGNC:1885 https://www.genenames.org/data/gene‐symbol‐report/#!/hgnc_id/HGNC:6584, http://www.uniprot.org/uniprot/P01215 http://www.uniprot.org/uniprot/P01229), http://www.guidetopharmacology.org/GRAC/LigandDisplayForward?ligandId=1160 (https://www.genenames.org/data/gene‐symbol‐report/#!/hgnc_id/HGNC:1885 https://www.genenames.org/data/gene‐symbol‐report/#!/hgnc_id/HGNC:1886, http://www.uniprot.org/uniprot/P01215 http://www.uniprot.org/uniprot/P01233) [http://www.ncbi.nlm.nih.gov/pubmed/1922095?dopt=AbstractPlus, http://www.ncbi.nlm.nih.gov/pubmed/12727981?dopt=AbstractPlus]	http://www.guidetopharmacology.org/GRAC/LigandDisplayForward?ligandId=3920 (https://www.genenames.org/data/gene‐symbol‐report/#!/hgnc_id/HGNC:1885 https://www.genenames.org/data/gene‐symbol‐report/#!/hgnc_id/HGNC:12372, http://www.uniprot.org/uniprot/P01215 http://www.uniprot.org/uniprot/P01222)
Labelled ligands	http://www.guidetopharmacology.org/GRAC/LigandDisplayForward?ligandId=3780 (Agonist)	http://www.guidetopharmacology.org/GRAC/LigandDisplayForward?ligandId=3793 (Agonist), [http://www.guidetopharmacology.org/GRAC/LigandDisplayForward?ligandId=3775 (Agonist)	http://www.guidetopharmacology.org/GRAC/LigandDisplayForward?ligandId=3806 (Agonist)

### Further reading on Glycoprotein hormone receptors

Jiang X *et al*. (2012) Structure of follicle‐stimulating hormone in complex with the entire ectodomain of its receptor. *Proc. Natl. Acad. Sci. U.S.A.* **109**: 12491‐6 https://www.ncbi.nlm.nih.gov/pubmed/22802634?dopt=AbstractPlus

Kleinau G *et al*. TSH receptor mutations and disease. http://www.thyroidmanager.org/chapter/tsh‐receptor‐mutations‐and‐diseases/. Accessed on 2017‐02‐23.

Tao YX *et al*. (2009) Follicle stimulating hormone receptor mutations and reproductive disorders. *Prog Mol Biol Transl Sci* **89**: 115‐31 https://www.ncbi.nlm.nih.gov/pubmed/20374735?dopt=AbstractPlus

Troppmann B *et al*. (2013) Structural and functional plasticity of the luteinizing hormone/choriogonadotrophin receptor. *Hum. Reprod. Update* **19**: 583‐602 https://www.ncbi.nlm.nih.gov/pubmed/23686864?dopt=AbstractPlus

## http://www.guidetopharmacology.org/GRAC/FamilyDisplayForward?familyId=31

### Overview

GnRH_1_ and GnRH_2_ receptors (**provisonal nomenclature** [http://www.ncbi.nlm.nih.gov/pubmed/15914470?dopt=AbstractPlus], also called Type I and Type II GnRH receptor, respectively [http://www.ncbi.nlm.nih.gov/pubmed/15082521?dopt=AbstractPlus]) have been cloned from numerous species, most of which express two or three types of GnRH receptor [http://www.ncbi.nlm.nih.gov/pubmed/16140177?dopt=AbstractPlus, http:http://www.ncbi.nlm.nih.gov/pubmed/15082521?dopt=AbstractPlus, http://www.ncbi.nlm.nih.gov/pubmed/15878963?dopt=AbstractPlus]. http://www.guidetopharmacology.org/GRAC/LigandDisplayForward?ligandId=1162 (https://www.genenames.org/data/gene‐symbol‐report/#!/hgnc_id/HGNC:4419, http://www.uniprot.org/uniprot/P01148) (p‐Glu‐His‐Trp‐Ser‐Tyr‐Gly‐LeuArg‐Pro‐Gly‐NH2) is a hypothalamic decapeptide also known as luteinizing hormone‐releasing hormone, gonadoliberin, luliberin, gonadorelin or simply as GnRH. It is a member of a family of similar peptides found in many species [http://www.ncbi.nlm.nih.gov/pubmed/16140177?dopt=AbstractPlus, http://www.ncbi.nlm.nih.gov/pubmed/15082521?dopt=AbstractPlus, http://www.ncbi.nlm.nih.gov/pubmed/15878963?dopt=AbstractPlus] including http://www.guidetopharmacology.org/GRAC/LigandDisplayForward?ligandId=1164 (https://www.genenames.org/data/gene‐symbol‐report/#!/hgnc_id/HGNC:4420, http://www.uniprot.org/uniprot/O43555) (pGlu‐His‐Trp‐Ser‐His‐Gly‐Trp‐Tyr‐Pro‐Gly‐NH_2_ (which is also known as chicken GnRH‐II). Receptors for three forms of GnRH exist in some species but only GnRH I and GnRH II and their cognate receptors have been found in mammals [http://www.ncbi.nlm.nih.gov/pubmed/16140177?dopt=AbstractPlus, http://www.ncbi.nlm.nih.gov/pubmed/15082521?dopt=AbstractPlus, http://www.ncbi.nlm.nih.gov/pubmed/15878963?dopt=AbstractPlus]. GnRH_1_ receptors are expressed by pituitary gonadotrophs, where they mediate the effects of GnRH on gonadotropin hormone synthesis and secretion that underpin central control of mammalian reproduction. GnRH analogues are used in assisted reproduction and to treat steroid hormone‐dependent conditions [http://www.ncbi.nlm.nih.gov/pubmed/12072036?dopt=AbstractPlus]. Notably, agonists cause desensitization of GnRH‐stimulated gonadotropin secretion and the consequent reduction in circulating sex steroids is exploited to treat hormone‐dependent cancers of the breast, ovary and prostate [http://www.ncbi.nlm.nih.gov/pubmed/12072036?dopt=AbstractPlus]. GnRH1 receptors are selectively activated by GnRH I and all lack the COOH‐terminal tails found in other GPCRs. GnRH_2_ receptors do have COOH‐terminal tails and (where tested) are selective for GnRH II over GnRH I. GnRH_2_ receptors are expressed by some primates but not by humans [http://www.ncbi.nlm.nih.gov/pubmed/12538601?dopt=AbstractPlus]. Phylogenetic classifications divide GnRH receptors into three [http://www.ncbi.nlm.nih.gov/pubmed/15082521?dopt=AbstractPlus] or five groups [http://www.ncbi.nlm.nih.gov/pubmed/25344287?dopt=AbstractPlus] and highlight examples of gene loss through evolution, with humans retaining only one ancient gene.

**Table:** 

Nomenclature	http://www.guidetopharmacology.org/GRAC/ObjectDisplayForward?objectId=256	http://www.guidetopharmacology.org/GRAC/ObjectDisplayForward?objectId=257
HGNC, UniProt	https://www.genenames.org/data/gene‐symbol‐report/#!/hgnc_id/HGNC:4421, http://www.uniprot.org/uniprot/P30968	https://www.genenames.org/data/gene‐symbol‐report/#!/hgnc_id/HGNC:16341, http://www.uniprot.org/uniprot/Q96P88
Potency order of endogenous ligands	http://www.guidetopharmacology.org/GRAC/LigandDisplayForward?ligandId=1162 (https://www.genenames.org/data/gene‐symbol‐report/#!/hgnc_id/HGNC:4419, http://www.uniprot.org/uniprot/P01148) > http://www.guidetopharmacology.org/GRAC/LigandDisplayForward?ligandId=1164 (https://www.genenames.org/data/gene‐symbol‐report/#!/hgnc_id/HGNC:4420, http://www.uniprot.org/uniprot/O43555) [http://www.ncbi.nlm.nih.gov/pubmed/15082521?dopt=AbstractPlus]	http://www.guidetopharmacology.org/GRAC/LigandDisplayForward?ligandId=1164 (https://www.genenames.org/data/gene‐symbol‐report/#!/hgnc_id/HGNC:4420, http://www.uniprot.org/uniprot/O43555) > http://www.guidetopharmacology.org/GRAC/LigandDisplayForward?ligandId=1162 (https://www.genenames.org/data/gene‐symbol‐report/#!/hgnc_id/HGNC:4419, http://www.uniprot.org/uniprot/P01148) (Monkey) [http://www.ncbi.nlm.nih.gov/pubmed/11493674?dopt=AbstractPlus]
Endogenous agonists	http://www.guidetopharmacology.org/GRAC/LigandDisplayForward?ligandId=1162 (https://www.genenames.org/data/gene‐symbol‐report/#!/hgnc_id/HGNC:4419, http://www.uniprot.org/uniprot/P01148) [http://www.ncbi.nlm.nih.gov/pubmed/17452338?dopt=AbstractPlus], http://www.guidetopharmacology.org/GRAC/LigandDisplayForward?ligandId=1164 (https://www.genenames.org/data/gene‐symbol‐report/#!/hgnc_id/HGNC:4420, http://www.uniprot.org/uniprot/O43555) [http://www.ncbi.nlm.nih.gov/pubmed/19638591?dopt=AbstractPlus, http://www.ncbi.nlm.nih.gov/pubmed/17452338?dopt=AbstractPlus, http://www.ncbi.nlm.nih.gov/pubmed/17942747?dopt=AbstractPlus]	http://www.guidetopharmacology.org/GRAC/LigandDisplayForward?ligandId=1164 (https://www.genenames.org/data/gene‐symbol‐report/#!/hgnc_id/HGNC:4420, http://www.uniprot.org/uniprot/O43555) [http://www.ncbi.nlm.nih.gov/pubmed/11493674?dopt=AbstractPlus] – Monkey, http://www.guidetopharmacology.org/GRAC/LigandDisplayForward?ligandId=1162 (https://www.genenames.org/data/gene‐symbol‐report/#!/hgnc_id/HGNC:4419, http://www.uniprot.org/uniprot/P01148) [http://www.ncbi.nlm.nih.gov/pubmed/11493674?dopt=AbstractPlus, http://www.ncbi.nlm.nih.gov/pubmed/15082521?dopt=AbstractPlus] – Monkey
Selective agonists	http://www.guidetopharmacology.org/GRAC/LigandDisplayForward?ligandId=3860 [http://www.ncbi.nlm.nih.gov/pubmed/26774084?dopt=AbstractPlus], http://www.guidetopharmacology.org/GRAC/LigandDisplayForward?ligandId=3860 [http://www.ncbi.nlm.nih.gov/pubmed/26398856?dopt=AbstractPlus], http://www.guidetopharmacology.org/GRAC/LigandDisplayForward?ligandId=3860 [http://www.ncbi.nlm.nih.gov/pubmed/26398856?dopt=AbstractPlus], http://www.guidetopharmacology.org/GRAC/LigandDisplayForward?ligandId=1177 [http://www.ncbi.nlm.nih.gov/pubmed/11726197?dopt=AbstractPlus], http://www.guidetopharmacology.org/GRAC/LigandDisplayForward?ligandId=1175 [http://www.ncbi.nlm.nih.gov/pubmed/17095587?dopt=AbstractPlus], http://www.guidetopharmacology.org/GRAC/LigandDisplayForward?ligandId=3879, http://www.guidetopharmacology.org/GRAC/LigandDisplayForward?ligandId=3884, http://www.guidetopharmacology.org/GRAC/LigandDisplayForward?ligandId=3902	–
Antagonists	http://www.guidetopharmacology.org/GRAC/LigandDisplayForward?ligandId=3854 (p*K*_i_ 9.5) [http://www.ncbi.nlm.nih.gov/pubmed/1850267?dopt=AbstractPlus]	–
Selective antagonists	http://www.guidetopharmacology.org/GRAC/LigandDisplayForward?ligandId=1190 (p*K*_i_ 9.3–10) [http://www.ncbi.nlm.nih.gov/pubmed/7649152?dopt=AbstractPlus, http://www.ncbi.nlm.nih.gov/pubmed/9300077?dopt=AbstractPlus, http://www.ncbi.nlm.nih.gov/pubmed/17095587?dopt=AbstractPlus], http://www.guidetopharmacology.org/GRAC/LigandDisplayForward?ligandId=1188 (p*K*_i_ 9.1–9.5) [http://www.ncbi.nlm.nih.gov/pubmed/17095587?dopt=AbstractPlus], http://www.guidetopharmacology.org/GRAC/LigandDisplayForward?ligandId=5585 (p*K*_i_ 8.8) [http://www.ncbi.nlm.nih.gov/pubmed/21188095?dopt=AbstractPlus], http://www.guidetopharmacology.org/GRAC/LigandDisplayForward?ligandId=3877	http://www.guidetopharmacology.org/GRAC/LigandDisplayForward?ligandId=3916 [http://www.ncbi.nlm.nih.gov/pubmed/14651258?dopt=AbstractPlus] – Monkey
Labelled ligands	http://www.guidetopharmacology.org/GRAC/LigandDisplayForward?ligandId=1186 (Antagonist) (p*K*_d_ 9.7) [http://www.ncbi.nlm.nih.gov/pubmed/10894158?dopt=AbstractPlus], http://www.guidetopharmacology.org/GRAC/LigandDisplayForward?ligandId=1168 (Agonist) [http://www.ncbi.nlm.nih.gov/pubmed/9484907?dopt=AbstractPlus] – Rat, [http://www.guidetopharmacology.org/GRAC/LigandDisplayForward?ligandId=1167 (Agonist) [http://www.ncbi.nlm.nih.gov/pubmed/8013367?dopt=AbstractPlus] – Rat, http://www.guidetopharmacology.org/GRAC/LigandDisplayForward?ligandId=3786 (Agonist)	–

### Comments

GnRH_1_ and GnRH_2_ receptors couple primarily to G_q/11_ [http://www.ncbi.nlm.nih.gov/pubmed/10734055?dopt=AbstractPlus] but coupling to G_s_ and G_i_ is evident in some systems [http://www.ncbi.nlm.nih.gov/pubmed/12591945?dopt=AbstractPlus, http://www.ncbi.nlm.nih.gov/pubmed/8013367?dopt=AbstractPlus]. GnRH_2_ receptors may also mediate (heterotrimeric) G protein‐independent signalling to protein kinases [http://www.ncbi.nlm.nih.gov/pubmed/15059960?dopt=AbstractPlus]. There is increasing evidence for expression of GnRH receptors on hormone‐dependent cancer cells where they can exert antiproliferative and/or proapoptotic effects and mediate effects of cytotoxins conjugated to GnRH analogues [http://www.ncbi.nlm.nih.gov/pubmed/15561800?dopt=AbstractPlus, http://www.ncbi.nlm.nih.gov/pubmed/15613448?dopt=AbstractPlus, http://www.ncbi.nlm.nih.gov/pubmed/14726258?dopt=AbstractPlus, http://www.ncbi.nlm.nih.gov/pubmed/15350601?dopt=AbstractPlus]. In some human cancer cell models http://www.guidetopharmacology.org/GRAC/LigandDisplayForward?ligandId=1164 (https://www.genenames.org/data/gene‐symbol‐report/#!/hgnc_id/HGNC:4420, http://www.uniprot.org/uniprot/O43555) is more potent than http://www.guidetopharmacology.org/GRAC/LigandDisplayForward?ligandId=1162 (https://www.genenames.org/data/gene‐symbol‐report/#!/hgnc_id/HGNC:4419, http://www.uniprot.org/uniprot/P01148), implying mediation by GnRH_2_ receptors [http://www.ncbi.nlm.nih.gov/pubmed/12237622?dopt=AbstractPlus], but GnRH_2_ receptors are not expressed by humans because the human GNRHR2 gene contains a frame shift and internal stop codon [http://www.ncbi.nlm.nih.gov/pubmed/12538601?dopt=AbstractPlus]. The possibility remains that this gene generates GnRH_2_ receptor‐related proteins (other than the full‐length receptor) that mediate responses to http://www.guidetopharmacology.org/GRAC/LigandDisplayForward?ligandId=1164 (https://www.genenames.org/data/gene‐symbol‐report/#!/hgnc_id/HGNC:4420, http://www.uniprot.org/uniprot/O43555) (see [http://www.ncbi.nlm.nih.gov/pubmed/11861490?dopt=AbstractPlus]). Alternatively, evidence for multiple active GnRH receptor conformations [http://www.ncbi.nlm.nih.gov/pubmed/15059960?dopt=AbstractPlus, http://www.ncbi.nlm.nih.gov/pubmed/22808094?dopt=AbstractPlus, http://www.ncbi.nlm.nih.gov/pubmed/20009083?dopt=AbstractPlus, http://www.ncbi.nlm.nih.gov/pubmed/15492280?dopt=AbstractPlus, http://www.ncbi.nlm.nih.gov/pubmed/15082521?dopt=AbstractPlus] raises the possibility that GnRH_1_ receptor‐mediated proliferation inhibition in hormone‐dependent cancer cells is dependent upon a conformation that couples to G_i_ rather than G_q/11_ proteins as in pituitary cells [http://www.ncbi.nlm.nih.gov/pubmed/22808094?dopt=AbstractPlus, http://www.ncbi.nlm.nih.gov/pubmed/15492280?dopt=AbstractPlus]. Loss‐of‐function mutations in the GnRH_1_ receptor and deficiency of http://www.guidetopharmacology.org/GRAC/LigandDisplayForward?ligandId=1162 (https://www.genenames.org/data/gene‐symbol‐report/#!/hgnc_id/HGNC:4419, http://www.uniprot.org/uniprot/P01148) are associated with hypogonadotropic hypogonadism although some ’loss of function’ mutations may actually prevent trafficking of ’functional’ GnRH_1_ receptors to the cell surface, as evidenced by recovery of function by nonpeptide antagonists [http://www.ncbi.nlm.nih.gov/pubmed/12843188?dopt=AbstractPlus]. Human GnRH_1_ receptors are poorly expressed at the cell surface because of failure to meet structural quality control criteria for endoplasmic reticulum exit [http://www.ncbi.nlm.nih.gov/pubmed/19888967?dopt=AbstractPlus, http://www.ncbi.nlm.nih.gov/pubmed/12843188?dopt=AbstractPlus], and this increases susceptibility to point mutations that further impair trafficking [http://www.ncbi.nlm.nih.gov/pubmed/19888967?dopt=AbstractPlus, http://www.ncbi.nlm.nih.gov/pubmed/12843188?dopt=AbstractPlus]. GnRH receptor signalling may require receptor oligomerisation [http://www.ncbi.nlm.nih.gov/pubmed/6282571?dopt=AbstractPlus, http://www.ncbi.nlm.nih.gov/pubmed/11278883?dopt=AbstractPlus].

### Further reading on Gonadotrophin‐releasing hormone receptors

Desaulniers AT *et al*. (2017) Expression and Role of Gonadotropin‐Releasing Hormone 2 and Its Receptor in Mammals. *Front Endocrinol (Lausanne)* **8**: 269 https://www.ncbi.nlm.nih.gov/pubmed/29312140?dopt=AbstractPlus

Limonta P *et al*. (2012) GnRH receptors in cancer: from cell biology to novel targeted therapeutic strategies. *Endocr. Rev.* **33**: 784‐811 https://www.ncbi.nlm.nih.gov/pubmed/22778172?dopt=AbstractPlus

McArdle CA and Roberson MS.. (2015) Gonadotropes and gonadotropin‐releasing hormone signaling. *In Knobil and Neill's Physiology of Reproduction (4th edition).* Edited by Plant TM and Zeleznik AJ.: Elsevier Inc.: [ISBN: 9780123971753]

Millar RP *et al*. (2004) Gonadotropin‐releasing hormone receptors. *Endocr Rev* **25**: 235‐275 https://www.ncbi.nlm.nih.gov/pubmed/15082521?dopt=AbstractPlus

Tao YX *et al*. (2014) Chaperoning G protein‐coupled receptors: from cell biology to therapeutics. *Endocr. Rev.* **35**: 602‐47 https://www.ncbi.nlm.nih.gov/pubmed/24661201?dopt=AbstractPlus

## http://www.guidetopharmacology.org/GRAC/FamilyDisplayForward?familyId=114

### Overview

GPR18, GPR55 and GPR119 (**provisional nomenclature**), although showing little structural similarity to CB_1_ and CB_2_ cannabinoid receptors, respond to endogenous agents analogous to the endogenous cannabinoid ligands, as well as some natural/synthetic cannabinoid receptor ligands [http://www.ncbi.nlm.nih.gov/pubmed/21079038?dopt=AbstractPlus]. Although there are multiple reports to indicate that GPR18, GPR55 and GPR119 can be activated *in vitro* by http://www.guidetopharmacology.org/GRAC/LigandDisplayForward?ligandId=3635, http://www.guidetopharmacology.org/GRAC/LigandDisplayForward?ligandId=4028 and http://www.guidetopharmacology.org/GRAC/LigandDisplayForward?ligandId=2661, respectively, there is a lack of evidence for activation by these lipid messengers *in vivo*. As such, therefore, these receptors retain their orphan status.

**Table:** 

Nomenclature	http://www.guidetopharmacology.org/GRAC/ObjectDisplayForward?objectId=89	http://www.guidetopharmacology.org/GRAC/ObjectDisplayForward?objectId=109	http://www.guidetopharmacology.org/GRAC/ObjectDisplayForward?objectId=126
HGNC, UniProt	https://www.genenames.org/data/gene‐symbol‐report/#!/hgnc_id/HGNC:4472, http://www.uniprot.org/uniprot/Q14330	https://www.genenames.org/data/gene‐symbol‐report/#!/hgnc_id/HGNC:4511, http://www.uniprot.org/uniprot/Q9Y2T6	https://www.genenames.org/data/gene‐symbol‐report/#!/hgnc_id/HGNC:19060, http://www.uniprot.org/uniprot/Q8TDV5
Potency order of endogenous ligands	–	–	http://www.guidetopharmacology.org/GRAC/LigandDisplayForward?ligandId=2661, http://www.guidetopharmacology.org/GRAC/LigandDisplayForward?ligandId=3622 > http://www.guidetopharmacology.org/GRAC/LigandDisplayForward?ligandId=3621 (http://www.guidetopharmacology.org/GRAC/LigandDisplayForward?ligandId=2364 is ineffective) [http://www.ncbi.nlm.nih.gov/pubmed/16517404?dopt=AbstractPlus]
Endogenous agonists	http://www.guidetopharmacology.org/GRAC/LigandDisplayForward?ligandId=3635 [http://www.ncbi.nlm.nih.gov/pubmed/16844083?dopt=AbstractPlus]	http://www.guidetopharmacology.org/GRAC/LigandDisplayForward?ligandId=4028 [http://www.ncbi.nlm.nih.gov/pubmed/20136841?dopt=AbstractPlus, http://www.ncbi.nlm.nih.gov/pubmed/17765871?dopt=AbstractPlus, http://www.ncbi.nlm.nih.gov/pubmed/23396314?dopt=AbstractPlus], http://www.guidetopharmacology.org/GRAC/LigandDisplayForward?ligandId=4029 [http://www.ncbi.nlm.nih.gov/pubmed/18845565?dopt=AbstractPlus]	http://www.guidetopharmacology.org/GRAC/LigandDisplayForward?ligandId=2661 [http://www.ncbi.nlm.nih.gov/pubmed/19901198?dopt=AbstractPlus, http://www.ncbi.nlm.nih.gov/pubmed/16517404?dopt=AbstractPlus, http://www.ncbi.nlm.nih.gov/pubmed/23396314?dopt=AbstractPlus], http://www.guidetopharmacology.org/GRAC/LigandDisplayForward?ligandId=3622, http://www.guidetopharmacology.org/GRAC/LigandDisplayForward?ligandId=3621
Selective agonists	–	http://www.guidetopharmacology.org/GRAC/LigandDisplayForward?ligandId=3317 [http://www.ncbi.nlm.nih.gov/pubmed/20136841?dopt=AbstractPlus, http://www.ncbi.nlm.nih.gov/pubmed/19723626?dopt=AbstractPlus, http://www.ncbi.nlm.nih.gov/pubmed/17876302?dopt=AbstractPlus]	http://www.guidetopharmacology.org/GRAC/LigandDisplayForward?ligandId=4027 [http://www.ncbi.nlm.nih.gov/pubmed/20804735?dopt=AbstractPlus], http://www.guidetopharmacology.org/GRAC/LigandDisplayForward?ligandId=3319 [http://www.ncbi.nlm.nih.gov/pubmed/16517404?dopt=AbstractPlus], http://www.guidetopharmacology.org/GRAC/LigandDisplayForward?ligandId=3318 [http://www.ncbi.nlm.nih.gov/pubmed/16517404?dopt=AbstractPlus]
Selective antagonists	–	http://www.guidetopharmacology.org/GRAC/LigandDisplayForward?ligandId=6577 (apparent pA_2_) (p*A*_2_ 7.3) [http://www.ncbi.nlm.nih.gov/pubmed/23639801?dopt=AbstractPlus], http://www.guidetopharmacology.org/GRAC/LigandDisplayForward?ligandId=9967 (plC_50_ 6.7) [http://www.ncbi.nlm.nih.gov/pubmed/22091481?dopt=AbstractPlus]	–
Comments	The pairing of http://www.guidetopharmacology.org/GRAC/LigandDisplayForward?ligandId=3635 with GPR18 was not replicated in two studies based on arrestin assays [http://www.ncbi.nlm.nih.gov/pubmed/23396314?dopt=AbstractPlus, http://www.ncbi.nlm.nih.gov/pubmed/19286662?dopt=AbstractPlus]. See [http://www.ncbi.nlm.nih.gov/pubmed/23686350?dopt=AbstractPlus] for discussion.	See reviews [http://www.ncbi.nlm.nih.gov/pubmed/23686350?dopt=AbstractPlus] and [http://www.ncbi.nlm.nih.gov/pubmed/25926795?dopt=AbstractPlus].	In addition to those shown above, further small molecule agonists have been reported [http://www.ncbi.nlm.nih.gov/pubmed/26048791?dopt=AbstractPlus].

### Comments

GPR18 failed to respond to a variety of lipid‐derived agents in an *in vitro* screen [http://www.ncbi.nlm.nih.gov/pubmed/19286662?dopt=AbstractPlus], but has been reported to be activated by http://www.guidetopharmacology.org/GRAC/LigandDisplayForward?ligandId=2424 [http://www.ncbi.nlm.nih.gov/pubmed/21595653?dopt=AbstractPlus]. GPR55 responds to http://www.guidetopharmacology.org/GRAC/LigandDisplayForward?ligandId=3317 and http://www.guidetopharmacology.org/GRAC/LigandDisplayForward?ligandId=743 at micromolar concentrations, compared to their nanomolar affinity as CB_1_ receptor antagonists/inverse agonists [http://www.ncbi.nlm.nih.gov/pubmed/21079038?dopt=AbstractPlus]. It has been reported that http://www.guidetopharmacology.org/GRAC/LigandDisplayForward?ligandId=4028 acts at other sites in addition to GPR55 [http://www.ncbi.nlm.nih.gov/pubmed/23714700?dopt=AbstractPlus]. N‐Arachidonoylserine has been suggested to act as a low efficacy agonist/antagonist at GPR18 *in vitro* [http://www.ncbi.nlm.nih.gov/pubmed/20346144?dopt=AbstractPlus]. It has also been suggested http://www.guidetopharmacology.org/GRAC/LigandDisplayForward?ligandId=3623 acts, at least in part, through GPR119 [http://www.ncbi.nlm.nih.gov/pubmed/18724386?dopt=AbstractPlus]. Although http://www.guidetopharmacology.org/GRAC/LigandDisplayForward?ligandId=3318 and http://www.guidetopharmacology.org/GRAC/LigandDisplayForward?ligandId=3319 produce GPR119‐dependent responses in heterologous expression systems, comparison with http://www.guidetopharmacology.org/GRAC/LigandDisplayForward?ligandId=2661‐mediated responses suggests additional mechanisms of action [http://www.ncbi.nlm.nih.gov/pubmed/18724386?dopt=AbstractPlus].

### Further reading on GPR18, GPR55 and GPR119

Davenport AP *et al*. (2013) International Union of Basic and Clinical Pharmacology. LXXXVIII. G protein‐coupled receptor list: recommendations for new pairings with cognate ligands. *Pharmacol. Rev.* **65**: 967‐86 https://www.ncbi.nlm.nih.gov/pubmed/23686350?dopt=AbstractPlus

Hassing HA *et al*. (2016) Biased signaling of lipids and allosteric actions of synthetic molecules for GPR119. *Biochem. Pharmacol.* **119**: 66‐75 https://www.ncbi.nlm.nih.gov/pubmed/27569424?dopt=AbstractPlus

Irving A *et al*. (2017) Cannabinoid Receptor‐Related Orphan G Protein‐Coupled Receptors. *Adv Pharmacol* **80**: 223‐247 https://www.ncbi.nlm.nih.gov/pubmed/28826536

Liu B *et al*. (2015) GPR55: from orphan to metabolic regulator? *Pharmacol. Ther.* **145**: 35‐42 https://www.ncbi.nlm.nih.gov/pubmed/24972076?dopt=AbstractPlus

Pertwee RG *et al*. (2010) International Union of Basic and Clinical Pharmacology. LXXIX. Cannabinoid receptors and their ligands: beyond CB_1 and CB_2. *Pharmacol. Rev.* **62**: 588‐631 https://www.ncbi.nlm.nih.gov/pubmed/21079038?dopt=AbstractPlus

## http://www.guidetopharmacology.org/GRAC/FamilyDisplayForward?familyId=33

### Overview

Histamine receptors (**nomenclature as agreed by the NC‐IUPHAR Subcommittee on Histamine Receptors** [http://www.ncbi.nlm.nih.gov/pubmed/9311023?dopt=AbstractPlus, http://www.ncbi.nlm.nih.gov/pubmed/26084539?dopt=AbstractPlus]) are activated by the endogenous ligand http://www.guidetopharmacology.org/GRAC/LigandDisplayForward?ligandId=1204. Marked species differences exist between histamine receptor orthologues [http://www.ncbi.nlm.nih.gov/pubmed/9311023?dopt=AbstractPlus]. The human and rat H_3_ receptor genes are subject to significant splice variance [http://www.ncbi.nlm.nih.gov/pubmed/16415177?dopt=AbstractPlus]. The potency order of histamine at histamine receptor subtypes is H_3_ = H_4_ > H_2_ > H_1_ [http://www.ncbi.nlm.nih.gov/pubmed/26084539?dopt=AbstractPlus]. Some agonists at the human H_3_ receptor display significant ligand bias [http://www.ncbi.nlm.nih.gov/pubmed/27864425?dopt=AbstractPlus]. Antagonists of all 4 histamine receptors have clinical uses: H_1_ antagonists for allergies (*e.g.* http://www.guidetopharmacology.org/GRAC/LigandDisplayForward?ligandId=1222), H_2_ antagonists for acid‐reflux diseases (*e.g.* http://www.guidetopharmacology.org/GRAC/LigandDisplayForward?ligandId=1234), H_3_ antagonists for narcolepsy (*e.g.* http://www.guidetopharmacology.org/GRAC/LigandDisplayForward?ligandId=8924/WAKIX; Registered) and H_4_ antagonists for atopic dermatitis (*e.g.* http://www.guidetopharmacology.org/GRAC/LigandDisplayForward?ligandId=8985; Phase IIa) [http://www.ncbi.nlm.nih.gov/pubmed/26084539?dopt=AbstractPlus] and vestibular neuritis (AUV) (SENS‐111 (Seliforant, previously UR‐63325), entered and completed vestibular neuritis (AUV) Phase IIa efficacy and safety trials, respectively) [http://www.ncbi.nlm.nih.gov/pubmed/27673668?dopt=AbstractPlus, http://www.ncbi.nlm.nih.gov/pubmed/30152527?dopt=AbstractPlus].

**Table:** 

Nomenclature	http://www.guidetopharmacology.org/GRAC/ObjectDisplayForward?objectId=262	http://www.guidetopharmacology.org/GRAC/ObjectDisplayForward?objectId=263
HGNC, UniProt	https://www.genenames.org/data/gene‐symbol‐report/#!/hgnc_id/HGNC:5182, http://www.uniprot.org/uniprot/P35367	https://www.genenames.org/data/gene‐symbol‐report/#!/hgnc_id/HGNC:5183, http://www.uniprot.org/uniprot/P25021
Selective agonists	http://www.guidetopharmacology.org/GRAC/LigandDisplayForward?ligandId=1205 [http://www.ncbi.nlm.nih.gov/pubmed/12626648?dopt=AbstractPlus], http://www.guidetopharmacology.org/GRAC/LigandDisplayForward?ligandId=4026 [http://www.ncbi.nlm.nih.gov/pubmed/15947036?dopt=AbstractPlus]	http://www.guidetopharmacology.org/GRAC/LigandDisplayForward?ligandId=4025 [http://www.ncbi.nlm.nih.gov/pubmed/19072936?dopt=AbstractPlus]
Antagonists	http://www.guidetopharmacology.org/GRAC/LigandDisplayForward?ligandId=277 (p*K*_i_ 10.2) [http://www.ncbi.nlm.nih.gov/pubmed/7925364?dopt=AbstractPlus], http://www.guidetopharmacology.org/GRAC/LigandDisplayForward?ligandId=7282 (p*K*_i_ 9.6) [http://www.ncbi.nlm.nih.gov/pubmed/16782354?dopt=AbstractPlus], http://www.guidetopharmacology.org/GRAC/LigandDisplayForward?ligandId=1227 (Inverse agonist) (p*K*_i_ 8.7–9) [http://www.ncbi.nlm.nih.gov/pubmed/12065734?dopt=AbstractPlus, http://www.ncbi.nlm.nih.gov/pubmed/15206929?dopt=AbstractPlus], http://www.guidetopharmacology.org/GRAC/LigandDisplayForward?ligandId=1222 (Inverse agonist) (p*K*_i_ 8.2) [http://www.ncbi.nlm.nih.gov/pubmed/7925364?dopt=AbstractPlus], http://www.guidetopharmacology.org/GRAC/LigandDisplayForward?ligandId=1224 (p*K*_i_ 7.9) [http://www.ncbi.nlm.nih.gov/pubmed/12065734?dopt=AbstractPlus]	–
Selective antagonists	http://www.guidetopharmacology.org/GRAC/LigandDisplayForward?ligandId=6063 (p*K*_i_ 10.3) [79], http://www.guidetopharmacology.org/GRAC/LigandDisplayForward?ligandId=7157 (p*K*_i_ 9) [http://www.ncbi.nlm.nih.gov/pubmed/15482930?dopt=AbstractPlus], http://www.guidetopharmacology.org/GRAC/LigandDisplayForward?ligandId=1228 (p*K*_i_ 8.5–9) [http://www.ncbi.nlm.nih.gov/pubmed/12065734?dopt=AbstractPlus, http://www.ncbi.nlm.nih.gov/pubmed/7925364?dopt=AbstractPlus], http://www.guidetopharmacology.org/GRAC/LigandDisplayForward?ligandId=7121 (p*K*_i_ 8.9) [http://www.ncbi.nlm.nih.gov/pubmed/21381763?dopt=AbstractPlus], http://www.guidetopharmacology.org/GRAC/LigandDisplayForward?ligandId=2603 (p*K*_i_ 8.5) [http://www.ncbi.nlm.nih.gov/pubmed/12747773?dopt=AbstractPlus]	http://www.guidetopharmacology.org/GRAC/LigandDisplayForward?ligandId=1235 (p*K*_i_ 7.5) [http://www.ncbi.nlm.nih.gov/pubmed/9384502?dopt=AbstractPlus] – Rat, http://www.guidetopharmacology.org/GRAC/LigandDisplayForward?ligandId=1234 (p*K*_i_ 7.1) [http://www.ncbi.nlm.nih.gov/pubmed/7921611?dopt=AbstractPlus], http://www.guidetopharmacology.org/GRAC/LigandDisplayForward?ligandId=1231 (p*K*_i_ 6.8) [http://www.ncbi.nlm.nih.gov/pubmed/23466604?dopt=AbstractPlus]
Labelled ligands	http://www.guidetopharmacology.org/GRAC/LigandDisplayForward?ligandId=1220 (Antagonist, Inverse agonist) (p*K*_d_ 8.4–9.1) [http://www.ncbi.nlm.nih.gov/pubmed/9794809?dopt=AbstractPlus, http://www.ncbi.nlm.nih.gov/pubmed/7925364?dopt=AbstractPlus, http://www.ncbi.nlm.nih.gov/pubmed/8935801?dopt=AbstractPlus, http://www.ncbi.nlm.nih.gov/pubmed/12626648?dopt=AbstractPlus], http://www.guidetopharmacology.org/GRAC/LigandDisplayForward?ligandId=3958 (Antagonist) (p*K*_d_ 9) [http://www.ncbi.nlm.nih.gov/pubmed/15093820?dopt=AbstractPlus], http://www.guidetopharmacology.org/GRAC/LigandDisplayForward?ligandId=3957 (Antagonist, Inverse agonist)	http://www.guidetopharmacology.org/GRAC/LigandDisplayForward?ligandId=1230 (Antagonist) (p*K*_d_ 8.7) [http://www.ncbi.nlm.nih.gov/pubmed/8961278?dopt=AbstractPlus] – Rat, http://www.guidetopharmacology.org/GRAC/LigandDisplayForward?ligandId=3959 (Antagonist) (p*K*_d_ 7.7–8.7) [http://www.ncbi.nlm.nih.gov/pubmed/12869657?dopt=AbstractPlus]

**Table:** 

Nomenclature	http://www.guidetopharmacology.org/GRAC/ObjectDisplayForward?objectId=264	http://www.guidetopharmacology.org/GRAC/ObjectDisplayForward?objectId=265
HGNC, UniProt	https://www.genenames.org/data/gene‐symbol‐report/#!/hgnc_id/HGNC:5184, http://www.uniprot.org/uniprot/Q9Y5N1	https://www.genenames.org/data/gene‐symbol‐report/#!/hgnc_id/HGNC:17383, http://www.uniprot.org/uniprot/Q9H3N8
Selective agonists	http://www.guidetopharmacology.org/GRAC/LigandDisplayForward?ligandId=4024 [http://www.ncbi.nlm.nih.gov/pubmed/15115383?dopt=AbstractPlus], http://www.guidetopharmacology.org/GRAC/LigandDisplayForward?ligandId=1254 [http://www.ncbi.nlm.nih.gov/pubmed/15771452?dopt=AbstractPlus], http://www.guidetopharmacology.org/GRAC/LigandDisplayForward?ligandId=7346 (Inverse agonist) [http://www.ncbi.nlm.nih.gov/pubmed/18598020?dopt=AbstractPlus]	http://www.guidetopharmacology.org/GRAC/LigandDisplayForward?ligandId=1223 (Partial agonist) [http://www.ncbi.nlm.nih.gov/pubmed/12606603?dopt=AbstractPlus, http://www.ncbi.nlm.nih.gov/pubmed/15947036?dopt=AbstractPlus, http://www.ncbi.nlm.nih.gov/pubmed/11179434?dopt=AbstractPlus, http://www.ncbi.nlm.nih.gov/pubmed/11561071?dopt=AbstractPlus, http://www.ncbi.nlm.nih.gov/pubmed/11181941?dopt=AbstractPlus], http://www.guidetopharmacology.org/GRAC/LigandDisplayForward?ligandId=1269 [http://www.ncbi.nlm.nih.gov/pubmed/16432504?dopt=AbstractPlus, http://www.ncbi.nlm.nih.gov/pubmed/15947036?dopt=AbstractPlus], http://www.guidetopharmacology.org/GRAC/LigandDisplayForward?ligandId=8983 [http://www.ncbi.nlm.nih.gov/pubmed/26084539?dopt=AbstractPlus], http://www.guidetopharmacology.org/GRAC/LigandDisplayForward?ligandId=1274 [http://www.ncbi.nlm.nih.gov/pubmed/17154494?dopt=AbstractPlus]
Antagonists	http://www.guidetopharmacology.org/GRAC/LigandDisplayForward?ligandId=1266 (p*K*_i_ 8.2–8.7) [http://www.ncbi.nlm.nih.gov/pubmed/11714875?dopt=AbstractPlus, http://www.ncbi.nlm.nih.gov/pubmed/12393057?dopt=AbstractPlus]	–
Selective antagonists	http://www.guidetopharmacology.org/GRAC/LigandDisplayForward?ligandId=8924 (p*K*_i_ 8.1–8.6) [http://www.ncbi.nlm.nih.gov/pubmed/26084539?dopt=AbstractPlus, http://www.ncbi.nlm.nih.gov/pubmed/19329325?dopt=AbstractPlus], http://www.guidetopharmacology.org/GRAC/LigandDisplayForward?ligandId=4023 (p*K*_i_ 8.5) [http://www.ncbi.nlm.nih.gov/pubmed/15033391?dopt=AbstractPlus], http://www.guidetopharmacology.org/GRAC/LigandDisplayForward?ligandId=8981 (p*K*_i_ 8.3) [http://www.ncbi.nlm.nih.gov/pubmed/26084539?dopt=AbstractPlus], http://www.guidetopharmacology.org/GRAC/LigandDisplayForward?ligandId=7346 (p*K*_i_ 8.2) [http://www.ncbi.nlm.nih.gov/pubmed/26084539?dopt=AbstractPlus], http://www.guidetopharmacology.org/GRAC/LigandDisplayForward?ligandId=1267 (Selective for H_3_/H_4_ compared to H_1_ and H_3_.) (p*K*_i_ 7.1–7.7) [http://www.ncbi.nlm.nih.gov/pubmed/11284713?dopt=AbstractPlus, http://www.ncbi.nlm.nih.gov/pubmed/15294456?dopt=AbstractPlus, http://www.ncbi.nlm.nih.gov/pubmed/12606603?dopt=AbstractPlus, http://www.ncbi.nlm.nih.gov/pubmed/11090094?dopt=AbstractPlus, http://www.ncbi.nlm.nih.gov/pubmed/10869375?dopt=AbstractPlus, http://www.ncbi.nlm.nih.gov/pubmed/11714875?dopt=AbstractPlus, http://www.ncbi.nlm.nih.gov/pubmed/12393057?dopt=AbstractPlus], http://www.guidetopharmacology.org/GRAC/LigandDisplayForward?ligandId=1265 (p*K*_i_ 6.7–7.3) [http://www.ncbi.nlm.nih.gov/pubmed/11284713?dopt=AbstractPlus, http://www.ncbi.nlm.nih.gov/pubmed/15294456?dopt=AbstractPlus, http://www.ncbi.nlm.nih.gov/pubmed/12606603?dopt=AbstractPlus, http://www.ncbi.nlm.nih.gov/pubmed/11090094?dopt=AbstractPlus, http://www.ncbi.nlm.nih.gov/pubmed/26084539?dopt=AbstractPlus, http://www.ncbi.nlm.nih.gov/pubmed/12393057?dopt=AbstractPlus]	http://www.guidetopharmacology.org/GRAC/LigandDisplayForward?ligandId=8985 (p*K*_i_ 8.3) [http://www.ncbi.nlm.nih.gov/pubmed/26084539?dopt=AbstractPlus], http://www.guidetopharmacology.org/GRAC/LigandDisplayForward?ligandId=8982 (p*K*_i_ 8.3) [http://www.ncbi.nlm.nih.gov/pubmed/26084539?dopt=AbstractPlus], http://www.guidetopharmacology.org/GRAC/LigandDisplayForward?ligandId=1278 (p*K*_i_ 7.8–8.3) [http://www.ncbi.nlm.nih.gov/pubmed/15947036?dopt=AbstractPlus, http://www.ncbi.nlm.nih.gov/pubmed/16854056?dopt=AbstractPlus, http://www.ncbi.nlm.nih.gov/pubmed/14722321?dopt=AbstractPlus], http://www.guidetopharmacology.org/GRAC/LigandDisplayForward?ligandId=8984 (p*K*_i_ 7.9) [http://www.ncbi.nlm.nih.gov/pubmed/26084539?dopt=AbstractPlus, http://www.ncbi.nlm.nih.gov/pubmed/24495018?dopt=AbstractPlus], http://www.guidetopharmacology.org/GRAC/LigandDisplayForward?ligandId=1267 (Selective for H_3_/H_4_ compared to H_1_ and H_3_.) (p*K*_i_ 6.3–7.6) [http://www.ncbi.nlm.nih.gov/pubmed/15294456?dopt=AbstractPlus, http://www.ncbi.nlm.nih.gov/pubmed/12606603?dopt=AbstractPlus, http://www.ncbi.nlm.nih.gov/pubmed/11179434?dopt=AbstractPlus, http://www.ncbi.nlm.nih.gov/pubmed/11561071?dopt=AbstractPlus, http://www.ncbi.nlm.nih.gov/pubmed/11181941?dopt=AbstractPlus, http://www.ncbi.nlm.nih.gov/pubmed/11179436?dopt=AbstractPlus]
Labelled ligands	http://www.guidetopharmacology.org/GRAC/LigandDisplayForward?ligandId=1264 (Antagonist) (p*K*_d_ 10.2) [http://www.ncbi.nlm.nih.gov/pubmed/11090094?dopt=AbstractPlus], http://www.guidetopharmacology.org/GRAC/LigandDisplayForward?ligandId=3960 (Antagonist) (p*K*_d_ 9.2) [http://www.ncbi.nlm.nih.gov/pubmed/7834183?dopt=AbstractPlus] – Rat, http://www.guidetopharmacology.org/GRAC/LigandDisplayForward?ligandId=1237 (Agonist) [http://www.ncbi.nlm.nih.gov/pubmed/11179434?dopt=AbstractPlus], http://www.guidetopharmacology.org/GRAC/LigandDisplayForward?ligandId=1240 (Agonist) [http://www.ncbi.nlm.nih.gov/pubmed/12706455?dopt=AbstractPlus] – Mouse	http://www.guidetopharmacology.org/GRAC/LigandDisplayForward?ligandId=1279 (Antagonist) (p*K*_d_ 8.4) [http://www.ncbi.nlm.nih.gov/pubmed/14722321?dopt=AbstractPlus]

### Comments

http://www.guidetopharmacology.org/GRAC/LigandDisplayForward?ligandId=4026 and http://www.guidetopharmacology.org/GRAC/LigandDisplayForward?ligandId=1205 are reduced efficacy agonists. The H_4_ receptor appears to exhibit broadly similar pharmacology to the H_3_ receptor for imidazole‐containing ligands, although http://www.guidetopharmacology.org/GRAC/LigandDisplayForward?ligandId=1236 and http://www.guidetopharmacology.org/GRAC/LigandDisplayForward?ligandId=1239 are less potent, while http://www.guidetopharmacology.org/GRAC/LigandDisplayForward?ligandId=1223 acts as a reduced efficacy agonist at the H_4_ receptor and an antagonist at the H_3_ receptor [http://www.ncbi.nlm.nih.gov/pubmed/11179434?dopt=AbstractPlus, http://www.ncbi.nlm.nih.gov/pubmed/11118334?dopt=AbstractPlus, http://www.ncbi.nlm.nih.gov/pubmed/11179435?dopt=AbstractPlus, http://www.ncbi.nlm.nih.gov/pubmed/10973974?dopt=AbstractPlus, http://www.ncbi.nlm.nih.gov/pubmed/11179436?dopt=AbstractPlus]. Moreover, http://www.guidetopharmacology.org/GRAC/LigandDisplayForward?ligandId=1269 is identified as a high affinity, full agonist for the human H_4_ receptor [http://www.ncbi.nlm.nih.gov/pubmed/15947036?dopt=AbstractPlus]. http://www.guidetopharmacology.org/GRAC/LigandDisplayForward?ligandId=1247 has been used to label the H_4_ receptor heterologous expression systems.

### Further reading on Histamine receptors

Gbahou F *et al*. (2012) The histamine autoreceptor is a short isoform of the H_3 receptor. *Br. J.* *Pharmacol.* **166**: 1860‐71 https://www.ncbi.nlm.nih.gov/pubmed/22356432?dopt=AbstractPlus

Nieto‐Alamilla G *et al*. (2016) The Histamine H3 Receptor: Structure, Pharmacology, and Function. *Mol. Pharmacol.* **90**: 649‐673 https://www.ncbi.nlm.nih.gov/pubmed/27563055?dopt=AbstractPlus

Panula P *et al*. (2015) International Union of Basic and Clinical Pharmacology. XCVIII. Histamine Receptors. *Pharmacol. Rev.* **67**: 601‐55 https://www.ncbi.nlm.nih.gov/pubmed/26084539?dopt=AbstractPlus

van Rijn RM *et al*. (2008) Cloning and characterization of dominant negative splice variants of the human histamine H4 receptor. *Biochem. J.* **414**: 121‐31 [https://www.ncbi.nlm.nih.gov/pubmed/18452403?dopt=AbstractPlus]

## http://www.guidetopharmacology.org/GRAC/FamilyDisplayForward?familyId=48

### Overview

The hydroxycarboxylic acid family of receptors (http://www.ensembl.org/Homo_sapiens/Gene/Family/Genes?family=ENSFM00500000271913, **nomenclature as agreed by the** **NC‐IUPHAR** **Subcommittee on Hydroxycarboxylic acid receptors** [http://www.ncbi.nlm.nih.gov/pubmed/23686350?dopt=AbstractPlus, http://www.ncbi.nlm.nih.gov/pubmed/21454438?dopt=AbstractPlus]) respond to organic acids, including the endogenous hydroxy carboxylic acids 3‐hydroxy butyric acid and http://www.guidetopharmacology.org/GRAC/LigandDisplayForward?ligandId=2932, as well as the lipid lowering agents http://www.guidetopharmacology.org/GRAC/LigandDisplayForward?ligandId=1588 (niacin), http://www.guidetopharmacology.org/GRAC/LigandDisplayForward?ligandId=1596 and http://www.guidetopharmacology.org/GRAC/LigandDisplayForward?ligandId=1595 [http://www.ncbi.nlm.nih.gov/pubmed/12646212?dopt=AbstractPlus, http://www.ncbi.nlm.nih.gov/pubmed/12563315?dopt=AbstractPlus, http://www.ncbi.nlm.nih.gov/pubmed/12522134?dopt=AbstractPlus]. These receptors were provisionally described as nicotinic acid receptors, although nicotinic acid shows submicromolar potency at HCA_2_ receptors only and is unlikely to be the natural ligand [http://www.ncbi.nlm.nih.gov/pubmed/12563315?dopt=AbstractPlus, https://www.ncbi.nlm.nih.gov/pubmed/12522134?dopt=AbstractPlus].

**Table:** 

Nomenclature	http://www.guidetopharmacology.org/GRAC/ObjectDisplayForward?objectId=311	http://www.guidetopharmacology.org/GRAC/ObjectDisplayForward?objectId=312	http://www.guidetopharmacology.org/GRAC/ObjectDisplayForward?objectId=313
HGNC, UniProt	https://www.genenames.org/data/gene‐symbol‐report/#!/hgnc_id/HGNC:4532, http://www.uniprot.org/uniprot/Q9BXC0	https://www.genenames.org/data/gene‐symbol‐report/#!/hgnc_id/HGNC:24827, http://www.uniprot.org/uniprot/Q8TDS4	https://www.genenames.org/data/gene‐symbol‐report/#!/hgnc_id/HGNC:16824, http://www.uniprot.org/uniprot/P49019
Potency order of endogenous ligands	–	http://www.guidetopharmacology.org/GRAC/LigandDisplayForward?ligandId=1593 > http://www.guidetopharmacology.org/GRAC/LigandDisplayForward?ligandId=1059	–
Endogenous agonists	http://www.guidetopharmacology.org/GRAC/LigandDisplayForward?ligandId=2932 [http://www.ncbi.nlm.nih.gov/pubmed/20374963?dopt=AbstractPlus, http://www.ncbi.nlm.nih.gov/pubmed/18952058?dopt=AbstractPlus, http://www.ncbi.nlm.nih.gov/pubmed/19047060?dopt=AbstractPlus, http://www.ncbi.nlm.nih.gov/pubmed/23396314?dopt=AbstractPlus]	http://www.guidetopharmacology.org/GRAC/LigandDisplayForward?ligandId=1593 [http://www.ncbi.nlm.nih.gov/pubmed/15929991?dopt=AbstractPlus], http://www.guidetopharmacology.org/GRAC/LigandDisplayForward?ligandId=1059	http://www.guidetopharmacology.org/GRAC/LigandDisplayForward?ligandId=2933 [http://www.ncbi.nlm.nih.gov/pubmed/19561068?dopt=AbstractPlus]
Agonists	http://www.guidetopharmacology.org/GRAC/LigandDisplayForward?ligandId=8467 [http://www.ncbi.nlm.nih.gov/pubmed/24486398?dopt=AbstractPlus], http://www.guidetopharmacology.org/GRAC/LigandDisplayForward?ligandId=5783 [http://www.ncbi.nlm.nih.gov/pubmed/22434674?dopt=AbstractPlus]	http://www.guidetopharmacology.org/GRAC/LigandDisplayForward?ligandId=8469 [http://www.ncbi.nlm.nih.gov/pubmed/24900372?dopt=AbstractPlus], http://www.guidetopharmacology.org/GRAC/LigandDisplayForward?ligandId=8470 [http://www.ncbi.nlm.nih.gov/pubmed/25773497?dopt=AbstractPlus]	–
Selective agonists	–	http://www.guidetopharmacology.org/GRAC/LigandDisplayForward?ligandId=5788 [http://www.ncbi.nlm.nih.gov/pubmed/20184326?dopt=AbstractPlus], http://www.guidetopharmacology.org/GRAC/LigandDisplayForward?ligandId=5785 [http://www.ncbi.nlm.nih.gov/pubmed/22435740?dopt=AbstractPlus], http://www.guidetopharmacology.org/GRAC/LigandDisplayForward?ligandId=1588 [http://www.ncbi.nlm.nih.gov/pubmed/12646212?dopt=AbstractPlus, http://www.ncbi.nlm.nih.gov/pubmed/12563315?dopt=AbstractPlus, http://www.ncbi.nlm.nih.gov/pubmed/12522134?dopt=AbstractPlus], http://www.guidetopharmacology.org/GRAC/LigandDisplayForward?ligandId=1596 [https://www.ncbi.nlm.nih.gov/pubmed/12646212?dopt=AbstractPlus, http://www.ncbi.nlm.nih.gov/pubmed/12522134?dopt=AbstractPlus], http://www.guidetopharmacology.org/GRAC/LigandDisplayForward?ligandId=5786 [http://www.ncbi.nlm.nih.gov/pubmed/18722346?dopt=AbstractPlus]	http://www.guidetopharmacology.org/GRAC/LigandDisplayForward?ligandId=5800 [http://www.ncbi.nlm.nih.gov/pubmed/19524438?dopt=AbstractPlus], http://www.guidetopharmacology.org/GRAC/LigandDisplayForward?ligandId=1597 [http://www.ncbi.nlm.nih.gov/pubmed/16480258?dopt=AbstractPlus]
Labelled ligands	–	http://www.guidetopharmacology.org/GRAC/LigandDisplayForward?ligandId=1594 (Agonist) [http://www.ncbi.nlm.nih.gov/pubmed/12646212?dopt=AbstractPlus, http://www.ncbi.nlm.nih.gov/pubmed/12563315?dopt=AbstractPlus, http://www.ncbi.nlm.nih.gov/pubmed/12522134?dopt=AbstractPlus]	–

### Comments

Further closely‐related GPCRs include the http://www.guidetopharmacology.org/GRAC/FamilyDisplayForward?familyId=35 (https://www.genenames.org/data/gene‐symbol‐report/#!/hgnc_id/HGNC:24884, http://www.uniprot.org/uniprot/Q8TDS5)and https://www.genenames.org/data/gene‐symbol‐report/#!/hgnc_id/HGNC:4486 (http://www.uniprot.org/uniprot/O00270). Lactate activates HCA_1_ on adipocytes in an autocrine manner. It inhibits lipolysis and thereby promotes anabolic effects. HCA_2_ and HCA_3_ regulate adipocyte lipolysis and immune functions under conditions of increased FFA formation through lipolysis (e.g., during fasting). HCA_2_ agonists acting mainly through the receptor on immune cells exert antiatherogenic and anti‐inflammatory effects. HCA_2_ is also a receptor for butyrate and mediates some of the beneficial effects of short‐chain fatty acids produced by gut microbiota. HCA_3_ has been shown to be activated by aromatic D‐amino acids.

### Further reading on Hydroxycarboxylic acid receptors

Boatman PD *et al*. (2008) Nicotinic acid receptor agonists. *J. Med. Chem.* **51**: 7653‐62 https://www.ncbi.nlm.nih.gov/pubmed/18983141?dopt=AbstractPlus

Graff EC *et al*. (2016) Anti‐inflammatory effects of the hydroxycarboxylic acid receptor 2. *Metab. Clin. Exp.* **65**: 102‐13 https://www.ncbi.nlm.nih.gov/pubmed/26773933?dopt=AbstractPlus

Kamanna VS *et al*. (2013) Recent advances in niacin and lipid metabolism. *Curr. Opin. Lipidol.* **24**: 239‐45 [https://www.ncbi.nlm.nih.gov/pubmed/23619367?dopt=AbstractPlus]

Offermanns S. (2017) Hydroxy‐Carboxylic Acid Receptor Actions in Metabolism. *Trends Endocrinol.* *Metab.* **28**: 227‐236 https://www.ncbi.nlm.nih.gov/pubmed/28087125?dopt=AbstractPlus

Offermanns S *et al*. (2011) International Union of Basic and Clinical Pharmacology. LXXXII: Nomenclature and Classification of Hydroxy‐carboxylic Acid Receptors (GPR81, GPR109A, and GPR109B). *Pharmacol. Rev.* **63**: 269‐90 https://www.ncbi.nlm.nih.gov/pubmed/21454438?dopt=AbstractPlus

Offermanns S *et al*. (2015) Nutritional or pharmacological activation of HCA(2) ameliorates neuroinflammation. *Trends Mol Med* **21**: 245‐55 https://www.ncbi.nlm.nih.gov/pubmed/25766751?dopt=AbstractPlus

## http://www.guidetopharmacology.org/GRAC/FamilyDisplayForward?familyId=34

### Overview

The kisspeptin receptor (**nomenclature as agreed by the NC‐IUPHAR Subcommittee on the kisspeptin receptor** [http://www.ncbi.nlm.nih.gov/pubmed/21079036?dopt=AbstractPlus]), like neuropeptide FF (NPFF), prolactin‐releasing peptide (PrP) and QRFP receptors (provisional nomenclature) responds to endogenous peptides with an arginine‐phenylalanine amide (RFamide) motif. http://www.guidetopharmacology.org/GRAC/LigandDisplayForward?ligandId=1288 (https://www.genenames.org/data/gene‐symbol‐report/#!/hgnc_id/HGNC:6341, http://www.uniprot.org/uniprot/Q15726) (KP54, originally named metastin), http://www.guidetopharmacology.org/GRAC/LigandDisplayForward?ligandId=1284 (https://www.genenames.org/data/gene‐symbol‐report/#!/hgnc_id/HGNC:6341, http://www.uniprot.org/uniprot/Q15726) (KP13) and http://www.guidetopharmacology.org/GRAC/LigandDisplayForward?ligandId=1283 (https://www.genenames.org/data/gene‐symbol‐report/#!/hgnc_id/HGNC:6341) (KP10) are biologically‐active peptides cleaved from the https://www.genenames.org/data/gene‐symbol‐report/#!/hgnc_id/HGNC:6341 (http://www.uniprot.org/uniprot/Q15726) gene product. Kisspeptins have roles in, for example, cancer metastasis, fertility/puberty regula tion and glucose homeostasis.

**Table:** 

Nomenclature	http://www.guidetopharmacology.org/GRAC/ObjectDisplayForward?objectId=266
HGNC, UniProt	https://www.genenames.org/data/gene‐symbol‐report/#!/hgnc_id/HGNC:4510, http://www.uniprot.org/uniprot/Q969F8
Endogenous agonists	http://www.guidetopharmacology.org/GRAC/LigandDisplayForward?ligandId=1283 (https://www.genenames.org/data/gene‐symbol‐report/#!/hgnc_id/HGNC:6341) [http://www.ncbi.nlm.nih.gov/pubmed/11457843?dopt=AbstractPlus, http://www.ncbi.nlm.nih.gov/pubmed/11385580?dopt=AbstractPlus], http://www.guidetopharmacology.org/GRAC/LigandDisplayForward?ligandId=1288 (https://www.genenames.org/data/gene‐symbol‐report/#!/hgnc_id/HGNC:6341, http://www.uniprot.org/uniprot/Q15726) [http://www.ncbi.nlm.nih.gov/pubmed/11457843?dopt=AbstractPlus, http://www.ncbi.nlm.nih.gov/pubmed/11385580?dopt=AbstractPlus], http://www.guidetopharmacology.org/GRAC/LigandDisplayForward?ligandId=1285 (https://www.genenames.org/data/gene‐symbol‐report/#!/hgnc_id/HGNC:6341, http://www.uniprot.org/uniprot/Q15726) [http://www.ncbi.nlm.nih.gov/pubmed/11457843?dopt=AbstractPlus], http://www.guidetopharmacology.org/GRAC/LigandDisplayForward?ligandId=1284 (https://www.genenames.org/data/gene‐symbol‐report/#!/hgnc_id/HGNC:6341, http://www.uniprot.org/uniprot/Q15726) [http://www.ncbi.nlm.nih.gov/pubmed/11457843?dopt=AbstractPlus]
Selective agonists	http://www.guidetopharmacology.org/GRAC/LigandDisplayForward?ligandId=4014 [http://www.ncbi.nlm.nih.gov/pubmed/18302161?dopt=AbstractPlus], http://www.guidetopharmacology.org/GRAC/LigandDisplayForward?ligandId=4015 [http://www.ncbi.nlm.nih.gov/pubmed/19934405?dopt=AbstractPlus] – Mouse, http://www.guidetopharmacology.org/GRAC/LigandDisplayForward?ligandId=10222 [http://www.ncbi.nlm.nih.gov/pubmed/27589480?dopt=AbstractPlus]
Selective antagonists	http://www.guidetopharmacology.org/GRAC/LigandDisplayForward?ligandId=4012 [http://www.ncbi.nlm.nih.gov/pubmed/19321788?dopt=AbstractPlus]
Labelled ligands	http://www.guidetopharmacology.org/GRAC/LigandDisplayForward?ligandId=1280 (Agonist) [http://www.ncbi.nlm.nih.gov/pubmed/11385580?dopt=AbstractPlus], http://www.guidetopharmacology.org/GRAC/LigandDisplayForward?ligandId=3792) (Agonist) [http://www.ncbi.nlm.nih.gov/pubmed/17023533?dopt=AbstractPlus], http://www.guidetopharmacology.org/GRAC/LigandDisplayForward?ligandId=1281) (Agonist) [http://www.ncbi.nlm.nih.gov/pubmed/11457843?dopt=AbstractPlus], http://www.guidetopharmacology.org/GRAC/LigandDisplayForward?ligandId=4013) (Agonist) [http://www.ncbi.nlm.nih.gov/pubmed/17023533?dopt=AbstractPlus], http://www.guidetopharmacology.org/GRAC/LigandDisplayForward?ligandId=10223 (Agonist) [http://www.ncbi.nlm.nih.gov/pubmed/30044673?dopt=AbstractPlus]

### Comments

2‐acylamino‐4,6‐diphenylpyridine derivatives have been described and are the first small molecule kisspeptin receptor antagonists reported with potential for treatment of sex‐hormone dependent diseases such as prostate cancer and endometriosis [http://www.ncbi.nlm.nih.gov/pubmed/20580563?dopt=AbstractPlus] .

### Further reading on Kisspeptin receptor

Harter CJL *et al*. (2018) The role of kisspeptin neurons in reproduction and metabolism. *J. Endocrinol.* **238**: R173‐R183 https://www.ncbi.nlm.nih.gov/pubmed/30042117?dopt=AbstractPlus

Kanda S *et al*. (2013) Structure, synthesis, and phylogeny of kisspeptin and its receptor. *Adv. Exp. Med. Biol.* **784**: 9‐26 https://www.ncbi.nlm.nih.gov/pubmed/23550000?dopt=AbstractPlus

Kirby HR *et al*. (2010) International Union of Basic and Clinical Pharmacology. LXXVII. Kisspeptin receptor nomenclature, distribution, and function. *Pharmacol. Rev.* **62**: 565‐78 https://www.ncbi.nlm.nih.gov/pubmed/21079036?dopt=AbstractPlus

Oakley AE *et al*. (2009) Kisspeptin signaling in the brain. *Endocr. Rev.* **30**: 713‐43 https://www.ncbi.nlm.nih.gov/pubmed/19770291?dopt=AbstractPlus

Pasquier J *et al*. (2014) Molecular evolution of GPCRs: Kisspeptin/kisspeptin receptors. *J. Mol. Endocrinol.* **52**: T101‐17 https://www.ncbi.nlm.nih.gov/pubmed/24577719?dopt=AbstractPlus

## http://www.guidetopharmacology.org/GRAC/FamilyDisplayForward?familyId=35

### Overview

The leukotriene receptors (**nomenclature as agreed by the NC‐IUPHAR subcommittee on Leukotriene Receptors** [http://www.ncbi.nlm.nih.gov/pubmed/21771892?dopt=AbstractPlus, http://www.ncbi.nlm.nih.gov/pubmed/24588652?dopt=AbstractPlus]) are activated by the endogenous ligands leukotrienes (LT), synthesized from lipoxygenase metabolism of arachidonic acid. The human BLT_1_ receptor is the high affinity LTB_4_ receptor whereas the BLT_2_ receptor in addition to being a low‐affinity LTB_4_ receptor also binds several other lipoxygenase‐products, such as http://www.guidetopharmacology.org/GRAC/LigandDisplayForward?ligandId=3404, http://www.guidetopharmacology.org/GRAC/LigandDisplayForward?ligandId=2481, http://www.guidetopharmacology.org/GRAC/LigandDisplayForward?ligandId=3401, and the thromboxane synthase product http://www.guidetopharmacology.org/GRAC/LigandDisplayForward?ligandId=6159. The BLT receptors mediate chemotaxis and immunomodulation in several leukocyte populations and are in addition expressed on non‐myeloid cells, such as vascular smooth muscle and endothelial cells. In addition to BLT receptors, LTB_4_ has been reported to bind to the peroxisome proliferator activated receptor (PPAR) α [http://www.ncbi.nlm.nih.gov/pubmed/9890897?dopt=AbstractPlus] and the vanilloid TRPV1 ligand‐gated nonselective cation channel [http://www.ncbi.nlm.nih.gov/pubmed/16207832?dopt=AbstractPlus]. The receptors for the cysteinyl‐leukotrienes (*i.e.* http://www.guidetopharmacology.org/GRAC/LigandDisplayForward?ligandId=3354, http://www.guidetopharmacology.org/GRAC/LigandDisplayForward?ligandId=3353 and http://www.guidetopharmacology.org/GRAC/LigandDisplayForward?ligandId=3352) aretermed CysLT_1_ and CysLT_2_ and exhibit distinct expression patterns in human tissues, mediating for example smooth muscle cell contraction, regulation of vascular permeability, and leukocyte activation. There is also evidence in the literature for additional CysLT receptor subtypes, derived from functional in vitro studies, radioligand binding and in mice lacking both CysLT_1_ and CysLT_2_ receptors [http://www.ncbi.nlm.nih.gov/pubmed/24588652?dopt=AbstractPlus]. Cysteinyl‐leukotrienes have also been suggested to signal through the P2Y_12_ receptor [http://www.ncbi.nlm.nih.gov/pubmed/20702811?dopt=AbstractPlus, http://www.ncbi.nlm.nih.gov/pubmed/16185654?dopt=AbstractPlus, http://www.ncbi.nlm.nih.gov/pubmed/19822647?dopt=AbstractPlus], GPR17 [http://www.ncbi.nlm.nih.gov/pubmed/16990797?dopt=AbstractPlus] and GPR99 [http://www.ncbi.nlm.nih.gov/pubmed/23504326?dopt=AbstractPlus].

**Table:** 

Nomenclature	http://www.guidetopharmacology.org/GRAC/ObjectDisplayForward?objectId=267	http://www.guidetopharmacology.org/GRAC/ObjectDisplayForward?objectId=268	http://www.guidetopharmacology.org/GRAC/ObjectDisplayForward?objectId=269	http://www.guidetopharmacology.org/GRAC/ObjectDisplayForward?objectId=270	http://www.guidetopharmacology.org/GRAC/ObjectDisplayForward?objectId=271	http://www.guidetopharmacology.org/GRAC/ObjectDisplayForward?objectId=223
HGNC, UniProt	https://www.genenames.org/data/gene‐symbol‐report/#!/hgnc_id/HGNC:6713, http://www.uniprot.org/uniprot/Q15722	https://www.genenames.org/data/gene‐symbol‐report/#!/hgnc_id/HGNC:19260, http://www.uniprot.org/uniprot/Q9NPC1	https://www.genenames.org/data/gene‐symbol‐report/#!/hgnc_id/HGNC:17451, http://www.uniprot.org/uniprot/Q9Y271	https://www.genenames.org/data/gene‐symbol‐report/#!/hgnc_id/HGNC:18274, http://www.uniprot.org/uniprot/Q9NS75	https://www.genenames.org/data/gene‐symbol‐report/#!/hgnc_id/HGNC:24884, http://www.uniprot.org/uniprot/Q8TDS5	https://www.genenames.org/data/gene‐symbol‐report/#!/hgnc_id/HGNC:3827, http://www.uniprot.org/uniprot/P25090
Potency order of endogenous ligands	http://www.guidetopharmacology.org/GRAC/LigandDisplayForward?ligandId=2487 >http://www.guidetopharmacology.org/GRAC/LigandDisplayForward?ligandId=3399 ≫ http://www.guidetopharmacology.org/GRAC/LigandDisplayForward?ligandId=3405 [http://www.ncbi.nlm.nih.gov/pubmed/11278893?dopt=AbstractPlus]	http://www.guidetopharmacology.org/GRAC/LigandDisplayForward?ligandId=6159 > http://www.guidetopharmacology.org/GRAC/LigandDisplayForward?ligandId=2487 > http://www.guidetopharmacology.org/GRAC/LigandDisplayForward?ligandId=3404 = http://www.guidetopharmacology.org/GRAC/LigandDisplayForward?ligandId=2481 > http://www.guidetopharmacology.org/GRAC/LigandDisplayForward?ligandId=3401 > http://www.guidetopharmacology.org/GRAC/LigandDisplayForward?ligandId=3405 > http://www.guidetopharmacology.org/GRAC/LigandDisplayForward?ligandId=3399 [http://www.ncbi.nlm.nih.gov/pubmed/18378794?dopt=AbstractPlus, http://www.ncbi.nlm.nih.gov/pubmed/11278893?dopt=AbstractPlus]	http://www.guidetopharmacology.org/GRAC/LigandDisplayForward?ligandId=3353 > http://www.guidetopharmacology.org/GRAC/LigandDisplayForward?ligandId=3354 > http://www.guidetopharmacology.org/GRAC/LigandDisplayForward?ligandId=3352 [http://www.ncbi.nlm.nih.gov/pubmed/10391245?dopt=AbstractPlus, http://www.ncbi.nlm.nih.gov/pubmed/10462554?dopt=AbstractPlus]	http://www.guidetopharmacology.org/GRAC/LigandDisplayForward?ligandId=3354 ≥ http://www.guidetopharmacology.org/GRAC/LigandDisplayForward?ligandId=3353 ≫ http://www.guidetopharmacology.org/GRAC/LigandDisplayForward?ligandId=3352 [http://www.ncbi.nlm.nih.gov/pubmed/10851239?dopt=AbstractPlus, http://www.ncbi.nlm.nih.gov/pubmed/11093801?dopt=AbstractPlus, http://www.ncbi.nlm.nih.gov/pubmed/10913337?dopt=AbstractPlus]	http://www.guidetopharmacology.org/GRAC/LigandDisplayForward?ligandId=3391, http://www.guidetopharmacology.org/GRAC/LigandDisplayForward?ligandId=3392, http://www.guidetopharmacology.org/GRAC/LigandDisplayForward?ligandId=6164 > http://www.guidetopharmacology.org/GRAC/LigandDisplayForward?ligandId=6167 > http://www.guidetopharmacology.org/GRAC/LigandDisplayForward?ligandId=2483 > http://www.guidetopharmacology.org/GRAC/LigandDisplayForward?ligandId=3390 [http://www.ncbi.nlm.nih.gov/pubmed/19450703?dopt=AbstractPlus, http://www.ncbi.nlm.nih.gov/pubmed/12065583?dopt=AbstractPlus, http://www.ncbi.nlm.nih.gov/pubmed/12606753?dopt=AbstractPlus, http://www.ncbi.nlm.nih.gov/pubmed/9829988?dopt=AbstractPlus, http://www.ncbi.nlm.nih.gov/pubmed/18292294?dopt=AbstractPlus, http://www.ncbi.nlm.nih.gov/pubmed/1326548?dopt=AbstractPlus, http://www.ncbi.nlm.nih.gov/pubmed/7797484?dopt=AbstractPlus]	http://www.guidetopharmacology.org/GRAC/LigandDisplayForward?ligandId=1034 = http://www.guidetopharmacology.org/GRAC/LigandDisplayForward?ligandId=3933 = http://www.guidetopharmacology.org/GRAC/LigandDisplayForward?ligandId=3383 = http://www.guidetopharmacology.org/GRAC/LigandDisplayForward?ligandId=3934 > http://www.guidetopharmacology.org/GRAC/LigandDisplayForward?ligandId=3354 = http://www.guidetopharmacology.org/GRAC/LigandDisplayForward?ligandId=3353 ≫ http://www.guidetopharmacology.org/GRAC/LigandDisplayForward?ligandId=3402 ≫ http://www.guidetopharmacology.org/GRAC/LigandDisplayForward?ligandId=1022 [http://www.ncbi.nlm.nih.gov/pubmed/10393980?dopt=AbstractPlus, http://www.ncbi.nlm.nih.gov/pubmed/8006586?dopt=AbstractPlus, http://www.ncbi.nlm.nih.gov/pubmed/8527441?dopt=AbstractPlus, http://www.ncbi.nlm.nih.gov/pubmed/11141472?dopt=AbstractPlus, http://www.ncbi.nlm.nih.gov/pubmed/9151906?dopt=AbstractPlus]
Endogenous agonists	–	–	–	–	–	http://www.guidetopharmacology.org/GRAC/LigandDisplayForward?ligandId=1034 [http://www.ncbi.nlm.nih.gov/pubmed/20080636?dopt=AbstractPlus], http://www.guidetopharmacology.org/GRAC/LigandDisplayForward?ligandId=3934 [https://www.ncbi.nlm.nih.gov/pubmed/20080636?dopt=AbstractPlus], http://www.guidetopharmacology.org/GRAC/LigandDisplayForward?ligandId=6239 [http://www.ncbi.nlm.nih.gov/pubmed/22449948?dopt=AbstractPlus], http://www.guidetopharmacology.org/GRAC/LigandDisplayForward?ligandId=3933
Selective agonists	–	–	–	–	–	http://www.guidetopharmacology.org/GRAC/LigandDisplayForward?ligandId=3383 [http://www.ncbi.nlm.nih.gov/pubmed/15056011?dopt=AbstractPlus]
Endogenous antagonists	–	–	–	–	http://www.guidetopharmacology.org/GRAC/LigandDisplayForward?ligandId=6171 (pIC_50_ 6.3) [http://www.ncbi.nlm.nih.gov/pubmed/9920859?dopt=AbstractPlus]	–
Antagonists	–	–	http://www.guidetopharmacology.org/GRAC/LigandDisplayForward?ligandId=3634 (p*K*_i_ 7.1–8.8) [http://www.ncbi.nlm.nih.gov/pubmed/9504401?dopt=AbstractPlus, http://www.ncbi.nlm.nih.gov/pubmed/11996896?dopt=AbstractPlus], http://www.guidetopharmacology.org/GRAC/LigandDisplayForward?ligandId=3334 (p*K*i 7.1) [http://www.ncbi.nlm.nih.gov/pubmed/9547367?dopt=AbstractPlus]	http://www.guidetopharmacology.org/GRAC/LigandDisplayForward?ligandId=3634 (p*A*_2_ 7.1) [http://www.ncbi.nlm.nih.gov/pubmed/25839425?dopt=AbstractPlus], http://www.guidetopharmacology.org/GRAC/LigandDisplayForward?ligandId=3334 (p*A*_2_ 6.2) [http://www.ncbi.nlm.nih.gov/pubmed/25839425?dopt=AbstractPlus]	–	–
Selective antagonists	http://www.guidetopharmacology.org/GRAC/LigandDisplayForward?ligandId=6154 (p*K*_i_ 8.8) [http://www.ncbi.nlm.nih.gov/pubmed/11259574?dopt=AbstractPlus, http://www.ncbi.nlm.nih.gov/pubmed/17170051?dopt=AbstractPlus], http://www.guidetopharmacology.org/GRAC/LigandDisplayForward?ligandId=3368 (pIC_50_ 8.1) [http://www.ncbi.nlm.nih.gov/pubmed/7714764?dopt=AbstractPlus], http://www.guidetopharmacology.org/GRAC/LigandDisplayForward?ligandId=3325 (p*K*_i_ 6.4) [http://www.ncbi.nlm.nih.gov/pubmed/10513580?dopt=AbstractPlus]	http://www.guidetopharmacology.org/GRAC/LigandDisplayForward?ligandId=3351 (pIC_50_ 6–7.1) [http://www.ncbi.nlm.nih.gov/pubmed/1316967?dopt=AbstractPlus, http://www.ncbi.nlm.nih.gov/pubmed/11278893?dopt=AbstractPlus]	http://www.guidetopharmacology.org/GRAC/LigandDisplayForward?ligandId=3358 (p*K*_i_ 9.7) [http://www.ncbi.nlm.nih.gov/pubmed/2170649?dopt=AbstractPlus] – Guinea pig, http://www.guidetopharmacology.org/GRAC/LigandDisplayForward?ligandId=3322 (zafirlukast is only about 100‐fold selective for CysLT_1_) (p*K*_i_ 8.9) [http://www.ncbi.nlm.nih.gov/pubmed/9504401?dopt=AbstractPlus, http://www.ncbi.nlm.nih.gov/pubmed/11996896?dopt=AbstractPlus], http://www.guidetopharmacology.org/GRAC/LigandDisplayForward?ligandId=3340 (p*K*i 8.6) [http://www.ncbi.nlm.nih.gov/pubmed/11996896?dopt=AbstractPlus], http://www.guidetopharmacology.org/GRAC/LigandDisplayForward?ligandId=10346 (pIC_50_ 8) [http://www.ncbi.nlm.nih.gov/pubmed/10391245?dopt=AbstractPlus]	http://www.guidetopharmacology.org/GRAC/LigandDisplayForward?ligandId=6196 (p*A*_2_ 8.4) [http://www.ncbi.nlm.nih.gov/pubmed/21753081?dopt=AbstractPlus], http://www.guidetopharmacology.org/GRAC/LigandDisplayForward?ligandId=6196 (p*A*_2_ 8.3) [http://www.ncbi.nlm.nih.gov/pubmed/21753081?dopt=AbstractPlus], http://www.guidetopharmacology.org/GRAC/LigandDisplayForward?ligandId=6197 (pIC_50_ 7.4) [http://www.ncbi.nlm.nih.gov/pubmed/20423349?dopt=AbstractPlus]	–	http://www.guidetopharmacology.org/GRAC/LigandDisplayForward?ligandId=1040 (pIC_50_ 6.6) [http://www.ncbi.nlm.nih.gov/pubmed/15210823?dopt=AbstractPlus], http://www.guidetopharmacology.org/GRAC/LigandDisplayForward?ligandId=1030 (pIC_50_ 4.3–6) [http://www.ncbi.nlm.nih.gov/pubmed/6280748?dopt=AbstractPlus, http://www.ncbi.nlm.nih.gov/pubmed/17687636?dopt=AbstractPlus, http://www.ncbi.nlm.nih.gov/pubmed/10882119?dopt=AbstractPlus]
Labelled ligands	http://www.guidetopharmacology.org/GRAC/LigandDisplayForward?ligandId=3413 (Agonist) [http://www.ncbi.nlm.nih.gov/pubmed/9177352?dopt=AbstractPlus], http://www.guidetopharmacology.org/GRAC/LigandDisplayForward?ligandId=3415 (Antagonist) (p*K*_d_ 7.9) [http://www.ncbi.nlm.nih.gov/pubmed/1320692?dopt=AbstractPlus]	http://www.guidetopharmacology.org/GRAC/LigandDisplayForward?ligandId=3413 (p^*K*^_d_ 7.6–9.7)	http://www.guidetopharmacology.org/GRAC/LigandDisplayForward?ligandId=3412 (Agonist), http://www.guidetopharmacology.org/GRAC/LigandDisplayForward?ligandId=3414 (Antagonist) (p*K*_d_ 10.6) [http://www.ncbi.nlm.nih.gov/pubmed/1329767?dopt=AbstractPlus]	http://www.guidetopharmacology.org/GRAC/LigandDisplayForward?ligandId=3412 (Agonist) [http://www.ncbi.nlm.nih.gov/pubmed/10851239?dopt=AbstractPlus]	http://www.guidetopharmacology.org/GRAC/LigandDisplayForward?ligandId=3416 (Agonist) [http://www.ncbi.nlm.nih.gov/pubmed/9829988?dopt=AbstractPlus]	http://www.guidetopharmacology.org/GRAC/LigandDisplayForward?ligandId=3411 (Agonist) [http://www.ncbi.nlm.nih.gov/pubmed/8006586?dopt=AbstractPlus, http://www.ncbi.nlm.nih.gov/pubmed/1322894?dopt=AbstractPlus]

### Comments

The FPR2/ALX receptor (**nomenclatureas agreed by the NC‐IUPHAR subcommittee on Leukotriene and Lipoxin Receptors** [http://www.ncbi.nlm.nih.gov/pubmed/24588652?dopt=AbstractPlus]) is activated by the endogenous lipidderived, anti‐inflammatory ligands lipoxin A_4_ (http://www.guidetopharmacology.org/GRAC/LigandDisplayForward?ligandId=1034) and 15‐epiLXA_4_ (http://www.guidetopharmacology.org/GRAC/LigandDisplayForward?ligandId=3933, ATL). The FPR2/ALX receptor also interacts with endogenous peptide and protein ligands, such as MHC binding peptide [http://www.ncbi.nlm.nih.gov/pubmed/10748237?dopt=AbstractPlus] as well as http://www.guidetopharmacology.org/GRAC/LigandDisplayForward?ligandId=1031 (https://www.genenames.org/data/gene‐symbol‐report/#!/hgnc_id/HGNC:533, http://www.uniprot.org/uniprot/P04083) (ANXA1) and its *N*‐terminal peptides [http://www.ncbi.nlm.nih.gov/pubmed/24108355?dopt=AbstractPlus, http://www.ncbi.nlm.nih.gov/pubmed/12368905?dopt=AbstractPlus]. In addition, a soluble hydrolytic product of protease action on the urokinase‐type plasminogen activator receptor has been reported to activate the FPR2/ALX receptor [http://www.ncbi.nlm.nih.gov/pubmed/11818541?dopt=AbstractPlus]. Furthermore, FPR2/ALX has been suggested to act as a receptor mediating the proinflammatory actions of the acute‐phase reactant, serum amyloid A [http://www.ncbi.nlm.nih.gov/pubmed/15171815?dopt=AbstractPlus, http://www.ncbi.nlm.nih.gov/pubmed/9892621?dopt=AbstractPlus]. The agonist activity of the lipid mediators described has been questioned [http://www.ncbi.nlm.nih.gov/pubmed/23643932?dopt=AbstractPlus, http://www.ncbi.nlm.nih.gov/pubmed/23607720?dopt=AbstractPlus], which may derive from batchto‐batch differences, partial agonism or biased agonism. Results from Cooray *et al.* (2013) [http://www.ncbi.nlm.nih.gov/pubmed/24108355?dopt=AbstractPlus] have addressed this issue and the role of homodimers and heterodimers in intracellular signaling. A receptor selective for http://www.guidetopharmacology.org/GRAC/LigandDisplayForward?ligandId=5216 has been suggested from functional studies [http://www.ncbi.nlm.nih.gov/pubmed/12794159?dopt=AbstractPlus, http://www.ncbi.nlm.nih.gov/pubmed/8551217?dopt=AbstractPlus, http://www.ncbi.nlm.nih.gov/pubmed/8757340?dopt=AbstractPlus]. Note that the data for FPR2/ALX are also reproduced on the http://www.guidetopharmacology.org/GRAC/FamilyDisplayForward?familyId=23.

Oxoeicosanoid receptors (OXE, **nomenclature agreed by the** **NC‐IUPHAR subcommittee on Oxoeicosanoid Receptors** [http://www.ncbi.nlm.nih.gov/pubmed/15001665?dopt=AbstractPlus]) are activated by endogenous chemotactic eicosanoid ligands oxidised at the C‐5 position, with http://www.guidetopharmacology.org/GRAC/LigandDisplayForward?ligandId=3391 the most potent agonist identified for this receptor. Initial characterization of the heterologously expressed OXE receptor suggested that polyunsaturated fatty acids, such as http://www.guidetopharmacology.org/GRAC/LigandDisplayForward?ligandId=1051 and http://www.guidetopharmacology.org/GRAC/LigandDisplayForward?ligandId=3362, acted as receptor antagonists [http://www.ncbi.nlm.nih.gov/pubmed/12065583?dopt=AbstractPlus].

### Further reading on Leukotriene receptors

Brink C *et al*. (2004) International Union of Pharmacology XLIV. Nomenclature for the Oxoeicosanoid Receptor. *Pharmacol. Rev.* **56**: 149‐157 https://www.ncbi.nlm.nih.gov/pubmed/15001665?dopt=AbstractPlus

Brink C *et al*. (2003) International Union of Pharmacology XXXVII. Nomenclature for leukotriene and lipoxin receptors. *Pharmacol. Rev.* **55**: 195‐227 https://www.ncbi.nlm.nih.gov/pubmed/12615958?dopt=AbstractPlus

Bäck M *et al*. (2011) International Union of Basic and Clinical Pharmacology. LXXXIV: leukotriene receptor nomenclature, distribution, and pathophysiological functions. *Pharmacol. Rev.* **63**: 539‐84 https://www.ncbi.nlm.nih.gov/pubmed/21771892?dopt=AbstractPlus

Bäck M *et al*. (2014) Update on leukotriene, lipoxin and oxoeicosanoid receptors: IUPHAR Review 7. *Br. J. Pharmacol.* **171**: 3551‐74 https://www.ncbi.nlm.nih.gov/pubmed/24588652?dopt=AbstractPlus

Laidlaw TM *et al*. (2012) Cysteinyl leukotriene receptors, old and new; implications for asthma. *Clin. Exp. Allergy* **42**: 1313‐20 https://www.ncbi.nlm.nih.gov/pubmed/22925317?dopt=AbstractPlus

## http://www.guidetopharmacology.org/GRAC/FamilyDisplayForward?familyId=36

### Overview

Lysophosphatidic acid (LPA) receptors (**nomenclature as agreed by the NC‐IUPHAR Subcommittee on Lysophospholipid (LPA) receptors Lysophospholipid Receptors** [http://www.ncbi.nlm.nih.gov/pubmed/23686350?dopt=AbstractPlus, http://www.ncbi.nlm.nih.gov/pubmed/24602016?dopt=AbstractPlus]) are activated by the endogenous phospholipid http://www.guidetopharmacology.org/GRAC/LigandDisplayForward?ligandId=2906. The first receptor, LPA_1_, was identified as *ventricular zone gene‐1* (*vzg‐1*) [http://www.ncbi.nlm.nih.gov/pubmed/8922387?dopt=AbstractPlus], leading to deorphanisation of members of the endothelial differentiation gene (*edg*) family as other LPA receptors along with sphingosine 1phosphate (S1P) receptors. Additional LPA receptor GPCRs were later identified. Gene names have been codified as *LPAR1*, *etc*. to reflect the receptor function of proteins. The crystal structure of LPA_1_ was solved and demonstrates extracellular LPA access to the binding pocket, consistent with proposed delivery *via* autotaxin [http://www.ncbi.nlm.nih.gov/pubmed/26091040?dopt=AbstractPlus]. These studies have also implicated cross‐talk with endocannabinoids *via* phosphorylated intermediates that can also activate these receptors. The identified receptors can account for most, although not all, LPA‐induced phenomena in the literature, indicating that a majority of LPA‐dependent phenomena are receptor‐mediated. Binding affinities of unlabeled, natural LPA and AEApto LPA_1_ weremeasured using backscattering interferometry (pK_d_ = 9) [http://www.ncbi.nlm.nih.gov/pubmed/30463988?dopt=AbstractPlus]. Binding affinities were 77‐fold lower than than values obtained using radioactivity [http://www.ncbi.nlm.nih.gov/pubmed/19386608?dopt=AbstractPlus]. Targeted deletion of LPA receptors has clarified signalling pathways and identified physiological and pathophysiological roles. Independent validation by multiple groups has been reported in the peer‐reviewed literature for all six LPA receptors described in the tables, including further validation using a distinct read‐out *via* a novel TGFα “shedding” assay [http://www.ncbi.nlm.nih.gov/pubmed/22983457?dopt=AbstractPlus]. http://www.guidetopharmacology.org/GRAC/LigandDisplayForward?ligandId=2906 has also been described as an agonist for the transient receptor potential (Trp) ion channel TRPV1 [http://www.ncbi.nlm.nih.gov/pubmed/22101604?dopt=AbstractPlus] and TRPA1 [http://www.ncbi.nlm.nih.gov/pubmed/28176353?dopt=AbstractPlus]. LPA was originally proposed to be a ligand for GPCR35, but data show that in fact it is a receptor for http://www.guidetopharmacology.org/GRAC/LigandDisplayForward?ligandId=6479 (https://www.genenames.org/data/gene‐symbol‐report/#!/hgnc_id/HGNC:19232, http://www.uniprot.org/uniprot/Q6UXB2) [http://www.ncbi.nlm.nih.gov/pubmed/25411203?dopt=AbstractPlus]. All of these proposed non‐GPCR receptor identities require confirmation and are not currently recognized as *bona fide* LPA receptors.

**Table:** 

Nomenclature	http://www.guidetopharmacology.org/GRAC/ObjectDisplayForward?objectId=272	http://www.guidetopharmacology.org/GRAC/ObjectDisplayForward?objectId=273	http://www.guidetopharmacology.org/GRAC/ObjectDisplayForward?objectId=274	http://www.guidetopharmacology.org/GRAC/ObjectDisplayForward?objectId=94	http://www.guidetopharmacology.org/GRAC/ObjectDisplayForward?objectId=124	http://www.guidetopharmacology.org/GRAC/ObjectDisplayForward?objectId=163
HGNC, UniProt	https://www.genenames.org/data/gene‐symbol‐report/#!/hgnc_id/HGNC:3166, http://www.uniprot.org/uniprot/Q92633	https://www.genenames.org/data/gene‐symbol‐report/#!/hgnc_id/HGNC:3168, http://www.uniprot.org/uniprot/Q9HBW0	https://www.genenames.org/data/gene‐symbol‐report/#!/hgnc_id/HGNC:14298, http://www.uniprot.org/uniprot/Q9UBY5	https://www.genenames.org/data/gene‐symbol‐report/#!/hgnc_id/HGNC:4478, http://www.uniprot.org/uniprot/Q99677	https://www.genenames.org/data/gene‐symbol‐report/#!/hgnc_id/HGNC:13307, http://www.uniprot.org/uniprot/Q9H1C0	https://www.genenames.org/data/gene‐symbol‐report/#!/hgnc_id/HGNC:15520, http://www.uniprot.org/uniprot/P43657
Selective agonists	–	http://www.guidetopharmacology.org/GRAC/LigandDisplayForward?ligandId=4031 [http://www.ncbi.nlm.nih.gov/pubmed/12695531?dopt=AbstractPlus], http://www.guidetopharmacology.org/GRAC/LigandDisplayForward?ligandId=4030 [https://www.ncbi.nlm.nih.gov/pubmed/12695531?dopt=AbstractPlus], http://www.guidetopharmacology.org/GRAC/LigandDisplayForward?ligandId=6580 [http://www.ncbi.nlm.nih.gov/pubmed/22968304?dopt=AbstractPlus]	http://www.guidetopharmacology.org/GRAC/LigandDisplayForward?ligandId=2912 [http://www.ncbi.nlm.nih.gov/pubmed/12554733?dopt=AbstractPlus]	–	–	–
Antagonists	http://www.guidetopharmacology.org/GRAC/LigandDisplayForward?ligandId=2907 (pIC_50_ 6.6–6.9) [http://www.ncbi.nlm.nih.gov/pubmed/14500756?dopt=AbstractPlus] – Mouse, http://www.guidetopharmacology.org/GRAC/LigandDisplayForward?ligandId=2909 (p*K*_i_ 5.2–6.9) [http://www.ncbi.nlm.nih.gov/pubmed/11723223?dopt=AbstractPlus] – Mouse, http://www.guidetopharmacology.org/GRAC/LigandDisplayForward?ligandId=5450 [http://www.ncbi.nlm.nih.gov/pubmed/15125924?dopt=AbstractPlus]	–	http://www.guidetopharmacology.org/GRAC/LigandDisplayForward?ligandId=2909 (p*K*_i_ 6.4) [http://www.ncbi.nlm.nih.gov/pubmed/11723223?dopt=AbstractPlus], http://www.guidetopharmacology.org/GRAC/LigandDisplayForward?ligandId=5450 [http://www.ncbi.nlm.nih.gov/pubmed/15125924?dopt=AbstractPlus]	–	–	–
Sub/family‐selective antagonists	–	–	http://www.guidetopharmacology.org/GRAC/LigandDisplayForward?ligandId=2907 (p*K*_i_ 6.4) [http://www.ncbi.nlm.nih.gov/pubmed/14500756?dopt=AbstractPlus]	–	–	–
Selective antagonists	http://www.guidetopharmacology.org/GRAC/LigandDisplayForward?ligandId=9498 (pIC_50_ 8.9), http://www.guidetopharmacology.org/GRAC/LigandDisplayForward?ligandId=2905 (pIC_50_ 6.7–7.8) [http://www.ncbi.nlm.nih.gov/pubmed/21159750?dopt=AbstractPlus], http://www.guidetopharmacology.org/GRAC/LigandDisplayForward?ligandId=9499 (pIC_50_ 6.8) [http://www.ncbi.nlm.nih.gov/pubmed/27774128?dopt=AbstractPlus], http://www.guidetopharmacology.org/GRAC/LigandDisplayForward?ligandId=6988 (pIC_50_ 6–6.1) [http://www.ncbi.nlm.nih.gov/pubmed/21159750?dopt=AbstractPlus]	–	http://www.guidetopharmacology.org/GRAC/LigandDisplayForward?ligandId=2916 (p*K*_i_ 5.5–7) [http://www.ncbi.nlm.nih.gov/pubmed/11562440?dopt=AbstractPlus, http://www.ncbi.nlm.nih.gov/pubmed/14500756?dopt=AbstractPlus]	–	http://www.guidetopharmacology.org/GRAC/LigandDisplayForward?ligandId=10230 (pIC_50_ 7.4) [http://www.ncbi.nlm.nih.gov/pubmed/28859883?dopt=AbstractPlus], http://www.guidetopharmacology.org/GRAC/LigandDisplayForward?ligandId=9500 (pIC_50_ 6.1) [http://www.ncbi.nlm.nih.gov/pubmed/22801643?dopt=AbstractPlus]	–

### Comments

http://www.guidetopharmacology.org/GRAC/LigandDisplayForward?ligandId=2907 [http://www.ncbi.nlm.nih.gov/pubmed/14500756?dopt=AbstractPlus], http://www.guidetopharmacology.org/GRAC/LigandDisplayForward?ligandId=2909 [http://www.ncbi.nlm.nih.gov/pubmed/11723223?dopt=AbstractPlus] and http://www.guidetopharmacology.org/GRAC/LigandDisplayForward?ligandId=5450 [http://www.ncbi.nlm.nih.gov/pubmed/15125924?dopt=AbstractPlus] have dual antagonist activity at LPA_1_ and LPA_3_ receptors. There is growing evidence for *in vivo* efficacy of these chemical antagonists in several disorders, including fetal hydrocephalus [http://www.ncbi.nlm.nih.gov/pubmed/21900594?dopt=AbstractPlus], fetal hypoxia [http://www.ncbi.nlm.nih.gov/pubmed/21878565?dopt=AbstractPlus], lung fibrosis [http://www.ncbi.nlm.nih.gov/pubmed/25959255?dopt=AbstractPlus], systemic sclerosis [http://www.ncbi.nlm.nih.gov/pubmed/25959255?dopt=AbstractPlus] and atherosclerosis progression [http://www.ncbi.nlm.nih.gov/pubmed/27883026?dopt=AbstractPlus]. The LPA_2_ selective agonist, http://www.guidetopharmacology.org/GRAC/LigandDisplayForward?ligandId=6580, also shows efficacy in an animal model of multiple sclerosis [http://www.ncbi.nlm.nih.gov/pubmed/28578681?dopt=AbstractPlus]. The LPA5 selective antagonist, http://www.guidetopharmacology.org/GRAC/LigandDisplayForward?ligandId=10230, is effective in pain models [http://www.ncbi.nlm.nih.gov/pubmed/29208511?dopt=AbstractPlus].

### Further reading on Lysophospholipid (LPA) receptors

Chun J *et al*. (2010) International Union of Basic and Clinical Pharmacology. LXXVIII. Lysophospholipid receptor nomenclature. *Pharmacol. Rev.* **62**: 579‐87 https://www.ncbi.nlm.nih.gov/pubmed/21079037?dopt=AbstractPlus

Kihara Y *et al*. (2014) Lysophospholipid receptor nomenclature review: IUPHAR Review 8. *Br. J. Pharmacol.* **171**: 3575‐94 https://www.ncbi.nlm.nih.gov/pubmed/24602016?dopt=AbstractPlus

Yung YC *et al*. (2015) Lysophosphatidic Acid signaling in the nervous system. *Neuron* **85**: 669‐82 https://www.ncbi.nlm.nih.gov/pubmed/25695267?dopt=AbstractPlus

## http://www.guidetopharmacology.org/GRAC/FamilyDisplayForward?familyId=135

### Overview

Sphingosine 1‐phosphate (S1P) receptors (**nomenclature as agreed by the NC‐IUPHAR Subcommittee on Lysophospholipid receptors** [http://www.ncbi.nlm.nih.gov/pubmed/24602016?dopt=AbstractPlus]) are activated by the endogenous lipid http://www.guidetopharmacology.org/GRAC/LigandDisplayForward?ligandId=911 (S1P). Originally cloned as orphan members of the endothelial differentiation gene (*edg*) family, current gene names have been designated as S1P_1_R through S1P_5_R [http://www.ncbi.nlm.nih.gov/pubmed/2160972?dopt=AbstractPlus]. S1PRs, particularly S1P_1_, are expressed throughout all mammalian organ systems. Ligand delivery occurs *via* two known carriers (or “chaperones”): albumin and HDLbound apolipoprotein M (ApoM), the latter of which elicits biased agonist signaling by S1P_1_ in multiple cell types [http://www.ncbi.nlm.nih.gov/pubmed/26053123?dopt=AbstractPlus, http://www.ncbi.nlm.nih.gov/pubmed/26268607?dopt=AbstractPlus]. The five S1PRs, two chaperones, and active cellular metabolism have complicated analyses of receptor ligand binding in native systems. Signaling pathways and physiological roles have been characterized through radioligand binding in heterologous expression systems, targeted deletion of the different S1PRs, and most recently, mouse models that report *in vivo* S1P1R activation [http://www.ncbi.nlm.nih.gov/pubmed/29079828?dopt=AbstractPlus, http://www.ncbi.nlm.nih.gov/pubmed/24667638?dopt=AbstractPlus]. A crystal structure of an S1P_1_‐T4 fusion protein confirmed aspects and binding, specificity, and receptor activation determined previously through biochemical and genetic studies [http://www.ncbi.nlm.nih.gov/pubmed/30343728?dopt=AbstractPlus, http://www.ncbi.nlm.nih.gov/pubmed/22344443?dopt=AbstractPlus]. http://www.guidetopharmacology.org/GRAC/LigandDisplayForward?ligandId=2407 (FTY720), the first drug to target any of the lysophospholipid receptors, binds to four of the five S1PRs, and was the first oral therapy for multiple sclerosis [http://www.ncbi.nlm.nih.gov/pubmed/30625282?dopt=AbstractPlus]. The mechanisms of action of fingolimod and other S1PR modulating drugs in development include binding S1PRs in multiple organ systems, *e.g.*, immune and nervous systems, although the precise nature of their receptor interactions requires clarification [http://www.ncbi.nlm.nih.gov/pubmed/21520239?dopt=AbstractPlus, http://www.ncbi.nlm.nih.gov/pubmed/23518370?dopt=AbstractPlus, http://www.ncbi.nlm.nih.gov/pubmed/30255127?dopt=AbstractPlus, http://www.ncbi.nlm.nih.gov/pubmed/25831442?dopt=AbstractPlus].

**Table:** 

Nomenclature	http://www.guidetopharmacology.org/GRAC/ObjectDisplayForward?objectId=275	http://www.guidetopharmacology.org/GRAC/ObjectDisplayForward?objectId=276	http://www.guidetopharmacology.org/GRAC/ObjectDisplayForward?objectId=277	http://www.guidetopharmacology.org/GRAC/ObjectDisplayForward?objectId=278	http://www.guidetopharmacology.org/GRAC/ObjectDisplayForward?objectId=279
HGNC, UniProt	https://www.genenames.org/data/gene‐symbol‐report/#!/hgnc_id/HGNC:3165, http://www.uniprot.org/uniprot/P21453	https://www.genenames.org/data/gene‐symbol‐report/#!/hgnc_id/HGNC:3169, http://www.uniprot.org/uniprot/O95136	https://www.genenames.org/data/gene‐symbol‐report/#!/hgnc_id/HGNC:3167, http://www.uniprot.org/uniprot/Q99500	https://www.genenames.org/data/gene‐symbol‐report/#!/hgnc_id/HGNC:3170, http://www.uniprot.org/uniprot/O95977	https://www.genenames.org/data/gene‐symbol‐report/#!/hgnc_id/HGNC:14299, http://www.uniprot.org/uniprot/Q9H228
Potency order of endogenous ligands	http://www.guidetopharmacology.org/GRAC/LigandDisplayForward?ligandId=911 > http://www.guidetopharmacology.org/GRAC/LigandDisplayForward?ligandId=2921 [http://www.ncbi.nlm.nih.gov/pubmed/10383399?dopt=AbstractPlus, http://www.ncbi.nlm.nih.gov/pubmed/9765227?dopt=AbstractPlus]	http://www.guidetopharmacology.org/GRAC/LigandDisplayForward?ligandId=911 > http://www.guidetopharmacology.org/GRAC/LigandDisplayForward?ligandId=2921 [http://www.ncbi.nlm.nih.gov/pubmed/10383399?dopt=AbstractPlus, http://www.ncbi.nlm.nih.gov/pubmed/9765227?dopt=AbstractPlus]	http://www.guidetopharmacology.org/GRAC/LigandDisplayForward?ligandId=911 > http://www.guidetopharmacology.org/GRAC/LigandDisplayForward?ligandId=2921 [http://www.ncbi.nlm.nih.gov/pubmed/9765227?dopt=AbstractPlus]	http://www.guidetopharmacology.org/GRAC/LigandDisplayForward?ligandId=911 > http://www.guidetopharmacology.org/GRAC/LigandDisplayForward?ligandId=2921 [http://www.ncbi.nlm.nih.gov/pubmed/10753843?dopt=AbstractPlus]	http://www.guidetopharmacology.org/GRAC/LigandDisplayForward?ligandId=911 > http://www.guidetopharmacology.org/GRAC/LigandDisplayForward?ligandId=2921 [http://www.ncbi.nlm.nih.gov/pubmed/10799507?dopt=AbstractPlus]
Agonists	http://www.guidetopharmacology.org/GRAC/LigandDisplayForward?ligandId=2924 [http://www.ncbi.nlm.nih.gov/pubmed/11967257?dopt=AbstractPlus, http://www.ncbi.nlm.nih.gov/pubmed/14747617?dopt=AbstractPlus], http://www.guidetopharmacology.org/GRAC/LigandDisplayForward?ligandId=9289 [http://www.ncbi.nlm.nih.gov/pubmed/29735753?dopt=AbstractPlus, http://www.ncbi.nlm.nih.gov/pubmed/24900670?dopt=AbstractPlus], http://www.guidetopharmacology.org/GRAC/LigandDisplayForward?ligandId=9331 [http://www.ncbi.nlm.nih.gov/pubmed/25516790?dopt=AbstractPlus]	–	http://www.guidetopharmacology.org/GRAC/LigandDisplayForward?ligandId=2924 [http://www.ncbi.nlm.nih.gov/pubmed/11967257?dopt=AbstractPlus, http://www.ncbi.nlm.nih.gov/pubmed/14747617?dopt=AbstractPlus], http://www.guidetopharmacology.org/GRAC/LigandDisplayForward?ligandId=2924 [http://www.ncbi.nlm.nih.gov/pubmed/11967257?dopt=AbstractPlus, http://www.ncbi.nlm.nih.gov/pubmed/14747617?dopt=AbstractPlus]	http://www.guidetopharmacology.org/GRAC/LigandDisplayForward?ligandId=2924 [http://www.ncbi.nlm.nih.gov/pubmed/11967257?dopt=AbstractPlus, http://www.ncbi.nlm.nih.gov/pubmed/14747617?dopt=AbstractPlus, http://www.ncbi.nlm.nih.gov/pubmed/17114004?dopt=AbstractPlus, http://www.ncbi.nlm.nih.gov/pubmed/14732717?dopt=AbstractPlus, http://www.ncbi.nlm.nih.gov/pubmed/25347187?dopt=AbstractPlus], http://www.guidetopharmacology.org/GRAC/LigandDisplayForward?ligandId=9331 [http://www.ncbi.nlm.nih.gov/pubmed/12110609?dopt=AbstractPlus, http://www.ncbi.nlm.nih.gov/pubmed/25516790?dopt=AbstractPlus]	http://www.guidetopharmacology.org/GRAC/LigandDisplayForward?ligandId=2924 [http://www.ncbi.nlm.nih.gov/pubmed/11967257?dopt=AbstractPlus, http://www.ncbi.nlm.nih.gov/pubmed/14747617?dopt=AbstractPlus, http://www.ncbi.nlm.nih.gov/pubmed/17114004?dopt=AbstractPlus], http://www.guidetopharmacology.org/GRAC/LigandDisplayForward?ligandId=9289 [http://www.ncbi.nlm.nih.gov/pubmed/9735753?dopt=AbstractPlus, http://www.ncbi.nlm.nih.gov/pubmed/22646698?dopt=AbstractPlus, http://www.ncbi.nlm.nih.gov/pubmed/24125884?dopt=AbstractPlus], http://www.guidetopharmacology.org/GRAC/LigandDisplayForward?ligandId=9331 [http://www.ncbi.nlm.nih.gov/pubmed/25516790?dopt=AbstractPlus]
Selective agonists	http://www.guidetopharmacology.org/GRAC/LigandDisplayForward?ligandId=10309 [http://www.ncbi.nlm.nih.gov/pubmed/21445057?dopt=AbstractPlus], http://www.guidetopharmacology.org/GRAC/LigandDisplayForward?ligandId=9320 [http://www.ncbi.nlm.nih.gov/pubmed/20446681?dopt=AbstractPlus], http://www.guidetopharmacology.org/GRAC/LigandDisplayForward?ligandId=9824 [http://www.ncbi.nlm.nih.gov/pubmed/29226621?dopt=AbstractPlus], http://www.guidetopharmacology.org/GRAC/LigandDisplayForward?ligandId=2928 [http://www.ncbi.nlm.nih.gov/pubmed/18708635?dopt=AbstractPlus], http://www.guidetopharmacology.org/GRAC/LigandDisplayForward?ligandId=2926 [http://www.ncbi.nlm.nih.gov/pubmed/14732717?dopt=AbstractPlus] – Mouse	–	http://www.guidetopharmacology.org/GRAC/LigandDisplayForward?ligandId=9494 [http://www.ncbi.nlm.nih.gov/pubmed/22971058?dopt=AbstractPlus]	http://www.guidetopharmacology.org/GRAC/LigandDisplayForward?ligandId=10311 [http://www.ncbi.nlm.nih.gov/pubmed/21982495?dopt=AbstractPlus]	http://www.guidetopharmacology.org/GRAC/LigandDisplayForward?ligandId=9496 [http://www.ncbi.nlm.nih.gov/pubmed/29688337?dopt=AbstractPlus, http://www.ncbi.nlm.nih.gov/pubmed/26509640?dopt=AbstractPlus]
Antagonists	http://www.guidetopharmacology.org/GRAC/LigandDisplayForward?ligandId=3324 (p*K*_i_ 7.9) [http://www.ncbi.nlm.nih.gov/pubmed/15590668?dopt=AbstractPlus], http://www.guidetopharmacology.org/GRAC/LigandDisplayForward?ligandId=6992 (p*K*_i_ 7.6–7.7) [http://www.ncbi.nlm.nih.gov/pubmed/21632869?dopt=AbstractPlus], http://www.guidetopharmacology.org/GRAC/LigandDisplayForward?ligandId=2930 (pIC_50_ 7.6) [http://www.ncbi.nlm.nih.gov/pubmed/17113298?dopt=AbstractPlus]	–	http://www.guidetopharmacology.org/GRAC/LigandDisplayForward?ligandId=2930 (p*K*_i_ 6.5) [http://www.ncbi.nlm.nih.gov/pubmed/17113298?dopt=AbstractPlus], http://www.guidetopharmacology.org/GRAC/LigandDisplayForward?ligandId=3324 (p*K*_i_ 5.9) [http://www.ncbi.nlm.nih.gov/pubmed/15590668?dopt=AbstractPlus]	–	–
Selective antagonists	http://www.guidetopharmacology.org/GRAC/LigandDisplayForward?ligandId=6997 (pIC_50_ 8.6) [http://www.ncbi.nlm.nih.gov/pubmed/22999882?dopt=AbstractPlus], http://www.guidetopharmacology.org/GRAC/LigandDisplayForward?ligandId=2931 (p*K*_i_ 7.1) [http://www.ncbi.nlm.nih.gov/pubmed/16829954?dopt=AbstractPlus]	http://www.guidetopharmacology.org/GRAC/LigandDisplayForward?ligandId=2917 (pIC_50_ 7.8) [http://www.ncbi.nlm.nih.gov/pubmed/12445827?dopt=AbstractPlus]	http://www.guidetopharmacology.org/GRAC/LigandDisplayForward?ligandId=10310 (p*K*_i_ 7) [http://www.ncbi.nlm.nih.gov/pubmed/20097776?dopt=AbstractPlus]	http://www.guidetopharmacology.org/GRAC/LigandDisplayForward?ligandId=10312 (pIC_50_ 7.6) [http://www.ncbi.nlm.nih.gov/pubmed/23913862?dopt=AbstractPlus, http://www.ncbi.nlm.nih.gov/pubmed/21570287?dopt=AbstractPlus]	–

### Comments

The FDA‐approved immunomodulator http://www.guidetopharmacology.org/GRAC/LigandDisplayForward?ligandId=2407 (FTY720) is phosphorylated *in vivo* [http://www.ncbi.nlm.nih.gov/pubmed/16078855?dopt=AbstractPlus] to generatean agonist with activity at S1P_1_, S1P_3_, S1P_4_ and S1P_5_ receptors [http://www.ncbi.nlm.nih.gov/pubmed/11967257?dopt=AbstractPlus, http://www.ncbi.nlm.nih.gov/pubmed/11923495?dopt=AbstractPlus]. Many of the physiological consequences of http://www.guidetopharmacology.org/GRAC/LigandDisplayForward?ligandId=2924 administration, as well as those of other currently described S1P_1_ agonists, may involve functional antagonism *via* ubiquitination and subsequent degradation of S1P_1_ [http://www.ncbi.nlm.nih.gov/pubmed/17237497?dopt=AbstractPlus]. Additionally, receptor specificities of the different compounds may depend on the functional assay system utilized and from which species the receptor sequence originated.

### Further reading on Lysophospholipid (S1P) receptors

Chew WS *et al*. (2016) To fingolimod and beyond: The rich pipeline of drug candidates that target S1P signaling. *Pharmacol. Res.* **113**: 521‐532 https://www.ncbi.nlm.nih.gov/pubmed/27663260?dopt=AbstractPlus

Chun J *et al*. (2010) International Union of Basic and Clinical Pharmacology. LXXVIII. Lysophospholipid receptor nomenclature. *Pharmacol. Rev.* **62**: 579‐87 https://www.ncbi.nlm.nih.gov/pubmed/21079037?dopt=AbstractPlus

Cyster JG *et al*. (2012) Sphingosine‐1‐phosphate and lymphocyte egress from lymphoid organs. *Annu. Rev. Immunol.* **30**: 69‐94 https://www.ncbi.nlm.nih.gov/pubmed/22149932?dopt=AbstractPlus

Pyne NJ *et al*. (2017) Sphingosine 1‐Phosphate Receptor 1 Signaling in Mammalian Cells. *Molecules* **22**: https://www.ncbi.nlm.nih.gov/pubmed/28241498?dopt=AbstractPlus

Rosen H *et al*. (2013) Sphingosine‐1‐phosphate and its receptors: structure, signaling, and influence. *Annu. Rev. Biochem.* **82**: 637‐62 https://www.ncbi.nlm.nih.gov/pubmed/23527695?dopt=AbstractPlus

Yanagida K *et al*. (2017) Vascular and Immunobiology of the Circulatory Sphingosine 1‐Phosphate Gradient. *Annu. Rev. Physiol.* **79**: 67‐91 https://www.ncbi.nlm.nih.gov/pubmed/27813829?dopt=AbstractPlus

## http://www.guidetopharmacology.org/GRAC/FamilyDisplayForward?familyId=37

### Overview

Melanin‐concentrating hormone (MCH) receptors (**provisional nomenclature as recommended by** NC‐IUPHAR [http://www.ncbi.nlm.nih.gov/pubmed/15914470?dopt=AbstractPlus]) are activated by an endogenous nonadecameric cyclic peptide identical in humans and rats (DFDMLRCMLGRVYRPCWQV; mammalian MCH) generated from a precursor (https://www.genenames.org/data/gene‐symbol‐report/#!/hgnc_id/HGNC:9109, http://www.uniprot.org/uniprot/P20382), which also produces http://www.guidetopharmacology.org/GRAC/LigandDisplayForward?ligandId=5374 (https://www.genenames.org/data/gene‐symbol‐report/#!/hgnc_id/HGNC:9109, http://www.uniprot.org/uniprot/P20382) and http://www.guidetopharmacology.org/GRAC/LigandDisplayForward?ligandId=5375 (https://www.genenames.org/data/gene‐symbol‐report/#!/hgnc_id/HGNC:9109, http://www.uniprot.org/uniprot/P20382).

**Table:** 

Nomenclature	http://www.guidetopharmacology.org/GRAC/ObjectDisplayForward?objectId=280	http://www.guidetopharmacology.org/GRAC/ObjectDisplayForward?objectId=281
HGNC, UniProt	https://www.genenames.org/data/gene‐symbol‐report/#!/hgnc_id/HGNC:4479, http://www.uniprot.org/uniprot/Q99705	https://www.genenames.org/data/gene‐symbol‐report/#!/hgnc_id/HGNC:20867, http://www.uniprot.org/uniprot/Q969V1
Selective antagonists	http://www.guidetopharmacology.org/GRAC/LigandDisplayForward?ligandId=4033 (pIC_50_ 9.3) [http://www.ncbi.nlm.nih.gov/pubmed/16870432?dopt=AbstractPlus], http://www.guidetopharmacology.org/GRAC/LigandDisplayForward?ligandId=1313 (p*A*_2_ 9.2) [http://www.ncbi.nlm.nih.gov/pubmed/12118247?dopt=AbstractPlus], http://www.guidetopharmacology.org/GRAC/LigandDisplayForward?ligandId=1314 (pIC_50_ 8.3) [http://www.ncbi.nlm.nih.gov/pubmed/11909603?dopt=AbstractPlus], http://www.guidetopharmacology.org/GRAC/LigandDisplayForward?ligandId=1305 (pIC_50_ 7.9–8.1) [http://www.ncbi.nlm.nih.gov/pubmed/15677346?dopt=AbstractPlus]	–
Labelled ligands	[http://www.guidetopharmacology.org/GRAC/LigandDisplayForward?ligandId=1317 http://www.guidetopharmacology.org/GRAC/LigandDisplayForward?ligandId=1317 (Antagonist) (p*K*_d_ 9.2–9.5) [http://www.ncbi.nlm.nih.gov/pubmed/11375253?dopt=AbstractPlus], [http://www.guidetopharmacology.org/GRAC/LigandDisplayForward?ligandId=3844 http://www.guidetopharmacology.org/GRAC/LigandDisplayForward?ligandId=3844,http://www.guidetopharmacology.org/GRAC/LigandDisplayForward?ligandId=3844]http://www.guidetopharmacology.org/GRAC/LigandDisplayForward?ligandId=3844 (Agonist) [http://www.ncbi.nlm.nih.gov/pubmed/9434758?dopt=AbstractPlus], [http://www.guidetopharmacology.org/GRAC/LigandDisplayForward?ligandId=3827 http://www.guidetopharmacology.org/GRAC/LigandDisplayForward?ligandId=3827) (Agonist) [http://www.ncbi.nlm.nih.gov/pubmed/9434758?dopt=AbstractPlus]	–

### Comments

The MCH_2_ receptor appears to be a non‐functional pseudogene in rodents [http://www.ncbi.nlm.nih.gov/pubmed/12036292?dopt=AbstractPlus].

### Further reading on Melanin‐concentrating hormone receptors

Chung S *et al*. (2011) Recent updates on the melanin‐concentrating hormone (MCH) and its receptor system: lessons from MCH1R antagonists. *J. Mol. Neurosci.* **43**: 115‐21 [https://www.ncbi.nlm.nih.gov/pubmed/20582487?dopt=AbstractPlus]

Eberle AN *et al*. (2010) Cellular models for the study of the pharmacology and signaling of melaninconcentrating hormone receptors. *J. Recept. Signal Transduct. Res.* **30**: 385‐402 [https://www.ncbi.nlm.nih.gov/pubmed/21083507?dopt=AbstractPlus]

Foord SM *et al*. (2005) International Union of Pharmacology. XLVI. G protein‐coupled receptor list. *Pharmacol Rev* **57**: 279‐288 [https://www.ncbi.nlm.nih.gov/pubmed/15914470?dopt=AbstractPlus]

Takase K *et al*. (2014) Meta‐analysis of melanin‐concentrating hormone signaling‐deficient mice on behavioral and metabolic phenotypes. *PLoS ONE* **9**: e99961 [https://www.ncbi.nlm.nih.gov/pubmed/24924345?dopt=AbstractPlus]

## http://www.guidetopharmacology.org/GRAC/FamilyDisplayForward?familyId=38

### Overview

Melanocortin receptors (**provisional nomenclature as recommended by** NC‐IUPHAR [http://www.ncbi.nlm.nih.gov/pubmed/15914470?dopt=AbstractPlus]) are activated by members of the melanocortin family (http://www.guidetopharmacology.org/GRAC/LigandDisplayForward?ligandId=1320 (https://www.genenames.org/data/gene‐symbol‐report/#!/hgnc_id/HGNC:9201, http://www.uniprot.org/uniprot/P01189), http://www.guidetopharmacology.org/GRAC/LigandDisplayForward?ligandId=3606‐http://www.guidetopharmacology.org/GRAC/LigandDisplayForward?ligandId=3606, http://www.uniprot.org/uniprot/P01189) and http://www.guidetopharmacology.org/GRAC/LigandDisplayForward?ligandId=1333‐http://www.guidetopharmacology.org/GRAC/LigandDisplayForward?ligandId=1333, http://www.uniprot.org/uniprot/P01189) forms; *δ* form is not found in mammals) and adrenocorticotrophin (http://www.guidetopharmacology.org/GRAC/LigandDisplayForward?ligandId=3633, http://www.uniprot.org/uniprot/P01189)). Endogenous antagonists include http://www.guidetopharmacology.org/GRAC/LigandDisplayForward?ligandId=3609 (https://www.genenames.org/data/gene‐symbol‐report/#!/hgnc_id/HGNC:745, http://www.uniprot.org/uniprot/P42127) and http://www.guidetopharmacology.org/GRAC/LigandDisplayForward?ligandId=1335 (https://www.genenames.org/data/gene‐symbol‐report/#!/hgnc_id/HGNC:330, http://www.uniprot.org/uniprot/O00253). ACTH(1‐24) was approved by the US FDA as a diagnostic agent for adrenal function test, whilst NDP‐MSH was approved by EMA for the treatment of erythropoietic protoporphyria. Several synthetic melanocortin receptor agonists are under clinical development.

**Table:** 

Nomenclature	http://www.guidetopharmacology.org/GRAC/ObjectDisplayForward?objectId=282	http://www.guidetopharmacology.org/GRAC/ObjectDisplayForward?objectId=283	http://www.guidetopharmacology.org/GRAC/ObjectDisplayForward?objectId=284	http://www.guidetopharmacology.org/GRAC/ObjectDisplayForward?objectId=285	http://www.guidetopharmacology.org/GRAC/ObjectDisplayForward?objectId=286
HGNC, UniProt	https://www.genenames.org/data/gene‐symbol‐report/#!/hgnc_id/HGNC:6929, http://www.uniprot.org/uniprot/Q01726	https://www.genenames.org/data/gene‐symbol‐report/#!/hgnc_id/HGNC:6930, http://www.uniprot.org/uniprot/Q01718	https://www.genenames.org/data/gene‐symbol‐report/#!/hgnc_id/HGNC:6931, http://www.uniprot.org/uniprot/P41968	https://www.genenames.org/data/gene‐symbol‐report/#!/hgnc_id/HGNC:6932, http://www.uniprot.org/uniprot/P32245	https://www.genenames.org/data/gene‐symbol‐report/#!/hgnc_id/HGNC:6933, http://www.uniprot.org/uniprot/P33032
Potency order of endogenous agonists	http://www.guidetopharmacology.org/GRAC/LigandDisplayForward?ligandId=1320 (https://www.genenames.org/data/gene‐symbol‐report/#!/hgnc_id/HGNC:9201, http://www.uniprot.org/uniprot/P01189) > http://www.guidetopharmacology.org/GRAC/LigandDisplayForward?ligandId=3606‐http://www.guidetopharmacology.org/GRAC/LigandDisplayForward?ligandId=3606, http://www.uniprot.org/uniprot/P01189) > http://www.guidetopharmacology.org/GRAC/LigandDisplayForward?ligandId=3633, http://www.uniprot.org/uniprot/P01189), http://www.guidetopharmacology.org/GRAC/LigandDisplayForward?ligandId=1333‐http://www.guidetopharmacology.org/GRAC/LigandDisplayForward?ligandId=1333, http://www.uniprot.org/uniprot/P01189)	http://www.guidetopharmacology.org/GRAC/LigandDisplayForward?ligandId=3633 (https://www.genenames.org/data/gene‐symbol‐report/#!/hgnc_id/HGNC:9201, http://www.uniprot.org/uniprot/P01189)	http://www.guidetopharmacology.org/GRAC/LigandDisplayForward?ligandId=1333 (https://www.genenames.org/data/gene‐symbol‐report/#!/hgnc_id/HGNC:9201, http://www.uniprot.org/uniprot/P01189), http://www.guidetopharmacology.org/GRAC/LigandDisplayForward?ligandId=3606‐http://www.guidetopharmacology.org/GRAC/LigandDisplayForward?ligandId=3606, http://www.uniprot.org/uniprot/P01189) > http://www.guidetopharmacology.org/GRAC/LigandDisplayForward?ligandId=3633, http://www.uniprot.org/uniprot/P01189), http://www.guidetopharmacology.org/GRAC/LigandDisplayForward?ligandId=1320‐http://www.guidetopharmacology.org/GRAC/LigandDisplayForward?ligandId=1320, http://www.uniprot.org/uniprot/P01189)	http://www.guidetopharmacology.org/GRAC/LigandDisplayForward?ligandId=3606 (https://www.genenames.org/data/gene‐symbol‐report/#!/hgnc_id/HGNC:9201, http://www.uniprot.org/uniprot/P01189) > http://www.guidetopharmacology.org/GRAC/LigandDisplayForward?ligandId=1320‐http://www.guidetopharmacology.org/GRAC/LigandDisplayForward?ligandId=1320, http://www.uniprot.org/uniprot/P01189), http://www.guidetopharmacology.org/GRAC/LigandDisplayForward?ligandId=3633, http://www.uniprot.org/uniprot/P01189) > http://www.guidetopharmacology.org/GRAC/LigandDisplayForward?ligandId=1333‐http://www.guidetopharmacology.org/GRAC/LigandDisplayForward?ligandId=1333, http://www.uniprot.org/uniprot/P01189)	http://www.guidetopharmacology.org/GRAC/LigandDisplayForward?ligandId=1320 (https://www.genenames.org/data/gene‐symbol‐report/#!/hgnc_id/HGNC:9201, http://www.uniprot.org/uniprot/P01189) > http://www.guidetopharmacology.org/GRAC/LigandDisplayForward?ligandId=3606‐http://www.guidetopharmacology.org/GRAC/LigandDisplayForward?ligandId=3606, http://www.uniprot.org/uniprot/P01189) > http://www.guidetopharmacology.org/GRAC/LigandDisplayForward?ligandId=3633, http://www.uniprot.org/uniprot/P01189) >http://www.guidetopharmacology.org/GRAC/LigandDisplayForward?ligandId=1333‐http://www.guidetopharmacology.org/GRAC/LigandDisplayForward?ligandId=1333, http://www.uniprot.org/uniprot/P01189)
Selective agonists	–	http://www.guidetopharmacology.org/GRAC/LigandDisplayForward?ligandId=6967	[http://www.guidetopharmacology.org/GRAC/LigandDisplayForward?ligandId=1334]http://www.guidetopharmacology.org/GRAC/LigandDisplayForward?ligandId=1334‐http://www.guidetopharmacology.org/GRAC/LigandDisplayForward?ligandId=1334 [http://www.ncbi.nlm.nih.gov/pubmed/11150170?dopt=AbstractPlus]	http://www.guidetopharmacology.org/GRAC/LigandDisplayForward?ligandId=1338 [http://www.ncbi.nlm.nih.gov/pubmed/12361385?dopt=AbstractPlus]	–
Antagonists	–	–	http://www.guidetopharmacology.org/GRAC/LigandDisplayForward?ligandId=6546 (pIC_50_ 6.7) [http://www.ncbi.nlm.nih.gov/pubmed/17482720?dopt=AbstractPlus]	–	–
Selective antagonists	–	–	–	http://www.guidetopharmacology.org/GRAC/LigandDisplayForward?ligandId=1339 (pIC_50_ 10) [http://www.ncbi.nlm.nih.gov/pubmed/11606131?dopt=AbstractPlus], http://www.guidetopharmacology.org/GRAC/LigandDisplayForward?ligandId=1321 (p*K*_i_ 8.5) [http://www.ncbi.nlm.nih.gov/pubmed/9630346?dopt=AbstractPlus]	–
Labelled ligands	[http://www.guidetopharmacology.org/GRAC/LigandDisplayForward?ligandId=1326 http://www.guidetopharmacology.org/GRAC/LigandDisplayForward?ligandId=1326 (Agonist) [http://www.ncbi.nlm.nih.gov/pubmed/15840392?dopt=AbstractPlus]	[http://www.guidetopharmacology.org/GRAC/LigandDisplayForward?ligandId=3767 http://www.guidetopharmacology.org/GRAC/LigandDisplayForward?ligandId=3767) (Agonist)	[http://www.guidetopharmacology.org/GRAC/LigandDisplayForward?ligandId=1326 http://www.guidetopharmacology.org/GRAC/LigandDisplayForward?ligandId=1326 (Agonist) [http://www.ncbi.nlm.nih.gov/pubmed/15840392?dopt=AbstractPlus], [http://www.guidetopharmacology.org/GRAC/LigandDisplayForward?ligandId=3804 http://www.guidetopharmacology.org/GRAC/LigandDisplayForward?ligandId=3804 (Antagonist) [http://www.ncbi.nlm.nih.gov/pubmed/12604699?dopt=AbstractPlus]	[http://www.guidetopharmacology.org/GRAC/LigandDisplayForward?ligandId=3804 http://www.guidetopharmacology.org/GRAC/LigandDisplayForward?ligandId=3804 (Antagonist) (p*K*_d_ 9.2) [http://www.ncbi.nlm.nih.gov/pubmed/12604699?dopt=AbstractPlus], [http://www.guidetopharmacology.org/GRAC/LigandDisplayForward?ligandId=1326 http://www.guidetopharmacology.org/GRAC/LigandDisplayForward?ligandId=1326 (Agonist) [http://www.ncbi.nlm.nih.gov/pubmed/15840392?dopt=AbstractPlus, http://www.ncbi.nlm.nih.gov/pubmed/7774675?dopt=AbstractPlus]	[http://www.guidetopharmacology.org/GRAC/LigandDisplayForward?ligandId=1326 http://www.guidetopharmacology.org/GRAC/LigandDisplayForward?ligandId=1326 (Agonist) [http://www.ncbi.nlm.nih.gov/pubmed/15840392?dopt=AbstractPlus]

### Comments

Polymorphisms of the MC_1_ receptor have been linked to variations in skin pigmentation. Defects of the MC_2_ receptor underlie familial glucocorticoid deficiency. Polymorphisms of the MC4 receptor have been linked to obesity [http://www.ncbi.nlm.nih.gov/pubmed/9392003?dopt=AbstractPlus, http://www.ncbi.nlm.nih.gov/pubmed/18779842?dopt=AbstractPlus].

### Further reading on Melanocortin receptors

Caruso V *et al*. (2014) Synaptic changes induced by melanocortin signalling. *Nat. Rev. Neurosci.* **15**: 98‐110 [https://www.ncbi.nlm.nih.gov/pubmed/24588018?dopt=AbstractPlus]

Foord SM *et al*. (2005) International Union of Pharmacology. XLVI. G protein‐coupled receptor list. *Pharmacol Rev* **57**: 279‐288 [https://www.ncbi.nlm.nih.gov/pubmed/15914470?dopt=AbstractPlus]

Renquist BJ *et al*. (2011) Physiological roles of the melanocortin MC_3 receptor. *Eur. J. Pharmacol.* **660**: 13‐20 [https://www.ncbi.nlm.nih.gov/pubmed/21211527?dopt=AbstractPlus]

## http://www.guidetopharmacology.org/GRAC/FamilyDisplayForward?familyId=39

### Overview

Melatonin receptors (**nomenclature as agreed by the NC‐IUPHAR Subcommittee on Melatonin Receptors** [http://www.ncbi.nlm.nih.gov/pubmed/20605968?dopt=AbstractPlus]) are activated by the endogenous ligands http://www.guidetopharmacology.org/GRAC/LigandDisplayForward?ligandId=224 and clinically used drugs like http://www.guidetopharmacology.org/GRAC/LigandDisplayForward?ligandId=1356, http://www.guidetopharmacology.org/GRAC/LigandDisplayForward?ligandId=198 and http://www.guidetopharmacology.org/GRAC/LigandDisplayForward?ligandId=7393.

**Table:** 

Nomenclature	http://www.guidetopharmacology.org/GRAC/ObjectDisplayForward?objectId=287	http://www.guidetopharmacology.org/GRAC/ObjectDisplayForward?objectId=288
HGNC, UniProt	https://www.genenames.org/data/gene‐symbol‐report/#!/hgnc_id/HGNC:7463, http://www.uniprot.org/uniprot/P48039	https://www.genenames.org/data/gene‐symbol‐report/#!/hgnc_id/HGNC:7464, http://www.uniprot.org/uniprot/P49286
Endogenous agonists	http://www.guidetopharmacology.org/GRAC/LigandDisplayForward?ligandId=224 [http://www.ncbi.nlm.nih.gov/pubmed/12764576?dopt=AbstractPlus, http://www.ncbi.nlm.nih.gov/pubmed/2991499?dopt=AbstractPlus, http://www.ncbi.nlm.nih.gov/pubmed/9089668?dopt=AbstractPlus]	http://www.guidetopharmacology.org/GRAC/LigandDisplayForward?ligandId=224 [http://www.ncbi.nlm.nih.gov/pubmed/12764576?dopt=AbstractPlus, http://www.ncbi.nlm.nih.gov/pubmed/2991499?dopt=AbstractPlus, http://www.ncbi.nlm.nih.gov/pubmed/9089668?dopt=AbstractPlus]
Agonists	http://www.guidetopharmacology.org/GRAC/LigandDisplayForward?ligandId=1356 [http://www.ncbi.nlm.nih.gov/pubmed/15695169?dopt=AbstractPlus], http://www.guidetopharmacology.org/GRAC/LigandDisplayForward?ligandId=198 [http://www.ncbi.nlm.nih.gov/pubmed/12764576?dopt=AbstractPlus, http://www.ncbi.nlm.nih.gov/pubmed/9618428?dopt=AbstractPlus], http://www.guidetopharmacology.org/GRAC/LigandDisplayForward?ligandId=7393 [http://www.ncbi.nlm.nih.gov/pubmed/19054552?dopt=AbstractPlus]	http://www.guidetopharmacology.org/GRAC/LigandDisplayForward?ligandId=198 [http://www.ncbi.nlm.nih.gov/pubmed/12764576?dopt=AbstractPlus, http://www.ncbi.nlm.nih.gov/pubmed/9618428?dopt=AbstractPlus], http://www.guidetopharmacology.org/GRAC/LigandDisplayForward?ligandId=1356 [http://www.ncbi.nlm.nih.gov/pubmed/15695169?dopt=AbstractPlus, http://www.ncbi.nlm.nih.gov/pubmed/21182402?dopt=AbstractPlus], http://www.guidetopharmacology.org/GRAC/LigandDisplayForward?ligandId=7393 [http://www.ncbi.nlm.nih.gov/pubmed/19054552?dopt=AbstractPlus]
Selective agonists	–	http://www.guidetopharmacology.org/GRAC/LigandDisplayForward?ligandId=9226 [http://www.ncbi.nlm.nih.gov/pubmed/26334942?dopt=AbstractPlus], http://www.guidetopharmacology.org/GRAC/LigandDisplayForward?ligandId=1350 [http://www.ncbi.nlm.nih.gov/pubmed/10737738?dopt=AbstractPlus, http://www.ncbi.nlm.nih.gov/pubmed/10420436?dopt=AbstractPlus], http://www.guidetopharmacology.org/GRAC/LigandDisplayForward?ligandId=1364 (Partial agonist) [http://www.ncbi.nlm.nih.gov/pubmed/9089668?dopt=AbstractPlus]
Selective antagonists	–	http://www.guidetopharmacology.org/GRAC/LigandDisplayForward?ligandId=1358 (p*K*_i_ 8.8–9.4) [http://www.ncbi.nlm.nih.gov/pubmed/12764576?dopt=AbstractPlus, http://www.ncbi.nlm.nih.gov/pubmed/9089668?dopt=AbstractPlus, http://www.ncbi.nlm.nih.gov/pubmed/9737724?dopt=AbstractPlus], http://www.guidetopharmacology.org/GRAC/LigandDisplayForward?ligandId=1359 (p*K*_i_ 9.3) [http://www.ncbi.nlm.nih.gov/pubmed/10737738?dopt=AbstractPlus, http://www.ncbi.nlm.nih.gov/pubmed/10420436?dopt=AbstractPlus], http://www.guidetopharmacology.org/GRAC/LigandDisplayForward?ligandId=3366 (p*K*_i_ 8) [http://www.ncbi.nlm.nih.gov/pubmed/9840420?dopt=AbstractPlus]
Labelled ligands	[http://www.guidetopharmacology.org/GRAC/LigandDisplayForward?ligandId=7770 http://www.guidetopharmacology.org/GRAC/LigandDisplayForward?ligandId=7770 (Agonist) [http://www.ncbi.nlm.nih.gov/pubmed/23698757?dopt=AbstractPlus], http://www.guidetopharmacology.org/GRAC/LigandDisplayForward?ligandId=1344 http://www.guidetopharmacology.org/GRAC/LigandDisplayForward?ligandId=1344 (Agonist) [http://www.ncbi.nlm.nih.gov/pubmed/12764576?dopt=AbstractPlus, http://www.ncbi.nlm.nih.gov/pubmed/9089668?dopt=AbstractPlus], [http://www.guidetopharmacology.org/GRAC/LigandDisplayForward?ligandId=1357 http://www.guidetopharmacology.org/GRAC/LigandDisplayForward?ligandId=1357 (Agonist) [http://www.ncbi.nlm.nih.gov/pubmed/10696085?dopt=AbstractPlus]	[http://www.guidetopharmacology.org/GRAC/LigandDisplayForward?ligandId=7770 http://www.guidetopharmacology.org/GRAC/LigandDisplayForward?ligandId=7770 (Agonist) [http://www.ncbi.nlm.nih.gov/pubmed/23698757?dopt=AbstractPlus], http://www.guidetopharmacology.org/GRAC/LigandDisplayForward?ligandId=1344 http://www.guidetopharmacology.org/GRAC/LigandDisplayForward?ligandId=1344 (Agonist) [http://www.ncbi.nlm.nih.gov/pubmed/12764576?dopt=AbstractPlus, http://www.ncbi.nlm.nih.gov/pubmed/9089668?dopt=AbstractPlus], [http://www.guidetopharmacology.org/GRAC/LigandDisplayForward?ligandId=7771 http://www.guidetopharmacology.org/GRAC/LigandDisplayForward?ligandId=7771 (Agonist, Partial agonist) [http://www.ncbi.nlm.nih.gov/pubmed/23698757?dopt=AbstractPlus], [http://www.guidetopharmacology.org/GRAC/LigandDisplayForward?ligandId=1357 http://www.guidetopharmacology.org/GRAC/LigandDisplayForward?ligandId=1357 (Agonist) [http://www.ncbi.nlm.nih.gov/pubmed/10696085?dopt=AbstractPlus]

### Comments

http://www.guidetopharmacology.org/GRAC/LigandDisplayForward?ligandId=224, http://www.guidetopharmacology.org/GRAC/LigandDisplayForward?ligandId=1343, http://www.guidetopharmacology.org/GRAC/LigandDisplayForward?ligandId=198, http://www.guidetopharmacology.org/GRAC/LigandDisplayForward?ligandId=1349, http://www.guidetopharmacology.org/GRAC/LigandDisplayForward?ligandId=1351 and http://www.guidetopharmacology.org/GRAC/LigandDisplayForward?ligandId=1356 [http://www.ncbi.nlm.nih.gov/pubmed/15695169?dopt=AbstractPlus] are nonselective agonists for MT_1_ and MT_2_ receptors. (‐)‐AMMTC displays an ˜400‐fold greater agonist potency than (+)‐AMMTC at rat MT_1_ receptors (see http://www.guidetopharmacology.org/GRAC/LigandDisplayForward?ligandId=3385 for structure) [http://www.ncbi.nlm.nih.gov/pubmed/10433507?dopt=AbstractPlus]. http://www.guidetopharmacology.org/GRAC/LigandDisplayForward?ligandId=1363 is an MT_1_/MT_2_ non‐selective competitive melatonin receptor antagonist with about 15‐25 fold selectivity for the MT_2_ receptor [http://www.ncbi.nlm.nih.gov/pubmed/9737724?dopt=AbstractPlus]. MT_1_/MT_2_ heterodimers present differentpharmacological profiles from MT_1_ and MT_2_ receptors [http://www.ncbi.nlm.nih.gov/pubmed/15266022?dopt=AbstractPlus]. The *MT3* binding site of hamster brain and peripheral tissues such as kidney and testis, also termed the ML_2_ receptor, binds selectively http://www.guidetopharmacology.org/GRAC/LigandDisplayForward?ligandId=5396 http://www.guidetopharmacology.org/GRAC/LigandDisplayForward?ligandId=5396 [http://www.ncbi.nlm.nih.gov/pubmed/8773460?dopt=AbstractPlus]. Pharmacological investigations of *MT_3_* binding sites have primarily been conducted in hamster tissues. At this site, The endogenous ligand http://www.guidetopharmacology.org/GRAC/LigandDisplayForward?ligandId=5451 [http://www.ncbi.nlm.nih.gov/pubmed/8246675?dopt=AbstractPlus, http://www.ncbi.nlm.nih.gov/pubmed/9283717?dopt=AbstractPlus, http://www.ncbi.nlm.nih.gov/pubmed/9283717?dopt=AbstractPlus, http://www.ncbi.nlm.nih.gov/pubmed/7798906?dopt=AbstractPlus] and http://www.guidetopharmacology.org/GRAC/LigandDisplayForward?ligandId=3393 [http://www.ncbi.nlm.nih.gov/pubmed/7798906?dopt=AbstractPlus] appear to function as agonists, while http://www.guidetopharmacology.org/GRAC/LigandDisplayForward?ligandId=503 [http://www.ncbi.nlm.nih.gov/pubmed/9283717?dopt=AbstractPlus] functions as an antagonist. The *MT_3_* binding site of hamster kidney was also identified as the hamster homologue of human quinone reductase 2 (https://www.genenames.org/data/gene‐symbol‐report/#!/hgnc_id/HGNC:7856, http://www.uniprot.org/uniprot/P16083 [http://www.ncbi.nlm.nih.gov/pubmed/10913150?dopt=AbstractPlus, http://www.ncbi.nlm.nih.gov/pubmed/11331072?dopt=AbstractPlus]). The *MT_3_* binding site activated by http://www.guidetopharmacology.org/GRAC/LigandDisplayForward?ligandId=3393 in eye ciliary body is positively coupled to adenylyl cyclase and regulates chloride secretion [http://www.ncbi.nlm.nih.gov/pubmed/25344385?dopt=AbstractPlus]. *Xenopus* melanophores and chick brain express a distinct receptor (x420, P49219; c346, P49288, initially termed Mel_1C_) coupled to the G_i/o_ family of G proteins, for which GPR50 has recently been suggested to be a mammalian counterpart [http://www.ncbi.nlm.nih.gov/pubmed/18400093?dopt=AbstractPlus] although http://www.guidetopharmacology.org/GRAC/LigandDisplayForward?ligandId=224 does not bind to GPR50 receptors. Several variants of the *MTNR1B* gene have been associated with increased type 2 diabetes risk [http://www.ncbi.nlm.nih.gov/pubmed/30531911?dopt=AbstractPlus].

### Further reading on Melatonin receptors

Cecon E *et al*. (2018) Melatonin receptors: molecular pharmacology and signalling in the context of system bias. *Br. J. Pharmacol.* **175**: 3263‐3280 [https://www.ncbi.nlm.nih.gov/pubmed/28707298?dopt=AbstractPlus]

Dubocovich ML *et al*. (2010) International Union of Basic and Clinical Pharmacology. LXXV. Nomenclature, classification, and pharmacology of G protein‐coupled melatonin receptors. *Pharmacol. Rev.* **62**: 343‐80 [https://www.ncbi.nlm.nih.gov/pubmed/20605968?dopt=AbstractPlus]

Jockers R *et al*. (2016) Update on melatonin receptors: IUPHAR Review 20. *Br. J. Pharmacol.* **173**: 2702‐25 [https://www.ncbi.nlm.nih.gov/pubmed/27314810?dopt=AbstractPlus]

Karamitri A *et al*. (2019) Melatonin in type 2 diabetes mellitus and obesity. *Nat Rev Endocrinol* **15**: 105‐125 [https://www.ncbi.nlm.nih.gov/pubmed/30531911?dopt=AbstractPlus]

Liu J *et al*. (2016) MT1 and MT2 Melatonin Receptors: A Therapeutic Perspective. *Annu. Rev. Pharmacol. Toxicol.* **56**: 361‐83 [https://www.ncbi.nlm.nih.gov/pubmed/26514204?dopt=AbstractPlus]

Zlotos DP *et al*. (2014) MT1 and MT2 melatonin receptors: ligands, models, oligomers, and therapeutic potential. *J. Med. Chem.* **57**: 3161‐85 [https://www.ncbi.nlm.nih.gov/pubmed/24228714?dopt=AbstractPlus]

## http://www.guidetopharmacology.org/GRAC/FamilyDisplayForward?familyId=40

### Overview

Metabotropic glutamate (mGlu) receptors (**nomenclature as agreed by the NC‐IUPHAR Subcommittee on Metabotropic Glutamate Receptors[1899]**) area family of G protein‐coupled receptors activated by the neurotransmitter glutamate. The mGlu family is composed of eight members (named mGlu1 to mGlu8) which are divided in three groups based on similarities of agonist pharmacology, primary sequence and G protein coupling to effector: Group‐I (mGlu_1_ and mGlu_5_), Group‐II (mGlu_2_ and mGlu_3_) and Group‐III (mGlu_4_, mGlu_6_, mGlu_7_ and mGlu_8_) (see Further reading).

Structurally, mGlu are composed of three juxtaposed domains: a core G protein‐activating seven‐transmembrane domain (TM), common to all GPCRs, is linked via a rigid cysteine‐rich domain (CRD) to the Venus Flytrap domain (VFTD), a large bi‐lobed extracellular domain where glutamate binds. The structures of the VFTD of mGlu_1_, mGlu_2_, mGlu_3_, mGlu5 and mGlu7 have been solved [http://www.ncbi.nlm.nih.gov/pubmed/11069170?dopt=AbstractPlus, http://www.ncbi.nlm.nih.gov/pubmed/25602126?dopt=AbstractPlus, http://www.ncbi.nlm.nih.gov/pubmed/17360426?dopt=AbstractPlus, http://www.ncbi.nlm.nih.gov/pubmed/11867751?dopt=AbstractPlus]. The structure of the 7 transmembrane (TM) domains of both mGlu1 and mGlu5 have been solved, and confirm a general helical organization similar to that of other GPCRs, although the helices appear more compacted [http://www.ncbi.nlm.nih.gov/pubmed/29455526?dopt=AbstractPlus, http://www.ncbi.nlm.nih.gov/pubmed/25042998?dopt=AbstractPlus, http://www.ncbi.nlm.nih.gov/pubmed/24603153?dopt=AbstractPlus]. mGlu form constitutive dimers crosslinked by a disulfide bridge. Recent studies revealed the possible formation of heterodimers between either group‐I receptors, or within and between group‐II and ‐III receptors [http://www.ncbi.nlm.nih.gov/pubmed/20826542?dopt=AbstractPlus]. Although well characterized in transfected cells, co‐localization and specific pharmacological properties also suggest the existence of such heterodimers in the brain [http://www.ncbi.nlm.nih.gov/pubmed/28661401?dopt=AbstractPlus, http://www.ncbi.nlm.nih.gov/pubmed/24381270?dopt=AbstractPlus].

The endogenous ligands of mGlu are http://www.guidetopharmacology.org/GRAC/LigandDisplayForward?ligandId=1369, http://www.guidetopharmacology.org/GRAC/LigandDisplayForward?ligandId=1411, N‐acetylaspartylglutamate (http://www.guidetopharmacology.org/GRAC/LigandDisplayForward?ligandId=1405) and http://www.guidetopharmacology.org/GRAC/LigandDisplayForward?ligandId=5447. Group‐I mGlu receptors may be activated by http://www.guidetopharmacology.org/GRAC/LigandDisplayForward?ligandId=1367 and (http://www.guidetopharmacology.org/GRAC/LigandDisplayForward?ligandId=1366)http://www.guidetopharmacology.org/GRAC/LigandDisplayForward?ligandId=1366 [http://www.ncbi.nlm.nih.gov/pubmed/8532171?dopt=AbstractPlus] and antagonized by (http://www.guidetopharmacology.org/GRAC/LigandDisplayForward?ligandId=5448 [http://www.ncbi.nlm.nih.gov/pubmed/15996690?dopt=AbstractPlus]. Group‐II mGlu receptors may be activated by http://www.guidetopharmacology.org/GRAC/LigandDisplayForward?ligandId=3349 [http://www.ncbi.nlm.nih.gov/pubmed/10090786?dopt=AbstractPlus], http://www.guidetopharmacology.org/GRAC/LigandDisplayForward?ligandId=1394], http://www.guidetopharmacology.org/GRAC/LigandDisplayForward?ligandId=1393 [http://www.ncbi.nlm.nih.gov/pubmed/9144636?dopt=AbstractPlus http://www.ncbi.nlm.nih.gov/pubmed/9473604?dopt=AbstractPlus], http://www.guidetopharmacology.org/GRAC/LigandDisplayForward?ligandId=1377 and (http://www.guidetopharmacology.org/GRAC/LigandDisplayForward?ligandId=1392,http://www.guidetopharmacology.org/GRAC/LigandDisplayForward?ligandId=1392)http://www.guidetopharmacology.org/GRAC/LigandDisplayForward?ligandId=1392 [http://www.ncbi.nlm.nih.gov/pubmed/9076745?dopt=AbstractPlus, and antagonised by http://www.guidetopharmacology.org/GRAC/LigandDisplayForward?ligandId=1400 [http://www.ncbi.nlm.nih.gov/pubmed/9121605?dopt=AbstractPlus] and http://www.guidetopharmacology.org/GRAC/LigandDisplayForward?ligandId=3350 [http://www.ncbi.nlm.nih.gov/pubmed/9871538?dopt=AbstractPlus, http://www.ncbi.nlm.nih.gov/pubmed/8632404?dopt=AbstractPlus]. Group‐III mGlu receptors may be activated by http://www.guidetopharmacology.org/GRAC/LigandDisplayForward?ligandId=1410 and (http://www.guidetopharmacology.org/GRAC/LigandDisplayForward?ligandId=1406)http://www.guidetopharmacology.org/GRAC/LigandDisplayForward?ligandId=1406 [http://www.ncbi.nlm.nih.gov/pubmed/10336568?dopt=AbstractPlus]. An example of an antagonist selective for mGlu receptors is http://www.guidetopharmacology.org/GRAC/LigandDisplayForward?ligandId=1378, which blocks mGlu_2_ and mGlu_3_ at low nanomolar concentrations, mGlu_8_ at high nanomolar concentrations, and mGlu4, mGlu5, and mGlu7 in the micromolar range [http://www.ncbi.nlm.nih.gov/pubmed/9680254?dopt=AbstractPlus]. In addition to orthosteric ligands that directly interact with the glutamate recognition site, allosteric modulators that bind within the TM domain have been described. Negative allosteric modulators are listed separately. The positive allosteric modulators most often act as ‘potentiators’ of an orthosteric agonist response, without significantly activating the receptor in the absence of agonist.

**Table:** 

Nomenclature	http://www.guidetopharmacology.org/GRAC/ObjectDisplayForward?objectId=289	http://www.guidetopharmacology.org/GRAC/ObjectDisplayForward?objectId=290	http://www.guidetopharmacology.org/GRAC/ObjectDisplayForward?objectId=291	http://www.guidetopharmacology.org/GRAC/ObjectDisplayForward?objectId=292	http://www.guidetopharmacology.org/GRAC/ObjectDisplayForward?objectId=293
HGNC, UniProt	https://www.genenames.org/data/gene‐symbol‐report/#!/hgnc_id/HGNC:4593, http://www.uniprot.org/uniprot/Q13255	https://www.genenames.org/data/gene‐symbol‐report/#!/hgnc_id/HGNC:4594, http://www.uniprot.org/uniprot/Q14416	https://www.genenames.org/data/gene‐symbol‐report/#!/hgnc_id/HGNC:4595, http://www.uniprot.org/uniprot/Q14832	https://www.genenames.org/data/gene‐symbol‐report/#!/hgnc_id/HGNC:4596, http://www.uniprot.org/uniprot/Q14833	https://www.genenames.org/data/gene‐symbol‐report/#!/hgnc_id/HGNC:4597, http://www.uniprot.org/uniprot/P41594
Endogenous agonists	http://www.guidetopharmacology.org/GRAC/LigandDisplayForward?ligandId=1369 [http://www.ncbi.nlm.nih.gov/pubmed/10443583?dopt=AbstractPlus]	http://www.guidetopharmacology.org/GRAC/LigandDisplayForward?ligandId=1369 [http://www.ncbi.nlm.nih.gov/pubmed/10443583?dopt=AbstractPlus]	http://www.guidetopharmacology.org/GRAC/LigandDisplayForward?ligandId=1369 [http://www.ncbi.nlm.nih.gov/pubmed/10443583?dopt=AbstractPlus], http://www.guidetopharmacology.org/GRAC/LigandDisplayForward?ligandId=1405 [http://www.ncbi.nlm.nih.gov/pubmed/10884552?dopt=AbstractPlus]	http://www.guidetopharmacology.org/GRAC/LigandDisplayForward?ligandId=1369 [http://www.ncbi.nlm.nih.gov/pubmed/10443583?dopt=AbstractPlus]	http://www.guidetopharmacology.org/GRAC/LigandDisplayForward?ligandId=1369 [http://www.ncbi.nlm.nih.gov/pubmed/10443583?dopt=AbstractPlus]
Agonists	–	–	–	http://www.guidetopharmacology.org/GRAC/LigandDisplayForward?ligandId=1410 [http://www.ncbi.nlm.nih.gov/pubmed/9473604?dopt=AbstractPlus], http://www.guidetopharmacology.org/GRAC/LigandDisplayForward?ligandId=1411]	–
Selective agonists	–	–	–	http://www.guidetopharmacology.org/GRAC/LigandDisplayForward?ligandId=6706 [http://www.ncbi.nlm.nih.gov/pubmed/22223752?dopt=AbstractPlus]	(http://www.guidetopharmacology.org/GRAC/LigandDisplayForward?ligandId=3421 (Partial agonist) [http://www.ncbi.nlm.nih.gov/pubmed/10428410?dopt=AbstractPlus] – Rat, http://www.guidetopharmacology.org/GRAC/LigandDisplayForward?ligandId=1417 [http://www.ncbi.nlm.nih.gov/pubmed/11080213?dopt=AbstractPlus]
Antagonists	http://www.guidetopharmacology.org/GRAC/LigandDisplayForward?ligandId=1379 (pIC_50_ 5.1) [400]	–	–	http://www.guidetopharmacology.org/GRAC/LigandDisplayForward?ligandId=1414 (p*K*_i_ 4.6) [http://www.ncbi.nlm.nih.gov/pubmed/10187777?dopt=AbstractPlus] – Rat	–
Selective antagonists	http://www.guidetopharmacology.org/GRAC/LigandDisplayForward?ligandId=3396 (pIC_50_ 5.2) [http://www.ncbi.nlm.nih.gov/pubmed/12015200?dopt=AbstractPlus] – Rat, (http://www.guidetopharmacology.org/GRAC/LigandDisplayForward?ligandId=3421 (pIC_50_ 4.2) [http://www.ncbi.nlm.nih.gov/pubmed/10428410?dopt=AbstractPlus] – Rat, (http://www.guidetopharmacology.org/GRAC/LigandDisplayForward?ligandId=3419 (pIC_50_ 4.2) [http://www.ncbi.nlm.nih.gov/pubmed/11249114?dopt=AbstractPlus] – Rat, http://www.guidetopharmacology.org/GRAC/LigandDisplayForward?ligandId=1376 (p*A*_2_ 4.2) [http://www.ncbi.nlm.nih.gov/pubmed/9152378?dopt=AbstractPlus]	http://www.guidetopharmacology.org/GRAC/LigandDisplayForward?ligandId=3335 (pIC_50_ 5.1) [http://www.ncbi.nlm.nih.gov/pubmed/8667369?dopt=AbstractPlus] – Rat	–	–	http://www.guidetopharmacology.org/GRAC/LigandDisplayForward?ligandId=3388 (pIC_50_ 6.9) [http://www.ncbi.nlm.nih.gov/pubmed/15686941?dopt=AbstractPlus]
Allosteric modulators	–	http://www.guidetopharmacology.org/GRAC/LigandDisplayForward?ligandId=3372 (Positive) (pEC_50_ 7) [http://www.ncbi.nlm.nih.gov/pubmed/15717213?dopt=AbstractPlus], http://www.guidetopharmacology.org/GRAC/LigandDisplayForward?ligandId=1402 (Positive) (pIC_50_ 5.8) [http://www.ncbi.nlm.nih.gov/pubmed/15149652?dopt=AbstractPlus, http://www.ncbi.nlm.nih.gov/pubmed/12852748?dopt=AbstractPlus, http://www.ncbi.nlm.nih.gov/pubmed/12852748?dopt=AbstractPlus, http://www.ncbi.nlm.nih.gov/pubmed/14500736?dopt=AbstractPlus]	http://www.guidetopharmacology.org/GRAC/LigandDisplayForward?ligandId=6221 (Negative) (pIC_50_ 7.7) [http://www.ncbi.nlm.nih.gov/pubmed/17416742?dopt=AbstractPlus] – Rat, http://www.guidetopharmacology.org/GRAC/LigandDisplayForward?ligandId=10237 (Negative) (pIC_50_ 6.4) [http://www.ncbi.nlm.nih.gov/pubmed/26335039?dopt=AbstractPlus]	http://www.guidetopharmacology.org/GRAC/LigandDisplayForward?ligandId=1432 (Positive) (pEC_50_ 6.3–6.8) [http://www.ncbi.nlm.nih.gov/pubmed/12684257?dopt=AbstractPlus], http://www.guidetopharmacology.org/GRAC/LigandDisplayForward?ligandId=1426 (Positive) (pEC_50_ 6.3–6.6) [http://www.ncbi.nlm.nih.gov/pubmed/12684257?dopt=AbstractPlus], http://www.guidetopharmacology.org/GRAC/LigandDisplayForward?ligandId=1416 (Positive) (pEC_50_ 4.5) [http://www.ncbi.nlm.nih.gov/pubmed/14573382?dopt=AbstractPlus]	http://www.guidetopharmacology.org/GRAC/LigandDisplayForward?ligandId=8543 (Negative) (pIC_50_ 8.1) [http://www.ncbi.nlm.nih.gov/pubmed/25173999?dopt=AbstractPlus] – Rat, http://www.guidetopharmacology.org/GRAC/LigandDisplayForward?ligandId=1422 (Positive) (pEC_50_ 7.6–8) [http://www.ncbi.nlm.nih.gov/pubmed/15608073?dopt=AbstractPlus, http://www.ncbi.nlm.nih.gov/pubmed/15537338?dopt=AbstractPlus], http://www.guidetopharmacology.org/GRAC/LigandDisplayForward?ligandId=3336 (Negative) (p*K*_i_ 7.8) [http://www.ncbi.nlm.nih.gov/pubmed/12473093?dopt=AbstractPlus], http://www.guidetopharmacology.org/GRAC/LigandDisplayForward?ligandId=1426 (Negative) (pIC_50_ 7.4–7.7) [http://www.ncbi.nlm.nih.gov/pubmed/11814808?dopt=AbstractPlus, http://www.ncbi.nlm.nih.gov/pubmed/10530811?dopt=AbstractPlus], http://www.guidetopharmacology.org/GRAC/LigandDisplayForward?ligandId=1434 (Negative) (pIC_50_ 7.2) [http://www.ncbi.nlm.nih.gov/pubmed/16040814?dopt=AbstractPlus]
Selective allosteric modulators	http://www.guidetopharmacology.org/GRAC/LigandDisplayForward?ligandId=1381 (Negative) (p*K*_i_ 9.5) [http://www.ncbi.nlm.nih.gov/pubmed/11306677?dopt=AbstractPlus] – Rat, http://www.guidetopharmacology.org/GRAC/LigandDisplayForward?ligandId=1385 (Negative) (pIC_50_ 8.9) [http://www.ncbi.nlm.nih.gov/pubmed/15555631?dopt=AbstractPlus], http://www.guidetopharmacology.org/GRAC/LigandDisplayForward?ligandId=1386 (Positive) (p*K*_i_ 7.5–7.7) [http://www.ncbi.nlm.nih.gov/pubmed/11606768?dopt=AbstractPlus] – Rat, http://www.guidetopharmacology.org/GRAC/LigandDisplayForward?ligandId=3346 (Negative) (pIC_50_ 6.9) [1270], http://www.guidetopharmacology.org/GRAC/LigandDisplayForward?ligandId=1382 (Negative) (pIC_50_ 5.2–5.8) [http://www.ncbi.nlm.nih.gov/pubmed/10051528?dopt=AbstractPlus]	http://www.guidetopharmacology.org/GRAC/LigandDisplayForward?ligandId=3955 (Negative) (pIC_50_ 7) [http://www.ncbi.nlm.nih.gov/pubmed/10465539?dopt=AbstractPlus] – Rat, http://www.guidetopharmacology.org/GRAC/LigandDisplayForward?ligandId=3954 (Positive) (pEC_50_ 7) [http://www.ncbi.nlm.nih.gov/pubmed/16046122?dopt=AbstractPlus]	http://www.guidetopharmacology.org/GRAC/LigandDisplayForward?ligandId=8765 (Negative) (pIC_50_ 6.2) [http://www.ncbi.nlm.nih.gov/pubmed/23718281?dopt=AbstractPlus] – Rat	http://www.guidetopharmacology.org/GRAC/LigandDisplayForward?ligandId=3956 (Positive) (pEC_50_ 6.6) [http://www.ncbi.nlm.nih.gov/pubmed/19469556?dopt=AbstractPlus], http://www.guidetopharmacology.org/GRAC/LigandDisplayForward?ligandId=3323 (Positive) (pEC_50_ 6.1) [http://www.ncbi.nlm.nih.gov/pubmed/18664603?dopt=AbstractPlus]	http://www.guidetopharmacology.org/GRAC/LigandDisplayForward?ligandId=4059 (Positive) (pEC_50_ 8) [http://www.ncbi.nlm.nih.gov/pubmed/16722652?dopt=AbstractPlus]

**Table:** 

Nomenclature	http://www.guidetopharmacology.org/GRAC/ObjectDisplayForward?objectId=294	http://www.guidetopharmacology.org/GRAC/ObjectDisplayForward?objectId=295	http://www.guidetopharmacology.org/GRAC/ObjectDisplayForward?objectId=296
HGNC, UniProt	https://www.genenames.org/data/gene‐symbol‐report/#!/hgnc_id/HGNC:4598, http://www.uniprot.org/uniprot/O15303	https://www.genenames.org/data/gene‐symbol‐report/#!/hgnc_id/HGNC:4599, http://www.uniprot.org/uniprot/Q14831	https://www.genenames.org/data/gene‐symbol‐report/#!/hgnc_id/HGNC:4600, http://www.uniprot.org/uniprot/O00222
Endogenous agonists	http://www.guidetopharmacology.org/GRAC/LigandDisplayForward?ligandId=1369 [http://www.ncbi.nlm.nih.gov/pubmed/10443583?dopt=AbstractPlus]	http://www.guidetopharmacology.org/GRAC/LigandDisplayForward?ligandId=1369 [http://www.ncbi.nlm.nih.gov/pubmed/10443583?dopt=AbstractPlus]	http://www.guidetopharmacology.org/GRAC/LigandDisplayForward?ligandId=1411 [http://www.ncbi.nlm.nih.gov/pubmed/10216218?dopt=AbstractPlus, http://www.ncbi.nlm.nih.gov/pubmed/9473604?dopt=AbstractPlus], http://www.guidetopharmacology.org/GRAC/LigandDisplayForward?ligandId=1369 [http://www.ncbi.nlm.nih.gov/pubmed/10443583?dopt=AbstractPlus]
Agonists	–	http://www.guidetopharmacology.org/GRAC/LigandDisplayForward?ligandId=6706 [http://www.ncbi.nlm.nih.gov/pubmed/22223752?dopt=AbstractPlus], http://www.guidetopharmacology.org/GRAC/LigandDisplayForward?ligandId=1411 [http://www.ncbi.nlm.nih.gov/pubmed/9473604?dopt=AbstractPlus], http://www.guidetopharmacology.org/GRAC/LigandDisplayForward?ligandId=1410]	(http://www.guidetopharmacology.org/GRAC/LigandDisplayForward?ligandId=1407)http://www.guidetopharmacology.org/GRAC/LigandDisplayForward?ligandId=1407 [http://www.ncbi.nlm.nih.gov/pubmed/11166323?dopt=AbstractPlus], http://www.guidetopharmacology.org/GRAC/LigandDisplayForward?ligandId=1410 [http://www.ncbi.nlm.nih.gov/pubmed/10216218?dopt=AbstractPlus]
Selective agonists	http://www.guidetopharmacology.org/GRAC/LigandDisplayForward?ligandId=1436 [2158] – Rat, http://www.guidetopharmacology.org/GRAC/LigandDisplayForward?ligandId=3359 [http://www.ncbi.nlm.nih.gov/pubmed/8759641?dopt=AbstractPlus]	–	–
Antagonists	http://www.guidetopharmacology.org/GRAC/LigandDisplayForward?ligandId=1414 (pIC_50_ 3.5) [http://www.ncbi.nlm.nih.gov/pubmed/12769621?dopt=AbstractPlus] – Rat, http://www.guidetopharmacology.org/GRAC/LigandDisplayForward?ligandId=3326 [http://www.ncbi.nlm.nih.gov/pubmed/9144637?dopt=AbstractPlus]	–	http://www.guidetopharmacology.org/GRAC/LigandDisplayForward?ligandId=1415 (pIC_50_ 4.3) [http://www.ncbi.nlm.nih.gov/pubmed/9473604?dopt=AbstractPlus]
Allosteric modulators	–	http://www.guidetopharmacology.org/GRAC/LigandDisplayForward?ligandId=3341 (Negative) (pIC_50_ 6.1–7.6) [http://www.ncbi.nlm.nih.gov/pubmed/20026717?dopt=AbstractPlus, http://www.ncbi.nlm.nih.gov/pubmed/17609420?dopt=AbstractPlus] – Rat, http://www.guidetopharmacology.org/GRAC/LigandDisplayForward?ligandId=6217 (Negative) (pIC_50_ 7.2) [http://www.ncbi.nlm.nih.gov/pubmed/23257312?dopt=AbstractPlus], http://www.guidetopharmacology.org/GRAC/LigandDisplayForward?ligandId=1441 (Positive) (pEC_50_ 6.5–6.8) [http://www.ncbi.nlm.nih.gov/pubmed/16339898?dopt=AbstractPlus], http://www.guidetopharmacology.org/GRAC/LigandDisplayForward?ligandId=8545 (Negative) (pIC_50_ 5.6) [http://www.ncbi.nlm.nih.gov/pubmed/24596089?dopt=AbstractPlus]	http://www.guidetopharmacology.org/GRAC/LigandDisplayForward?ligandId=10239 (Positive) (p*K*_B_ 6.7) [http://www.ncbi.nlm.nih.gov/pubmed/25225882?dopt=AbstractPlus], http://www.guidetopharmacology.org/GRAC/LigandDisplayForward?ligandId=10238 (Positive) (p*K*_B_ 5) [http://www.ncbi.nlm.nih.gov/pubmed/25225882?dopt=AbstractPlus]

### Comments

The activity of http://www.guidetopharmacology.org/GRAC/LigandDisplayForward?ligandId=1405 as an agonist at mGlu_3_ receptors was questioned on the basis of contamination with glutamate [http://www.ncbi.nlm.nih.gov/pubmed/19389924?dopt=AbstractPlus, http://www.ncbi.nlm.nih.gov/pubmed/19285517?dopt=AbstractPlus], but this has been refuted [http://www.ncbi.nlm.nih.gov/pubmed/21740441?dopt=AbstractPlus].

Radioligand binding using a variety of radioligands has been conducted on recombinant receptors (for example, [http://www.guidetopharmacology.org/GRAC/LigandDisplayForward?ligandId=1391 http://www.guidetopharmacology.org/GRAC/LigandDisplayForward?ligandId=1391 [http://www.ncbi.nlm.nih.gov/pubmed/12695537?dopt=AbstractPlus] and [http://www.guidetopharmacology.org/GRAC/LigandDisplayForward?ligandId=5392 http://www.guidetopharmacology.org/GRAC/LigandDisplayForward?ligandId=5392 [http://www.ncbi.nlm.nih.gov/pubmed/15976016?dopt=AbstractPlus] at mGlu_1_ receptors and [http://www.guidetopharmacology.org/GRAC/LigandDisplayForward?ligandId=1425 http://www.guidetopharmacology.org/GRAC/LigandDisplayForward?ligandId=1425 [http://www.ncbi.nlm.nih.gov/pubmed/11814808?dopt=AbstractPlus] and [http://www.guidetopharmacology.org/GRAC/LigandDisplayForward?ligandId=5394 http://www.guidetopharmacology.org/GRAC/LigandDisplayForward?ligandId=5394 [http://www.ncbi.nlm.nih.gov/pubmed/12438526?dopt=AbstractPlus] at mGlu5 receptors; [http://www.guidetopharmacology.org/GRAC/LigandDisplayForward?ligandId=1399 http://www.guidetopharmacology.org/GRAC/LigandDisplayForward?ligandId=1399 and [http://www.guidetopharmacology.org/GRAC/LigandDisplayForward?ligandId=1396 http://www.guidetopharmacology.org/GRAC/LigandDisplayForward?ligandId=1396 for mGlu_2_ and mGlu_3_ receptors [http://www.ncbi.nlm.nih.gov/pubmed/10530814?dopt=AbstractPlus, http://www.ncbi.nlm.nih.gov/pubmed/10884552?dopt=AbstractPlus]). Although a number of radioligands have been used to examine binding in native tissues, correlation with individual subtypes is limited. Many pharmacological agents have not been fully tested across all known subtypes of mGlu receptors and may have unappreciated biased or neutral activity at other subtypes [http://www.ncbi.nlm.nih.gov/pubmed/29514854?dopt=AbstractPlus]. Potential differences linked to the species (*e.g.* human *versus* rat or mouse) of the receptors and the receptor splice variants are generally not known. The influence of receptor expression level on pharmacology and selectivity has not been controlled for in most studies, particularly those involving functional assays of receptor coupling.

(http://www.guidetopharmacology.org/GRAC/LigandDisplayForward?ligandId=3421 is an antagonist at mGlu_1_, but is an agonist (albeit of reduced efficacy) at mGlu_5_ receptors. http://www.guidetopharmacology.org/GRAC/LigandDisplayForward?ligandId=1377 also exhibits agonist activity at NMDA glutamate receptors [http://www.ncbi.nlm.nih.gov/pubmed/9106476?dopt=AbstractPlus], and is an antagonist at all Group‐III mGluRs with an IC_50_ of 30*μ*M. A potential novel metabotropic glutamate receptor coupled to phosphoinositide turnover has been observed in rat brain; it is activated by http://www.guidetopharmacology.org/GRAC/LigandDisplayForward?ligandId=5449 (ineffective as an agonist at recombinant Group I metabotropic glutamate receptors), but is resistant to http://www.guidetopharmacology.org/GRAC/LigandDisplayForward?ligandId=1378 [http://www.ncbi.nlm.nih.gov/pubmed/9353394?dopt=AbstractPlus]. There are also reports of a distinct metabotropic glutamatereceptor coupled tophospholipase D in ratbrain, which does not readily fit into the current classification [http://www.ncbi.nlm.nih.gov/pubmed/9175608?dopt=AbstractPlus, http://www.ncbi.nlm.nih.gov/pubmed/8799579?dopt=AbstractPlus]

A related class C receptor composed of two distinct subunits, T1R1 + T1R3 is also activated by glutamate and is responsible for umami taste detection.

All selective antagonists at metabotropic glutamate receptors are competitive.

### Further reading on Metabotropic glutamate receptors

Conn PJ *et al*. (1997) Pharmacology and functions of metabotropic glutamate receptors. *Annu. Rev. Pharmacol. Toxicol.* **37**: 205‐237 [https://www.ncbi.nlm.nih.gov/pubmed/9131252?dopt=AbstractPlus]

Ferraguti F *et al*. (2006) Metabotropic glutamate receptors. *Cell Tissue Res.* **326**: 483‐504 [https://www.ncbi.nlm.nih.gov/pubmed/16847639?dopt=AbstractPlus]

Nicoletti F *et al*. (2011) Metabotropic glutamate receptors: from the workbench to the bedside. *Neuropharmacology* **60**: 1017‐41 [https://www.ncbi.nlm.nih.gov/pubmed/21036182?dopt=AbstractPlus]

Niswender CM *et al*. (2010) Metabotropic glutamate receptors: physiology, pharmacology, and disease. *Annu. Rev. Pharmacol. Toxicol.* **50**: 295‐322 [https://www.ncbi.nlm.nih.gov/pubmed/20055706?dopt=AbstractPlus]

Pin JP *et al*. (2016) Organization and functions of mGlu and GABAB receptor complexes. *Nature* **540**: 60‐68 [https://www.ncbi.nlm.nih.gov/pubmed/27905440?dopt=AbstractPlus]

Rondard P *et al*. (2011) The complexity of their activation mechanism opens new possibilities for the modulation of mGlu and GABAB class C G protein‐coupled receptors. *Neuropharmacology* **60**: 82‐92 [https://www.ncbi.nlm.nih.gov/pubmed/20713070?dopt=AbstractPlus]

## http://www.guidetopharmacology.org/GRAC/FamilyDisplayForward?familyId=41

### Overview

Motilin receptors (**provisional nomenclature**) are activated by http://www.guidetopharmacology.org/GRAC/LigandDisplayForward?ligandId=1458 (https://www.genenames.org/data/gene‐symbol‐report/#!/hgnc_id/HGNC:7141, http://www.uniprot.org/uniprot/P12872), a 22 amino‐acid peptide derived from a precursor (https://www.genenames.org/data/gene‐symbol‐report/#!/hgnc_id/HGNC:7141, http://www.uniprot.org/uniprot/P12872), which may also generate a http://www.guidetopharmacology.org/GRAC/LigandDisplayForward?ligandId=5376 (https://www.genenames.org/data/gene‐symbol‐report/#!/hgnc_id/HGNC:7141, http://www.uniprot.org/uniprot/P12872). These receptors promote gastrointestinal motility and are suggested to be responsible for the gastrointestinal prokinetic effects of certain macrolide antibiotics (often called motilides; *e.g.* erythromycin), although for many of these molecules the evidence is sparse.

**Table:** 

Nomenclature	http://www.guidetopharmacology.org/GRAC/ObjectDisplayForward?objectId=297
HGNC, UniProt	https://www.genenames.org/data/gene‐symbol‐report/#!/hgnc_id/HGNC:4495, http://www.uniprot.org/uniprot/O43193
Endogenous agonists	http://www.guidetopharmacology.org/GRAC/LigandDisplayForward?ligandId=1458 (https://www.genenames.org/data/gene‐symbol‐report/#!/hgnc_id/HGNC:7141, http://www.uniprot.org/uniprot/P12872) [http://www.ncbi.nlm.nih.gov/pubmed/11461914?dopt=AbstractPlus, http://www.ncbi.nlm.nih.gov/pubmed/15677347?dopt=AbstractPlus, http://www.ncbi.nlm.nih.gov/pubmed/11781320?dopt=AbstractPlus, http://www.ncbi.nlm.nih.gov/pubmed/16531413?dopt=AbstractPlus]
Agonists	http://www.guidetopharmacology.org/GRAC/LigandDisplayForward?ligandId=1444 [http://www.ncbi.nlm.nih.gov/pubmed/15764739?dopt=AbstractPlus], http://www.guidetopharmacology.org/GRAC/LigandDisplayForward?ligandId=1456 [http://www.ncbi.nlm.nih.gov/pubmed/10381885?dopt=AbstractPlus, http://www.ncbi.nlm.nih.gov/pubmed/10381885?dopt=AbstractPlus], http://www.guidetopharmacology.org/GRAC/LigandDisplayForward?ligandId=6510 [http://www.ncbi.nlm.nih.gov/pubmed/23190027?dopt=AbstractPlus]
Selective agonists	http://www.guidetopharmacology.org/GRAC/LigandDisplayForward?ligandId=4035 [http://www.ncbi.nlm.nih.gov/pubmed/25341626?dopt=AbstractPlus, http://www.ncbi.nlm.nih.gov/pubmed/19374732?dopt=AbstractPlus], http://www.guidetopharmacology.org/GRAC/LigandDisplayForward?ligandId=3511 [1123, http://www.ncbi.nlm.nih.gov/pubmed/17183187?dopt=AbstractPlus] – Rabbit
Selective antagonists	http://www.guidetopharmacology.org/GRAC/LigandDisplayForward?ligandId=4036 (p*A*_2_ 9.2) [http://www.ncbi.nlm.nih.gov/pubmed/18164286?dopt=AbstractPlus], http://www.guidetopharmacology.org/GRAC/LigandDisplayForward?ligandId=1466 (pIC_50_ 8) [http://www.ncbi.nlm.nih.gov/pubmed/11806718?dopt=AbstractPlus] – Rabbit
Labelled ligands	[http://www.guidetopharmacology.org/GRAC/LigandDisplayForward?ligandId=3794 (Agonist) [http://www.ncbi.nlm.nih.gov/pubmed/10381885?dopt=AbstractPlus]

### Comments

In terms of structure, the motilin receptor has closest homology with the ghrelin receptor. Thus, the human motilin receptor shares 52% overall amino acid identity with the ghrelin receptor and 86% in the transmembrane regions [http://www.ncbi.nlm.nih.gov/pubmed/19696113?dopt=AbstractPlus, http://www.ncbi.nlm.nih.gov/pubmed/17183187?dopt=AbstractPlus, http://www.ncbi.nlm.nih.gov/pubmed/15764739?dopt=AbstractPlus]. However, differences between the N‐terminus regions of these receptors means that their cognate peptide ligands do not readily activate each other [http://www.ncbi.nlm.nih.gov/pubmed/14504130?dopt=AbstractPlus, http://www.ncbi.nlm.nih.gov/pubmed/19374732?dopt=AbstractPlus]. In laboratory rodents, the gene encoding the motilin percursor appears to be absent, while the receptor appears to be a pseudogene [http://www.ncbi.nlm.nih.gov/pubmed/19696113?dopt=AbstractPlus, http://www.ncbi.nlm.nih.gov/pubmed/21531468?dopt=AbstractPlus]. Functions of http://www.guidetopharmacology.org/GRAC/LigandDisplayForward?ligandId=1458 (https://www.genenames.org/data/gene‐symbol‐report/#!/hgnc_id/HGNC:7141, http://www.uniprot.org/uniprot/P12872) are not usually detected in rodents, although brain and other responses to motilin and the macrolide http://www.guidetopharmacology.org/GRAC/LigandDisplayForward?ligandId=1444 have been reported and the mechanism of these actions is obscure [http://www.ncbi.nlm.nih.gov/pubmed/9441746?dopt=AbstractPlus, http://www.ncbi.nlm.nih.gov/pubmed/10092986?dopt=AbstractPlus]. Notably, in some non‐laboratory rodents (*e.g.* the North American kangaroo rat (*Dipodomys*) and mouse (*Microdipodops*) a functional form of motilin may exist but the motilin receptor is non‐functional [http://www.ncbi.nlm.nih.gov/pubmed/15027861?dopt=AbstractPlus]. Marked differences in ligand affinities for the motilin receptor in dogs and humans may be explained by significant differences in receptor structure [http://www.ncbi.nlm.nih.gov/pubmed/23189978?dopt=AbstractPlus]. Note that for the complex macrolide structures, selectivity of action has often not been rigorously examined and other actions are possible (*e.g.* P2X inhibition by erythromycin; [http://www.ncbi.nlm.nih.gov/pubmed/10749750?dopt=AbstractPlus]). Small molecule motilin receptor agonists are now described [http://www.ncbi.nlm.nih.gov/pubmed/15027861?dopt=AbstractPlus, http://www.ncbi.nlm.nih.gov/pubmed/19374732?dopt=AbstractPlus, http://www.ncbi.nlm.nih.gov/pubmed/21544957?dopt=AbstractPlus]. The motilin receptor does not appear to have constitutive activity [http://www.ncbi.nlm.nih.gov/pubmed/12907757?dopt=AbstractPlus]. Although not proven, the existence of biased agonism at the receptor has been suggested [http://www.ncbi.nlm.nih.gov/pubmed/16531413?dopt=AbstractPlus, http://www.ncbi.nlm.nih.gov/pubmed/17074305?dopt=AbstractPlus, http://www.ncbi.nlm.nih.gov/pubmed/24438586?dopt=AbstractPlus]. A truncated 5‐transmembrane structure has been identified but this is without activity when transfected into a host cell [http://www.ncbi.nlm.nih.gov/pubmed/10381885?dopt=AbstractPlus]. Receptor dimerisation has not been reported.

### Further reading on Motilin receptor

De Smet B *et al*. (2009) Motilin and ghrelin as prokinetic drug targets. *Pharmacol. Ther.* **123**: 207‐23 [https://www.ncbi.nlm.nih.gov/pubmed/19427331?dopt=AbstractPlus]

Marrinan SL *etal*. (2018) A randomized, double‐blind, placebo‐controlled trial of camicinal in Parkinson's disease. *Mov. Disord.* **33**: 329‐332 [https://www.ncbi.nlm.nih.gov/pubmed/29278279?dopt=AbstractPlus]

Sanger GJ *et al*. (2016) Ghrelin and motilin receptors as drug targets for gastrointestinal disorders. *Nat Rev Gastroenterol Hepatol* **13**: 38‐48 [https://www.ncbi.nlm.nih.gov/pubmed/26392067?dopt=AbstractPlus]

## http://www.guidetopharmacology.org/GRAC/FamilyDisplayForward?familyId=42

### Overview

Neuromedin U receptors (**provisional nomenclature as recommended by NC‐IUPHAR** [http://www.ncbi.nlm.nih.gov/pubmed/15914470?dopt=AbstractPlus]) are activated by the endogenous 25 amino acid peptide neuromedin U (http://www.guidetopharmacology.org/GRAC/LigandDisplayForward?ligandId=1470 (https://www.genenames.org/data/gene‐symbol‐report/#!/hgnc_id/HGNC:7859, http://www.uniprot.org/uniprot/P48645), NmU‐25), a peptide originally isolated from pig spinal cord [http://www.ncbi.nlm.nih.gov/pubmed/3839674?dopt=AbstractPlus]. In humans, NmU‐25 appears to be the sole product of a precursor gene (https://www.genenames.org/data/gene‐symbol‐report/#!/hgnc_id/HGNC:7859, http://www.uniprot.org/uniprot/P48645) showing a broad tissue distribution, but which is expressed at highest levels in the upper gastrointestinal tract, CNS, bone marrow and fetal liver. Much shorter versions of NmU are found in some species, but not in human, and are derived at least in some instances from the proteolytic cleavage of the longer NmU. Despite species differences in NmU structure, the C‐terminal region (particularly the C‐terminal pentapeptide) is highly conserved and contains biological activity. Neuromedin S (http://www.guidetopharmacology.org/GRAC/LigandDisplayForward?ligandId=1468 (https://www.genenames.org/data/gene‐symbol‐report/#!/hgnc_id/HGNC:32203, http://www.uniprot.org/uniprot/Q5H8A3)) has also been identified as an endogenous agonist [http://www.ncbi.nlm.nih.gov/pubmed/15635449?dopt=AbstractPlus]. NmS33 is, as its name suggests, a 33 amino‐acid product of a precursor protein derived from a single gene and contains an amidated Cterminal heptapeptide identical to NmU. NmS‐33 appears to activate NMU receptors with equivalent potency to NmU‐25.

**Table:** 

Nomenclature	http://www.guidetopharmacology.org/GRAC/ObjectDisplayForward?objectId=298	http://www.guidetopharmacology.org/GRAC/ObjectDisplayForward?objectId=299
HGNC, UniProt	https://www.genenames.org/data/gene‐symbol‐report/#!/hgnc_id/HGNC:4518, http://www.uniprot.org/uniprot/Q9HB89	https://www.genenames.org/data/gene‐symbol‐report/#!/hgnc_id/HGNC:16454, http://www.uniprot.org/uniprot/Q9GZQ4
Antagonists	–	http://www.guidetopharmacology.org/GRAC/LigandDisplayForward?ligandId=3524 (p*K*_B_ 7) [http://www.ncbi.nlm.nih.gov/pubmed/19369576?dopt=AbstractPlus]

### Comments

NMU1 and NMU2 couple predominantly to G_q/11_ although there is evidence of good coupling to G_i/o_ [http://www.ncbi.nlm.nih.gov/pubmed/15331768?dopt=AbstractPlus, http://www.ncbi.nlm.nih.gov/pubmed/10887190?dopt=AbstractPlus, http://www.ncbi.nlm.nih.gov/pubmed/17652154?dopt=AbstractPlus]. NMU1 and NMU2 can be labelled with [^125^I]‐NmU and [^125^I]‐NmS (of various species, *e.g.* [http://www.ncbi.nlm.nih.gov/pubmed/18358099?dopt=AbstractPlus]), http://www.guidetopharmacology.org/GRAC/LigandDisplayForward?ligandId=3858 or http://www.guidetopharmacology.org/GRAC/LigandDisplayForward?ligandId=3863 [http://www.ncbi.nlm.nih.gov/pubmed/15331768?dopt=AbstractPlus]. A range of radiolabelled ( ^125^I‐), fluorescently labelled (*e.g.* Cy3, Cy5, rhodamine and FAM) and http://www.guidetopharmacology.org/GRAC/LigandDisplayForward?ligandId=4787 labelled versions of http://www.guidetopharmacology.org/GRAC/LigandDisplayForward?ligandId=1470 (https://www.genenames.org/data/gene‐symbol‐report/#!/hgnc_id/HGNC:7859, http://www.uniprot.org/uniprot/P48645) and http://www.guidetopharmacology.org/GRAC/LigandDisplayForward?ligandId=1468 (https://www.genenames.org/data/gene‐symbol‐report/#!/hgnc_id/HGNC:32203, http://www.uniprot.org/uniprot/Q5H8A3) are now commercially available.

### Further reading on Neuromedin U receptors

Brighton PJ *et al*. (2004) Neuromedin U and its receptors: structure, function, and physiological roles. *Pharmacol. Rev.* **56**: 231‐48 [https://www.ncbi.nlm.nih.gov/pubmed/15169928?dopt=AbstractPlus]

Budhiraja S *et al*. (2009) Neuromedin U: physiology, pharmacology and therapeutic potential. *Fundam Clin Pharmacol* **23**: 149‐57 [https://www.ncbi.nlm.nih.gov/pubmed/19645813?dopt=AbstractPlus]

Mitchell JD *et al*. (2009) Emerging pharmacology and physiology of neuromedin U and the structurally related peptide neuromedin S. *Br. J. Pharmacol.* **158**: 87‐103 [https://www.ncbi.nlm.nih.gov/pubmed/19519756?dopt=AbstractPlus]

Novak CM. (2009) Neuromedin S and U. *Endocrinology* **150**: 2985‐7 [https://www.ncbi.nlm.nih.gov/pubmed/19549882?dopt=AbstractPlus]

## http://www.guidetopharmacology.org/GRAC/FamilyDisplayForward?familyId=43

### Overview

The Neuropeptide FF receptor family contains two subtypes, NPFF1 and NPFF2 (**provisional nomenclature** [http://www.ncbi.nlm.nih.gov/pubmed/15914470?dopt=AbstractPlus]), which exhibit high affinities for neuropeptide FF (https://www.genenames.org/data/gene‐symbol‐report/#!/hgnc_id/HGNC:7901, http://www.uniprot.org/uniprot/O15130) and RFamide related peptides (RFRP: precursor genesymbol https://www.genenames.org/data/gene‐symbol‐report/#!/hgnc_id/HGNC:13782, http://www.uniprot.org/uniprot/Q9HCQ7). NPFF1 is broadly distributed in the central nervous system with the highest levels found in the limbic system and the hypothalamus. NPFF2 is present in high density in the superficial layers of the mammalian spinal cord where it is involved in nociception and modulation of opioid functions.

**Table:** 

Nomenclature	http://www.guidetopharmacology.org/GRAC/ObjectDisplayForward?objectId=300	http://www.guidetopharmacology.org/GRAC/ObjectDisplayForward?objectId=301
HGNC, UniProt	https://www.genenames.org/data/gene‐symbol‐report/#!/hgnc_id/HGNC:17425, http://www.uniprot.org/uniprot/Q9GZQ6	https://www.genenames.org/data/gene‐symbol‐report/#!/hgnc_id/HGNC:4525, http://www.uniprot.org/uniprot/Q9Y5X5
Potency order of endogenous ligands	http://www.guidetopharmacology.org/GRAC/LigandDisplayForward?ligandId=5340 (https://www.genenames.org/data/gene‐symbol‐report/#!/hgnc_id/HGNC:13782, http://www.uniprot.org/uniprot/Q9HCQ7) > http://www.guidetopharmacology.org/GRAC/LigandDisplayForward?ligandId=4016 (https://www.genenames.org/data/gene‐symbol‐report/#!/hgnc_id/HGNC:13782, http://www.uniprot.org/uniprot/Q9HCQ7) > FMRFhttp://www.guidetopharmacology.org/GRAC/LigandDisplayForward?ligandId=1479 (https://www.genenames.org/data/gene‐symbol‐report/#!/hgnc_id/HGNC:7901, http://www.uniprot.org/uniprot/O15130) > http://www.guidetopharmacology.org/GRAC/LigandDisplayForward?ligandId=3735 (https://www.genenames.org/data/gene‐symbol‐report/#!/hgnc_id/HGNC:7901, http://www.uniprot.org/uniprot/O15130) > http://www.guidetopharmacology.org/GRAC/LigandDisplayForward?ligandId=3736 (https://www.genenames.org/data/gene‐symbol‐report/#!/hgnc_id/HGNC:7901, http://www.uniprot.org/uniprot/O15130), http://www.guidetopharmacology.org/GRAC/LigandDisplayForward?ligandId=3665 (https://www.genenames.org/data/gene‐symbol‐report/#!/hgnc_id/HGNC:29982, http://www.uniprot.org/uniprot/P83859), http://www.guidetopharmacology.org/GRAC/LigandDisplayForward?ligandId=1873 (https://www.genenames.org/data/gene‐symbol‐report/#!/hgnc_id/HGNC:17945, http://www.uniprot.org/uniprot/P81277) [http://www.ncbi.nlm.nih.gov/pubmed/17011599?dopt=AbstractPlus]	http://www.guidetopharmacology.org/GRAC/LigandDisplayForward?ligandId=3735 (https://www.genenames.org/data/gene‐symbol‐report/#!/hgnc_id/HGNC:7901, http://www.uniprot.org/uniprot/O15130), http://www.guidetopharmacology.org/GRAC/LigandDisplayForward?ligandId=1479 (https://www.genenames.org/data/gene‐symbol‐report/#!/hgnc_id/HGNC:7901, http://www.uniprot.org/uniprot/O15130) > http://www.guidetopharmacology.org/GRAC/LigandDisplayForward?ligandId=1873 (https://www.genenames.org/data/gene‐symbol‐report/#!/hgnc_id/HGNC:17945, http://www.uniprot.org/uniprot/P81277) > FMRF, http://www.guidetopharmacology.org/GRAC/LigandDisplayForward?ligandId=3665 (https://www.genenames.org/data/gene‐symbol‐report/#!/hgnc_id/HGNC:29982, http://www.uniprot.org/uniprot/P83859) > http://www.guidetopharmacology.org/GRAC/LigandDisplayForward?ligandId=3736 (https://www.genenames.org/data/gene‐symbol‐report/#!/hgnc_id/HGNC:7901, http://www.uniprot.org/uniprot/O15130) [http://www.ncbi.nlm.nih.gov/pubmed/17011599?dopt=AbstractPlus]
Endogenous agonists	http://www.guidetopharmacology.org/GRAC/LigandDisplayForward?ligandId=1479 (https://www.genenames.org/data/gene‐symbol‐report/#!/hgnc_id/HGNC:7901, http://www.uniprot.org/uniprot/O15130) [http://www.ncbi.nlm.nih.gov/pubmed/17011599?dopt=AbstractPlus, http://www.ncbi.nlm.nih.gov/pubmed/12421602?dopt=AbstractPlus, http://www.ncbi.nlm.nih.gov/pubmed/12242085?dopt=AbstractPlus], http://www.guidetopharmacology.org/GRAC/LigandDisplayForward?ligandId=4016 (https://www.genenames.org/data/gene‐symbol‐report/#!/hgnc_id/HGNC:13782, http://www.uniprot.org/uniprot/Q9HCQ7 [http://www.ncbi.nlm.nih.gov/pubmed/12421602?dopt=AbstractPlus, http://www.ncbi.nlm.nih.gov/pubmed/17337079?dopt=AbstractPlus, http://www.ncbi.nlm.nih.gov/pubmed/12242085?dopt=AbstractPlus]	http://www.guidetopharmacology.org/GRAC/LigandDisplayForward?ligandId=1479 (https://www.genenames.org/data/gene‐symbol‐report/#!/hgnc_id/HGNC:7901, http://www.uniprot.org/uniprot/O15130) [http://www.ncbi.nlm.nih.gov/pubmed/12421602?dopt=AbstractPlus, http://www.ncbi.nlm.nih.gov/pubmed/11325787?dopt=AbstractPlus]
Selective agonists	–	http://www.guidetopharmacology.org/GRAC/LigandDisplayForward?ligandId=1489 [http://www.ncbi.nlm.nih.gov/pubmed/16129413?dopt=AbstractPlus], http://www.guidetopharmacology.org/GRAC/LigandDisplayForward?ligandId=4020 [http://www.ncbi.nlm.nih.gov/pubmed/20354177?dopt=AbstractPlus]
Antagonists	http://www.guidetopharmacology.org/GRAC/LigandDisplayForward?ligandId=1486 (p*K*_i_ 7.2) [http://www.ncbi.nlm.nih.gov/pubmed/16407169?dopt=AbstractPlus]	–
Selective antagonists	http://www.guidetopharmacology.org/GRAC/LigandDisplayForward?ligandId=4017 (p*K*_i_ 7.7–8.1) [http://www.ncbi.nlm.nih.gov/pubmed/20354177?dopt=AbstractPlus], http://www.guidetopharmacology.org/GRAC/LigandDisplayForward?ligandId=4018 (p*K*_i_ 7.4–8.1) [http://www.ncbi.nlm.nih.gov/pubmed/20354177?dopt=AbstractPlus]	–
Labelled ligands	[http://www.guidetopharmacology.org/GRAC/LigandDisplayForward?ligandId=1484 (Agonist) [http://www.ncbi.nlm.nih.gov/pubmed/12421602?dopt=AbstractPlus], [http://www.guidetopharmacology.org/GRAC/LigandDisplayForward?ligandId=4019 (Agonist) [http://www.ncbi.nlm.nih.gov/pubmed/19682524?dopt=AbstractPlus], [http://www.guidetopharmacology.org/GRAC/LigandDisplayForward?ligandId=3797 (Agonist) [http://www.ncbi.nlm.nih.gov/pubmed/17011599?dopt=AbstractPlus]	[http://www.guidetopharmacology.org/GRAC/LigandDisplayForward?ligandId=1488 (Agonist) [http://www.ncbi.nlm.nih.gov/pubmed/12242085?dopt=AbstractPlus], [http://www.guidetopharmacology.org/GRAC/LigandDisplayForward?ligandId=4021 (Agonist) [http://www.ncbi.nlm.nih.gov/pubmed/19682524?dopt=AbstractPlus], [http://www.guidetopharmacology.org/GRAC/LigandDisplayForward?ligandId=3797 (Agonist) [http://www.ncbi.nlm.nih.gov/pubmed/17011599?dopt=AbstractPlus]

### Comments

An orphan receptor https://www.genenames.org/data/gene‐symbol‐report/#!/hgnc_id/HGNC:4523 (http://www.uniprot.org/uniprot/Q9NYM4) shows sequence similarities with NPFF1, NPFF2, PrRP and QRFP receptors. The antagonist http://www.guidetopharmacology.org/GRAC/LigandDisplayForward?ligandId=1486 is selective for NPFF receptors, but does not distinguish between the NPFF1 and NPFF2 subtypes (p*K*i 7.1 and 7.2, respectively, [http://www.ncbi.nlm.nih.gov/pubmed/16407169?dopt=AbstractPlus]).

### Further reading on Neuropeptide FF/neuropeptide AF receptors

Moulédous L *et al*. (2010) Opioid‐modulating properties of the neuropeptide FF system. *Biofactors* **36**: 423‐9 [https://www.ncbi.nlm.nih.gov/pubmed/20803521?dopt=AbstractPlus]

Vyas N *et al*. (2006) Structure‐activity relationships of neuropeptide FF and related peptidic and non‐peptidic derivatives. *Peptides* **27**: 990‐6 [https://www.ncbi.nlm.nih.gov/pubmed/16490282?dopt=AbstractPlus]

Yang HY *et al*. (2008) Modulatory role of neuropeptide FF system in nociception and opiate analgesia. *Neuropeptides* **42**: 1‐18 [https://www.ncbi.nlm.nih.gov/pubmed/17854890?dopt=AbstractPlus]

## http://www.guidetopharmacology.org/GRAC/FamilyDisplayForward?familyId=44

### Overview

The neuropeptide S receptor (NPS, **provisional nomenclature** [http://www.ncbi.nlm.nih.gov/pubmed/15914470?dopt=AbstractPlus]) responds to the 20 amino‐acid peptide neuropeptide S derived from a precursor (https://www.genenames.org/data/gene‐symbol‐report/#!/hgnc_id/HGNC:33940, http://www.uniprot.org/uniprot/P0C0P6).

**Table:** 

Nomenclature	http://www.guidetopharmacology.org/GRAC/ObjectDisplayForward?objectId=302
HGNC, UniProt	https://www.genenames.org/data/gene‐symbol‐report/#!/hgnc_id/HGNC:23631, http://www.uniprot.org/uniprot/Q6W5P4
Endogenous agonists	http://www.guidetopharmacology.org/GRAC/LigandDisplayForward?ligandId=1490 (https://www.genenames.org/data/gene‐symbol‐report/#!/hgnc_id/HGNC:33940, http://www.uniprot.org/uniprot/P0C0P6) [http://www.ncbi.nlm.nih.gov/pubmed/15312648?dopt=AbstractPlus]
Selective agonists	http://www.guidetopharmacology.org/GRAC/LigandDisplayForward?ligandId=9167 [http://www.ncbi.nlm.nih.gov/pubmed/25692025?dopt=AbstractPlus] – Mouse
Selective antagonists	http://www.guidetopharmacology.org/GRAC/LigandDisplayForward?ligandId=9168 (p*A*_2_ 9) [http://www.ncbi.nlm.nih.gov/pubmed/23761908?dopt=AbstractPlus], http://www.guidetopharmacology.org/GRAC/LigandDisplayForward?ligandId=5813 (p*A*_2_ 8.1) [http://www.ncbi.nlm.nih.gov/pubmed/20172007?dopt=AbstractPlus] – Mouse, http://www.guidetopharmacology.org/GRAC/LigandDisplayForward?ligandId=7929 [http://www.ncbi.nlm.nih.gov/pubmed/18555684?dopt=AbstractPlus]
Labelled ligands	[http://www.guidetopharmacology.org/GRAC/LigandDisplayForward?ligandId=1493 (Agonist) [http://www.ncbi.nlm.nih.gov/pubmed/15312648?dopt=AbstractPlus]

### Comments

Multiple single‐nucleotide polymorphisms (SNP) and several splice variants have been identified in the human NPS receptor. The most interesting of these is an Asn‐Ile exchange at position 107 (Asn^107^Ile). The human NPS receptor Asn^107^Ile displayed similar binding affinity buthigher NPSpotency (by approx. 10‐fold) than human NPS receptor Asn107 [http://www.ncbi.nlm.nih.gov/pubmed/16144971?dopt=AbstractPlus]. Several epidemiological studies reported an association between Asn^107^Ile receptor variant and susceptibility to panic disorders [http://www.ncbi.nlm.nih.gov/pubmed/20603625?dopt=AbstractPlus, http://www.ncbi.nlm.nih.gov/pubmed/20705147?dopt=AbstractPlus, http://www.ncbi.nlm.nih.gov/pubmed/17669576?dopt=AbstractPlus, http://www.ncbi.nlm.nih.gov/pubmed/20628342?dopt=AbstractPlus]. The SNP Asn^107^Ile has also been linked to sleep behavior [http://www.ncbi.nlm.nih.gov/pubmed/17903308?dopt=AbstractPlus], inflammatory bowel disease [http://www.ncbi.nlm.nih.gov/pubmed/17854592?dopt=AbstractPlus], schizophrenia [http://www.ncbi.nlm.nih.gov/pubmed/22078257?dopt=AbstractPlus], increased impulsivity and ADHD symptoms [http://www.ncbi.nlm.nih.gov/pubmed/23325374?dopt=AbstractPlus]. Interestingly, a carboxy‐terminal splice variant of human NPS receptor was found to be overexpressed in asthmatic patients [http://www.ncbi.nlm.nih.gov/pubmed/15073379?dopt=AbstractPlus].

### Further reading on Neuropeptide S receptor

Grund T *et al*. (2019) Brain neuropeptide S: via GPCR activation to a powerful neuromodulator of socio‐emotional behaviors. *Cell Tissue Res.* **375**: 123‐132 [https://www.ncbi.nlm.nih.gov/pubmed/30112573?dopt=AbstractPlus]

Guerrini R *et al*. (2010) Neurobiology, pharmacology, and medicinal chemistry of neuropeptide S and its receptor. *Med Res Rev* **30**: 751‐77 [https://www.ncbi.nlm.nih.gov/pubmed/19824051?dopt=AbstractPlus]

Ruzza C *et al*. (2017) Neuropeptide S receptor ligands: a patent review (2005‐2016). *Expert Opin Ther Pat* **27**: 347‐362 [https://www.ncbi.nlm.nih.gov/pubmed/27788040?dopt=AbstractPlus]

Xu YL *et al*. (2004) Neuropeptide S: a neuropeptide promoting arousal and anxiolytic‐like effects. *Neuron* **43**: 487‐497 [https://www.ncbi.nlm.nih.gov/pubmed/15312648?dopt=AbstractPlus]

## http://www.guidetopharmacology.org/GRAC/FamilyDisplayForward?familyId=45

### Overview

The neuropeptide BW receptor 1 (NPBW1, **provisional nomenclature** [http://www.ncbi.nlm.nih.gov/pubmed/15914470?dopt=AbstractPlus]) is activated by two 23‐amino‐acid peptides, neuropeptide W (http://www.guidetopharmacology.org/GRAC/LigandDisplayForward?ligandId=1495 (https://www.genenames.org/data/gene‐symbol‐report/#!/hgnc_id/HGNC:30509, http://www.uniprot.org/uniprot/Q8N729)) and neuropeptide B (http://www.guidetopharmacology.org/GRAC/LigandDisplayForward?ligandId=1501 (https://www.genenames.org/data/gene‐symbol‐report/#!/hgnc_id/HGNC:30099, http://www.uniprot.org/uniprot/Q8NG41)) [http://www.ncbi.nlm.nih.gov/pubmed/12118011?dopt=AbstractPlus, http://www.ncbi.nlm.nih.gov/pubmed/12130646?dopt=AbstractPlus]. C‐terminally extended forms of the peptides (http://www.guidetopharmacology.org/GRAC/LigandDisplayForward?ligandId=1496 (https://www.genenames.org/data/gene‐symbol‐report/#!/hgnc_id/HGNC:30509, http://www.uniprot.org/uniprot/Q8N729) and http://www.guidetopharmacology.org/GRAC/LigandDisplayForward?ligandId=1502 (https://www.genenames.org/data/gene‐symbol‐report/#!/hgnc_id/HGNC:30099, http://www.uniprot.org/uniprot/Q8NG41)) also activate NPBW1[http://www.ncbi.nlm.nih.gov/pubmed/12401809?dopt=AbstractPlus]. Unique to both forms of neuropeptide B is the N‐terminal bromination of the first tryptophan residue, and it is from this post‐translational modification that the nomenclature NPB is derived. These peptides were first identified from bovine hypothalamus and therefore are classed as neuropeptides. Endogenous variants of the peptides without the N‐terminal bromination, http://www.guidetopharmacology.org/GRAC/LigandDisplayForward?ligandId=1499 (https://www.genenames.org/data/gene‐symbol‐report/#!/hgnc_id/HGNC:30099, http://www.uniprot.org/uniprot/Q8NG41) and http://www.guidetopharmacology.org/GRAC/LigandDisplayForward?ligandId=1500 (https://www.genenames.org/data/gene‐symbol‐report/#!/hgnc_id/HGNC:30099, http://www.uniprot.org/uniprot/Q8NG41), were not found to be major components of bovine hypothalamic tissue extracts. The NPBW2 receptor is activated by the short and C‐terminal extended forms of neuropeptide W and neuropeptide B [http://www.ncbi.nlm.nih.gov/pubmed/12401809?dopt=AbstractPlus].

**Table:** 

Nomenclature	http://www.guidetopharmacology.org/GRAC/ObjectDisplayForward?objectId=303	http://www.guidetopharmacology.org/GRAC/ObjectDisplayForward?objectId=304
HGNC, UniProt	https://www.genenames.org/data/gene‐symbol‐report/#!/hgnc_id/HGNC:4522, http://www.uniprot.org/uniprot/P48145	https://www.genenames.org/data/gene‐symbol‐report/#!/hgnc_id/HGNC:4530, http://www.uniprot.org/uniprot/P48146
Potency order of endogenous ligands	http://www.guidetopharmacology.org/GRAC/LigandDisplayForward?ligandId=1502 (https://www.genenames.org/data/gene‐symbol‐report/#!/hgnc_id/HGNC:30099, http://www.uniprot.org/uniprot/Q8NG41) > http://www.guidetopharmacology.org/GRAC/LigandDisplayForward?ligandId=1501 (https://www.genenames.org/data/gene‐symbol‐report/#!/hgnc_id/HGNC:30099, http://www.uniprot.org/uniprot/Q8NG41) > http://www.guidetopharmacology.org/GRAC/LigandDisplayForward?ligandId=1495 (https://www.genenames.org/data/gene‐symbol‐report/#!/hgnc_id/HGNC:30509, http://www.uniprot.org/uniprot/Q8N729) > http://www.guidetopharmacology.org/GRAC/LigandDisplayForward?ligandId=1496 (https://www.genenames.org/data/gene‐symbol‐report/#!/hgnc_id/HGNC:30509, http://www.uniprot.org/uniprot/Q8N729) [http://www.ncbi.nlm.nih.gov/pubmed/12401809?dopt=AbstractPlus]	http://www.guidetopharmacology.org/GRAC/LigandDisplayForward?ligandId=1495 (https://www.genenames.org/data/gene‐symbol‐report/#!/hgnc_id/HGNC:30509, http://www.uniprot.org/uniprot/Q8N729) > http://www.guidetopharmacology.org/GRAC/LigandDisplayForward?ligandId=1496 (https://www.genenames.org/data/gene‐symbol‐report/#!/hgnc_id/HGNC:30509, http://www.uniprot.org/uniprot/Q8N729) > http://www.guidetopharmacology.org/GRAC/LigandDisplayForward?ligandId=1502 (https://www.genenames.org/data/gene‐symbol‐report/#!/hgnc_id/HGNC:30099, http://www.uniprot.org/uniprot/Q8NG41) > http://www.guidetopharmacology.org/GRAC/LigandDisplayForward?ligandId=1501 (https://www.genenames.org/data/gene‐symbol‐report/#!/hgnc_id/HGNC:30099, http://www.uniprot.org/uniprot/Q8NG41) [http://www.ncbi.nlm.nih.gov/pubmed/12401809?dopt=AbstractPlus]
Selective agonists	http://www.guidetopharmacology.org/GRAC/LigandDisplayForward?ligandId=3855 [http://www.ncbi.nlm.nih.gov/pubmed/17486669?dopt=AbstractPlus], http://www.guidetopharmacology.org/GRAC/LigandDisplayForward?ligandId=3856 [http://www.ncbi.nlm.nih.gov/pubmed/17486669?dopt=AbstractPlus]	–
Labelled ligands	[http://www.guidetopharmacology.org/GRAC/LigandDisplayForward?ligandId=1497 (Agonist) [http://www.ncbi.nlm.nih.gov/pubmed/15261118?dopt=AbstractPlus]	[http://www.guidetopharmacology.org/GRAC/LigandDisplayForward?ligandId=1497 (Agonist) [http://www.ncbi.nlm.nih.gov/pubmed/12130646?dopt=AbstractPlus]

### Comments

Potency measurements were conducted with heterologously‐expressed receptors with a range of 0.14‐0.57 nM (NPBW1) and 0.98‐21 nM (NPBW2).

NPBW1^‐/‐^ mice show changes in social behavior, suggesting that the NPBW1pathway may have animportant role in theemotional responses of social interaction [http://www.ncbi.nlm.nih.gov/pubmed/21390312?dopt=AbstractPlus].

For a review of the contribution of neuropeptide B/W to social dominance, see Watanabe and Yamamoto, 2015 [http://www.ncbi.nlm.nih.gov/pubmed/26136644?dopt=AbstractPlus]. It has been reported that neuropeptide W may have a key role in the gating of stressful stimuli when mice are exposed to novel environments [http://www.ncbi.nlm.nih.gov/pubmed/27140610?dopt=AbstractPlus].

Two antagonists have been discovered and reported to have affinity for NPBW1, ML181 and ML250, the latter exhibiting improved selectivity (100 fold) for NPBW1 compared to MCH1 receptors [http://www.ncbi.nlm.nih.gov/pubmed/23762933?dopt=AbstractPlus, http://www.ncbi.nlm.nih.gov/pubmed/22834040?dopt=AbstractPlus]. Computational insights into the binding of antagonists to this receptor have also been described [http://www.ncbi.nlm.nih.gov/pubmed/24938207?dopt=AbstractPlus].

### Further reading on Neuropeptide W/neuropeptide B receptors

Sakurai T. (2013) NPBWR1 and NPBWR2: Implications in Energy Homeostasis, Pain, and Emotion. *Front Endocrinol (Lausanne)* **4**: 23 [https://www.ncbi.nlm.nih.gov/pubmed/23515889?dopt=AbstractPlus]

Singh G *et al*. (2006) Neuropeptide B and W: neurotransmitters in an emerging G‐protein‐coupled receptor system. *Br. J. Pharmacol.* **148**: 1033‐41 [https://www.ncbi.nlm.nih.gov/pubmed/16847439?dopt=AbstractPlus]

## http://www.guidetopharmacology.org/GRAC/FamilyDisplayForward?familyId=46

### Overview

Neuropeptide Y (NPY) receptors (**nomenclature as agreed by the NC‐IUPHAR Subcommittee on Neuropeptide Y Receptors** [http://www.ncbi.nlm.nih.gov/pubmed/9549761?dopt=AbstractPlus]) are activated by the endogenous peptides http://www.guidetopharmacology.org/GRAC/LigandDisplayForward?ligandId=1504 (https://www.genenames.org/data/gene‐symbol‐report/#!/hgnc_id/HGNC:7955, http://www.uniprot.org/uniprot/P01303), neuropeptide Y‐(3‐36), http://www.guidetopharmacology.org/GRAC/LigandDisplayForward?ligandId=1514 (https://www.genenames.org/data/gene‐symbol‐report/#!/hgnc_id/HGNC:9748, http://www.uniprot.org/uniprot/P10082), PYY‐(3‐36) and http://www.guidetopharmacology.org/GRAC/LigandDisplayForward?ligandId=1512 (https://www.genenames.org/data/gene‐symbol‐report/#!/hgnc_id/HGNC:9327, http://www.uniprot.org/uniprot/P01298) (PP). The receptor originally identified as the Y3 receptor has been identified as the http://www.guidetopharmacology.org/GRAC/ObjectDisplayForward?objectId=71 (originally named LESTR, [http://www.ncbi.nlm.nih.gov/pubmed/8276799?dopt=AbstractPlus]). The y6 receptor is a functional gene product in mouse, absent in rat, but contains a frame‐shift mutation in primates producing a truncated non‐functional gene [http://www.ncbi.nlm.nih.gov/pubmed/8641440?dopt=AbstractPlus]. Many of the agonists exhibit differing degrees of selectivity dependent on the species examined. For example, the potency of PP is greater at the rat Y4 receptor than at the human receptor [http://www.ncbi.nlm.nih.gov/pubmed/9802391?dopt=AbstractPlus]. In addition, many agonists lack selectivity for individual subtypes, but can exhibit comparable potency against pairs of NPY receptor subtypes, or have not been examined for activity at all subtypes. [^125^I]‐PYY or [^125^I]‐NPY can be used to label Y_1_, Y_2_, Y5 and y_6_ subtypes non‐selectively, while [http://www.guidetopharmacology.org/GRAC/LigandDisplayForward?ligandId=3921 may be used to label Y5 receptors preferentially (note that cPP denotes chicken peptide sequence and hPP is the human sequence).

**Table:** 

Nomenclature	http://www.guidetopharmacology.org/GRAC/ObjectDisplayForward?objectId=305	http://www.guidetopharmacology.org/GRAC/ObjectDisplayForward?objectId=306	http://www.guidetopharmacology.org/GRAC/ObjectDisplayForward?objectId=307	http://www.guidetopharmacology.org/GRAC/ObjectDisplayForward?objectId=308	http://www.guidetopharmacology.org/GRAC/ObjectDisplayForward?objectId=683
HGNC, UniProt	https://www.genenames.org/data/gene‐symbol‐report/#!/hgnc_id/HGNC:7956, http://www.uniprot.org/uniprot/P25929	https://www.genenames.org/data/gene‐symbol‐report/#!/hgnc_id/HGNC:7957, http://www.uniprot.org/uniprot/P49146	https://www.genenames.org/data/gene‐symbol‐report/#!/hgnc_id/HGNC:9329, http://www.uniprot.org/uniprot/P50391	https://www.genenames.org/data/gene‐symbol‐report/#!/hgnc_id/HGNC:7958, http://www.uniprot.org/uniprot/Q15761	https://www.genenames.org/data/gene‐symbol‐report/#!/hgnc_id/HGNC:7959, http://www.uniprot.org/uniprot/Q99463
Potency order of endogenous ligands	neuropeptide Y = peptide YY ≫ pancreatic polypeptide	peptide YY = peptide YY(3‐36) = neuropeptide Y = neuropeptide Y(3‐36) ≫ pancreatic polypeptide	pancreatic polypeptide ≫ neuropeptide Y = peptide YY	neuropeptide Y > peptide YY > pancreatic polypeptide	neuropeptide Y = peptide YY > pancreatic polypeptide
Endogenous agonists	http://www.guidetopharmacology.org/GRAC/LigandDisplayForward?ligandId=1504 (https://www.genenames.org/data/gene‐symbol‐report/#!/hgnc_id/HGNC:7955, http://www.uniprot.org/uniprot/P01303), http://www.guidetopharmacology.org/GRAC/LigandDisplayForward?ligandId=1514 (https://www.genenames.org/data/gene‐symbol‐report/#!/hgnc_id/HGNC:9748, http://www.uniprot.org/uniprot/P10082)	http://www.guidetopharmacology.org/GRAC/LigandDisplayForward?ligandId=1517) (https://www.genenames.org/data/gene‐symbol‐report/#!/hgnc_id/HGNC:9748, http://www.uniprot.org/uniprot/P10082) [http://www.ncbi.nlm.nih.gov/pubmed/8632753?dopt=AbstractPlus, http://www.ncbi.nlm.nih.gov/pubmed/7592910?dopt=AbstractPlus], http://www.guidetopharmacology.org/GRAC/LigandDisplayForward?ligandId=1504 (https://www.genenames.org/data/gene‐symbol‐report/#!/hgnc_id/HGNC:7955, http://www.uniprot.org/uniprot/P01303), http://www.guidetopharmacology.org/GRAC/LigandDisplayForward?ligandId=3627) (https://www.genenames.org/data/gene‐symbol‐report/#!/hgnc_id/HGNC:7955, http://www.uniprot.org/uniprot/P01303), http://www.guidetopharmacology.org/GRAC/LigandDisplayForward?ligandId=1514 (https://www.genenames.org/data/gene‐symbol‐report/#!/hgnc_id/HGNC:9748, http://www.uniprot.org/uniprot/P10082)	http://www.guidetopharmacology.org/GRAC/LigandDisplayForward?ligandId=1512 (https://www.genenames.org/data/gene‐symbol‐report/#!/hgnc_id/HGNC:9327, http://www.uniprot.org/uniprot/P01298) [http://www.ncbi.nlm.nih.gov/pubmed/7592911?dopt=AbstractPlus, http://www.ncbi.nlm.nih.gov/pubmed/7493937?dopt=AbstractPlus, http://www.ncbi.nlm.nih.gov/pubmed/16807358?dopt=AbstractPlus, http://www.ncbi.nlm.nih.gov/pubmed/8643460?dopt=AbstractPlus]	–	–
Agonists	http://www.guidetopharmacology.org/GRAC/LigandDisplayForward?ligandId=1545 [http://www.ncbi.nlm.nih.gov/pubmed/8590988?dopt=AbstractPlus], http://www.guidetopharmacology.org/GRAC/LigandDisplayForward?ligandId=1527, http://www.guidetopharmacology.org/GRAC/LigandDisplayForward?ligandId=3848, http://www.guidetopharmacology.org/GRAC/LigandDisplayForward?ligandId=1546	–	–	–	–
Selective agonists	–	–	–	http://www.guidetopharmacology.org/GRAC/LigandDisplayForward?ligandId=1522 [http://www.ncbi.nlm.nih.gov/pubmed/12069595?dopt=AbstractPlus]	–
Selective antagonists	http://www.guidetopharmacology.org/GRAC/LigandDisplayForward?ligandId=1528 (pIC_50_ 9.5) [http://www.ncbi.nlm.nih.gov/pubmed/9806339?dopt=AbstractPlus], http://www.guidetopharmacology.org/GRAC/LigandDisplayForward?ligandId=1485 (p*K*_i_ 8.1–9.3) [http://www.ncbi.nlm.nih.gov/pubmed/7562541?dopt=AbstractPlus, http://www.ncbi.nlm.nih.gov/pubmed/7562543?dopt=AbstractPlus]	http://www.guidetopharmacology.org/GRAC/LigandDisplayForward?ligandId=1547 (pIC_50_ 8.5) [http://www.ncbi.nlm.nih.gov/pubmed/10611450?dopt=AbstractPlus], http://www.guidetopharmacology.org/GRAC/LigandDisplayForward?ligandId=3504 (pIC_50_ 6.9–7.1) [http://www.ncbi.nlm.nih.gov/pubmed/14617685?dopt=AbstractPlus]	–	http://www.guidetopharmacology.org/GRAC/LigandDisplayForward?ligandId=1565 (p*K*_i_ 7.6) [http://www.ncbi.nlm.nih.gov/pubmed/10872822?dopt=AbstractPlus]	–
Selective allosteric modulators	–	–	http://www.guidetopharmacology.org/GRAC/LigandDisplayForward?ligandId=8494 (Positive) [http://www.ncbi.nlm.nih.gov/pubmed/27294784?dopt=AbstractPlus]	–	–
Labelled ligands	http://www.guidetopharmacology.org/GRAC/LigandDisplayForward?ligandId=3474 (Antagonist) (p*K*_d_ 8.7), [http://www.guidetopharmacology.org/GRAC/LigandDisplayForward?ligandId=3762 (Agonist)	[http://www.guidetopharmacology.org/GRAC/LigandDisplayForward?ligandId=3802 (Agonist)	[http://www.guidetopharmacology.org/GRAC/LigandDisplayForward?ligandId=3800 (Agonist)	[http://www.guidetopharmacology.org/GRAC/LigandDisplayForward?ligandId=3921 (Agonist) [http://www.ncbi.nlm.nih.gov/pubmed/15337369?dopt=AbstractPlus] – Rat	
Comments	Note that Pro^34^‐containing NPY and PYY can also bind Y_4_ and Y_5_ receptors, so strictly speaking are not selective, but are the ’preferred’ agonists.	–	–	–	–

### Comments

The Y_1_ agonists indicated are selective relative to Y_2_ receptors. http://www.guidetopharmacology.org/GRAC/LigandDisplayForward?ligandId=1485 is selective relative to Y_2_, Y_4_ and Y_5_ receptors [http://www.ncbi.nlm.nih.gov/pubmed/8700207?dopt=AbstractPlus]. http://www.guidetopharmacology.org/GRAC/LigandDisplayForward?ligandId=3903) is Y_2_ selective relative to Y_1_ and Y_5_ receptors. PYY‐(3‐36) is Y_2_ selective relative to Y_1_ receptors. Note that Pro34‐containing NPY and PYY can also bind Y_4_ and Y_5_, thus they are selective only relative to Y_2_. The y_6_ receptor is a pseudogene in humans, but is functional in mouse, rabbit and some other mammals.

### Further reading on Neuropeptide Y receptors

Bowers ME *et al*. (2012) Neuropeptide regulation of fear and anxiety: Implications of cholecystokinin, endogenous opioids, and neuropeptide Y. *Physiol. Behav.* **107**: 699‐710 [https://www.ncbi.nlm.nih.gov/pubmed/22429904?dopt=AbstractPlus]

Michel MC *et al*. (1998) XVI. International Union of Pharmacology recommendations for the nomenclature of neuropeptide Y, peptide YY and pancreatic polypeptide receptors. *Pharmacol. Rev.* **50**: 143‐150 [https://www.ncbi.nlm.nih.gov/pubmed/9549761?dopt=AbstractPlus]

Pedragosa‐Badia X *et al*. (2013) Neuropeptide Y receptors: how to get subtype selectivity. *Front En docrinol (Lausanne)* **4**: 5 [https://www.ncbi.nlm.nih.gov/pubmed/23382728?dopt=AbstractPlus]

Zhang L *et al*. (2011) The neuropeptide Y system: pathophysiological and therapeutic implications in obesity and cancer. *Pharmacol. Ther.* **131**: 91‐113 [https://www.ncbi.nlm.nih.gov/pubmed/21439311?dopt=AbstractPlus]

## http://www.guidetopharmacology.org/GRAC/FamilyDisplayForward?familyId=47

### Overview

Neurotensin receptors (**nomenclature as recommended by** **NC‐IUPHAR** [http://www.ncbi.nlm.nih.gov/pubmed/15914470?dopt=AbstractPlus]) are activated by the endogenous tridecapeptide neurotensin (pGlu‐Leu‐Tyr‐Glu‐Asn‐Lys‐Pro‐Arg‐Arg‐Pro‐Tyr‐Ile‐Leu) derived from a precursor (https://www.genenames.org/data/gene‐symbol‐report/#!/hgnc_id/HGNC:8038, http://www.uniprot.org/uniprot/30990), which also generates neuromedin N, an agonist at the NTS_2_ receptor. http://www.guidetopharmacology.org/GRAC/LigandDisplayForward?ligandId=3830) and http://www.guidetopharmacology.org/GRAC/LigandDisplayForward?ligandId=1574) may be used to label NTS_1_ and NTS_2_ receptors at 0.1‐0.3 and 3‐5 nM concentrations respectively.

**Table:** 

Nomenclature	http://www.guidetopharmacology.org/GRAC/ObjectDisplayForward?objectId=309	http://www.guidetopharmacology.org/GRAC/ObjectDisplayForward?objectId=310
HGNC, UniProt	https://www.genenames.org/data/gene‐symbol‐report/#!/hgnc_id/HGNC:8039, http://www.uniprot.org/uniprot/P30989	https://www.genenames.org/data/gene‐symbol‐report/#!/hgnc_id/HGNC:8040, http://www.uniprot.org/uniprot/O95665
Potency order of endogenous ligands	http://www.guidetopharmacology.org/GRAC/LigandDisplayForward?ligandId=1579 (https://www.genenames.org/data/gene‐symbol‐report/#!/hgnc_id/HGNC:8038, http://www.uniprot.org/uniprot/P30990) > http://www.guidetopharmacology.org/GRAC/LigandDisplayForward?ligandId=1578 {Mouse, Rat} [http://www.ncbi.nlm.nih.gov/pubmed/9283723?dopt=AbstractPlus]	http://www.guidetopharmacology.org/GRAC/LigandDisplayForward?ligandId=1579 (https://www.genenames.org/data/gene‐symbol‐report/#!/hgnc_id/HGNC:8038, http://www.uniprot.org/uniprot/P30990) = http://www.guidetopharmacology.org/GRAC/LigandDisplayForward?ligandId=1578 {Mouse, Rat} [http://www.ncbi.nlm.nih.gov/pubmed/8795617?dopt=AbstractPlus]
Selective agonists	http://www.guidetopharmacology.org/GRAC/LigandDisplayForward?ligandId=1570 [http://www.ncbi.nlm.nih.gov/pubmed/12869647?dopt=AbstractPlus] – Rat	http://www.guidetopharmacology.org/GRAC/LigandDisplayForward?ligandId=1586 [http://www.ncbi.nlm.nih.gov/pubmed/8795617?dopt=AbstractPlus, http://www.ncbi.nlm.nih.gov/pubmed/11723247?dopt=AbstractPlus]
Selective antagonists	http://www.guidetopharmacology.org/GRAC/LigandDisplayForward?ligandId=1582 (pIC_50_ 7.5–8.2) [http://www.ncbi.nlm.nih.gov/pubmed/9023294?dopt=AbstractPlus]	–
Labelled ligands	http://www.guidetopharmacology.org/GRAC/LigandDisplayForward?ligandId=1583 (Antagonist) (p*K*_d_ 8.5) [http://www.ncbi.nlm.nih.gov/pubmed/7746272?dopt=AbstractPlus] – Rat	–

### Comments

http://www.guidetopharmacology.org/GRAC/LigandDisplayForward?ligandId=1579 (https://www.genenames.org/data/gene‐symbol‐report/#!/hgnc_id/HGNC:8038, http://www.uniprot.org/uniprot/P30990) appears to be a lowefficacy agonist at the NTS_2_ receptor [http://www.ncbi.nlm.nih.gov/pubmed/9851594?dopt=AbstractPlus], while the NTS_1_ receptor antagonist http://www.guidetopharmacology.org/GRAC/LigandDisplayForward?ligandId=1582 is an agonist at NTS_2_ receptors [http://www.ncbi.nlm.nih.gov/pubmed/9851594?dopt=AbstractPlus]. An additional protein, provisionally termed NTS_3_ (also known as NTR3, gp95 and sortilin; http://www.ensembl.org/Homo_sapiens/Gene/Summary?g=ENSG00000134243;r=1:109852192‐109940573), has been suggested to bind lipoprotein lipase and mediate its degradation [http://www.ncbi.nlm.nih.gov/pubmed/10085125?dopt=AbstractPlus]. It has been reported to interact with the NTS_1_ receptor [http://www.ncbi.nlm.nih.gov/pubmed/12360476?dopt=AbstractPlus] and the NTS_2_ receptor [http://www.ncbi.nlm.nih.gov/pubmed/19891061?dopt=AbstractPlus], and has beenimplicated in hormone trafficking and/or neurotensin uptake. A splice variant of the NTS_2_ receptor bearing 5 transmembrane domains has been identified in mouse [http://www.ncbi.nlm.nih.gov/pubmed/9001400?dopt=AbstractPlus] and later in rat [http://www.ncbi.nlm.nih.gov/pubmed/15637074?dopt=AbstractPlus].

### Further reading on Neurotensin receptors

Boules M *et al*. (2013) Diverse roles of neurotensin agonists in the central nervous system. *Front Endocrinol (Lausanne)* **4**: 36 https://www.ncbi.nlm.nih.gov/pubmed/23526754?dopt=AbstractPlus

Mazella J *et al*. (2012) Neurotensin and its receptors in the control of glucose homeostasis. *Front Endocrinol (Lausanne)* **3**: 143 https://www.ncbi.nlm.nih.gov/pubmed/23230428?dopt=AbstractPlus

Myers RM *et al*. (2009) Cancer, chemistry, and the cell: molecules that interact with the neurotensin receptors. *ACS Chem. Biol.* **4**: 503‐25 https://www.ncbi.nlm.nih.gov/pubmed/19462983?dopt=AbstractPlus

Ouyang Q *et al*. (2017) Oncogenic role of neurotensin and neurotensin receptors in various cancers. *Clin. Exp. Pharmacol. Physiol.* **44**: 841‐846 https://www.ncbi.nlm.nih.gov/pubmed/28556374?dopt=AbstractPlus

## http://www.guidetopharmacology.org/GRAC/FamilyDisplayForward?familyId=50

### Overview

Opioid and opioid‐like receptors are activated by a variety of endogenous peptides including [http://www.guidetopharmacology.org/GRAC/LigandDisplayForward?ligandId=1614 (https://www.genenames.org/data/gene‐symbol‐report/#!/hgnc_id/HGNC:8831, http://www.uniprot.org/uniprot/P01210) (met), http://www.guidetopharmacology.org/GRAC/LigandDisplayForward?ligandId=1613 (https://www.genenames.org/data/gene‐symbol‐report/#!/hgnc_id/HGNC:8831, http://www.uniprot.org/uniprot/P01210) (leu), http://www.guidetopharmacology.org/GRAC/LigandDisplayForward?ligandId=1643 (https://www.genenames.org/data/gene‐symbol‐report/#!/hgnc_id/HGNC:9201, http://www.uniprot.org/uniprot/P01189) (β‐end), http://www.guidetopharmacology.org/GRAC/LigandDisplayForward?ligandId=3737 (https://www.genenames.org/data/gene‐symbol‐report/#!/hgnc_id/HGNC:8820, http://www.uniprot.org/uniprot/P01213), http://www.guidetopharmacology.org/GRAC/LigandDisplayForward?ligandId=1620 (https://www.genenames.org/data/gene‐symbol‐report/#!/hgnc_id/HGNC:8820, http://www.uniprot.org/uniprot/P01213) (dynA), http://www.guidetopharmacology.org/GRAC/LigandDisplayForward?ligandId=1622 (https://www.genenames.org/data/gene‐symbol‐report/#!/hgnc_id/HGNC:8820, http://www.uniprot.org/uniprot/P01213) (dynB), http://www.guidetopharmacology.org/GRAC/LigandDisplayForward?ligandId=3669 (https://www.genenames.org/data/gene‐symbol‐report/#!/hgnc_id/HGNC:8820, http://www.uniprot.org/uniprot/P01213) (Big dyn), http://www.guidetopharmacology.org/GRAC/LigandDisplayForward?ligandId=1681 (https://www.genenames.org/data/gene‐symbol‐report/#!/hgnc_id/HGNC:9163, http://www.uniprot.org/uniprot/Q13519) (N/OFQ); http://www.guidetopharmacology.org/GRAC/LigandDisplayForward?ligandId=1623 and http://www.guidetopharmacology.org/GRAC/LigandDisplayForward?ligandId=3668 are also potential endogenous peptides. The Greek letter nomenclature for the opioid receptors, *μ*, *δ* and *κ*, is well established, and **NC‐IUPHAR** considers this nomenclature appropriate, along with the symbols spelled out (mu, delta, and kappa), and the acronyms, MOP, DOP, and KOP. [http://www.ncbi.nlm.nih.gov/pubmed/24528283?dopt=AbstractPlus, http://www.ncbi.nlm.nih.gov/pubmed/8981566?dopt=AbstractPlus, http://www.ncbi.nlm.nih.gov/pubmed/15914470?dopt=AbstractPlus]. The human N/OFQ receptor, NOP, is considered ’opioid‐related’ rather than opioid because, while it exhibits a high degree of structural homology with the conventional opioid receptors [http://www.ncbi.nlm.nih.gov/pubmed/8137918?dopt=AbstractPlus], it displays a distinct pharmacology. Currently there are numerous clinically used drugs, such as http://www.guidetopharmacology.org/GRAC/LigandDisplayForward?ligandId=1627 and many other opioid analgesics, as well as antagonists such as http://www.guidetopharmacology.org/GRAC/LigandDisplayForward?ligandId=1638, however only for the *μ* receptor.

**Table:** 

Nomenclature	http://www.guidetopharmacology.org/GRAC/ObjectDisplayForward?objectId=317	http://www.guidetopharmacology.org/GRAC/ObjectDisplayForward?objectId=318
HGNC, UniProt	https://www.genenames.org/data/gene‐symbol‐report/#!/hgnc_id/HGNC:8153, http://www.uniprot.org/uniprot/P41143	https://www.genenames.org/data/gene‐symbol‐report/#!/hgnc_id/HGNC:8154, http://www.uniprot.org/uniprot/P41145
Principal endogenous agonists	http://www.guidetopharmacology.org/GRAC/LigandDisplayForward?ligandId=1643 (https://www.genenames.org/data/gene‐symbol‐report/#!/hgnc_id/HGNC:9201, http://www.uniprot.org/uniprot/P01189), http://www.guidetopharmacology.org/GRAC/LigandDisplayForward?ligandId=1613 (https://www.genenames.org/data/gene‐symbol‐report/#!/hgnc_id/HGNC:8831, http://www.uniprot.org/uniprot/P01210), http://www.guidetopharmacology.org/GRAC/LigandDisplayForward?ligandId=1614 (https://www.genenames.org/data/gene‐symbol‐report/#!/hgnc_id/HGNC:8831, http://www.uniprot.org/uniprot/P01210)	http://www.guidetopharmacology.org/GRAC/LigandDisplayForward?ligandId=3669 (https://www.genenames.org/data/gene‐symbol‐report/#!/hgnc_id/HGNC:8820, http://www.uniprot.org/uniprot/P01213), http://www.guidetopharmacology.org/GRAC/LigandDisplayForward?ligandId=1620 (https://www.genenames.org/data/gene‐symbol‐report/#!/hgnc_id/HGNC:8820, http://www.uniprot.org/uniprot/P01213)
Agonists	http://www.guidetopharmacology.org/GRAC/LigandDisplayForward?ligandId=1607 [http://www.ncbi.nlm.nih.gov/pubmed/9686407?dopt=AbstractPlus], http://www.guidetopharmacology.org/GRAC/LigandDisplayForward?ligandId=1625 [http://www.ncbi.nlm.nih.gov/pubmed/9686407?dopt=AbstractPlus], http://www.guidetopharmacology.org/GRAC/LigandDisplayForward?ligandId=1602 [http://www.ncbi.nlm.nih.gov/pubmed/9686407?dopt=AbstractPlus]	–
Sub/family‐selective agonists	http://www.guidetopharmacology.org/GRAC/LigandDisplayForward?ligandId=9294 (Partial agonist) [http://www.ncbi.nlm.nih.gov/pubmed/21177476?dopt=AbstractPlus]	http://www.guidetopharmacology.org/GRAC/LigandDisplayForward?ligandId=9294 [http://www.ncbi.nlm.nih.gov/pubmed/21177476?dopt=AbstractPlus]
Selective agonists	http://www.guidetopharmacology.org/GRAC/LigandDisplayForward?ligandId=9006 [http://www.ncbi.nlm.nih.gov/pubmed/18069089?dopt=AbstractPlus], http://www.guidetopharmacology.org/GRAC/LigandDisplayForward?ligandId=9002 [http://www.ncbi.nlm.nih.gov/pubmed/18788723?dopt=AbstractPlus], http://www.guidetopharmacology.org/GRAC/LigandDisplayForward?ligandId=9004 [http://www.ncbi.nlm.nih.gov/pubmed/18788723?dopt=AbstractPlus], http://www.guidetopharmacology.org/GRAC/LigandDisplayForward?ligandId=1608 [http://www.ncbi.nlm.nih.gov/pubmed/6310598?dopt=AbstractPlus, http://www.ncbi.nlm.nih.gov/pubmed/9686407?dopt=AbstractPlus], [http://www.guidetopharmacology.org/GRAC/LigandDisplayForward?ligandId=4060 [http://www.ncbi.nlm.nih.gov/pubmed/2544892?dopt=AbstractPlus], http://www.guidetopharmacology.org/GRAC/LigandDisplayForward?ligandId=9003 [http://www.ncbi.nlm.nih.gov/pubmed/19694468?dopt=AbstractPlus], http://www.guidetopharmacology.org/GRAC/LigandDisplayForward?ligandId=1611 [http://www.ncbi.nlm.nih.gov/pubmed/8035418?dopt=AbstractPlus, http://www.ncbi.nlm.nih.gov/pubmed/9178661?dopt=AbstractPlus]	http://www.guidetopharmacology.org/GRAC/LigandDisplayForward?ligandId=1652 [http://www.ncbi.nlm.nih.gov/pubmed/14718611?dopt=AbstractPlus, 1671, http://www.ncbi.nlm.nih.gov/pubmed/7624359?dopt=AbstractPlus, http://www.ncbi.nlm.nih.gov/pubmed/9686407?dopt=AbstractPlus, http://www.ncbi.nlm.nih.gov/pubmed/6129321?dopt=AbstractPlus, http://www.ncbi.nlm.nih.gov/pubmed/7869844?dopt=AbstractPlus, http://www.ncbi.nlm.nih.gov/pubmed/9262330?dopt=AbstractPlus], http://www.guidetopharmacology.org/GRAC/LigandDisplayForward?ligandId=1646 [http://www.ncbi.nlm.nih.gov/pubmed/2178014?dopt=AbstractPlus, http://www.ncbi.nlm.nih.gov/pubmed/14613319?dopt=AbstractPlus], http://www.guidetopharmacology.org/GRAC/LigandDisplayForward?ligandId=1655 [http://www.ncbi.nlm.nih.gov/pubmed/2986999?dopt=AbstractPlus, http://www.ncbi.nlm.nih.gov/pubmed/2986999?dopt=AbstractPlus], http://www.guidetopharmacology.org/GRAC/LigandDisplayForward?ligandId=1666 [http://www.ncbi.nlm.nih.gov/pubmed/15869877?dopt=AbstractPlus, http://www.ncbi.nlm.nih.gov/pubmed/12192085?dopt=AbstractPlus]
Antagonists	http://www.guidetopharmacology.org/GRAC/LigandDisplayForward?ligandId=9838 (p*K*_i_ 9.8) [http://www.ncbi.nlm.nih.gov/pubmed/22194444?dopt=AbstractPlus, http://www.ncbi.nlm.nih.gov/pubmed/29524334?dopt=AbstractPlus], http://www.guidetopharmacology.org/GRAC/LigandDisplayForward?ligandId=1639 (p*K*_i_ 8) [http://www.ncbi.nlm.nih.gov/pubmed/9686407?dopt=AbstractPlus], http://www.guidetopharmacology.org/GRAC/LigandDisplayForward?ligandId=1638 (p*K*_i_ 7.2) [http://www.ncbi.nlm.nih.gov/pubmed/9686407?dopt=AbstractPlus]	http://www.guidetopharmacology.org/GRAC/LigandDisplayForward?ligandId=1670 (p*K*_i_ 9.1–10.2) [http://www.ncbi.nlm.nih.gov/pubmed/9686407?dopt=AbstractPlus, http://www.ncbi.nlm.nih.gov/pubmed/9262330?dopt=AbstractPlus], http://www.guidetopharmacology.org/GRAC/LigandDisplayForward?ligandId=1628 (p*K*_i_ 9.5) [http://www.ncbi.nlm.nih.gov/pubmed/9686407?dopt=AbstractPlus], http://www.guidetopharmacology.org/GRAC/LigandDisplayForward?ligandId=1639 (p*K*_i_ 8.4–9.4) [1671, http://www.ncbi.nlm.nih.gov/pubmed/7624359?dopt=AbstractPlus, http://www.ncbi.nlm.nih.gov/pubmed/9686407?dopt=AbstractPlus], http://www.guidetopharmacology.org/GRAC/LigandDisplayForward?ligandId=1638 (p*K*_i_ 7.6–8.6) [1671, http://www.ncbi.nlm.nih.gov/pubmed/7624359?dopt=AbstractPlus, http://www.ncbi.nlm.nih.gov/pubmed/9686407?dopt=AbstractPlus, http://www.ncbi.nlm.nih.gov/pubmed/7869844?dopt=AbstractPlus, http://www.ncbi.nlm.nih.gov/pubmed/9262330?dopt=AbstractPlus]
Sub/family‐selective antagonists	http://www.guidetopharmacology.org/GRAC/LigandDisplayForward?ligandId=8870 (p*K*_i_ 7.7) [http://www.ncbi.nlm.nih.gov/pubmed/9686407?dopt=AbstractPlus, http://www.ncbi.nlm.nih.gov/pubmed/12801588?dopt=AbstractPlus]	http://www.guidetopharmacology.org/GRAC/LigandDisplayForward?ligandId=8870 (p*K*_i_ 8.9) [http://www.ncbi.nlm.nih.gov/pubmed/9686407?dopt=AbstractPlus, http://www.ncbi.nlm.nih.gov/pubmed/25635572?dopt=AbstractPlus]
Selective antagonists	http://www.guidetopharmacology.org/GRAC/LigandDisplayForward?ligandId=1640 (p*K*_i_ 10) [http://www.ncbi.nlm.nih.gov/pubmed/1851833?dopt=AbstractPlus, http://www.ncbi.nlm.nih.gov/pubmed/9686407?dopt=AbstractPlus], http://www.guidetopharmacology.org/GRAC/LigandDisplayForward?ligandId=1641 (p*K*_i_ 9.7) [http://www.ncbi.nlm.nih.gov/pubmed/2832195?dopt=AbstractPlus, http://www.ncbi.nlm.nih.gov/pubmed/9686407?dopt=AbstractPlus], http://www.guidetopharmacology.org/GRAC/LigandDisplayForward?ligandId=1637 (Inverse agonist) (p*K*_i_ 9) [http://www.ncbi.nlm.nih.gov/pubmed/8230106?dopt=AbstractPlus, http://www.ncbi.nlm.nih.gov/pubmed/9686407?dopt=AbstractPlus]	http://www.guidetopharmacology.org/GRAC/LigandDisplayForward?ligandId=1642 (p*K*_i_ 8.9–11) [1671, http://www.ncbi.nlm.nih.gov/pubmed/2444704?dopt=AbstractPlus, http://www.ncbi.nlm.nih.gov/pubmed/7624359?dopt=AbstractPlus, http://www.ncbi.nlm.nih.gov/pubmed/9686407?dopt=AbstractPlus, http://www.ncbi.nlm.nih.gov/pubmed/7869844?dopt=AbstractPlus, http://www.ncbi.nlm.nih.gov/pubmed/9262330?dopt=AbstractPlus], http://www.guidetopharmacology.org/GRAC/LigandDisplayForward?ligandId=1669 (p*K*_i_ 9.7–9.9) [http://www.ncbi.nlm.nih.gov/pubmed/10822054?dopt=AbstractPlus, 1671, http://www.ncbi.nlm.nih.gov/pubmed/10893314?dopt=AbstractPlus], http://www.guidetopharmacology.org/GRAC/LigandDisplayForward?ligandId=7362 (p*K*_i_ 9–9.4) [http://www.ncbi.nlm.nih.gov/pubmed/23976952?dopt=AbstractPlus, http://www.ncbi.nlm.nih.gov/pubmed/11495579?dopt=AbstractPlus, http://www.ncbi.nlm.nih.gov/pubmed/25635572?dopt=AbstractPlus]
Labelled ligands	http://www.guidetopharmacology.org/GRAC/LigandDisplayForward?ligandId=3829 (Antagonist) (p*K*_d_ 10.4) [http://www.ncbi.nlm.nih.gov/pubmed/1313133?dopt=AbstractPlus] – Rat, http://www.guidetopharmacology.org/GRAC/LigandDisplayForward?ligandId=9065 (Selective Agonist) [http://www.ncbi.nlm.nih.gov/pubmed/1329092?dopt=AbstractPlus], http://www.guidetopharmacology.org/GRAC/LigandDisplayForward?ligandId=1612 (Agonist) [http://www.ncbi.nlm.nih.gov/pubmed/1313812?dopt=AbstractPlus, http://www.ncbi.nlm.nih.gov/pubmed/9686407?dopt=AbstractPlus], http://www.guidetopharmacology.org/GRAC/LigandDisplayForward?ligandId=3819 (Agonist) [http://www.ncbi.nlm.nih.gov/pubmed/2986120?dopt=AbstractPlus], http://www.guidetopharmacology.org/GRAC/LigandDisplayForward?ligandId=3816 (Agonist) [http://www.ncbi.nlm.nih.gov/pubmed/1313131?dopt=AbstractPlus], http://www.guidetopharmacology.org/GRAC/LigandDisplayForward?ligandId=3828 (Antagonist) [http://www.ncbi.nlm.nih.gov/pubmed/9696425?dopt=AbstractPlus]	http://www.guidetopharmacology.org/GRAC/LigandDisplayForward?ligandId=1612 (Antagonist) (p*K*_d_ 9.1) [http://www.ncbi.nlm.nih.gov/pubmed/1313812?dopt=AbstractPlus, http://www.ncbi.nlm.nih.gov/pubmed/7624359?dopt=AbstractPlus], http://www.guidetopharmacology.org/GRAC/LigandDisplayForward?ligandId=1656 (Agonist) [http://www.ncbi.nlm.nih.gov/pubmed/2986999?dopt=AbstractPlus, 1671, http://www.ncbi.nlm.nih.gov/pubmed/7624359?dopt=AbstractPlus], http://www.guidetopharmacology.org/GRAC/LigandDisplayForward?ligandId=3475 (Agonist) [http://www.ncbi.nlm.nih.gov/pubmed/11239918?dopt=AbstractPlus]

**Table:** 

Nomenclature	http://www.guidetopharmacology.org/GRAC/ObjectDisplayForward?objectId=319	http://www.guidetopharmacology.org/GRAC/ObjectDisplayForward?objectId=320
HGNC, UniProt	https://www.genenames.org/data/gene‐symbol‐report/#!/hgnc_id/HGNC:8156, http://www.uniprot.org/uniprot/P35372	https://www.genenames.org/data/gene‐symbol‐report/#!/hgnc_id/HGNC:8155, http://www.uniprot.org/uniprot/P41146
Potential endogenous agonists	http://www.guidetopharmacology.org/GRAC/LigandDisplayForward?ligandId=1623, http://www.guidetopharmacology.org/GRAC/LigandDisplayForward?ligandId=3668	–
Principal endogenous agonists	http://www.guidetopharmacology.org/GRAC/LigandDisplayForward?ligandId=1643 (https://www.genenames.org/data/gene‐symbol‐report/#!/hgnc_id/HGNC:9201, http://www.uniprot.org/uniprot/P01189), http://www.guidetopharmacology.org/GRAC/LigandDisplayForward?ligandId=1614 (https://www.genenames.org/data/gene‐symbol‐report/#!/hgnc_id/HGNC:8831, http://www.uniprot.org/uniprot/P01210), http://www.guidetopharmacology.org/GRAC/LigandDisplayForward?ligandId=1613 (https://www.genenames.org/data/gene‐symbol‐report/#!/hgnc_id/HGNC:8831, http://www.uniprot.org/uniprot/P01210)	–
Endogenous agonists	–	http://www.guidetopharmacology.org/GRAC/LigandDisplayForward?ligandId=1681 (https://www.genenames.org/data/gene‐symbol‐report/#!/hgnc_id/HGNC:9163, http://www.uniprot.org/uniprot/Q13519) [http://www.ncbi.nlm.nih.gov/pubmed/9413015?dopt=AbstractPlus, http://www.ncbi.nlm.nih.gov/pubmed/12070757?dopt=AbstractPlus, http://www.ncbi.nlm.nih.gov/pubmed/10369464?dopt=AbstractPlus]
Agonists	http://www.guidetopharmacology.org/GRAC/LigandDisplayForward?ligandId=7595 [http://www.ncbi.nlm.nih.gov/pubmed/12502358?dopt=AbstractPlus], http://www.guidetopharmacology.org/GRAC/LigandDisplayForward?ligandId=7082 [http://www.ncbi.nlm.nih.gov/pubmed/19282177?dopt=AbstractPlus], http://www.guidetopharmacology.org/GRAC/LigandDisplayForward?ligandId=1626 [http://www.ncbi.nlm.nih.gov/pubmed/9686407?dopt=AbstractPlus], http://www.guidetopharmacology.org/GRAC/LigandDisplayForward?ligandId=1670 (Partial agonist) [http://www.ncbi.nlm.nih.gov/pubmed/9686407?dopt=AbstractPlus], http://www.guidetopharmacology.org/GRAC/LigandDisplayForward?ligandId=5458 [http://www.ncbi.nlm.nih.gov/pubmed/11585443?dopt=AbstractPlus], http://www.guidetopharmacology.org/GRAC/LigandDisplayForward?ligandId=9838 [http://www.ncbi.nlm.nih.gov/pubmed/22194444?dopt=AbstractPlus, http://www.ncbi.nlm.nih.gov/pubmed/29524334?dopt=AbstractPlus], http://www.guidetopharmacology.org/GRAC/LigandDisplayForward?ligandId=1671 [http://www.ncbi.nlm.nih.gov/pubmed/9686407?dopt=AbstractPlus], http://www.guidetopharmacology.org/GRAC/LigandDisplayForward?ligandId=7477 [http://www.ncbi.nlm.nih.gov/pubmed/17656655?dopt=AbstractPlus], http://www.guidetopharmacology.org/GRAC/LigandDisplayForward?ligandId=7221 [http://www.ncbi.nlm.nih.gov/pubmed/11585443?dopt=AbstractPlus]	–
Sub/family‐selective agonists	http://www.guidetopharmacology.org/GRAC/LigandDisplayForward?ligandId=9294 (Partial agonist) [http://www.ncbi.nlm.nih.gov/pubmed/21177476?dopt=AbstractPlus]	http://www.guidetopharmacology.org/GRAC/LigandDisplayForward?ligandId=8866 [http://www.ncbi.nlm.nih.gov/pubmed/24713140?dopt=AbstractPlus], http://www.guidetopharmacology.org/GRAC/LigandDisplayForward?ligandId=9294 (Partial agonist) [http://www.ncbi.nlm.nih.gov/pubmed/21177476?dopt=AbstractPlus]
Selective agonists	http://www.guidetopharmacology.org/GRAC/LigandDisplayForward?ligandId=3534 [http://www.ncbi.nlm.nih.gov/pubmed/21215785?dopt=AbstractPlus], http://www.guidetopharmacology.org/GRAC/LigandDisplayForward?ligandId=1647 [http://www.ncbi.nlm.nih.gov/pubmed/6263640?dopt=AbstractPlus, http://www.ncbi.nlm.nih.gov/pubmed/9686407?dopt=AbstractPlus], http://www.guidetopharmacology.org/GRAC/LigandDisplayForward?ligandId=7215 [http://www.ncbi.nlm.nih.gov/pubmed/15454210?dopt=AbstractPlus], http://www.guidetopharmacology.org/GRAC/LigandDisplayForward?ligandId=1627 [http://www.ncbi.nlm.nih.gov/pubmed/2549383?dopt=AbstractPlus, http://www.ncbi.nlm.nih.gov/pubmed/9686407?dopt=AbstractPlus], http://www.guidetopharmacology.org/GRAC/LigandDisplayForward?ligandId=1671 [http://www.ncbi.nlm.nih.gov/pubmed/6313901?dopt=AbstractPlus, http://www.ncbi.nlm.nih.gov/pubmed/9686407?dopt=AbstractPlus]	http://www.guidetopharmacology.org/GRAC/LigandDisplayForward?ligandId=1682 [http://www.ncbi.nlm.nih.gov/pubmed/12070757?dopt=AbstractPlus, http://www.ncbi.nlm.nih.gov/pubmed/9191955?dopt=AbstractPlus, http://www.ncbi.nlm.nih.gov/pubmed/12967935?dopt=AbstractPlus, http://www.ncbi.nlm.nih.gov/pubmed/10369464?dopt=AbstractPlus], http://www.guidetopharmacology.org/GRAC/LigandDisplayForward?ligandId=1680 (Partial agonist) [http://www.ncbi.nlm.nih.gov/pubmed/9353393?dopt=AbstractPlus, http://www.ncbi.nlm.nih.gov/pubmed/12967935?dopt=AbstractPlus], http://www.guidetopharmacology.org/GRAC/LigandDisplayForward?ligandId=8868 [http://www.ncbi.nlm.nih.gov/pubmed/18492950?dopt=AbstractPlus], http://www.guidetopharmacology.org/GRAC/LigandDisplayForward?ligandId=1684 [http://www.ncbi.nlm.nih.gov/pubmed/10758169?dopt=AbstractPlus, http://www.ncbi.nlm.nih.gov/pubmed/11006485?dopt=AbstractPlus], http://www.guidetopharmacology.org/GRAC/LigandDisplayForward?ligandId=9747 [http://www.ncbi.nlm.nih.gov/pubmed/29232769?dopt=AbstractPlus]
Antagonists	http://www.guidetopharmacology.org/GRAC/LigandDisplayForward?ligandId=1639 (p*K*_i_ 9.1–9.7) [http://www.ncbi.nlm.nih.gov/pubmed/24973897?dopt=AbstractPlus, http://www.ncbi.nlm.nih.gov/pubmed/9686407?dopt=AbstractPlus], http://www.guidetopharmacology.org/GRAC/LigandDisplayForward?ligandId=1628 (p*K*_i_ 9.5) [http://www.ncbi.nlm.nih.gov/pubmed/9686407?dopt=AbstractPlus], http://www.guidetopharmacology.org/GRAC/LigandDisplayForward?ligandId=1629 (p*K*i 8.9) [http://www.ncbi.nlm.nih.gov/pubmed/9686407?dopt=AbstractPlus], http://www.guidetopharmacology.org/GRAC/LigandDisplayForward?ligandId=1638 (p*K*_i_ 8.9) [http://www.ncbi.nlm.nih.gov/pubmed/9686407?dopt=AbstractPlus], http://www.guidetopharmacology.org/GRAC/LigandDisplayForward?ligandId=7563 (p*K*_i_ 8.7) [http://www.ncbi.nlm.nih.gov/pubmed/19282177?dopt=AbstractPlus]	–
Sub/family‐selective antagonists	http://www.guidetopharmacology.org/GRAC/LigandDisplayForward?ligandId=8870 (p*K*_i_ 8.8) [http://www.ncbi.nlm.nih.gov/pubmed/9686407?dopt=AbstractPlus, http://www.ncbi.nlm.nih.gov/pubmed/25635572?dopt=AbstractPlus]	http://www.guidetopharmacology.org/GRAC/LigandDisplayForward?ligandId=8870 (p*K*_i_ 8.8) [http://www.ncbi.nlm.nih.gov/pubmed/25635572?dopt=AbstractPlus]
Selective antagonists	http://www.guidetopharmacology.org/GRAC/LigandDisplayForward?ligandId=7471 (p*K*_i_ 9.3) [http://www.ncbi.nlm.nih.gov/pubmed/18313920?dopt=AbstractPlus], http://www.guidetopharmacology.org/GRAC/LigandDisplayForward?ligandId=7209 (p*K*_i_ 8.8–9.3) [http://www.ncbi.nlm.nih.gov/pubmed/21621410?dopt=AbstractPlus], http://www.guidetopharmacology.org/GRAC/LigandDisplayForward?ligandId=1635 (p*K*_i_ 8.6) [http://www.ncbi.nlm.nih.gov/pubmed/6313901?dopt=AbstractPlus, http://www.ncbi.nlm.nih.gov/pubmed/9686407?dopt=AbstractPlus]	http://www.guidetopharmacology.org/GRAC/LigandDisplayForward?ligandId=1694 (p*K*_i_ 10.2) [http://www.ncbi.nlm.nih.gov/pubmed/12010780?dopt=AbstractPlus], http://www.guidetopharmacology.org/GRAC/LigandDisplayForward?ligandId=9462 (p*K*_i_ 10) [http://www.ncbi.nlm.nih.gov/pubmed/24678969?dopt=AbstractPlus], http://www.guidetopharmacology.org/GRAC/LigandDisplayForward?ligandId=7361 (p*K*_i_ 9.6) [http://www.ncbi.nlm.nih.gov/pubmed/19445927?dopt=AbstractPlus], http://www.guidetopharmacology.org/GRAC/LigandDisplayForward?ligandId=1693 (p*K*_i_ 9.2–9.5) [http://www.ncbi.nlm.nih.gov/pubmed/17329552?dopt=AbstractPlus, http://www.ncbi.nlm.nih.gov/pubmed/14593080?dopt=AbstractPlus], http://www.guidetopharmacology.org/GRAC/LigandDisplayForward?ligandId=1691 (pIC_50_ 8.3) [http://www.ncbi.nlm.nih.gov/pubmed/10602690?dopt=AbstractPlus]
Allosteric modulators	http://www.guidetopharmacology.org/GRAC/LigandDisplayForward?ligandId=9159 (Neutral) (p*K*_B_ 6) [http://www.ncbi.nlm.nih.gov/pubmed/23754417?dopt=AbstractPlus], http://www.guidetopharmacology.org/GRAC/LigandDisplayForward?ligandId=9156 (Positive) (p*K*_B_ 5.7) [http://www.ncbi.nlm.nih.gov/pubmed/23754417?dopt=AbstractPlus], http://www.guidetopharmacology.org/GRAC/LigandDisplayForward?ligandId=9160 (Neutral) (p*K*_B_ 5.7) [http://www.ncbi.nlm.nih.gov/pubmed/23754417?dopt=AbstractPlus], http://www.guidetopharmacology.org/GRAC/LigandDisplayForward?ligandId=9157 (Positive) (p*K*_B_ 5.3) [http://www.ncbi.nlm.nih.gov/pubmed/23754417?dopt=AbstractPlus]	–
Labelled ligands	http://www.guidetopharmacology.org/GRAC/LigandDisplayForward?ligandId=1612 (Antagonist) (p*K*_d_ 10.1) [http://www.ncbi.nlm.nih.gov/pubmed/8114680?dopt=AbstractPlus] – Mouse, http://www.guidetopharmacology.org/GRAC/LigandDisplayForward?ligandId=3815 (Agonist) [http://www.ncbi.nlm.nih.gov/pubmed/8114680?dopt=AbstractPlus] – Rat, http://www.guidetopharmacology.org/GRAC/LigandDisplayForward?ligandId=3832 (Agonist) [http://www.ncbi.nlm.nih.gov/pubmed/3030778?dopt=AbstractPlus] – Rat	http://www.guidetopharmacology.org/GRAC/LigandDisplayForward?ligandId=1688 (Agonist) [http://www.ncbi.nlm.nih.gov/pubmed/9353393?dopt=AbstractPlus, http://www.ncbi.nlm.nih.gov/pubmed/8849681?dopt=AbstractPlus]

### Comments

Three naloxone‐sensitive opioid receptor genes havebeen identified in humans, and while the *μ*‐receptor in particular may be subject to extensive alternative splicing [http://www.ncbi.nlm.nih.gov/pubmed/24076545?dopt=AbstractPlus], these putative isoforms have not been correlated with any of the subtypesof receptor proposed inyears past. Opioid receptors may heterodimerize with each other or with other 7TM receptors [http://www.ncbi.nlm.nih.gov/pubmed/10385123?dopt=AbstractPlus], and give rise to complexes with a unique pharmacology, however, evidence for such heterodimers in native cells is equivocal and the consequences of this heterodimerization for signalling remains largely unknown. For *μ*‐opioid receptors at least, dimerization does not seem to be required for signalling [http://www.ncbi.nlm.nih.gov/pubmed/19542234?dopt=AbstractPlus]. A distinct met‐enkephalin receptor lacking structural resemblance to the opioid receptors listed has been identified (https://www.genenames.org/data/gene‐symbol‐report/#!/hgnc_id/HGNC:15768, http://www.uniprot.org/uniprot/9NZT2) and termed an opioid growth factor receptor [http://www.ncbi.nlm.nih.gov/pubmed/11890982?dopt=AbstractPlus].

http://www.guidetopharmacology.org/GRAC/LigandDisplayForward?ligandId=1623 and http://www.guidetopharmacology.org/GRAC/LigandDisplayForward?ligandId=3668 have been identified as highly selective, putative endogenous agonists for the *μ*‐opioid receptor. At present, however, the mechanisms for endomorphin synthesis *in vivo* have not been established, and there is no gene identified that encodes for either. Thus, the status of these peptides as endogenous ligands remains unproven.

Two areas of increasing importance in defining opioid receptor function are the presence of functionally relevant single nucleotide polymorphisms in human *μ*‐receptors [http://www.ncbi.nlm.nih.gov/pubmed/19116204?dopt=AbstractPlus] and the identification of biased signalling by opioid receptor ligands, in particular, compounds previously characterized as antagonists [http://www.ncbi.nlm.nih.gov/pubmed/17702750?dopt=AbstractPlus]. Pathway bias for agonists makes general rank orders of potency and efficacy somewhat obsolete, so these do not appear in the table. As ever, the mechanisms underlying the acute and long term regulation of opiod receptor function are the subject of intense investigation and debate.

The richness of opioid receptor pharmacology has been enhanced with the recent discovery of allosteric modulators of *μ* and *δ* receptors, notably thepositive allosteric modulators and silent allosteric “antagonists” outlined in [http://www.ncbi.nlm.nih.gov/pubmed/23754417?dopt=AbstractPlus, http://www.ncbi.nlm.nih.gov/pubmed/25901762?dopt=AbstractPlus]. Negative allosteric modulation of opioid receptors has been previously suggested [http://www.ncbi.nlm.nih.gov/pubmed/16489449?dopt=AbstractPlus], whether all compounds are acting at a similar site remains to be established.

### Further reading on Opioid receptors

Butelman ER *et al*. (2012) *κ*‐opioid receptor/dynorphin system: genetic and pharmacotherapeutic implications for addiction. *Trends Neurosci.* **35**: 587‐96 https://www.ncbi.nlm.nih.gov/pubmed/22709632?dopt=AbstractPlus

Cox BM *et al*. (2015) Challenges for opioid receptor nomenclature: IUPHAR Review 9. *Br. J. Pharmacol.* **172**: 317‐23 https://www.ncbi.nlm.nih.gov/pubmed/24528283?dopt=AbstractPlus

Pradhan AA *et al*. (2011) The delta opioid receptor: an evolving target for the treatment of brain disorders. *Trends Pharmacol. Sci.* **32**: 581‐90 https://www.ncbi.nlm.nih.gov/pubmed/21925742?dopt=AbstractPlus

Williams JT *et al*. (2013) Regulation of *μ*‐opioid receptors: desensitization, phosphorylation, internalization, and tolerance. *Pharmacol. Rev.* **65**: 223‐54 https://www.ncbi.nlm.nih.gov/pubmed/23321159?dopt=AbstractPlus

## http://www.guidetopharmacology.org/GRAC/FamilyDisplayForward?familyId=51

### Overview

Orexin receptors **(nomenclature as agreed by the NC‐IUPHAR Subcommittee on Orexin receptors** [http://www.ncbi.nlm.nih.gov/pubmed/15914470?dopt=AbstractPlus]) are activated by the endogenous polypeptides http://www.guidetopharmacology.org/GRAC/LigandDisplayForward?ligandId=1697 (https://www.genenames.org/data/gene‐symbol‐report/%23!/hgnc_id/HGNC:4847, http://www.uniprot.org/uniprot/O43612)and http://www.guidetopharmacology.org/GRAC/LigandDisplayForward?ligandId=1699 (https://www.genenames.org/data/gene‐symbol‐report/%23!/hgnc_id/HGNC:4847,http://www.uniprot.org/uniprot/O43612) (also known as hypocretin‐1 and ‐2; 33 and 28 aa) derived from a common precursor, https://www.genenames.org/data/gene‐symbol‐report/%23!/hgnc_id/HGNC:4847, by proteolytic cleavage and some typical peptide modifications [http://www.ncbi.nlm.nih.gov/pubmed/9491897?dopt=AbstractPlus]. Currently the only orexin receptor ligand in clinical use is http://www.guidetopharmacology.org/GRAC/LigandDisplayForward?ligandId=2890,whichisused as a hypnotic. Orexin receptor crystal structures have been solved [http://www.ncbi.nlm.nih.gov/pubmed/26950369?dopt=AbstractPlus,http://www.ncbi.nlm.nih.gov/pubmed/25533960?dopt=AbstractPlus].

**Table:** 

Nomenclature	http://www.guidetopharmacology.org/GRAC/ObjectDisplayForward?objectId=321	http://www.guidetopharmacology.org/GRAC/ObjectDisplayForward?objectId=322
HGNC, UniProt	https://www.genenames.org/data/gene‐symbol‐report/#!/hgnc_id/HGNC:4848, http://www.uniprot.org/uniprot/O43613	https://www.genenames.org/data/gene‐symbol‐report/#!/hgnc_id/HGNC:4849, http://www.uniprot.org/uniprot/O43614
Potency order of endogenous ligands	http://www.guidetopharmacology.org/GRAC/LigandDisplayForward?ligandId=1697 (https://www.genenames.org/data/gene‐symbol‐report/#!/hgnc_id/HGNC:4847, http://www.uniprot.org/uniprot/O43612) > http://www.guidetopharmacology.org/GRAC/LigandDisplayForward?ligandId=1699, http://www.uniprot.org/uniprot/O43612)	http://www.guidetopharmacology.org/GRAC/LigandDisplayForward?ligandId=1697 (https://www.genenames.org/data/gene‐symbol‐report/#!/hgnc_id/HGNC:4847, http://www.uniprot.org/uniprot/O43612) = http://www.guidetopharmacology.org/GRAC/LigandDisplayForward?ligandId=1699, http://www.uniprot.org/uniprot/O43612)
Selective agonists	–	[http://www.guidetopharmacology.org/GRAC/LigandDisplayForward?ligandId=1700, http://www.guidetopharmacology.org/GRAC/LigandDisplayForward?ligandId=1700]http://www.guidetopharmacology.org/GRAC/LigandDisplayForward?ligandId=1700 [http://www.ncbi.nlm.nih.gov/pubmed/12467628?dopt=AbstractPlus, http://www.ncbi.nlm.nih.gov/pubmed/21362456?dopt=AbstractPlus], http://www.guidetopharmacology.org/GRAC/LigandDisplayForward?ligandId=9305 [http://www.ncbi.nlm.nih.gov/pubmed/26267383?dopt=AbstractPlus, http://www.ncbi.nlm.nih.gov/pubmed/30194937?dopt=AbstractPlus], http://www.guidetopharmacology.org/GRAC/LigandDisplayForward?ligandId=10277 [http://www.ncbi.nlm.nih.gov/pubmed/28507129?dopt=AbstractPlus, http://www.ncbi.nlm.nih.gov/pubmed/28507129?dopt=AbstractPlus]
Antagonists	http://www.guidetopharmacology.org/GRAC/LigandDisplayForward?ligandId=4461 (p*K*_i_ 9.3–9.6) [http://www.ncbi.nlm.nih.gov/pubmed/24376396?dopt=AbstractPlus, http://www.ncbi.nlm.nih.gov/pubmed/20565075?dopt=AbstractPlus], http://www.guidetopharmacology.org/GRAC/LigandDisplayForward?ligandId=2890 (p*K*_i_ 8.7–9.3) [http://www.ncbi.nlm.nih.gov/pubmed/24376396?dopt=AbstractPlus, http://www.ncbi.nlm.nih.gov/pubmed/23692283?dopt=AbstractPlus], http://www.guidetopharmacology.org/GRAC/LigandDisplayForward?ligandId=4460 (p*K*_i_ 8.4–9.1) [http://www.ncbi.nlm.nih.gov/pubmed/24376396?dopt=AbstractPlus, http://www.ncbi.nlm.nih.gov/pubmed/24376396?dopt=AbstractPlus, http://www.ncbi.nlm.nih.gov/pubmed/22019562?dopt=AbstractPlus], http://www.guidetopharmacology.org/GRAC/LigandDisplayForward?ligandId=9304 (p*K*_i_ 8.5) [http://www.ncbi.nlm.nih.gov/pubmed/18207395?dopt=AbstractPlus], http://www.guidetopharmacology.org/GRAC/LigandDisplayForward?ligandId=2886 (p*K*_i_ 7.8–8.5) [http://www.ncbi.nlm.nih.gov/pubmed/24376396?dopt=AbstractPlus, http://www.ncbi.nlm.nih.gov/pubmed/22796453?dopt=AbstractPlus, http://www.ncbi.nlm.nih.gov/pubmed/19751316?dopt=AbstractPlus, http://www.ncbi.nlm.nih.gov/pubmed/19542319?dopt=AbstractPlus, http://www.ncbi.nlm.nih.gov/pubmed/20404073?dopt=AbstractPlus], http://www.guidetopharmacology.org/GRAC/LigandDisplayForward?ligandId=10280 (p*K*_i_ 7.6–8) [http://www.ncbi.nlm.nih.gov/pubmed/19751316?dopt=AbstractPlus]	http://www.guidetopharmacology.org/GRAC/LigandDisplayForward?ligandId=4461 (p*K*_i_ 8.9–9.8) [http://www.ncbi.nlm.nih.gov/pubmed/24376396?dopt=AbstractPlus, http://www.ncbi.nlm.nih.gov/pubmed/20565075?dopt=AbstractPlus], http://www.guidetopharmacology.org/GRAC/LigandDisplayForward?ligandId=9304 (p*K*_i_ 9.7) [http://www.ncbi.nlm.nih.gov/pubmed/18207395?dopt=AbstractPlus], http://www.guidetopharmacology.org/GRAC/LigandDisplayForward?ligandId=2890 (p*K*_i_ 8.9–9.5) [http://www.ncbi.nlm.nih.gov/pubmed/24376396?dopt=AbstractPlus, http://www.ncbi.nlm.nih.gov/pubmed/23692283?dopt=AbstractPlus], http://www.guidetopharmacology.org/GRAC/LigandDisplayForward?ligandId=10280 (p*K*_i_ 8.5–9.3) [http://www.ncbi.nlm.nih.gov/pubmed/19751316?dopt=AbstractPlus, http://www.ncbi.nlm.nih.gov/pubmed/19542319?dopt=AbstractPlus], http://www.guidetopharmacology.org/GRAC/LigandDisplayForward?ligandId=4460 (p*K*i 8.9–9.1) [http://www.ncbi.nlm.nih.gov/pubmed/24376396?dopt=AbstractPlus, http://www.ncbi.nlm.nih.gov/pubmed/24376396?dopt=AbstractPlus, http://www.ncbi.nlm.nih.gov/pubmed/22019562?dopt=AbstractPlus]
Selective antagonists	http://www.guidetopharmacology.org/GRAC/LigandDisplayForward?ligandId=9136 (70–300‐fold selective) (p*K*_i_ 8.7–9.3) [http://www.ncbi.nlm.nih.gov/pubmed/14691055?dopt=AbstractPlus, http://www.ncbi.nlm.nih.gov/pubmed/19751316?dopt=AbstractPlus, http://www.ncbi.nlm.nih.gov/pubmed/19542319?dopt=AbstractPlus, http://www.ncbi.nlm.nih.gov/pubmed/20404073?dopt=AbstractPlus], http://www.guidetopharmacology.org/GRAC/LigandDisplayForward?ligandId=1703 (50–150‐fold selective) (p*K*_i_ 7.2–7.9) [http://www.ncbi.nlm.nih.gov/pubmed/14691055?dopt=AbstractPlus, http://www.ncbi.nlm.nih.gov/pubmed/19542319?dopt=AbstractPlus, http://www.ncbi.nlm.nih.gov/pubmed/11459658?dopt=AbstractPlus, http://www.ncbi.nlm.nih.gov/pubmed/22079339?dopt=AbstractPlus, http://www.ncbi.nlm.nih.gov/pubmed/11250867?dopt=AbstractPlus], http://www.guidetopharmacology.org/GRAC/LigandDisplayForward?ligandId=1704 (60–80‐fold selective) (p*K*_i_ 7.2–7.9) [http://www.ncbi.nlm.nih.gov/pubmed/14691055?dopt=AbstractPlus, http://www.ncbi.nlm.nih.gov/pubmed/23692283?dopt=AbstractPlus]	http://www.guidetopharmacology.org/GRAC/LigandDisplayForward?ligandId=4037 (300–3000‐fold selective) (p*K*_i_ 8.4–9.2) [http://www.ncbi.nlm.nih.gov/pubmed/19751316?dopt=AbstractPlus, http://www.ncbi.nlm.nih.gov/pubmed/19542319?dopt=AbstractPlus, http://www.ncbi.nlm.nih.gov/pubmed/20404073?dopt=AbstractPlus, http://www.ncbi.nlm.nih.gov/pubmed/23692283?dopt=AbstractPlus, http://www.ncbi.nlm.nih.gov/pubmed/21679703?dopt=AbstractPlus], http://www.guidetopharmacology.org/GRAC/LigandDisplayForward?ligandId=1701 (200–800‐fold selective) (p*K*_i_ 7.7–8.4) [http://www.ncbi.nlm.nih.gov/pubmed/20565075?dopt=AbstractPlus, http://www.ncbi.nlm.nih.gov/pubmed/15261275?dopt=AbstractPlus], http://www.guidetopharmacology.org/GRAC/LigandDisplayForward?ligandId=4038 (p*K*_i_ 6.9–7.5) [http://www.ncbi.nlm.nih.gov/pubmed/18207395?dopt=AbstractPlus, http://www.ncbi.nlm.nih.gov/pubmed/14643355?dopt=AbstractPlus]
Labelled ligands	[http://www.guidetopharmacology.org/GRAC/LigandDisplayForward?ligandId=1706 http://www.guidetopharmacology.org/GRAC/LigandDisplayForward?ligandId=1706 (Antagonist) (p*K*_d_ 8.3–9.1) [http://www.ncbi.nlm.nih.gov/pubmed/14691055?dopt=AbstractPlus, http://www.ncbi.nlm.nih.gov/pubmed/19542319?dopt=AbstractPlus, http://www.ncbi.nlm.nih.gov/pubmed/20404073?dopt=AbstractPlus], [http://www.guidetopharmacology.org/GRAC/LigandDisplayForward?ligandId=9465 http://www.guidetopharmacology.org/GRAC/LigandDisplayForward?ligandId=9465 (Antagonist) (p*K*_d_ 8.6–8.9) [http://www.ncbi.nlm.nih.gov/pubmed/19542319?dopt=AbstractPlus], [http://www.guidetopharmacology.org/GRAC/LigandDisplayForward?ligandId=9464 http://www.guidetopharmacology.org/GRAC/LigandDisplayForward?ligandId=9464 (Agonist) [http://www.ncbi.nlm.nih.gov/pubmed/26582739?dopt=AbstractPlus, http://www.ncbi.nlm.nih.gov/pubmed/25132134?dopt=AbstractPlus, http://www.ncbi.nlm.nih.gov/pubmed/9491897?dopt=AbstractPlus]	[http://www.guidetopharmacology.org/GRAC/LigandDisplayForward?ligandId=9465 http://www.guidetopharmacology.org/GRAC/LigandDisplayForward?ligandId=9465 (Selective Antagonist) (p*K*_d_ 8.9–9.8) [http://www.ncbi.nlm.nih.gov/pubmed/19542319?dopt=AbstractPlus, http://www.ncbi.nlm.nih.gov/pubmed/20404073?dopt=AbstractPlus], [http://www.guidetopharmacology.org/GRAC/LigandDisplayForward?ligandId=9466 http://www.guidetopharmacology.org/GRAC/LigandDisplayForward?ligandId=9466 (Selective Antagonist) (p*K*_d_ 9.2–9.4) [http://www.ncbi.nlm.nih.gov/pubmed/19542319?dopt=AbstractPlus], [http://www.guidetopharmacology.org/GRAC/LigandDisplayForward?ligandId=9467 http://www.guidetopharmacology.org/GRAC/LigandDisplayForward?ligandId=9467 (Selective Antagonist) (p*K*_d_ 8.6–9) [http://www.ncbi.nlm.nih.gov/pubmed/19751316?dopt=AbstractPlus], [http://www.guidetopharmacology.org/GRAC/LigandDisplayForward?ligandId=9464 http://www.guidetopharmacology.org/GRAC/LigandDisplayForward?ligandId=9464 (Agonist) [http://www.ncbi.nlm.nih.gov/pubmed/26582739?dopt=AbstractPlus, http://www.ncbi.nlm.nih.gov/pubmed/25132134?dopt=AbstractPlus, http://www.ncbi.nlm.nih.gov/pubmed/9491897?dopt=AbstractPlus]

### Comments

The primary coupling of orexin receptors to G_q/11_ proteins is rather speculative and based on the strong activation of phospholipase C, though recent studies in recombinant cells also stress the importance of G_q/11_ [http://www.ncbi.nlm.nih.gov/pubmed/27237973?dopt=AbstractPlus]. Coupling of both receptors to G_i/o_ and Gs has also been reported [http://www.ncbi.nlm.nih.gov/pubmed/15687100?dopt=AbstractPlus, http://www.ncbi.nlm.nih.gov/pubmed/23902572?dopt=AbstractPlus, http://www.ncbi.nlm.nih.gov/pubmed/23848055?dopt=AbstractPlus, http://www.ncbi.nlm.nih.gov/pubmed/11600545?dopt=AbstractPlus]. For most native cellular responses observed, the G protein pathway is unknown. The relative potency order of endogenous ligands depends on the cellular signal transduction machinery [http://www.ncbi.nlm.nih.gov/pubmed/23034387?dopt=AbstractPlus]. Similarly, [http://www.guidetopharmacology.org/GRAC/LigandDisplayForward?ligandId=1700, http://www.guidetopharmacology.org/GRAC/LigandDisplayForward?ligandId=1700]http://www.guidetopharmacology.org/GRAC/LigandDisplayForward?ligandId=1700, http://www.guidetopharmacology.org/GRAC/LigandDisplayForward?ligandId=9305 and http://www.guidetopharmacology.org/GRAC/LigandDisplayForward?ligandId=10277 may show variable selectivity for OX_2_ receptors and are also likely to activate OX_1_ receptors [http://www.ncbi.nlm.nih.gov/pubmed/21362456?dopt=AbstractPlus, http://www.ncbi.nlm.nih.gov/pubmed/30194937?dopt=AbstractPlus]. Many antagonists and radioligands are not well‐characterized, and thus the affinities are uncertain. Among radioligands, [http://www.guidetopharmacology.org/GRAC/LigandDisplayForward?ligandId=1706 http://www.guidetopharmacology.org/GRAC/LigandDisplayForward?ligandId=1706, [http://www.guidetopharmacology.org/GRAC/LigandDisplayForward?ligandId=9467 http://www.guidetopharmacology.org/GRAC/LigandDisplayForward?ligandId=9467 and [http://www.guidetopharmacology.org/GRAC/LigandDisplayForward?ligandId=9465 http://www.guidetopharmacology.org/GRAC/LigandDisplayForward?ligandId=9465 are commercially available. [^3^H]‐TCS 1102 (pK_d/OX1_ 8.2, pK_d/OX2_ 9.0) [http://www.ncbi.nlm.nih.gov/pubmed/24376396?dopt=AbstractPlus] and Rhodamine Green‐orexinA [http://www.ncbi.nlm.nih.gov/pubmed/11266181?dopt=AbstractPlus] are also useful radioligand tools. Orexin receptors have been reported to be able to form complexes with each other and some other GPCRs as well as *σ*1 receptors, which might affect the signaling and pharmacology [http://www.ncbi.nlm.nih.gov/pubmed/27909990?dopt=AbstractPlus, http://www.ncbi.nlm.nih.gov/pubmed/25926444?dopt=AbstractPlus]. Loss‐of‐function mutations in the gene encoding the OX_2_ receptor underlie canine hereditary narcolepsy [http://www.ncbi.nlm.nih.gov/pubmed/10458611?dopt=AbstractPlus]. Antagonists of the orexin receptors are the focus of major drug discovery efforts for their potential to treat insomnia and other disorders of wakefulness [http://www.ncbi.nlm.nih.gov/pubmed/26317591?dopt=AbstractPlus], while agonists would likely be useful in human narcolepsy.

### Further reading on Orexin receptors

Baimel C *et al*. (2015) Orexin/hypocretin role in reward: implications for opioid and other addictions. *Br. J. Pharmacol.* **172**: 334‐48 [https://www.ncbi.nlm.nih.gov/pubmed/24641197?dopt=AbstractPlus]

Burdakov D. (2018) Reactive and predictive homeostasis: Roles of orexin/hypocretin neurons. *Neuropharmacology* [https://www.ncbi.nlm.nih.gov/pubmed/30347195?dopt=AbstractPlus]

Kukkonen JP. (2013) Physiology of the orexinergic/hypocretinergic system: a revisit in 2012. *Am. J. Physiol., Cell Physiol.* **304**: C2‐32 [https://www.ncbi.nlm.nih.gov/pubmed/23034387?dopt=AbstractPlus]

Li SB *et al*. (2016) Hypocretins, Neural Systems, Physiology, and Psychiatric Disorders. *Curr Psychiatry Rep* **18**: 7 [https://www.ncbi.nlm.nih.gov/pubmed/26733323?dopt=AbstractPlus]

Mahler SV *et al*. (2014) Motivational activation: a unifying hypothesis of orexin/hypocretin function. *Nat. Neurosci.* **17**: 1298‐303 [https://www.ncbi.nlm.nih.gov/pubmed/25254979?dopt=AbstractPlus]

## http://www.guidetopharmacology.org/GRAC/FamilyDisplayForward?familyId=447

### Overview

**Nomenclature as recommended by NC‐IUPHAR** [http://www.ncbi.nlm.nih.gov/pubmed/23686350?dopt=AbstractPlus].

**Table:** 

Nomenclature	http://www.guidetopharmacology.org/GRAC/ObjectDisplayForward?objectId=162
HGNC, UniProt	https://www.genenames.org/data/gene‐symbol‐report/#!/hgnc_id/HGNC:4531, http://www.uniprot.org/uniprot/Q96P68
Endogenous agonists	http://www.guidetopharmacology.org/GRAC/LigandDisplayForward?ligandId=3636 [http://www.ncbi.nlm.nih.gov/pubmed/15141213?dopt=AbstractPlus, http://www.ncbi.nlm.nih.gov/pubmed/23396314?dopt=AbstractPlus]

### Further reading on Oxoglutarate receptor

Davenport AP *et al*. (2013) International Union of Basic and Clinical Pharmacology. LXXXVIII. G protein‐coupled receptor list: recommendations for new pairings with cognate ligands. *Pharmacol. Rev.* **65**: 967‐86 [https://www.ncbi.nlm.nih.gov/pubmed/23686350?dopt=AbstractPlus]

Grimm PR and Welling PA (2017) alpha‐Ketoglutarate drives electroneutral NaCl reabsorption in intercalated cells by activating a G‐protein coupled receptor. *Oxgr1. Curr. Opin. Nephrol. Hypertens.* **26**: 426‐433 [https://www.ncbi.nlm.nih.gov/pubmed/28771454]

## http://www.guidetopharmacology.org/GRAC/FamilyDisplayForward?familyId=52

### Overview

P2Y receptors (**nomenclature as agreed by the NC‐IUPHAR Subcommittee on P2Y Receptors** [http://www.ncbi.nlm.nih.gov/pubmed/12559763?dopt=AbstractPlus, http://www.ncbi.nlm.nih.gov/pubmed/16968944?dopt=AbstractPlus]) are activated by the endogenous ligands http://www.guidetopharmacology.org/GRAC/LigandDisplayForward?ligandId=1713, http://www.guidetopharmacology.org/GRAC/LigandDisplayForward?ligandId=1712, http://www.guidetopharmacology.org/GRAC/LigandDisplayForward?ligandId=1734, http://www.guidetopharmacology.org/GRAC/LigandDisplayForward?ligandId=1749 and http://www.guidetopharmacology.org/GRAC/LigandDisplayForward?ligandId=1783. The relationship of many of the cloned receptors to endogenously expressed receptors is not yet established and so it might be appropriate to use wording such as ’http://www.guidetopharmacology.org/GRAC/LigandDisplayForward?ligandId=1734‐preferring (or http://www.guidetopharmacology.org/GRAC/LigandDisplayForward?ligandId=1713‐, *etc*.) P2Y receptor’ or ’P2Y_1_‐like’, *etc.*, until further, as yet undefined, corroborative criteria can be applied [271, http://www.ncbi.nlm.nih.gov/pubmed/21586366?dopt=AbstractPlus, http://www.ncbi.nlm.nih.gov/pubmed/23597047?dopt=AbstractPlus, http://www.ncbi.nlm.nih.gov/pubmed/21586365?dopt=AbstractPlus, http://www.ncbi.nlm.nih.gov/pubmed/22963441?dopt=AbstractPlus]. Clinically used drugs acting on these receptors include the dinucleoside polyphosphate http://www.guidetopharmacology.org/GRAC/LigandDisplayForward?ligandId=1736, agonist of the P2Y_2_ receptor subtype, approved in Japan for the management of dry eye disease [http://www.ncbi.nlm.nih.gov/pubmed/24511227?dopt=AbstractPlus], and the P2Y_12_ receptor antagonists http://www.guidetopharmacology.org/GRAC/LigandDisplayForward?ligandId=7562, http://www.guidetopharmacology.org/GRAC/LigandDisplayForward?ligandId=1765 and http://www.guidetopharmacology.org/GRAC/LigandDisplayForward?ligandId=1776, all approved as antiplatelet drugs [http://www.ncbi.nlm.nih.gov/pubmed/23809135?dopt=AbstractPlus, http://www.ncbi.nlm.nih.gov/pubmed/27886821?dopt=AbstractPlus].

**Table:** 

Nomenclature	http://www.guidetopharmacology.org/GRAC/ObjectDisplayForward?objectId=323	http://www.guidetopharmacology.org/GRAC/ObjectDisplayForward?objectId=324	http://www.guidetopharmacology.org/GRAC/ObjectDisplayForward?objectId=325	http://www.guidetopharmacology.org/GRAC/ObjectDisplayForward?objectId=326
HGNC, UniProt	https://www.genenames.org/data/gene‐symbol‐report/#!/hgnc_id/HGNC:8539, http://www.uniprot.org/uniprot/P47900	https://www.genenames.org/data/gene‐symbol‐report/#!/hgnc_id/HGNC:8541, http://www.uniprot.org/uniprot/P41231	https://www.genenames.org/data/gene‐symbol‐report/#!/hgnc_id/HGNC:8542, http://www.uniprot.org/uniprot/P51582	https://www.genenames.org/data/gene‐symbol‐report/#!/hgnc_id/HGNC:8543, http://www.uniprot.org/uniprot/Q15077
Potency order of endogenous ligands	http://www.guidetopharmacology.org/GRAC/LigandDisplayForward?ligandId=1712 http://www.guidetopharmacology.org/GRAC/LigandDisplayForward?ligandId=1713	http://www.guidetopharmacology.org/GRAC/LigandDisplayForward?ligandId=1734 > http://www.guidetopharmacology.org/GRAC/LigandDisplayForward?ligandId=1713 [http://www.ncbi.nlm.nih.gov/pubmed/8564228?dopt=AbstractPlus]	http://www.guidetopharmacology.org/GRAC/LigandDisplayForward?ligandId=1734 http://www.guidetopharmacology.org/GRAC/LigandDisplayForward?ligandId=1713 (at rat recombinant receptors, UTP = ATP)	http://www.guidetopharmacology.org/GRAC/LigandDisplayForward?ligandId=1749 http://www.guidetopharmacology.org/GRAC/LigandDisplayForward?ligandId=1734 > http://www.guidetopharmacology.org/GRAC/LigandDisplayForward?ligandId=1712
Endogenous agonists	–	http://www.guidetopharmacology.org/GRAC/LigandDisplayForward?ligandId=1734 [http://www.ncbi.nlm.nih.gov/pubmed/11754592?dopt=AbstractPlus, http://www.ncbi.nlm.nih.gov/pubmed/8564228?dopt=AbstractPlus]	–	–
Agonists	http://www.guidetopharmacology.org/GRAC/LigandDisplayForward?ligandId=1755 http://www.guidetopharmacology.org/GRAC/LigandDisplayForward?ligandId=1755 [http://www.ncbi.nlm.nih.gov/pubmed/11502873?dopt=AbstractPlus], http://www.guidetopharmacology.org/GRAC/LigandDisplayForward?ligandId=1710 [http://www.ncbi.nlm.nih.gov/pubmed/9154346?dopt=AbstractPlus, http://www.ncbi.nlm.nih.gov/pubmed/12391289?dopt=AbstractPlus]	–	–	–
Sub/family‐selective agonists	–	http://www.guidetopharmacology.org/GRAC/LigandDisplayForward?ligandId=1736 [http://www.ncbi.nlm.nih.gov/pubmed/11206448?dopt=AbstractPlus], http://www.guidetopharmacology.org/GRAC/LigandDisplayForward?ligandId=1737 [http://www.ncbi.nlm.nih.gov/pubmed/8825364?dopt=AbstractPlus, http://www.ncbi.nlm.nih.gov/pubmed/8825364?dopt=AbstractPlus, http://www.ncbi.nlm.nih.gov/pubmed/12183642?dopt=AbstractPlus], http://www.guidetopharmacology.org/GRAC/LigandDisplayForward?ligandId=1735 http://www.guidetopharmacology.org/GRAC/LigandDisplayForward?ligandId=1735 [http://www.ncbi.nlm.nih.gov/pubmed/8564228?dopt=AbstractPlus]	http://www.guidetopharmacology.org/GRAC/LigandDisplayForward?ligandId=1736 [http://www.ncbi.nlm.nih.gov/pubmed/16475938?dopt=AbstractPlus], http://www.guidetopharmacology.org/GRAC/LigandDisplayForward?ligandId=1737 [http://www.ncbi.nlm.nih.gov/pubmed/12183642?dopt=AbstractPlus], http://www.guidetopharmacology.org/GRAC/LigandDisplayForward?ligandId=1735 http://www.guidetopharmacology.org/GRAC/LigandDisplayForward?ligandId=1735 [http://www.ncbi.nlm.nih.gov/pubmed/8825364?dopt=AbstractPlus]	–
Selective agonists	http://www.guidetopharmacology.org/GRAC/LigandDisplayForward?ligandId=3338 [http://www.ncbi.nlm.nih.gov/pubmed/15345752?dopt=AbstractPlus], http://www.guidetopharmacology.org/GRAC/LigandDisplayForward?ligandId=8447‐http://www.guidetopharmacology.org/GRAC/LigandDisplayForward?ligandId=8447) [http://www.ncbi.nlm.nih.gov/pubmed/23751098?dopt=AbstractPlus]	http://www.guidetopharmacology.org/GRAC/LigandDisplayForward?ligandId=1733 [http://www.ncbi.nlm.nih.gov/pubmed/17302398?dopt=AbstractPlus], http://www.guidetopharmacology.org/GRAC/LigandDisplayForward?ligandId=3398 [http://www.ncbi.nlm.nih.gov/pubmed/17125260?dopt=AbstractPlus], http://www.guidetopharmacology.org/GRAC/LigandDisplayForward?ligandId=5902 (EC_50_ value determined using an IP_3_ functional assay) [http://www.ncbi.nlm.nih.gov/pubmed/17125260?dopt=AbstractPlus, http://www.ncbi.nlm.nih.gov/pubmed/21417463?dopt=AbstractPlus, http://www.ncbi.nlm.nih.gov/pubmed/17088057?dopt=AbstractPlus]	http://www.guidetopharmacology.org/GRAC/LigandDisplayForward?ligandId=4044 [http://www.ncbi.nlm.nih.gov/pubmed/21528910?dopt=AbstractPlus], http://www.guidetopharmacology.org/GRAC/LigandDisplayForward?ligandId=6199], (http://www.guidetopharmacology.org/GRAC/LigandDisplayForward?ligandId=1740 [http://www.ncbi.nlm.nih.gov/pubmed/11754592?dopt=AbstractPlus]	http://www.guidetopharmacology.org/GRAC/LigandDisplayForward?ligandId=5910 http://www.guidetopharmacology.org/GRAC/LigandDisplayForward?ligandId=5910 [http://www.ncbi.nlm.nih.gov/pubmed/22901672?dopt=AbstractPlus, http://www.ncbi.nlm.nih.gov/pubmed/24970757?dopt=AbstractPlus], http://www.guidetopharmacology.org/GRAC/LigandDisplayForward?ligandId=5903 [http://www.ncbi.nlm.nih.gov/pubmed/20446735?dopt=AbstractPlus], http://www.guidetopharmacology.org/GRAC/LigandDisplayForward?ligandId=1747 [http://www.ncbi.nlm.nih.gov/pubmed/16942026?dopt=AbstractPlus]
Antagonists	http://www.guidetopharmacology.org/GRAC/LigandDisplayForward?ligandId=1728 (p*K*_i_ 5.3) [http://www.ncbi.nlm.nih.gov/pubmed/12391289?dopt=AbstractPlus], http://www.guidetopharmacology.org/GRAC/LigandDisplayForward?ligandId=1725 (p*K*i 5.2) [http://www.ncbi.nlm.nih.gov/pubmed/12391289?dopt=AbstractPlus]	–	–	–
Sub/family‐selective antagonists	–	http://www.guidetopharmacology.org/GRAC/LigandDisplayForward?ligandId=1739 (pIC_50_ 6) [http://www.ncbi.nlm.nih.gov/pubmed/10401562?dopt=AbstractPlus], http://www.guidetopharmacology.org/GRAC/LigandDisplayForward?ligandId=1728 (pIC_50_ 4.3) [http://www.ncbi.nlm.nih.gov/pubmed/10401562?dopt=AbstractPlus, http://www.ncbi.nlm.nih.gov/pubmed/9154346?dopt=AbstractPlus]	http://www.guidetopharmacology.org/GRAC/LigandDisplayForward?ligandId=1725 (pEC_50_ 2–5) [http://www.ncbi.nlm.nih.gov/pubmed/18600475?dopt=AbstractPlus], http://www.guidetopharmacology.org/GRAC/LigandDisplayForward?ligandId=1739 (pIC_50_ 4.7) [http://www.ncbi.nlm.nih.gov/pubmed/9647463?dopt=AbstractPlus] – Rat	http://www.guidetopharmacology.org/GRAC/LigandDisplayForward?ligandId=1739 (p*K*_B_ 6) [http://www.ncbi.nlm.nih.gov/pubmed/26519900?dopt=AbstractPlus], http://www.guidetopharmacology.org/GRAC/LigandDisplayForward?ligandId=1725 (p*K*_B_ 4) [http://www.ncbi.nlm.nih.gov/pubmed/26519900?dopt=AbstractPlus], http://www.guidetopharmacology.org/GRAC/LigandDisplayForward?ligandId=1728 (p*K*_B_ 4) [http://www.ncbi.nlm.nih.gov/pubmed/26519900?dopt=AbstractPlus]
Selective antagonists	http://www.guidetopharmacology.org/GRAC/LigandDisplayForward?ligandId=1724 (p*K*_i_ 8.8–9.1) [http://www.ncbi.nlm.nih.gov/pubmed/15476670?dopt=AbstractPlus, http://www.ncbi.nlm.nih.gov/pubmed/14584948?dopt=AbstractPlus], http://www.guidetopharmacology.org/GRAC/LigandDisplayForward?ligandId=1721 (p*K*_i_ 7.9) [http://www.ncbi.nlm.nih.gov/pubmed/12391289?dopt=AbstractPlus], http://www.guidetopharmacology.org/GRAC/LigandDisplayForward?ligandId=1720 (p*K*_i_ 7–7.1) [http://www.ncbi.nlm.nih.gov/pubmed/8913364?dopt=AbstractPlus]	http://www.guidetopharmacology.org/GRAC/LigandDisplayForward?ligandId=5907 (pIC_50_ ∼6) [http://www.ncbi.nlm.nih.gov/pubmed/15231488?dopt=AbstractPlus], http://www.guidetopharmacology.org/GRAC/LigandDisplayForward?ligandId=1738 (pEC_50_ 6) [http://www.ncbi.nlm.nih.gov/pubmed/17302398?dopt=AbstractPlus], http://www.guidetopharmacology.org/GRAC/LigandDisplayForward?ligandId=9468 (pIC_50_ 4.7) [http://www.ncbi.nlm.nih.gov/pubmed/19419204?dopt=AbstractPlus]	http://www.guidetopharmacology.org/GRAC/LigandDisplayForward?ligandId=1713 (p*K*_d_ 6.2) [http://www.ncbi.nlm.nih.gov/pubmed/10779375?dopt=AbstractPlus]	http://www.guidetopharmacology.org/GRAC/LigandDisplayForward?ligandId=1753 (pIC_50_ 7.4) [http://www.ncbi.nlm.nih.gov/pubmed/15081875?dopt=AbstractPlus], http://www.guidetopharmacology.org/GRAC/LigandDisplayForward?ligandId=1752 (pIC_50_ 6.9) [http://www.ncbi.nlm.nih.gov/pubmed/15081875?dopt=AbstractPlus]
Selective allosteric modulators	http://www.guidetopharmacology.org/GRAC/LigandDisplayForward?ligandId=5808 (Negative) (p*K*_i_ 6.9) [http://www.ncbi.nlm.nih.gov/pubmed/25822790?dopt=AbstractPlus], http://www.guidetopharmacology.org/GRAC/LigandDisplayForward?ligandId=1729 (Negative) (pIC_50_ 6.8) [http://www.ncbi.nlm.nih.gov/pubmed/15193995?dopt=AbstractPlus]	–	–	–
Labelled ligands	[http://www.guidetopharmacology.org/GRAC/LigandDisplayForward?ligandId=1727 http://www.guidetopharmacology.org/GRAC/LigandDisplayForward?ligandId=1727 (Antagonist) (p*K*_d_ 8.1) [http://www.ncbi.nlm.nih.gov/pubmed/12391289?dopt=AbstractPlus], [http://www.guidetopharmacology.org/GRAC/LigandDisplayForward?ligandId=1763 http://www.guidetopharmacology.org/GRAC/LigandDisplayForward?ligandId=1763 (Agonist) [http://www.ncbi.nlm.nih.gov/pubmed/11502873?dopt=AbstractPlus], [http://www.guidetopharmacology.org/GRAC/LigandDisplayForward?ligandId=3408 http://www.guidetopharmacology.org/GRAC/LigandDisplayForward?ligandId=3408 http://www.guidetopharmacology.org/GRAC/LigandDisplayForward?ligandId=3408 (Agonist)	–	–	http://www.guidetopharmacology.org/GRAC/LigandDisplayForward?ligandId=9469 (Selective Antagonist) (pEC_50_ 7.6) [http://www.ncbi.nlm.nih.gov/pubmed/24712832?dopt=AbstractPlus]

**Table:** 

Nomenclature	http://www.guidetopharmacology.org/GRAC/ObjectDisplayForward?objectId=327	http://www.guidetopharmacology.org/GRAC/ObjectDisplayForward?objectId=328	http://www.guidetopharmacology.org/GRAC/ObjectDisplayForward?objectId=329	http://www.guidetopharmacology.org/GRAC/ObjectDisplayForward?objectId=330
HGNC, UniProt	https://www.genenames.org/data/gene‐symbol‐report/#!/hgnc_id/HGNC:8540, http://www.uniprot.org/uniprot/Q96G91	https://www.genenames.org/data/gene‐symbol‐report/#!/hgnc_id/HGNC:18124, http://www.uniprot.org/uniprot/Q9H244	https://www.genenames.org/data/gene‐symbol‐report/#!/hgnc_id/HGNC:4537, http://www.uniprot.org/uniprot/Q9BPV8	https://www.genenames.org/data/gene‐symbol‐report/#!/hgnc_id/HGNC:16442, http://www.uniprot.org/uniprot/Q15391
Potency order of endogenous ligands	http://www.guidetopharmacology.org/GRAC/LigandDisplayForward?ligandId=1713 http://www.guidetopharmacology.org/GRAC/LigandDisplayForward?ligandId=1712	http://www.guidetopharmacology.org/GRAC/LigandDisplayForward?ligandId=1712 http://www.guidetopharmacology.org/GRAC/LigandDisplayForward?ligandId=1713	http://www.guidetopharmacology.org/GRAC/LigandDisplayForward?ligandId=1712 http://www.guidetopharmacology.org/GRAC/LigandDisplayForward?ligandId=1713	http://www.guidetopharmacology.org/GRAC/LigandDisplayForward?ligandId=1749 http://www.guidetopharmacology.org/GRAC/LigandDisplayForward?ligandId=1783 [http://www.ncbi.nlm.nih.gov/pubmed/19759354?dopt=AbstractPlus]
Endogenous agonists	–	http://www.guidetopharmacology.org/GRAC/LigandDisplayForward?ligandId=1712 [http://www.ncbi.nlm.nih.gov/pubmed/15199474?dopt=AbstractPlus]	–	–
Sub/family‐selective agonists	–	http://www.guidetopharmacology.org/GRAC/LigandDisplayForward?ligandId=1710 [http://www.ncbi.nlm.nih.gov/pubmed/15199474?dopt=AbstractPlus], http://www.guidetopharmacology.org/GRAC/LigandDisplayForward?ligandId=1755 http://www.guidetopharmacology.org/GRAC/LigandDisplayForward?ligandId=1755 [http://www.ncbi.nlm.nih.gov/pubmed/11502873?dopt=AbstractPlus]	http://www.guidetopharmacology.org/GRAC/LigandDisplayForward?ligandId=1710 [http://www.ncbi.nlm.nih.gov/pubmed/12815166?dopt=AbstractPlus], http://www.guidetopharmacology.org/GRAC/LigandDisplayForward?ligandId=1711], http://www.guidetopharmacology.org/GRAC/LigandDisplayForward?ligandId=1755]	–
Selective agonists	http://www.guidetopharmacology.org/GRAC/LigandDisplayForward?ligandId=1756 [http://www.ncbi.nlm.nih.gov/pubmed/15893764?dopt=AbstractPlus, http://www.ncbi.nlm.nih.gov/pubmed/10578132?dopt=AbstractPlus], http://www.guidetopharmacology.org/GRAC/LigandDisplayForward?ligandId=4046 [http://www.ncbi.nlm.nih.gov/pubmed/19815812?dopt=AbstractPlus], http://www.guidetopharmacology.org/GRAC/LigandDisplayForward?ligandId=1714 http://www.guidetopharmacology.org/GRAC/LigandDisplayForward?ligandId=1714 [http://www.ncbi.nlm.nih.gov/pubmed/10578132?dopt=AbstractPlus]	–	–	http://www.guidetopharmacology.org/GRAC/LigandDisplayForward?ligandId=6203.http://www.guidetopharmacology.org/GRAC/LigandDisplayForward?ligandId=6203 [http://www.ncbi.nlm.nih.gov/pubmed/19902968?dopt=AbstractPlus], http://www.guidetopharmacology.org/GRAC/LigandDisplayForward?ligandId=5908 [http://www.ncbi.nlm.nih.gov/pubmed/21484092?dopt=AbstractPlus], http://www.guidetopharmacology.org/GRAC/LigandDisplayForward?ligandId=6202]
Antagonists	–	http://www.guidetopharmacology.org/GRAC/LigandDisplayForward?ligandId=1776 (pIC_50_ 9.4) [http://www.ncbi.nlm.nih.gov/pubmed/12213051?dopt=AbstractPlus], http://www.guidetopharmacology.org/GRAC/LigandDisplayForward?ligandId=1732 http://www.guidetopharmacology.org/GRAC/LigandDisplayForward?ligandId=1732 (pIC_50_ 6) [http://www.ncbi.nlm.nih.gov/pubmed/12815166?dopt=AbstractPlus], http://www.guidetopharmacology.org/GRAC/LigandDisplayForward?ligandId=1764 (pIC_50_ 5.4) [http://www.ncbi.nlm.nih.gov/pubmed/11502873?dopt=AbstractPlus]	http://www.guidetopharmacology.org/GRAC/LigandDisplayForward?ligandId=1776 (pIC_50_ 8.3) [http://www.ncbi.nlm.nih.gov/pubmed/12815166?dopt=AbstractPlus], http://www.guidetopharmacology.org/GRAC/LigandDisplayForward?ligandId=1732 http://www.guidetopharmacology.org/GRAC/LigandDisplayForward?ligandId=1732 (pIC_50_ 6.7) [http://www.ncbi.nlm.nih.gov/pubmed/12815166?dopt=AbstractPlus], http://www.guidetopharmacology.org/GRAC/LigandDisplayForward?ligandId=1764 (pIC_50_ 5.6) [http://www.ncbi.nlm.nih.gov/pubmed/12815166?dopt=AbstractPlus]	–
Sub/family‐selective antagonists	http://www.guidetopharmacology.org/GRAC/LigandDisplayForward?ligandId=1728 (pIC_50_ 4.8–6) [http://www.ncbi.nlm.nih.gov/pubmed/10578132?dopt=AbstractPlus], http://www.guidetopharmacology.org/GRAC/LigandDisplayForward?ligandId=1739 (pIC_50_ 5) [http://www.ncbi.nlm.nih.gov/pubmed/10578132?dopt=AbstractPlus]	–	–	–
Selective antagonists	http://www.guidetopharmacology.org/GRAC/LigandDisplayForward?ligandId=1761 (p*K*_i_ 7.3) [http://www.ncbi.nlm.nih.gov/pubmed/16250663?dopt=AbstractPlus], http://www.guidetopharmacology.org/GRAC/LigandDisplayForward?ligandId=1762 (pIC_50_ 6.4–7.1) [http://www.ncbi.nlm.nih.gov/pubmed/19815812?dopt=AbstractPlus]	http://www.guidetopharmacology.org/GRAC/LigandDisplayForward?ligandId=7360 (p*K*_i_ 8) [http://www.ncbi.nlm.nih.gov/pubmed/24215345?dopt=AbstractPlus, http://www.ncbi.nlm.nih.gov/pubmed/24670650?dopt=AbstractPlus], http://www.guidetopharmacology.org/GRAC/LigandDisplayForward?ligandId=3384 (pIC_50_ 7.9) [http://www.ncbi.nlm.nih.gov/pubmed/7582510?dopt=AbstractPlus, http://www.ncbi.nlm.nih.gov/pubmed/7858849?dopt=AbstractPlus], http://www.guidetopharmacology.org/GRAC/LigandDisplayForward?ligandId=1765 (p*K*i 7.8) [http://www.ncbi.nlm.nih.gov/pubmed/22984835?dopt=AbstractPlus]	http://www.guidetopharmacology.org/GRAC/LigandDisplayForward?ligandId=1778 (pIC_50_ 6.2) [http://www.ncbi.nlm.nih.gov/pubmed/15913566?dopt=AbstractPlus], http://www.guidetopharmacology.org/GRAC/LigandDisplayForward?ligandId=1777 (pIC_50_ 6) [http://www.ncbi.nlm.nih.gov/pubmed/15913566?dopt=AbstractPlus]	http://www.guidetopharmacology.org/GRAC/LigandDisplayForward?ligandId=5802 (p*K*_i_ 10.1) [http://www.ncbi.nlm.nih.gov/pubmed/23592514?dopt=AbstractPlus]
Labelled ligands	–	[http://www.guidetopharmacology.org/GRAC/LigandDisplayForward?ligandId=1763 http://www.guidetopharmacology.org/GRAC/LigandDisplayForward?ligandId=1763 (Agonist) [http://www.ncbi.nlm.nih.gov/pubmed/11502873?dopt=AbstractPlus], [http://www.guidetopharmacology.org/GRAC/LigandDisplayForward?ligandId=6147 http://www.guidetopharmacology.org/GRAC/LigandDisplayForward?ligandId=6147 (Antagonist) (p*K*_d_ 8.3–8.5) [http://www.ncbi.nlm.nih.gov/pubmed/16213725?dopt=AbstractPlus, http://www.ncbi.nlm.nih.gov/pubmed/22892887?dopt=AbstractPlus]	[http://www.guidetopharmacology.org/GRAC/LigandDisplayForward?ligandId=1774 http://www.guidetopharmacology.org/GRAC/LigandDisplayForward?ligandId=1774 (Agonist) [http://www.ncbi.nlm.nih.gov/pubmed/12815166?dopt=AbstractPlus]	http://www.guidetopharmacology.org/GRAC/LigandDisplayForward?ligandId=9470 (Selective Antagonist) (p*K*i 10.1) [http://www.ncbi.nlm.nih.gov/pubmed/25299434?dopt=AbstractPlus], http://www.guidetopharmacology.org/GRAC/LigandDisplayForward?ligandId=9471 (Selective Agonist) [http://www.ncbi.nlm.nih.gov/pubmed/26303895?dopt=AbstractPlus]

### Comments

A series of 4‐alkyloxyimino derivatives of uridine5’‐triphosphate which could be useful for derivatization as fluorescent P2Y_2_/4/6 receptor probes has been recently synthesized [http://www.ncbi.nlm.nih.gov/pubmed/24712832?dopt=AbstractPlus].

Single nucleotide polymorphisms of the P2YR_1_ gene have been associated to different platelet reactivity to ADP http://www.guidetopharmacology.org/GRAC/LigandDisplayForward?ligandId=1712 [http://www.ncbi.nlm.nih.gov/pubmed/15514209?dopt=AbstractPlus]. Three frequent nonsynonymous P2Y_2_ receptor polymorphisms have been identified, one of which was significantly more common in cystic fibrosis patients. This polymorphism is linked to increases in Ca^2+^ influx in transfected cells, and might therefore play a role in disease development [http://www.ncbi.nlm.nih.gov/pubmed/16495779?dopt=AbstractPlus]. Although http://www.guidetopharmacology.org/GRAC/LigandDisplayForward?ligandId=1734 (UTP) was also shown to be a biased agonist at P2Y_11_, this is still under debate [http://www.ncbi.nlm.nih.gov/pubmed/25015314?dopt=AbstractPlus, http://www.ncbi.nlm.nih.gov/pubmed/12761346?dopt=AbstractPlus]. A group of single nucleotide polymorphisms in the P2Y_12_ gene, forming the so called P2Y_12_ H2 haplotype, has been associated with increased platelet responsiveness to ADP, increased risk of peripheral arterial disease and with coronary artery disease [http://www.ncbi.nlm.nih.gov/pubmed/17803810?dopt=AbstractPlus]. The platelet‐type bleeding disorder due to P2Y_12_ receptor defects is an autosomal recessive condition characterized by mild to moderate mucocutaneous bleeding and excessive bleeding after surgery or trauma. The defect is due to the inability of ADP to induce platelet aggregation [http://www.ncbi.nlm.nih.gov/pubmed/12578987?dopt=AbstractPlus]. The P2Y_13_ receptor Met‐158‐Thr polymorphism, which is in linkage disequilibrium with the P2Y_12_ locus, is not associated with acute myocardial infarction, diabetes mellitus or related risk factors [http://www.ncbi.nlm.nih.gov/pubmed/18213371?dopt=AbstractPlus]. The P2Y_14_ receptor was previously considered to exclusively bind sugar nucleotides such as http://www.guidetopharmacology.org/GRAC/LigandDisplayForward?ligandId=1783 and http://www.guidetopharmacology.org/GRAC/LigandDisplayForward?ligandId=1782 [http://www.ncbi.nlm.nih.gov/pubmed/10753868?dopt=AbstractPlus]. However, more recent evidence with several cell lines has demonstrated that http://www.guidetopharmacology.org/GRAC/LigandDisplayForward?ligandId=1749 (UDP) is 5‐fold more potent than http://www.guidetopharmacology.org/GRAC/LigandDisplayForward?ligandId=1783 [http://www.ncbi.nlm.nih.gov/pubmed/19759354?dopt=AbstractPlus]. UDP was also shown to competitively antagonise the UDP‐glucose response at the human recombinant P2Y_14_ receptor [http://www.ncbi.nlm.nih.gov/pubmed/18252808?dopt=AbstractPlus].

### Further reading on P2Y receptors

Abbracchio MP *et al*. (2006) International Union of Pharmacology LVIII: update on the P2Y G protein‐coupled nucleotide receptors: frommolecular mechanisms andpathophysiology totherapy. *Pharmacol. Rev.* **58**: 281‐341 [https://www.ncbi.nlm.nih.gov/pubmed/16968944?dopt=AbstractPlus]

Jacobson KA *et al*. (2015) Nucleotides Acting at P2Y Receptors: Connecting Structure and Function. *Mol. Pharmacol.* **88**: 220‐30 [https://www.ncbi.nlm.nih.gov/pubmed/25837834?dopt=AbstractPlus]

von Kügelgen I *et al*. (2016) Pharmacology and structure of P2Y receptors. *Neuropharmacology* **104**: 50‐61 [https://www.ncbi.nlm.nih.gov/pubmed/26519900?dopt=AbstractPlus]

## http://www.guidetopharmacology.org/GRAC/FamilyDisplayForward?familyId=53

### Overview

The parathyroid hormone receptors (**nomenclature as agreed by the NC‐IUPHAR Subcommittee on Parathyroid Hormone Receptors** [http://www.ncbi.nlm.nih.gov/pubmed/25713287?dopt=AbstractPlus]) are family B G protein‐coupled receptors. The parathyroid hormone (PTH)/parathyroid hormone‐related peptide (PTHrP) receptor (PTH1 receptor) is activated by precursor‐derived peptides: http://www.guidetopharmacology.org/GRAC/LigandDisplayForward?ligandId=1785 (https://www.genenames.org/data/gene‐symbol‐report/#!/hgnc_id/HGNC:9606, http://www.uniprot.org/uniprot/P01270) (84 amino acids), and http://www.guidetopharmacology.org/GRAC/LigandDisplayForward?ligandId=3738 (https://www.genenames.org/data/gene‐symbol‐report/#!/hgnc_id/HGNC:9607, http://www.uniprot.org/uniprot/P12272) (141 amino‐acids) and related peptides (PTH‐(1‐34), http://www.guidetopharmacology.org/GRAC/LigandDisplayForward?ligandId=1790, http://www.uniprot.org/uniprot/P12272)). The parathyroid hormone 2 receptor (PTH2 receptor) is activated by the precursor‐derived peptide http://www.guidetopharmacology.org/GRAC/LigandDisplayForward?ligandId=1815 (https://www.genenames.org/data/gene‐symbol‐report/#!/hgnc_id/HGNC:30828, http://www.uniprot.org/uniprot/Q96A98) (39 amino acids). [^125^I]PTH may be used to label both PTH1 and PTH2 receptors.

**Table:** 

Nomenclature	http://www.guidetopharmacology.org/GRAC/ObjectDisplayForward?objectId=331	http://www.guidetopharmacology.org/GRAC/ObjectDisplayForward?objectId=332
HGNC, UniProt	https://www.genenames.org/data/gene‐symbol‐report/#!/hgnc_id/HGNC:9608, http://www.uniprot.org/uniprot/Q03431	https://www.genenames.org/data/gene‐symbol‐report/#!/hgnc_id/HGNC:9609, http://www.uniprot.org/uniprot/P49190
Potency order of endogenous ligands	http://www.guidetopharmacology.org/GRAC/LigandDisplayForward?ligandId=1785 (https://www.genenames.org/data/gene‐symbol‐report/#!/hgnc_id/HGNC:9606, http://www.uniprot.org/uniprot/P01270) = http://www.guidetopharmacology.org/GRAC/LigandDisplayForward?ligandId=3738 (https://www.genenames.org/data/gene‐symbol‐report/#!/hgnc_id/HGNC:9607, http://www.uniprot.org/uniprot/P12272)	http://www.guidetopharmacology.org/GRAC/LigandDisplayForward?ligandId=1815 (https://www.genenames.org/data/gene‐symbol‐report/#!/hgnc_id/HGNC:30828, http://www.uniprot.org/uniprot/Q96A98), http://www.guidetopharmacology.org/GRAC/LigandDisplayForward?ligandId=1785 (https://www.genenames.org/data/gene‐symbol‐report/#!/hgnc_id/HGNC:9606, http://www.uniprot.org/uniprot/P01270) ≫ http://www.guidetopharmacology.org/GRAC/LigandDisplayForward?ligandId=3738 (https://www.genenames.org/data/gene‐symbol‐report/#!/hgnc_id/HGNC:9607, http://www.uniprot.org/uniprot/P12272)
Agonists	http://www.guidetopharmacology.org/GRAC/LigandDisplayForward?ligandId=4448 [http://www.ncbi.nlm.nih.gov/pubmed/8702701?dopt=AbstractPlus]	http://www.guidetopharmacology.org/GRAC/LigandDisplayForward?ligandId=1815 (https://www.genenames.org/data/gene‐symbol‐report/#!/hgnc_id/HGNC:30828, http://www.uniprot.org/uniprot/Q96A98) [http://www.ncbi.nlm.nih.gov/pubmed/11602681?dopt=AbstractPlus, http://www.ncbi.nlm.nih.gov/pubmed/10854439?dopt=AbstractPlus]
Selective agonists	http://www.guidetopharmacology.org/GRAC/LigandDisplayForward?ligandId=1822) [http://www.ncbi.nlm.nih.gov/pubmed/7896796?dopt=AbstractPlus] – Rat	–

### Comments

The parathyroid hormone type 1 receptor (PTHR) is the canonical GPCR for PTH and PTHrP. It is coupled to Gs and Gq and regulates the development of bone, heart, mammary glands and other tissues in response to PTHrP, and blood concentrations of calcium and phosphate ions, as well as vitamin D, in response to PTH. Another important action of the PTH/PTHR system is to stimulate bone formation when the hormone is intermittently administrated (daily injection).

Although http://www.guidetopharmacology.org/GRAC/LigandDisplayForward?ligandId=1785 (https://www.genenames.org/data/gene‐symbol‐report/#!/hgnc_id/HGNC:9606, http://www.uniprot.org/uniprot/P01270) is an agonist at human PTH2 receptors, it fails to activate the rodent orthologues. http://www.guidetopharmacology.org/GRAC/LigandDisplayForward?ligandId=1815 (https://www.genenames.org/data/gene‐symbol‐report/#!/hgnc_id/HGNC:30828, http://www.uniprot.org/uniprot/Q96A98) is a weak antagonist at PTH1 receptors [http://www.ncbi.nlm.nih.gov/pubmed/11159842?dopt=AbstractPlus].

### Further reading on Parathyroid hormone receptors

Cheloha RW *et al*. (2015) PTH receptor‐1 signalling‐mechanistic insights and therapeutic prospects. *Nat Rev Endocrinol* **11**: 712‐24 [https://www.ncbi.nlm.nih.gov/pubmed/26303600?dopt=AbstractPlus]

Gardella TJ *et al*. (2015) International Union of Basic and Clinical Pharmacology. XCIII. The Parathyroid Hormone Receptors‐Family B G Protein‐Coupled Receptors. *Pharmacol. Rev.* **67**: 310‐37 [https://www.ncbi.nlm.nih.gov/pubmed/25713287?dopt=AbstractPlus]

Vilardaga JP *et al*. (2014) Endosomal generation of cAMP in GPCR signaling. *Nat. Chem. Biol.* **10**: 700‐6 [https://www.ncbi.nlm.nih.gov/pubmed/25271346?dopt=AbstractPlus]

## http://www.guidetopharmacology.org/GRAC/FamilyDisplayForward?familyId=55

### Overview

Platelet‐activating factor (http://www.guidetopharmacology.org/GRAC/LigandDisplayForward?ligandId=1831, 1‐*O*‐alkyl‐2‐acetyl‐sn‐glycero‐3‐phosphocholine) is an ether phospholipid mediator associated with platelet coagulation, but also subserves inflammatory roles. The PAF receptor (**provisional nomenclature recommended by NC‐IUPHAR** [http://www.ncbi.nlm.nih.gov/pubmed/15914470?dopt=AbstractPlus]) is activated by http://www.guidetopharmacology.org/GRAC/LigandDisplayForward?ligandId=1831 and other suggested endogenous ligands are oxidized phosphatidylcholine [http://www.ncbi.nlm.nih.gov/pubmed/10497200?dopt=AbstractPlus] and http://www.guidetopharmacology.org/GRAC/LigandDisplayForward?ligandId=2508 [http://www.ncbi.nlm.nih.gov/pubmed/9038918?dopt=AbstractPlus]. It may also be activated by bacterial lipopolysaccharide [http://www.ncbi.nlm.nih.gov/pubmed/1333988?dopt=AbstractPlus].

**Table:** 

Nomenclature	http://www.guidetopharmacology.org/GRAC/ObjectDisplayForward?objectId=334
HGNC, UniProt	https://www.genenames.org/data/gene‐symbol‐report/#!/hgnc_id/HGNC:9582, http://www.uniprot.org/uniprot/P25105
Selective agonists	http://www.guidetopharmacology.org/GRAC/LigandDisplayForward?ligandId=3427
Selective antagonists	http://www.guidetopharmacology.org/GRAC/LigandDisplayForward?ligandId=1856 (p*K*_i_ 10.3) [http://www.ncbi.nlm.nih.gov/pubmed/8395255?dopt=AbstractPlus], http://www.guidetopharmacology.org/GRAC/LigandDisplayForward?ligandId=1850 (p*K*_i_ 9.2) [http://www.ncbi.nlm.nih.gov/pubmed/9151941?dopt=AbstractPlus], http://www.guidetopharmacology.org/GRAC/LigandDisplayForward?ligandId=1852 (pIC_50_ 8.1–8.3) [http://www.ncbi.nlm.nih.gov/pubmed/15922596?dopt=AbstractPlus, http://www.ncbi.nlm.nih.gov/pubmed/1657923?dopt=AbstractPlus], http://www.guidetopharmacology.org/GRAC/LigandDisplayForward?ligandId=3426 (p*K*_i_ 7.8) [http://www.ncbi.nlm.nih.gov/pubmed/2841449?dopt=AbstractPlus], http://www.guidetopharmacology.org/GRAC/LigandDisplayForward?ligandId=1860 (p*K*_i_ 5.2–7.5) [http://www.ncbi.nlm.nih.gov/pubmed/8798529?dopt=AbstractPlus, http://www.ncbi.nlm.nih.gov/pubmed/8380690?dopt=AbstractPlus]
Labelled ligands	http://www.guidetopharmacology.org/GRAC/LigandDisplayForward?ligandId=1833 (Agonist) [http://www.ncbi.nlm.nih.gov/pubmed/11560941?dopt=AbstractPlus, http://www.ncbi.nlm.nih.gov/pubmed/1657923?dopt=AbstractPlus]

### Comments

Note that a previously recommended radioligand (http://www.guidetopharmacology.org/GRAC/LigandDisplayForward?ligandId=1859 K_d_ 44.6 nM) is currently unavailable.

### Further reading on Platelet‐activating factor receptor

Foord SM *et al*. (2005) International Union of Pharmacology. XLVI. G protein‐coupled receptor list. *Pharmacol Rev* **57**: 279‐288 [https://www.ncbi.nlm.nih.gov/pubmed/15914470?dopt=AbstractPlus]

Ishii S *et al*. (2000) Platelet‐activating factor (PAF) receptor and genetically engineered PAF receptor mutant mice. *Prog. Lipid Res.* **39**: 41‐82 [https://www.ncbi.nlm.nih.gov/pubmed/10729607?dopt=AbstractPlus]

Prescott SM *et al*. (2000) Platelet‐activating factor and related lipid mediators. *Annu. Rev. Biochem.* **69**: 419‐45 [https://www.ncbi.nlm.nih.gov/pubmed/10966465?dopt=AbstractPlus]

## http://www.guidetopharmacology.org/GRAC/FamilyDisplayForward?familyId=56

### Overview

Prokineticin receptors, PKR_1_ and PKR_2_ (**provisional nomenclature as recommended by NC‐IUPHAR** [http://www.ncbi.nlm.nih.gov/pubmed/15914470?dopt=AbstractPlus]) respond to the cysteine‐rich 81‐86 amino‐acid peptides http://www.guidetopharmacology.org/GRAC/LigandDisplayForward?ligandId=1866 (https://www.genenames.org/data/gene‐symbol‐report/#!/hgnc_id/HGNC:18454, http://www.uniprot.org/uniprot/Q9HC23) (also known as endocrine glandderived vascular endothelial growth factor, mambakine) and http://www.guidetopharmacology.org/GRAC/LigandDisplayForward?ligandId=1867 (https://www.genenames.org/data/gene‐symbol‐report/#!/hgnc_id/HGNC:18455, https://www.genenames.org/data/gene‐symbol‐report/#!/hgnc_id/HGNC:18455) (protein Bv8 homologue). An orthologue of PROK1 from black mamba (*Dendroaspis polylepi*s) venom, mamba intestinal toxin 1 (http://www.guidetopharmacology.org/GRAC/LigandDisplayForward?ligandId=1865, [http://www.ncbi.nlm.nih.gov/pubmed/10567694?dopt=AbstractPlus]) is a potent, nonselective agonist at prokineticin receptors [http://www.ncbi.nlm.nih.gov/pubmed/12054613?dopt=AbstractPlus], while http://www.guidetopharmacology.org/GRAC/LigandDisplayForward?ligandId=5362, an orthologue of PROK2 from amphibians (*Bombina sp.*, [http://www.ncbi.nlm.nih.gov/pubmed/10422759?dopt=AbstractPlus]), is equipotent at recombinant PKR_1_ and PKR_2_ [http://www.ncbi.nlm.nih.gov/pubmed/16113687?dopt=AbstractPlus], and has high potency in macrophagechemotaxis assays, which arelost in PKR_1_null mice.

**Table:** 

Nomenclature	http://www.guidetopharmacology.org/GRAC/ObjectDisplayForward?objectId=335	http://www.guidetopharmacology.org/GRAC/ObjectDisplayForward?objectId=336
HGNC, UniProt	https://www.genenames.org/data/gene‐symbol‐report/#!/hgnc_id/HGNC:4524, http://www.uniprot.org/uniprot/Q8TCW9	https://www.genenames.org/data/gene‐symbol‐report/#!/hgnc_id/HGNC:15836, http://www.uniprot.org/uniprot/Q8NFJ6
Potency order of endogenous ligands	http://www.guidetopharmacology.org/GRAC/LigandDisplayForward?ligandId=1867 (https://www.genenames.org/data/gene‐symbol‐report/#!/hgnc_id/HGNC:18455, http://www.uniprot.org/uniprot/Q9HC23) > http://www.guidetopharmacology.org/GRAC/LigandDisplayForward?ligandId=1866 (https://www.genenames.org/data/gene‐symbol‐report/#!/hgnc_id/HGNC:18454, https://www.genenames.org/data/gene‐symbol‐report/#!/hgnc_id/HGNC:18454) > http://www.guidetopharmacology.org/GRAC/LigandDisplayForward?ligandId=1868 http://www.guidetopharmacology.org/GRAC/LigandDisplayForward?ligandId=1868 (https://www.genenames.org/data/gene‐symbol‐report/#!/hgnc_id/HGNC:18455) [http://www.ncbi.nlm.nih.gov/pubmed/15772293?dopt=AbstractPlus, http://www.ncbi.nlm.nih.gov/pubmed/11886876?dopt=AbstractPlus, http://www.ncbi.nlm.nih.gov/pubmed/12054613?dopt=AbstractPlus, http://www.ncbi.nlm.nih.gov/pubmed/12427552?dopt=AbstractPlus]	http://www.guidetopharmacology.org/GRAC/LigandDisplayForward?ligandId=1867 (https://www.genenames.org/data/gene‐symbol‐report/#!/hgnc_id/HGNC:18455, http://www.uniprot.org/uniprot/Q9HC23) > http://www.guidetopharmacology.org/GRAC/LigandDisplayForward?ligandId=1866 (https://www.genenames.org/data/gene‐symbol‐report/#!/hgnc_id/HGNC:18454, https://www.genenames.org/data/gene‐symbol‐report/#!/hgnc_id/HGNC:18454) > http://www.guidetopharmacology.org/GRAC/LigandDisplayForward?ligandId=1868 http://www.guidetopharmacology.org/GRAC/LigandDisplayForward?ligandId=1868 (https://www.genenames.org/data/gene‐symbol‐report/#!/hgnc_id/HGNC:18455) [http://www.ncbi.nlm.nih.gov/pubmed/15772293?dopt=AbstractPlus, http://www.ncbi.nlm.nih.gov/pubmed/11886876?dopt=AbstractPlus, http://www.ncbi.nlm.nih.gov/pubmed/12054613?dopt=AbstractPlus, http://www.ncbi.nlm.nih.gov/pubmed/12427552?dopt=AbstractPlus]
Agonists	http://www.guidetopharmacology.org/GRAC/LigandDisplayForward?ligandId=1865 [http://www.ncbi.nlm.nih.gov/pubmed/12054613?dopt=AbstractPlus]	http://www.guidetopharmacology.org/GRAC/LigandDisplayForward?ligandId=1865 [http://www.ncbi.nlm.nih.gov/pubmed/12054613?dopt=AbstractPlus]
Selective agonists	http://www.guidetopharmacology.org/GRAC/LigandDisplayForward?ligandId=8518 [http://www.ncbi.nlm.nih.gov/pubmed/25831128?dopt=AbstractPlus], http://www.guidetopharmacology.org/GRAC/LigandDisplayForward?ligandId=8517 [http://www.ncbi.nlm.nih.gov/pubmed/25831128?dopt=AbstractPlus]	–
Labelled ligands	http://www.guidetopharmacology.org/GRAC/LigandDisplayForward?ligandId=8522 (Agonist) [http://www.ncbi.nlm.nih.gov/pubmed/12054613?dopt=AbstractPlus]	http://www.guidetopharmacology.org/GRAC/LigandDisplayForward?ligandId=8522 (Agonist) [http://www.ncbi.nlm.nih.gov/pubmed/12054613?dopt=AbstractPlus]

### Comments

Genetic mutations in *PROKR1* are associated with Hirschsprung's disease [http://www.ncbi.nlm.nih.gov/pubmed/21858136?dopt=AbstractPlus], while genetic mutations in *PROKR2* are associated with hypogonadotropic hypogonadism with anosmia [http://www.ncbi.nlm.nih.gov/pubmed/23596439?dopt=AbstractPlus], hypopituitarism with pituitary stalk interruption [http://www.ncbi.nlm.nih.gov/pubmed/22466334?dopt=AbstractPlus] and Hirschsprung's disease [http://www.ncbi.nlm.nih.gov/pubmed/21858136?dopt=AbstractPlus]. PKR_2_ has been recently identified as a receptor for *T. cruzi* natural infection [http://www.ncbi.nlm.nih.gov/pubmed/25324134?dopt=AbstractPlus].

### Further reading on Prokineticin receptors

Boulberdaa M *et al*. (2011) Prokineticin receptor 1 (PKR1) signalling in cardiovascular and kidney functions. *Cardiovasc. Res.* **92**: 191‐8 [https://www.ncbi.nlm.nih.gov/pubmed/21856786?dopt=AbstractPlus]

Negri L *et al*. (2018) The Prokineticins: Neuromodulators and Mediators of Inflammation and Myeloid Cell‐Dependent Angiogenesis. *Physiol. Rev.* **98**: 1055‐1082 [https://www.ncbi.nlm.nih.gov/pubmed/29537336?dopt=AbstractPlus]

Negri L *et al*. (2012) Bv8/PK2 and prokineticin receptors: a druggable pronociceptive system. *Curr Opin Pharmacol* **12**: 62‐6 [https://www.ncbi.nlm.nih.gov/pubmed/22136937?dopt=AbstractPlus]

Negri L *et al*. (2007) Bv8/Prokineticin proteins and their receptors. *Life Sci.* **81**: 1103‐16 [https://www.ncbi.nlm.nih.gov/pubmed/17881008?dopt=AbstractPlus]

Ngan ES *et al*. (2008) Prokineticin‐signaling pathway. *Int. J. Biochem. Cell Biol.* **40**: 1679‐84 [https://www.ncbi.nlm.nih.gov/pubmed/18440852?dopt=AbstractPlus]

## http://www.guidetopharmacology.org/GRAC/FamilyDisplayForward?familyId=57

### Overview

The precursor (https://www.genenames.org/data/gene‐symbol‐report/#!/hgnc_id/HGNC:17945, http://www.uniprot.org/uniprot/P81277) for PrRP generates 31 and 20‐amino‐acid versions. http://www.guidetopharmacology.org/GRAC/LigandDisplayForward?ligandId=3665 (https://www.genenames.org/data/gene‐symbol‐report/#!/hgnc_id/HGNC:29982, http://www.uniprot.org/uniprot/P83859) (named after a pyroglutamylated arginine‐phenylalanine‐amide peptide) is a 43 amino acid peptide derived from https://www.genenames.org/data/gene‐symbol‐report/#!/hgnc_id/HGNC:29982 (http://www.uniprot.org/uniprot/P83859) and is also known as P518 or 26RFa. RFRP is an RF amide‐related peptide [http://www.ncbi.nlm.nih.gov/pubmed/11025660?dopt=AbstractPlus] derived from a FMRFamide‐related peptide precursor (https://www.genenames.org/data/gene‐symbol‐report/#!/hgnc_id/HGNC:13782, http://www.uniprot.org/uniprot/Q9HCQ7), which is cleaved to generate http://www.guidetopharmacology.org/GRAC/LigandDisplayForward?ligandId=3736 (https://www.genenames.org/data/gene‐symbol‐report/#!/hgnc_id/HGNC:7901, http://www.uniprot.org/uniprot/O15130), neuropeptide http://www.guidetopharmacology.org/GRAC/LigandDisplayForward?ligandId=5340 (https://www.genenames.org/data/gene‐symbol‐report/#!/hgnc_id/HGNC:13782, http://www.uniprot.org/uniprot/Q9HCQ7), neuropeptide http://www.guidetopharmacology.org/GRAC/LigandDisplayForward?ligandId=5373 (https://www.genenames.org/data/gene‐symbol‐report/#!/hgnc_id/HGNC:13782, http://www.uniprot.org/uniprot/Q9HCQ7) and neuropeptide http://www.guidetopharmacology.org/GRAC/LigandDisplayForward?ligandId=4016 (https://www.genenames.org/data/gene‐symbol‐report/#!/hgnc_id/HGNC:13782, http://www.uniprot.org/uniprot/Q9HCQ7) (neuropeptide NPVF).

**Table:** 

Nomenclature	http://www.guidetopharmacology.org/GRAC/ObjectDisplayForward?objectId=337
HGNC, UniProt	https://www.genenames.org/data/gene‐symbol‐report/#!/hgnc_id/HGNC:4464, http://www.uniprot.org/uniprot/P49683
Potency order of endogenous ligands	http://www.guidetopharmacology.org/GRAC/LigandDisplayForward?ligandId=1871 (https://www.genenames.org/data/gene‐symbol‐report/#!/hgnc_id/HGNC:17945, http://www.uniprot.org/uniprot/P81277) = http://www.guidetopharmacology.org/GRAC/LigandDisplayForward?ligandId=1873 (https://www.genenames.org/data/gene‐symbol‐report/#!/hgnc_id/HGNC:17945, http://www.uniprot.org/uniprot/P81277) [http://www.ncbi.nlm.nih.gov/pubmed/11030716?dopt=AbstractPlus]
Endogenous agonists	http://www.guidetopharmacology.org/GRAC/LigandDisplayForward?ligandId=1871 (https://www.genenames.org/data/gene‐symbol‐report/#!/hgnc_id/HGNC:17945, http://www.uniprot.org/uniprot/P81277) [http://www.ncbi.nlm.nih.gov/pubmed/12606605?dopt=AbstractPlus, http://www.ncbi.nlm.nih.gov/pubmed/11030716?dopt=AbstractPlus], http://www.guidetopharmacology.org/GRAC/LigandDisplayForward?ligandId=1873 (https://www.genenames.org/data/gene‐symbol‐report/#!/hgnc_id/HGNC:17945, http://www.uniprot.org/uniprot/P81277) [http://www.ncbi.nlm.nih.gov/pubmed/12606605?dopt=AbstractPlus, http://www.ncbi.nlm.nih.gov/pubmed/11030716?dopt=AbstractPlus]
Endogenous antagonists	http://www.guidetopharmacology.org/GRAC/LigandDisplayForward?ligandId=1504 (https://www.genenames.org/data/gene‐symbol‐report/#!/hgnc_id/HGNC:7955, http://www.uniprot.org/uniprot/P01303) (p*K*_i_ 5.4) [http://www.ncbi.nlm.nih.gov/pubmed/15885496?dopt=AbstractPlus]
Labelled ligands	http://www.guidetopharmacology.org/GRAC/LigandDisplayForward?ligandId=1875 (Agonist) [http://www.ncbi.nlm.nih.gov/pubmed/11030716?dopt=AbstractPlus], http://www.guidetopharmacology.org/GRAC/LigandDisplayForward?ligandId=4022 (Agonist) [http://www.ncbi.nlm.nih.gov/pubmed/15752583?dopt=AbstractPlus]

### Comments

The orphan receptor https://www.genenames.org/data/gene‐symbol‐report/#!/hgnc_id/HGNC:4523 (http://www.uniprot.org/uniprot/Q9NYM4) shows sequence similarities with NPFF1, NPFF2, PrRP and QRFP receptors.

### Further reading on Prolactin‐releasing peptide receptor

Samson WK *et al*. (2006) Prolactin releasing peptide (PrRP): an endogenous regulator of cell growth. *Peptides* **27**: 1099‐103 [https://www.ncbi.nlm.nih.gov/pubmed/16500730?dopt=AbstractPlus]

Takayanagi Y *et al*. (2010) Roles of prolactin‐releasing peptide and RFamide related peptides in the control of stress and food intake. *FEBS J.* **277**: 4998‐5005 [https://www.ncbi.nlm.nih.gov/pubmed/21126313?dopt=AbstractPlus]

## http://www.guidetopharmacology.org/GRAC/FamilyDisplayForward?familyId=58

### Overview

Prostanoid receptors (**nomenclature as agreed by the NC‐IUPHAR Subcommittee on Prostanoid Receptors** [http://www.ncbi.nlm.nih.gov/pubmed/21752876?dopt=AbstractPlus]) are activated by the endogenous ligands prostaglandins http://www.guidetopharmacology.org/GRAC/LigandDisplayForward?ligandId=1881, http://www.guidetopharmacology.org/GRAC/LigandDisplayForward?ligandId=1882, http://www.guidetopharmacology.org/GRAC/LigandDisplayForward?ligandId=1883, http://www.guidetopharmacology.org/GRAC/LigandDisplayForward?ligandId=1884, http://www.guidetopharmacology.org/GRAC/LigandDisplayForward?ligandId=4483, prostacyclin [http://www.guidetopharmacology.org/GRAC/LigandDisplayForward?ligandId=1915] and http://www.guidetopharmacology.org/GRAC/LigandDisplayForward?ligandId=4482. Measurement of the potency of http://www.guidetopharmacology.org/GRAC/LigandDisplayForward?ligandId=1915 and http://www.guidetopharmacology.org/GRAC/LigandDisplayForward?ligandId=4482 is hampered by their instability in physiological salt solution; they are often replaced by http://www.guidetopharmacology.org/GRAC/LigandDisplayForward?ligandId=1917 and http://www.guidetopharmacology.org/GRAC/LigandDisplayForward?ligandId=1888, respectively, in receptor characterization studies.

**Table:** 

Nomenclature	http://www.guidetopharmacology.org/GRAC/ObjectDisplayForward?objectId=338	http://www.guidetopharmacology.org/GRAC/ObjectDisplayForward?objectId=339
HGNC, UniProt	https://www.genenames.org/data/gene‐symbol‐report/#!/hgnc_id/HGNC:9591, http://www.uniprot.org/uniprot/Q13258	https://www.genenames.org/data/gene‐symbol‐report/#!/hgnc_id/HGNC:4502, http://www.uniprot.org/uniprot/Q9Y5Y4
Potency order of endogenous ligands	http://www.guidetopharmacology.org/GRAC/LigandDisplayForward?ligandId=1881 > http://www.guidetopharmacology.org/GRAC/LigandDisplayForward?ligandId=1882 http://www.guidetopharmacology.org/GRAC/LigandDisplayForward?ligandId=1883 ≫ http://www.guidetopharmacology.org/GRAC/LigandDisplayForward?ligandId=1884 > http://www.guidetopharmacology.org/GRAC/LigandDisplayForward?ligandId=1915, http://www.guidetopharmacology.org/GRAC/LigandDisplayForward?ligandId=4482	http://www.guidetopharmacology.org/GRAC/LigandDisplayForward?ligandId=1881 ≫ http://www.guidetopharmacology.org/GRAC/LigandDisplayForward?ligandId=1884, http://www.guidetopharmacology.org/GRAC/LigandDisplayForward?ligandId=1883 > http://www.guidetopharmacology.org/GRAC/LigandDisplayForward?ligandId=1915, http://www.guidetopharmacology.org/GRAC/LigandDisplayForward?ligandId=4482
Agonists	http://www.guidetopharmacology.org/GRAC/LigandDisplayForward?ligandId=5820 [http://www.ncbi.nlm.nih.gov/pubmed/25542069?dopt=AbstractPlus, http://www.ncbi.nlm.nih.gov/pubmed/22480736?dopt=AbstractPlus]	–
Selective agonists	http://www.guidetopharmacology.org/GRAC/LigandDisplayForward?ligandId=1878 [http://www.ncbi.nlm.nih.gov/pubmed/7642548?dopt=AbstractPlus, http://www.ncbi.nlm.nih.gov/pubmed/9579725?dopt=AbstractPlus, http://www.ncbi.nlm.nih.gov/pubmed/10448933?dopt=AbstractPlus], http://www.guidetopharmacology.org/GRAC/LigandDisplayForward?ligandId=1879 [http://www.ncbi.nlm.nih.gov/pubmed/9579725?dopt=AbstractPlus, http://www.ncbi.nlm.nih.gov/pubmed/10448933?dopt=AbstractPlus]	http://www.guidetopharmacology.org/GRAC/LigandDisplayForward?ligandId=1902 [http://www.ncbi.nlm.nih.gov/pubmed/12721327?dopt=AbstractPlus, http://www.ncbi.nlm.nih.gov/pubmed/12490611?dopt=AbstractPlus, http://www.ncbi.nlm.nih.gov/pubmed/16256979?dopt=AbstractPlus]
Antagonists	–	http://www.guidetopharmacology.org/GRAC/LigandDisplayForward?ligandId=8995 (p*K*_d_ 9) [2072, http://www.ncbi.nlm.nih.gov/pubmed/26916831?dopt=AbstractPlus], http://www.guidetopharmacology.org/GRAC/LigandDisplayForward?ligandId=1911 (p*K*_i_ 7.4) [http://www.ncbi.nlm.nih.gov/pubmed/16256979?dopt=AbstractPlus]
Selective antagonists	http://www.guidetopharmacology.org/GRAC/LigandDisplayForward?ligandId=3356 (p*K*_i_ 10.1) [http://www.ncbi.nlm.nih.gov/pubmed/17300164?dopt=AbstractPlus], http://www.guidetopharmacology.org/GRAC/LigandDisplayForward?ligandId=1897 (p*K*_i_ 8.6–9.3) [http://www.ncbi.nlm.nih.gov/pubmed/7642548?dopt=AbstractPlus, http://www.ncbi.nlm.nih.gov/pubmed/2924081?dopt=AbstractPlus, http://www.ncbi.nlm.nih.gov/pubmed/9579725?dopt=AbstractPlus], http://www.guidetopharmacology.org/GRAC/LigandDisplayForward?ligandId=8537 (p*K*_i_ 7.7) [http://www.ncbi.nlm.nih.gov/pubmed/21819041?dopt=AbstractPlus, http://www.ncbi.nlm.nih.gov/pubmed/15388164?dopt=AbstractPlus, http://www.ncbi.nlm.nih.gov/pubmed/18178816?dopt=AbstractPlus]	http://www.guidetopharmacology.org/GRAC/LigandDisplayForward?ligandId=1905 (pIC_50_ 8.9) [http://www.ncbi.nlm.nih.gov/pubmed/17714552?dopt=AbstractPlus, http://www.ncbi.nlm.nih.gov/pubmed/15715457?dopt=AbstractPlus]
Labelled ligands	http://www.guidetopharmacology.org/GRAC/LigandDisplayForward?ligandId=1891 (Agonist) [http://www.ncbi.nlm.nih.gov/pubmed/16604093?dopt=AbstractPlus, http://www.ncbi.nlm.nih.gov/pubmed/9579725?dopt=AbstractPlus]	http://www.guidetopharmacology.org/GRAC/LigandDisplayForward?ligandId=1891 (Agonist) [http://www.ncbi.nlm.nih.gov/pubmed/16418339?dopt=AbstractPlus, http://www.ncbi.nlm.nih.gov/pubmed/12975488?dopt=AbstractPlus]

**Table:** 

Nomenclature	http://www.guidetopharmacology.org/GRAC/ObjectDisplayForward?objectId=340 http://www.guidetopharmacology.org/GRAC/ObjectDisplayForward?objectId=340	http://www.guidetopharmacology.org/GRAC/ObjectDisplayForward?objectId=341	http://www.guidetopharmacology.org/GRAC/ObjectDisplayForward?objectId=342	http://www.guidetopharmacology.org/GRAC/ObjectDisplayForward?objectId=343
HGNC, UniProt	https://www.genenames.org/data/gene‐symbol‐report/#!/hgnc_id/HGNC:9593, http://www.uniprot.org/uniprot/P34995	https://www.genenames.org/data/gene‐symbol‐report/#!/hgnc_id/HGNC:9594, http://www.uniprot.org/uniprot/P43116	https://www.genenames.org/data/gene‐symbol‐report/#!/hgnc_id/HGNC:9595, http://www.uniprot.org/uniprot/P43115	https://www.genenames.org/data/gene‐symbol‐report/#!/hgnc_id/HGNC:9596, http://www.uniprot.org/uniprot/P35408
Potency order of endogenous ligands	http://www.guidetopharmacology.org/GRAC/LigandDisplayForward?ligandId=1883 > http://www.guidetopharmacology.org/GRAC/LigandDisplayForward?ligandId=1882 > http://www.guidetopharmacology.org/GRAC/LigandDisplayForward?ligandId=1884, http://www.guidetopharmacology.org/GRAC/LigandDisplayForward?ligandId=1915 > http://www.guidetopharmacology.org/GRAC/LigandDisplayForward?ligandId=1881, http://www.guidetopharmacology.org/GRAC/LigandDisplayForward?ligandId=4482	http://www.guidetopharmacology.org/GRAC/LigandDisplayForward?ligandId=1883 = http://www.guidetopharmacology.org/GRAC/LigandDisplayForward?ligandId=1882 > http://www.guidetopharmacology.org/GRAC/LigandDisplayForward?ligandId=1884, http://www.guidetopharmacology.org/GRAC/LigandDisplayForward?ligandId=1915 > http://www.guidetopharmacology.org/GRAC/LigandDisplayForward?ligandId=1881, http://www.guidetopharmacology.org/GRAC/LigandDisplayForward?ligandId=4482	http://www.guidetopharmacology.org/GRAC/LigandDisplayForward?ligandId=1883, http://www.guidetopharmacology.org/GRAC/LigandDisplayForward?ligandId=1882 > http://www.guidetopharmacology.org/GRAC/LigandDisplayForward?ligandId=1884, http://www.guidetopharmacology.org/GRAC/LigandDisplayForward?ligandId=1915 > http://www.guidetopharmacology.org/GRAC/LigandDisplayForward?ligandId=1881, http://www.guidetopharmacology.org/GRAC/LigandDisplayForward?ligandId=4482	http://www.guidetopharmacology.org/GRAC/LigandDisplayForward?ligandId=1883 = http://www.guidetopharmacology.org/GRAC/LigandDisplayForward?ligandId=1882 > http://www.guidetopharmacology.org/GRAC/LigandDisplayForward?ligandId=1884, http://www.guidetopharmacology.org/GRAC/LigandDisplayForward?ligandId=1915 > http://www.guidetopharmacology.org/GRAC/LigandDisplayForward?ligandId=1881, http://www.guidetopharmacology.org/GRAC/LigandDisplayForward?ligandId=4482
Endogenous agonists	–	http://www.guidetopharmacology.org/GRAC/LigandDisplayForward?ligandId=1883 [http://www.ncbi.nlm.nih.gov/pubmed/10634944?dopt=AbstractPlus, http://www.ncbi.nlm.nih.gov/pubmed/10462542?dopt=AbstractPlus, http://www.ncbi.nlm.nih.gov/pubmed/16604093?dopt=AbstractPlus]	http://www.guidetopharmacology.org/GRAC/LigandDisplayForward?ligandId=1883 (EP_3_‐III isoform) [http://www.ncbi.nlm.nih.gov/pubmed/10634944?dopt=AbstractPlus]	–
Agonists	http://www.guidetopharmacology.org/GRAC/LigandDisplayForward?ligandId=1912 [http://www.ncbi.nlm.nih.gov/pubmed/11999132?dopt=AbstractPlus]	http://www.guidetopharmacology.org/GRAC/LigandDisplayForward?ligandId=5820 [http://www.ncbi.nlm.nih.gov/pubmed/25542069?dopt=AbstractPlus, http://www.ncbi.nlm.nih.gov/pubmed/22480736?dopt=AbstractPlus], http://www.guidetopharmacology.org/GRAC/LigandDisplayForward?ligandId=1882 [http://www.ncbi.nlm.nih.gov/pubmed/8163486?dopt=AbstractPlus]	http://www.guidetopharmacology.org/GRAC/LigandDisplayForward?ligandId=1936) (EP_3_‐III isoform) [http://www.ncbi.nlm.nih.gov/pubmed/10634944?dopt=AbstractPlus]	http://www.guidetopharmacology.org/GRAC/LigandDisplayForward?ligandId=1912 [http://www.ncbi.nlm.nih.gov/pubmed/27664754?dopt=AbstractPlus]
Selective agonists	http://www.guidetopharmacology.org/GRAC/LigandDisplayForward?ligandId=1914 [http://www.ncbi.nlm.nih.gov/pubmed/10746663?dopt=AbstractPlus] – Mouse	http://www.guidetopharmacology.org/GRAC/LigandDisplayForward?ligandId=1932 [http://www.ncbi.nlm.nih.gov/pubmed/10746663?dopt=AbstractPlus] – Mouse, http://www.guidetopharmacology.org/GRAC/LigandDisplayForward?ligandId=10315 [http://www.ncbi.nlm.nih.gov/pubmed/29332128?dopt=AbstractPlus], http://www.guidetopharmacology.org/GRAC/LigandDisplayForward?ligandId=1892 [http://www.ncbi.nlm.nih.gov/pubmed/10634944?dopt=AbstractPlus, http://www.ncbi.nlm.nih.gov/pubmed/10462542?dopt=AbstractPlus]	http://www.guidetopharmacology.org/GRAC/LigandDisplayForward?ligandId=1919 (EP_3_‐III isoform) [http://www.ncbi.nlm.nih.gov/pubmed/10634944?dopt=AbstractPlus], http://www.guidetopharmacology.org/GRAC/LigandDisplayForward?ligandId=1931 [http://www.ncbi.nlm.nih.gov/pubmed/21323896?dopt=AbstractPlus, http://www.ncbi.nlm.nih.gov/pubmed/22342278?dopt=AbstractPlus]	http://www.guidetopharmacology.org/GRAC/LigandDisplayForward?ligandId=3357 [http://www.ncbi.nlm.nih.gov/pubmed/18516068?dopt=AbstractPlus, http://www.ncbi.nlm.nih.gov/pubmed/19584306?dopt=AbstractPlus], http://www.guidetopharmacology.org/GRAC/LigandDisplayForward?ligandId=1933 [http://www.ncbi.nlm.nih.gov/pubmed/21323896?dopt=AbstractPlus]
Antagonists	–	–	–	http://www.guidetopharmacology.org/GRAC/LigandDisplayForward?ligandId=1952 (p*K*_i_ 7.6–8.5) [http://www.ncbi.nlm.nih.gov/pubmed/11408598?dopt=AbstractPlus, 2406]
Selective antagonists	http://www.guidetopharmacology.org/GRAC/LigandDisplayForward?ligandId=1920 (p*K*_i_ 9.2) [http://www.ncbi.nlm.nih.gov/pubmed/10537280?dopt=AbstractPlus], http://www.guidetopharmacology.org/GRAC/LigandDisplayForward?ligandId=1924 (p*K*_i_ 7.9) [http://www.ncbi.nlm.nih.gov/pubmed/10634944?dopt=AbstractPlus]	http://www.guidetopharmacology.org/GRAC/LigandDisplayForward?ligandId=5817 (PF‐04418948 has weaker affinity at the EP_2_‐receptor in guinea‐pigs) (p*K*_B_ 8.3) [http://www.ncbi.nlm.nih.gov/pubmed/21595651?dopt=AbstractPlus, http://www.ncbi.nlm.nih.gov/pubmed/22747912?dopt=AbstractPlus], http://www.guidetopharmacology.org/GRAC/LigandDisplayForward?ligandId=9283 (p*K*_B_ 8.1) [http://www.ncbi.nlm.nih.gov/pubmed/23914286?dopt=AbstractPlus]	http://www.guidetopharmacology.org/GRAC/LigandDisplayForward?ligandId=5844 (EP_3_‐III isoform (pK_i_=8.04 in the presence of HSA)) (p*K*_i_ 9.1) [http://www.ncbi.nlm.nih.gov/pubmed/11504634?dopt=AbstractPlus], http://www.guidetopharmacology.org/GRAC/LigandDisplayForward?ligandId=1943 (pIC_50_ 8.8) [http://www.ncbi.nlm.nih.gov/pubmed/12538661?dopt=AbstractPlus] – Mouse, http://www.guidetopharmacology.org/GRAC/LigandDisplayForward?ligandId=5822 (p*K*_i_ 8.4) [http://www.ncbi.nlm.nih.gov/pubmed/19486006?dopt=AbstractPlus]	http://www.guidetopharmacology.org/GRAC/LigandDisplayForward?ligandId=1942 (p*K*_i_ 8.5), http://www.guidetopharmacology.org/GRAC/LigandDisplayForward?ligandId=1953 (p*K*_i_ 7–7.1) [http://www.ncbi.nlm.nih.gov/pubmed/16604093?dopt=AbstractPlus, http://www.ncbi.nlm.nih.gov/pubmed/15655509?dopt=AbstractPlus]
Labelled ligands	http://www.guidetopharmacology.org/GRAC/LigandDisplayForward?ligandId=1916 (Agonist) [http://www.ncbi.nlm.nih.gov/pubmed/10634944?dopt=AbstractPlus, http://www.ncbi.nlm.nih.gov/pubmed/11999132?dopt=AbstractPlus, http://www.ncbi.nlm.nih.gov/pubmed/16604093?dopt=AbstractPlus]	http://www.guidetopharmacology.org/GRAC/LigandDisplayForward?ligandId=1916 (Agonist) [http://www.ncbi.nlm.nih.gov/pubmed/10634944?dopt=AbstractPlus, http://www.ncbi.nlm.nih.gov/pubmed/16604093?dopt=AbstractPlus]	http://www.guidetopharmacology.org/GRAC/LigandDisplayForward?ligandId=1916 (Agonist) [http://www.ncbi.nlm.nih.gov/pubmed/10634944?dopt=AbstractPlus, http://www.ncbi.nlm.nih.gov/pubmed/16604093?dopt=AbstractPlus]	http://www.guidetopharmacology.org/GRAC/LigandDisplayForward?ligandId=1916 (Agonist) [http://www.ncbi.nlm.nih.gov/pubmed/10634944?dopt=AbstractPlus, http://www.ncbi.nlm.nih.gov/pubmed/10952683?dopt=AbstractPlus, http://www.ncbi.nlm.nih.gov/pubmed/22480736?dopt=AbstractPlus, http://www.ncbi.nlm.nih.gov/pubmed/16604093?dopt=AbstractPlus]

**Table:** 

Nomenclature	http://www.guidetopharmacology.org/GRAC/ObjectDisplayForward?objectId=344	http://www.guidetopharmacology.org/GRAC/ObjectDisplayForward?objectId=345	http://www.guidetopharmacology.org/GRAC/ObjectDisplayForward?objectId=346
HGNC, UniProt	https://www.genenames.org/data/gene‐symbol‐report/#!/hgnc_id/HGNC:9600, http://www.uniprot.org/uniprot/P43088	https://www.genenames.org/data/gene‐symbol‐report/#!/hgnc_id/HGNC:9602, http://www.uniprot.org/uniprot/P43119	https://www.genenames.org/data/gene‐symbol‐report/#!/hgnc_id/HGNC:11608, http://www.uniprot.org/uniprot/P21731
Potency order of endogenous ligands	http://www.guidetopharmacology.org/GRAC/LigandDisplayForward?ligandId=1884 > http://www.guidetopharmacology.org/GRAC/LigandDisplayForward?ligandId=1881 > http://www.guidetopharmacology.org/GRAC/LigandDisplayForward?ligandId=1883 > http://www.guidetopharmacology.org/GRAC/LigandDisplayForward?ligandId=1915, http://www.guidetopharmacology.org/GRAC/LigandDisplayForward?ligandId=4482	http://www.guidetopharmacology.org/GRAC/LigandDisplayForward?ligandId=1915 http://www.guidetopharmacology.org/GRAC/LigandDisplayForward?ligandId=1882 ≫ http://www.guidetopharmacology.org/GRAC/LigandDisplayForward?ligandId=1881, http://www.guidetopharmacology.org/GRAC/LigandDisplayForward?ligandId=1884 > http://www.guidetopharmacology.org/GRAC/LigandDisplayForward?ligandId=4482	http://www.guidetopharmacology.org/GRAC/LigandDisplayForward?ligandId=4482 = http://www.guidetopharmacology.org/GRAC/LigandDisplayForward?ligandId=4483 ≫ http://www.guidetopharmacology.org/GRAC/LigandDisplayForward?ligandId=1881, http://www.guidetopharmacology.org/GRAC/LigandDisplayForward?ligandId=1883, http://www.guidetopharmacology.org/GRAC/LigandDisplayForward?ligandId=1884, http://www.guidetopharmacology.org/GRAC/LigandDisplayForward?ligandId=1915
Endogenous agonists	–	http://www.guidetopharmacology.org/GRAC/LigandDisplayForward?ligandId=1915 [http://www.ncbi.nlm.nih.gov/pubmed/372237?dopt=AbstractPlus], http://www.guidetopharmacology.org/GRAC/LigandDisplayForward?ligandId=1882 [http://www.ncbi.nlm.nih.gov/pubmed/11454473?dopt=AbstractPlus, http://www.ncbi.nlm.nih.gov/pubmed/17704830?dopt=AbstractPlus]	–
Agonists	http://www.guidetopharmacology.org/GRAC/LigandDisplayForward?ligandId=9875 [http://www.ncbi.nlm.nih.gov/pubmed/25788650?dopt=AbstractPlus]	http://www.guidetopharmacology.org/GRAC/LigandDisplayForward?ligandId=1895 [http://www.ncbi.nlm.nih.gov/pubmed/10634944?dopt=AbstractPlus, http://www.ncbi.nlm.nih.gov/pubmed/16604093?dopt=AbstractPlus], http://www.guidetopharmacology.org/GRAC/LigandDisplayForward?ligandId=5820 [http://www.ncbi.nlm.nih.gov/pubmed/22480736?dopt=AbstractPlus]	–
Selective agonists	http://www.guidetopharmacology.org/GRAC/LigandDisplayForward?ligandId=1940 [http://www.ncbi.nlm.nih.gov/pubmed/10634944?dopt=AbstractPlus], http://www.guidetopharmacology.org/GRAC/LigandDisplayForward?ligandId=1960 [http://www.ncbi.nlm.nih.gov/pubmed/10634944?dopt=AbstractPlus]	http://www.guidetopharmacology.org/GRAC/LigandDisplayForward?ligandId=1917 [http://www.ncbi.nlm.nih.gov/pubmed/10634944?dopt=AbstractPlus], http://www.guidetopharmacology.org/GRAC/LigandDisplayForward?ligandId=5852 [http://www.ncbi.nlm.nih.gov/pubmed/23850788?dopt=AbstractPlus, http://www.ncbi.nlm.nih.gov/pubmed/17545310?dopt=AbstractPlus]	http://www.guidetopharmacology.org/GRAC/LigandDisplayForward?ligandId=1888 [http://www.ncbi.nlm.nih.gov/pubmed/10634944?dopt=AbstractPlus]
Antagonists	–	–	http://www.guidetopharmacology.org/GRAC/LigandDisplayForward?ligandId=1911 (p*K*_i_ 8) [http://www.ncbi.nlm.nih.gov/pubmed/1387312?dopt=AbstractPlus]
Selective antagonists	http://www.guidetopharmacology.org/GRAC/LigandDisplayForward?ligandId=3424 (p*K*_i_ 7.5) [http://www.ncbi.nlm.nih.gov/pubmed/17618756?dopt=AbstractPlus]	http://www.guidetopharmacology.org/GRAC/LigandDisplayForward?ligandId=1969 (p*K*_i_ 8.7) [http://www.ncbi.nlm.nih.gov/pubmed/16331286?dopt=AbstractPlus], http://www.guidetopharmacology.org/GRAC/LigandDisplayForward?ligandId=4042 (p*A*_2_ 8.5) [http://www.ncbi.nlm.nih.gov/pubmed/16331286?dopt=AbstractPlus]	http://www.guidetopharmacology.org/GRAC/LigandDisplayForward?ligandId=1976 (p*K*_i_ 8.3–9.4) [http://www.ncbi.nlm.nih.gov/pubmed/8242228?dopt=AbstractPlus, http://www.ncbi.nlm.nih.gov/pubmed/2527074?dopt=AbstractPlus], http://www.guidetopharmacology.org/GRAC/LigandDisplayForward?ligandId=1980 (p*K*_i_ 8.1–9.1) [http://www.ncbi.nlm.nih.gov/pubmed/10634944?dopt=AbstractPlus, http://www.ncbi.nlm.nih.gov/pubmed/2975605?dopt=AbstractPlus, http://www.ncbi.nlm.nih.gov/pubmed/16604093?dopt=AbstractPlus]
Labelled ligands	http://www.guidetopharmacology.org/GRAC/LigandDisplayForward?ligandId=1957 (Agonist) [http://www.ncbi.nlm.nih.gov/pubmed/10634944?dopt=AbstractPlus, http://www.ncbi.nlm.nih.gov/pubmed/8300593?dopt=AbstractPlus, http://www.ncbi.nlm.nih.gov/pubmed/16604093?dopt=AbstractPlus], http://www.guidetopharmacology.org/GRAC/LigandDisplayForward?ligandId=3417 (Agonist)	http://www.guidetopharmacology.org/GRAC/LigandDisplayForward?ligandId=1966 (Agonist) [http://www.ncbi.nlm.nih.gov/pubmed/10634944?dopt=AbstractPlus, http://www.ncbi.nlm.nih.gov/pubmed/7512962?dopt=AbstractPlus, http://www.ncbi.nlm.nih.gov/pubmed/22480736?dopt=AbstractPlus, http://www.ncbi.nlm.nih.gov/pubmed/16604093?dopt=AbstractPlus]	http://www.guidetopharmacology.org/GRAC/LigandDisplayForward?ligandId=1982 (Antagonist) (p*K*_d_ 7.7–9.3) [http://www.ncbi.nlm.nih.gov/pubmed/1386885?dopt=AbstractPlus], http://www.guidetopharmacology.org/GRAC/LigandDisplayForward?ligandId=1973 (Agonist) [http://www.ncbi.nlm.nih.gov/pubmed/2530338?dopt=AbstractPlus], http://www.guidetopharmacology.org/GRAC/LigandDisplayForward?ligandId=1985 (Antagonist) (p*K*_d_ 7.4–8.2) [http://www.ncbi.nlm.nih.gov/pubmed/10634944?dopt=AbstractPlus, http://www.ncbi.nlm.nih.gov/pubmed/16604093?dopt=AbstractPlus]

### Comments

Whilst http://www.guidetopharmacology.org/GRAC/LigandDisplayForward?ligandId=1917 is selective for IP receptors, it does exhibit moderate agonist potency at EP_4_ receptors [http://www.ncbi.nlm.nih.gov/pubmed/10634944?dopt=AbstractPlus]. Apart from IP receptors, http://www.guidetopharmacology.org/GRAC/LigandDisplayForward?ligandId=1895 also binds to EP_1_ receptors.

The EP_1_ agonist http://www.guidetopharmacology.org/GRAC/LigandDisplayForward?ligandId=1912 also shows agonist activity at EP_3_ and EP_4_ receptors [http://www.ncbi.nlm.nih.gov/pubmed/21323896?dopt=AbstractPlus, http://www.ncbi.nlm.nih.gov/pubmed/27664754?dopt=AbstractPlus]. http://www.guidetopharmacology.org/GRAC/LigandDisplayForward?ligandId=3379 and http://www.guidetopharmacology.org/GRAC/LigandDisplayForward?ligandId=3331 may require de‐esterification within tissues to attain full agonist potency. There is evidence for subtypes of FP [http://www.ncbi.nlm.nih.gov/pubmed/7830272?dopt=AbstractPlus] and TP receptors [http://www.ncbi.nlm.nih.gov/pubmed/8882612?dopt=AbstractPlus, http://www.ncbi.nlm.nih.gov/pubmed/8034687?dopt=AbstractPlus]. mRNA for the EP_3_ receptor undergoes alternative splicing to produce variants which can interfere with signalling [http://www.ncbi.nlm.nih.gov/pubmed/8940129?dopt=AbstractPlus] or generate complex patterns of G‐protein (G_i/o_, G_q/11_, G_s_ and G_12_,_13_) coupling (*e.g.* [http://www.ncbi.nlm.nih.gov/pubmed/7476918?dopt=AbstractPlus, http://www.ncbi.nlm.nih.gov/pubmed/7608175?dopt=AbstractPlus]). The number of EP_3_ receptor (protein) variants are variable depending on species, with five in human, three in rat and three in mouse. Putative receptor(s) for prostamide F (which as yet lack molecular correlates) and which preferentially recognize http://www.guidetopharmacology.org/GRAC/LigandDisplayForward?ligandId=5456 and its analogues (*e.g*. http://www.guidetopharmacology.org/GRAC/LigandDisplayForward?ligandId=1958) have been identified, together with moderate‐potency antagonists (*e.g.* http://www.guidetopharmacology.org/GRAC/LigandDisplayForward?ligandId=5455) [http://www.ncbi.nlm.nih.gov/pubmed/18700152?dopt=AbstractPlus].

The free acid form of AL‐12182, http://www.guidetopharmacology.org/GRAC/LigandDisplayForward?ligandId=3386, used in *in vitro* studies, has a EC_50_ of 15nM which is the concentration of the compound giving half‐maximal stimulation of inositol phosphate turnover in HEK‐293 cells expressing the human FP receptor [http://www.ncbi.nlm.nih.gov/pubmed/17076623?dopt=AbstractPlus].

References given alongside the TP receptor agonists I‐BOP [http://www.ncbi.nlm.nih.gov/pubmed/1830308?dopt=AbstractPlus] and STA_2_ [http://www.ncbi.nlm.nih.gov/pubmed/8242228?dopt=AbstractPlus] use human platelets as the source of TP receptors for competition radio‐ligand binding assays to determine the indicated activity values.

Pharmacological evidence for a second IP receptor, denoted IP_2_, in the central nervous system [http://www.ncbi.nlm.nih.gov/pubmed/8621463?dopt=AbstractPlus, http://www.ncbi.nlm.nih.gov/pubmed/10349870?dopt=AbstractPlus] and in the BEAS‐2B human airway epithelial cell line [http://www.ncbi.nlm.nih.gov/pubmed/21173040?dopt=AbstractPlus] is available. This receptor is selectively activated by 15R‐17,18,19,20‐tetranor‐16‐m‐tolylisocarbacyclin (http://www.guidetopharmacology.org/GRAC/LigandDisplayForward?ligandId=5864) and 15R‐Deoxy 17,18,19,20‐tetranor‐16m‐tolyl‐isocarbacyclin (http://www.guidetopharmacology.org/GRAC/LigandDisplayForward?ligandId=5865). However, molecular biological evidence for an IP_2_ subtype is currently lacking.

### Further reading on Prostanoid receptors

Woodward DF *et al*. (2011) International union of basic and clinical pharmacology. LXXXIII: classification of prostanoid receptors, updating 15 years of progress. *Pharmacol. Rev.* **63**: 471‐538 [https://www.ncbi.nlm.nih.gov/pubmed/21752876?dopt=AbstractPlus]

## http://www.guidetopharmacology.org/GRAC/FamilyDisplayForward?familyId=59

### Overview

Proteinase‐activated receptors (PARs, **nomenclature as agreed by the NC‐IUPHAR Subcommittee on Proteinase‐activated Receptors** [http://www.ncbi.nlm.nih.gov/pubmed/12037136?dopt=AbstractPlus]) are unique members of the GPCR superfamily activated by proteolytic cleavage of their amino terminal exodomains. Agonist proteinase‐induced hydrolysis unmasks a tethered ligand (TL) at the exposed amino terminus, which acts intramolecularly at the binding site in the body of the receptor to effect transmembrane signalling. TL sequences at human PAR1‐4 are http://www.guidetopharmacology.org/GRAC/LigandDisplayForward?ligandId=5361, http://www.guidetopharmacology.org/GRAC/LigandDisplayForward?ligandId=3740, http://www.guidetopharmacology.org/GRAC/LigandDisplayForward?ligandId=5360 and http://www.guidetopharmacology.org/GRAC/LigandDisplayForward?ligandId=3739, respectively. With the exception of PAR3, synthetic peptides with these sequences (as carboxyl terminal amides) are able to act as agonists at their respective receptors. Several proteinases, including neutrophil elastase, cathepsin G and chymotrypsin can have inhibitory effects at PAR1 and PAR2 such that they cleave the exodomain of the receptor without inducing activation of Gαq‐coupled calcium signalling, thereby preventing activation by activating proteinases but not by agonist peptides. Neutrophil elastase (NE) cleavage of PAR1 and PAR2 can however activate MAP kinase signaling by exposing a TL that is different from the one revealed by trypsin [http://www.ncbi.nlm.nih.gov/pubmed/22212680?dopt=AbstractPlus]. PAR2 ectivation by NE regulates inflammation and pain responses [http://www.ncbi.nlm.nih.gov/pubmed/26140667?dopt=AbstractPlus, http://www.ncbi.nlm.nih.gov/pubmed/25878251?dopt=AbstractPlus] and triggers mucin secretion from airway epithelial cells [http://www.ncbi.nlm.nih.gov/pubmed/23392769?dopt=AbstractPlus].

**Table:** 

Nomenclature	http://www.guidetopharmacology.org/GRAC/ObjectDisplayForward?objectId=347	http://www.guidetopharmacology.org/GRAC/ObjectDisplayForward?objectId=348	http://www.guidetopharmacology.org/GRAC/ObjectDisplayForward?objectId=349	http://www.guidetopharmacology.org/GRAC/ObjectDisplayForward?objectId=350
HGNC, UniProt	https://www.genenames.org/data/gene‐symbol‐report/#!/hgnc_id/HGNC:3537, http://www.uniprot.org/uniprot/P25116	https://www.genenames.org/data/gene‐symbol‐report/#!/hgnc_id/HGNC:3538, http://www.uniprot.org/uniprot/P55085	https://www.genenames.org/data/gene‐symbol‐report/#!/hgnc_id/HGNC:3539, http://www.uniprot.org/uniprot/O00254	https://www.genenames.org/data/gene‐symbol‐report/#!/hgnc_id/HGNC:3540, http://www.uniprot.org/uniprot/Q96RI0
Agonist proteases	http://www.guidetopharmacology.org/GRAC/LigandDisplayForward?ligandId=4453 (https://www.genenames.org/data/gene‐symbol‐report/#!/hgnc_id/HGNC:3535, http://www.uniprot.org/uniprot/P00734), activated http://www.guidetopharmacology.org/GRAC/LigandDisplayForward?ligandId=4473 (https://www.genenames.org/data/gene‐symbol‐report/#!/hgnc_id/HGNC:9451, http://www.uniprot.org/uniprot/P04070), http://www.guidetopharmacology.org/GRAC/LigandDisplayForward?ligandId=6655 (https://www.genenames.org/data/gene‐symbol‐report/#!/hgnc_id/HGNC:7155, http://www.uniprot.org/uniprot/P45452), http://www.guidetopharmacology.org/GRAC/LigandDisplayForward?ligandId=6656 (https://www.genenames.org/data/gene‐symbol‐report/#!/hgnc_id/HGNC:7159, http://www.uniprot.org/uniprot/P45452) [http://www.ncbi.nlm.nih.gov/pubmed/23086754?dopt=AbstractPlus]	Trypsin, tryptase, TF/VIIa, Xa; http://www.guidetopharmacology.org/GRAC/ObjectDisplayForward?objectId=2358 http://www.guidetopharmacology.org/GRAC/ObjectDisplayForward?objectId=2353 [http://www.ncbi.nlm.nih.gov/pubmed/30012612?dopt=AbstractPlus, http://www.ncbi.nlm.nih.gov/pubmed/21576245?dopt=AbstractPlus]	http://www.guidetopharmacology.org/GRAC/LigandDisplayForward?ligandId=4453 (https://www.genenames.org/data/gene‐symbol‐report/#!/hgnc_id/HGNC:3535, http://www.uniprot.org/uniprot/P00734)	http://www.guidetopharmacology.org/GRAC/LigandDisplayForward?ligandId=4453 (https://www.genenames.org/data/gene‐symbol‐report/#!/hgnc_id/HGNC:3535, http://www.uniprot.org/uniprot/P00734), trypsin, http://www.guidetopharmacology.org/GRAC/LigandDisplayForward?ligandId=3570 (https://www.genenames.org/data/gene‐symbol‐report/#!/hgnc_id/HGNC:2532, http://www.uniprot.org/uniprot/P08311)
Agonists	http://www.guidetopharmacology.org/GRAC/LigandDisplayForward?ligandId=9255	–	–	–
Selective agonists	http://www.guidetopharmacology.org/GRAC/LigandDisplayForward?ligandId=3742 [http://www.ncbi.nlm.nih.gov/pubmed/11877318?dopt=AbstractPlus]	http://www.guidetopharmacology.org/GRAC/LigandDisplayForward?ligandId=9455 [http://www.ncbi.nlm.nih.gov/pubmed/26819675?dopt=AbstractPlus], http://www.guidetopharmacology.org/GRAC/LigandDisplayForward?ligandId=6657 [http://www.ncbi.nlm.nih.gov/pubmed/20873792?dopt=AbstractPlus], http://www.guidetopharmacology.org/GRAC/LigandDisplayForward?ligandId=3851 [http://www.ncbi.nlm.nih.gov/pubmed/14976230?dopt=AbstractPlus], http://www.guidetopharmacology.org/GRAC/LigandDisplayForward?ligandId=3740 [http://www.ncbi.nlm.nih.gov/pubmed/18179608?dopt=AbstractPlus], http://www.guidetopharmacology.org/GRAC/LigandDisplayForward?ligandId=3741 [http://www.ncbi.nlm.nih.gov/pubmed/18179608?dopt=AbstractPlus]	–	http://www.guidetopharmacology.org/GRAC/LigandDisplayForward?ligandId=4061, http://www.guidetopharmacology.org/GRAC/LigandDisplayForward?ligandId=3883, http://www.guidetopharmacology.org/GRAC/LigandDisplayForward?ligandId=3739
Selective antagonists	http://www.guidetopharmacology.org/GRAC/LigandDisplayForward?ligandId=4047 (p*K*_i_ 8.1) [http://www.ncbi.nlm.nih.gov/pubmed/18447380?dopt=AbstractPlus], http://www.guidetopharmacology.org/GRAC/LigandDisplayForward?ligandId=4048 (pIC_50_ 7.7) [http://www.ncbi.nlm.nih.gov/pubmed/21300059?dopt=AbstractPlus], http://www.guidetopharmacology.org/GRAC/LigandDisplayForward?ligandId=3525 (pIC_50_ 6.4) [http://www.ncbi.nlm.nih.gov/pubmed/10535908?dopt=AbstractPlus]	http://www.guidetopharmacology.org/GRAC/LigandDisplayForward?ligandId=10268 (pIC_50_ 7.1) [http://www.ncbi.nlm.nih.gov/pubmed/29263243?dopt=AbstractPlus], http://www.guidetopharmacology.org/GRAC/LigandDisplayForward?ligandId=9585 (p*K*_d_ 6.5) [http://www.ncbi.nlm.nih.gov/pubmed/28445455?dopt=AbstractPlus], http://www.guidetopharmacology.org/GRAC/LigandDisplayForward?ligandId=6658 (pIC_50_ 5.7) [http://www.ncbi.nlm.nih.gov/pubmed/21806599?dopt=AbstractPlus], http://www.guidetopharmacology.org/GRAC/LigandDisplayForward?ligandId=6659 [http://www.ncbi.nlm.nih.gov/pubmed/21536878?dopt=AbstractPlus]	–	http://www.guidetopharmacology.org/GRAC/LigandDisplayForward?ligandId=10269 (p*K*_d_ 10) [http://www.ncbi.nlm.nih.gov/pubmed/28053157?dopt=AbstractPlus], http://www.guidetopharmacology.org/GRAC/LigandDisplayForward?ligandId=9458 (pIC_50_ 6.9) [http://www.ncbi.nlm.nih.gov/pubmed/25176330?dopt=AbstractPlus], http://www.guidetopharmacology.org/GRAC/LigandDisplayForward?ligandId=9459 (pIC_50_ 6.8) [http://www.ncbi.nlm.nih.gov/pubmed/25176330?dopt=AbstractPlus], http://www.guidetopharmacology.org/GRAC/LigandDisplayForward?ligandId=10270 [http://www.ncbi.nlm.nih.gov/pubmed/12357249?dopt=AbstractPlus], http://www.guidetopharmacology.org/GRAC/LigandDisplayForward?ligandId=10271 (Agonist) [http://www.ncbi.nlm.nih.gov/pubmed/28126849?dopt=AbstractPlus]
Allosteric modulators	–	http://www.guidetopharmacology.org/GRAC/LigandDisplayForward?ligandId=9560 (Negative) (pIC_50_ 7.6) [http://www.ncbi.nlm.nih.gov/pubmed/28445455?dopt=AbstractPlus]	–	–
Labelled ligands	[http://www.guidetopharmacology.org/GRAC/LigandDisplayForward?ligandId=3823 (Agonist) [http://www.ncbi.nlm.nih.gov/pubmed/9203642?dopt=AbstractPlus]	http://www.guidetopharmacology.org/GRAC/LigandDisplayForward?ligandId=4050 (Agonist) [http://www.ncbi.nlm.nih.gov/pubmed/18477767?dopt=AbstractPlus], http://www.guidetopharmacology.org/GRAC/LigandDisplayForward?ligandId=4049 (Agonist) [http://www.ncbi.nlm.nih.gov/pubmed/18477767?dopt=AbstractPlus], [http://www.guidetopharmacology.org/GRAC/LigandDisplayForward?ligandId=6660 (Selective Agonist) [http://www.ncbi.nlm.nih.gov/pubmed/15765104?dopt=AbstractPlus], http://www.guidetopharmacology.org/GRAC/LigandDisplayForward?ligandId=3914 (Agonist) [http://www.ncbi.nlm.nih.gov/pubmed/10411588?dopt=AbstractPlus]	–	–
Comments	http://www.guidetopharmacology.org/GRAC/LigandDisplayForward?ligandId=3742 is selective relative to the PAR_2_ receptor [http://www.ncbi.nlm.nih.gov/pubmed/8663335?dopt=AbstractPlus, http://www.ncbi.nlm.nih.gov/pubmed/9862790?dopt=AbstractPlus].	2‐Furoyl‐LIGRLO‐NH_2_ activity was measured via calcium mobilisation in HEK 293 cells which constitutively coexpress human PAR_1_ and PAR_2_.	–	–

### Comments

Endogenous serine proteases (EC 3.4.21.) active at the proteinase‐activated receptors include: http://www.guidetopharmacology.org/GRAC/LigandDisplayForward?ligandId=4453 (https://www.genenames.org/data/gene‐symbol‐report/#!/hgnc_id/HGNC:3535, http://www.uniprot.org/uniprot/P00734), generated by the action of Factor X (https://www.genenames.org/data/gene‐symbol‐report/#!/hgnc_id/HGNC:3528, http://www.uniprot.org/uniprot/P00742) on liver‐derived prothrombin (https://www.genenames.org/data/gene‐symbol‐report/#!/hgnc_id/HGNC:3535, http://www.uniprot.org/uniprot/P00734); trypsin, generated by the action of enterokinase (https://www.genenames.org/data/gene‐symbol‐report/#!/hgnc_id/HGNC:9490, http://www.uniprot.org/uniprot/P98073) on pancreatic‐derived trypsinogen (https://www.genenames.org/data/gene‐symbol‐report/#!/hgnc_id/HGNC:9475, http://www.uniprot.org/uniprot/P07477); tryptase, a family of enzymes (α/β1 https://www.genenames.org/data/gene‐symbol‐report/#!/hgnc_id/HGNC:12019, http://www.uniprot.org/uniprot/Q15661; γ1 https://www.genenames.org/data/gene‐symbol‐report/#!/hgnc_id/HGNC:14134, http://www.uniprot.org/uniprot/Q9NRR2 *δ*1 https://www.genenames.org/data/gene‐symbol‐report/#!/hgnc_id/HGNC:14118, http://www.uniprot.org/uniprot/Q9BZJ3) secreted from mast cells; cathepsin G (https://www.genenames.org/data/gene‐symbol‐report/#!/hgnc_id/HGNC:2532, http://www.uniprot.org/uniprot/P08311) generated from leukocytes; liver‐derived protein C (https://www.genenames.org/data/gene‐symbol‐report/#!/hgnc_id/HGNC:9451, http://www.uniprot.org/uniprot/P04070) generated in plasma by http://www.guidetopharmacology.org/GRAC/LigandDisplayForward?ligandId=4453 (https://www.genenames.org/data/gene‐symbol‐report/#!/hgnc_id/HGNC:3535, http://www.uniprot.org/uniprot/P00734) and http://www.guidetopharmacology.org/GRAC/LigandDisplayForward?ligandId=6655 (https://www.genenames.org/data/gene‐symbol‐report/#!/hgnc_id/HGNC:7155, http://www.uniprot.org/uniprot/P45452).

### Further reading on Proteinase‐activated receptors

Adams MN *et al.* (2011) Structure, function and pathophysiology of protease activated receptors. *Pharmacol. Ther.* **130:** 248‐82 https://www.ncbi.nlm.nih.gov/pubmed/21277892?dopt=AbstractPlus

Canto I *et al.* (2012) Allosteric modulation of protease‐activated receptor signaling. *Mini Rev Med Chem* **12:** 804‐11 https://www.ncbi.nlm.nih.gov/pubmed/22681248?dopt=AbstractPlus

García PS *et al.* (2010) The role of thrombin and protease‐activated receptors in pain mechanisms. *Thromb. Haemost.* **103:** 1145‐51 https://www.ncbi.nlm.nih.gov/pubmed/20431855?dopt=AbstractPlus

Hollenberg MD *et al.* (2002) International Union of Pharmacology. XXVIII. Proteinase‐activated receptors. *Pharmacol. Rev.* **54:** 203‐17 https://www.ncbi.nlm.nih.gov/pubmed/12037136?dopt=AbstractPlus

Ramachandran R *et al.* (2012) Targeting proteinase‐activated receptors: therapeutic potential and challenges. *Nat Rev Drug Discov* **11:** 69‐86 https://www.ncbi.nlm.nih.gov/pubmed/22212680?dopt=AbstractPlus

Soh UJ *et al.* (2010) Signal transduction by protease‐activated receptors. *Br. J. Pharmacol.* **160:** 191‐203 https://www.ncbi.nlm.nih.gov/pubmed/20423334?dopt=AbstractPlus

## http://www.guidetopharmacology.org/GRAC/FamilyDisplayForward?familyId=54

### Overview

The human gene encoding the QRFP receptor (**nomenclature as agreed by the NC‐IUPHAR Subcommittee on the QRFP receptor** [http://www.ncbi.nlm.nih.gov/pubmed/28613414?dopt=AbstractPlus]; QRFPR, formerly known as the Peptide P518 receptor), previously designated as an orphan GPCR receptor was identified in 2001 by Lee *et al.* from a hypothalamus cDNA library [http://www.ncbi.nlm.nih.gov/pubmed/11574155?dopt=AbstractPlus]. However, the reported cDNA (AF411117) is a chimera with bases 1‐127 derived from chromosome 1 and bases 155‐1368 derived from chromosome 4. When corrected, QRFPR (also referred to as SP9155 or AQ27) encodes a 431 amino acid protein that shares sequence similarities in the transmembrane spanning regions with other peptide receptors. These include neuropeptide FF2 (38%), neuropeptide Y_2_ (37%) and galanin Gal_1_ (35%) receptors.

**Table:** 

Nomenclature	http://www.guidetopharmacology.org/GRAC/ObjectDisplayForward?objectId=333
HGNC, UniProt	https://www.genenames.org/data/gene‐symbol‐report/#!/hgnc_id/HGNC:15565, http://www.uniprot.org/uniprot/Q96P65
Endogenous agonists	http://www.guidetopharmacology.org/GRAC/LigandDisplayForward?ligandId=5895 (https://www.genenames.org/data/gene‐symbol‐report/#!/hgnc_id/HGNC:29982) [http://www.ncbi.nlm.nih.gov/pubmed/14657341?dopt=AbstractPlus, http://www.ncbi.nlm.nih.gov/pubmed/12714592?dopt=AbstractPlus]
Agonists	http://www.guidetopharmacology.org/GRAC/LigandDisplayForward?ligandId=10252 [http://www.ncbi.nlm.nih.gov/pubmed/30358997?dopt=AbstractPlus], http://www.guidetopharmacology.org/GRAC/LigandDisplayForward?ligandId=9442 [http://www.ncbi.nlm.nih.gov/pubmed/22800498?dopt=AbstractPlus]
Selective antagonists	http://www.guidetopharmacology.org/GRAC/LigandDisplayForward?ligandId=9443 (pIC_50_ 7.3) [http://www.ncbi.nlm.nih.gov/pubmed/24937104?dopt=AbstractPlus, http://www.ncbi.nlm.nih.gov/pubmed/25875054?dopt=AbstractPlus]
Labelled ligands	[http://www.guidetopharmacology.org/GRAC/LigandDisplayForward?ligandId=3803) (Agonist) [http://www.ncbi.nlm.nih.gov/pubmed/12960173?dopt=AbstractPlus, 1164, http://www.ncbi.nlm.nih.gov/pubmed/16648250?dopt=AbstractPlus], [http://www.guidetopharmacology.org/GRAC/LigandDisplayForward?ligandId=10253) (Agonist) [http://www.ncbi.nlm.nih.gov/pubmed/17534937?dopt=AbstractPlus]

### Comments

The orphan receptor http://www.guidetopharmacology.org/GRAC/ (http://www.uniprot.org/uniprot/9NYM4) shows sequence similarities with the QRFP receptor, as well as with the NPFF1, NPFF2, and PrRP receptors.

### Further reading on QRFP receptor

Chartrel N *et al.* (2011) The RFamide neuropeptide 26RFa and its role in the control of neuroendocrine functions. *Front Neuroendocrinol* **32**: 387‐97 https://www.ncbi.nlm.nih.gov/pubmed/21530572?dopt=AbstractPlus

Fukusumi S *et al.* (2006) Recent advances in mammalian RFamide peptides: the discovery and functional analyses of PrRP, RFRPs and QRFP. *Peptides* **27:** 1073‐86 https://www.ncbi.nlm.nih.gov/pubmed/16500002?dopt=AbstractPlus

Leprince J *et al.* (2017) The Arg‐Phe‐amide peptide 26RFa/glutamine RF‐amide peptide and its receptor: IUPHAR Review 24. *Br. J. Pharmacol.* **174**: 3573‐3607 https://www.ncbi.nlm.nih.gov/pubmed/28613414?dopt=AbstractPlus

## http://www.guidetopharmacology.org/GRAC/FamilyDisplayForward?familyId=60

### Overview

Relaxin family peptide receptors (RXFP, **nomenclature as agreed by the NC‐IUPHAR Subcommittee on Relaxin family peptide receptors** [http://www.ncbi.nlm.nih.gov/pubmed/16507880?dopt=AbstractPlus, http://www.ncbi.nlm.nih.gov/pubmed/25761609?dopt=AbstractPlus]) may be divided into two pairs, RXFP1/2 and RXFP3/4. Endogenous agonists at these receptors are heterodimeric peptide hormones structurally related to insulin: http://www.guidetopharmacology.org/GRAC/LigandDisplayForward?ligandId=1988 (https://www.genenames.org/data/gene‐symbol‐report/#!/hgnc_id/HGNC:10026, http://www.uniprot.org/uniprot/P04808), http://www.guidetopharmacology.org/GRAC/LigandDisplayForward?ligandId=1989 (https://www.genenames.org/data/gene‐symbol‐report/#!/hgnc_id/HGNC:10027, http://www.uniprot.org/uniprot/P04090), http://www.guidetopharmacology.org/GRAC/LigandDisplayForward?ligandId=1990 (https://www.genenames.org/data/gene‐symbol‐report/#!/hgnc_id/HGNC:17135, http://www.uniprot.org/uniprot/Q8WXF3) (also known as INSL7), insulin‐like peptide 3 (http://www.guidetopharmacology.org/GRAC/LigandDisplayForward?ligandId=1995 (https://www.genenames.org/data/gene‐symbol‐report/#!/hgnc_id/HGNC:6086, http://www.uniprot.org/uniprot/P51460)) and http://www.guidetopharmacology.org/GRAC/LigandDisplayForward?ligandId=2000 (https://www.genenames.org/data/gene‐symbol‐report/#!/hgnc_id/HGNC:6088, http://www.uniprot.org/uniprot/Q9Y5Q6). Species homologues of relaxin have distinct pharmacology and http://www.guidetopharmacology.org/GRAC/LigandDisplayForward?ligandId=1989 (https://www.genenames.org/data/gene‐symbol‐report/#!/hgnc_id/HGNC:10027, http://www.uniprot.org/uniprot/P04090) interacts with RXFP1, RXFP2 and RXFP3, whereas mouse and rat relaxin selectively bind to and activate RXFP1 [http://www.ncbi.nlm.nih.gov/pubmed/15956680?dopt=AbstractPlus]. http://www.guidetopharmacology.org/GRAC/LigandDisplayForward?ligandId=1990 (https://www.genenames.org/data/gene‐symbol‐report/#!/hgnc_id/HGNC:17135, http://www.uniprot.org/uniprot/Q8WXF3) is the ligand for RXFP3 but it also binds to RXFP1 and RXFP4 and has differential affinity for RXFP2 between species [http://www.ncbi.nlm.nih.gov/pubmed/15956681?dopt=AbstractPlus]. http://www.guidetopharmacology.org/GRAC/LigandDisplayForward?ligandId=2000 (https://www.genenames.org/data/gene‐symbol‐report/#!/hgnc_id/HGNC:6088, http://www.uniprot.org/uniprot/Q9Y5Q6) is the ligand for RXFP4 but is a weak antagonist of RXFP3. http://www.guidetopharmacology.org/GRAC/LigandDisplayForward?ligandId=1989 (https://www.genenames.org/data/gene‐symbol‐report/#!/hgnc_id/HGNC:10027, http://www.uniprot.org/uniprot/P04090) and http://www.guidetopharmacology.org/GRAC/LigandDisplayForward?ligandId=1995 (https://www.genenames.org/data/gene‐symbol‐report/#!/hgnc_id/HGNC:6086, http://www.uniprot.org/uniprot/P51460) have multiple complex binding interactions with RXFP1 [http://www.ncbi.nlm.nih.gov/pubmed/27088579?dopt=AbstractPlus] and RXFP2 [http://www.ncbi.nlm.nih.gov/pubmed/30594862?dopt=AbstractPlus] which direct the N‐terminal LDLa modules of the receptors together with a linker domain to act as a tethered ligand to direct receptor signaling [http://www.ncbi.nlm.nih.gov/pubmed/16963451?dopt=AbstractPlus]. http://www.guidetopharmacology.org/GRAC/LigandDisplayForward?ligandId=2000 (https://www.genenames.org/data/gene‐symbol‐report/#!/hgnc_id/HGNC:6088, http://www.uniprot.org/uniprot/Q9Y5Q6) and http://www.guidetopharmacology.org/GRAC/LigandDisplayForward?ligandId=1990 (https://www.genenames.org/data/gene‐symbol‐report/#!/hgnc_id/HGNC:17135, http://www.uniprot.org/uniprot/Q8WXF3) interact with their receptors using distinct residues in their B‐chains for binding, and activation, respectively [http://www.ncbi.nlm.nih.gov/pubmed/28274616?dopt=AbstractPlus, http://www.ncbi.nlm.nih.gov/pubmed/30131340?dopt=AbstractPlus].

**Table:** 

Nomenclature	http://www.guidetopharmacology.org/GRAC/ObjectDisplayForward?objectId=351	http://www.guidetopharmacology.org/GRAC/ObjectDisplayForward?objectId=352	http://www.guidetopharmacology.org/GRAC/ObjectDisplayForward?objectId=353	http://www.guidetopharmacology.org/GRAC/ObjectDisplayForward?objectId=354
HGNC, UniProt	https://www.genenames.org/data/gene‐symbol‐report/#!/hgnc_id/HGNC:19718, http://www.uniprot.org/uniprot/Q9HBX9	https://www.genenames.org/data/gene‐symbol‐report/#!/hgnc_id/HGNC:17318, http://www.uniprot.org/uniprot/Q8WXD0	https://www.genenames.org/data/gene‐symbol‐report/#!/hgnc_id/HGNC:24883, http://www.uniprot.org/uniprot/Q9NSD7	https://www.genenames.org/data/gene‐symbol‐report/#!/hgnc_id/HGNC:14666, http://www.uniprot.org/uniprot/Q8TDU9
Potency order of endogenous ligands	http://www.guidetopharmacology.org/GRAC/LigandDisplayForward?ligandId=1989 (https://www.genenames.org/data/gene‐symbol‐report/#!/hgnc_id/HGNC:10027, http://www.uniprot.org/uniprot/P04090) = http://www.guidetopharmacology.org/GRAC/LigandDisplayForward?ligandId=1988 (https://www.genenames.org/data/gene‐symbol‐report/#!/hgnc_id/HGNC:10026, http://www.uniprot.org/uniprot/P04808) > http://www.guidetopharmacology.org/GRAC/LigandDisplayForward?ligandId=1990 (https://www.genenames.org/data/gene‐symbol‐report/#!/hgnc_id/HGNC:17135, http://www.uniprot.org/uniprot/Q8WXF3) [http://www.ncbi.nlm.nih.gov/pubmed/12506116?dopt=AbstractPlus]	http://www.guidetopharmacology.org/GRAC/LigandDisplayForward?ligandId=1995 (https://www.genenames.org/data/gene‐symbol‐report/#!/hgnc_id/HGNC:6086, http://www.uniprot.org/uniprot/P51460) > http://www.guidetopharmacology.org/GRAC/LigandDisplayForward?ligandId=1989 (https://www.genenames.org/data/gene‐symbol‐report/#!/hgnc_id/HGNC:10027, http://www.uniprot.org/uniprot/P04090) ≫ http://www.guidetopharmacology.org/GRAC/LigandDisplayForward?ligandId=1990 (https://www.genenames.org/data/gene‐symbol‐report/#!/hgnc_id/HGNC:17135, http://www.uniprot.org/uniprot/Q8WXF3) [http://www.ncbi.nlm.nih.gov/pubmed/12114498?dopt=AbstractPlus, http://www.ncbi.nlm.nih.gov/pubmed/12506116?dopt=AbstractPlus]	http://www.guidetopharmacology.org/GRAC/LigandDisplayForward?ligandId=1990 (https://www.genenames.org/data/gene‐symbol‐report/#!/hgnc_id/HGNC:17135, http://www.uniprot.org/uniprot/Q8WXF3) > http://www.guidetopharmacology.org/GRAC/LigandDisplayForward?ligandId=1997 (https://www.genenames.org/data/gene‐symbol‐report/#!/hgnc_id/HGNC:17135, http://www.uniprot.org/uniprot/Q8WXF3) > http://www.guidetopharmacology.org/GRAC/LigandDisplayForward?ligandId=1989 (https://www.genenames.org/data/gene‐symbol‐report/#!/hgnc_id/HGNC:10027, http://www.uniprot.org/uniprot/P04090) [http://www.ncbi.nlm.nih.gov/pubmed/14522968?dopt=AbstractPlus]	http://www.guidetopharmacology.org/GRAC/LigandDisplayForward?ligandId=2000 (https://www.genenames.org/data/gene‐symbol‐report/#!/hgnc_id/HGNC:6088, http://www.uniprot.org/uniprot/Q9Y5Q6) = http://www.guidetopharmacology.org/GRAC/LigandDisplayForward?ligandId=1990 (https://www.genenames.org/data/gene‐symbol‐report/#!/hgnc_id/HGNC:17135, http://www.uniprot.org/uniprot/Q8WXF3) > http://www.guidetopharmacology.org/GRAC/LigandDisplayForward?ligandId=1997 (https://www.genenames.org/data/gene‐symbol‐report/#!/hgnc_id/HGNC:17135, http://www.uniprot.org/uniprot/Q8WXF3) [http://www.ncbi.nlm.nih.gov/pubmed/15465925?dopt=AbstractPlus, http://www.ncbi.nlm.nih.gov/pubmed/14522967?dopt=AbstractPlus]
Endogenous antagonists	–	–	http://www.guidetopharmacology.org/GRAC/LigandDisplayForward?ligandId=2000 (https://www.genenames.org/data/gene‐symbol‐report/#!/hgnc_id/HGNC:6088, http://www.uniprot.org/uniprot/Q9Y5Q6) (p*K*_i_ 7) [http://www.ncbi.nlm.nih.gov/pubmed/18582868?dopt=AbstractPlus]	–
Antagonists	http://www.guidetopharmacology.org/GRAC/LigandDisplayForward?ligandId=4371 (pEC_50_ 5.7–6.7) [http://www.ncbi.nlm.nih.gov/pubmed/20043231?dopt=AbstractPlus, http://www.ncbi.nlm.nih.gov/pubmed/24812057?dopt=AbstractPlus]	–	http://www.guidetopharmacology.org/GRAC/LigandDisplayForward?ligandId=3907 (pIC_50_ 9.2) [http://www.ncbi.nlm.nih.gov/pubmed/17606621?dopt=AbstractPlus]	http://www.guidetopharmacology.org/GRAC/LigandDisplayForward?ligandId=3907 (pIC_50_ 8–8.6) [http://www.ncbi.nlm.nih.gov/pubmed/21384867?dopt=AbstractPlus, http://www.ncbi.nlm.nih.gov/pubmed/17606621?dopt=AbstractPlus]
Selective antagonists	–	http://www.guidetopharmacology.org/GRAC/LigandDisplayForward?ligandId=8329 (p*K*_i_ 9.1) [http://www.ncbi.nlm.nih.gov/pubmed/18434306?dopt=AbstractPlus], http://www.guidetopharmacology.org/GRAC/LigandDisplayForward?ligandId=8330 (p*K*_i_ 8.7) [http://www.ncbi.nlm.nih.gov/pubmed/18434306?dopt=AbstractPlus], http://www.guidetopharmacology.org/GRAC/LigandDisplayForward?ligandId=8331 (p*K*_i_ 8.6) [http://www.ncbi.nlm.nih.gov/pubmed/20570702?dopt=AbstractPlus], http://www.guidetopharmacology.org/GRAC/LigandDisplayForward?ligandId=4372 (p*K*i 8.5) [http://www.ncbi.nlm.nih.gov/pubmed/20560146?dopt=AbstractPlus], http://www.guidetopharmacology.org/GRAC/LigandDisplayForward?ligandId=8332 (p*K*_i_ 8.3) [http://www.ncbi.nlm.nih.gov/pubmed/20570702?dopt=AbstractPlus], http://www.guidetopharmacology.org/GRAC/LigandDisplayForward?ligandId=8333 (p*K*i 6.7) [http://www.ncbi.nlm.nih.gov/pubmed/17120268?dopt=AbstractPlus], http://www.guidetopharmacology.org/GRAC/LigandDisplayForward?ligandId=3885 (p*K*i 5.1) [http://www.ncbi.nlm.nih.gov/pubmed/16547350?dopt=AbstractPlus], (http://www.guidetopharmacology.org/GRAC/LigandDisplayForward?ligandId=3754 [http://www.ncbi.nlm.nih.gov/pubmed/15708846?dopt=AbstractPlus]	http://www.guidetopharmacology.org/GRAC/LigandDisplayForward?ligandId=8326 (p*K*_i_ 7.6) [http://www.ncbi.nlm.nih.gov/pubmed/22257012?dopt=AbstractPlus], http://www.guidetopharmacology.org/GRAC/LigandDisplayForward?ligandId=4373 (p*K*_i_ 7.4) [http://www.ncbi.nlm.nih.gov/pubmed/21384867?dopt=AbstractPlus]	http://www.guidetopharmacology.org/GRAC/LigandDisplayForward?ligandId=8326 (pIC_50_ 6.6) [http://www.ncbi.nlm.nih.gov/pubmed/22257012?dopt=AbstractPlus]
Allosteric modulators	http://www.guidetopharmacology.org/GRAC/LigandDisplayForward?ligandId=8322 (Agonist) (pEC_50_ 7) [http://www.ncbi.nlm.nih.gov/pubmed/23905199?dopt=AbstractPlus, http://www.ncbi.nlm.nih.gov/pubmed/23764525?dopt=AbstractPlus]	–	–	–
Labelled ligands	[http://www.guidetopharmacology.org/GRAC/LigandDisplayForward?ligandId=1991 (Agonist) [http://www.ncbi.nlm.nih.gov/pubmed/15649866?dopt=AbstractPlus, http://www.ncbi.nlm.nih.gov/pubmed/12506116?dopt=AbstractPlus], http://www.guidetopharmacology.org/GRAC/LigandDisplayForward?ligandId=3874 (Agonist) [http://www.ncbi.nlm.nih.gov/pubmed/22425984?dopt=AbstractPlus], [http://www.guidetopharmacology.org/GRAC/LigandDisplayForward?ligandId=5398 (Agonist)	[http://www.guidetopharmacology.org/GRAC/LigandDisplayForward?ligandId=1996 (Agonist) [http://www.ncbi.nlm.nih.gov/pubmed/16051677?dopt=AbstractPlus], [http://www.guidetopharmacology.org/GRAC/LigandDisplayForward?ligandId=1991 (Agonist) [http://www.ncbi.nlm.nih.gov/pubmed/15649866?dopt=AbstractPlus, http://www.ncbi.nlm.nih.gov/pubmed/12506116?dopt=AbstractPlus]	[http://www.guidetopharmacology.org/GRAC/LigandDisplayForward?ligandId=1999) (Agonist) [http://www.ncbi.nlm.nih.gov/pubmed/14522968?dopt=AbstractPlus], [http://www.guidetopharmacology.org/GRAC/LigandDisplayForward?ligandId=3789 (Agonist) [http://www.ncbi.nlm.nih.gov/pubmed/15465925?dopt=AbstractPlus], http://www.guidetopharmacology.org/GRAC/LigandDisplayForward?ligandId=4374 (Agonist) [http://www.ncbi.nlm.nih.gov/pubmed/21384867?dopt=AbstractPlus]	[http://www.guidetopharmacology.org/GRAC/LigandDisplayForward?ligandId=1999) (Agonist) [http://www.ncbi.nlm.nih.gov/pubmed/14522967?dopt=AbstractPlus], [http://www.guidetopharmacology.org/GRAC/LigandDisplayForward?ligandId=3789 (Agonist) [http://www.ncbi.nlm.nih.gov/pubmed/15465925?dopt=AbstractPlus], http://www.guidetopharmacology.org/GRAC/LigandDisplayForward?ligandId=6522 (Agonist) [http://www.ncbi.nlm.nih.gov/pubmed/21866895?dopt=AbstractPlus], http://www.guidetopharmacology.org/GRAC/LigandDisplayForward?ligandId=4374 (Agonist) [http://www.ncbi.nlm.nih.gov/pubmed/21384867?dopt=AbstractPlus], http://www.guidetopharmacology.org/GRAC/LigandDisplayForward?ligandId=4375 (p*K*_d_ 8.3) [http://www.ncbi.nlm.nih.gov/pubmed/21384867?dopt=AbstractPlus]
Comments	–	http://www.guidetopharmacology.org/GRAC/LigandDisplayForward?ligandId=3875 is a fluorescent ligand for this receptor (*K*_d_=1nM) [http://www.ncbi.nlm.nih.gov/pubmed/18529069?dopt=AbstractPlus].	–	–

### Comments

http://www.guidetopharmacology.org/GRAC/LigandDisplayForward?ligandId=1989 (https://www.genenames.org/data/gene‐symbol‐report/#!/hgnc_id/HGNC:10027, http://www.uniprot.org/uniprot/P04090) is the cognate peptide ligand for RXFP1 and is in extended Phase III clinical trials for the treatment of acute heart failure [http://www.ncbi.nlm.nih.gov/pubmed/23273292?dopt=AbstractPlus]. Relaxin has vasodilatory, anti‐fibrotic, angiogenic, anti‐apoptotic and anti‐inflammatory effects. A small molecule allosteric agonists http://www.guidetopharmacology.org/GRAC/LigandDisplayForward?ligandId=8322 has been developed [http://www.ncbi.nlm.nih.gov/pubmed/18854305?dopt=AbstractPlus, http://www.ncbi.nlm.nih.gov/pubmed/23764525?dopt=AbstractPlus], and a relaxin B‐chain mimetic peptide B7‐33 has been developed which that has cell specific signaling properties [http://www.ncbi.nlm.nih.gov/pubmed/30155023?dopt=AbstractPlus]. The antifibrotic actions of relaxin are dependent on the angiotensin receptor AT_2_ [http://www.ncbi.nlm.nih.gov/pubmed/24429402?dopt=AbstractPlus]. http://www.guidetopharmacology.org/GRAC/LigandDisplayForward?ligandId=1995 (https://www.genenames.org/data/gene‐symbol‐report/#!/hgnc_id/HGNC:6086, http://www.uniprot.org/uniprot/P51460) is the cognate peptide for RXFP2 and is a circulating hormone that in males is essential for testicular descent *in utero* [http://www.ncbi.nlm.nih.gov/pubmed/10391220?dopt=AbstractPlus] and in females has important roles in ovarian follicle function [http://www.ncbi.nlm.nih.gov/pubmed/30204868?dopt=AbstractPlus]. In adults, INSL3 has potential roles in testicular function [http://www.ncbi.nlm.nih.gov/pubmed/20952422?dopt=AbstractPlus] and the musculoskeletal system [http://www.ncbi.nlm.nih.gov/pubmed/30625346?dopt=AbstractPlus]. RXFP2 is also present in brain, associated with cortico‐thalamic motor circuits [http://www.ncbi.nlm.nih.gov/pubmed/18706979?dopt=AbstractPlus]. cAMP elevation is the major signalling pathway for both RXFP1 and RXFP2 [http://www.ncbi.nlm.nih.gov/pubmed/10935549?dopt=AbstractPlus, http://www.ncbi.nlm.nih.gov/pubmed/11809971?dopt=AbstractPlus], but RXFP1 also activates MAP kinases, nitric oxide signalling, and tyrosine kinase phosphorylation; and relaxin can interact with glucocorticoid receptors [http://www.ncbi.nlm.nih.gov/pubmed/17293890?dopt=AbstractPlus]. Receptor expression profiles suggest that RXFP3 is a brain neuropeptide receptor and RXFP4 a gut hormone receptor. The brain relaxin‐3/RXFP3 system modulates feeding [http://www.ncbi.nlm.nih.gov/pubmed/23135160?dopt=AbstractPlus, http://www.ncbi.nlm.nih.gov/pubmed/22854307?dopt=AbstractPlus, http://www.ncbi.nlm.nih.gov/pubmed/21384867?dopt=AbstractPlus, http://www.ncbi.nlm.nih.gov/pubmed/22257012?dopt=AbstractPlus, http://www.ncbi.nlm.nih.gov/pubmed/21899720?dopt=AbstractPlus] *via* effects in hypothalamus [http://www.ncbi.nlm.nih.gov/pubmed/28864207?dopt=AbstractPlus, http://www.ncbi.nlm.nih.gov/pubmed/23135160?dopt=AbstractPlus, http://www.ncbi.nlm.nih.gov/pubmed/28098344?dopt=AbstractPlus], anxiety [http://www.ncbi.nlm.nih.gov/pubmed/24297931?dopt=AbstractPlus, http://www.ncbi.nlm.nih.gov/pubmed/26057358?dopt=AbstractPlus], reward and motivated, goal‐directed behaviours [http://www.ncbi.nlm.nih.gov/pubmed/25257104?dopt=AbstractPlus, http://www.ncbi.nlm.nih.gov/pubmed/24297931?dopt=AbstractPlus, http://www.ncbi.nlm.nih.gov/pubmed/25849482?dopt=AbstractPlus], and spatial and social memory [http://www.ncbi.nlm.nih.gov/pubmed/30368554?dopt=AbstractPlus, http://www.ncbi.nlm.nih.gov/pubmed/28100033?dopt=AbstractPlus]. Of the other relaxin peptides, http://www.guidetopharmacology.org/GRAC/LigandDisplayForward?ligandId=1990 (https://www.genenames.org/data/gene‐symbol‐report/#!/hgnc_id/HGNC:17135, http://www.uniprot.org/uniprot/Q8WXF3)is anagonist atRXFP3 and RXFP4 whereashttp://www.guidetopharmacology.org/GRAC/LigandDisplayForward?ligandId=2000 (https://www.genenames.org/data/gene‐symbol‐report/#!/hgnc_id/HGNC:6088, http://www.uniprot.org/uniprot/Q9Y5Q6) is an agonist at RXFP4 and a weak antagonist at RXFP3. http://www.guidetopharmacology.org/GRAC/LigandDisplayForward?ligandId=2000 (https://www.genenames.org/data/gene‐symbol‐report/#!/hgnc_id/HGNC:6088, http://www.uniprot.org/uniprot/Q9Y5Q6) is secreted from enteroendocrine L cells and the INSL5/RXFP4 system affects food intake [http://www.ncbi.nlm.nih.gov/pubmed/25028498?dopt=AbstractPlus] and glucose homeostasis [http://www.ncbi.nlm.nih.gov/pubmed/25514935?dopt=AbstractPlus]. RXFP3 and RXFP4 couple to G_i/o_ and inhibit adenylyl cyclase [http://www.ncbi.nlm.nih.gov/pubmed/14522968?dopt=AbstractPlus, http://www.ncbi.nlm.nih.gov/pubmed/20159943?dopt=AbstractPlus], and also cause Erk1/2 phosphorylation [http://www.ncbi.nlm.nih.gov/pubmed/20159943?dopt=AbstractPlus]. RXFP4 also causes phosphorylation of p38MAPK, Akt and S6RP [http://www.ncbi.nlm.nih.gov/pubmed/27243554?dopt=AbstractPlus] and GLP‐1 secretion *in vitro* [http://www.ncbi.nlm.nih.gov/pubmed/29535183?dopt=AbstractPlus]. There is evidence that at RXFP3, http://www.guidetopharmacology.org/GRAC/LigandDisplayForward?ligandId=1989 (https://www.genenames.org/data/gene‐symbol‐report/#!/hgnc_id/HGNC:10027, http://www.uniprot.org/uniprot/P04090) is a biased ligand compared to the cognate ligand http://www.guidetopharmacology.org/GRAC/LigandDisplayForward?ligandId=1990 (https://www.genenames.org/data/gene‐symbol‐report/#!/hgnc_id/HGNC:17135, http://www.uniprot.org/uniprot/Q8WXF3) [http://www.ncbi.nlm.nih.gov/pubmed/20159943?dopt=AbstractPlus].

### Further reading on Relaxin family peptide receptors

Bathgate RA *et al.* (2013) Relaxin family peptides and their receptors. *Physiol. Rev.* **93:** 405‐80 https://www.ncbi.nlm.nih.gov/pubmed/23303914?dopt=AbstractPlus

Du XJ *et al.* (2010) Cardiovascular effects of relaxin: from basic science to clinical therapy. *Nat Rev Cardiol* **7:** 48‐58 https://www.ncbi.nlm.nih.gov/pubmed/19935741?dopt=AbstractPlus

Halls ML *et al.* (2015) International Union of Basic and Clinical Pharmacology. XCV. Recent advances in the understanding of the pharmacology and biological roles of relaxin family peptide receptors 1‐4, the receptors for relaxin family peptides. *Pharmacol. Rev.* **67:** 389‐440 https://www.ncbi.nlm.nih.gov/pubmed/25761609?dopt=AbstractPlus

Ivell R *etal.* (2011) Relaxin family peptides in the male reproductive system‐a critical appraisal. *Mol. Hum. Reprod.* **17:** 71‐84 https://www.ncbi.nlm.nih.gov/pubmed/20952422?dopt=AbstractPlus

## http://www.guidetopharmacology.org/GRAC/FamilyDisplayForward?familyId=61

### Overview

Somatostatin (somatotropin release inhibiting factor) is an abundant neuropeptide, which acts on five subtypes of somatostatin receptor (SST_1_‐SST_5_; **nomenclature as agreed by the NC‐IUPHAR Subcommittee on Somatostatin Receptors** [http://www.ncbi.nlm.nih.gov/pubmed/30232095?dopt=AbstractPlus]). Activation of these receptors produces a wide range of physiological effects throughout the body including the inhibition of secretion of many hormones. Endogenous ligands for these receptors are somatostatin‐14 (http://www.guidetopharmacology.org/GRAC/LigandDisplayForward?ligandId=2019 (https://www.genenames.org/data/gene‐symbol‐report/#!/hgnc_id/HGNC:11329, http://www.uniprot.org/uniprot/P61278)) and somatostatin‐28 (http://www.guidetopharmacology.org/GRAC/LigandDisplayForward?ligandId=2020 (https://www.genenames.org/data/gene‐symbol‐report/#!/hgnc_id/HGNC:11329, http://www.uniprot.org/uniprot/P61278)). http://www.guidetopharmacology.org/GRAC/LigandDisplayForward?ligandId=2007 {Mouse, Rat} has also been suggested to be an endogenous ligand for somatostatin receptors [http://www.ncbi.nlm.nih.gov/pubmed/8622767?dopt=AbstractPlus].

**Table:** 

Nomenclature	http://www.guidetopharmacology.org/GRAC/ObjectDisplayForward?objectId=355	http://www.guidetopharmacology.org/GRAC/ObjectDisplayForward?objectId=356	http://www.guidetopharmacology.org/GRAC/ObjectDisplayForward?objectId=357	http://www.guidetopharmacology.org/GRAC/ObjectDisplayForward?objectId=358	http://www.guidetopharmacology.org/GRAC/ObjectDisplayForward?objectId=359
HGNC, UniProt	https://www.genenames.org/data/gene‐symbol‐report/#!/hgnc_id/HGNC:11330, http://www.uniprot.org/uniprot/P30872	https://www.genenames.org/data/gene‐symbol‐report/#!/hgnc_id/HGNC:11331, http://www.uniprot.org/uniprot/P30874	https://www.genenames.org/data/gene‐symbol‐report/#!/hgnc_id/HGNC:11332, http://www.uniprot.org/uniprot/P32745	https://www.genenames.org/data/gene‐symbol‐report/#!/hgnc_id/HGNC:11333, http://www.uniprot.org/uniprot/P31391	https://www.genenames.org/data/gene‐symbol‐report/#!/hgnc_id/HGNC:11334, http://www.uniprot.org/uniprot/P35346
Agonists	http://www.guidetopharmacology.org/GRAC/LigandDisplayForward?ligandId=2018 [http://www.ncbi.nlm.nih.gov/pubmed/15477717?dopt=AbstractPlus]	http://www.guidetopharmacology.org/GRAC/LigandDisplayForward?ligandId=2018 [http://www.ncbi.nlm.nih.gov/pubmed/15477717?dopt=AbstractPlus], http://www.guidetopharmacology.org/GRAC/LigandDisplayForward?ligandId=8487 [http://www.ncbi.nlm.nih.gov/pubmed/11145612?dopt=AbstractPlus]	http://www.guidetopharmacology.org/GRAC/LigandDisplayForward?ligandId=2018 [http://www.ncbi.nlm.nih.gov/pubmed/15477717?dopt=AbstractPlus]	http://www.guidetopharmacology.org/GRAC/LigandDisplayForward?ligandId=3513 [http://www.ncbi.nlm.nih.gov/pubmed/9822540?dopt=AbstractPlus], http://www.guidetopharmacology.org/GRAC/LigandDisplayForward?ligandId=8487 [http://www.ncbi.nlm.nih.gov/pubmed/11145612?dopt=AbstractPlus]	http://www.guidetopharmacology.org/GRAC/LigandDisplayForward?ligandId=2018 [http://www.ncbi.nlm.nih.gov/pubmed/15477717?dopt=AbstractPlus], http://www.guidetopharmacology.org/GRAC/LigandDisplayForward?ligandId=8487 [http://www.ncbi.nlm.nih.gov/pubmed/11145612?dopt=AbstractPlus]
Selective agonists	http://www.guidetopharmacology.org/GRAC/LigandDisplayForward?ligandId=2015 [http://www.ncbi.nlm.nih.gov/pubmed/9784130?dopt=AbstractPlus], http://www.guidetopharmacology.org/GRAC/LigandDisplayForward?ligandId=2010 [http://www.ncbi.nlm.nih.gov/pubmed/15658864?dopt=AbstractPlus]	http://www.guidetopharmacology.org/GRAC/LigandDisplayForward?ligandId=2046 [http://www.ncbi.nlm.nih.gov/pubmed/9724791?dopt=AbstractPlus], http://www.guidetopharmacology.org/GRAC/LigandDisplayForward?ligandId=2033 [http://www.ncbi.nlm.nih.gov/pubmed/8646408?dopt=AbstractPlus], http://www.guidetopharmacology.org/GRAC/LigandDisplayForward?ligandId=2050 [http://www.ncbi.nlm.nih.gov/pubmed/9784130?dopt=AbstractPlus], http://www.guidetopharmacology.org/GRAC/LigandDisplayForward?ligandId=2055 [http://www.ncbi.nlm.nih.gov/pubmed/8769372?dopt=AbstractPlus, http://www.ncbi.nlm.nih.gov/pubmed/7988476?dopt=AbstractPlus, http://www.ncbi.nlm.nih.gov/pubmed/9652348?dopt=AbstractPlus, http://www.ncbi.nlm.nih.gov/pubmed/10598788?dopt=AbstractPlus, http://www.ncbi.nlm.nih.gov/pubmed/9650799?dopt=AbstractPlus, http://www.ncbi.nlm.nih.gov/pubmed/9724791?dopt=AbstractPlus], http://www.guidetopharmacology.org/GRAC/LigandDisplayForward?ligandId=2031 [http://www.ncbi.nlm.nih.gov/pubmed/8769372?dopt=AbstractPlus, http://www.ncbi.nlm.nih.gov/pubmed/7988476?dopt=AbstractPlus, http://www.ncbi.nlm.nih.gov/pubmed/9652348?dopt=AbstractPlus, http://www.ncbi.nlm.nih.gov/pubmed/10598788?dopt=AbstractPlus, http://www.ncbi.nlm.nih.gov/pubmed/9650799?dopt=AbstractPlus]	http://www.guidetopharmacology.org/GRAC/LigandDisplayForward?ligandId=2073 [http://www.ncbi.nlm.nih.gov/pubmed/9784130?dopt=AbstractPlus]	http://www.guidetopharmacology.org/GRAC/LigandDisplayForward?ligandId=2082 [http://www.ncbi.nlm.nih.gov/pubmed/9784130?dopt=AbstractPlus], http://www.guidetopharmacology.org/GRAC/LigandDisplayForward?ligandId=10115 [http://www.ncbi.nlm.nih.gov/pubmed/15333679?dopt=AbstractPlus]	http://www.guidetopharmacology.org/GRAC/LigandDisplayForward?ligandId=2004 [http://www.ncbi.nlm.nih.gov/pubmed/9600011?dopt=AbstractPlus, http://www.ncbi.nlm.nih.gov/pubmed/9652348?dopt=AbstractPlus, http://www.ncbi.nlm.nih.gov/pubmed/10598788?dopt=AbstractPlus, http://www.ncbi.nlm.nih.gov/pubmed/9650799?dopt=AbstractPlus], http://www.guidetopharmacology.org/GRAC/LigandDisplayForward?ligandId=2016 [http://www.ncbi.nlm.nih.gov/pubmed/9784130?dopt=AbstractPlus]
Selective antagonists	http://www.guidetopharmacology.org/GRAC/LigandDisplayForward?ligandId=2030 (p*K*_d_ 8–8.1) [http://www.ncbi.nlm.nih.gov/pubmed/15135911?dopt=AbstractPlus]	http://www.guidetopharmacology.org/GRAC/LigandDisplayForward?ligandId=10113 [http://www.ncbi.nlm.nih.gov/pubmed/18543899?dopt=AbstractPlus]	http://www.guidetopharmacology.org/GRAC/LigandDisplayForward?ligandId=10114 (pIC_50_ 9.2) [http://www.ncbi.nlm.nih.gov/pubmed/24900499?dopt=AbstractPlus], http://www.guidetopharmacology.org/GRAC/LigandDisplayForward?ligandId=2076 (p*K*_i_ 7.9) [917]	–	http://www.guidetopharmacology.org/GRAC/LigandDisplayForward?ligandId=10116 (p*K*_i_ 9.3) [http://www.ncbi.nlm.nih.gov/pubmed/28938487?dopt=AbstractPlus]

### Comments

[http://www.guidetopharmacology.org/GRAC/LigandDisplayForward?ligandId=2060, [http://www.guidetopharmacology.org/GRAC/LigandDisplayForward?ligandId=2023, [http://www.guidetopharmacology.org/GRAC/LigandDisplayForward?ligandId=2022 and [http://www.guidetopharmacology.org/GRAC/LigandDisplayForward?ligandId=2024 may be used to label somatostatin receptors nonselectively. A number of nonpeptide subtype‐selective agonists have been synthesised [http://www.ncbi.nlm.nih.gov/pubmed/9784130?dopt=AbstractPlus]. Octreotide and lanreotide are being used in the treatment of SST_2_‐expressing neuroendocrine tumors and pasireotide for SST_5_‐expressing neuroendocrine tumors. A novel peptide somatostatin analogue, veldoreotide (COR‐005), has affinity for SST_2_, SST_4_ and SST_5_ receptors and is a potent inhibitor of GH secretion [http://www.ncbi.nlm.nih.gov/pubmed/22065857?dopt=AbstractPlus, http://www.ncbi.nlm.nih.gov/pubmed/15636423?dopt=AbstractPlus].

### Further reading on Somatostatin receptors

Colao A *et al.* (2011) Resistance to somatostatin analogs in acromegaly. *Endocr. Rev.* **32:** 247‐71 https://www.ncbi.nlm.nih.gov/pubmed/21123741?dopt=AbstractPlus

Günther T *et al.* (2018) International Union of Basic and Clinical Pharmacology. CV. Somatostatin Receptors: Structure, Function, Ligands, and New Nomenclature. *Pharmacol. Rev.* **70:** 763‐835 https://www.ncbi.nlm.nih.gov/pubmed/30232095?dopt=AbstractPlus

Hoyer D *et al.* (2000) Somatostatin receptors. *In The IUPHAR Compendium of Receptor Characterization and Classification, 2nd edn.* Edited by Watson SP, Girdlestone D: IUPHAR Media: 354‐364

Schulz S *et al.* (2014) Fine‐tuning somatostatin receptor signalling by agonist‐selective phosphorylation and dephosphorylation: IUPHAR Review 5. *Br. J. Pharmacol.* **171:** 1591‐9 https://www.ncbi.nlm.nih.gov/pubmed/24328848?dopt=AbstractPlus

Weckbecker G *et al.* (2003) Opportunities in somatostatin research: biological, chemical and therapeutic aspects. *Nat Rev Drug Discov* **2:** 999‐1017 https://www.ncbi.nlm.nih.gov/pubmed/14654798?dopt=AbstractPlus

## http://www.guidetopharmacology.org/GRAC/FamilyDisplayForward?familyId=446

### Overview

**Nomenclature as recommended by NC‐IUPHAR** [http://www.ncbi.nlm.nih.gov/pubmed/23686350?dopt=AbstractPlus]. The Succinate receptor has been identified as being activated by physiological levels of the Kreb's cycle intermediate succinate and other dicarboxylic acids such as maleate in 2004. Since its pairing with its endogenous ligand, the receptor has been the focus of intensive research and its role has been evidenced in various (patho)physiological processes such as regulation of renin production, retinal angiogenesis, inflammation or immune response.

**Table:** 

Nomenclature	http://www.guidetopharmacology.org/GRAC/ObjectDisplayForward?objectId=166
HGNC, UniProt	https://www.genenames.org/data/gene‐symbol‐report/#!/hgnc_id/HGNC:4542, http://www.uniprot.org/uniprot/Q9BXA5
Endogenous agonists	http://www.guidetopharmacology.org/GRAC/LigandDisplayForward?ligandId=3637 [http://www.ncbi.nlm.nih.gov/pubmed/15141213?dopt=AbstractPlus, http://www.ncbi.nlm.nih.gov/pubmed/23396314?dopt=AbstractPlus], http://www.guidetopharmacology.org/GRAC/LigandDisplayForward?ligandId=9630 [http://www.ncbi.nlm.nih.gov/pubmed/28160606?dopt=AbstractPlus]
Agonists	http://www.guidetopharmacology.org/GRAC/LigandDisplayForward?ligandId=10296 (Partial agonist) [http://www.ncbi.nlm.nih.gov/pubmed/29968758?dopt=AbstractPlus], http://www.guidetopharmacology.org/GRAC/LigandDisplayForward?ligandId=9631 [http://www.ncbi.nlm.nih.gov/pubmed/29157600?dopt=AbstractPlus], http://www.guidetopharmacology.org/GRAC/LigandDisplayForward?ligandId=10294 (Partial agonist) [http://www.ncbi.nlm.nih.gov/pubmed/29157600?dopt=AbstractPlus], http://www.guidetopharmacology.org/GRAC/LigandDisplayForward?ligandId=9631 [http://www.ncbi.nlm.nih.gov/pubmed/28160606?dopt=AbstractPlus], http://www.guidetopharmacology.org/GRAC/LigandDisplayForward?ligandId=9630 [http://www.ncbi.nlm.nih.gov/pubmed/26386312?dopt=AbstractPlus], http://www.guidetopharmacology.org/GRAC/LigandDisplayForward?ligandId=9630 [http://www.ncbi.nlm.nih.gov/pubmed/15141213?dopt=AbstractPlus]

### Comments

In humans, there is the possibility of two open‐reading frames (ORFs) for *SUCNR1*, one giving a protein of 330 amino acids (AA) and the other one 334‐AA. Wittenberger *et al.* [http://www.ncbi.nlm.nih.gov/pubmed/11273702?dopt=AbstractPlus] noted that the 330‐AA protein was more likely to be expressed given the Kozak sequence surrounding the second ATG. Some databases report *SUCNR1* as being 334‐AA long.

### Further reading on Succinate receptor

Ariza AC *et al.* (2012) The succinate receptor as a novel therapeutic target for oxidative and metabolic stress‐related conditions. *Front Endocrinol (Lausanne)* **3**:22 [https://www.ncbi.nlm.nih.gov/pubmed/22649411?dopt=AbstractPlus]

de Castro Fonseca M *et al.* (2016) GPR91: expanding the frontiers of Krebs cycle intermediates. *Cell Commun. Signal* **14**:3[https://www.ncbi.nlm.nih.gov/pubmed/26759054?dopt=AbstractPlus]

Gilissen J *et al.* (2016) Insight into SUCNR1 (GPR91) structure and function. *Pharmacol. Ther.* **159:** 56‐65[https://www.ncbi.nlm.nih.gov/pubmed/26808164?dopt=AbstractPlus]

Grimolizzi F *et al.* (2018) Multiple faces of succinate beyond metabolism in blood. *Haematologica* **103:** 1586‐1592[https://www.ncbi.nlm.nih.gov/pubmed/29954939?dopt=AbstractPlus]

## http://www.guidetopharmacology.org/GRAC/FamilyDisplayForward?familyId=62

### Overview

Tachykinin receptors (**provisional nomenclature as recommended by NC‐IUPHAR** [http://www.ncbi.nlm.nih.gov/pubmed/15914470?dopt=AbstractPlus]) are activated by the endogenous peptides http://www.guidetopharmacology.org/GRAC/LigandDisplayForward?ligandId=2098 (https://www.genenames.org/data/gene‐symbol‐report/#!/hgnc_id/HGNC:11517, http://www.uniprot.org/uniprot/P20366) (SP), http://www.guidetopharmacology.org/GRAC/LigandDisplayForward?ligandId=2089) (NKA; previously known as substance K, neurokinin α, neuromedin L), http://www.guidetopharmacology.org/GRAC/LigandDisplayForward?ligandId=2090 (https://www.genenames.org/data/gene‐symbol‐report/#!/hgnc_id/HGNC:11521, http://www.uniprot.org/uniprot/Q9UHF0) (NKB; previously known as neurokinin β, neuromedin K), http://www.guidetopharmacology.org/GRAC/LigandDisplayForward?ligandId=2091 (https://www.genenames.org/data/gene‐symbol‐report/#!/hgnc_id/HGNC:11517, http://www.uniprot.org/uniprot/P20366) and http://www.guidetopharmacology.org/GRAC/LigandDisplayForward?ligandId=3667 (https://www.genenames.org/data/gene‐symbol‐report/#!/hgnc_id/HGNC:11517) (N‐terminally extended forms of neurokinin A). The neurokinins (A and B) are mammalian members of the tachykinin family, which includes peptides of mammalian and nonmammalian origin containing the consensus sequence: Phe‐x‐Gly‐LeuMet. Marked species differences in *in vitro* pharmacology exist for all three receptors, in the context of nonpeptide ligands. Antagonists such as http://www.guidetopharmacology.org/GRAC/LigandDisplayForward?ligandId=3490 and http://www.guidetopharmacology.org/GRAC/LigandDisplayForward?ligandId=7623 were approved by FDA and EMA, in combination with other antiemetic agents, for the prevention of nausea and vomiting associated with emetogenic cancer chemotherapy.

**Table:** 

Nomenclature	http://www.guidetopharmacology.org/GRAC/ObjectDisplayForward?objectId=360	http://www.guidetopharmacology.org/GRAC/ObjectDisplayForward?objectId=361	http://www.guidetopharmacology.org/GRAC/ObjectDisplayForward?objectId=362
HGNC, UniProt	https://www.genenames.org/data/gene‐symbol‐report/#!/hgnc_id/HGNC:11526, http://www.uniprot.org/uniprot/P25103	https://www.genenames.org/data/gene‐symbol‐report/#!/hgnc_id/HGNC:11527, http://www.uniprot.org/uniprot/P21452	https://www.genenames.org/data/gene‐symbol‐report/#!/hgnc_id/HGNC:11528, http://www.uniprot.org/uniprot/P29371
Potency order of endogenous ligands	http://www.guidetopharmacology.org/GRAC/LigandDisplayForward?ligandId=2098 (https://www.genenames.org/data/gene‐symbol‐report/#!/hgnc_id/HGNC:11517, http://www.uniprot.org/uniprot/P20366) > http://www.guidetopharmacology.org/GRAC/LigandDisplayForward?ligandId=2089) > http://www.guidetopharmacology.org/GRAC/LigandDisplayForward?ligandId=2090 (https://www.genenames.org/data/gene‐symbol‐report/#!/hgnc_id/HGNC:11521, http://www.uniprot.org/uniprot/Q9UHF0)	http://www.guidetopharmacology.org/GRAC/LigandDisplayForward?ligandId=2089 (https://www.genenames.org/data/gene‐symbol‐report/#!/hgnc_id/HGNC:11517, http://www.uniprot.org/uniprot/P20366) > http://www.guidetopharmacology.org/GRAC/LigandDisplayForward?ligandId=2090 (https://www.genenames.org/data/gene‐symbol‐report/#!/hgnc_id/HGNC:11521, http://www.uniprot.org/uniprot/Q9UHF0) http://www.guidetopharmacology.org/GRAC/LigandDisplayForward?ligandId=2098)	http://www.guidetopharmacology.org/GRAC/LigandDisplayForward?ligandId=2090 (https://www.genenames.org/data/gene‐symbol‐report/#!/hgnc_id/HGNC:11521, http://www.uniprot.org/uniprot/Q9UHF0) > http://www.guidetopharmacology.org/GRAC/LigandDisplayForward?ligandId=2089 (https://www.genenames.org/data/gene‐symbol‐report/#!/hgnc_id/HGNC:11517, http://www.uniprot.org/uniprot/P20366) > http://www.guidetopharmacology.org/GRAC/LigandDisplayForward?ligandId=2098)
Agonists	http://www.guidetopharmacology.org/GRAC/LigandDisplayForward?ligandId=2101 [http://www.ncbi.nlm.nih.gov/pubmed/8702757?dopt=AbstractPlus]	–	–
Selective agonists	[http://www.guidetopharmacology.org/GRAC/LigandDisplayForward?ligandId=3850 [http://www.ncbi.nlm.nih.gov/pubmed/8702757?dopt=AbstractPlus], http://www.guidetopharmacology.org/GRAC/LigandDisplayForward?ligandId=2095 [http://www.ncbi.nlm.nih.gov/pubmed/11786503?dopt=AbstractPlus, http://www.ncbi.nlm.nih.gov/pubmed/8985159?dopt=AbstractPlus], [http://www.guidetopharmacology.org/GRAC/LigandDisplayForward?ligandId=3849 [http://www.ncbi.nlm.nih.gov/pubmed/9243521?dopt=AbstractPlus] – Rat	[http://www.guidetopharmacology.org/GRAC/LigandDisplayForward?ligandId=3843) [http://www.ncbi.nlm.nih.gov/pubmed/9718274?dopt=AbstractPlus] – Rat, http://www.guidetopharmacology.org/GRAC/LigandDisplayForward?ligandId=3881 [http://www.ncbi.nlm.nih.gov/pubmed/1331460?dopt=AbstractPlus] – Rat, [http://www.guidetopharmacology.org/GRAC/LigandDisplayForward?ligandId=2112) [http://www.ncbi.nlm.nih.gov/pubmed/7682062?dopt=AbstractPlus]	[http://www.guidetopharmacology.org/GRAC/LigandDisplayForward?ligandId=2113 [http://www.ncbi.nlm.nih.gov/pubmed/11226387?dopt=AbstractPlus, http://www.ncbi.nlm.nih.gov/pubmed/9190866?dopt=AbstractPlus], http://www.guidetopharmacology.org/GRAC/LigandDisplayForward?ligandId=2127, http://www.ncbi.nlm.nih.gov/pubmed/9190866?dopt=AbstractPlus, http://www.ncbi.nlm.nih.gov/pubmed/8702757?dopt=AbstractPlus]
Antagonists	http://www.guidetopharmacology.org/GRAC/LigandDisplayForward?ligandId=10349 (pIC_50_ 9.7) [http://www.ncbi.nlm.nih.gov/pubmed/11708932?dopt=AbstractPlus]	–	–
Selective antagonists	http://www.guidetopharmacology.org/GRAC/LigandDisplayForward?ligandId=3490 (p*K*_i_ 10.1) [http://www.ncbi.nlm.nih.gov/pubmed/10737756?dopt=AbstractPlus, http://www.ncbi.nlm.nih.gov/pubmed/9804700?dopt=AbstractPlus], http://www.guidetopharmacology.org/GRAC/LigandDisplayForward?ligandId=2102 (p*K*i 9.3–9.7) [http://www.ncbi.nlm.nih.gov/pubmed/12206858?dopt=AbstractPlus, http://www.ncbi.nlm.nih.gov/pubmed/9190866?dopt=AbstractPlus], http://www.guidetopharmacology.org/GRAC/LigandDisplayForward?ligandId=3522 (pIC_50_ 7.7) [http://www.ncbi.nlm.nih.gov/pubmed/1281470?dopt=AbstractPlus]	http://www.guidetopharmacology.org/GRAC/LigandDisplayForward?ligandId=3882 (p*K*_i_ 9.8) [http://www.ncbi.nlm.nih.gov/pubmed/8027981?dopt=AbstractPlus], http://www.guidetopharmacology.org/GRAC/LigandDisplayForward?ligandId=2111 (p*K*_i_ 9.4–9.7) [http://www.ncbi.nlm.nih.gov/pubmed/12206858?dopt=AbstractPlus, http://www.ncbi.nlm.nih.gov/pubmed/7682062?dopt=AbstractPlus, http://www.ncbi.nlm.nih.gov/pubmed/9190866?dopt=AbstractPlus], http://www.guidetopharmacology.org/GRAC/LigandDisplayForward?ligandId=2115 (p*K*_d_ 7.8–9.5) [http://www.ncbi.nlm.nih.gov/pubmed/7713168?dopt=AbstractPlus, http://www.ncbi.nlm.nih.gov/pubmed/7562907?dopt=AbstractPlus], http://www.guidetopharmacology.org/GRAC/LigandDisplayForward?ligandId=3901 (p*K*_i_ 9.2) [http://www.ncbi.nlm.nih.gov/pubmed/11063600?dopt=AbstractPlus], http://www.guidetopharmacology.org/GRAC/LigandDisplayForward?ligandId=2123 (p*K*i 8.5–8.7) [http://www.ncbi.nlm.nih.gov/pubmed/9484857?dopt=AbstractPlus, http://www.ncbi.nlm.nih.gov/pubmed/16979621?dopt=AbstractPlus]	http://www.guidetopharmacology.org/GRAC/LigandDisplayForward?ligandId=2110 (p*K*_i_ 8.4–9.7) [http://www.ncbi.nlm.nih.gov/pubmed/12206858?dopt=AbstractPlus, http://www.ncbi.nlm.nih.gov/pubmed/9042606?dopt=AbstractPlus, http://www.ncbi.nlm.nih.gov/pubmed/7476898?dopt=AbstractPlus, http://www.ncbi.nlm.nih.gov/pubmed/7830490?dopt=AbstractPlus, http://www.ncbi.nlm.nih.gov/pubmed/11757797?dopt=AbstractPlus, http://www.ncbi.nlm.nih.gov/pubmed/7616392?dopt=AbstractPlus, http://www.ncbi.nlm.nih.gov/pubmed/11226387?dopt=AbstractPlus, http://www.ncbi.nlm.nih.gov/pubmed/9190866?dopt=AbstractPlus, http://www.ncbi.nlm.nih.gov/pubmed/8702757?dopt=AbstractPlus], http://www.guidetopharmacology.org/GRAC/LigandDisplayForward?ligandId=2132 (p*K*_i_ 7.4–9) [http://www.ncbi.nlm.nih.gov/pubmed/15265501?dopt=AbstractPlus, http://www.ncbi.nlm.nih.gov/pubmed/8691422?dopt=AbstractPlus, http://www.ncbi.nlm.nih.gov/pubmed/11226387?dopt=AbstractPlus, http://www.ncbi.nlm.nih.gov/pubmed/9190866?dopt=AbstractPlus], http://www.guidetopharmacology.org/GRAC/LigandDisplayForward?ligandId=2125 (pIC_50_ 7.8–7.9) [http://www.ncbi.nlm.nih.gov/pubmed/8648606?dopt=AbstractPlus, http://www.ncbi.nlm.nih.gov/pubmed/8702757?dopt=AbstractPlus]
Labelled ligands	[http://www.guidetopharmacology.org/GRAC/LigandDisplayForward?ligandId=3471 (Antagonist) (p*K*_d_ 9.5) [http://www.ncbi.nlm.nih.gov/pubmed/8287060?dopt=AbstractPlus], [http://www.guidetopharmacology.org/GRAC/LigandDisplayForward?ligandId=3769 (Agonist) [http://www.ncbi.nlm.nih.gov/pubmed/1705465?dopt=AbstractPlus] – Rat, [http://www.guidetopharmacology.org/GRAC/LigandDisplayForward?ligandId=3835) (Agonist) [http://www.ncbi.nlm.nih.gov/pubmed/2410593?dopt=AbstractPlus], [http://www.guidetopharmacology.org/GRAC/LigandDisplayForward?ligandId=3805) (Agonist), [http://www.guidetopharmacology.org/GRAC/LigandDisplayForward?ligandId=3473 (Antagonist) [367]	[http://www.guidetopharmacology.org/GRAC/LigandDisplayForward?ligandId=3481 (Antagonist) (p*K*_d_ 9.7) [http://www.ncbi.nlm.nih.gov/pubmed/7719707?dopt=AbstractPlus] – Rat, [http://www.guidetopharmacology.org/GRAC/LigandDisplayForward?ligandId=3795) (Agonist) [http://www.ncbi.nlm.nih.gov/pubmed/10455255?dopt=AbstractPlus], [http://www.guidetopharmacology.org/GRAC/LigandDisplayForward?ligandId=3822 (Antagonist) (p*K*_d_ 9.2) [http://www.ncbi.nlm.nih.gov/pubmed/8210508?dopt=AbstractPlus]	[http://www.guidetopharmacology.org/GRAC/LigandDisplayForward?ligandId=3480 (Antagonist) (p*K*_d_ 9.9), [http://www.guidetopharmacology.org/GRAC/LigandDisplayForward?ligandId=3834 (Agonist) [http://www.ncbi.nlm.nih.gov/pubmed/1694464?dopt=AbstractPlus] – Guinea pig, [http://www.guidetopharmacology.org/GRAC/LigandDisplayForward?ligandId=3763 (Agonist)

### Comments

The NK_1_ receptor has also been described to couple to G proteins other than G_q/11_ [http://www.ncbi.nlm.nih.gov/pubmed/9654151?dopt=AbstractPlus]. The crystal structure of the human NK_1_ receptor in complex with antagonists has been determined [http://www.ncbi.nlm.nih.gov/pubmed/30604743?dopt=AbstractPlus, http://www.ncbi.nlm.nih.gov/pubmed/30538204?dopt=AbstractPlus]. The hexapeptide agonist septide appears to bind to an overlapping but non‐identical site to http://www.guidetopharmacology.org/GRAC/LigandDisplayForward?ligandId=2098 (https://www.genenames.org/data/gene‐symbol‐report/%23!/hgnc_id/HGNC:11517, http://www.uniprot.org/uniprot/P20366) on the NK_1_ receptor. There are additional subtypes of tachykinin receptor;an orphan receptor (Swis‐sProt http://www.ncbi.nlm.nih.gov/protein/266702/) with structural similarities to the NK_3_ receptor was found to respond to NKB when expressed in *Xenopus* oocytes or Chinese hamster ovary cells [http://www.ncbi.nlm.nih.gov/pubmed/8947459?dopt=AbstractPlus, http://www.ncbi.nlm.nih.gov/pubmed/8990205?dopt=AbstractPlus].

### Further reading on Tachykinin receptors

Douglas SD *et al.* (2011) Neurokinin‐1 receptor: functional significance in the immune system in reference to selected infections and inflammation. *Ann. N. Y. Acad. Sci.* **1217:** 83‐95[https://www.ncbi.nlm.nih.gov/pubmed/21091716?dopt=AbstractPlus]

Foord SM *et al.* (2005) International Union of Pharmacology. XLVI. G protein‐coupled receptor list. *Pharmacol Rev* **57:** 279‐288[https://www.ncbi.nlm.nih.gov/pubmed/15914470?dopt=AbstractPlus]

Jones S *et al.* (2008) The neurokinin 1 receptor: a potential new target for anti‐platelet therapy? *Curr Opin Pharmacol* **8:** 114‐9[https://www.ncbi.nlm.nih.gov/pubmed/18296119?dopt=AbstractPlus]

Steinhoff MS *et al.* (2014) Tachykinins and their receptors: contributions to physiological control and the mechanisms of disease. *Physiol. Rev.* **94:** 265‐301[https://www.ncbi.nlm.nih.gov/pubmed/24382888?dopt=AbstractPlus]

Yin J *et al.* (2018) Crystal structure of the human NK_1 tachykinin receptor. *Proc. Natl. Acad. Sci. U.S.A.* **115:** 13264‐13269[https://www.ncbi.nlm.nih.gov/pubmed/30538204?dopt=AbstractPlus]

## http://www.guidetopharmacology.org/GRAC/FamilyDisplayForward?familyId=63

### Overview

Thyrotropin‐releasing hormone (TRH) receptors (**provisional nomenclature as recommended by NC‐IUPHAR** [http://www.ncbi.nlm.nih.gov/pubmed/15914470?dopt=AbstractPlus]) are activated by the endogenous tripeptide http://www.guidetopharmacology.org/GRAC/LigandDisplayForward?ligandId=2139 (https://www.genenames.org/data/gene‐symbol‐report/#!/hgnc_id/HGNC:12298, http://www.uniprot.org/uniprot/P20396) (pGlu‐His‐ProNH2). http://www.guidetopharmacology.org/GRAC/LigandDisplayForward?ligandId=2139 (https://www.genenames.org/data/gene‐symbol‐report/#!/hgnc_id/HGNC:12298, http://www.uniprot.org/uniprot/P20396) and TRH analogues fail to distinguish TRH_1_ and TRH_2_ receptors [http://www.ncbi.nlm.nih.gov/pubmed/12683933?dopt=AbstractPlus]. [http://www.guidetopharmacology.org/GRAC/LigandDisplayForward?ligandId=3836) is able to label both TRH_1_ and TRH_2_ receptors with K_d_ values of 13 and 9 nM respectively. Synthesis and biology of ring‐modified L‐Histidine containing TRH analogues has been reported [http://www.ncbi.nlm.nih.gov/pubmed/26854379?dopt=AbstractPlus].

**Table:** 

Nomenclature	http://www.guidetopharmacology.org/GRAC/ObjectDisplayForward?objectId=363	http://www.guidetopharmacology.org/GRAC/ObjectDisplayForward?objectId=754
HGNC, UniProt	https://www.genenames.org/data/gene‐symbol‐report/#!/hgnc_id/HGNC:12299, http://www.uniprot.org/uniprot/P34981	–
Antagonists	http://www.guidetopharmacology.org/GRAC/LigandDisplayForward?ligandId=3364 (p*K*_i_ 5.2) [http://www.ncbi.nlm.nih.gov/pubmed/2566295?dopt=AbstractPlus] – Rat	–
Selective antagonists	http://www.guidetopharmacology.org/GRAC/LigandDisplayForward?ligandId=3342 (p*K*_i_ 5.5) [http://www.ncbi.nlm.nih.gov/pubmed/2566295?dopt=AbstractPlus] – Rat, http://www.guidetopharmacology.org/GRAC/LigandDisplayForward?ligandId=3370 (p*K*_i_ 4.8) [http://www.ncbi.nlm.nih.gov/pubmed/2566295?dopt=AbstractPlus] – Rat, http://www.guidetopharmacology.org/GRAC/LigandDisplayForward?ligandId=3370 (p*K*_i_ 4.7) [http://www.ncbi.nlm.nih.gov/pubmed/2175902?dopt=AbstractPlus] – Mouse	–
Comments	–	A class A G protein‐coupled receptor: not present in man

### Further reading on Thyrotropin‐releasing hormone receptors

Bílek R *etal.* (2011) TRH‐like peptides. *Physiol Res* **60:** 207‐15[https://www.ncbi.nlm.nih.gov/pubmed/21114375?dopt=AbstractPlus]

Foord SM *et al.* (2005) International Union of Pharmacology. XLVI. G protein‐coupled receptor list. *Pharmacol Rev* **57:** 279‐288[https://www.ncbi.nlm.nih.gov/pubmed/15914470?dopt=AbstractPlus]

Nillni EA. (2010) Regulation of the hypothalamic thyrotropin releasing hormone (TRH) neuron by neuronal and peripheral inputs. *Front Neuroendocrinol* **31:** 134‐56[https://www.ncbi.nlm.nih.gov/pubmed/20074584?dopt=AbstractPlus]

## http://www.guidetopharmacology.org/GRAC/FamilyDisplayForward?familyId=64

### Overview

Trace amine‐associated receptors were discovered from a search for novel 5‐HT receptors [http://www.ncbi.nlm.nih.gov/pubmed/11459929?dopt=AbstractPlus], where 15 mammalian orthologues were identified and divided into two families. The TA_1_ receptor (**nomenclature as agreed by the** **NC‐IUPHAR Subcommittee for the Trace amine receptor** [http://www.ncbi.nlm.nih.gov/pubmed/19325074?dopt=AbstractPlus]) has affinity for the endogenous trace amines http://www.guidetopharmacology.org/GRAC/LigandDisplayForward?ligandId=2150, http://www.guidetopharmacology.org/GRAC/LigandDisplayForward?ligandId=2144 and http://www.guidetopharmacology.org/GRAC/LigandDisplayForward?ligandId=2149 in addition to the classical amine http://www.guidetopharmacology.org/GRAC/LigandDisplayForward?ligandId=940 [http://www.ncbi.nlm.nih.gov/pubmed/11459929?dopt=AbstractPlus]. Emerging evidence suggests that TA_1_ is a modulator of monoaminergic activity in the brain [http://www.ncbi.nlm.nih.gov/pubmed/19482011?dopt=AbstractPlus] with TA_1_ and dopamine D_2_ receptors shown to form constitutive heterodimers when co‐expressed [http://www.ncbi.nlm.nih.gov/pubmed/21670104?dopt=AbstractPlus]. In addition to trace amines, receptors can be activated by amphetamine‐like psychostimulants, and endogenous thyronamines.

**Table:** 

Nomenclature	http://www.guidetopharmacology.org/GRAC/ObjectDisplayForward?objectId=364
HGNC, UniProt	https://www.genenames.org/data/gene‐symbol‐report/#!/hgnc_id/HGNC:17734, http://www.uniprot.org/uniprot/Q96RJ0
Potency order of endogenous ligands	http://www.guidetopharmacology.org/GRAC/LigandDisplayForward?ligandId=2150 > http://www.guidetopharmacology.org/GRAC/LigandDisplayForward?ligandId=2144 > http://www.guidetopharmacology.org/GRAC/LigandDisplayForward?ligandId=2149 = http://www.guidetopharmacology.org/GRAC/LigandDisplayForward?ligandId=940 [http://www.ncbi.nlm.nih.gov/pubmed/11459929?dopt=AbstractPlus]
Agonists	http://www.guidetopharmacology.org/GRAC/LigandDisplayForward?ligandId=5862 [http://www.ncbi.nlm.nih.gov/pubmed/21525407?dopt=AbstractPlus]
Antagonists	http://www.guidetopharmacology.org/GRAC/LigandDisplayForward?ligandId=5457 (Inverse agonist) (pIC_50_ 5.1) [http://www.ncbi.nlm.nih.gov/pubmed/19892733?dopt=AbstractPlus]
Labelled ligands	[http://www.guidetopharmacology.org/GRAC/LigandDisplayForward?ligandId=2148 (Agonist) [http://www.ncbi.nlm.nih.gov/pubmed/11459929?dopt=AbstractPlus]

### Comments

In addition to TA_1_, in man there are up to 5 functional TAAR genes (TAAR2,5,6,8,9). See [http://www.ncbi.nlm.nih.gov/pubmed/11459929?dopt=AbstractPlus] for detailed discussion. The product of the gene TAAR2 (also known as GPR58) appears to respond to http://www.guidetopharmacology.org/GRAC/LigandDisplayForward?ligandId=2144 > http://www.guidetopharmacology.org/GRAC/LigandDisplayForward?ligandId=2150 and to couple through Gs [http://www.ncbi.nlm.nih.gov/pubmed/11459929?dopt=AbstractPlus]. http://www.guidetopharmacology.org/GRAC/FamilyDisplayForward?familyId=115#show_object_168, in some individuals, and http://www.guidetopharmacology.org/GRAC/FamilyDisplayForward?familyId=115#show_object_169 are pseudogenes in man, although functional in rodents. The signalling characteristics and pharmacology of http://www.guidetopharmacology.org/GRAC/FamilyDisplayForward?familyId=115#show_object_170 (PNR, Putative Neurotransmitter Receptor: https://www.genenames.org/data/gene‐symbol‐report/#!/hgnc_id/HGNC:30236, http://www.uniprot.org/uniprot/O14804), http://www.guidetopharmacology.org/GRAC/FamilyDisplayForward?familyId=115#show_object_171 http://www.guidetopharmacology.org/GRAC/FamilyDisplayForward?familyId=115#show_object_171 (Trace amine receptor 4, TaR‐4: https://www.genenames.org/data/gene‐symbol‐report/#!/hgnc_id/HGNC:20978, http://www.uniprot.org/uniprot/96RI8), http://www.guidetopharmacology.org/GRAC/FamilyDisplayForward?familyId=115#show_object_172 (Trace amine receptor 5, GPR102: https://www.genenames.org/data/gene‐symbol‐report/#!/hgnc_id/HGNC:14964, http://www.uniprot.org/uniprot/Q969N4) and http://www.guidetopharmacology.org/GRAC/FamilyDisplayForward?familyId=115#show_object_173 http://www.guidetopharmacology.org/GRAC/FamilyDisplayForward?familyId=115#show_object_173 (trace amine associated receptor 9: https://www.genenames.org/data/gene‐symbol‐report/#!/hgnc_id/HGNC:20977, http://www.uniprot.org/uniprot/96RI9) are lacking. The thyronamines, endogenous derivatives of thyroid hormone, have affinity for rodent cloned trace amine receptors, including TA_1_ [http://www.ncbi.nlm.nih.gov/pubmed/15146179?dopt=AbstractPlus]. An antagonist http://www.guidetopharmacology.org/GRAC/LigandDisplayForward?ligandId=5457 has recently been described with a *p*Ki of 9.1 at the mouse TA_1_ but >5.3 for human TA_1_ [http://www.ncbi.nlm.nih.gov/pubmed/21237643?dopt=AbstractPlus].

### Further reading on Trace amine receptor

Maguire JJ *et al*. (2009) International Union of Pharmacology. LXXII. Recommendations for trace amine receptor nomenclature. *Pharmacol. Rev.* **61**: 1‐8 [https://www.ncbi.nlm.nih.gov/pubmed/19325074?dopt=AbstractPlus]

Pei Y *et al*. (2016) Trace Amines and the Trace Amine‐Associated Receptor 1: Pharmacology, Neurochemistry, and Clinical Implications. *Front Neurosci* **10**: 148 [https://www.ncbi.nlm.nih.gov/pubmed/27092049?dopt=AbstractPlus]

## http://www.guidetopharmacology.org/GRAC/FamilyDisplayForward?familyId=65

### Overview

The urotensin‐II (U‐II) receptor (UT, **nomenclature as agreed by the NC‐IUPHAR Subcommittee on the Urotensin receptor** [516, http://www.ncbi.nlm.nih.gov/pubmed/15914470?dopt=AbstractPlus, http://www.ncbi.nlm.nih.gov/pubmed/25535277?dopt=AbstractPlus]) is activated by the endogenous dodecapeptide http://www.guidetopharmacology.org/GRAC/LigandDisplayForward?ligandId=2153 (https://www.genenames.org/data/gene‐symbol‐report/#!/hgnc_id/HGNC:12636, http://www.uniprot.org/uniprot/O95399), originally isolated from the urophysis, the endocrine organ of the caudal neurosecretory system of teleost fish [http://www.ncbi.nlm.nih.gov/pubmed/2864726?dopt=AbstractPlus, http://www.ncbi.nlm.nih.gov/pubmed/20633133?dopt=AbstractPlus]. Several structural forms of U‐II exist in fish and amphibians. The goby orthologue was used to identify U‐II as the cognate ligand for the predicted receptor encoded by the rat gene *gpr14* [http://www.ncbi.nlm.nih.gov/pubmed/9861051?dopt=AbstractPlus, http://www.ncbi.nlm.nih.gov/pubmed/10581185?dopt=AbstractPlus, http://www.ncbi.nlm.nih.gov/pubmed/10548501?dopt=AbstractPlus, http://www.ncbi.nlm.nih.gov/pubmed/10559967?dopt=AbstractPlus]. Human http://www.guidetopharmacology.org/GRAC/LigandDisplayForward?ligandId=2153), an 11‐amino‐acid peptide [http://www.ncbi.nlm.nih.gov/pubmed/9861051?dopt=AbstractPlus], retains the cyclohexapeptide sequence of goby U‐II that is thought to beimportant in ligand binding [http://www.ncbi.nlm.nih.gov/pubmed/12807997?dopt=AbstractPlus, http://www.ncbi.nlm.nih.gov/pubmed/12203418?dopt=AbstractPlus]. This sequence is also conserved in the deduced amino‐acid sequence of rat http://www.guidetopharmacology.org/GRAC/LigandDisplayForward?ligandId=2155 {Rat} (14 amino‐acids) and mouse http://www.guidetopharmacology.org/GRAC/LigandDisplayForward?ligandId=2154 {Mouse} (14 amino‐acids), although the N‐terminal is more divergent from the human sequence [http://www.ncbi.nlm.nih.gov/pubmed/10486557?dopt=AbstractPlus]. A second endogenous ligand for the UT has been discovered in rat [http://www.ncbi.nlm.nih.gov/pubmed/17628210?dopt=AbstractPlus]. This is the http://www.guidetopharmacology.org/GRAC/LigandDisplayForward?ligandId=2156 (https://www.genenames.org/data/gene‐symbol‐report/#!/hgnc_id/HGNC:30894, http://www.uniprot.org/uniprot/Q765I0), an octapeptide that is derived from a different gene, but shares the C‐terminal sequence (CFWKYCV) common to U‐II from other species. Identical sequences to rat http://www.guidetopharmacology.org/GRAC/LigandDisplayForward?ligandId=2156) are predicted for the mature mouse and human peptides [http://www.ncbi.nlm.nih.gov/pubmed/18710417?dopt=AbstractPlus]. UT exhibits relatively high sequence identity with somatostatin, opioid and galanin receptors [http://www.ncbi.nlm.nih.gov/pubmed/25535277?dopt=AbstractPlus].

**Table:** 

Nomenclature	http://www.guidetopharmacology.org/GRAC/ObjectDisplayForward?objectId=365
HGNC, UniProt	https://www.genenames.org/data/gene‐symbol‐report/#!/hgnc_id/HGNC:4468, http://www.uniprot.org/uniprot/Q9UKP6
Endogenous agonists	http://www.guidetopharmacology.org/GRAC/LigandDisplayForward?ligandId=2156 (https://www.genenames.org/data/gene‐symbol‐report/#!/hgnc_id/HGNC:30894, http://www.uniprot.org/uniprot/Q765I0) [http://www.ncbi.nlm.nih.gov/pubmed/18710417?dopt=AbstractPlus, http://www.ncbi.nlm.nih.gov/pubmed/11015293?dopt=AbstractPlus], http://www.guidetopharmacology.org/GRAC/LigandDisplayForward?ligandId=2153 (https://www.genenames.org/data/gene‐symbol‐report/#!/hgnc_id/HGNC:12636, http://www.uniprot.org/uniprot/O95399) [http://www.ncbi.nlm.nih.gov/pubmed/15852036?dopt=AbstractPlus, http://www.ncbi.nlm.nih.gov/pubmed/11976263?dopt=AbstractPlus, http://www.ncbi.nlm.nih.gov/pubmed/12238917?dopt=AbstractPlus]
Selective agonists	[http://www.guidetopharmacology.org/GRAC/LigandDisplayForward?ligandId=2160) [http://www.ncbi.nlm.nih.gov/pubmed/12238917?dopt=AbstractPlus], http://www.guidetopharmacology.org/GRAC/LigandDisplayForward?ligandId=2152], [http://www.guidetopharmacology.org/GRAC/LigandDisplayForward?ligandId=9436) [http://www.ncbi.nlm.nih.gov/pubmed/12943190?dopt=AbstractPlus], http://www.guidetopharmacology.org/GRAC/LigandDisplayForward?ligandId=9441 [http://www.ncbi.nlm.nih.gov/pubmed/27791374?dopt=AbstractPlus], http://www.guidetopharmacology.org/GRAC/LigandDisplayForward?ligandId=3501 [http://www.ncbi.nlm.nih.gov/pubmed/19481466?dopt=AbstractPlus, http://www.ncbi.nlm.nih.gov/pubmed/17112638?dopt=AbstractPlus], http://www.guidetopharmacology.org/GRAC/LigandDisplayForward?ligandId=2151 [http://www.ncbi.nlm.nih.gov/pubmed/12408704?dopt=AbstractPlus, http://www.ncbi.nlm.nih.gov/pubmed/15781415?dopt=AbstractPlus]
Selective antagonists	http://www.guidetopharmacology.org/GRAC/LigandDisplayForward?ligandId=2167 (p*K*_i_ 8.3) [http://www.ncbi.nlm.nih.gov/pubmed/14645137?dopt=AbstractPlus], [http://www.guidetopharmacology.org/GRAC/LigandDisplayForward?ligandId=9438 (p*K*_i_ 7.2) [http://www.ncbi.nlm.nih.gov/pubmed/18082287?dopt=AbstractPlus] – Rat, http://www.guidetopharmacology.org/GRAC/LigandDisplayForward?ligandId=3516 (pIC_50_ 7.1) [http://www.ncbi.nlm.nih.gov/pubmed/15146030?dopt=AbstractPlus], http://www.guidetopharmacology.org/GRAC/LigandDisplayForward?ligandId=3531 (p*K*_i_ 6.6) [http://www.ncbi.nlm.nih.gov/pubmed/16171813?dopt=AbstractPlus], [http://www.guidetopharmacology.org/GRAC/LigandDisplayForward?ligandId=9439) (p*K*_i_ 6.4) [http://www.ncbi.nlm.nih.gov/pubmed/17125276?dopt=AbstractPlus] – Rat
Labelled ligands	[http://www.guidetopharmacology.org/GRAC/LigandDisplayForward?ligandId=2158) (Agonist) [http://www.ncbi.nlm.nih.gov/pubmed/10499587?dopt=AbstractPlus, http://www.ncbi.nlm.nih.gov/pubmed/1546952?dopt=AbstractPlus, http://www.ncbi.nlm.nih.gov/pubmed/17125276?dopt=AbstractPlus, http://www.ncbi.nlm.nih.gov/pubmed/11015293?dopt=AbstractPlus], [http://www.guidetopharmacology.org/GRAC/LigandDisplayForward?ligandId=9440) [http://www.ncbi.nlm.nih.gov/pubmed/22044114?dopt=AbstractPlus]

### Comments

In the human vasculature, human http://www.guidetopharmacology.org/GRAC/LigandDisplayForward?ligandId=2153 (https://www.genenames.org/data/gene‐symbol‐report/#!/hgnc_id/HGNC:12636, http://www.uniprot.org/uniprot/O95399) elicits both vasoconstrictor (p*D*_2_ 9.3‐10.1, [http://www.ncbi.nlm.nih.gov/pubmed/11015293?dopt=AbstractPlus]) and vasodilator (pIC_50_ 10.3‐10.4, [http://www.ncbi.nlm.nih.gov/pubmed/11158995?dopt=AbstractPlus]) responses.

### Further reading on Urotensin receptor

Foord SM *et al.* (2005) International Union of Pharmacology. XLVI. G protein‐coupled receptor list. *Pharmacol Rev* **57:** 279‐288[https://www.ncbi.nlm.nih.gov/pubmed/15914470?dopt=AbstractPlus]

Hunt BD *et al.* (2010) A rat brain atlas of urotensin‐II receptor expression and a review of central urotensin‐II effects. *Naunyn Schmiedebergs Arch. Pharmacol.* **382**:1‐31[https://www.ncbi.nlm.nih.gov/pubmed/20422157?dopt=AbstractPlus]

Maryanoff BE *et al.* (2010) Urotensin‐II receptor modulators as potential drugs. *J. Med. Chem.* **53:** 2695‐708[https://www.ncbi.nlm.nih.gov/pubmed/20043680?dopt=AbstractPlus]

Ross B *et al.* (2010) Role of urotensin II in health and disease. *Am.J.Physiol.Regul.Integr.Comp. Physiol.* **298:** R1156‐72[https://www.ncbi.nlm.nih.gov/pubmed/20421634?dopt=AbstractPlus]

Vaudry H *et al.* (2015) International Union of Basic and Clinical Pharmacology. XCII. Urotensin II, urotensin II‐related peptide, and their receptor: from structure to function. *Pharmacol. Rev.* **67:** 214‐58[https://www.ncbi.nlm.nih.gov/pubmed/25535277?dopt=AbstractPlus]

## http://www.guidetopharmacology.org/GRAC/FamilyDisplayForward?familyId=66

### Overview

Vasopressin (AVP) and oxytocin (OT) receptors (**nomenclature as recommended by NC‐IUPHAR** [http://www.ncbi.nlm.nih.gov/pubmed/15914470?dopt=AbstractPlus]) are activated by the endogenous cyclic nonapeptides http://www.guidetopharmacology.org/GRAC/LigandDisplayForward?ligandId=2168 (https://www.genenames.org/data/gene‐symbol‐report/#!/hgnc_id/HGNC:894, http://www.uniprot.org/uniprot/P01185) and http://www.guidetopharmacology.org/GRAC/LigandDisplayForward?ligandId=2174 (https://www.genenames.org/data/gene‐symbol‐report/#!/hgnc_id/HGNC:8528, http://www.uniprot.org/uniprot/P01178). These peptides are derived from precursors which also produce neurophysins (neurophysin I for oxytocin; neurophysin II for vasopressin). Vasopressin and oxytocin differ at only 2 amino acids (positions 3 and 8). There are metabolites of these neuropeptides that may be biologically active [http://www.ncbi.nlm.nih.gov/pubmed/8258377?dopt=AbstractPlus].

**Table:** 

Nomenclature	http://www.guidetopharmacology.org/GRAC/ObjectDisplayForward?objectId=366	http://www.guidetopharmacology.org/GRAC/ObjectDisplayForward?objectId=367
HGNC, UniProt	https://www.genenames.org/data/gene‐symbol‐report/#!/hgnc_id/HGNC:895, http://www.uniprot.org/uniprot/P37288	https://www.genenames.org/data/gene‐symbol‐report/#!/hgnc_id/HGNC:896, http://www.uniprot.org/uniprot/P47901
Potency order of endogenous ligands	http://www.guidetopharmacology.org/GRAC/LigandDisplayForward?ligandId=2168 (https://www.genenames.org/data/gene‐symbol‐report/#!/hgnc_id/HGNC:894, http://www.uniprot.org/uniprot/P01185) > http://www.guidetopharmacology.org/GRAC/LigandDisplayForward?ligandId=2174 (https://www.genenames.org/data/gene‐symbol‐report/#!/hgnc_id/HGNC:8528, http://www.uniprot.org/uniprot/P01178) [http://www.ncbi.nlm.nih.gov/pubmed/10519430?dopt=AbstractPlus, http://www.ncbi.nlm.nih.gov/pubmed/15084136?dopt=AbstractPlus, http://www.ncbi.nlm.nih.gov/pubmed/10866830?dopt=AbstractPlus, http://www.ncbi.nlm.nih.gov/pubmed/10780976?dopt=AbstractPlus, http://www.ncbi.nlm.nih.gov/pubmed/9264324?dopt=AbstractPlus, http://www.ncbi.nlm.nih.gov/pubmed/9884074?dopt=AbstractPlus, http://www.ncbi.nlm.nih.gov/pubmed/8106369?dopt=AbstractPlus]	http://www.guidetopharmacology.org/GRAC/LigandDisplayForward?ligandId=2168 (https://www.genenames.org/data/gene‐symbol‐report/#!/hgnc_id/HGNC:894, http://www.uniprot.org/uniprot/P01185) > http://www.guidetopharmacology.org/GRAC/LigandDisplayForward?ligandId=2174 (https://www.genenames.org/data/gene‐symbol‐report/#!/hgnc_id/HGNC:8528, http://www.uniprot.org/uniprot/P01178) [http://www.ncbi.nlm.nih.gov/pubmed/10519430?dopt=AbstractPlus, http://www.ncbi.nlm.nih.gov/pubmed/15084136?dopt=AbstractPlus, http://www.ncbi.nlm.nih.gov/pubmed/12446593?dopt=AbstractPlus, http://www.ncbi.nlm.nih.gov/pubmed/16158071?dopt=AbstractPlus, http://www.ncbi.nlm.nih.gov/pubmed/10780976?dopt=AbstractPlus, http://www.ncbi.nlm.nih.gov/pubmed/9264324?dopt=AbstractPlus, http://www.ncbi.nlm.nih.gov/pubmed/9884074?dopt=AbstractPlus, http://www.ncbi.nlm.nih.gov/pubmed/9322919?dopt=AbstractPlus]
Selective agonists	http://www.guidetopharmacology.org/GRAC/LigandDisplayForward?ligandId=2171 [http://www.ncbi.nlm.nih.gov/pubmed/11934825?dopt=AbstractPlus, http://www.ncbi.nlm.nih.gov/pubmed/10866830?dopt=AbstractPlus]	http://www.guidetopharmacology.org/GRAC/LigandDisplayForward?ligandId=2191 [http://www.ncbi.nlm.nih.gov/pubmed/17300166?dopt=AbstractPlus], http://www.guidetopharmacology.org/GRAC/LigandDisplayForward?ligandId=2187 [http://www.ncbi.nlm.nih.gov/pubmed/12446593?dopt=AbstractPlus, http://www.ncbi.nlm.nih.gov/pubmed/16158071?dopt=AbstractPlus]
Antagonists	http://www.guidetopharmacology.org/GRAC/LigandDisplayForward?ligandId=2203 (p*K*_i_ 8.2–8.4) [http://www.ncbi.nlm.nih.gov/pubmed/9884074?dopt=AbstractPlus, http://www.ncbi.nlm.nih.gov/pubmed/9459574?dopt=AbstractPlus]	http://www.guidetopharmacology.org/GRAC/LigandDisplayForward?ligandId=2202 (p*K*_i_ 8.4–9.3) [http://www.ncbi.nlm.nih.gov/pubmed/16158071?dopt=AbstractPlus, http://www.ncbi.nlm.nih.gov/pubmed/11861823?dopt=AbstractPlus]
Selective antagonists	http://www.guidetopharmacology.org/GRAC/LigandDisplayForward?ligandId=2200 (p*K*_i_ 8.1–9.3) [http://www.ncbi.nlm.nih.gov/pubmed/10519430?dopt=AbstractPlus, http://www.ncbi.nlm.nih.gov/pubmed/10866830?dopt=AbstractPlus, http://www.ncbi.nlm.nih.gov/pubmed/16158071?dopt=AbstractPlus, http://www.ncbi.nlm.nih.gov/pubmed/9884074?dopt=AbstractPlus, http://www.ncbi.nlm.nih.gov/pubmed/8106369?dopt=AbstractPlus], http://www.guidetopharmacology.org/GRAC/LigandDisplayForward?ligandId=3867 (p*K*_i_ 9)	–
Labelled ligands	http://www.guidetopharmacology.org/GRAC/LigandDisplayForward?ligandId=2207 (Antagonist) (p*K*_d_ 10.3–10.4) [http://www.ncbi.nlm.nih.gov/pubmed/7774575?dopt=AbstractPlus, http://www.ncbi.nlm.nih.gov/pubmed/10866830?dopt=AbstractPlus], http://www.guidetopharmacology.org/GRAC/LigandDisplayForward?ligandId=2175) (Agonist) [http://www.ncbi.nlm.nih.gov/pubmed/11337500?dopt=AbstractPlus, http://www.ncbi.nlm.nih.gov/pubmed/7774575?dopt=AbstractPlus, http://www.ncbi.nlm.nih.gov/pubmed/9884074?dopt=AbstractPlus, http://www.ncbi.nlm.nih.gov/pubmed/9459574?dopt=AbstractPlus, http://www.ncbi.nlm.nih.gov/pubmed/8106369?dopt=AbstractPlus], http://www.guidetopharmacology.org/GRAC/LigandDisplayForward?ligandId=3813 (Antagonist) (p*K*_d_ 9)	http://www.guidetopharmacology.org/GRAC/LigandDisplayForward?ligandId=2175) (Agonist) [http://www.ncbi.nlm.nih.gov/pubmed/12446593?dopt=AbstractPlus, http://www.ncbi.nlm.nih.gov/pubmed/9264324?dopt=AbstractPlus, http://www.ncbi.nlm.nih.gov/pubmed/9884074?dopt=AbstractPlus, http://www.ncbi.nlm.nih.gov/pubmed/9459574?dopt=AbstractPlus, http://www.ncbi.nlm.nih.gov/pubmed/9322919?dopt=AbstractPlus]

**Table:** 

Nomenclature	http://www.guidetopharmacology.org/GRAC/ObjectDisplayForward?objectId=368	http://www.guidetopharmacology.org/GRAC/ObjectDisplayForward?objectId=369
HGNC, UniProt	https://www.genenames.org/data/gene‐symbol‐report/#!/hgnc_id/HGNC:897, http://www.uniprot.org/uniprot/P30518	https://www.genenames.org/data/gene‐symbol‐report/#!/hgnc_id/HGNC:8529, http://www.uniprot.org/uniprot/P30559
Potency order of endogenous ligands	http://www.guidetopharmacology.org/GRAC/LigandDisplayForward?ligandId=2168 (https://www.genenames.org/data/gene‐symbol‐report/#!/hgnc_id/HGNC:894, http://www.uniprot.org/uniprot/P01185) > http://www.guidetopharmacology.org/GRAC/LigandDisplayForward?ligandId=2174 (https://www.genenames.org/data/gene‐symbol‐report/#!/hgnc_id/HGNC:8528, http://www.uniprot.org/uniprot/P01178) [http://www.ncbi.nlm.nih.gov/pubmed/10519430?dopt=AbstractPlus, http://www.ncbi.nlm.nih.gov/pubmed/15084136?dopt=AbstractPlus, http://www.ncbi.nlm.nih.gov/pubmed/7774575?dopt=AbstractPlus, http://www.ncbi.nlm.nih.gov/pubmed/17300166?dopt=AbstractPlus, http://www.ncbi.nlm.nih.gov/pubmed/11012895?dopt=AbstractPlus, http://www.ncbi.nlm.nih.gov/pubmed/9884074?dopt=AbstractPlus, http://www.ncbi.nlm.nih.gov/pubmed/9864265?dopt=AbstractPlus]	http://www.guidetopharmacology.org/GRAC/LigandDisplayForward?ligandId=2174 (https://www.genenames.org/data/gene‐symbol‐report/#!/hgnc_id/HGNC:8528, http://www.uniprot.org/uniprot/P01178) > http://www.guidetopharmacology.org/GRAC/LigandDisplayForward?ligandId=2168 (https://www.genenames.org/data/gene‐symbol‐report/#!/hgnc_id/HGNC:894, http://www.uniprot.org/uniprot/P01185) [http://www.ncbi.nlm.nih.gov/pubmed/10519430?dopt=AbstractPlus, http://www.ncbi.nlm.nih.gov/pubmed/7774575?dopt=AbstractPlus, http://www.ncbi.nlm.nih.gov/pubmed/8955347?dopt=AbstractPlus, http://www.ncbi.nlm.nih.gov/pubmed/12660315?dopt=AbstractPlus, http://www.ncbi.nlm.nih.gov/pubmed/16158071?dopt=AbstractPlus, http://www.ncbi.nlm.nih.gov/pubmed/7475979?dopt=AbstractPlus, http://www.ncbi.nlm.nih.gov/pubmed/7921228?dopt=AbstractPlus]
Selective agonists	http://www.guidetopharmacology.org/GRAC/LigandDisplayForward?ligandId=3537 [http://www.ncbi.nlm.nih.gov/pubmed/16297621?dopt=AbstractPlus], http://www.guidetopharmacology.org/GRAC/LigandDisplayForward?ligandId=2173 [http://www.ncbi.nlm.nih.gov/pubmed/10780976?dopt=AbstractPlus], http://www.guidetopharmacology.org/GRAC/LigandDisplayForward?ligandId=3869	[http://www.guidetopharmacology.org/GRAC/LigandDisplayForward?ligandId=2179 [http://www.ncbi.nlm.nih.gov/pubmed/8955347?dopt=AbstractPlus, http://www.ncbi.nlm.nih.gov/pubmed/2827511?dopt=AbstractPlus, http://www.ncbi.nlm.nih.gov/pubmed/7475979?dopt=AbstractPlus]
Antagonists	–	http://www.guidetopharmacology.org/GRAC/LigandDisplayForward?ligandId=2252 (p*K*_i_ 8.8) [http://www.ncbi.nlm.nih.gov/pubmed/16158071?dopt=AbstractPlus]
Selective antagonists	http://www.guidetopharmacology.org/GRAC/LigandDisplayForward?ligandId=2203 (p*K*_i_ 9.4) [http://www.ncbi.nlm.nih.gov/pubmed/20471258?dopt=AbstractPlus], http://www.guidetopharmacology.org/GRAC/LigandDisplayForward?ligandId=2226 (p*K*_i_ 9.4) [http://www.ncbi.nlm.nih.gov/pubmed/9864265?dopt=AbstractPlus], http://www.guidetopharmacology.org/GRAC/LigandDisplayForward?ligandId=2199 (p*K*_i_ 8.4–9.3) [http://www.ncbi.nlm.nih.gov/pubmed/10519430?dopt=AbstractPlus, http://www.ncbi.nlm.nih.gov/pubmed/9792651?dopt=AbstractPlus, http://www.ncbi.nlm.nih.gov/pubmed/8981918?dopt=AbstractPlus, http://www.ncbi.nlm.nih.gov/pubmed/11012895?dopt=AbstractPlus, http://www.ncbi.nlm.nih.gov/pubmed/9884074?dopt=AbstractPlus], http://www.guidetopharmacology.org/GRAC/LigandDisplayForward?ligandId=2238 (Inverse agonist) (p*K*_i_ 8.9–9.2) [http://www.ncbi.nlm.nih.gov/pubmed/9651149?dopt=AbstractPlus], http://www.guidetopharmacology.org/GRAC/LigandDisplayForward?ligandId=2236 (p*K*_i_ 6.9–8.4) [http://www.ncbi.nlm.nih.gov/pubmed/9792651?dopt=AbstractPlus], http://www.guidetopharmacology.org/GRAC/LigandDisplayForward?ligandId=2197 (Inverse agonist) (p*K*_i_ 7.4–8.1) [http://www.ncbi.nlm.nih.gov/pubmed/9792651?dopt=AbstractPlus, http://www.ncbi.nlm.nih.gov/pubmed/9322919?dopt=AbstractPlus]	http://www.guidetopharmacology.org/GRAC/LigandDisplayForward?ligandId=2201 (p*K*_i_ 8.8–9.1) [http://www.ncbi.nlm.nih.gov/pubmed/14722330?dopt=AbstractPlus], http://www.guidetopharmacology.org/GRAC/LigandDisplayForward?ligandId=3870 (p*K*_i_ 8.5), http://www.guidetopharmacology.org/GRAC/LigandDisplayForward?ligandId=2253 (p*K*_i_ 8.4) [http://www.ncbi.nlm.nih.gov/pubmed/9622556?dopt=AbstractPlus]
Labelled ligands	http://www.guidetopharmacology.org/GRAC/LigandDisplayForward?ligandId=2175) (Agonist) [http://www.ncbi.nlm.nih.gov/pubmed/9792651?dopt=AbstractPlus, http://www.ncbi.nlm.nih.gov/pubmed/10780976?dopt=AbstractPlus, http://www.ncbi.nlm.nih.gov/pubmed/9884074?dopt=AbstractPlus, http://www.ncbi.nlm.nih.gov/pubmed/9459574?dopt=AbstractPlus, http://www.ncbi.nlm.nih.gov/pubmed/9864265?dopt=AbstractPlus], http://www.guidetopharmacology.org/GRAC/LigandDisplayForward?ligandId=2228 (Agonist) [http://www.ncbi.nlm.nih.gov/pubmed/2964362?dopt=AbstractPlus], [http://www.guidetopharmacology.org/GRAC/LigandDisplayForward?ligandId=3817 (p*K*_d_ 8.6)	[http://www.guidetopharmacology.org/GRAC/LigandDisplayForward?ligandId=3778 (Antagonist) (p*K*_d_ 10), http://www.guidetopharmacology.org/GRAC/LigandDisplayForward?ligandId=2176) (Agonist) [http://www.ncbi.nlm.nih.gov/pubmed/7774575?dopt=AbstractPlus, http://www.ncbi.nlm.nih.gov/pubmed/6278592?dopt=AbstractPlus, http://www.ncbi.nlm.nih.gov/pubmed/7475979?dopt=AbstractPlus, http://www.ncbi.nlm.nih.gov/pubmed/7921228?dopt=AbstractPlus], [http://www.guidetopharmacology.org/GRAC/LigandDisplayForward?ligandId=3756 (p*K*_d_ 8.3) [http://www.ncbi.nlm.nih.gov/pubmed/12942128?dopt=AbstractPlus]

### Comments

Vasopressin and oxytocin receptors have a characteristic and sometimes overlapping distribution in a number of tissues including brain. There are phylogenetic, ontogenetic and sex‐specific differences in the levels and distribution of these receptors, particularly in the brain. The V_2_ receptor exhibits marked species differences, such that many ligands (http://www.guidetopharmacology.org/GRAC/LigandDisplayForward?ligandId=2236 and [http://www.guidetopharmacology.org/GRAC/LigandDisplayForward?ligandId=3817) exhibit low affinity at human V_2_ receptors [http://www.ncbi.nlm.nih.gov/pubmed/9773787?dopt=AbstractPlus]. Similarly, http://www.guidetopharmacology.org/GRAC/LigandDisplayForward?ligandId=2170 is more V_2_ selective in the rat than in the human [http://www.ncbi.nlm.nih.gov/pubmed/9264324?dopt=AbstractPlus]. The gene encoding the V_2_ receptor is polymorphic in man, underlying nephrogenic diabetes insipidus [http://www.ncbi.nlm.nih.gov/pubmed/9756088?dopt=AbstractPlus]. http://www.guidetopharmacology.org/GRAC/LigandDisplayForward?ligandId=2187 is selective only for the human and bovine V_1_B receptors [http://www.ncbi.nlm.nih.gov/pubmed/12446593?dopt=AbstractPlus], while http://www.guidetopharmacology.org/GRAC/LigandDisplayForward?ligandId=2191 has high affinity for the rat V_1_B receptor [http://www.ncbi.nlm.nih.gov/pubmed/17300166?dopt=AbstractPlus]. Knockouts of vasopressin and oxytocin receptors have systemspecific defects (e.g., impaired ability toconcentrate urine in V_2_ receptor knockouts) which include behavioural deficits (principally in V_1_A^, V^_1_B and OT receptor knockouts).

### Further reading on Vasopressin and oxytocin receptors

Knepper MA. (2012) Systems biology in physiology: the vasopressin signaling network in kidney. *Am. J. Physiol., Cell Physiol.* **303:** C1115‐24[https://www.ncbi.nlm.nih.gov/pubmed/22932685?dopt=AbstractPlus]

Koshimizu TA *et al.* (2012) Vasopressin V1a and V1b receptors: from molecules to physiological systems. *Physiol. Rev.* **92:** 1813‐64[https://www.ncbi.nlm.nih.gov/pubmed/23073632?dopt=AbstractPlus]

Manning M *et al.* (2012) Oxytocin and vasopressin agonists and antagonists as research tools and potential therapeutics. *J. Neuroendocrinol.* **24:** 609‐28[https://www.ncbi.nlm.nih.gov/pubmed/22375852?dopt=AbstractPlus]

Meyer‐Lindenberg A *et al.* (2011) Oxytocin and vasopressin in the human brain: social neuropeptides for translational medicine. *Nat. Rev. Neurosci.* **12:** 524‐38[https://www.ncbi.nlm.nih.gov/pubmed/21852800?dopt=AbstractPlus]

Neumann ID *et al.* (2012) Balance of brain oxytocin and vasopressin: implications for anxiety, depression, and social behaviors. *Trends Neurosci.* **35:** 649‐59[https://www.ncbi.nlm.nih.gov/pubmed/22974560?dopt=AbstractPlus]

## http://www.guidetopharmacology.org/GRAC/FamilyDisplayForward?familyId=67

### Overview

Vasoactive intestinal peptide (VIP) and pituitary adenylate cyclase‐activating peptide (PACAP) receptors (**nomenclature as agreed by the NC‐IUPHAR Subcommittee on Vasoactive Intestinal Peptide Receptors** [http://www.ncbi.nlm.nih.gov/pubmed/9647867?dopt=AbstractPlus, http://www.ncbi.nlm.nih.gov/pubmed/22289055?dopt=AbstractPlus]) are activated by the endogenous peptides http://www.guidetopharmacology.org/GRAC/LigandDisplayForward?ligandId=1152 (https://www.genenames.org/data/gene‐symbol‐report/#!/hgnc_id/HGNC:12693, http://www.uniprot.org/uniprot/P01282), http://www.guidetopharmacology.org/GRAC/LigandDisplayForward?ligandId=2258 (https://www.genenames.org/data/gene‐symbol‐report/#!/hgnc_id/HGNC:241, http://www.uniprot.org/uniprot/P18509), http://www.guidetopharmacology.org/GRAC/LigandDisplayForward?ligandId=2257), peptide histidine isoleucineamide (http://www.guidetopharmacology.org/GRAC/LigandDisplayForward?ligandId=4397 {Mouse, Rat}), peptide histidine methionineamide (http://www.guidetopharmacology.org/GRAC/LigandDisplayForward?ligandId=2274)) and peptide histidine valine (http://www.guidetopharmacology.org/GRAC/LigandDisplayForward?ligandId=3706)). VPAC_1_ and VPAC_2_ receptors display comparable affinity for the PACAP peptides, http://www.guidetopharmacology.org/GRAC/LigandDisplayForward?ligandId=2257) and http://www.guidetopharmacology.org/GRAC/LigandDisplayForward?ligandId=2258), and http://www.guidetopharmacology.org/GRAC/LigandDisplayForward?ligandId=1152 (https://www.genenames.org/data/gene‐symbol‐report/#!/hgnc_id/HGNC:12693, http://www.uniprot.org/uniprot/P01282), whereas http://www.guidetopharmacology.org/GRAC/LigandDisplayForward?ligandId=2257 (https://www.genenames.org/data/gene‐symbol‐report/#!/hgnc_id/HGNC:241, http://www.uniprot.org/uniprot/P18509) and http://www.guidetopharmacology.org/GRAC/LigandDisplayForward?ligandId=2258) are >100 fold more potent than http://www.guidetopharmacology.org/GRAC/LigandDisplayForward?ligandId=1152) as agonists of most isoforms of the PAC_1_ receptor. However, one splice variant of the human PAC_1_ receptor has been reported to respond to http://www.guidetopharmacology.org/GRAC/LigandDisplayForward?ligandId=2258), http://www.guidetopharmacology.org/GRAC/LigandDisplayForward?ligandId=2257) and http://www.guidetopharmacology.org/GRAC/LigandDisplayForward?ligandId=1152) with comparable affinity [http://www.ncbi.nlm.nih.gov/pubmed/10583729?dopt=AbstractPlus]. http://www.guidetopharmacology.org/GRAC/LigandDisplayForward?ligandId=2272 [http://www.ncbi.nlm.nih.gov/pubmed/11068102?dopt=AbstractPlus] has been used as a selective VPAC_2_ receptor antagonist in a number of physiological studies, but has been reported to have significant activity at VPAC_1_ and PAC_1_ receptors [http://www.ncbi.nlm.nih.gov/pubmed/16930633?dopt=AbstractPlus]. The selective PAC_1_ receptor agonist http://www.guidetopharmacology.org/GRAC/LigandDisplayForward?ligandId=2264, was extracted from the salivary glands of sand flies (*Lutzomyia longipalpis*) and has no sequence homology to http://www.guidetopharmacology.org/GRAC/LigandDisplayForward?ligandId=1152) or the PACAP peptides [http://www.ncbi.nlm.nih.gov/pubmed/8995389?dopt=AbstractPlus]. Two deletion variants of http://www.guidetopharmacology.org/GRAC/LigandDisplayForward?ligandId=2264, http://www.guidetopharmacology.org/GRAC/LigandDisplayForward?ligandId=3305 [http://www.ncbi.nlm.nih.gov/pubmed/9928019?dopt=AbstractPlus] and http://www.guidetopharmacology.org/GRAC/LigandDisplayForward?ligandId=2265 [http://www.ncbi.nlm.nih.gov/pubmed/10438479?dopt=AbstractPlus] have been reported to be PAC_1_ receptor antagonists, but these peptides have not been extensively characterised.

**Table:** 

Nomenclature	http://www.guidetopharmacology.org/GRAC/ObjectDisplayForward?objectId=370	http://www.guidetopharmacology.org/GRAC/ObjectDisplayForward?objectId=371	http://www.guidetopharmacology.org/GRAC/ObjectDisplayForward?objectId=372
HGNC, UniProt	https://www.genenames.org/data/gene‐symbol‐report/#!/hgnc_id/HGNC:242, http://www.uniprot.org/uniprot/P41586	https://www.genenames.org/data/gene‐symbol‐report/#!/hgnc_id/HGNC:12694, http://www.uniprot.org/uniprot/P32241	https://www.genenames.org/data/gene‐symbol‐report/#!/hgnc_id/HGNC:12695, http://www.uniprot.org/uniprot/P41587
Potency order of endogenous ligands	http://www.guidetopharmacology.org/GRAC/LigandDisplayForward?ligandId=2257 (https://www.genenames.org/data/gene‐symbol‐report/#!/hgnc_id/HGNC:241, http://www.uniprot.org/uniprot/P18509), http://www.guidetopharmacology.org/GRAC/LigandDisplayForward?ligandId=2258) http://www.guidetopharmacology.org/GRAC/LigandDisplayForward?ligandId=1152 (https://www.genenames.org/data/gene‐symbol‐report/#!/hgnc_id/HGNC:12693, http://www.uniprot.org/uniprot/P01282)	http://www.guidetopharmacology.org/GRAC/LigandDisplayForward?ligandId=1152 (https://www.genenames.org/data/gene‐symbol‐report/#!/hgnc_id/HGNC:12693, http://www.uniprot.org/uniprot/P01282), http://www.guidetopharmacology.org/GRAC/LigandDisplayForward?ligandId=2257 (https://www.genenames.org/data/gene‐symbol‐report/#!/hgnc_id/HGNC:241, http://www.uniprot.org/uniprot/P18509), http://www.guidetopharmacology.org/GRAC/LigandDisplayForward?ligandId=2258) http://www.guidetopharmacology.org/GRAC/LigandDisplayForward?ligandId=2270 (https://www.genenames.org/data/gene‐symbol‐report/#!/hgnc_id/HGNC:4265, http://www.uniprot.org/uniprot/P01286), http://www.guidetopharmacology.org/GRAC/LigandDisplayForward?ligandId=2273 {Pig}, http://www.guidetopharmacology.org/GRAC/LigandDisplayForward?ligandId=3643 (https://www.genenames.org/data/gene‐symbol‐report/#!/hgnc_id/HGNC:10607, http://www.uniprot.org/uniprot/P09683)	http://www.guidetopharmacology.org/GRAC/LigandDisplayForward?ligandId=1152 (https://www.genenames.org/data/gene‐symbol‐report/#!/hgnc_id/HGNC:12693, http://www.uniprot.org/uniprot/P01282), http://www.guidetopharmacology.org/GRAC/LigandDisplayForward?ligandId=2258 (https://www.genenames.org/data/gene‐symbol‐report/#!/hgnc_id/HGNC:241, http://www.uniprot.org/uniprot/P18509), http://www.guidetopharmacology.org/GRAC/LigandDisplayForward?ligandId=2257 (https://www.genenames.org/data/gene‐symbol‐report/#!/hgnc_id/HGNC:241, http://www.uniprot.org/uniprot/P18509) > http://www.guidetopharmacology.org/GRAC/LigandDisplayForward?ligandId=2273 {Pig} ≫ http://www.guidetopharmacology.org/GRAC/LigandDisplayForward?ligandId=2270 (https://www.genenames.org/data/gene‐symbol‐report/#!/hgnc_id/HGNC:4265, http://www.uniprot.org/uniprot/P01286), http://www.guidetopharmacology.org/GRAC/LigandDisplayForward?ligandId=3643 (https://www.genenames.org/data/gene‐symbol‐report/#!/hgnc_id/HGNC:10607, http://www.uniprot.org/uniprot/P09683)
Selective agonists	http://www.guidetopharmacology.org/GRAC/LigandDisplayForward?ligandId=2264 [http://www.ncbi.nlm.nih.gov/pubmed/16930633?dopt=AbstractPlus], http://www.guidetopharmacology.org/GRAC/LigandDisplayForward?ligandId=2264]	[http://www.guidetopharmacology.org/GRAC/LigandDisplayForward?ligandId=3842 [http://www.ncbi.nlm.nih.gov/pubmed/11931347?dopt=AbstractPlus], [http://www.guidetopharmacology.org/GRAC/LigandDisplayForward?ligandId=2278 [http://www.ncbi.nlm.nih.gov/pubmed/10801840?dopt=AbstractPlus]	http://www.guidetopharmacology.org/GRAC/LigandDisplayForward?ligandId=2259 [http://www.ncbi.nlm.nih.gov/pubmed/9145428?dopt=AbstractPlus, http://www.ncbi.nlm.nih.gov/pubmed/10570056?dopt=AbstractPlus, http://www.ncbi.nlm.nih.gov/pubmed/11931347?dopt=AbstractPlus], http://www.guidetopharmacology.org/GRAC/LigandDisplayForward?ligandId=2276 [http://www.ncbi.nlm.nih.gov/pubmed/9152366?dopt=AbstractPlus]
Selective antagonists	–	http://www.guidetopharmacology.org/GRAC/LigandDisplayForward?ligandId=2268 (pIC_50_ 8.7) [http://www.ncbi.nlm.nih.gov/pubmed/9437716?dopt=AbstractPlus, http://www.ncbi.nlm.nih.gov/pubmed/10570056?dopt=AbstractPlus]	–
Labelled ligands	http://www.guidetopharmacology.org/GRAC/LigandDisplayForward?ligandId=2261 (Agonist) [http://www.ncbi.nlm.nih.gov/pubmed/11193823?dopt=AbstractPlus]	http://www.guidetopharmacology.org/GRAC/LigandDisplayForward?ligandId=2277) (Agonist) [http://www.ncbi.nlm.nih.gov/pubmed/10801840?dopt=AbstractPlus], http://www.guidetopharmacology.org/GRAC/LigandDisplayForward?ligandId=2261 (Agonist)	http://www.guidetopharmacology.org/GRAC/LigandDisplayForward?ligandId=2277) (Agonist) [http://www.ncbi.nlm.nih.gov/pubmed/10801840?dopt=AbstractPlus], http://www.guidetopharmacology.org/GRAC/LigandDisplayForward?ligandId=2261 (Agonist)

### Comments

Subtypes of PAC_1_ receptors have been proposed based on tissue differences in the potencies of http://www.guidetopharmacology.org/GRAC/LigandDisplayForward?ligandId=2257 (https://www.genenames.org/data/gene‐symbol‐report/#!/hgnc_id/HGNC:241, http://www.uniprot.org/uniprot/P18509) and http://www.guidetopharmacology.org/GRAC/LigandDisplayForward?ligandId=2258); these might result from differences in G protein coupling and second messenger mechanisms [http://www.ncbi.nlm.nih.gov/pubmed/8967982?dopt=AbstractPlus], or from alternative splicing of PAC_1_ receptor mRNA [http://www.ncbi.nlm.nih.gov/pubmed/8396727?dopt=AbstractPlus].

### Further reading on VIP and PACAP receptors

Harmar AJ *et al.* (1998) International Union of Pharmacology. XVIII. Nomenclature of receptors for vasoactive intestinal peptide and pituitary adenylate cyclase‐activating polypeptide. *Pharmacol Rev* **50:** 265‐270[https://www.ncbi.nlm.nih.gov/pubmed/9647867?dopt=AbstractPlus]

Harmar AJ *et al.* (2012) Pharmacology and functions of receptors for vasoactive intestinal peptide and pituitary adenylate cyclase‐activating polypeptide: IUPHAR review 1. *Br. J. Pharmacol.* **166:** 4‐17[https://www.ncbi.nlm.nih.gov/pubmed/22289055?dopt=AbstractPlus]

Reglodi D *et al.* (2012) Effects of pituitary adenylate cyclase activating polypeptide in the urinary system, with special emphasis on its protective effects in the kidney. *Neuropeptides* **46:** 61‐70[https://www.ncbi.nlm.nih.gov/pubmed/21621841?dopt=AbstractPlus]

Smith CB *et al.* (2012) Is PACAP the major neurotransmitter for stress transduction at the adrenomedullary synapse? *J. Mol. Neurosci.* **48:** 403‐12[https://www.ncbi.nlm.nih.gov/pubmed/22610912?dopt=AbstractPlus]
